# Thiadiazino-indole, thiadiazino-carbazole and benzothiadiazino-carbazole dioxides: synthesis, physicochemical and early ADME characterization of representatives of new tri-, tetra- and pentacyclic ring systems and their intermediates

**DOI:** 10.3762/bjoc.21.169

**Published:** 2025-10-21

**Authors:** Gyöngyvér Pusztai, László Poszávácz, Anna Vincze, András Marton, Ahmed Qasim Abdulhussein, Judit Halász, András Dancsó, Gyula Simig, György Tibor Balogh, Balázs Volk

**Affiliations:** 1 Department of Chemical and Environmental Process Engineering, Faculty of Chemical Technology and Biotechnology, Budapest University of Technology and Economics, Műegyetem rkp. 3, H-1111 Budapest, Hungary; 2 Egis Pharmaceuticals Plc., Directorate of Drug Substance Development, P.O. Box 100, H-1475 Budapest, Hungary; 3 Department of Pharmaceutical Chemistry, Semmelweis University, Hőgyes Endre u. 9, H-1092 Budapest, Hungary; 4 Ambimass Ltd., Záhony u. 7, H-1031 Budapest, Hungary; 5 Center for Pharmacology and Drug Research & Development, Department of Pharmaceutical Chemistry, Semmelweis University, H-1085 Budapest, Hungary

**Keywords:** early ADME characterization, Fischer indole cyclization, heterocycles, indoles, lead-likeness, new ring systems, physicochemical characterization

## Abstract

Motivated by the in vivo anxiolytic activity of previously described 1,2,3-benzothiadiazine 1,1-dioxides, we aimed at elaborating a synthetic procedure for the preparation of their pyrrole-fused counterparts, 2,9-dihydro[1,2,3]thiadiazino[5,6-*g*]indole 1,1-dioxide derivatives. The simple and versatile process led, via Fischer indole cyclization of the corresponding hydrazones, to a wide structural variety of new tri-, tetra- and pentacyclic ring systems. The structural characterization of (*E*)- and (*Z*)-hydrazones was supported by 2D NMR techniques, while that of the target compounds by single-crystal X-ray measurements. The hydrazone intermediates and the new title compounds were subjected to a physicochemical and early ADME characterization study, in the framework of which log*P*, p*K*_a_ and log*k* values were calculated. Following that, kinetic solubility and in vitro gastrointestinal membrane-specific permeability measurements were carried out to assess the lead-likeness of the compounds. Subsequently, the metabolic stability of the most promising derivatives was also determined using human liver microsomes.

## Introduction

**Figure 1:**
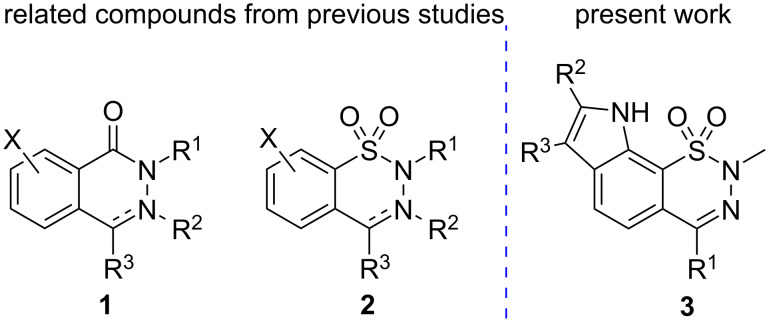
Phthalazinones **1**, benzothiadiazine dioxides **2**, and thiadiazinoindole dioxides **3**.

Considering the published pharmacological activity of phthalazin-1(2*H*)-ones **1** (Figure 1) [1–5], we have devoted significant effort to the synthesis and pharmacological investigation of structurally related 1,2,3-benzothiadiazine 1,1-dioxides **2** over the last decade [6–11]. As 4-methyl-2*H*-1,2,3-benzothiadiazine 1,1-dioxides and their 3,4-dihydro derivatives **2** (R^3^ = Me) were found to have remarkable in vivo anxiolytic activity [12], we aimed to prepare further congeners exhibiting a higher potency in this field. It is well known that synthetic as well as naturally occurring compounds containing an indole moiety exhibit diverse biological activities and have found application for the treatment of psychiatric disorders and neurodegenerative diseases [13–17]. Therefore, we now report our results in the synthesis and characterization of compounds **3** containing a 2,9-dihydro[1,2,3]thiadiazino[5,6-*g*]indole 1,1-dioxide structural element.

## Results and Discussion

**Scheme 1:**
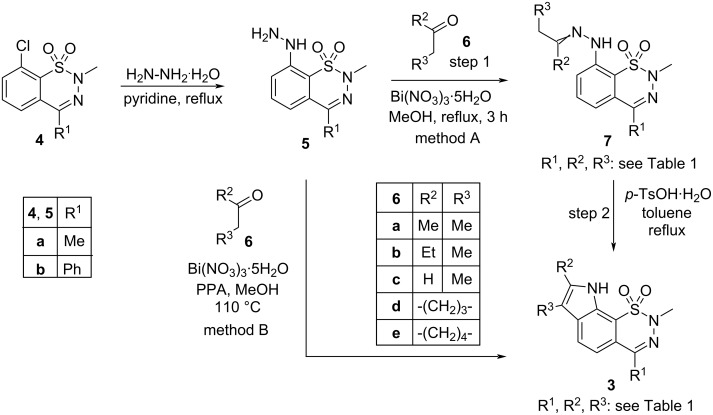
Synthesis of tri- and tetracyclic thiadiazinoindole dioxides **3**.

We synthesized target compounds **3** exhibiting new tricyclic (**3a**–**c**, **3f**–**h**, Scheme 1, Table 1) and tetracyclic (**3d**,**e**,**i**,**j**) ring systems starting from 8-chloro-2,4-dimethyl-2*H*-1,2,3-benzothiadiazine 1,1-dioxide (**4a**) and 8-chloro-2-methyl-4-phenyl-2*H*-1,2,3-benzothiadiazine 1,1-dioxide (**4b**), both described in our earlier publication [8]. Treatment of compounds **4a**,**b** with hydrazine monohydrate afforded 8-hydrazino derivatives **5a**,**b** as suitable starting materials for the construction of the indole structural element of compounds **3** by Fischer indole synthesis [18–21]. Sudhakara et al. described the advantages of using bismuth nitrate as catalyst in the synthesis of hydrazones and in the one-pot Fischer synthesis of indoles from ketones and hydrazines [22–23]. Adopting this method, hydrazone intermediates **7a**–**j** were obtained by treatment of hydrazino derivatives **5a**,**b** with ketones **6a**–**e** in the presence of bismuth nitrate pentahydrate catalyst in refluxing methanol with good to excellent yields (method A, step 1). As regards *cis*–*trans* isomerism, compounds (*E*)-**7a** and (*E*)-**7f** were isolated in high yields (94% and 84%, respectively), however, in the case of **7c** and **7h**, a substantial amount of (*Z*)-isomer was also obtained.

**Table 1:** Yields of the one- and two-step variants of Fischer indole synthesis.

Compound **3**, **7**	R^1^	R^2^	R^3^	Method A yield (%)		Method B yield (%)

				Step 1	Step 2	

**a**	Me	Me	Me	(*E*): 94	65	47
**b**	Me	Et	Me	83	92	50
**c**	Me	H	Me	(*E*): 48	46	30
				(*Z*): 23		
**d**	Me	-(CH_2_)_3_-		92	33	25
**e**	Me	-(CH_2_)_4_-		83	66	67
**f**	Ph	Me	Me	(*E*): 84	70	67
**g**	Ph	Et	Me	88	75	60
**h**	Ph	H	Me	(*E*): 40	50	46
				(*Z*): 15		
**i**	Ph	-(CH_2_)_3_-		94	45	35
**j**	Ph	-(CH_2_)_4_-		92	66	67

In view of the expected similar electronic effect of the sulfonamide and the nitro functional groups on the Fischer indole cyclization, first we applied those reaction conditions for the **7a → 3a** transformation (Scheme 1) which were previously used for the synthesis of 7-nitroindole derivatives: heating in polyphosphoric acid (PPA) at 80 °C [24–25]. However, our attempt was not successful. Experiments with zinc chloride, the most commonly used Lewis acid catalyst in Fischer indole syntheses, also failed under various conditions. Finally, we were able to achieve the Fischer cyclization of compounds **7a**–**j** by using *p*-toluenesulfonic acid monohydrate as catalyst in boiling toluene (method A, step 2) [26–27]. On the other hand, the one-pot synthesis of target compounds **3a**–**j** starting from hydrazino derivatives **5a**,**b** was performed using the method described in the literature [18], i.e., by heating compounds **5a**,**b** with ketones **6a**–**e** in the presence of bismuth nitrate pentahydrate catalyst and PPA in methanol at 110 °C in a closed vial (Scheme 1, method B).

**Figure 2:**
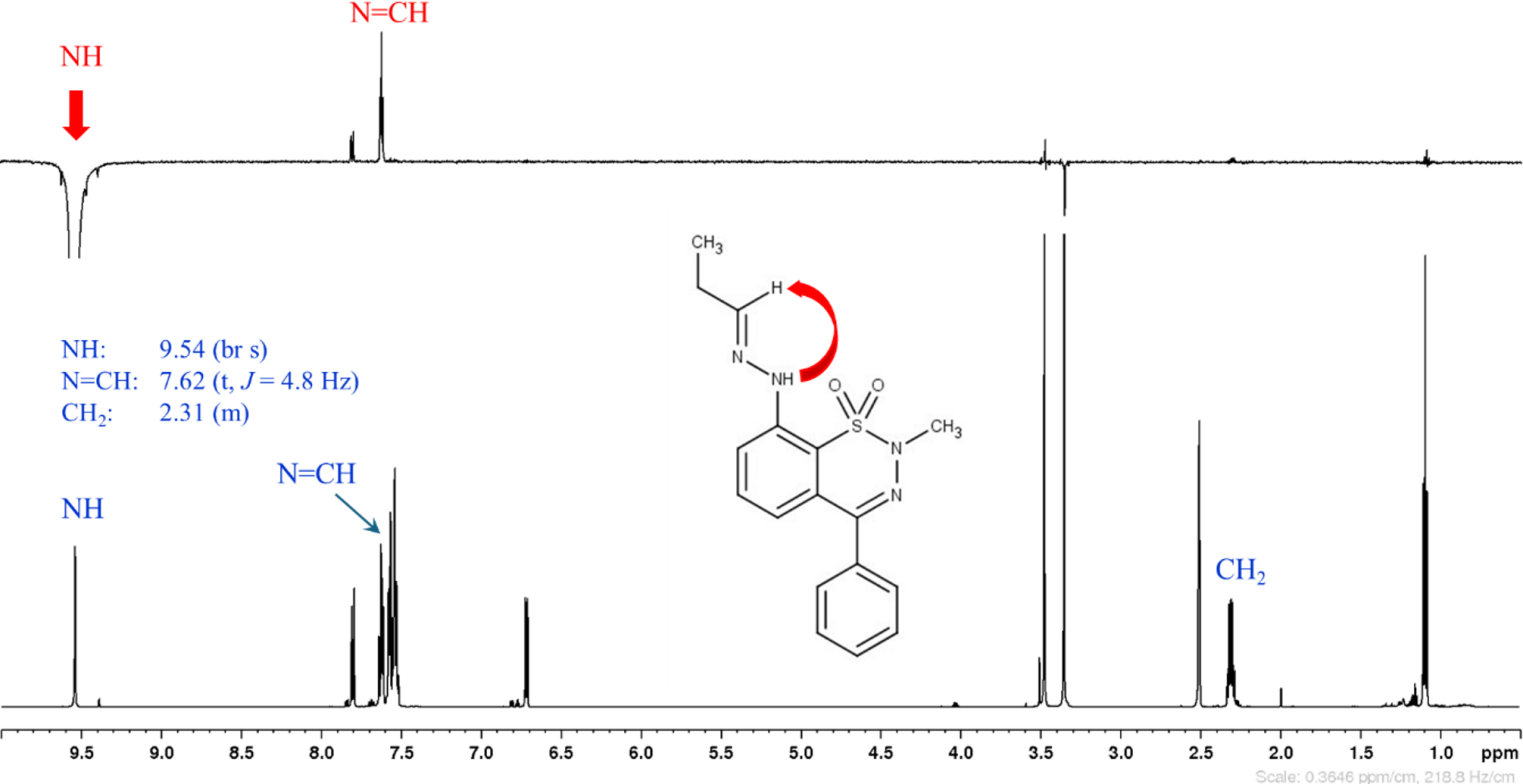
^1^H NMR and selective 1D NOESY (with the excitation of NH) spectra of (*E*)-**7h**.

**Figure 3:**
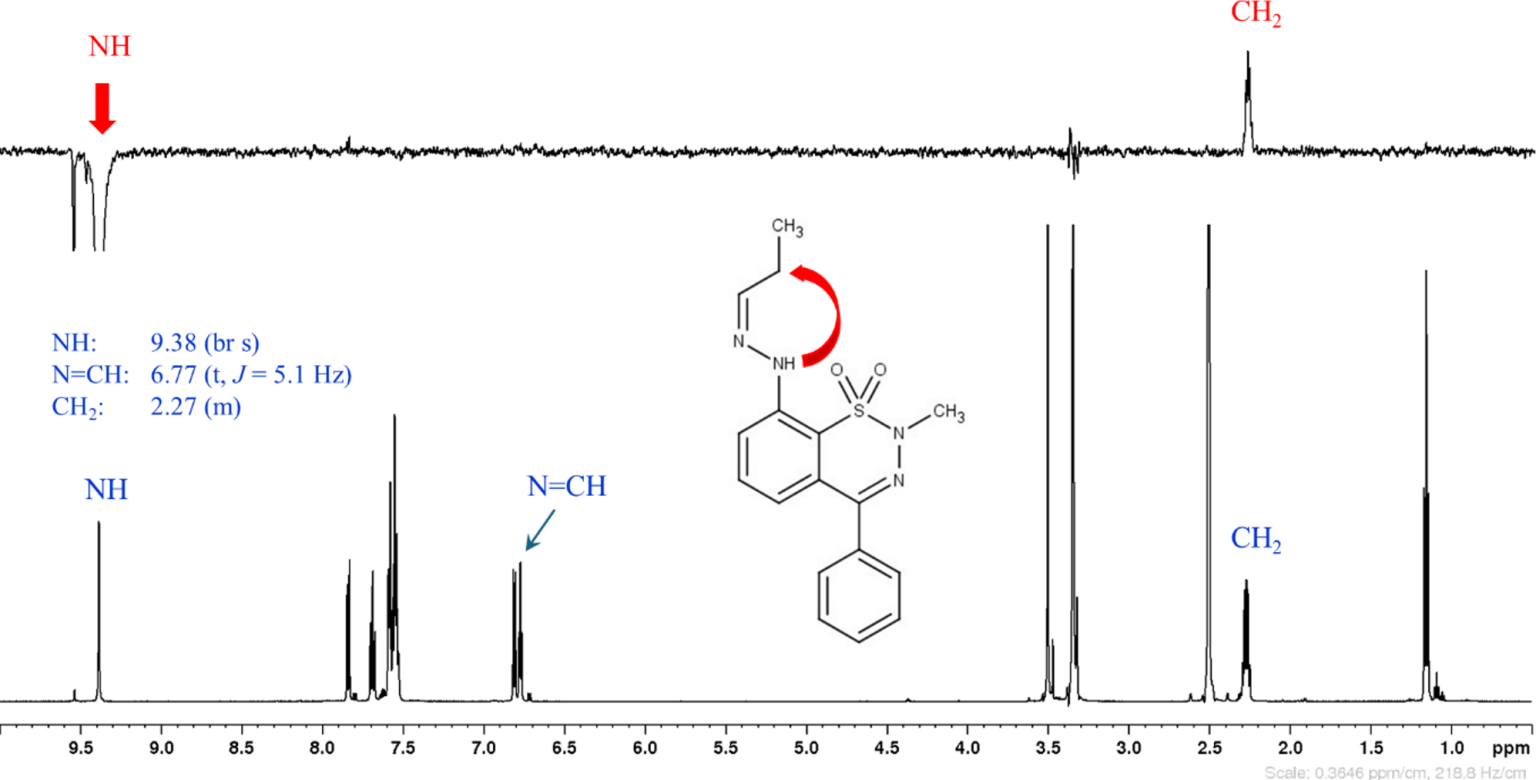
^1^H NMR and selective 1D NOESY (with the excitation of NH) spectra of (*Z*)-**7h**.

In the synthesis of asymmetric hydrazones **7a**,**c**,**f**,**h**, the major product (according to LC–MS) was always the (*E*) isomer. However, in the case of compounds **7c** and **7h**, the (*Z*) isomers were present in relatively high quantities and could also be successfully isolated and characterized after flash chromatography. The configuration of the C=N double bond was determined on the basis of spatial proximities obtained from either NOESY or ROESY NMR measurements. The NH moiety is close to N=CH in the (*E*) isomer, while NH is close to CH_2_ in the (*Z*) isomer. As an example, in case of hydrazone **7h**, a selective 1D NOESY spectrum was recorded with the excitation of NH (Figure 2 and Figure 3). In both Figures, beside the selective 1D NOESY spectrum (upper part in Figure 2 and Figure 3), the ^1^H NMR spectrum (lower part) is also presented. The spatial proximity of NH with N=CH (marked with a red arrow in Figure 2) or with CH_2_ (red arrow in Figure 3) proves the (*E*) or (*Z*) configuration of the N=C double bond, respectively.

**Scheme 2:**
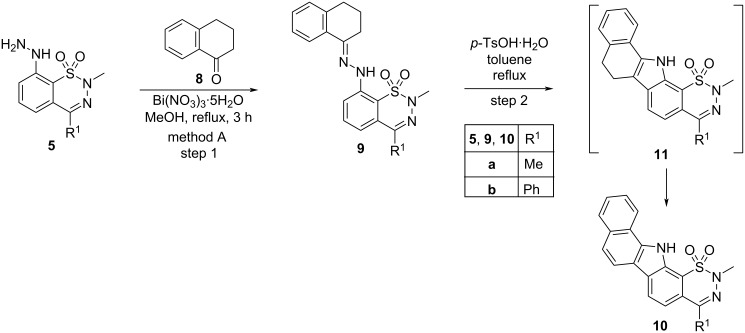
Synthesis of pentacyclic compounds **10**.

Pentacyclic derivatives **10a**,**b** were also prepared in the same way as compounds **3** above, starting from hydrazines **5a**,**b**, in two steps (Scheme 2). Treatment of compounds **5a**,**b** with 1-tetralone (**8**) in the presence of bismuth nitrate pentahydrate in refluxing methanol (method A, step 1) afforded hydrazones (*E*)-**9a**,**b**. According to LC–MS, only the (*E*) isomers were present in the reaction mixtures and in the crude products. Hydrazones (*E*)-**9a**,**b** were then cyclized by refluxing in toluene in the presence of *p*-toluenesulfonic acid monohydrate (method A, step 2). It is noteworthy that derivatives **10a**,**b** exhibiting an extended aromatic ring system were isolated instead of the expected primarily formed congeners **11a**,**b**, due to in situ oxidation of the C–C bond.

Alternatively, when the one-pot method (method B, bismuth nitrate pentahydrate + PPA, MeOH, closed vial, 110 °C) was applied for the reaction of compounds **5a**,**b** with ketone **8**, a crude mixture of pentacyclic derivatives **10** and **11** was obtained (**10a**/**11a** = 50:50 and **10b**/**11b** = 46:54, respectively). Under these conditions, a full conversion to the oxidized product **10** could not be reached even after long reaction times, presumably because the closed vial in which the reaction is carried out does not contain the necessary amount of oxygen.

**Figure 4:**
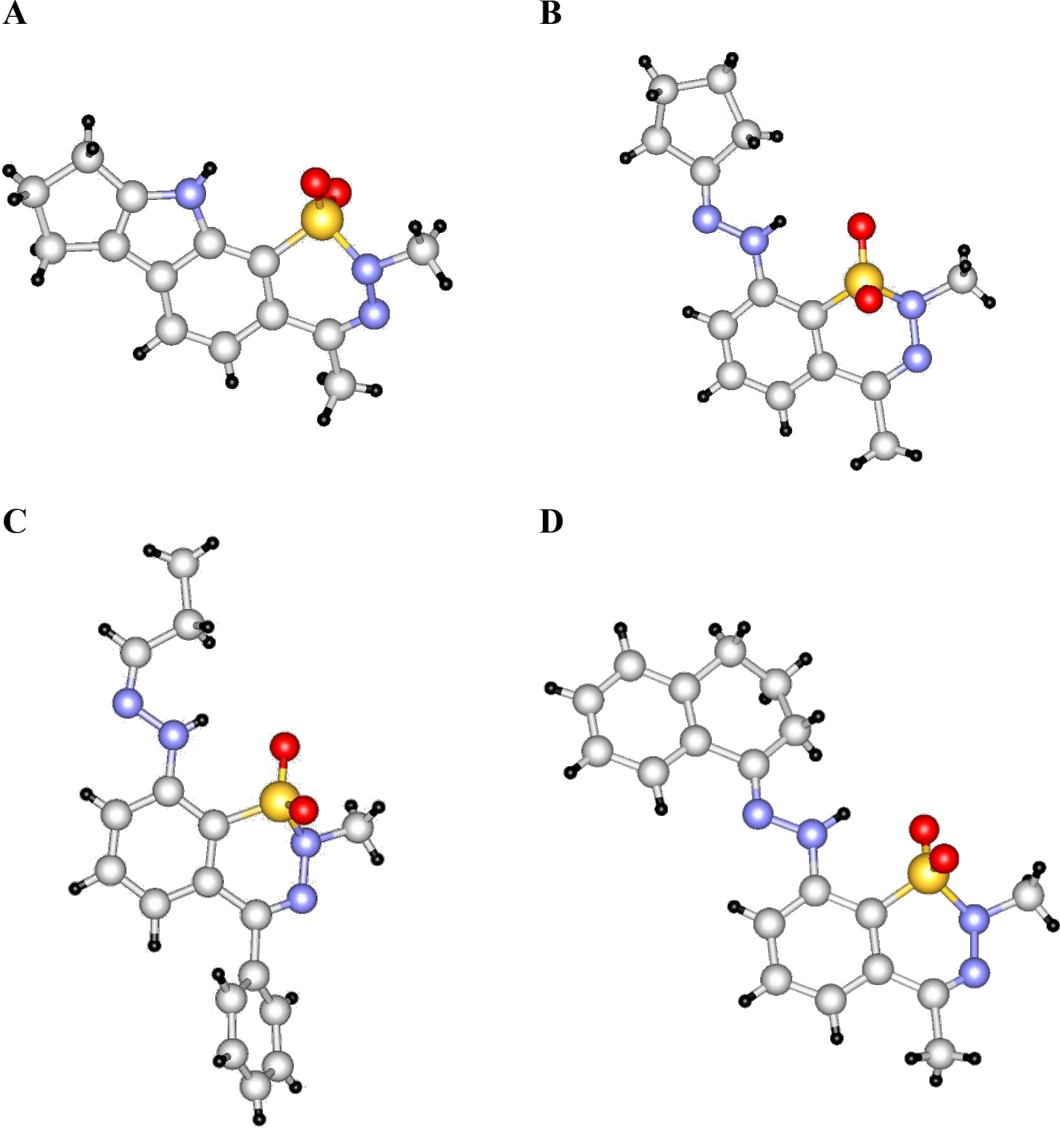
X-ray structures of compounds **3d** (**A**), **7d** (**B**), (*Z*)-**7h** (**C**), and (*E*)-**9a** (**D**).

Structure determination of the products was also supported by single-crystal X-ray diffraction in the case of several representatives: **3b**, **3d**, **3e**, **3g**, **3h**, (*E*)-**7a**, **7b**, **7d**, **7e**, (*E*)-**7f**, (*Z*)-**7h**, **7i**, and (*E*)-**9a**. Among these, **3d**, **7d**, (*Z*)-**7h** and (*E*)-**9a** are shown in Figure 4.

To assess the lead-likeness of the newly synthesized compounds **3a**–**j** and **10a**,**b** and to make a priority list among them prior to pharmacological testing, a detailed early-phase pharmacokinetic evaluation has been carried out. Since the phenylhydrazone structural unit is present in several marketed drugs (herombopag [28], eltrombopag [29], levosimendan [30]) and in drug candidates that reached the human clinical trials (totrombopag [31], FP-21399 [32], sivifene [33]), hydrazone intermediates **7a**–**j** and (*E*)-**9a**,**b** were also involved in the tests.

The results of physicochemical and early ADME characterization for hydrazones **7** and **9**, and target compounds **3** and **10** are summarized in Table 2 and Table 3, respectively (individual column charts on kinetic solubility, permeability, and membrane retention measurements can be found in Supporting Information File 1, Figures S1–S3). Based on their calculated physicochemical properties, all compounds satisfied Lipinski’s Rule of Five suggested by Wager et al. [34] (molecular weight < 500 Da, log*P* < 5, number of hydrogen-bond donors < 5, number of hydrogen-bond acceptors < 10) [35], and the topological polar surface area (TPSA) values fell in a desirable range (40–90 Å^2^) (see the extended table on physicochemical parameters in Supporting Information File 1, Table S1). Based on their p*K*_a_ values, all compounds are non-ionizable or slightly basic. Kinetic solubility was considered good above 30 µM, moderate between 10 µM and 30 µM, and poor under 10 µM. Compounds with no value were under the detection limit. As a result, seven compounds had good solubilities: (*E*)-**7c**, (*Z*)-**7c**, **7d**, **7e**, **3b**, **3c**, and **3e**. We also carried out a gastrointestinal parallel artificial membrane permeability assay (GI PAMPA). The permeability (*P*_e_) results were considered good above 10 × 10^−6^ cm/s, moderate under 10 × 10^−6^ cm/s and low, when no substance was detected on the acceptor side. Compounds with low kinetic solubility were not measured in the permeability assay. As a result, four compounds presented good permeability: (*E*)-**7a**, (*E*)-**7c**, (*Z*)-**7c** and **3c**. GI PAMPA membrane retention (MR) was considered moderate under 80%, elevated between 80% and 90%, while extremely high above 90%. MR represents the affinity of the compound to the phospholipids as a basic component of the biological membrane. A high MR is considered a toxicological risk, as the strongly bound compounds affect membrane integrity, thus influencing the conformation of receptors and possibly causing side effects.

**Table 2:** Summary of physicochemical and early ADME characterization (measured parameters are presented as means of 3 replicates) of hydrazones **7** and **9**.

Compound	clog*P* [36]	cp*K*_a_ (basic) [36]	No. of rings	log*k* (−) pH 7.0	Kinetic solubility (µM)	GI PAMPA penetration^a^ (10^−6^ cm/s)/ GI PAMPA membrane retention (%)	HLM *t*_1/2_ (min)/ Cl_int_ (μL/min/kg)^b^

(*E*)-**7a**	2.13	3.37	2	0.547	20.5 ± 0.8	33.6 ± 3.0/ 61.0 ± 5.1	34 ± 1/ 20 ± 0.7
**7b**	2.44	3.37	2	0.625	6.2 ± 0.3	–	–
(*E*)-**7c**	1.75	2.46	2	0.579	77.2 ± 2.2	24.0 ± 1.4/ 90.9 ± 0.7	28 ± 1/ 24.2 ± 1.1
(*Z*)-**7c**	1.75	2.46	2	0.570	78.4 ± 2.2	21.8 ± 4.0/ 92.0 ± 3.6	30.3 ± 1.8/ 22.4 ± 0.7
**7d**	1.75	3.37	3	0.599	46.9 ± 2.8	7.6 ± 2.9/ 93.3 ± 0.2	31 ± 3/ 22.3 ± 2.1
**7e**	2.28	3.11	3	0.626	86.3 ± 3.9	0.0/ 97.5 ± 0.3	–
(*E*)-**7f**	3.26	3.29	3	0.662	0.8 ± 0.11	–	–
**7g**	3.57	3.29	3	0.677	–	–	–
crude **7h**	2.89	2.38	3	0.645	0.7 ± 0.02	–	–
**7i**	2.94	3.72	4	0.661	2.2 ± 0.1	–	–
**7j**	3.15	3.72	4	0.660	2.8 ± 0.2	–	–
(*E*)-**9a**	2.96	1.49	4	0.653	–	–	–
(*E*)-**9b**	4.11	1.41	5	0.695	–	–	–

^a^GI PAMPA: Gastrointestinal-specific parallel artificial membrane permeability assay. ^b^HLM *t*_1/2_/Cl_int_: in vitro metabolic half-life and intrinsic clearance determined by human liver microsome system.

**Table 3:** Summary of physicochemical and early ADME characterization (measured parameters are presented as means of 3 replicates) of indolothiadiazines **3** and **10**.

Compound	clog*P* [36]	No. of rings	log*k* (–) pH 7.0	Kinetic solubility (µM)	GI PAMPA penetration^a^ (10^−6^ cm/s)/ GI PAMPA membrane retention (%)	HLM *t*_1/2_ (min)/ Cl_int_ (μL/min/kg)^b^

**3a**	1.93	3	0.580	1.5 ± 0.09	–	–
**3b**	2.28	3	0.603	31.0 ± 2.2	0.0/ 98.5 ± 0.1	–
**3c**	1.55	3	0.567	41.4 ± 0.9	30.3 ± 4.2/ 39.8 ± 0.2	29 ± 2/ 23.6 ± 1.5
**3d**	1.86	4	0.591	3.2 ± 0.2	–	–
**3e** *^c^*	2.26	4	0.516	51.4 ± 1.3	7.1 ± 1.7/ 82.3 ± 0.8	100 ± 23/ 6.8 ± 1.7
**3f**	3.26	4	0.629	5.0 ± 0.3	–	–
**3g**	2.94	4	0.648	0.7 ± 0.03	–	–
**3h**	2.49	4	0.614	8.6 ± 0.3	–	–
**3i**	3.62	5	0.645	1.7 ± 0.3	–	–
**3j**	3.77	5	0.660	–	–	–
**10a**	3.56	5	0.637	–	–	–
**10b**	4.91	6	0.685	–	–	–

^a^GI PAMPA: gastrointestinal-specific parallel artificial membrane permeability assay. ^b^HLM *t*_1/2_/Cl_int_: in vitro metabolic half-life and intrinsic clearance determined by human liver microsome system. ^c^Selected candidate considering low Cl_int_ (increased metabolic stability).

As the outcome of pharmacokinetic characterization, lead-likeness was interpreted in Table 2 and Table 3, considering kinetic solubility, GI PAMPA permeability and MR. Compounds (*E*)-**7a** and **3c** with moderate to good kinetic solubility, good permeability, and moderate MR were labelled as primary leads, i.e., promising molecules for further development. Hydrazone isomers (*E*)-**7c** and (*Z*)-**7c** were categorized as secondary leads because, despite the good solubility and permeability values, their high MR limited their potential. As expected, these geometrical isomers performed almost identically in each assay, thus supporting the robustness of the models used. Similarly, derivatives **7d** and **3e** were classified as secondary leads, due to their moderate permeability and high MR. Finally, despite having good solubility, compounds **7e** and **3b** were labelled only as backup leads, demonstrating nearly zero permeability as a consequence of their extremely high MR. From a pharmacokinetic point of view, the rest of the compounds were discarded, since they did not meet the basic criterion of moderate kinetic solubility required for further biological assays.

**Figure 5:**
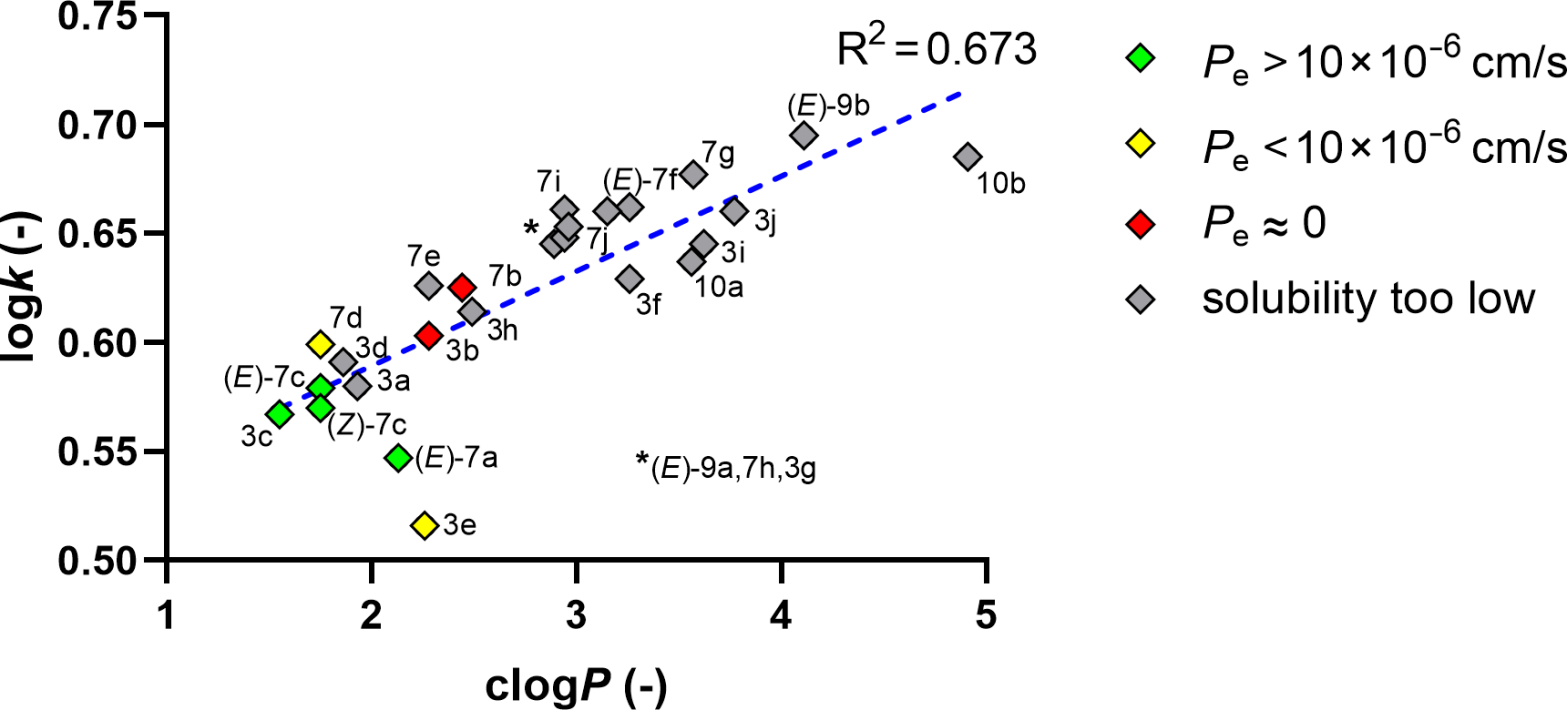
The capacity factor (log*k*) vs calculated partition coefficients (clog*P*) by ACD Labs/Percepta [36]); the coloring indicates the permeability categories of the compounds.

As in some cases the calculated log*P* (clog*P*) values did not align with the observed solubility and permeability trends, the reversed-phase chromatographic capacity factor (log*k*) was involved in further analysis. When correlating clog*P* and log*k* in Figure 5, the fitted line showed a moderate linear correlation (R^2^ = 0.673). According to the plot, the partition coefficients of the outliers (*E*)-**7a**, **3e**, and **10b** seemed to be overestimated by the Percepta software [36]. The lipophilicity of hydrazone (*E*)-**7a** was estimated closer to the structurally similar compound **7b** in silico, however, according to the log*k* value, it should be in the lower right corner also supported by its permeability value (green category). Derivative **10b** is clearly an extremity among these compounds, being the only molecule containing 6 rings, it is likely the reason for the in silico overestimation. Removal of the outliers would have resulted in a substantially better correlation with R^2^ = 0.835 (see Supporting Information File 1). The positions of the colored datapoints clearly show that the least lipophilic compounds had the best permeability values, meanwhile, most of the gray datapoints with insufficient solubility scattered in the right side of the plot, indicating their high lipophilicity.

As part of the discussion on structure–property relationships, most of the compounds with low kinetic solubility showed similarities in their structure. It can be clearly seen that the majority of these compounds have at least 4 rings (Table 2). Moreover, all derivatives with a phenyl moiety in position 4 fell into this category (**3f**–**j**, **7f**–**j**, (*E*)-**9b**, **10b**) obviously making the 4-phenyl substituent undesirable. The solubility of compounds is also influenced by their tendency to form thermodynamically stable crystals since disruption of such stable structures is more challenging [37–42]. Molecules with symmetric elements and plane rings typically tend to form such stable crystals. The notable solubility difference of hydrazones **7a** and **7c** compared to **7b** is likely due to the structural differences, with **7a** and **7c** having asymmetric substitutions (methyl for **7c**, R^2^ = H, R^3^ = Me and ethyl, methyl for **7a**, R^2^ = Me, R^3^ = Me), while **7b** having a symmetric diethyl substitution pattern (R^2^ = Et, R^3^ = Me). The comparison of the solubility of compound **3a** to that of less symmetric derivatives **3b** and **3c** leads to a similar conclusion. When comparing compounds **3e** and **3d**, it can be suggested that the cyclohexyl ring in **3e** with its higher flexibility is a more plane-disrupting structural element than the cyclopentyl ring in **3d**.

After the initial physicochemical characterization, we conducted standardized metabolic stability assessments on pooled human liver microsome (HLM) isolates of the primary and secondary lead compounds identified during the solubility and permeability studies. The measured values are summarized in the last column of Table 2 and Table 3. The preliminary ADME investigation, characterized by first-order kinetics due to low substrate concentration (≤10 µM), aids in establishing intrinsic clearance (Cl_int_), which indicates the predicted elimination efficacy of drugs [43]. According to the generally accepted Cl_int_ ≈ 10 μL/min/kg (or half-life: *t*_1/2_ ≈ 60 min) threshold, compound **3e** qualifies as a suitable candidate for subsequent medicinal chemistry investigation [44]. The outcome somewhat elucidates the distinctive behavior of **3e** in the aforementioned clog*P*–log*k* relationship: despite the expected elevated clog*P*, we obtained a reduced measured log*k* value, which diverges from this class of compounds. This finding indicates a lower lipophilicity of **3e**, supported by the observation that a reduced log*P* value is typically linked to a decreased liver microsomal metabolism and any hepatotoxicity-related side effects [45]. Despite compound **3e** being classified only as a secondary lead from initial in vitro permeability (GI PAMPA) screening, the relevant HLM study indicated that the enhanced membrane integrity (MR) and penetration of other selected derivatives also improved integration with the microsome, potentially linked to an accelerated hepatic clearance (Cl_int_) due to the metabolic sensitivity of this class of compounds. Thus, a strategy for additional lead optimization towards central nervous system drug candidates, commencing with compound **3e**, is advised based on the preliminary ADME data.

## Conclusion

The aforementioned synthetic process led, via Fischer indole cyclization of the corresponding hydrazones, to several novel 2,9-dihydro[1,2,3]thiadiazino[5,6-*g*]indole 1,1-dioxide derivatives, containing new tri-, tetra- and pentacyclic ring systems. The hydrazone intermediates and new target compounds were investigated regarding their physicochemical and ADME parameters. Based on literature analogy with related compounds, furthermore on calculated properties, and results from kinetic solubility, in vitro membrane permeability and metabolic stability measurements, a tetracyclic derivative, 2,4-dimethyl-2,7,8,9,10,11-hexahydro[1,2,3]thiadiazino[6,5-*a*]carbazole 1,1-dioxide (**3e**) has been identified as a promising central nervous system drug candidate for pharmacological testing and eventual further structure–activity optimization.

## Experimental

Compounds **5a**, **5b**, **7a**–**j**, (*E*)-**9a**, (*E*)-**9b**, **3a**–**j**, **10a**, **10b** are new and characterized in detail either below (**5a**, **5b**, **7e**, (*E*)-**7h**, (*Z*)-**7h**, **3e**, **3h**) or in Supporting Information File 1 ((*E*)-**7a**, **7b**, (*E*)-**7c**, (*Z*)-**7c**, **7d**, (*E*)-**7f**, **7g**, **7i**, **7j**, (*E*)-**9a**, (*E*)-**9b**, **3a**–**d**, **3f**, **3g**, **3i**, **3j**, **10a**, **10b**). Melting points were determined using a Büchi B-540 melting point apparatus. IR spectra were obtained on a Bruker ALPHA FT-IR spectrometer in KBr pellets, ν̃ was reported in cm^−1^. NMR spectra were recorded at 295 K on a Bruker Avance III HD 600 (600 and 150 MHz for ^1^H and ^13^C NMR spectra, respectively) spectrometer equipped with a Prodigy cryo-probe head. Full ^1^H and ^13^C assignments were achieved using widely accepted strategies [46–47]. DMSO-*d*_6_ or CDCl_3_ were used as the solvents and tetramethylsilane (TMS) as the internal standard. Chemical shifts (δ) and coupling constants (*J*) are given in ppm and in Hz, respectively. High-resolution mass spectra were recorded on an Agilent 2750 GC/Q-TOF mass spectrometer equipped with a Direct Insertion Probe (EI^+^ ionization) or on a Bruker Q-TOF Maxis Impact mass spectrometer (ESI^+^ ionization) coupled with a Waters Acquity I-Class UPLC system equipped with a diode array detector. The reactions were followed by analytical thin-layer chromatography on silica gel 60 F_254_ and by UHPLC–MS on a Shimadzu LC-40 UHPLC equipment equipped with a quaternary pump, degasser, autosampler, column oven, diode array detector, and an LCMS-2020 quadrupole mass spectrometer. Single-crystal X-ray diffraction (SC-XRD) measurements were carried out on a Rigaku R-Axis Spider diffractometer with an imaging plate area detector using graphite monochromatic Cu Kα radiation. Single crystal X-ray structures were deposited at the Cambridge Crystallographic Data Centre under the following numbers: CCDC 2447633 (**3b**), CCDC 2447638 (**3d**), CCDC 2447632 (**3e**), CCDC 2447636 (**3g**), CCDC 2447639 (**3h**), CCDC 2447635 [(*E*)-**7a**], CCDC 2447644 (**7b**), CCDC 2447641 (**7d**), CCDC 2447634 (**7e**), CCDC 2447643 [(*E*)-**7f**], CCDC 2447642 [(*Z*)-**7h**], CCDC 2447640 (**7i**) and CCDC 2447637 [(*E*)-**9a**].

All solvents used in the pharmacokinetic characterization and for HPLC measurements were of analytical grade, purchased from Merck KGaA (Darmstadt, Germany). For gastrointestinal permeability studies, ʟ-α-phosphatidylcholine and cholesterol were purchased from Sigma-Aldrich (Merck KGaA, Darmstadt, Germany). Phosphate buffered saline (0.01 M, pH 6.5 and 7.4) was prepared from pre-mixed PBS powder also sold by Sigma-Aldrich (Merck KGaA, Darmstadt, Germany), adjusting the pH with HCl. Water for buffer and eluent preparation were provided by a Millipore Milli-Q water purification system.

**Kinetic aqueous solubility assay.** All compounds were dissolved in DMSO to make stock solutions at 10 mM concentration. In the case of **7i**, **7j**, and (*E*)-**9b**, the highest concentration achieved in DMSO was 0.5 mM, while for (*E*)-**9a** it was 1 mM (DMSO solubility results of the compounds can be found in Supporting Information File 1). 5 μL of the stock solutions was pipetted into a 96-well plate containing 495 μL PBS (pH 6.5) in each well to achieve 100 μM target concentration (1% DMSO). In the case of **7i**, **7j**, (*E*)-**9a**, and (*E*)-**9b**, samples were prepared with 50 μM (1% DMSO) and 100 μM (10% DMSO) target concentration, respectively. The plate was then sealed and shaken for 2 hours at room temperature. Each compound was investigated in 3 replicates. After 2 hours, the samples were filtered on a Multriscreen_HTS_ filter plate (MSSLBPC, polycarbonate membrane, Merck KGaA, Darmstadt, Germany) by a Millipore Vacuum Manifold (Merck KGaA, Darmstadt, Germany). Before analyzing, 20 vol % acetonitrile was added to each well to avoid sample precipitation during HPLC analysis.

**In vitro gastrointestinal permeability assay.** For in vitro GI PAMPA permeability studies, only compounds with a kinetic solubility higher than 10 µM were chosen. Like in the kinetic solubility assay, 5 μL of DMSO stock solutions was pipetted into a 96-well plate containing 495 μL PBS (pH 6.5) in each well to achieve 100 μM target concentration (1% DMSO). These samples were shaken for 1 hour at room temperature then filtered the same way as the kinetic solubility samples to make initial solutions for the PAMPA assay. The artificial membrane was fabricated by dissolving ʟ-α-phosphatidylcholine (16 mg) and cholesterol (4 mg) in a solvent mixture of chloroform, hexane and dodecane (5/70/25 vol %). Each well of the PAMPA donor plate (MAIPNTR, PVDF membrane, Merck KGaA, Darmstadt, Germany) was coated with 5 μL lipid solution. Thereafter the donor plate was fitted into the acceptor plate (MSSACCEPTOR, Merck KGaA, Darmstadt, Germany) already containing 300 μL PBS (pH 7.4) solutions with 1% DMSO. Finally, 150 μL of the initial solutions was pipetted into the donor plate, the plate sandwich was covered with a wet paper tissue and with a plate lid to avoid evaporation, and the system was incubated at 37 °C for 4 hours. Then samples were taken from the donor and acceptor wells and analyzed along with the initial solutions by HPLC. Effective permeability and membrane retention were calculated with Equation 1 and Equation 2 as suggested by Avdeef [48]:


[1] $$P_{\text{e}}=\frac{-2.303}{A\cdot (t-\tau_{\text{ss}})}\cdot \left(\frac{1}{1+r_{\text{a}}}\right)\cdot \mathrm{lg}\left[-r_{\text{a}}+\left(\frac{1+r_{\text{a}}}{1-\text{MR}}\right)\cdot \frac{c_{\text{D}}(t)}{c_{\text{D}}(0)}\right]$$

[2] $$\text{MR}=1-\frac{c_{\text{D}}(t)}{c_{\text{D}}(0)}-\frac{V_{\text{A}}c_{\text{A}}(t)}{V_{\text{D}}c_{\text{D}}(0)}$$

where *A* is the filter area (0.24 cm^2^), *t* is the incubation time (s), τ_ss_ is the time to reach steady-state (s), *r*_a_ is the sink asymmetry ratio approximated with a value of 10^−14^, *V*_D_ and *V*_A_ are the volumes in the donor (0.15 mL) and the acceptor phase (0.3 mL), *c*_D_(t) is the concentration of the compound in the donor phase at time point *t* (mol/L), *c*_D_(0) is the concentration of the compound in the donor phase at time point zero (initial solutions, mol/L), *c*_A_(*t*) is the concentration of the compound in the acceptor phase at time point *t* (mol/L), *c*_A_(0) is the concentration of the compound in the acceptor phase at time point zero (mol/L).

**Analytical methods.** All samples of the kinetic solubility and in vitro permeability assays were analyzed on a Waters Alliance 2695 Separations Module equipped with a 2996 PDA detector, using a Water Xterra RP 18 chromatographic column (100 × 4.6 mm, 3.5 µm) at 45 °C, applying 1.2 mL/min flow rate. For a 7-minute long gradient program, eluents A (0.1% formic acid in water) and B (0.1% formic acid in acetonitrile) were used as follows: initial conditions with 30% B were kept for 1 minute, then 90% B was achieved within 3 minutes, kept for further 1 minute, finally the initial condition was adjusted and equilibrated for further 2 minutes. 5-Point calibration series were measured from each compound resulting in R^2^ ≈ 0.9990–0.9999.

**Determination of the capacity factor.** The capacity factor was calculated using the RP-HPLC retention times of each compound at a pH where their ionization was completely suppressed (cp*K*_a_ values can be found in Table 2). The same analytical method was used with modified eluents: eluent A was 20 mM triethylamine with phosphoric acid (pH 7.0), and eluent B was acetonitrile without additives. The method time was slightly extended, with a 1-minute longer gradient, resulting in an 8-minute program. The capacity factor was calculated with Equation 3:


[3] $$\log k=\log\left(\frac{t_{R}-t_{0}}{t_{0}}\right)$$

where *k* is the capacity factor, *t*_R_ is the retention time (min) of the compounds and *t*_0_ is the dead-time of the column (min).

**Metabolic stability assay in human liver microsome fractions.** The metabolic stability of the test compounds was assessed in vitro using pooled liver microsome fractions derived from humans (Xenotech LLC). The assay was conducted in a 96-well plate format according to a standardized protocol, utilizing cofactor-supplemented incubation systems, mainly to mimic phase I oxidative metabolic pathways. Human liver microsome (HLM) fractions were thawed on ice and diluted in 0.05 M potassium phosphate buffer (pH 7.4) to a final protein concentration of 1.25 mg/mL. Test compounds were dissolved in DMSO to create stock solutions of 5 mM. The drug concentration in the final assays was 10 μM. The assay was performed in triplicate. Reaction mixtures containing the diluted HLM fractions and test compounds were pre-incubated for 5 minutes at 37 °C while shaking at 450 rpm. The reaction was initiated by adding the 0.05 M NADPH cofactor mixture to the wells (100 µL per well) for the “with cofactors” conditions or by adding phosphate buffer for the “without cofactors” control. Aliquots (100 µL) were taken at zero time and at pre-defined time points of 30 and 60 minutes and quenched with an equal volume of cold acetonitrile. The samples were centrifuged at 2600 RCF for 10 minutes to pellet proteins. Supernatants were transferred to LC vials and stored at −20 °C until LC–MS/MS analysis. The concentration of the parent compound remaining in each sample was determined using liquid chromatography coupled with tandem mass spectrometry (LC–MS/MS). The percentage of the remaining substrate was calculated relative to the time-zero sample. The natural logarithm of the percentage remaining was plotted against incubation time to determine the first-order elimination rate constant (*k*) using linear regression. The half-life (*t*₁/₂) was calculated using Equation 4:


[4] $$t_{1/2}=\frac{\ln2}{-k}$$

Intrinsic clearance (Cl_int_) was calculated and scaled to in vivo conditions using Equation 5:


[5] $$\begin{array}{c} {\text{Cl}}_{\mathrm{int}}=\frac{\ln2}{t_{1/2}}\times \frac{\text{liver weight (g)}}{\text{standard body weight (kg)}}\\ \times \frac{\text{incubation volume (mL)}}{\frac{\text{HLM protein}}{\text{well}}}\times \frac{\text{HLM protein (mg)}}{\text{gram of liver}} \end{array}$$

where the HLM protein per gram of liver was equal to 25 and the liver weight per standard body was equal to 49. Testosterone was included as a positive control to confirm metabolic competence of the HLM fractions, with and without cofactors. Zero-time samples served as 100% reference for clearance calculations.

**HPLC–MS analysis.** The semi-quantitative determination of test compounds was carried out using a Waters Xevo G2-XS QToF mass spectrometer equipped with an electrospray ionization (ESI) source operating in positive ion mode. Chromatographic separation was performed on a Waters XBridgeTM Premier BEH C18 (3.5 μm, 4.6 × 150 mm) column maintained at 40 °C. The mobile phase consisted of water with 0.1% formic acid (A) and acetonitrile with 0.1% formic acid (B). The injection volume was 10 µL. The autosampler was maintained at 5 °C. The gradient elution program shown in Table 4 was applied at a constant flow rate of 1.0 mL/min.

**Table 4:** Gradient elution program.

Time (min)	A (%)	B (%)

0.0	95	5
1.5	95	5
12.0	20	80
13.0	10	90
13.5	10	90
13.7	95	5
17.5	95	5

The mass spectrometer operated in sensitivity mode with a capillary voltage of 3.0 kV, source temperature was 100 °C, and desolvation temperature was 350 °C. The cone gas flow was set to 0 L/h and desolvation gas flow to 800 L/h. LockSpray calibration was performed using leucine enkephalin as the reference compound (*m*/*z* 556.2771), introduced at 15 µL/min. Data acquisition was performed in MSE mode with alternating low collision energy (6 eV) and ramped high-energy collision dissociation (20–40 eV). The mass range was set from 50 to 1200 Da. Each function used a scan time of 0.5 s. Data were acquired in the continuum mode. Instrument mass accuracy calibration was verified using sodium formate. The system was controlled with a MassLynx v4.2 SCN996 software.

**8-Hydrazino-2,4-dimethyl-2*H*-1,2,3-benzothiadiazine 1,1-dioxide (5a).** To a mixture of 8-chloro-2,4-dimethyl-2*H*-benzo[*e*][1,2,3]thiadiazine 1,1-dioxide (**4a**, 7.10 g, 29.0 mmol) and pyridine (80 mL), hydrazine monohydrate (14.1 mL, 14.5 g, 290 mmol) was added dropwise. After refluxing for 26 h, the reaction mixture was evaporated to dryness, the residue was triturated with water, filtered and washed with water to give **5a** (6.56 g, 94%) as a yellow powder. Mp 160.5–161.5 °C (EtOH); ^1^H NMR (600 MHz, DMSO-*d*_6_) δ 7.64 (m, 2H), 7.21 (br s, 1H), 7.00 (m, 1H), 4.51 (br s, 2H), 3.33 (s, 3H), 2.40 (s, 3H); ^13^C{^1^H} NMR (150 MHz, DMSO-*d*_6_) δ 149.6, 147.3, 134.1, 129.1, 116.0, 114.6, 112.9, 34.7, 20.5; IR (KBr) ν̃: 3407, 3360, 1477, 1302, 1151, 1106 cm^−1^; HREIMS (*m*/*z*): [M^•^]^+^ calcd for C_9_H_12_N_4_O_2_S, 240.0675; found, 240.0677.

**8-Hydrazino-2-methyl-4-phenyl-2*H*-1,2,3-benzothiadiazine 1,1-dioxide (5b).** To a mixture of **4b** (4.00 g, 13.0 mmol) and pyridine (36 mL), hydrazine monohydrate (6.30 mL, 6.50 g, 130.4 mmol) was added dropwise. After refluxing for 26 h, the reaction mixture was evaporated to dryness, the residue was triturated with water, filtered and washed with water to give **5b** (3.65 g, 93%) as a yellow powder. Mp 165–166 °C (EtOH); ^1^H NMR (600 MHz, DMSO-*d*_6_) δ 7.67 (d, *J* = 8.4 Hz, 1H), 7.58 (m, 1H), 7.53 (m, 5H), 7.32 (br s, 1H), 6.54 (dd, *J* = 7.7, 0.9 Hz, 1H), 4.55 (br s, 2H), 3.45 (s, 3H); ^13^C{^1^H} NMR (150 MHz, DMSO-*d*_6_) δ 152.6, 147.4, 135.2, 133.9, 12 9.9, 129.0 (2C), 128.8 (2C), 128.6, 116.4, 116.2, 113.5, 35.2; IR (KBr) ν̃: 3407, 3374, 3335, 1467, 1300, 1155, 1111 cm^−1^; HREIMS (*m*/*z*): [M^•^]^+^ calcd for C_14_H_14_N_4_O_2_S, 302.0837; found, 302.0827.

**General procedure for the synthesis of hydrazones 7a–e, (*E*)-9a** [22–23]. ***Method A, step 1:*** A suspension of **5a** (100 mg, 0.416 mmol), the corresponding ketone **6a–e** or **8** (1.1 equiv) and bismuth(III) nitrate pentahydrate (44.4 mg, 0.092 mmol) in MeOH (1 mL) was refluxed for 3 h, then it was poured into ice-water. The precipitate was filtered and washed with water, or in the case of an oily product, the mixture was extracted with EtOAc (3 × 20 mL), washed with water (15 mL) and brine (15 mL), dried over MgSO_4_ and evaporated to give crude products.

**8-(2-Cyclohexylidenehydrazino)-2,4-dimethyl-2*H*-1,2,3-benzothiadiazine 1,1-dioxide (7e).** Prepared according to the general procedure, using **5a** and **6e** as the starting materials. Yield: 111.0 mg (83%), yellow crystals. Mp 99–100 °C (EtOH); ^1^H NMR (600 MHz, DMSO-*d*_6_) δ 9.19 (br s, 1H), 7.80 (d, *J* = 8.5 Hz, 1H), 7.71 (m, 1H), 7.19 (dd, *J* =7.7, 0.8 Hz, 1H), 3.37 (s, 3H), 2.44 (s, 3H), 2.34 (m, 2H), 2.32 (m, 2H), 1.66 (m, 4H), 1.61 (m, 2H); ^13^C{^1^H} NMR (150 MHz, DMSO-*d*_6_) δ 154.9, 149.9, 141.3, 134.6, 129.2, 116.6, 116.5, 113.2, 34.8 (2C), 26.7, 26.0, 25.4, 25.2, 20.4; IR (KBr) ν̃: 3368, 1598, 1569, 1475, 1299, 1152 cm^−1^; HRESIMS (*m*/*z*): [M + H]^+^ calcd for C_15_H_21_N_4_O_2_S, 321.1385; found, 321.1377.

**General procedure for the synthesis of hydrazones 7f–j, (*E*)-9b** [22–23]. A suspension of **5b** (100 mg, 0.331 mmol), the corresponding ketone **6a–e** or **8** (1.1 equiv) and bismuth(III) nitrate pentahydrate (35.4 mg, 0.073 mmol) in MeOH (1 mL) was refluxed for 3 h, then it was poured into ice-water. The precipitate was filtered and washed with water, or in the case of an oily product, the mixture was extracted with EtOAc (3 × 20 mL), washed with water (15 mL) and brine (15 mL), dried over MgSO_4_ and evaporated to give the crude products.

**2-Methyl-4-phenyl-8-[(2*E*)-2-propylidenehydrazino]-2*H*-1,2,3-benzothiadiazine 1,1-dioxide [(*E*)-7h].** Prepared according to the general procedure, using **5b** and **6c** as the starting materials. Yield: 102.0 mg (90%), crude **7h**, mixture of *E* and *Z* isomers in a 6:4 ratio (according to LC–MS). Pure (*E*)-**7h** was obtained by purification using flash chromatography (hexane–EtOAc). Yield: 45 mg (40%), colorless crystals. Mp 138–139 °C (EtOH); ^1^H NMR (600 MHz, DMSO-*d*_6_) δ 9.54 (br s, 1H), 7.80 (d, *J* = 8.6 Hz, 1H), 7.62 (m, 2H), 7.57 (m, 2H), 7.54 (m, 3H), 6.71 (d, *J* = 7.7 Hz, 1H), 3.47 (s, 3H), 2.31 (m, 2H), 1.09 (t, *J* = 7.4 Hz, 3H); ^13^C{^1^H} NMR (150 MHz, DMSO-*d*_6_) δ 152.8, 149.0, 141.1, 135.0, 134.2, 130.0, 129.1 (2C), 128.9 (2C), 128.8, 118.4, 117.3, 114.0, 35.2, 25.5, 10.9; IR (KBr) ν̃: 3328, 1595, 1470, 1320, 1299, 1160, 1109 cm^−1^; HREIMS (*m/z*): [M^•^]^+^ calcd for C_17_H_18_N_4_O_2_S, 342.1156; found, 342.1152.

**2-Methyl-4-phenyl-8-[(2*Z*)-2-propylidenehydrazino]-2*H*-1,2,3-benzothiadiazine 1,1-dioxide [(*Z*)-7h].** Prepared according to the general procedure, using **5b** and **6c** as the starting materials. Yield: 102.0 mg (90%), crude **7h**, mixture of *E* and *Z* isomers in a 6:4 ratio (according to LC–MS). Pure (*Z*)-**7h** was obtained by purification using flash chromatography (hexane–EtOAc). Yield: 17 mg (15%), pale yellow crystals. Mp 145–146 °C (EtOH); ^1^H NMR (600 MHz, DMSO-*d*_6_) δ 9.38 (br s, 1H), 7.84 (d, *J* = 8.5 Hz, 1H), 7.69 (m, 1H), 7.59 (m, 2H), 7.55 (m, 3H), 6.81 (d, *J* = 7.7 Hz, 1H), 6.77 (t, *J* = 5.2 Hz, 1H), 3.50 (s, 3H), 2.27 (m, 2H), 1.16 (t, *J* = 7.5 Hz, 3H); ^13^C{^1^H} NMR (150 MHz, DMSO-*d*_6_) δ 152.7, 147.9, 140.9, 134.8, 134.6, 130.1, 129.1 (2C), 128.9 (2C), 128.8, 119.3, 117.0, 114.5, 35.3, 20.0, 10.3; IR (KBr) ν̃: 3352, 1595, 1472, 1304, 1157, 676 cm^−1^; HREIMS (*m/z*): [M^•^]^+^ calcd for C_17_H_18_N_4_O_2_S, 342.1156; found, 342.1151.

**General methods for the synthesis of compounds 3a**–**j and 10a,b. *Method A, step 2*** [26–27]: A suspension of the corresponding crude hydrazone **7** or **9** (100 mg) and *p-*TsOH monohydrate (1.80 equiv) in toluene (1 mL) was refluxed until the starting material was consumed. Then water (5 mL) was added. The mixture was extracted with EtOAc (3 × 5 mL), washed with water (5 mL) and brine (5 mL), dried over MgSO_4_ and evaporated to give crude products **3** or **10**, which were purified by recrystallization from isopropyl alcohol or by flash chromatography. ***Method B for the preparation of compounds 3*** [22–23]: A suspension of **5** (100 mg), ketone **6** (1.10 equiv), bismuth(III) nitrate pentahydrate (0.22 equiv) and PPA (2.7 equiv) in MeOH (1 mL) was heated at 110 °C in a glass screw cap vial until the starting material and the hydrazone intermediate **7** were consumed. Then the solids were filtered off and washed with EtOAc (3 × 5 mL). The organic phase was washed with water (5 mL) and brine (5 mL), dried over MgSO_4_ and evaporated to give crude products **3**, which were purified by flash chromatography.

**2,4-Dimethyl-2,7,8,9,10,11-hexahydro[1,2,3]thiadiazino[6,5-*a*]carbazole 1,1-dioxide (3e).** Prepared according to Method A, using crude **7e** as the starting material. Yield: 63.0 mg (66%). Prepared according to Method B, using **5a** and **6e** as the starting materials. Yield: 115.0 mg (67%), off-white crystals. Mp 200–201 °C; ^1^H NMR (600 MHz, CDCl_3_) δ 9.00 (br s, 1H), 7.72 (d, *J* = 8.4 Hz, 1H), 7.29 (d, *J* = 8.4 Hz, 1H), 3.54 (s, 3H), 2.82 (m, 2H), 2.73 (m, 2H), 2.56 (s, 3H), 1.95 (m, 2H), 1.89 (m, 2H); ^13^C{^1^H} NMR (150 MHz, CDCl_3_) δ 149.9, 139.6, 131.8, 127.1, 122.1, 121.9, 116.4, 114.6, 111.6, 34.7, 23.3, 22.8, 22.7, 20.7, 20.6; IR (KBr) ν̃: 3397, 1292, 1184, 1126, 1098 cm^−1^; HREIMS (*m/z*): [M^•^]^+^ calcd for C_15_H_17_N_3_O_2_S, 303.1036; found, 303.1038.

**2,7-Dimethyl-4-phenyl-2,9-dihydro[1,2,3]thiadiazino[5,6-*g*]indole 1,1-dioxide (3h).** Prepared according to Method A, using crude **7h** as the starting material. Yield: 47.5 mg (50%). Prepared according to Method B, using **5b** and **6c** as the starting materials. Yield: 60.0 mg (46%), yellow crystals. Mp 157–158 °C (EtOH); ^1^H NMR (600 MHz, DMSO-*d*_6_) δ 11.57 (br s, 1H), 7.94 (d, *J* = 8.5 Hz, 1H), 7.62 (m, 2H), 7.56 (m, 3H), 7.50 (br s, 1H), 7.05 (d, *J* = 8.5 Hz, 1H), 3.55 (s, 3H), 2.33 (s, 3H); ^13^C{^1^H} NMR (150 MHz, DMSO-*d*_6_) δ 152.9, 135.6, 132.5, 129.9, 129.3, 129.2 (2C), 128.8 (2C), 126.4, 123.6, 121.2, 118.1, 116.4, 111.8, 35.0, 9.3; IR (KBr) ν̃: 3377, 1354, 1292, 1137, 1084, 694 cm^−1^; HREIMS (*m*/*z*): [M^•^]^+^ calcd for C_17_H_15_N_3_O_2_S, 325.0885; found, 325.0886.

## Supporting Information

Crystallographic data were deposited at the Cambridge Crystallographic Data Centre under the following numbers: CCDC 2447633 (**3b**), CCDC 2447638 (**3d**), CCDC 2447632 (**3e**), CCDC 2447636 (**3g**), CCDC 2447639 (**3h**), CCDC 2447635 [(*E*)-**7a**], CCDC 2447644 (**7b**), CCDC 2447641 (**7d**), CCDC 2447634 (**7e**), CCDC 2447643 [(*E*)-(**7f**)], CCDC 2447642 [(*Z*)-**7h**], CCDC 2447640 (**7i**) and CCDC 2447637 [(*E*)-**9a**].

File 1 Experimental procedures and characterization of compounds (*E*)-**7a**, **7b**, (*E*)-**7c**, (*Z*)-**7c**, **7d**, (*E*)-**7f**, **7g**, **7i**, **7j**, (*E*)-**9a**, (*E*)-**9b**, **3a**–**d**, **3f**, **3g**, **3i**, **3j**, **10a**, **10b**. Kinetic solubility of compounds **7a**–**j**, **3a**–**j**, (*E*)-**9a**, (*E*)-**9b**, **10a** and **10b**; permeability and membrane retention of compounds (*E*)-**7a**, (*E*)-**7c**, (*Z*)-**7c**, **7d**, **7e**, **3b**, **3c** and **3e**.

File 2 Crystallographic information files, checkcif and structure report files for compounds **3b**, **3d**, **3e**, **3g**, **3h**, (*E*)-**7a**, **7b**, **7d**, **7e**, (*E*)-**7f**, (*Z*)-**7h**, **7i** and (*E*)-**9a**.

## Data Availability

All data that supports the findings of this study is available in the published article and/or the supporting information of this article.

## References

[1] Thomas T L, Radov L A (1987). Anti-inflammatory phthalazinones. U.S. Patent.

[2] Bennani Y, Tumey L N, Glelason E A, Robarge M J (2006). Indole acetic acids exhibiting CRTH2 receptor antagonism and uses thereof. Int. Pat. Appl..

[3] Li J H, Tays K L, Zhang J (1999). Oxo-substituted compounds, process of making, and compositions and methods for inhibiting PARP activity. Int. Patent.

[4] Radov L A, Thomas T L (1989). 1(2H)-Phthalazinones as cytoprotective agents. Eur. Pat. Appl..

[5] Vogelsang D, Scheffer G, Brock N, Lenke D (1974). Basically substituted benzyl phthalazone derivatives, acid salts thereof and process for the production thereof. U.S. Patent.

[6] Porcs-Makkay M, Lukács G, Pandur A, Simig G, Volk B (2014). Tetrahedron.

[7] Porcs‐Makkay M, Gyűjtő I, Lukács G, Komáromi A, Tóth G, Garádi Z, Simig G, Volk B (2019). ChemistrySelect.

[8] Gyűjtő I, Porcs-Makkay M, Lukács G, Pusztai G, Garádi Z, Tóth G, Nyulasi B, Simig G, Volk B (2019). Synth Commun.

[9] Gyűjtő I, Simig G, Porcs-Makkay M, Volk B (2020). Chemistry.

[10] Gyűjtő I, Porcs-Makkay M, Várda E F, Pusztai G, Tóth G, Simig G, Volk B (2020). Synth Commun.

[11] Pusztai G, Porcs-Makkay M, Gál D, Kelemen Z, Simig G, Volk B (2023). Tetrahedron.

[12] Porcs-Makkay M, Lukács G, Kapus G, Gacsályi I, Simig G, Lévay G, Mezei T, Végh M, Kertész S, Barkóczy J (2008). Benzo[1,2,3]-thiadiazine derivatives. Int. Pat. Appl..

[13] Chadha N, Silakari O (2018). Key Heterocycle Cores for Designing Multitargeting Molecules.

[14] Sharma R L, Kour D, Singh J, Kumar S, Gupta P, Gupta S, Kour B, Sachar A (2008). J Heterocycl Chem.

[15] Kaushik N K, Kaushik N, Attri P, Kumar N, Kim C H, Verma A K, Choi E H (2013). Molecules.

[16] Ciccolini C, De Crescentini L, Mantellini F, Mari G, Santeusanio S, Favi G (2020). Molecules.

[17] Singh A, Bhutani C, Khanna P, Talwar S, Singh S K, Khanna L (2025). Eur J Med Chem.

[18] Fischer E, Hess O (1884). Ber Dtsch Chem Ges.

[19] Robinson B (1963). Chem Rev.

[20] Hughes D L (1993). Org Prep Proced Int.

[21] Hughes D L, Zhao D (1993). J Org Chem.

[22] Sudhakara A, Jayadevappa H, Mahadevan K M, Hulikal V (2009). Synth Commun.

[23] Sudhakara A, Jayadevappa H, Kumar H N H, Mahadevan K M (2009). Lett Org Chem.

[24] Bie J, Liu S, Zhou J, Xu B, Shen Z (2014). Bioorg Med Chem.

[25] Miller F M, Schinske W N (1978). J Org Chem.

[26] Murakami Y, Yokoyama Y, Miura T, Hirasawa H, Kamimura Y, Izaki M (1984). Heterocycles.

[27] Lim Y-K, Cho C-G (2004). Tetrahedron Lett.

[28] Zhou M, Li T, Zhang P, Lai Y, Sheng L, Ouyang G (2024). Ann Hematol.

[29] Bussel J B, Cheng G, Saleh M N, Psaila B, Kovaleva L, Meddeb B, Kloczko J, Hassani H, Mayer B, Stone N L (2007). N Engl J Med.

[30] Papp Z, Édes I, Fruhwald S, De Hert S G, Salmenperä M, Leppikangas H, Mebazaa A, Landoni G, Grossini E, Caimmi P (2012). Int J Cardiol.

[31] Alper P B, Marsilje T H, Mutnick D, Lu W, Chatterjee A, Roberts M J, He Y, Karanewsky D S, Chow D, Lao J (2008). Bioorg Med Chem Lett.

[32] Ono M, Wada Y, Wu Y, Nemori R, Jinbo Y, Wang H, Lo K-M, Yamaguchi N, Brunkhorst B, Otomo H (1997). Nat Biotechnol.

[33] Eilender D, LoRusso P, Thomas L, McCormick C, Rodgers A H, Hooper C L, Tornyos K, Krementz E T, Parker S, Morgan L R (2006). Cancer Chemother Pharmacol.

[34] Wager T T, Hou X, Verhoest P R, Villalobos A (2016). ACS Chem Neurosci.

[35] Lipinski C A, Lombardo F, Dominy B W, Feeney P J (2001). Adv Drug Delivery Rev.

[36] (2019). ACD/Labs Percepta.

[37] Ishikawa M, Hashimoto Y (2011). J Med Chem.

[38] Pinal R (2004). Org Biomol Chem.

[39] Dannenfelser R M, Surendran N, Yalkowsky S H (1993). SAR QSAR Environ Res.

[40] Arranz-Gibert P, Guixer B, Malakoutikhah M, Muttenthaler M, Guzmán F, Teixidó M, Giralt E (2015). J Am Chem Soc.

[41] Abramowitz R, Yalkowsky S H (1990). Pharm Res.

[42] Hansen C M (1969). Ind Eng Chem Prod Res Dev.

[43] Chiba M, Ishii Y, Sugiyama Y (2009). AAPS J.

[44] Di L, Obach R S (2015). AAPS J.

[45] Lewis D F V, Dickins M (2003). Drug Metab Rev.

[46] Duddeck H, Dietrich W, Tóth G (1998). Structure Elucidation by Modern NMR.

[47] Pretsch E, Tóth G, Munk M E (2002). Computer-Aided Structure Elucidation: Spectra Interpretation and Structure Generation.

[48] Avdeef A (2012). Absorption and Drug Development: Solubility, Permeability, and Charge State.

