# Supplementary material for: Thiadiazino-indole, thiadiazino-carbazole and benzothiadiazino-carbazole dioxides: synthesis, physicochemical and early ADME characterization of representatives of new tri-, tetra- and pentacyclic ring systems and their intermediates
Source: Beilstein J Org Chem. 2025 Oct 21;21:2220–33. doi: 10.3762/bjoc.21.169 (PMC12557438; doi:10.3762/bjoc.21.169)

BEILSTEIN JOURNAL OF ORGANIC CHEMISTRY

Supporting Information

for

Thiadiazino-indole, thiadiazino-carbazole and benzothiadiazino-carbazole dioxides: synthesis, physicochemical and early ADME characterization of representatives of new tri-, tetra- and pentacyclic ring systems and their intermediates

Gyöngyvér Pusztai, László Poszávácz, Anna Vincze, András Marton, Ahmed Qasim Abdulhussein, Judit Halász, András Dancsó, Gyula Simig, György Tibor Balogh and Balázs Volk

Beilstein J. Org. Chem. **2025,** *21,* 2220–2233. doi:10.3762/bjoc.21.169

Experimental procedures and characterization of compounds (*E*)-7a, 7b, (*E*)-7c, (*Z*)-7c, 7d, (*E*)-7f, 7g, 7i, 7j, (*E*)-9a, (*E*)-9b, 3a–d, 3f, 3g, 3i, 3j, 10a, 10b. Kinetic solubility of compounds 7a–j, 3a–j, (*E*)-9a, (*E*)-9b, 10a and 10b; permeability and membrane retention of compounds (*E*)-7a, (*E*)-7c, (*Z*)-7c, 7d, 7e, 3b, 3c and 3e

License and Terms: This is a supporting information file under the terms of the Creative Commons Attribution License (https://creativecommons.org/licenses/by/4.0). Please note that the reuse, redistribution and reproduction in particular requires that the author(s) and source are credited and that individual graphics may be subject to special legal provisions.
The license is subject to the *Beilstein Journal of Organic Chemistry* terms and conditions: (https://www.beilstein-journals.org/bjoc/terms)

Table of contents

Table S1: Calculated physicochemical properties of the compounds S2

Figure S1: Kinetic solubility of the compounds S3

Figure S2: GI PAMPA permeability of the compounds with a minimal kinetic solubility of 10 μM S3

Figure S3: GI PAMPA membrane retention of the compounds with a minimal kinetic solubility of 10 μM S4

Experimental procedures and characterization of compounds (*E*)-**7a**, **7b**, (*E*)-**7c**, (*Z*)-**7c**, **7d**, (*E*)-**7f**, **7g**, **7i**, **7j**, (*E*)-**9a**, (*E*)-**9b**, **3a–d**, **3f**, **3g**, **3i**, **3j**, **10a**, and **10b** S4

^{1}H NMR, ^{13}C NMR, IR and HRMS spectra of all compounds S17

Table S1. Calculated physicochemical properties of the compounds (ACD Labs/Percepta[a])

Compound	Molecular weight (Da)	clog*P* (-)	cpK_a (basic)	No of rings	TPSA ($Å^2$)	No of hydrogen donors	No of hydrogen acceptors
(*E*)-**7a**	294.37	2.13	3.37	2	82.51	1	6
**7b**	308.4	2.44	3.37	2	82.51	1	6
(*E*)-**7c**	280.35	1.75	2.46	2	82.51	1	6
(*Z*)-**7c**	280.35	1.75	2.46	2	82.51	1	6
**7d**	306.38	1.75	3.37	3	82.51	1	6
**7e**	320.41	2.28	3.11	3	82.51	1	6
(*E*)-**7f**	356.44	3.26	3.29	3	82.51	1	6
**7g**	370.47	3.57	3.29	3	82.51	1	6
crude **7h**	342.42	2.89	2.38	3	82.51	1	6
**7i**	370.47	2.94	3.72	4	82.18	2	6
**7j**	384.5	3.15	3.72	4	82.18	2	6
**3a**	277.34	1.93	-	3	73.91	1	5
**3b**	291.37	2.28	-	3	73.91	1	5
**3c**	263.32	1.55	-	3	73.91	1	5
**3d**	289.35	1.86	-	4	73.91	1	5
**3e**	303.38	2.26	-	4	73.91	1	5
**3f**	339.41	3.26	-	4	73.91	1	5
**3g**	353.44	2.94	-	4	73.91	1	5
**3h**	325.39	2.49	-	4	73.91	1	5
**3i**	351.42	3.62	-	5	73.91	1	5
**3j**	365.45	3.77	-	5	73.91	1	5
(*E*)-**9a**	368.45	2.96	1.49	4	82.51	1	6
(*E*)-**9b**	430.52	4.11	1.41	5	82.51	1	6
**10a**	351.42	3.56	-	5	73.91	1	5
**10b**	413.49	4.91	-	6	73.91	1	5

[a]ACD/Labs Percepta, version: 2019.1.0; https://www.acdlabs.com/products/percepta/ Accessed: Jan 08, 2025

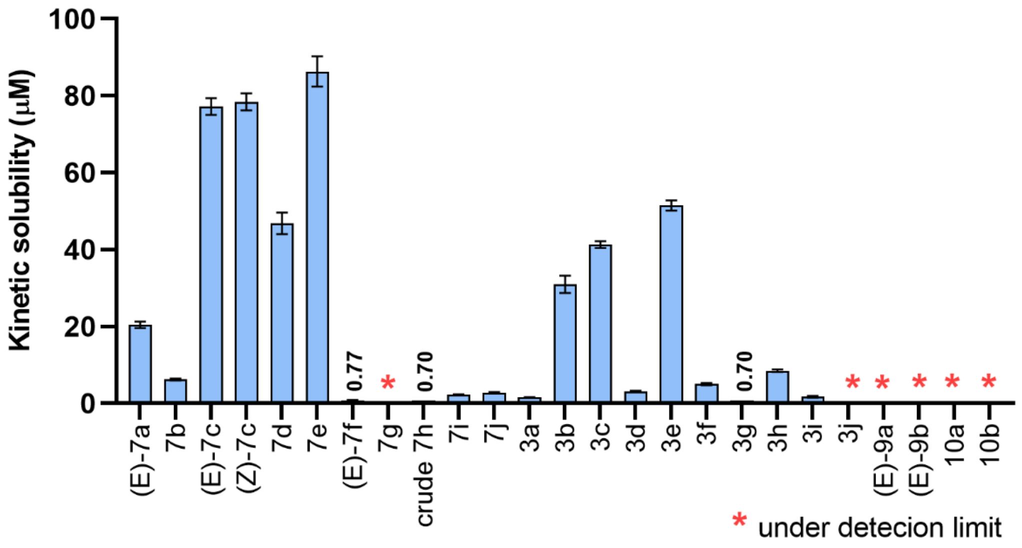

Figure S1. Kinetic solubility of the compounds (PBS pH 7.4, 1–10% DMSO, 2 h, 37 °C; *n* = 3).

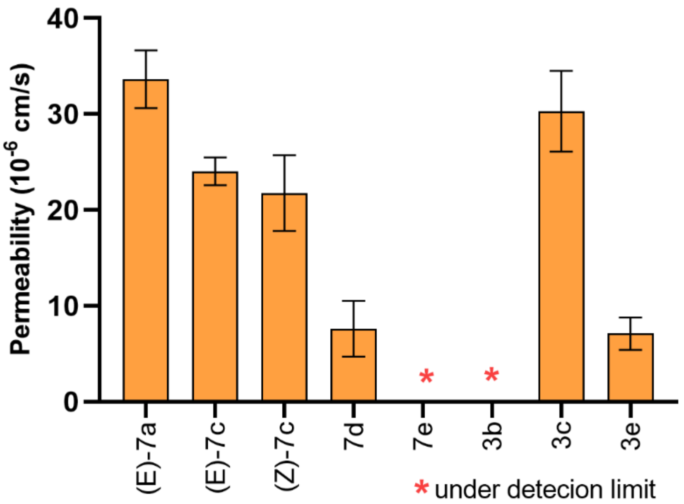

Figure S2. GI PAMPA permeability of the compounds with a minimal kinetic solubility of 10 μM. (membrane PC:chol 4:1; 1% DMSO, donor pH 6.5, acceptor pH 7.4; 4 h, 37 °C; *n* = 3).

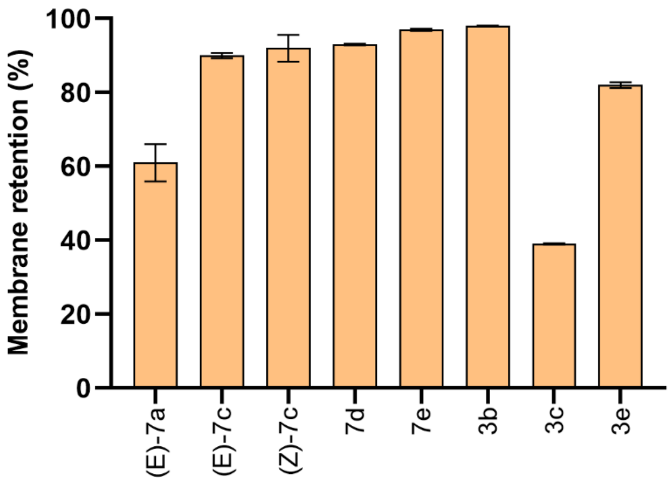

Figure S3. GI PAMPA membrane retention of the compounds with a minimal kinetic solubility of 10 µM. (membrane PC:chol 4:1; 1% DMSO, donor pH 6.5, acceptor pH 7.4; 4 h, 37 °C; n=3)

Experimental procedures and characterization of compounds (*E*)-7a, 7b, (*E*)-7c, (*Z*)-7c, 7d, (*E*)-7f, 7g, 7i, 7j, (*E*)-9a, (*E*)-9b, 3a–d, 3f, 3g, 3i, 3j, 10a, 10b

2,4-Dimethyl-8-[(2*E*)-2-(1-methylpropylidene)hydrazino]-2*H*-1,2,3-benzothiadiazine 1,1-dioxide [(*E*)-7a]. Prepared according to the general procedure, using **5a** and **6a** as the starting materials. Yield: 121.0 mg (99%), crude **7a**, mixture of *E* and *Z* isomers in a 96:4 ratio (according to LC–MS). Pure (*E*)-**7a** was obtained by purification using flash chromatography (hexane–EtOAc). Yield: 115.0 mg (94%), yellow powder. Mp 99–100 °C (EtOH). ^{1}H NMR (600 MHz, DMSO-d_6): 9.04 (br s, 1H), 7.81 (d, J = 8.5 Hz, 1H), 7.72 (m, 1H), 7.20 (d, J = 7.7 Hz, 1H), 3.38 (s, 3H), 2.44 (s, 3H), 2.34 (q, J = 7.4 Hz, 2H), 1.88 (s, 3H), 1.10 (t, J = 7.4 Hz, 3H). ^{13}C{^{1}H} NMR (150 MHz, DMSO-d_6): 153.0, 149.9, 141.2, 134.6, 129.2, 116.7 (2C), 113.3, 34.8, 31.5, 20.4, 15.1, 10.8. IR (KBr): 3363, 1570, 1472, 1301, 1151; HRMS (EI) *m*/*z*: $M^{\bullet+}$ calcd for $C_{13}H_{19}N_4O_2S$ 295.1229; found: 295.1228.

8-[2-(1-Ethylpropylidene)hydrazino]-2,4-dimethyl-2*H*-1,2,3-benzothiadiazine 1,1-dioxide (7b). Prepared according to the general procedure, using **5a** and **6b** as the starting materials. Yield: 106.5 mg (83%), yellow crystals. Mp 94–95 °C (EtOH). ^{1}H NMR (600 MHz, DMSO-d_6): 9.20 (br s, 1H), 7.82 (d, J = 8.4 Hz, 1H), 7.73 (m, 1H), 7.20 (d, J = 7.7 Hz, 1H), 3.38 (s, 3H), 2.44 (s, 3H), 2.35 (q, J = 7.3 Hz, 2H), 2.27 (q, J = 7.7 Hz, 2H), 1.11 (t, J = 7.3 Hz, 3H), 1.10 (t, J = 7.7 Hz, 3H). ^{13}C{^{1}H} NMR (150 MHz, DMSO-d_6): 157.1, 149.8, 141.3, 134.7,

129.2, 116.7, 116.6, 113.2, 34.8, 29.2, 22.3, 20.4, 10.7, 9.3. IR (KBr): 3355, 1570, 1475, 1297, 1150; HRMS (EI) *m*/*z*: $M^{\bullet+}$ calcd for $C_{14}H_{20}N_4O_2S$ 308.1301; found: 308.1306.

2,4-Dimethyl-8-[(2*E*)-2-propylidenehydrazino]-2*H*-1,2,3-benzothiadiazine 1,1-dioxide [(*E*)-7c]. Prepared according to the general procedure, using **5a** and **6c** as the starting materials. Yield: 85 mg (74%), crude **7c**, mixture of *E* and *Z* isomers in a 2:1 ratio (according to LC–MS). Pure (*E*)-**7c** was obtained by purification using flash chromatography (hexane–EtOAc). Yield: 55 mg (48%), colorless crystals. Mp 90–91 °C (EtOH). ^{1}H NMR (600 MHz, DMSO-d_6): 9.40 (br s, 1H), 7.77 (d, *J* = 8.6 Hz, 1H), 7.69 (m, 1H), 7.57 (t, *J* = 5.0 Hz, 1H), 7.18 (d, *J* = 7.6 Hz, 1H), 3.36 (s, 3H), 2.43 (s, 3H), 2.29 (m, 2H), 1.08 (t, *J* = 7.4 Hz, 3H). $^{13}C\{^{1}H\}$ NMR (150 MHz, DMSO-d_6): 149.9, 148.8, 141.0, 134.4, 129.3, 117.0, 116.6, 113.2, 34.8, 25.5, 20.4, 10.9. IR (KBr): 3324, 1599, 1472, 1333, 1311, 1156; HRMS (EI) *m*/*z*: $M^{\bullet+}$ calcd for $C_{12}H_{16}N_4O_2S$ 280.0998; found: 280.0993.

2,4-Dimethyl-8-[(2*Z*)-2-propylidenehydrazino]-2*H*-1,2,3-benzothiadiazine 1,1-dioxide [(*Z*)-7c]. Prepared according to the general procedure, using **5a** and **6c** as the starting materials. Yield: 85 mg (74%), crude **7c**, mixture of *E* and *Z* isomers in a 2:1 ratio (according to LC–MS). Pure (*Z*)-**7c** was obtained by purification using flash chromatography (hexane–EtOAc). Yield: 26 mg (23%), pale yellow crystals. Mp 110–111 °C (EtOH). ^{1}H NMR (600 MHz, DMSO-d_6): 9.32 (br s, 1H), 7.80 (d, *J* = 8.4 Hz, 1H), 7.75 (m, 1H), 7.27 (d, *J* = 7.6 Hz, 1H), 6.74 (t, *J* = 5.1 Hz, 1H), 3.38 (s, 3H), 2.45 (s, 3H), 2.23 (m, 2H), 1.14 (t, *J* = 7.5 Hz, 3H). $^{13}C\{^{1}H\}$ NMR (150 MHz, DMSO-d_6): 149.9, 147.6, 140.8, 134.7, 129.2, 117.5, 116.8, 113.8, 34.8, 20.4, 19.9, 10.3. IR (KBr): 3337, 1601, 1477, 1292, 1157; HRMS (EI) *m*/*z*: $M^{\bullet+}$ calcd for $C_{12}H_{16}N_4O_2S$ 280.0998; found: 280.0993.

8-(2-Cyclopentylidenehydrazino)-2,4-dimethyl-2*H*-1,2,3-benzothiadiazine 1,1-dioxide (7d). Prepared according to the general procedure, using **5a** and **6d** as the starting materials. Yield: 117.5 mg (92%), yellow crystals. Mp 121–122 °C (EtOH). ^{1}H NMR (600 MHz, DMSO-d_6): 8.84 (br s, 1H), 7.75 (d, *J* = 8.5 Hz, 1H), 7.70 (m, 1H), 7.18 (d, *J* = 7.5 Hz, 1H), 3.37 (s, 3H), 2.44 (s, 3H), 2.42 (t, *J* = 7.3 Hz, 2H), 2.28 (t, *J* = 7.3 Hz, 2H), 1.84 (m, 2H), 1.73 (m, 2H). $^{13}C\{^{1}H\}$ NMR (150 MHz, DMSO-d_6): 161.8, 149.8, 141.1, 134.6, 129.2, 116.6, 116.5, 113.1, 34.8, 32.8, 27.2, 24.7, 24.6, 20.4. IR (KBr): 3337, 1598, 1473, 1297, 1154; HRMS (EI) *m*/*z*: $M^{\bullet+}$ calcd for $C_{14}H_{18}N_4O_2S$ 306.1150; found: 306.1145.

8-[(2*E*)-2-(3,4-Dihydronaphthalen-1(2*H*)-ylidene)hydrazino]-2,4-dimethyl-2*H*-1,2,3-benzothiadiazine 1,1-dioxide [(*E*)-9a]. Prepared according to the general procedure, using **5a** and **8** as the starting materials. Yield: 145.5 mg (95%), yellow crystals. Mp 180–181 °C (EtOH).

^{1}H NMR (600 MHz, DMSO-d_6): 9.46 (br s, 1H), 8.12 (m, 1H), 8.05 (d, J = 8.5 Hz, 1H), 7.80 (m, 1H), 7.28 (m, 3H), 7.21 (m, 1H), 3.41 (s, 3H), 2.76 (m, 2H), 2.63 (t, J = 6.4 Hz, 2H), 2.47 (s, 3H), 1.92 (m, 2H). ^{13}C{^{1}H} NMR (150 MHz, DMSO-d_6): 150.0, 146.6, 140.7, 139.5, 134.8, 132.5, 129.3, 128.8, 128.7, 126.6, 124.3, 117.5, 117.1, 114.1, 34.8, 28.9, 24.8, 21.3, 20.4. IR (KBr): 3346, 1572, 1473, 1311, 1293, 1104; HRMS (EI) *m/z:* $M^{\bullet+}$ calcd for $C_{19}H_{20}N_4O_2S$ 368.1307; found: 368.1296.

2-Methyl-8-[(2*E*)-2-(1-methylpropylidene)hydrazino]-4-phenyl-2*H*-1,2,3-benzothiadiazine 1,1-dioxide [(*E*)-7f]. Prepared according to the general procedure, using **5b** and **6a** as the starting materials. Yield: 110.0 mg (93%), crude **7f**, mixture of *E* and *Z* isomers in a 9:1 ratio (according to LC–MS). Pure (*E*)-**7f** was obtained by purification using flash chromatography (hexane–EtOAc). Yield: 100.3 mg (84%), yellow crystals. Mp 123–124 °C (EtOH). ^{1}H NMR (600 MHz, DMSO-d_6): 9.11 (br s, 1H), 7.84 (d, J = 8.5 Hz, 1H), 7.66 (m, 1H), 7.58 (m, 2H), 7.54 (m, 3H), 6.74 (d, J = 7.6 Hz, 1H), 3.49 (s, 3H), 2.35 (q, J = 7.4 Hz, 2H), 1.92 (s, 3H), 1.11 (t, J = 7.4 Hz, 3H). ^{13}C{^{1}H} NMR (150 MHz, DMSO-d_6): 153.4, 152.7, 141.4, 134.9, 134.5, 130.0, 129.1 (2C), 128.9 (2C), 128.8, 118.5, 117.0, 114.0, 35.3, 31.5, 15.1, 10.8. IR (KBr): 3359, 1592, 1469, 1315, 1299, 1151; HRMS (EI) *m/z:* $M^{\bullet+}$ calcd for $C_{18}H_{20}N_4O_2S$ 356.1310; found: 356.1304.

8-[2-(1-Ethylpropylidene)hydrazino]-2-methyl-4-phenyl-2*H*-1,2,3-benzothiadiazine 1,1-dioxide (7g). Prepared according to the general procedure, using **5b** and **6b** as the starting materials. Yield: 108.0 mg (88%), yellow crystals. Mp 160–161 °C (EtOH). ^{1}H NMR (600 MHz, DMSO-d_6): 9.26 (br s, 1H), 7.85 (d, J = 8.5 Hz, 1H), 7.66 (m, 1H), 7.58 (m, 2H), 7.55 (m, 1H), 7.54 (m, 2H), 6.74 (d, J = 8.5 Hz, 1H), 3.50 (s, 3H), 2.37 (q, J = 7.3 Hz, 2H), 2.31 (q, J = 7.7 Hz, 2H), 1.13 (t, J = 7.7 Hz, 3H), 1.12 (t, J = 7.3 Hz, 3H). ^{13}C{^{1}H} NMR (150 MHz, DMSO-d_6): 157.4, 152.7, 141.4, 134.9, 134.5, 130.0, 129.1 (2C), 128.9 (2C), 128.7, 118.5, 116.9, 113.9, 35.3, 29.2, 22.4, 10.1, 9.3. IR (KBr): 3367, 1594, 1471, 1317, 1299, 1152; HRMS (EI) *m/z:* $M^{\bullet+}$ calcd for $C_{19}H_{22}N_4O_2S$ 370.1463; found: 370.1465.

8-(2-Cyclopentylidenehydrazino)-2-methyl-4-phenyl-2*H*-1,2,3-benzothiadiazine 1,1-dioxide (7i). Prepared according to the general procedure, using **5b** and **6d** as the starting materials. Yield: 114.6 mg (94%), yellow crystals. Mp 151–152 °C (EtOH). ^{1}H NMR (600 MHz, DMSO-d_6): 8.91 (br s, 1H), 7.78 (d, J = 8.5 Hz, 1H), 7.64 (m, 1H), 7.58 (m, 2H), 7.55 (m, 1H), 7.54 (m, 2H), 6.73 (d, J = 7.7 Hz, 1H), 3.49 (s, 3H), 2.44 (t, J = 7.1 Hz, 2H), 2.32 (t, J = 7.1 Hz, 2H), 1.86 (m, 2H), 1.74 (m, 2H). ^{13}C{^{1}H} NMR (150 MHz, DMSO-d_6): 162.1, 152.7, 141.3, 134.9, 134.5, 130.0, 129.1 (2C), 128.9 (2C), 128.8, 118.5, 116.8, 113.8, 35.2, 32.9,

27.3, 24.7, 24.6. IR (KBr): 3343, 1591, 1469, 1348, 1153, 1106, 692; HRMS (ESI) *m/z:* $[M+H]^+$ calcd for $C_{19}H_{21}N_4O_2S$ 369.1380; found: 369.1381.

8-(2-Cyclohexylidenehydrazino)-2-methyl-4-phenyl-2*H*-1,2,3-benzothiadiazine 1,1-dioxide (7j). Prepared according to the general procedure, using **5b** and **6e** as the starting materials. Yield: 116.4 mg (92%), yellow crystals. Mp 175–176 °C (EtOH). ^{1}H NMR (600 MHz, DMSO-d_6): 9.26 (br s, 1H), 7.83 (d, J = 8.6 Hz, 1H), 7.64 (m, 1H), 7.58 (m, 2H), 7.55 (m, 1H), 7.54 (m, 2H), 6.73 (d, J = 7.7 Hz, 1H), 3.49 (s, 3H), 2.35 (m, 4H), 1.67 (m, 4H), 1.62 (m, 2H). ^{13}C{^{1}H} NMR (150 MHz, DMSO-d_6): 155.2, 152.7, 141.5, 134.9, 134.5, 130.0, 129.1 (2C), 128.9 (2C), 128.8, 118.4, 116.8, 113.9, 35.3, 34.9, 26.7, 26.1, 25.4, 25.2. IR (KBr): 3367, 1593, 1472, 1299, 1152, 733; HRMS (EI) *m/z:* $M^{\bullet+}$ calcd for $C_{20}H_{22}N_4O_2S$ 382.1463; found: 382.1467.

8-[(2*E*)-2-(3,4-Dihydronaphthalen-1(2*H*)-ylidene)hydrazino]-2-methyl-4-phenyl-2*H*-1,2,3-benzothiadiazine 1,1-dioxide [(*E*)-9b]. Prepared according to the general procedure, using **5b** and **8** as the starting materials. Yield: 145.0 mg (95%), orange crystals. Mp 235–236 °C (EtOH). ^{1}H NMR (600 MHz, DMSO-d_6): 9.53 (br s, 1H), 8.15 (m, 1H), 8.09 (d, J = 8.5 Hz, 1H), 7.74 (m, 1H), 7.61 (m, 2H), 7.57 (m, 1H), 7.56 (m, 2H), 7.29 (m, 1H), 7.28 (m, 1H), 7.22 (m, 1H), 6.83 (m, 1H), 3.52 (s, 3H), 2.78 (m, 2H), 2.68 (t, J = 6.5 Hz, 2H), 1.94 (m, 2H). ^{13}C{^{1}H} NMR (150 MHz, DMSO-d_6): 152.8, 146.9, 140.9, 139.6, 134.8, 134.7, 132.5, 130.1, 129.1 (2C), 128.9 (3C), 128.8, 128.7, 126.6, 124.4, 119.3, 117.5, 114.6, 35.3, 28.9, 24.9, 21.3. ^{1}H NMR (600 MHz, $CDCl_3$): 9.63 (br s, 1H), 8.18 (m, 1H), 8.07 (d, J = 8.4 Hz, 1H), 7.62 (m, 2H), 7.52 (m, 1H), 7.48 (m, 3H), 7.25 (m, 2H*, overlapping with the solvent signal), 7.16 (m, 1H), 6.81 (d, J = 7.8 Hz, 1H), 3.61 (s, 3H), 2.81 (m, 2H), 2.72 (t, J = 6.6 Hz, 2H), 2.03 (m, 2H). ^{13}C{^{1}H} NMR (150 MHz, $CDCl_3$): 153.1, 146.0, 141.3, 139.2, 135.3, 133.4, 132.9, 129.6, 129.4, 129.1 (2C), 128.5 (3C), 128.4, 126.5, 124.4, 119.0, 117.2, 114.9, 35.4, 29.6, 25.1, 21.6. IR (KBr): 3329, 1589, 1465, 1315, 1153, 1105, 721; HRMS (EI) *m/z:* $M^{\bullet+}$ calcd for $C_{24}H_{22}N_4O_2S$ 430.1463; found: 430.1467.

2,4,7,8-Tetramethyl-2,9-dihydro[1,2,3]thiadiazino[5,6-*g*]indole 1,1-dioxide (3a). Prepared according to Method A, using crude **7a** as the starting material. Yield: 70 mg (65%). Prepared according to Method B, using **5a** and **6a** as the starting materials. Yield: 102.0 mg (47%), yellow crystals. Mp 188–189 °C. ^{1}H NMR (600 MHz, DMSO-d_6): 11.35 (br s, 1H), 7.87 (d, J = 8.4 Hz, 1H), 7.42 (d, J = 8.4 Hz, 1H), 3.40 (s, 3H), 2.51 (s, 3H), 2.44 (s, 3H), 2.23 (s, 3H). ^{13}C{^{1}H} NMR (150 MHz, DMSO-d_6): 150.1, 138.9, 133.3, 125.4, 122.4, 120.8, 116.6, 114.9, 107.8,

34.5, 20.7, 11.7, 8.2. IR (KBr): 3373, 1304, 1185, 1153, 1123; HRMS (EI) *m/z:* $M^{•+}$ calcd for $C_{13}H_{15}N_3O_2S$ 277.0879; found: 277.0883.

8-Ethyl-2,4,7-trimethyl-2,9-dihydro[1,2,3]thiadiazino[5,6-*g*]indole 1,1-dioxide (3b). Prepared according to Method A, using crude **7b** as the starting material. Yield: 88.0 mg (92%). Prepared according to Method B, using **5a** and **6b** as the starting materials. Yield: 61.0 mg (50%), off-white crystals. Mp 190–191 °C. ^{1}H NMR (600 MHz, DMSO-*d*$_6$): 11.32 (br s, 1H), 7.89 (d, *J* = 8.4 Hz, 1H), 7.43 (d, *J* = 8.4 Hz, 1H), 3.41 (s, 3H), 2.85 (q, *J* = 7.5 Hz, 2H), 2.51 (s, 3H), 2.25 (s, 3H), 1.20 (t, *J* = 7.5 Hz, 3H). ^{13}C{^{1}H} NMR (150 MHz, DMSO-*d*$_6$): 150.1, 144.7, 133.4, 125.4, 122.6, 121.0, 116.6, 115.1, 107.0, 34.6, 20.7, 19.1, 14.7, 8.1. IR (KBr): 3388, 3363, 1303, 1186, 1123; HRMS (EI) *m/z:* $M^{•+}$ calcd for $C_{14}H_{18}N_3O_2S$ 292.1120; found: 292.1110.

2,4,7-Trimethyl-2,9-dihydro[1,2,3]thiadiazino[5,6-*g*]indole 1,1-dioxide (3c). Prepared according to Method A, using crude **7c** as the starting material. Yield: 42.5 mg (46%). Prepared according to Method B, using **5a** and **6c** as the starting materials. Yield: 33.0 mg (30%), yellow crystals. Mp 192–193 °C (EtOH). ^{1}H NMR (600 MHz, DMSO-*d*$_6$): 11.42 (br s, 1H), 8.01 (d, *J* = 8.4 Hz, 1H), 7.48 (d, *J* = 8.4 Hz, 1H), 7.44 (br s, 1H), 3.42 (s, 3H), 2.53 (s, 3H), 2.34 (s, 3H). ^{13}C{^{1}H} NMR (150 MHz, DMSO-*d*$_6$): 149.9, 132.4, 128.9, 126.3, 123.8, 122.0, 116.5, 115.5, 111.6, 34.6, 20.7, 9.3. IR (KBr): 3377, 1305, 1187, 1140, 1081; HRMS (ESI) *m/z:* $[M+H]^+$ calcd for $C_{12}H_{13}N_3O_2S$: 263.0723; found: 263.0727.

2,4-Dimethyl-7,8,9,10-tetrahydro-2*H*-cyclopenta[*b*][1,2,3]thiadiazino[5,6-*g*]indole 1,1-dioxide (3d). Prepared according to Method A, using crude **7d** as the starting material. Yield: 30.2 mg (33%). Prepared according to Method B, using **5a** and **6d** as the starting materials. Yield: 30.0 mg (25%), yellow crystals. Mp 198–199 °C (decomp). ^{1}H NMR (600 MHz, DMSO-*d*$_6$): 11.51 (br s, 1H), 7.80 (d, *J* = 8.4 Hz, 1H), 7.42 (d, *J* = 8.4 Hz, 1H), 3.41 (s, 3H), 2.91 (t, *J* = 7.2 Hz, 2H), 2.81 (t, *J* = 7.0 Hz, 2H), 2.50 (s, 3H), 2.50 (m, 2H). ^{13}C{^{1}H} NMR (150 MHz, DMSO-*d*$_6$): 151.9, 150.1, 130.5, 128.2, 122.8, 120.4, 119.6, 117.1, 115.8, 34.6, 28.4, 26.2, 23.9, 20.6. IR (KBr): 3373, 1310, 1136, 1102; HRMS (EI) *m/z:* $M^{•+}$ calcd for $C_{14}H_{15}N_3O_2S$ 289.0885; found: 289.0884.

2,7,8-Trimethyl-4-phenyl-2,9-dihydro[1,2,3]thiadiazino[5,6-*g*]indole 1,1-dioxide (3f). Prepared according to Method A, using crude **7f** as the starting material. Yield: 6.07 mg (70%). Prepared according to Method B, using **5b** and **6a** as the starting materials. Yield: 75.0 mg (67%), yellow crystals. Mp 122–123 °C (EtOH). ^{1}H NMR (600 MHz, DMSO-*d*$_6$): 11.50 (br s, 1H), 7.80 (d, *J* = 8.5 Hz, 1H), 7.61 (m, 2H), 7.55 (m, 3H), 7.00 (d, *J* = 8.5 Hz, 1H), 3.52 (s,

3H), 2.47 (s, 3H), 2.21 (s, 3H). $^{13}C\{^{1}H\}$ NMR (150 MHz, DMSO-d_6): 153.1, 139.4, 135.7, 133.4, 129.8, 129.3 (2C), 128.8 (2C), 125.5, 122.2, 120.0, 118.3, 115.7, 108.0, 35.0, 11.7, 8.2. IR (KBr): 3389, 1357, 1312, 1123, 696, 592; HRMS (EI) *m/z:* $M^{\bullet+}$ calcd for $C_{18}H_{17}N_3O_2S$ 339.1041; found: 339.1034.

8-Ethyl-2,7-dimethyl-4-phenyl-2,9-dihydro[1,2,3]thiadiazino[5,6-*g*]indole 1,1-dioxide (3g). Prepared according to Method A, using crude **7g** as the starting material. Yield: 71.0 mg (75%). Prepared according to Method B, using **5b** and **6b** as the starting materials. Yield: 70.0 mg (60%), yellow crystals. Mp 133–134 °C (EtOH). ^{1}H NMR (600 MHz, DMSO-d_6): 11.47 (br s, 1H), 7.82 (d, J = 8.5 Hz, 1H), 7.61 (m, 2H), 7.55 (m, 3H), 7.01 (d, J = 8.5 Hz, 1H), 3.53 (s, 3H), 2.88 (q, J = 7.6 Hz, 2H), 2.24 (s, 3H), 1.23 (t, J =7.6 Hz, 3H). $^{13}C\{^{1}H\}$ NMR (150 MHz, DMSO-d_6): 153.2, 145.1, 135.7, 133.5, 129.8, 129.2 (2C), 128.8 (2C), 125.5, 122.3, 120.2, 118.3, 116.0, 107.2, 35.0, 19.1, 14.7, 8.0. IR (KBr): 3367, 1360, 1309, 1124; HRMS (EI) *m/z:* $M^{\bullet+}$ calcd for $C_{19}H_{19}N_3O_2S$ 353.1198; found: 353.1196.

2-Methyl-4-phenyl-7,8,9,10-tetrahydro-2*H*-cyclopenta[*b*][1,2,3]thiadiazino[5,6-*g*]indole 1,1-dioxide (3i). Prepared according to Method A, using crude **7i** as the starting material. Yield: 43.0 mg (45%). Prepared according to Method B, using **5b** and **6d** as the starting materials. Yield: 41.0 mg (35%), yellow crystals. Mp 150–151 °C (EtOH). ^{1}H NMR (600 MHz, DMSO-d_6): 11.65 (br s, 1H), 7.74 (d, J = 8.4 Hz, 1H), 7.60 (m, 2H), 7.55 (m, 3H), 7.00 (d, J = 8.4 Hz, 1H), 3.52 (s, 3H), 3.94 (m, 2H), 2.80 (m, 2H), 2.51 (m, 2H). $^{13}C\{^{1}H\}$ NMR (150 MHz, DMSO-d_6): 153.1, 152.4, 135.7, 130.6, 129.8, 129.3 (2C), 128.8 (2C), 128.2, 122.6, 119.8, 119.6, 118.8, 116.7, 35.0, 28.4, 26.2, 23.9. IR (KBr): 3438, 1347, 1316, 1135, 1105, 694; HRMS (EI) *m/z:* $M^{\bullet+}$ calcd for $C_{19}H_{17}N_3O_2S$ 351.1041; found: 351.1042.

2-Methyl-4-phenyl-2,7,8,9,10,11-hexahydro[1,2,3]thiadiazino[6,5-*a*]carbazole 1,1-dioxide (3j). Prepared according to Method A, using crude **7j** as the starting material. Yield: 63.5 mg (66%). Prepared according to Method B, using **5b** and **6e** as the starting materials. Yield: 81.0 mg (67%), yellow crystals. Mp 190 °C (decomp). ^{1}H NMR (600 MHz, DMSO-d_6): 11.52 (br s, 1H), 7.78 (d, J = 8.4 Hz, 1H), 7.60 (m, 2H), 7.55 (m, 1H), 7.54 (m, 2H), 7.00 (d, J = 8.4 Hz, 1H), 3.52 (s, 3H), 2.85 (m, 2H), 2.68 (m, 2H), 1.86 (m, 2H), 1.82 (m, 2H). $^{13}C\{^{1}H\}$ NMR (150 MHz, DMSO-d_6): 153.1, 142.2, 135.7, 131.9, 129.8, 129.3 (2C), 128.8 (2C), 126.0, 122.0, 120.1, 118.3, 116.0, 110.7, 35.0, 23.4, 22.7, 22.6, 20.4. IR (KBr): 3385, 1305, 1163, 1123, 697; HRMS (EI) *m/z:* $M^{\bullet+}$ calcd for $C_{20}H_{19}N_3O_2S$ 365.1198; found: 365.1195.

2,4-Dimethyl-2,13-dihydrobenzo[*a*][1,2,3]thiadiazino[5,6-*i*]carbazole 1,1-dioxide (10a). Prepared according to Method A, using crude (*E*)-**9a** as the starting material. Yield: 76 mg

(80%), yellow crystals. Mp 223–224 °C (EtOH). ^{1}H NMR (600 MHz, DMSO-d_6): 12.31 (br s, 1H), 9.12 (d, J = 8.0 Hz, 1H), 8.74 (d, J = 8.0 Hz, 1H), 8.38 (d, J = 8.0 Hz, 1H), 8.11 (d, J = 7.6 Hz, 1H), 7.82 (d, J = 8.0 Hz, 1H), 7.75 (d, J = 8.0 Hz, 1H), 7.70 (m, 1H), 7.66 (m, 1H), 3.50 (s, 3H), 2.62 (s, 3H). ^{13}C{^{1}H} NMR (150 MHz, DMSO-d_6): 150.2, 138.1, 133.2, 129.7, 128.8, 128.1, 126.8, 126.2, 124.8, 124.6, 123.9, 121.9, 121.5, 119.3, 117.9, 117.1, 115.9, 34.8, 20.8. IR (KBr): 3366, 1313, 1137, 801; HRMS (EI) *m/z:* $M^{\bullet+}$ calcd for $C_{19}H_{15}N_3O_2S$ 349.0889; found: 349.0883.

2-Methyl-4-phenyl-2,13-dihydrobenzo[*a*][1,2,3]thiadiazino[5,6-*i*]carbazole 1,1-dioxide (10b). Prepared according to Method A, using crude (*E*)-**9b** as the starting material. Yield: 80 mg (84%), orange crystals. Mp 259–260 °C (EtOH). ^{1}H NMR (600 MHz, DMSO-d_6): 12.48 (br s, 1H), 9.13 (d, J = 8.0 Hz, 1H), 8.66 (d, J = 8.3 Hz, 1H), 8.34 (d, J = 8.5 Hz, 1H), 8.11 (d, J = 8.0 Hz, 1H), 7.82 (d, J = 8.5 Hz, 1H), 7.72 (m, 1H), 7.70 (m, 2H), 7.67 (m, 1H), 7.60 (m, 1H), 7.59 (m, 2H), 7.30 (d, J = 8.3 Hz, 1H), 3.62 (s, 3H). ^{13}C{^{1}H} NMR (150 MHz, DMSO-d_6): 153.0, 138.4, 135.5, 133.3, 130.0, 129.8, 129.3 (2C), 128.9 (2C), 128.8, 128.2, 126.9, 126.3, 124.6, 123.9, 123.8, 122.0, 121.5, 119.5, 119.2, 117.0, 116.9, 35.2. IR (KBr): 3453, 2923, 1315, 1299, 1135, 797, 727, 693; HRMS (EI) *m/z:* $M^{\bullet+}$ calcd for $C_{24}H_{17}N_3O_2S$ 411.1047; found: 411.1037.

^{1}H NMR spectrum of **5a**

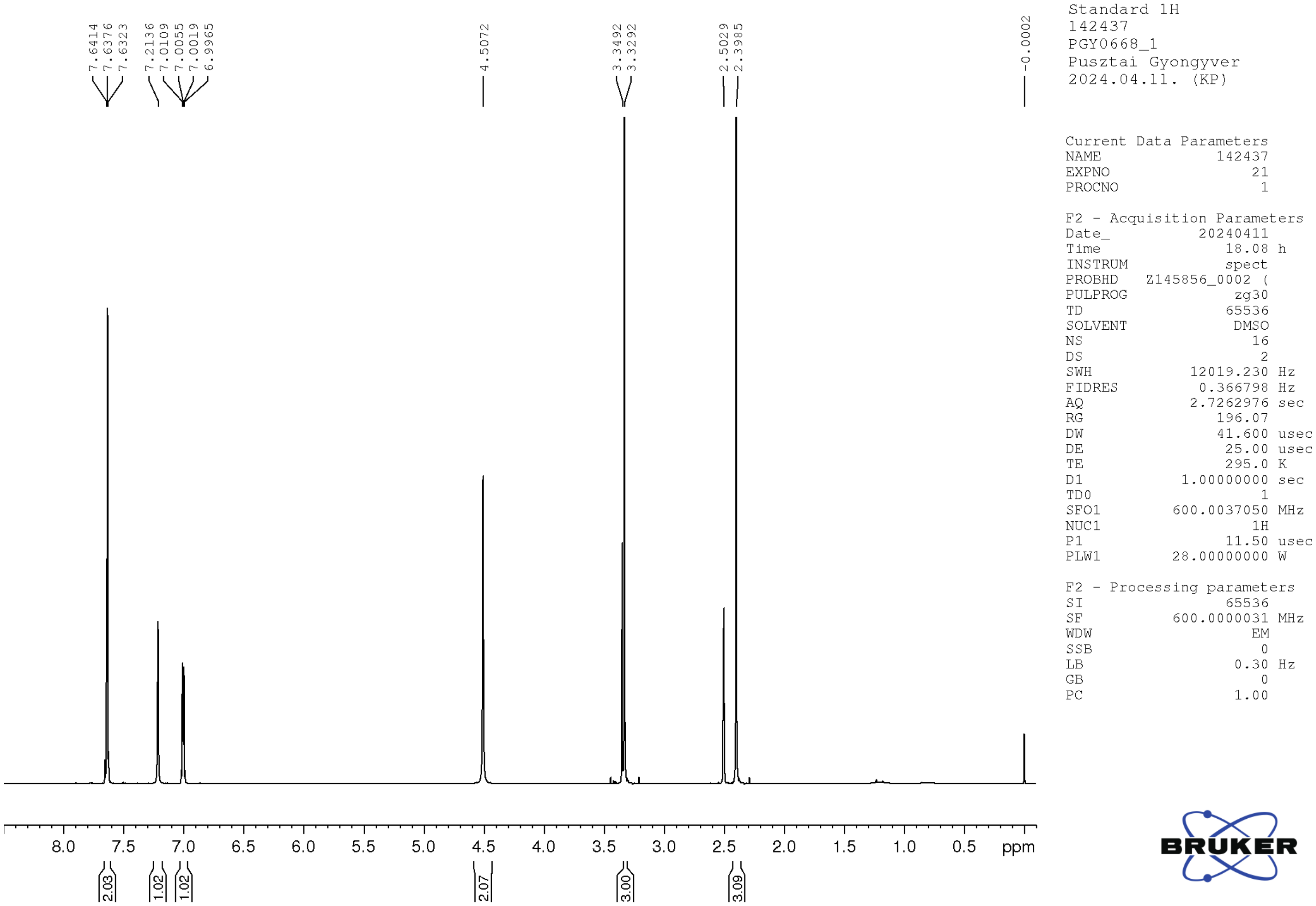

^{13}C NMR spectrum of **5a**

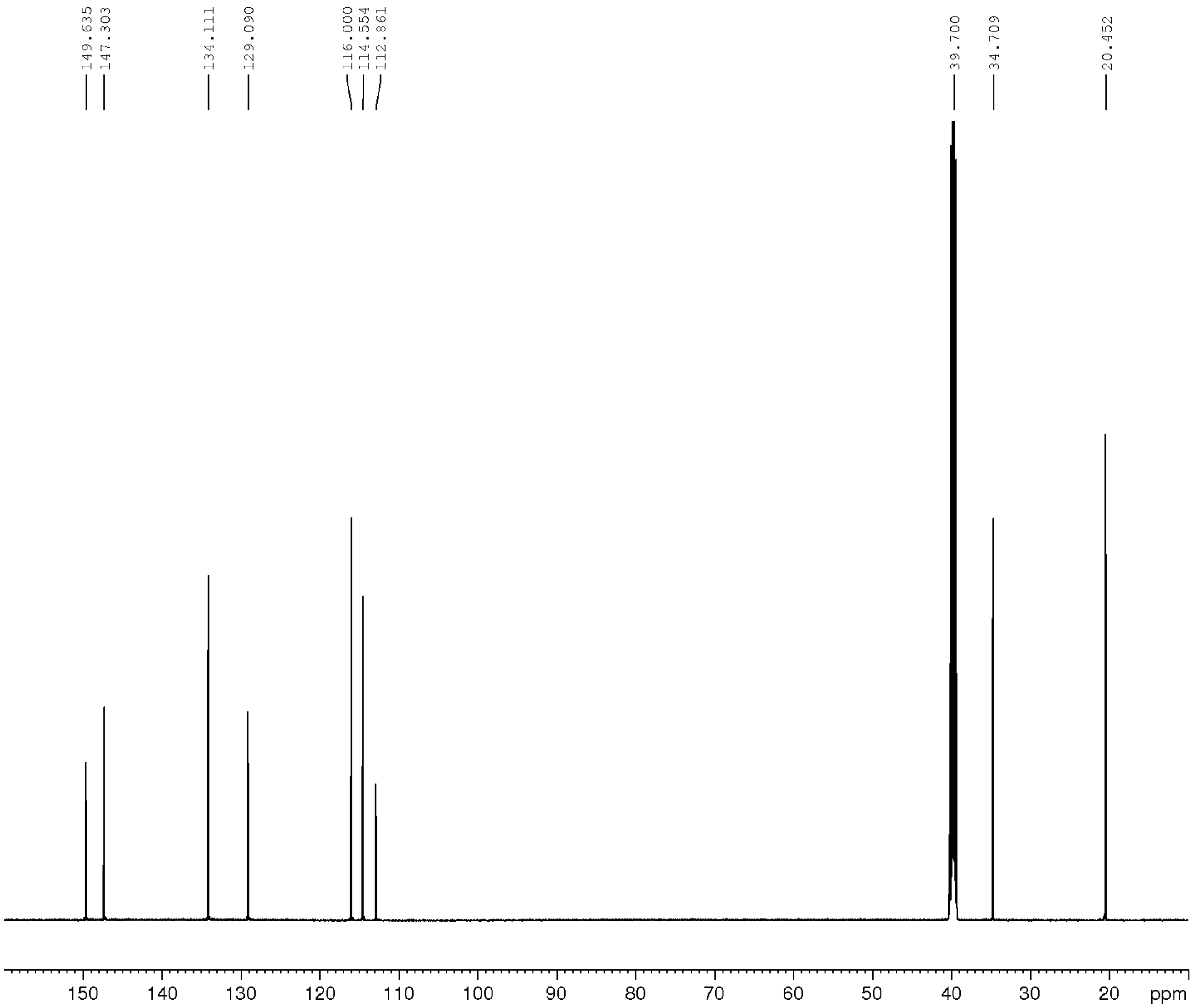

Standard 13C
142437
PGY0668_1
Pusztai Gyongyver
2024.04.11. (KP)

```
Current Data Parameters
NAME              142437
EXPNO                 22
PROCNO                 1

F2 - Acquisition Parameters
Date_           20240411
Time               19.16 h
INSTRUM            spect
PROBHD   Z145856_0002 (
PULPROG          zgpg30
TD                 65536
SOLVENT             DMSO
NS                  2048
DS                     4
SWH            36231.883 Hz
FIDRES          1.105709 Hz
AQ             0.9043968 sec
RG                196.07
DW                13.800 usec
DE                 18.00 usec
TE                 295.0 K
D1            1.00000000 sec
D11           0.03000000 sec
TD0                    1
SFO1         150.8852070 MHz
NUC1                 13C
P1                  9.90 usec
PLW1         71.00000000 W
SFO2         600.0024000 MHz
NUC2                  1H
CPDPRG[2         waltz16
PCPD2              80.00 usec
PLW2         32.90000153 W
PLW12         0.70370001 W
PLW13         0.35339001 W

F2 - Processing parameters
SI                 32768
SF           150.8701591 MHz
WDW                   EM
SSB                    0
LB                  1.00 Hz
GB                     0
PC                  1.40
```

IR spectrum of **5a**

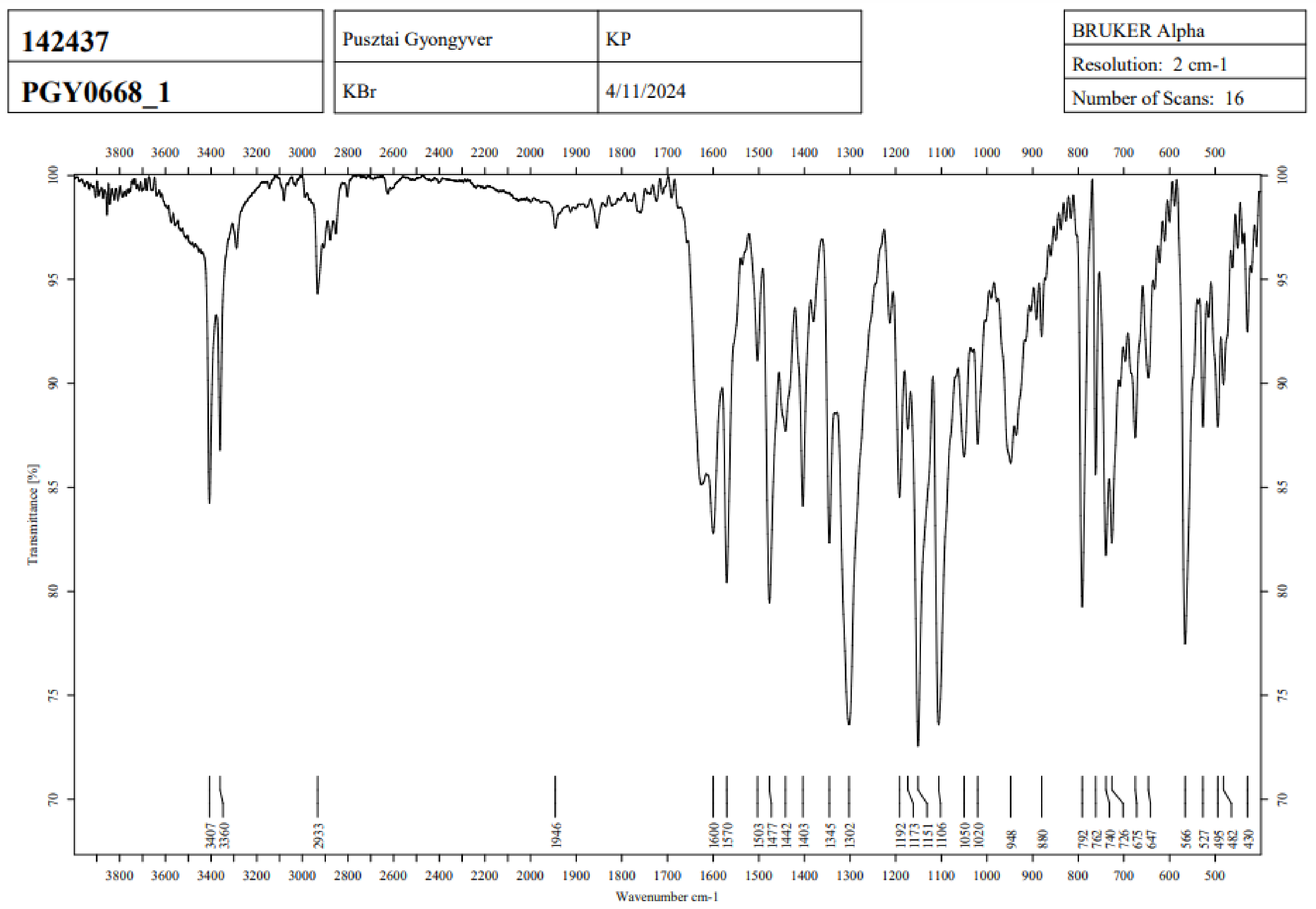

HRMS spectrum of **5a**

Spectrum Plot Report

Name	PGY0668_1, Pusztai Gyongyver	Rack Pos.		Instrument	7250A with DIP	Operator	MM
Inj. Vol. (ul)	0.5	Plate Pos.		IRM Status	Success		
Data File	142437.D	Method (Acq)	DIP_70eV.M	Comment		Acq. Time (Local)	4/16/2024 2:29:07 PM (UTC+02:00)

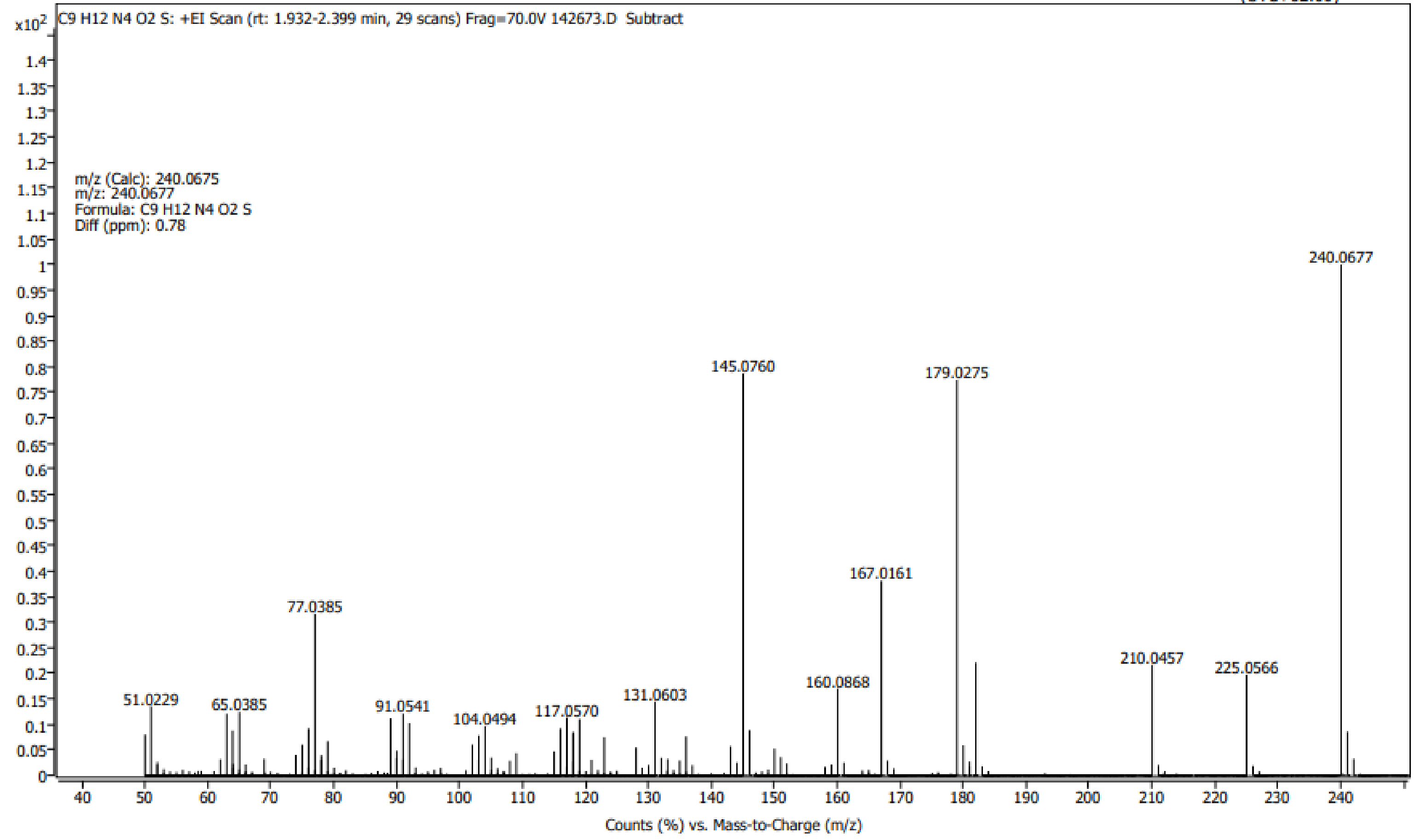

^{1}H NMR spectrum of **5b**

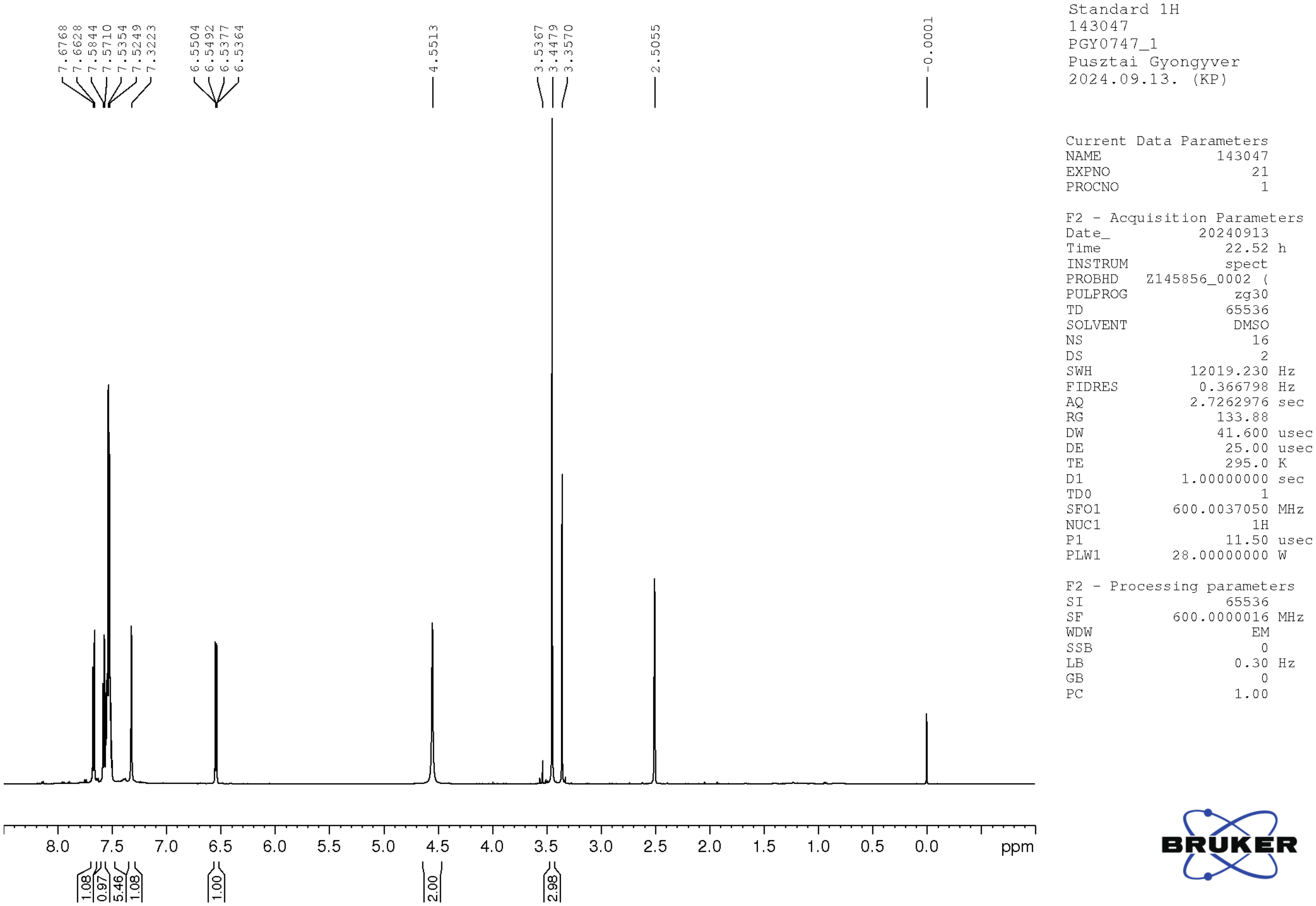

^{13}C NMR spectrum of **5b**

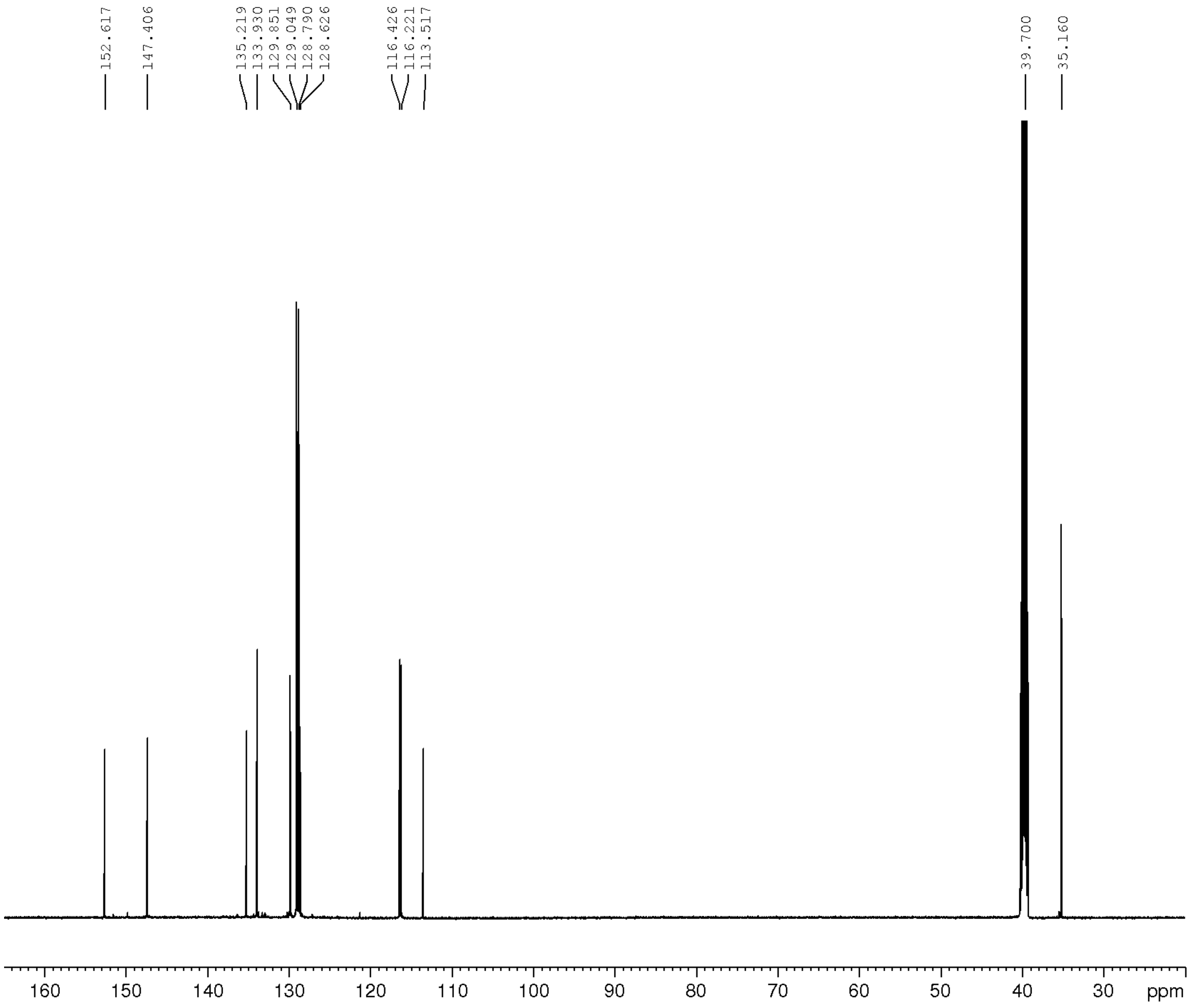

Standard 13C
143047
PGY0747_1
Pusztai Gyongyver
2024.09.13. (KP)

Current Data Parameters		
NAME	143047	
EXPNO	22	
PROCNO	1	
F2 - Acquisition Parameters		
Date_	20240914	
Time	0.01	h
INSTRUM	spect	
PROBHD	Z145856_0002 (	
PULPROG	zgpg30	
TD	65536	
SOLVENT	DMSO	
NS	2048	
DS	4	
SWH	36231.883	Hz
FIDRES	1.105709	Hz
AQ	0.9043968	sec
RG	196.07	
DW	13.800	usec
DE	18.00	usec
TE	295.0	K
D1	1.00000000	sec
D11	0.03000000	sec
TD0	1	
SFO1	150.8852070	MHz
NUC1	13C	
P1	9.90	usec
PLW1	71.00000000	W
SFO2	600.0024000	MHz
NUC2	1H	
CPDPRG[2	waltz16	
PCPD2	80.00	usec
PLW2	32.90000153	W
PLW12	0.70370001	W
PLW13	0.35339001	W
F2 - Processing parameters		
SI	131072	
SF	150.8701603	MHz
WDW	EM	
SSB	0	
LB	1.00	Hz
GB	0	
PC	1.40	

IR spectrum of **5b**

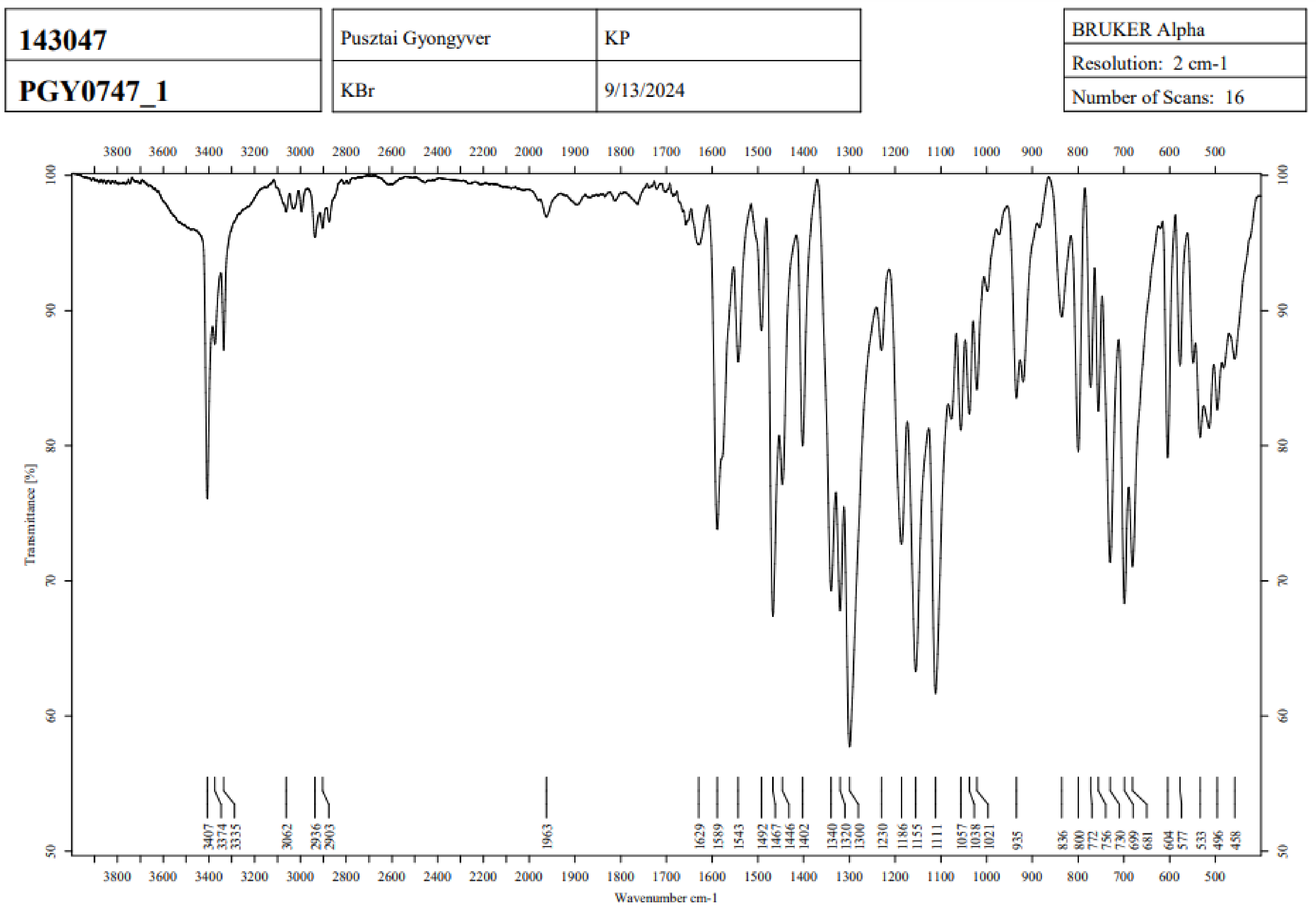

HRMS spectrum of **5b**

Spectrum Plot Report

Name	PGY0747_1, Pusztai Gyongyver	Rack Pos.		Instrument	7250A with DIP	Operator	MM
Inj. Vol. (ul)	0.5	Plate Pos.		IRM Status	Success		
Data File	143047msqtof_dip.D	Method (Acq)	DIP_70eV.M	Comment		Acq. Time (Local)	9/30/2024 1:08:59 PM (UTC+02:00)

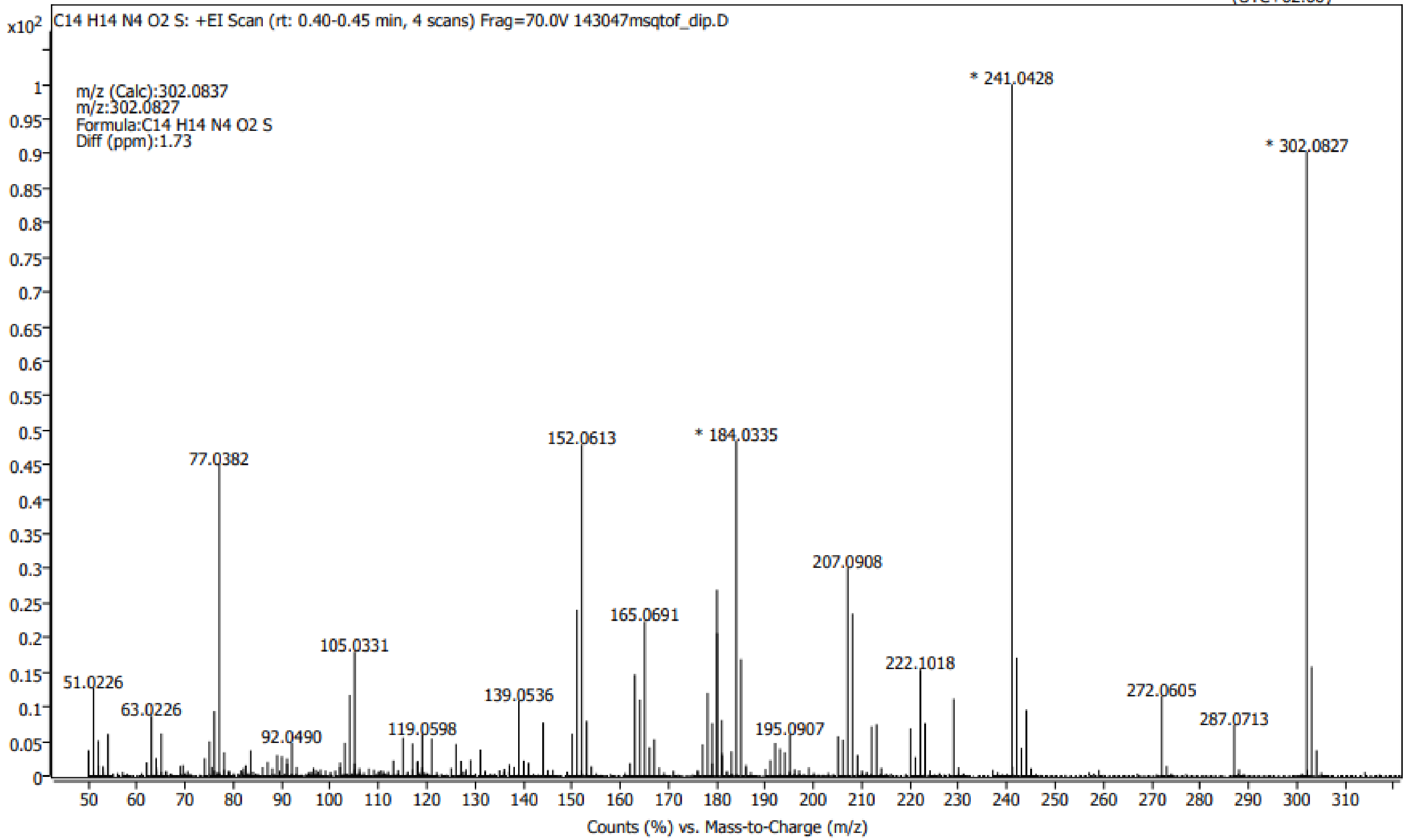

^{1}H NMR spectrum of **7e**

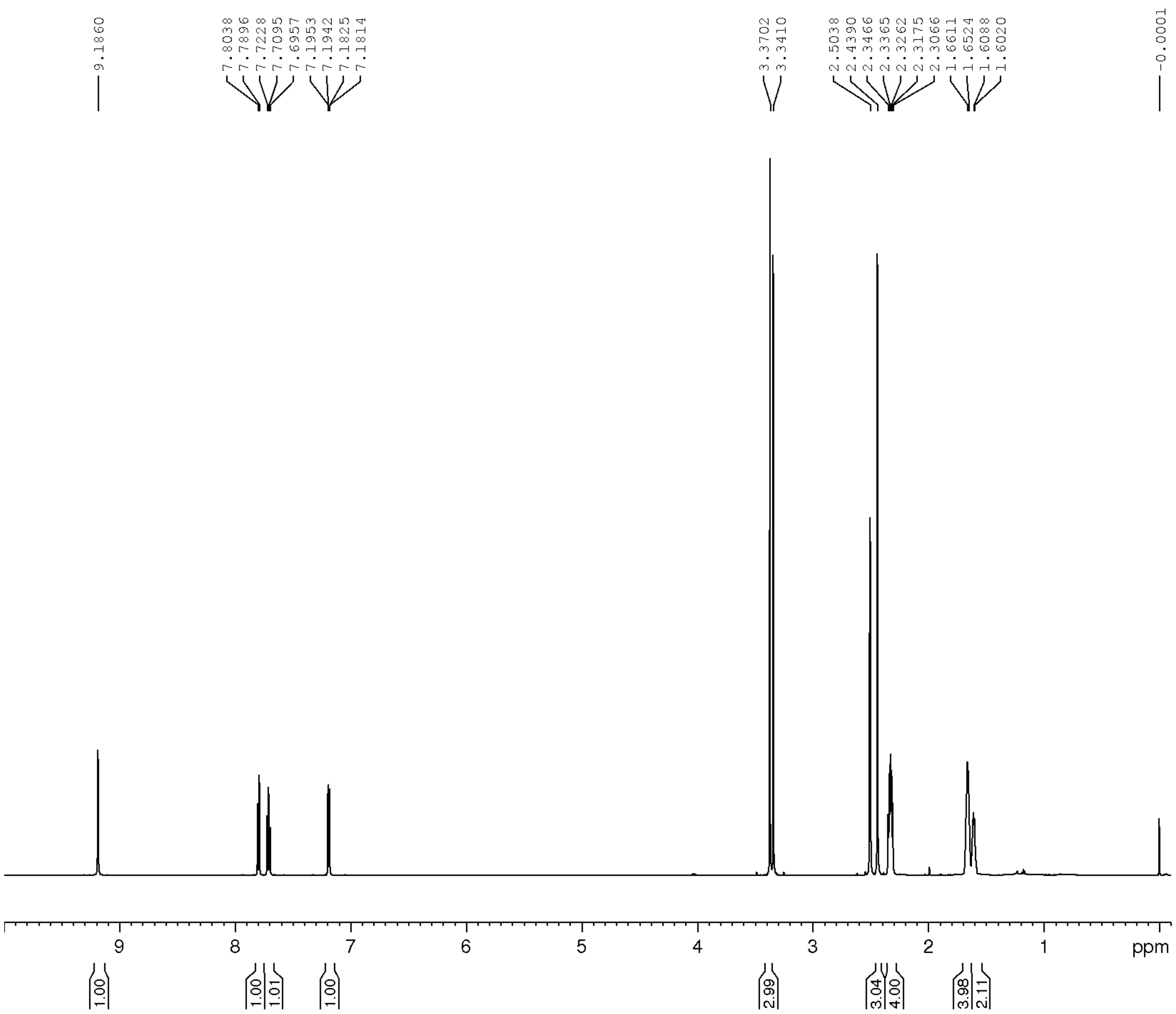

^{13}C NMR spectrum of **7e**

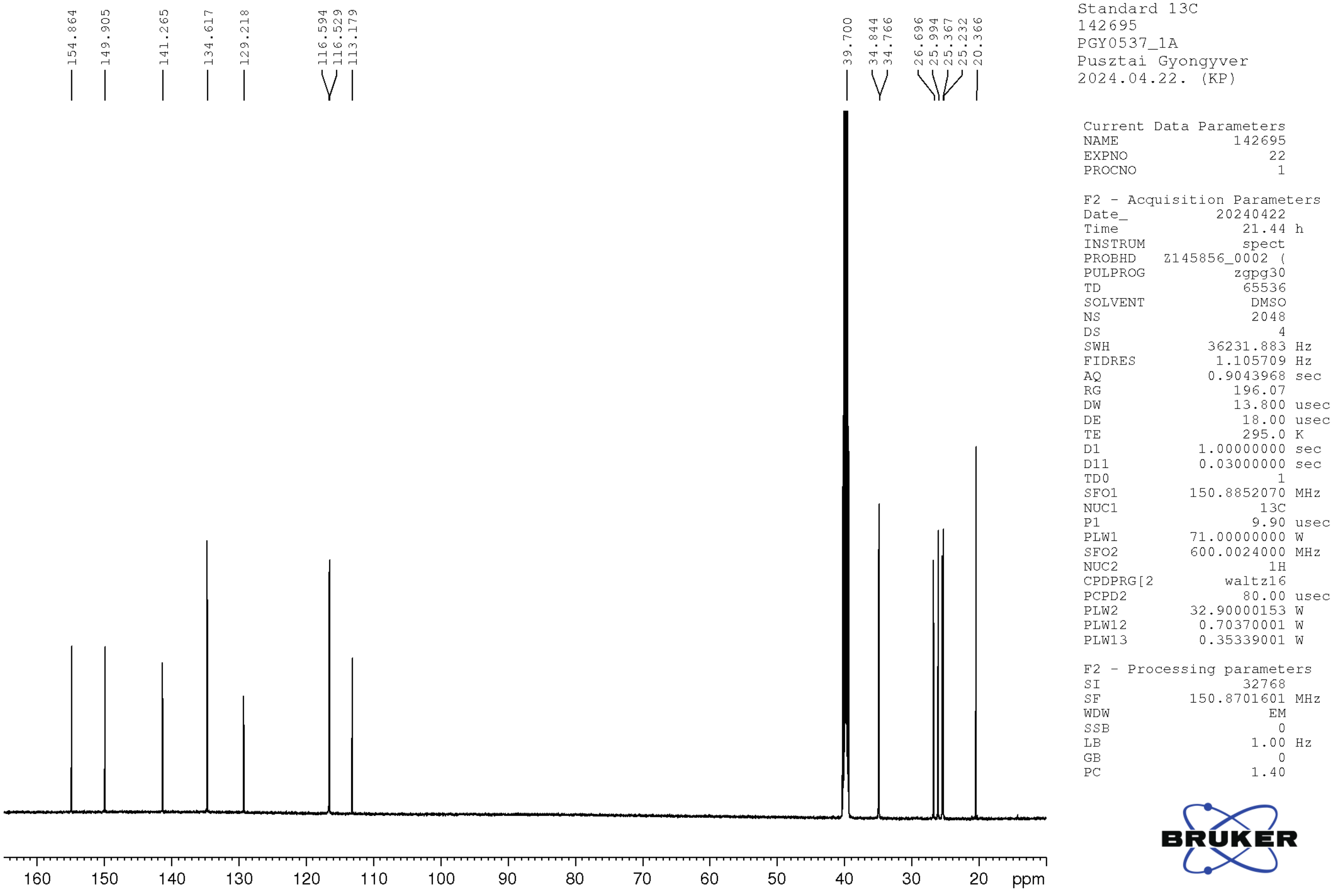

IR spectrum of **7e**

**142695**	Pusztai Gyongyver	KP		BRUKER Alpha
**PGY0537_1A**	KBr	4/23/2024		Resolution: 2 cm-1
				Number of Scans: 16

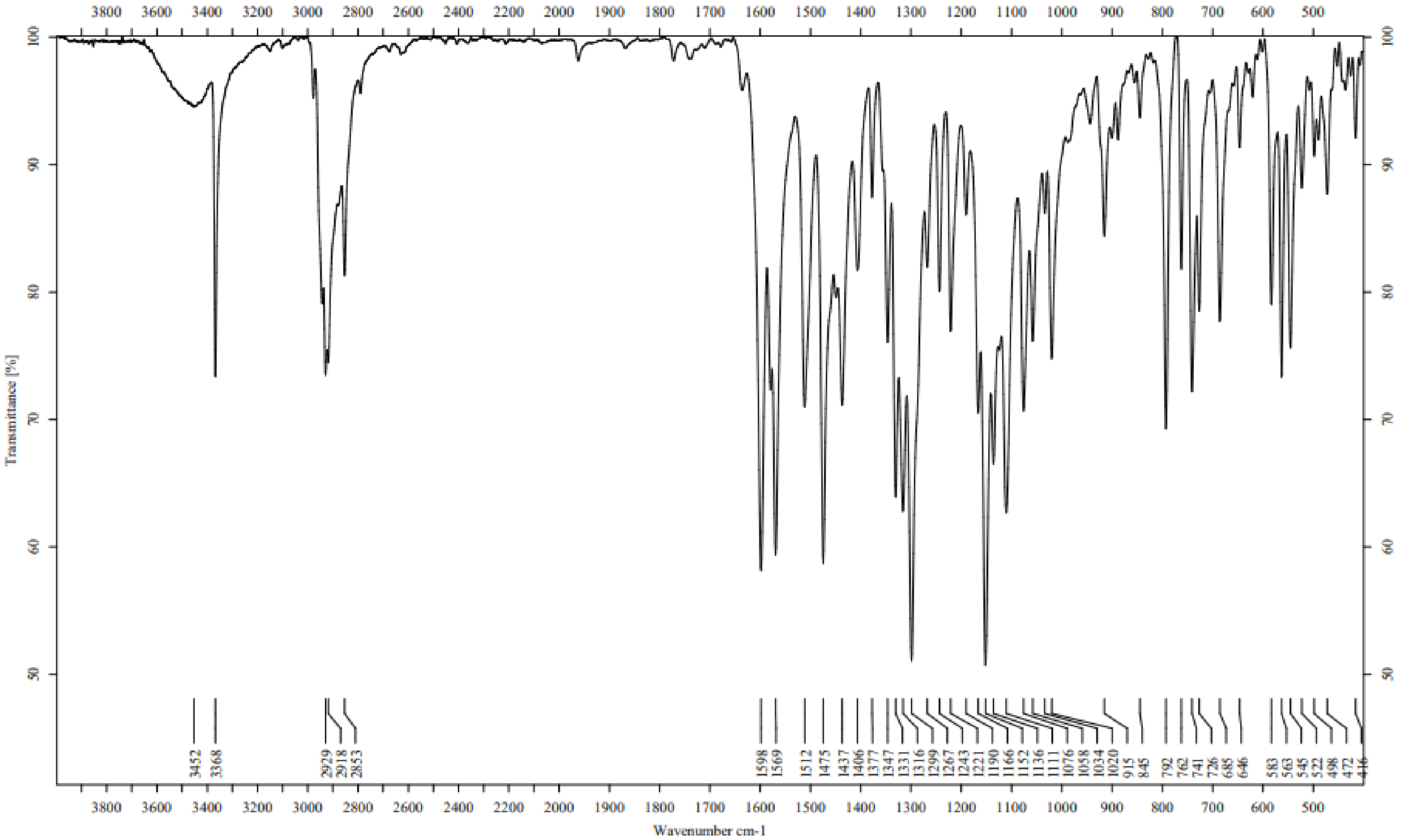

HRMS spectrum of **7e**

Sample: PGY0537_1A	Lab code: Nsz - 28997
Submitter: Pusztai Gyongyve	Project: Other

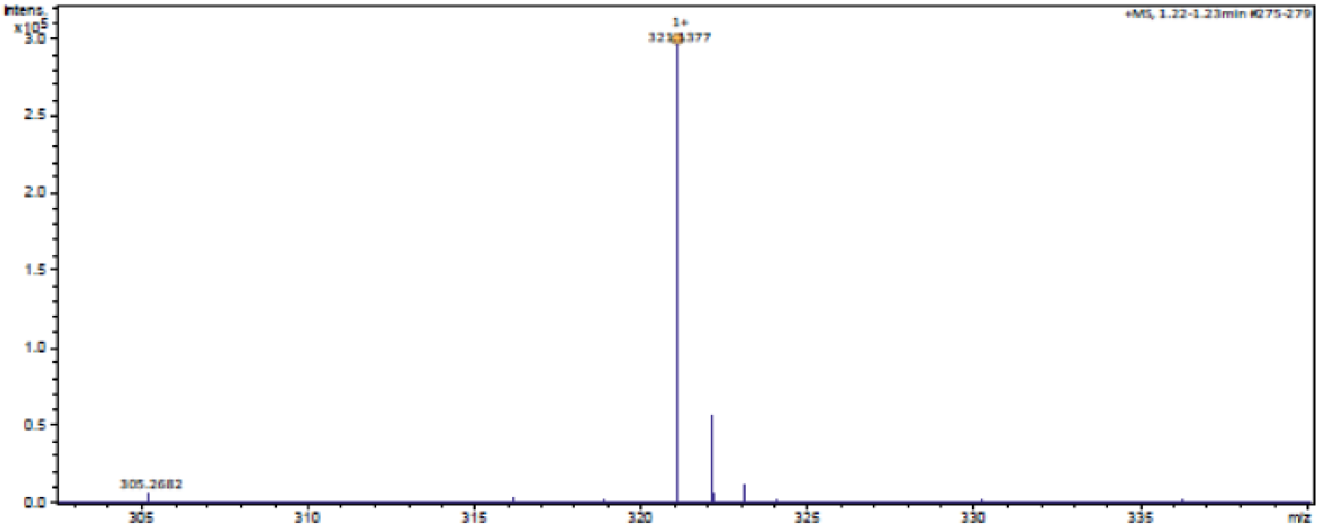

Meas. m/z	Ion Formula	m/z	|err| [ppm]
321.1377	C15H21N4O2S	321.1385	1.0

^{1}H NMR spectrum of (*E*)-**7h**

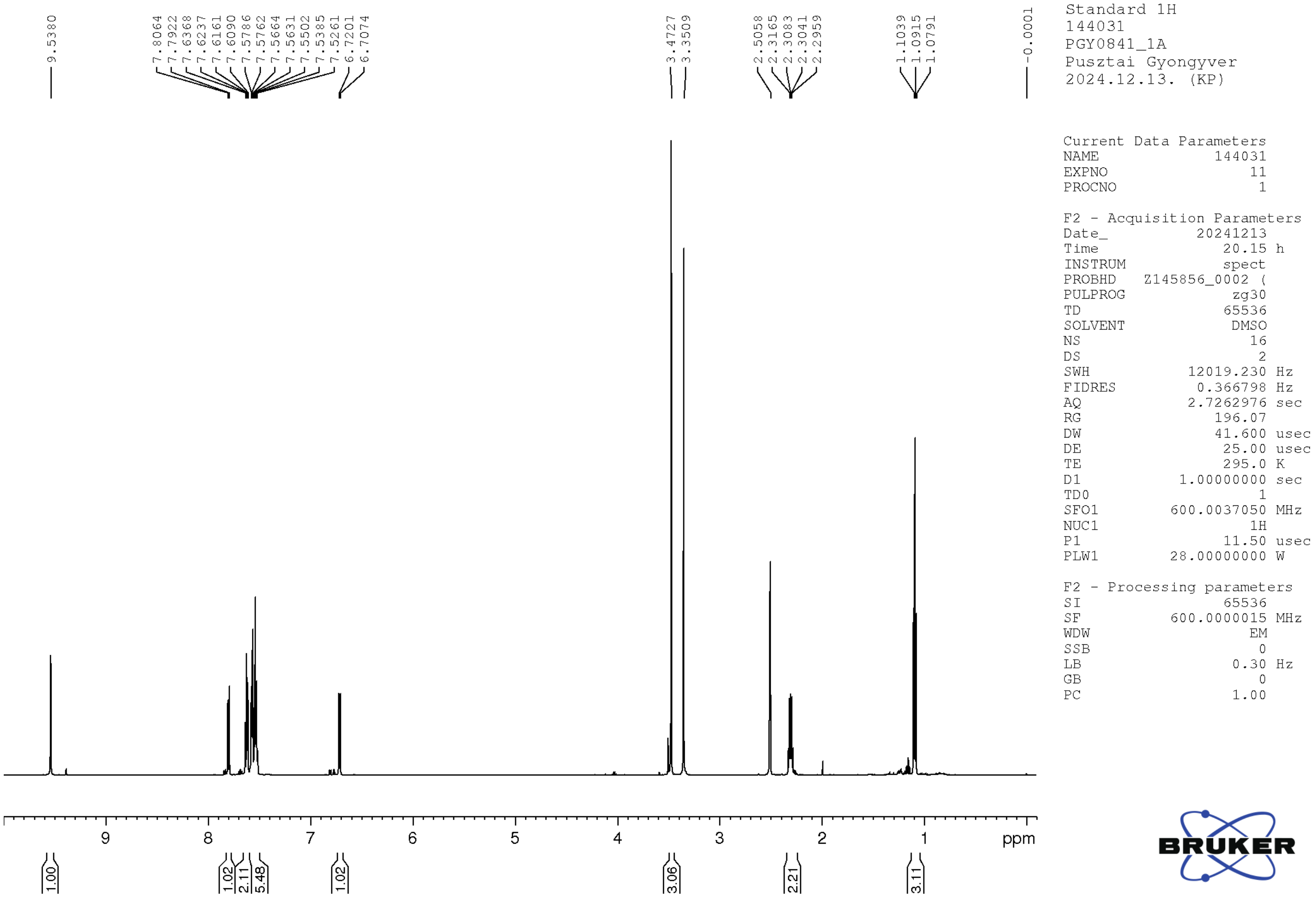

^{13}C NMR spectrum of (*E*)-**7h**

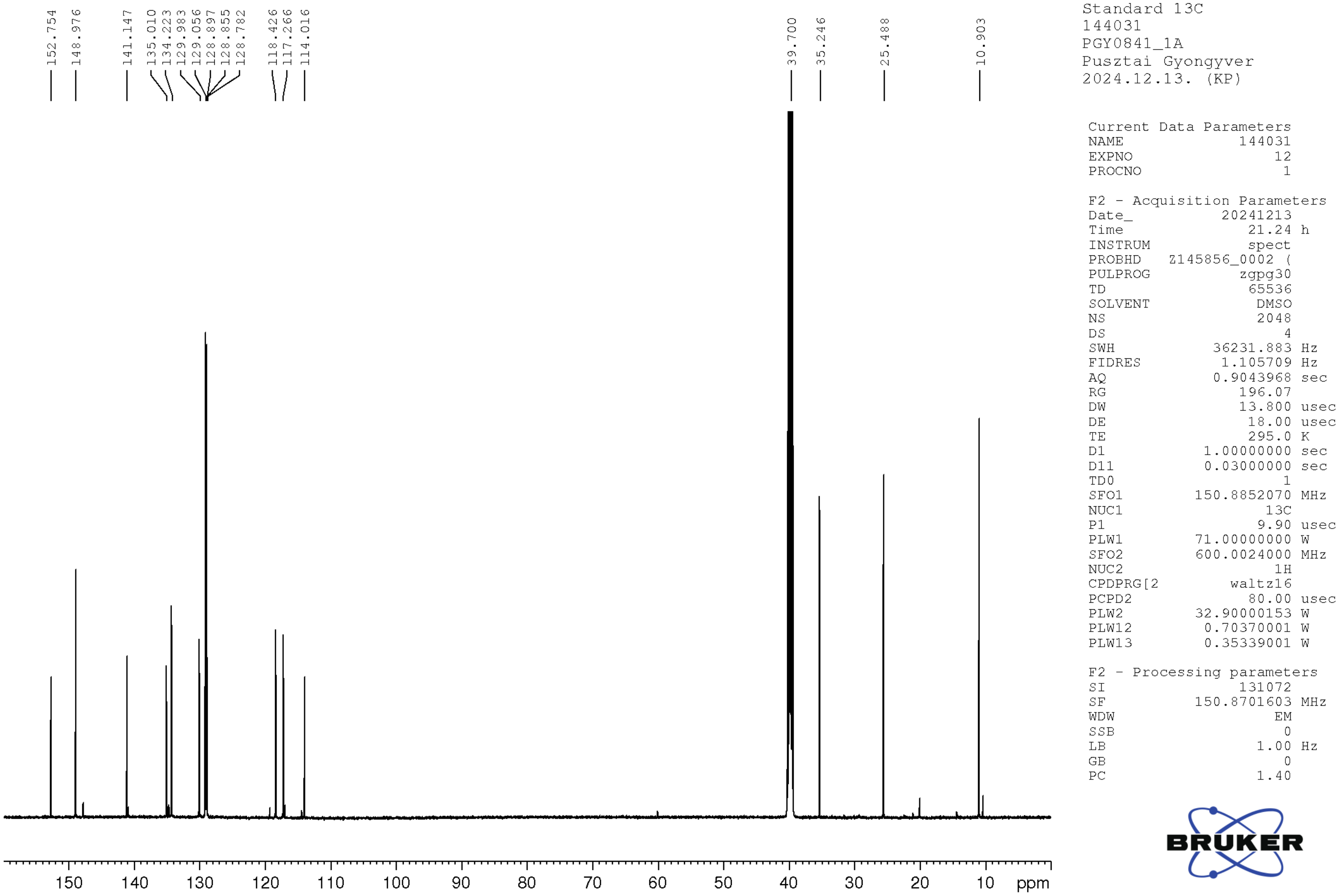

IR spectrum of (*E*)-**7h**

144031	Pusztai Gyongyver	KP
PGY0841_1A	KBr	12/16/2024

BRUKER Alpha
Resolution: 2 cm-1
Number of Scans: 16

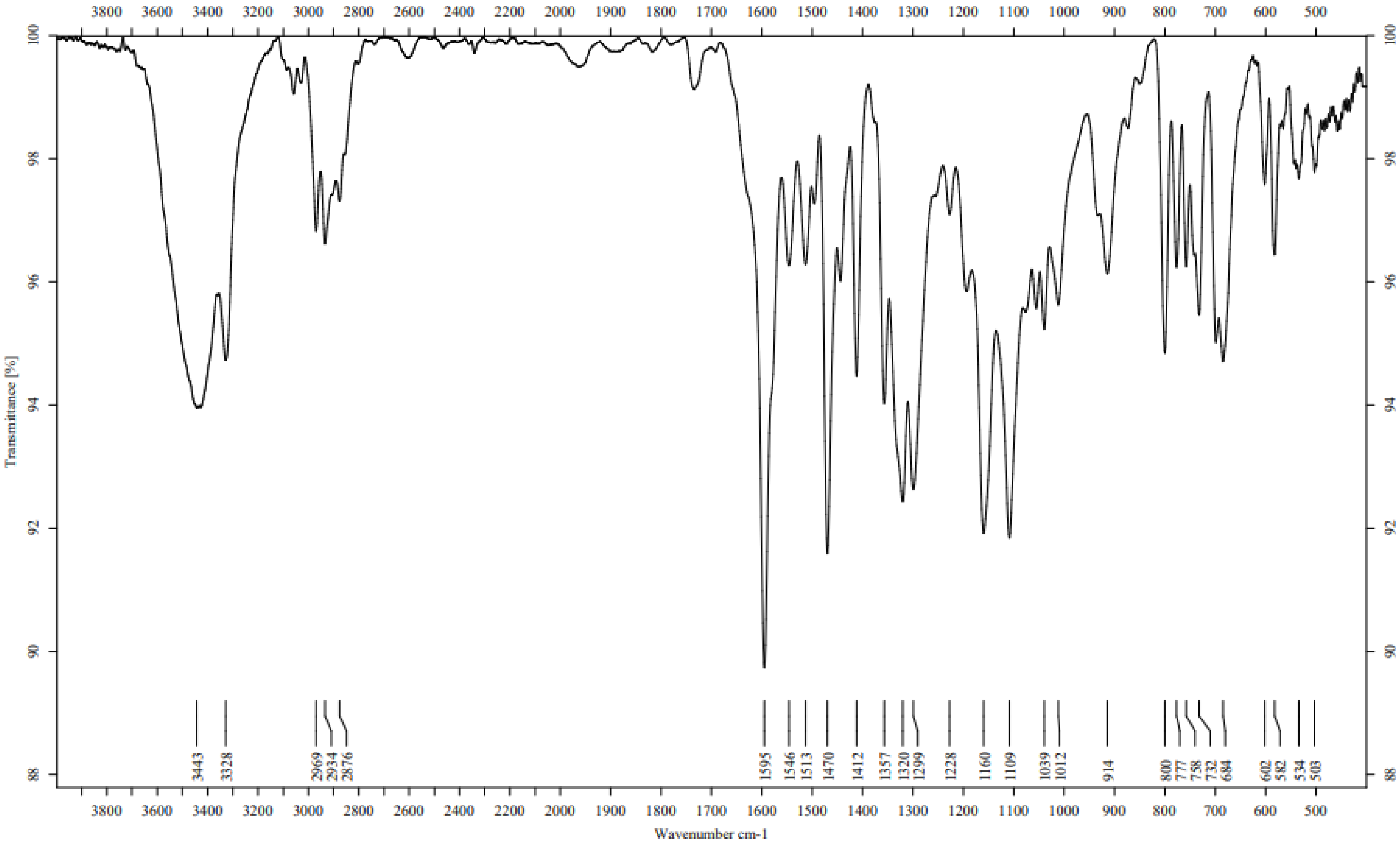

HRMS spectrum of (*E*)-**7h**

Spectrum Plot Report

Name	PGY0841_1A, Pusztai Gyongyver	Rack Pos.		Instrument	7250A with DIP	Operator	MM
Inj. Vol. (ul)	0.5	Plate Pos.		IRM Status	Success		
Data File	144031_qtof_dip.D	Method (Acq)	DIP_70eV.M	Comment		Acq. Time (Local)	1/3/2025 11:33:10 AM (UTC+01:00)

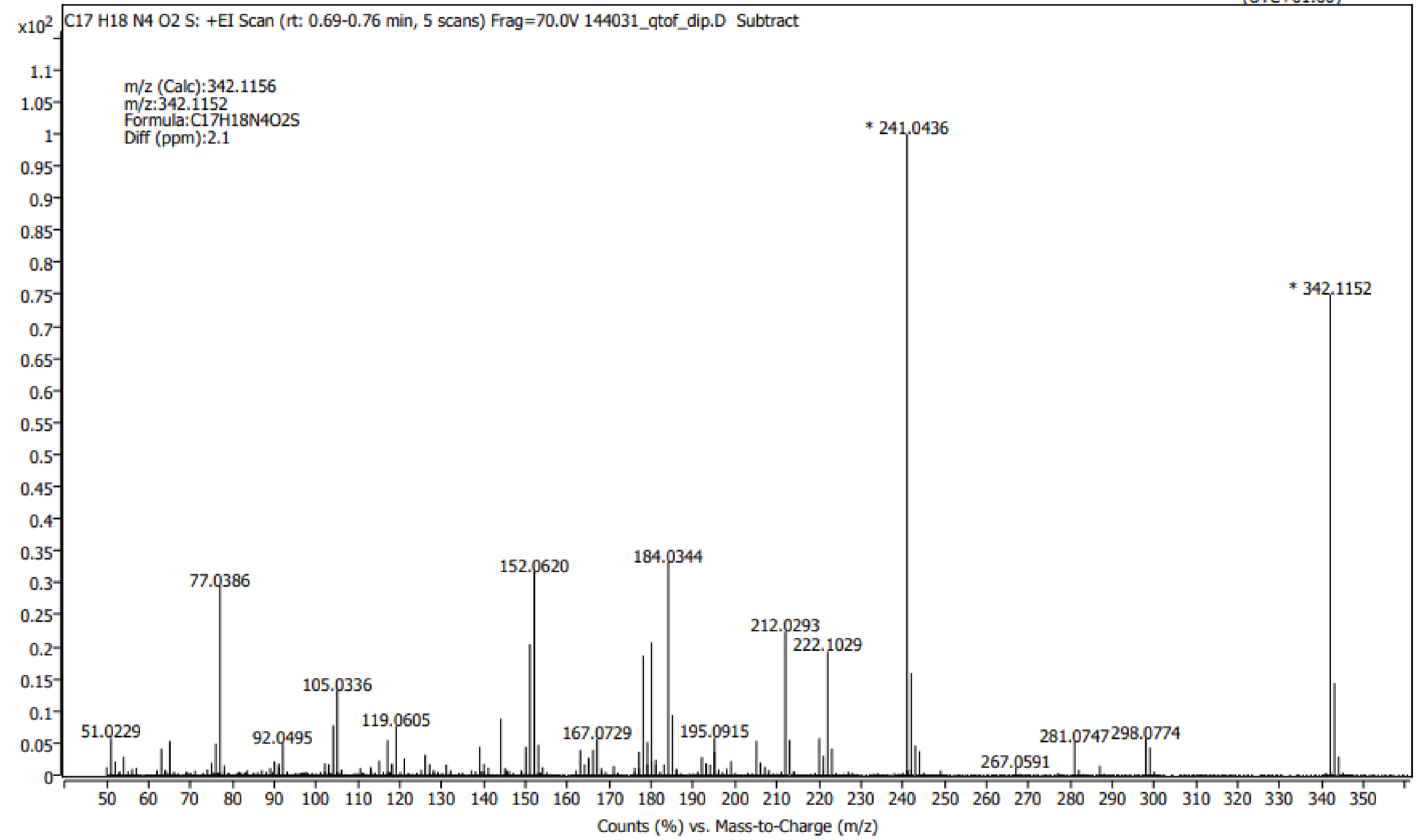

^{1}H NMR spectrum of (*Z*)-**7h**

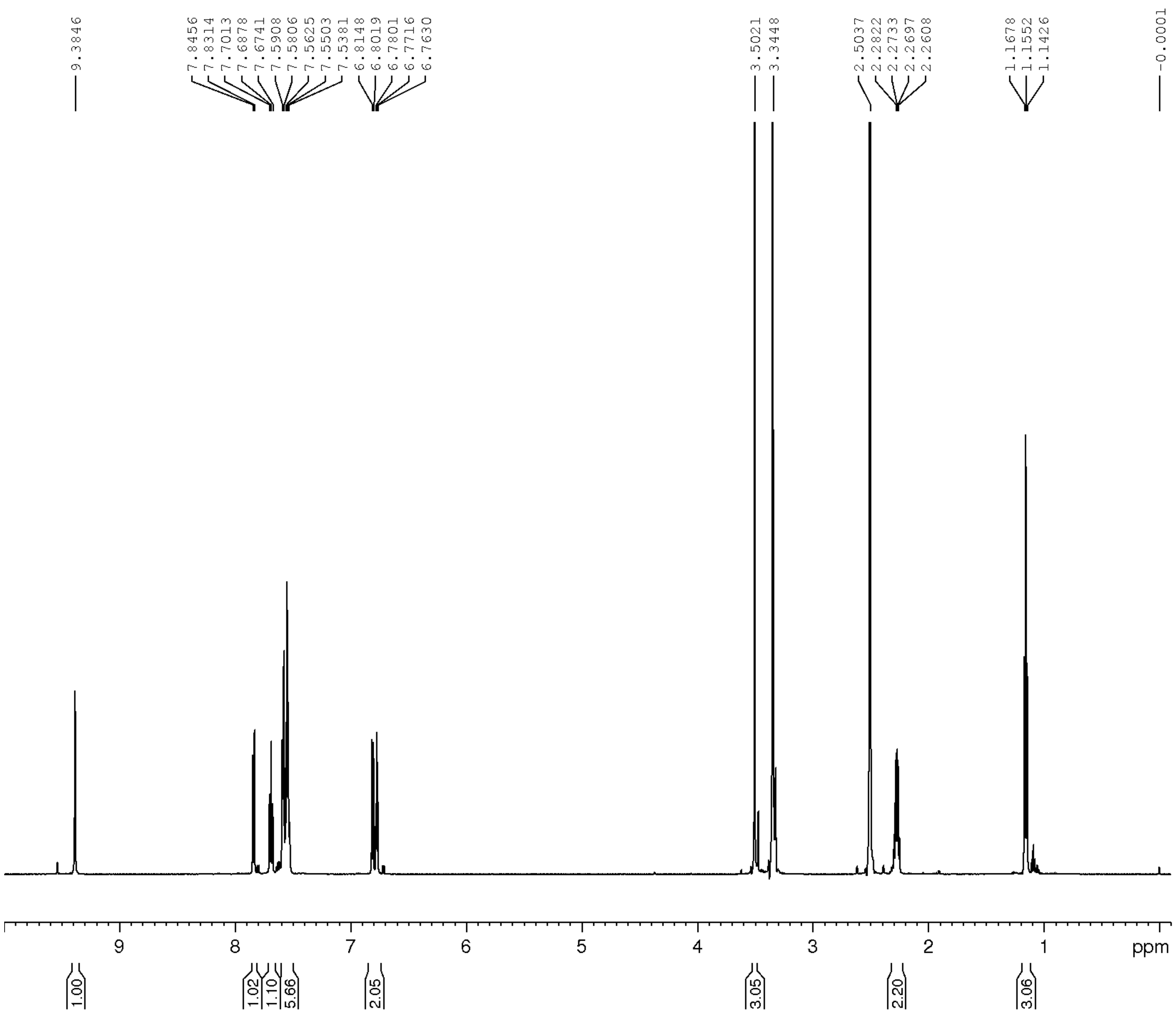

Standard 1H
144038
PGY0792_1A
Pusztai Gyongyver
2024.12.17. (KP)

Current Data Parameters
NAME 144038
EXPNO 11
PROCNO 1

F2 - Acquisition Parameters
Date_ 20241217
Time 13.20 h
INSTRUM spect
PROBHD Z145856_0002 (
PULPROG zg30
TD 65536
SOLVENT DMSO
NS 16
DS 2
SWH 12019.230 Hz
FIDRES 0.366798 Hz
AQ 2.7262976 sec
RG 196.07
DW 41.600 usec
DE 25.00 usec
TE 295.0 K
D1 1.00000000 sec
TD0 1
SFO1 600.0037050 MHz
NUC1 1H
P1 11.50 usec
PLW1 28.00000000 W

F2 - Processing parameters
SI 65536
SF 600.0000028 MHz
WDW EM
SSB 0
LB 0.30 Hz
GB 0
PC 1.00

^{13}C NMR spectrum of (*Z*)-**7h**

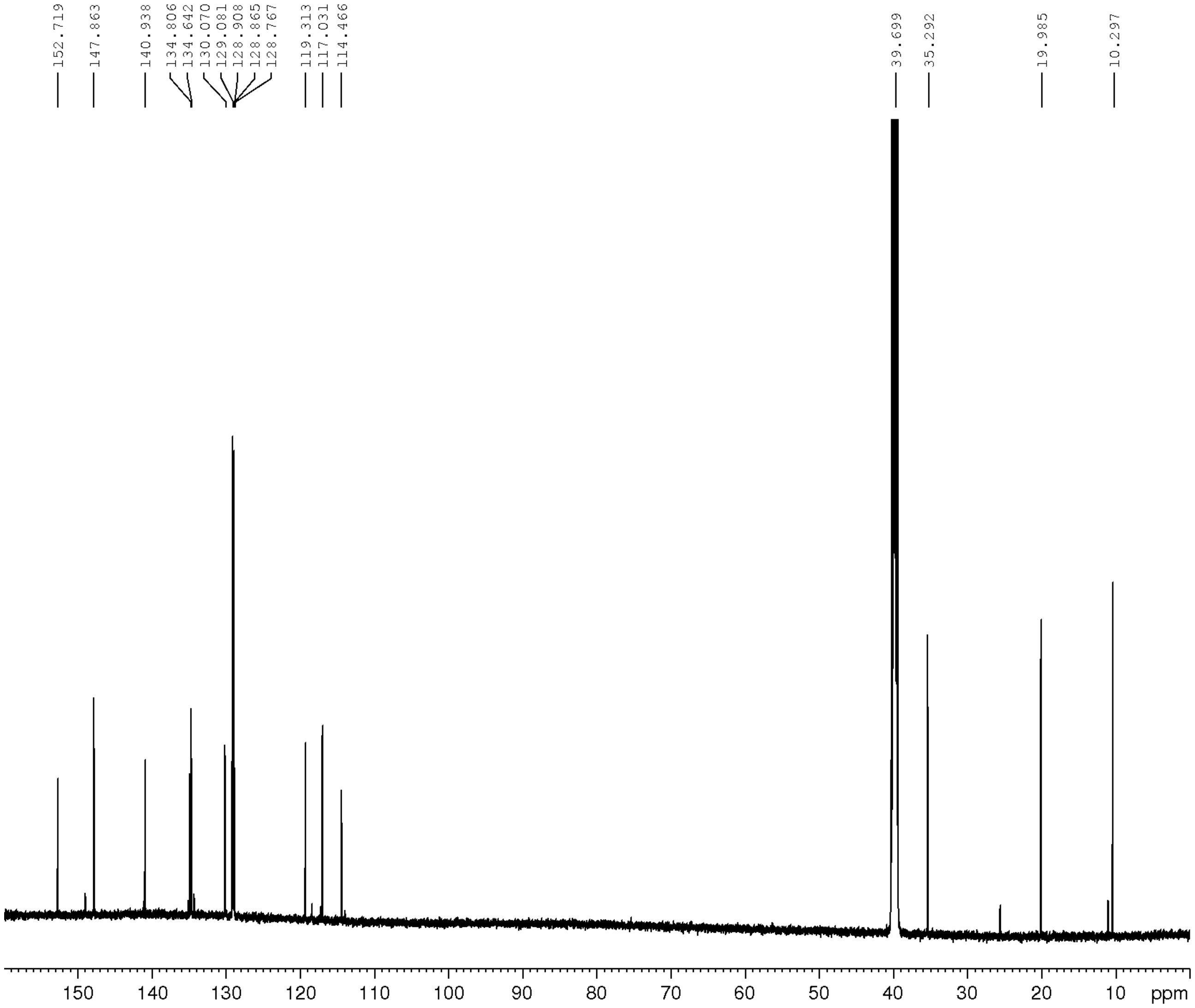

Standard 13C
144038
PGY0792_1A
Pusztai Gyongyver
2024.12.18. (KP)

Current Data Parameters
NAME 144038
EXPNO 12
PROCNO 1

F2 - Acquisition Parameters
Date_ 20241218
Time 18.21 h
INSTRUM spect
PROBHD Z145856_0002 (
PULPROG zgpg30
TD 65536
SOLVENT DMSO
NS 2048
DS 4
SWH 36231.883 Hz
FIDRES 1.105709 Hz
AQ 0.9043968 sec
RG 196.07
DW 13.800 usec
DE 18.00 usec
TE 295.0 K
D1 1.00000000 sec
D11 0.03000000 sec
TD0 1
SFO1 150.8852070 MHz
NUC1 13C
P1 9.90 usec
PLW1 71.00000000 W
SFO2 600.0024000 MHz
NUC2 1H
CPDPRG[2 waltz16
PCPD2 80.00 usec
PLW2 32.90000153 W
PLW12 0.70370001 W
PLW13 0.35339001 W

F2 - Processing parameters
SI 131072
SF 150.8701601 MHz
WDW EM
SSB 0
LB 1.00 Hz
GB 0
PC 1.40

IR spectrum of (*Z*)-**7h**

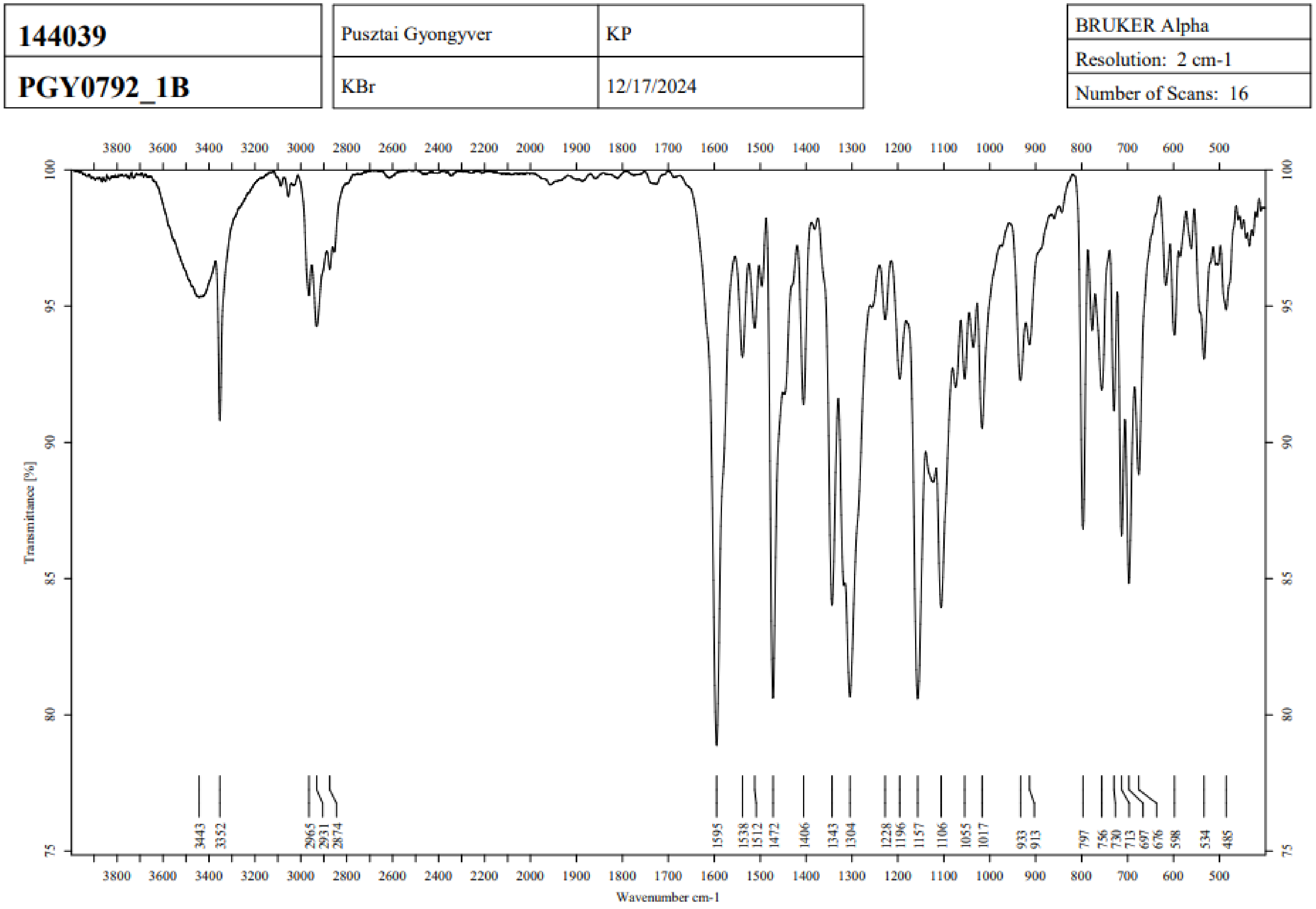

HRMS spectrum of (*Z*)-**7h**

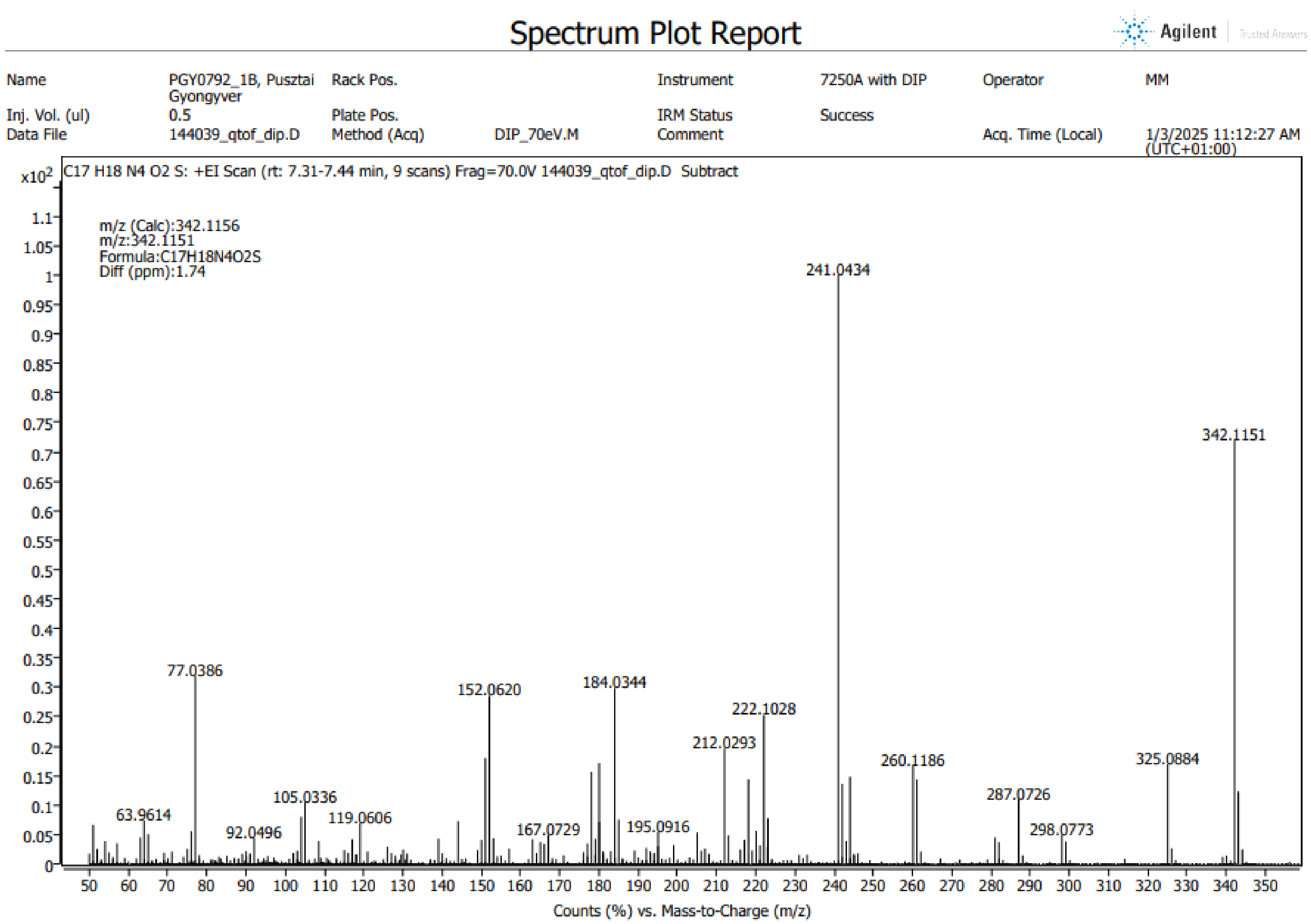

3

^{1}H NMR spectrum of **3e**

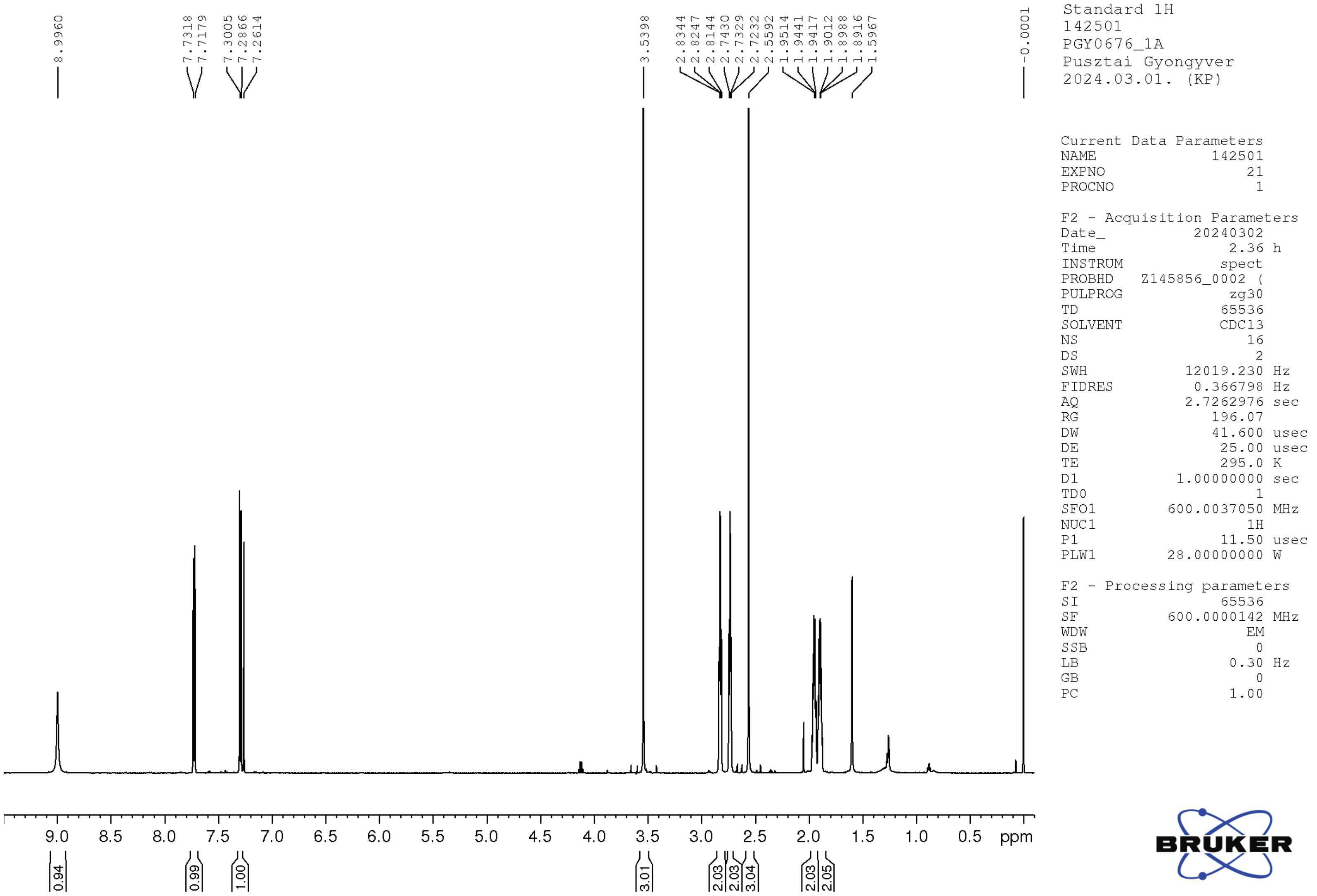

^{13}C NMR spectrum of **3e**

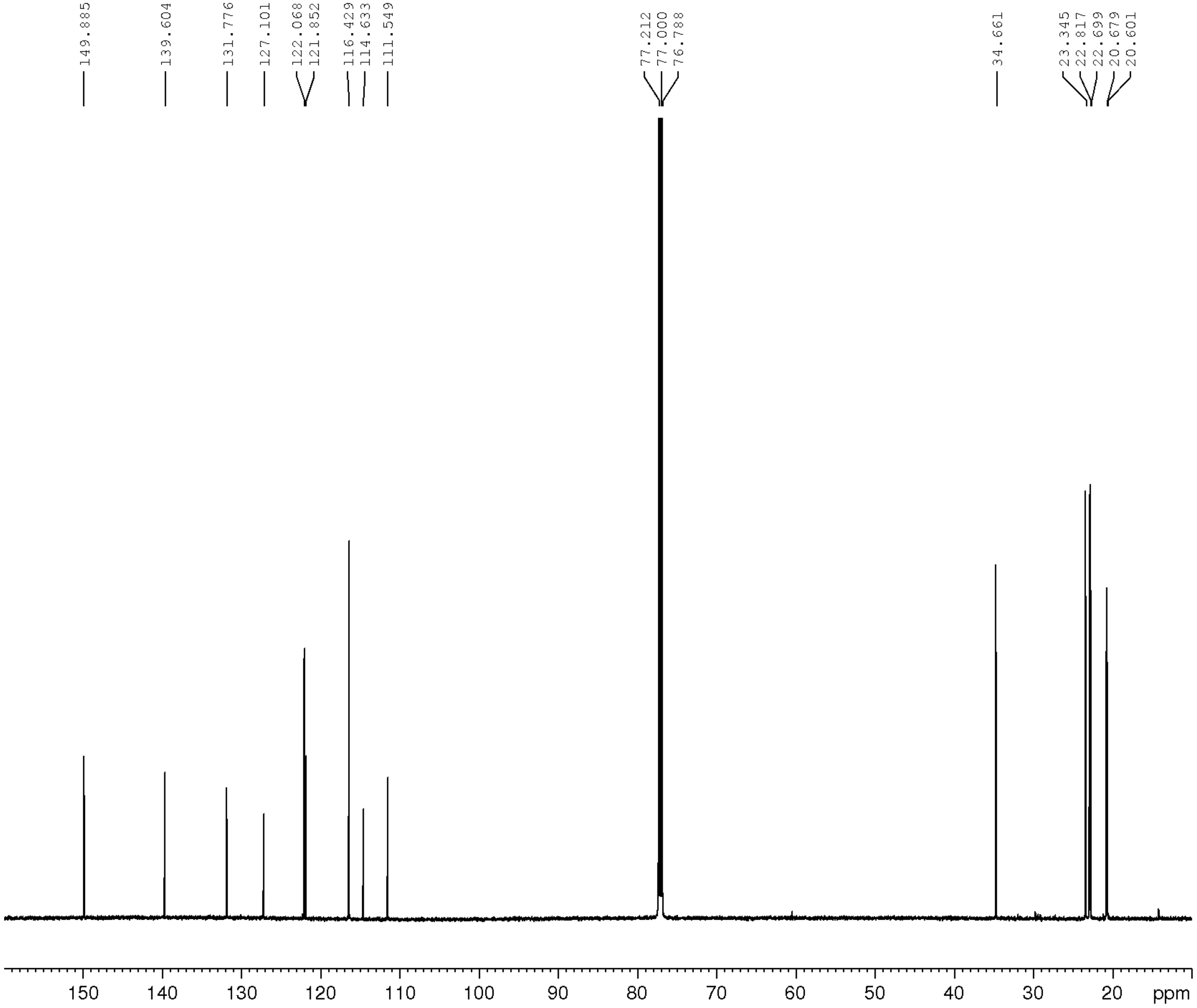

Standard 13C
142501
PGY0676_1A
Pusztai Gyongyver
2024.03.01. (KP)

Current Data Parameters
NAME 142501
EXPNO 22
PROCNO 1

F2 - Acquisition Parameters
Date_ 20240302
Time 3.45 h
INSTRUM spect
PROBHD Z145856_0002 (
PULPROG zgpg30
TD 65536
SOLVENT CDCl3
NS 2048
DS 4
SWH 36231.883 Hz
FIDRES 1.105709 Hz
AQ 0.9043968 sec
RG 196.07
DW 13.800 usec
DE 18.00 usec
TE 295.0 K
D1 1.00000000 sec
D11 0.03000000 sec
TD0 1
SFO1 150.8852070 MHz
NUC1 13C
P1 9.90 usec
PLW1 71.00000000 W
SFO2 600.0024000 MHz
NUC2 1H
CPDPRG[2 waltz16
PCPD2 80.00 usec
PLW2 32.90000153 W
PLW12 0.70370001 W
PLW13 0.35339001 W

F2 - Processing parameters
SI 32768
SF 150.8701265 MHz
WDW EM
SSB 0
LB 1.00 Hz
GB 0
PC 1.40

IR spectrum of **3e**

**142501**	Pusztai Gyongyver	KP	BRUKER Alpha
**PGY0676_1A**	KBr	3/1/2024	Resolution: 2 cm-1
			Number of Scans: 16

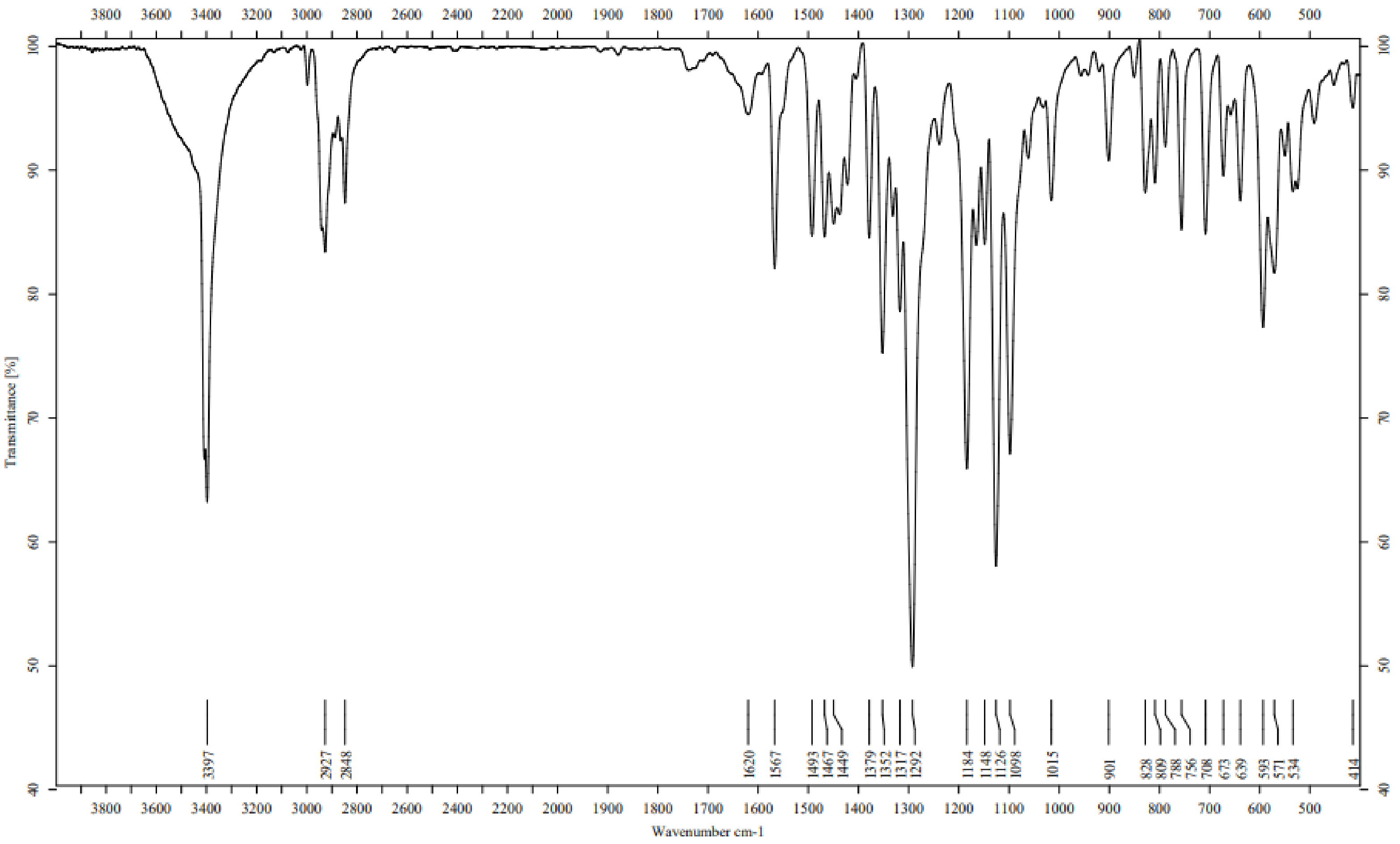

HRMS spectrum of **3e**

Spectrum Plot Report

Agilent

Name	PGY0676_1A, Pusztai Gyongyver	Rack Pos.		Instrument	7250A with DIP	Operator	MM
Inj. Vol. (ul)	0.5	Plate Pos.		IRM Status	Success		
Data File	142501.D	Method (Acq)	DIP_70eV.M	Comment		Acq. Time (Local)	3/5/2024 10:57:51 AM (UTC+01:00)

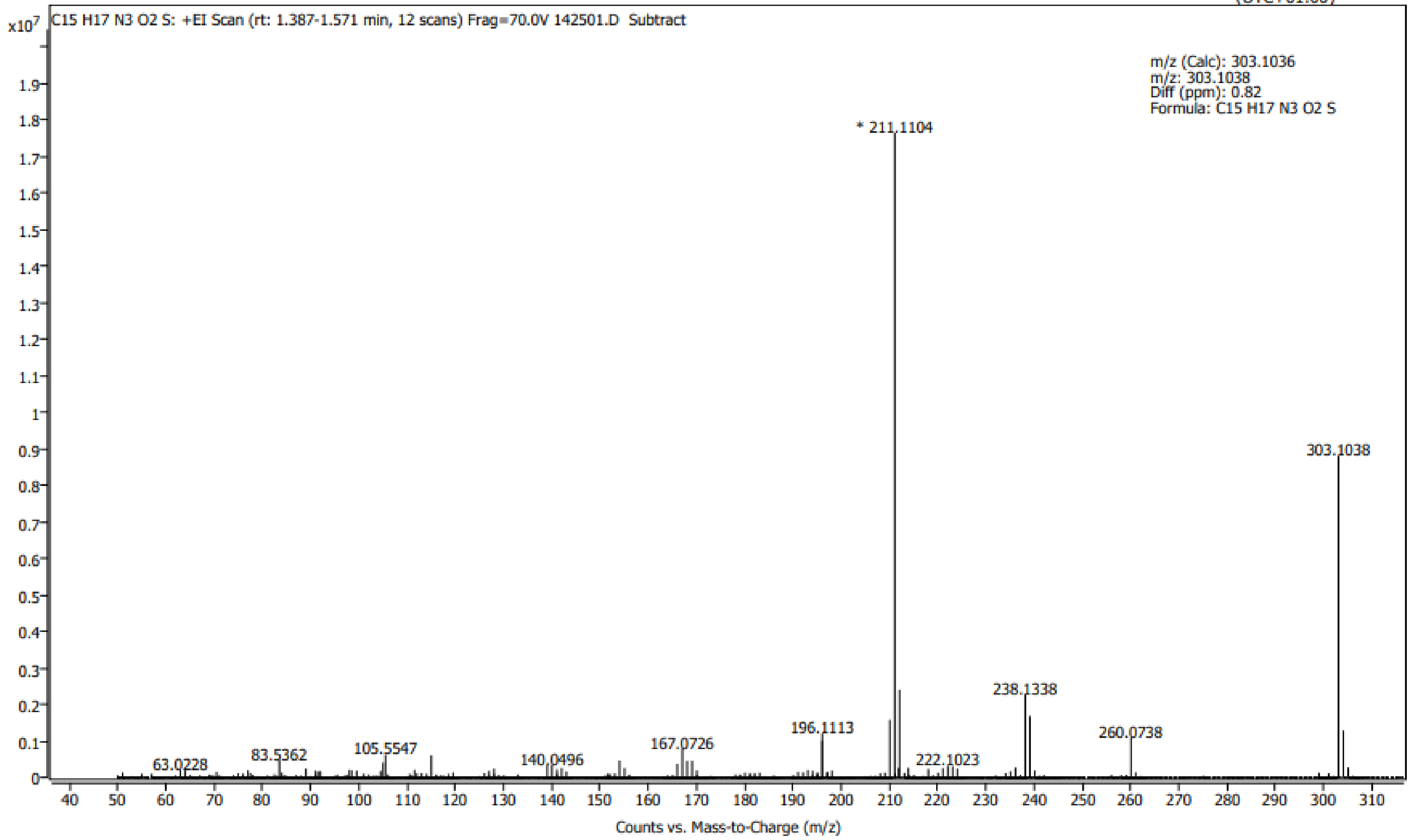

^{1}H NMR spectrum of **3h**

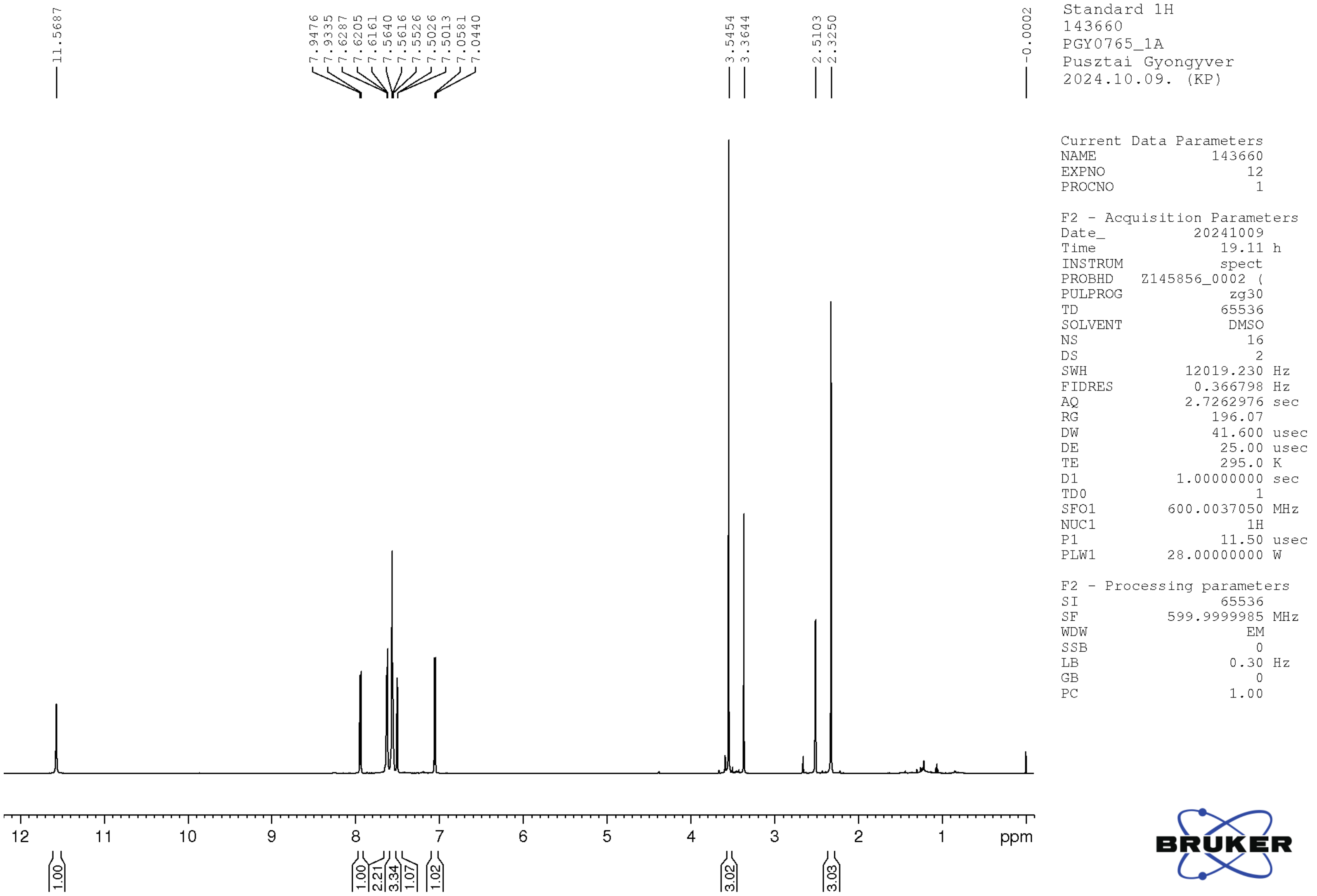

^{13}C NMR spectrum of **3h**

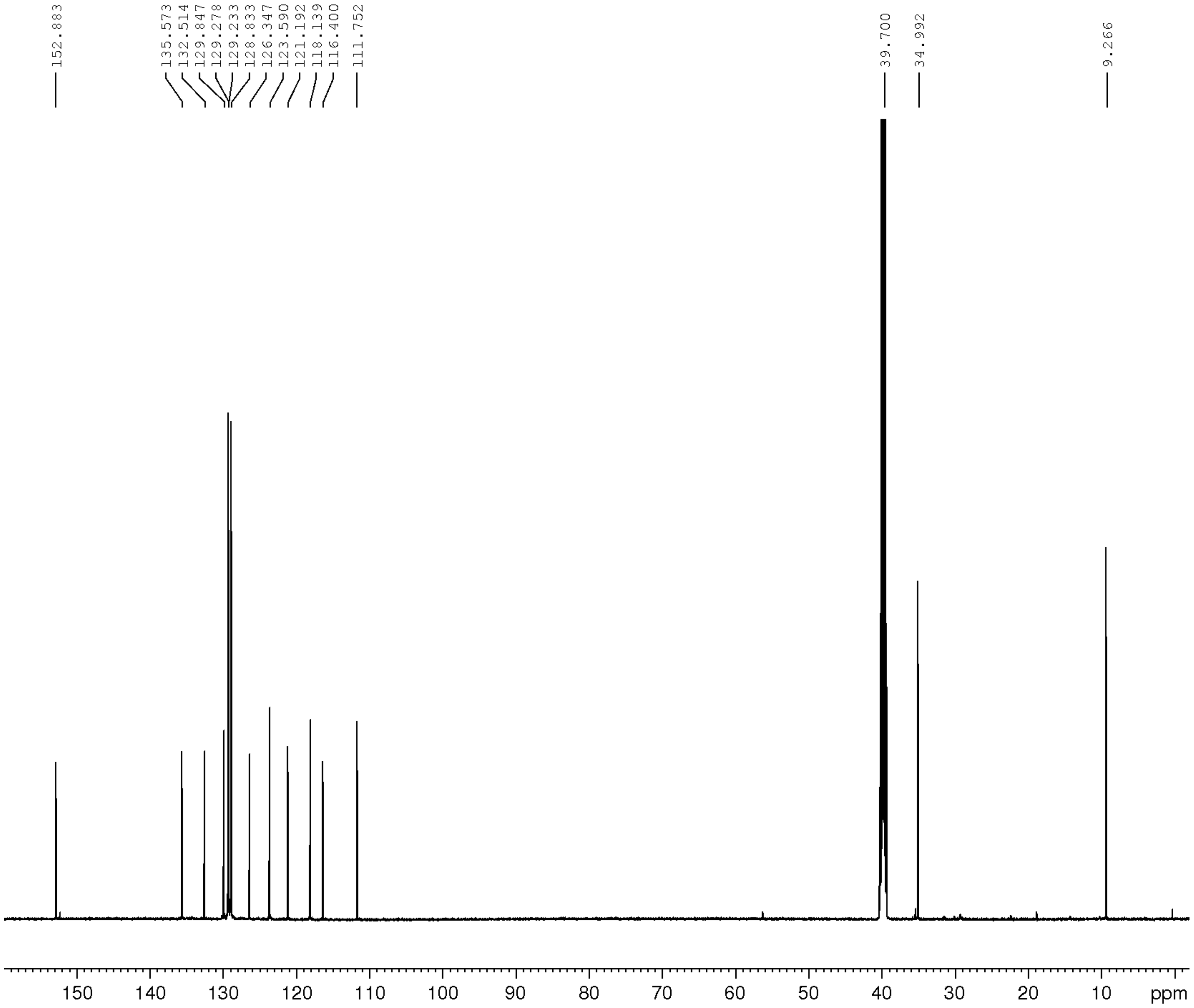

Standard 13C
143660
PGY0765_1A
Pusztai Gyongyver
2024.10.09. (KP)

Current Data Parameters
NAME 143660
EXPNO 13
PROCNO 1

F2 - Acquisition Parameters
Date_ 20241009
Time 20.21 h
INSTRUM spect
PROBHD Z145856_0002 (
PULPROG zgpg30
TD 65536
SOLVENT DMSO
NS 2048
DS 4
SWH 36231.883 Hz
FIDRES 1.105709 Hz
AQ 0.9043968 sec
RG 196.07
DW 13.800 usec
DE 18.00 usec
TE 295.0 K
D1 1.00000000 sec
D11 0.03000000 sec
TD0 1
SFO1 150.8852070 MHz
NUC1 13C
P1 9.90 usec
PLW1 71.00000000 W
SFO2 600.0024000 MHz
NUC2 1H
CPDPRG[2 waltz16
PCPD2 80.00 usec
PLW2 32.90000153 W
PLW12 0.70370001 W
PLW13 0.35339001 W

F2 - Processing parameters
SI 131072
SF 150.8701601 MHz
WDW EM
SSB 0
LB 1.00 Hz
GB 0
PC 1.40

IR spectrum of **3h**

**143660**	
**PGY0765_1A**	

Pusztai Gyongyver	KP
KBr	10/9/2024

BRUKER Alpha
Resolution: 2 cm-1
Number of Scans: 16

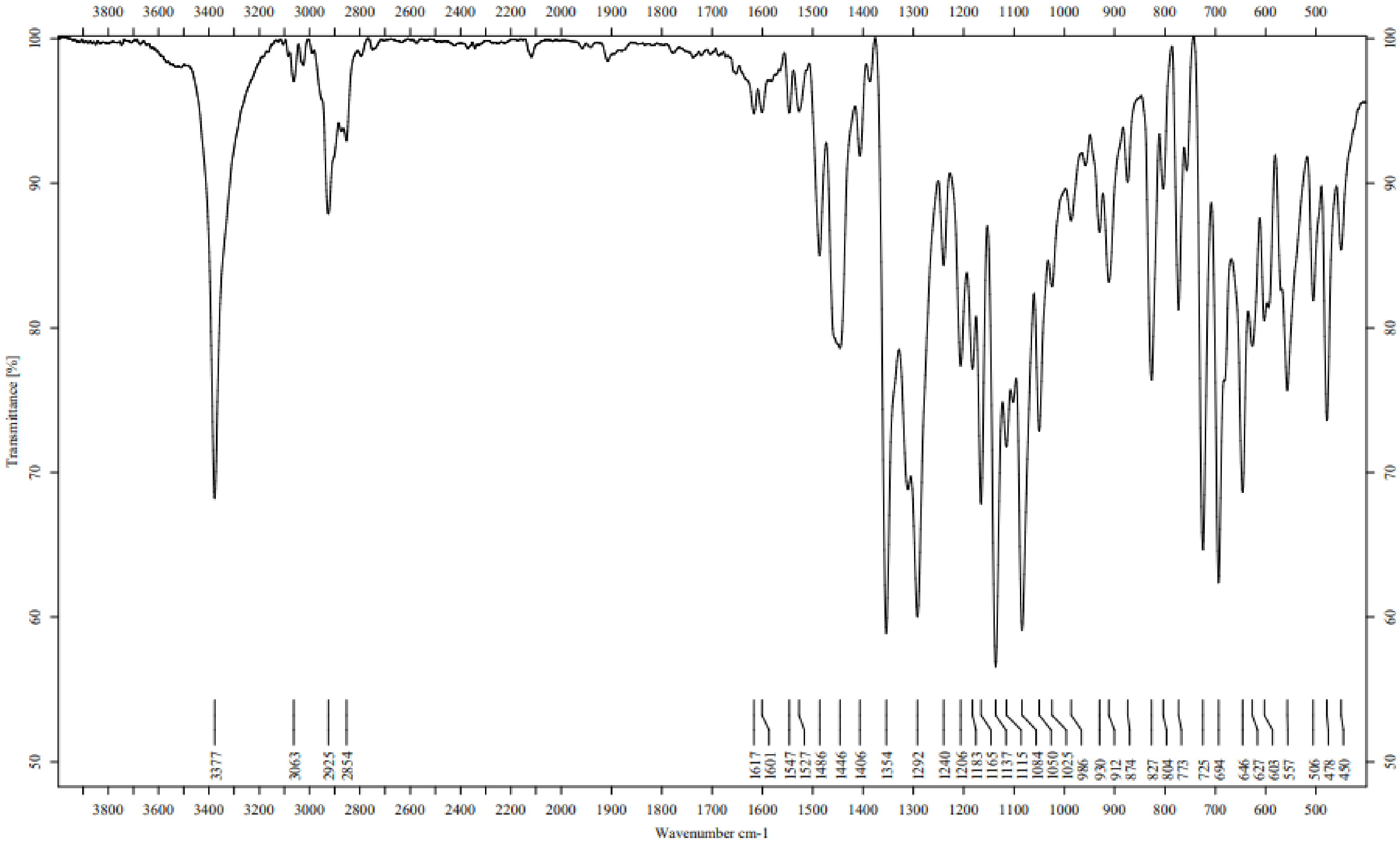

HRMS spectrum of **3h**

Spectrum Plot Report

Name	PGY0765_1A, Pusztai Gyongyver	Rack Pos.		Instrument	7250A with DIP	Operator	MM
Inj. Vol. (ul)	0.5	Plate Pos.		IRM Status	Success		
Data File	143660msaqtof_dip.D	Method (Acq)	DIP_70eV.M	Comment		Acq. Time (Local)	10/24/2024 8:35:54 AM (UTC+02:00)

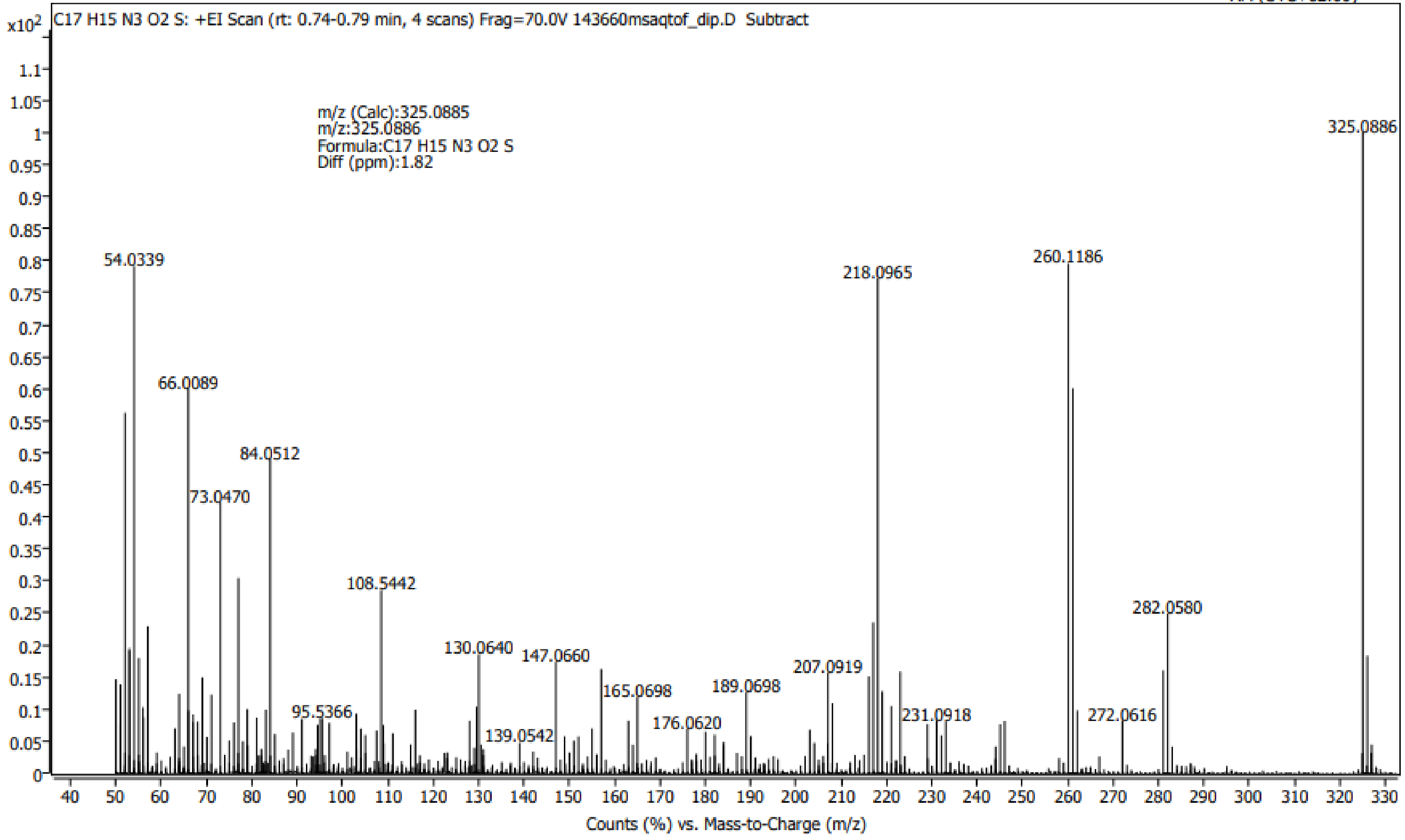

^{1}H NMR spectrum of (*E*)-**7a**

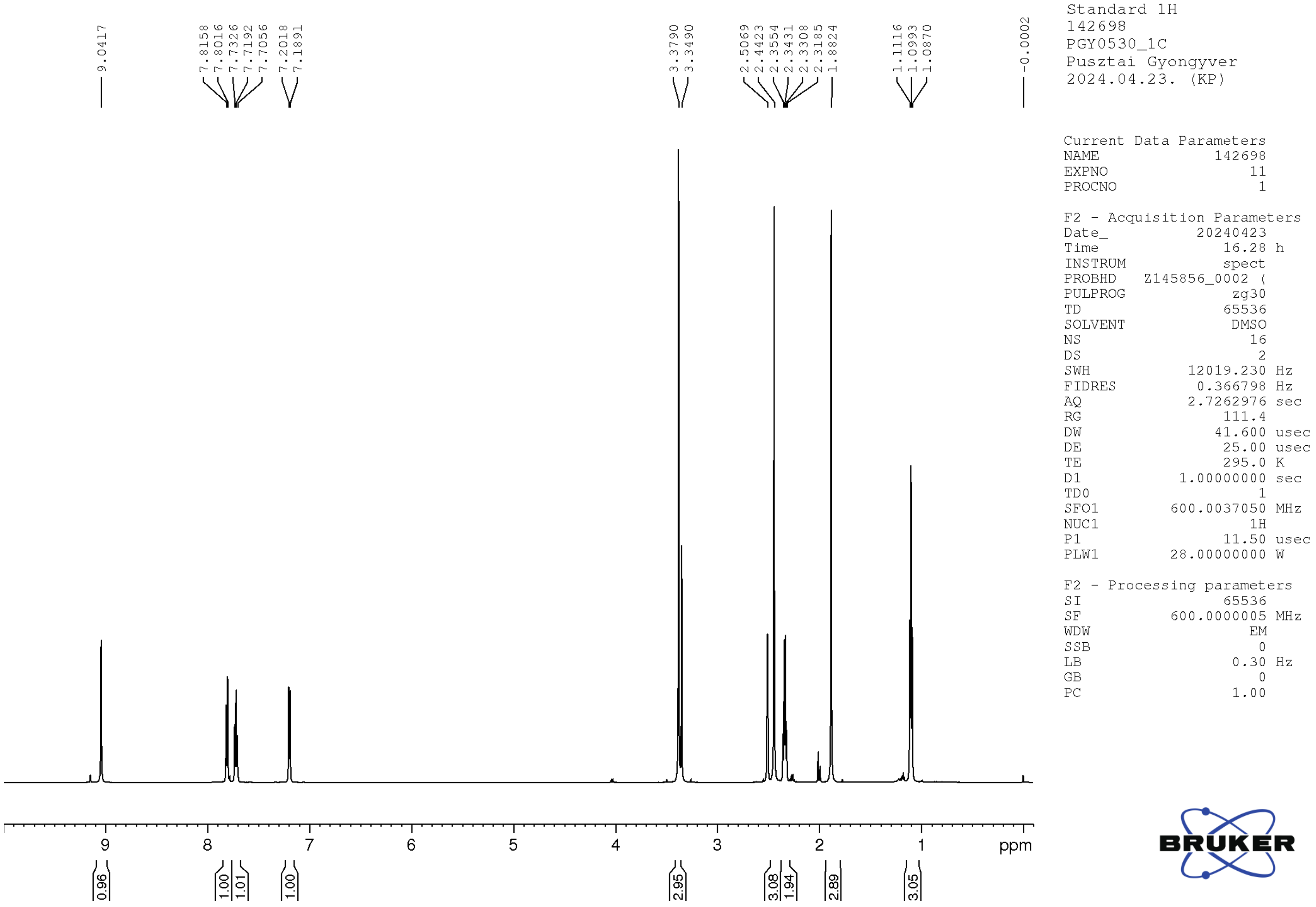

^{13}C NMR spectrum of (*E*)-**7a**

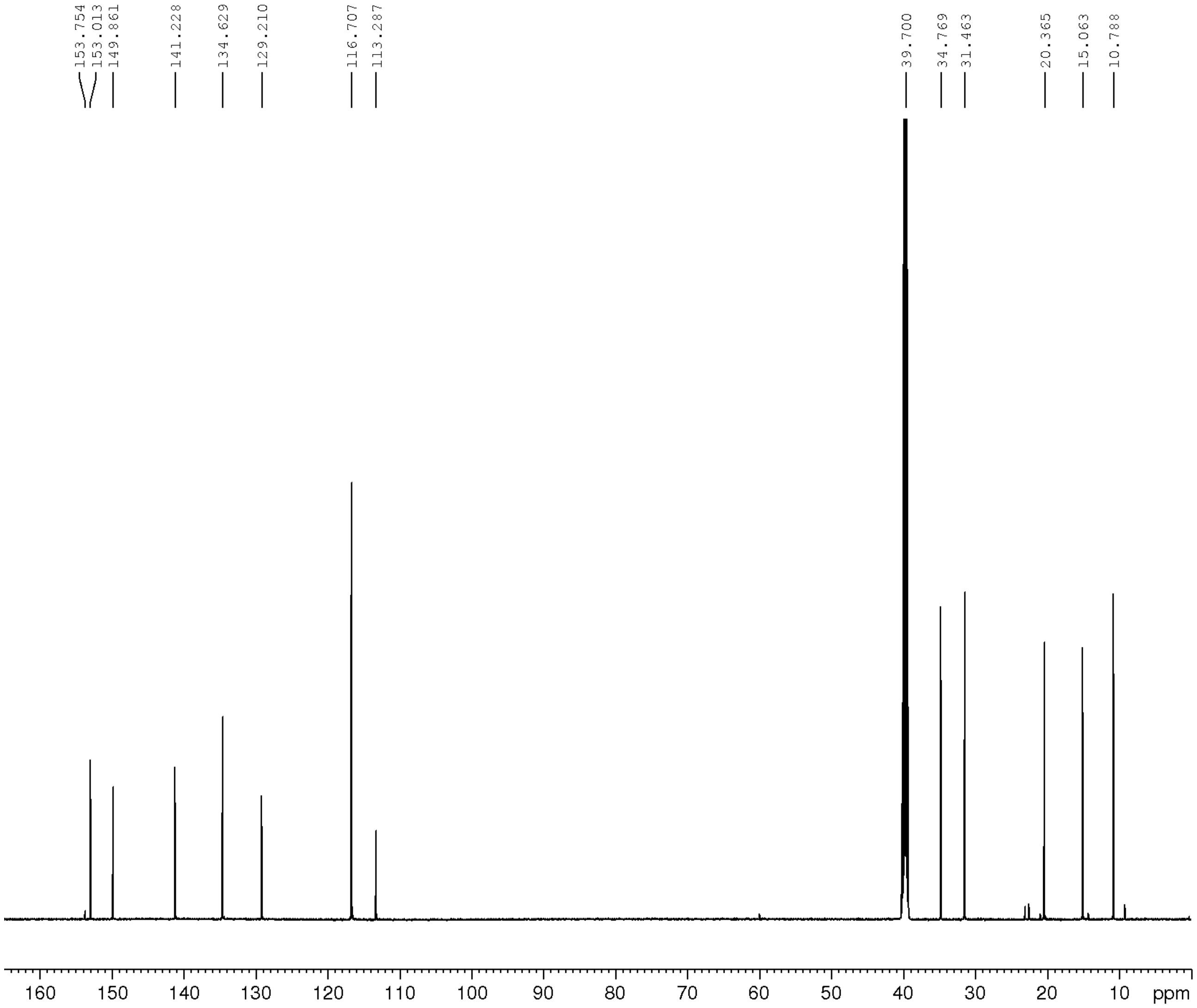

Standard 13C
142698
PGY0530_1C
Pusztai Gyongyver
2024.04.23. (KP)

Current Data Parameters

NAME	142698
EXPNO	12
PROCNO	1

F2 - Acquisition Parameters

Date_	20240423
Time	17.36 h
INSTRUM	spect
PROBHD	Z145856_0002 (
PULPROG	zgpg30
TD	65536
SOLVENT	DMSO
NS	2048
DS	4
SWH	36231.883 Hz
FIDRES	1.105709 Hz
AQ	0.9043968 sec
RG	196.07
DW	13.800 usec
DE	18.00 usec
TE	295.0 K
D1	1.00000000 sec
D11	0.03000000 sec
TD0	1
SFO1	150.8852070 MHz
NUC1	13C
P1	9.90 usec
PLW1	71.00000000 W
SFO2	600.0024000 MHz
NUC2	1H
CPDPRG[2	waltz16
PCPD2	80.00 usec
PLW2	32.90000153 W
PLW12	0.70370001 W
PLW13	0.35339001 W

F2 - Processing parameters

SI	32768
SF	150.8701599 MHz
WDW	EM
SSB	0
LB	1.00 Hz
GB	0
PC	1.40

IR spectrum of (*E*)-**7a**

**142698**	Pusztai Gyongyver	KP	BRUKER Alpha
			Resolution: 2 cm-1
**PGY0530_1C**	KBr	4/23/2024	Number of Scans: 16

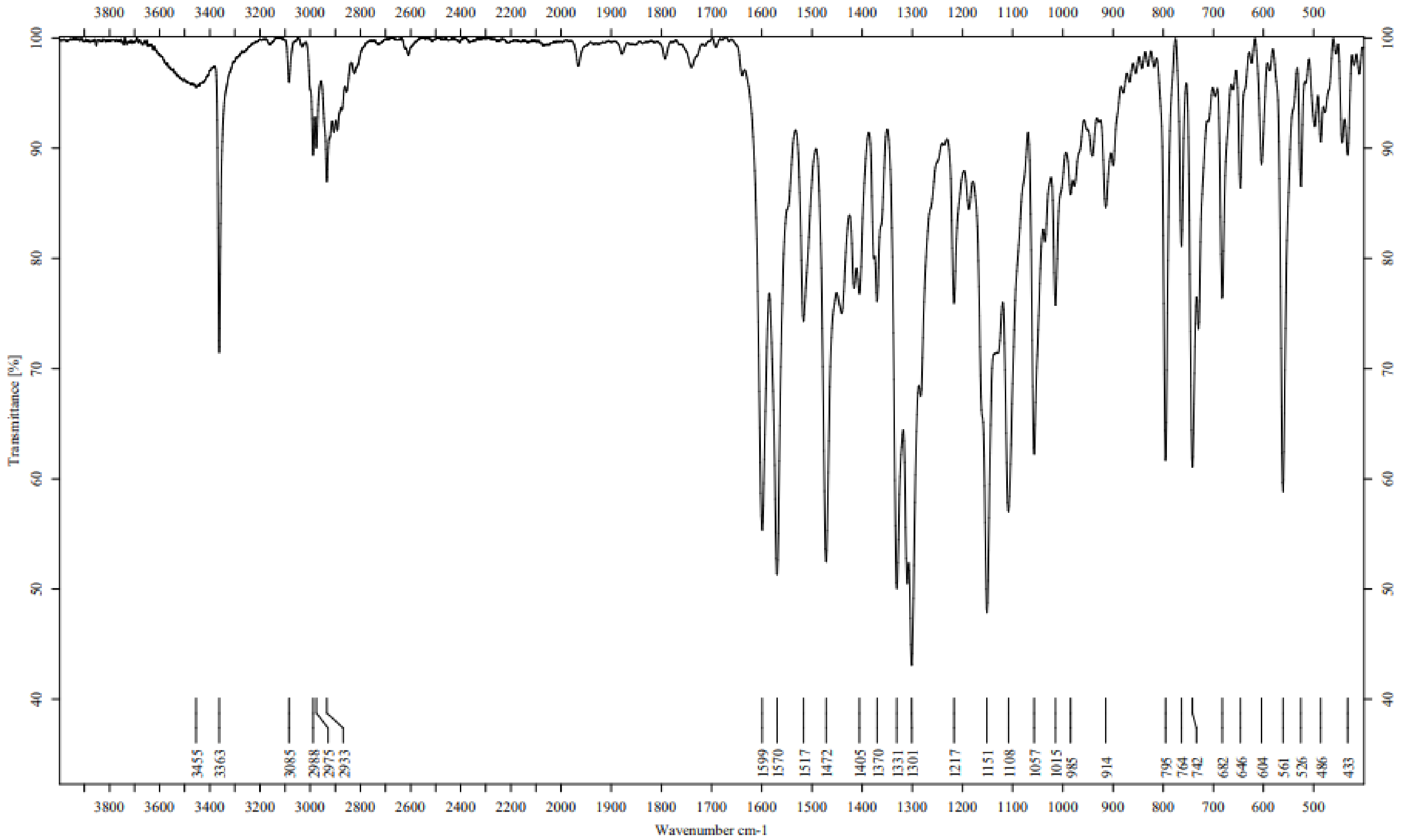

HRMS spectrum of (*E*)-**7a**

Sample: *PGY0530_1C*	*Lab code:* *Nsz - 28996*
Submitter: *Pusztai Gyongyve*	*Project:* *Other*

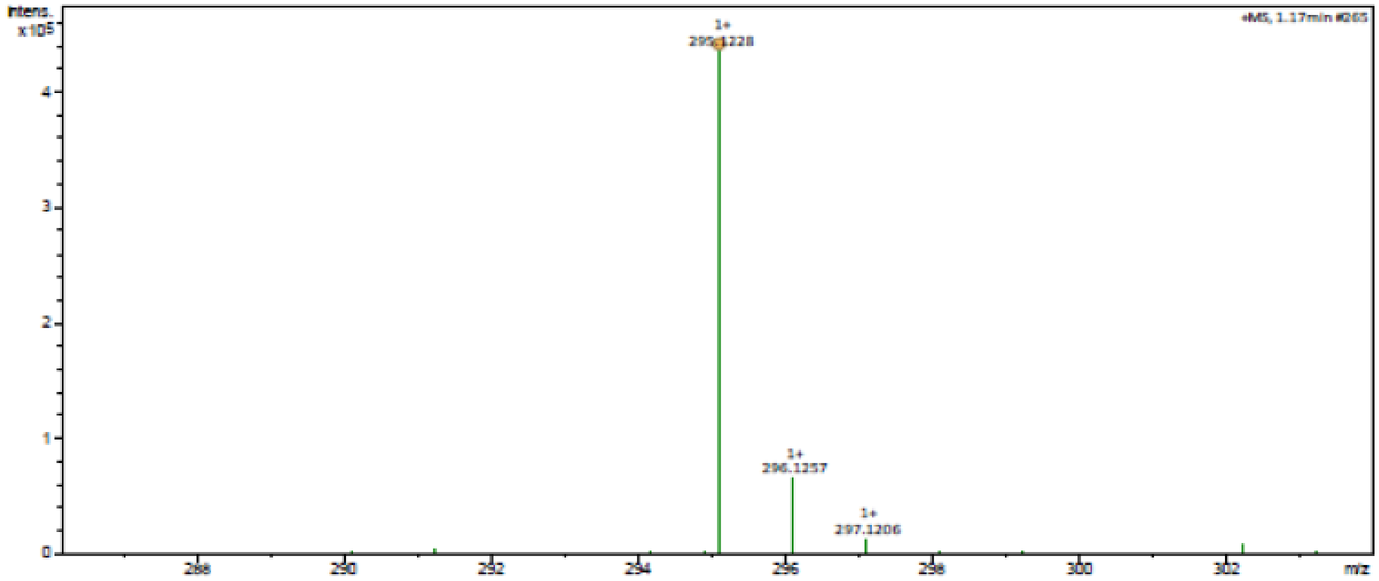

Meas. m/z	Ion Formula	m/z	|err| [ppm]
295.1228	C13H19N4O2S	295.1229	1.5

^{1}H NMR spectrum of **7b**

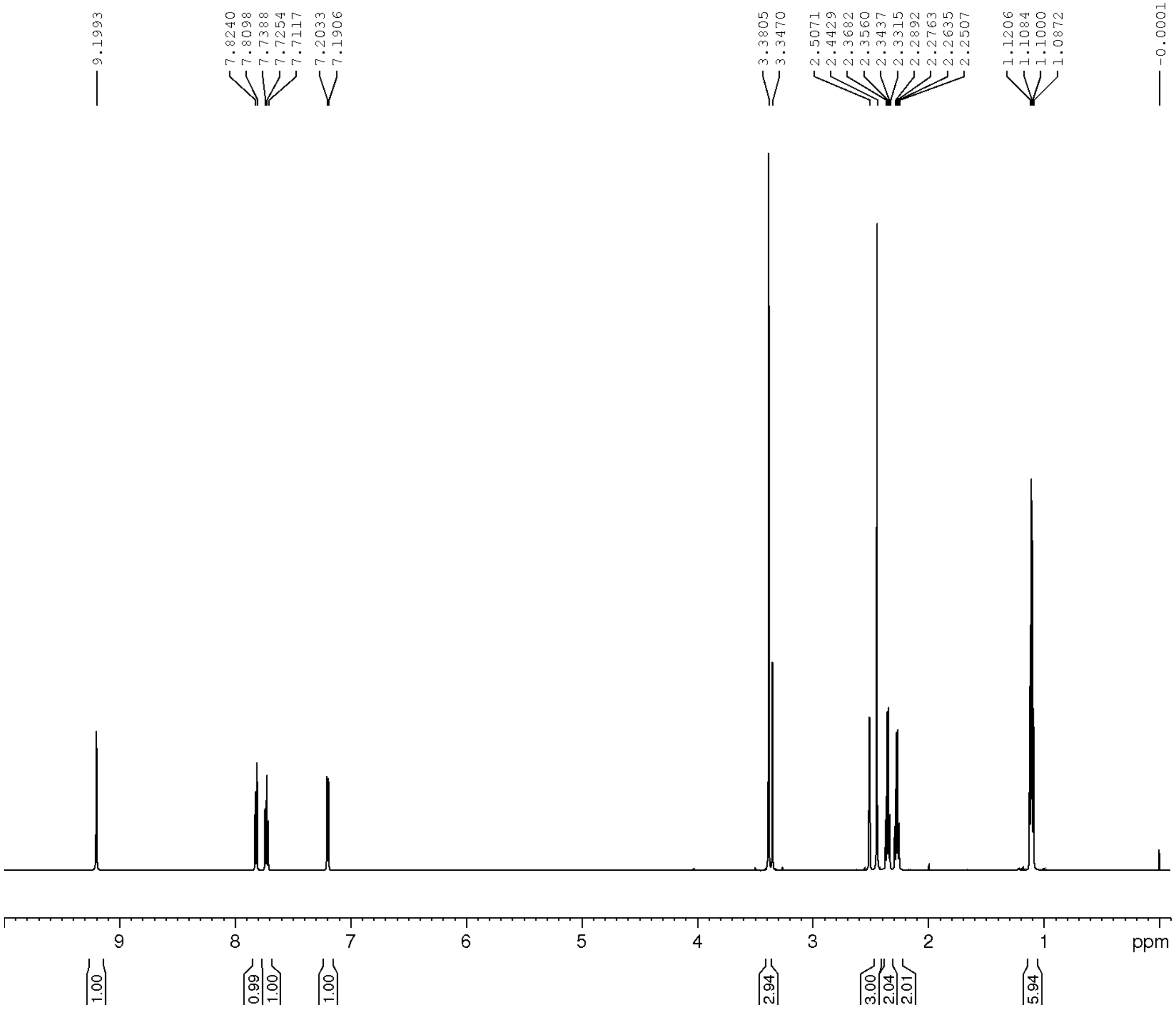

Standard 1H
142657
PGY0538_1A
Pusztai Gyongyver
2024.04.11. (KP)

Current Data Parameters
NAME 142657
EXPNO 11
PROCNO 1

F2 - Acquisition Parameters
Date_ 20240411
Time 21.02 h
INSTRUM spect
PROBHD Z145856_0002 (
PULPROG zg30
TD 65536
SOLVENT DMSO
NS 16
DS 2
SWH 12019.230 Hz
FIDRES 0.366798 Hz
AQ 2.7262976 sec
RG 119.07
DW 41.600 usec
DE 25.00 usec
TE 295.0 K
D1 1.00000000 sec
TD0 1
SFO1 600.0037050 MHz
NUC1 1H
P1 11.50 usec
PLW1 28.00000000 W

F2 - Processing parameters
SI 65536
SF 600.0000004 MHz
WDW EM
SSB 0
LB 0.30 Hz
GB 0
PC 1.00

^{13}C NMR spectrum of **7b**

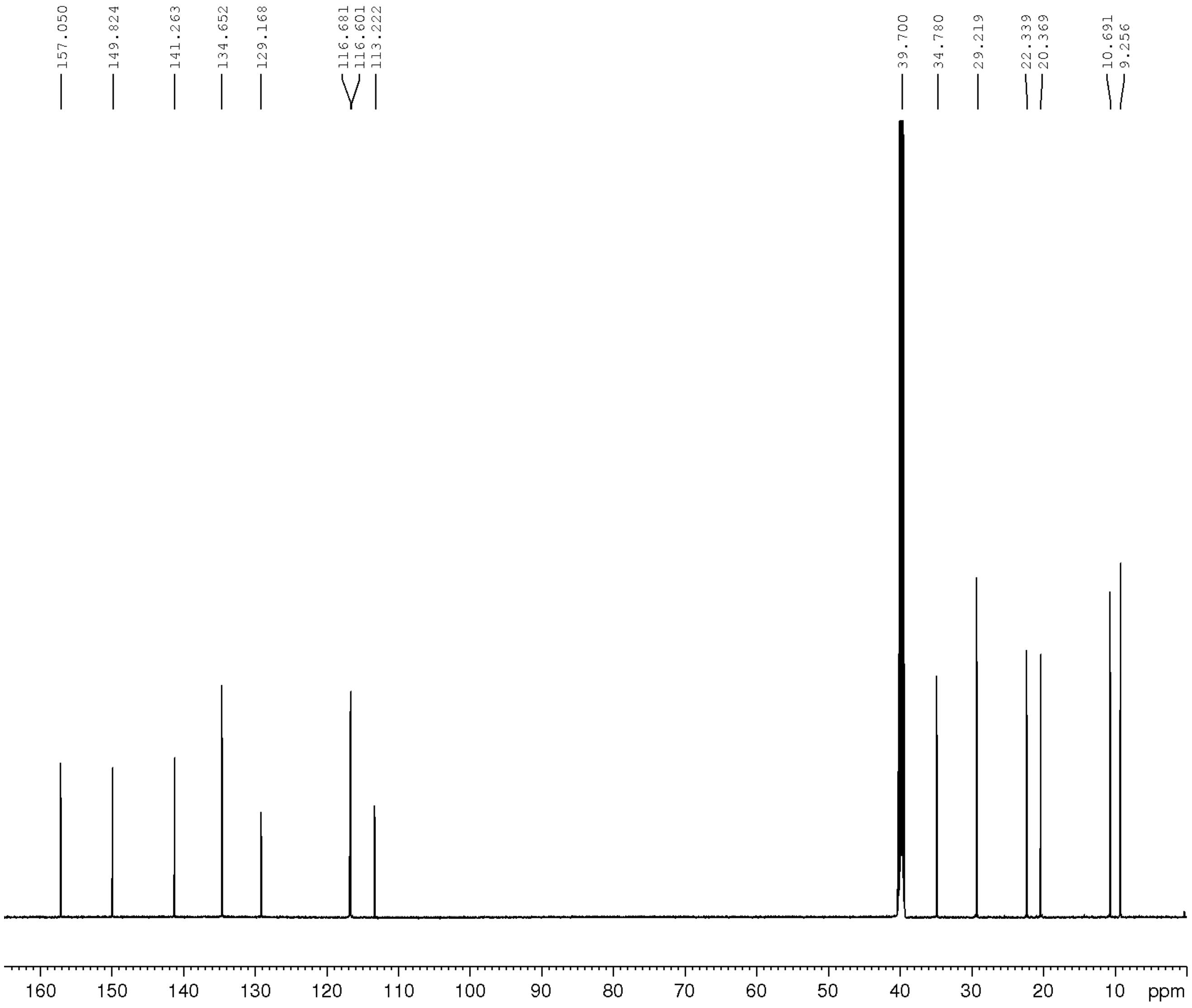

Standard 13C
142657
PGY0538_1A
Pusztai Gyongyver
2024.04.11. (KP)

Current Data Parameters	
NAME	142657
EXPNO	12
PROCNO	1

F2 - Acquisition Parameters	
Date_	20240411
Time	22.12 h
INSTRUM	spect
PROBHD	Z145856_0002 (
PULPROG	zgpg30
TD	65536
SOLVENT	DMSO
NS	2048
DS	4
SWH	36231.883 Hz
FIDRES	1.105709 Hz
AQ	0.9043968 sec
RG	196.07
DW	13.800 usec
DE	18.00 usec
TE	295.0 K
D1	1.00000000 sec
D11	0.03000000 sec
TD0	1
SFO1	150.8852070 MHz
NUC1	13C
P1	9.90 usec
PLW1	71.00000000 W
SFO2	600.0024000 MHz
NUC2	1H
CPDPRG[2	waltz16
PCPD2	80.00 usec
PLW2	32.90000153 W
PLW12	0.70370001 W
PLW13	0.35339001 W

F2 - Processing parameters	
SI	32768
SF	150.8701601 MHz
WDW	EM
SSB	0
LB	1.00 Hz
GB	0
PC	1.40

IR spectrum of **7b**

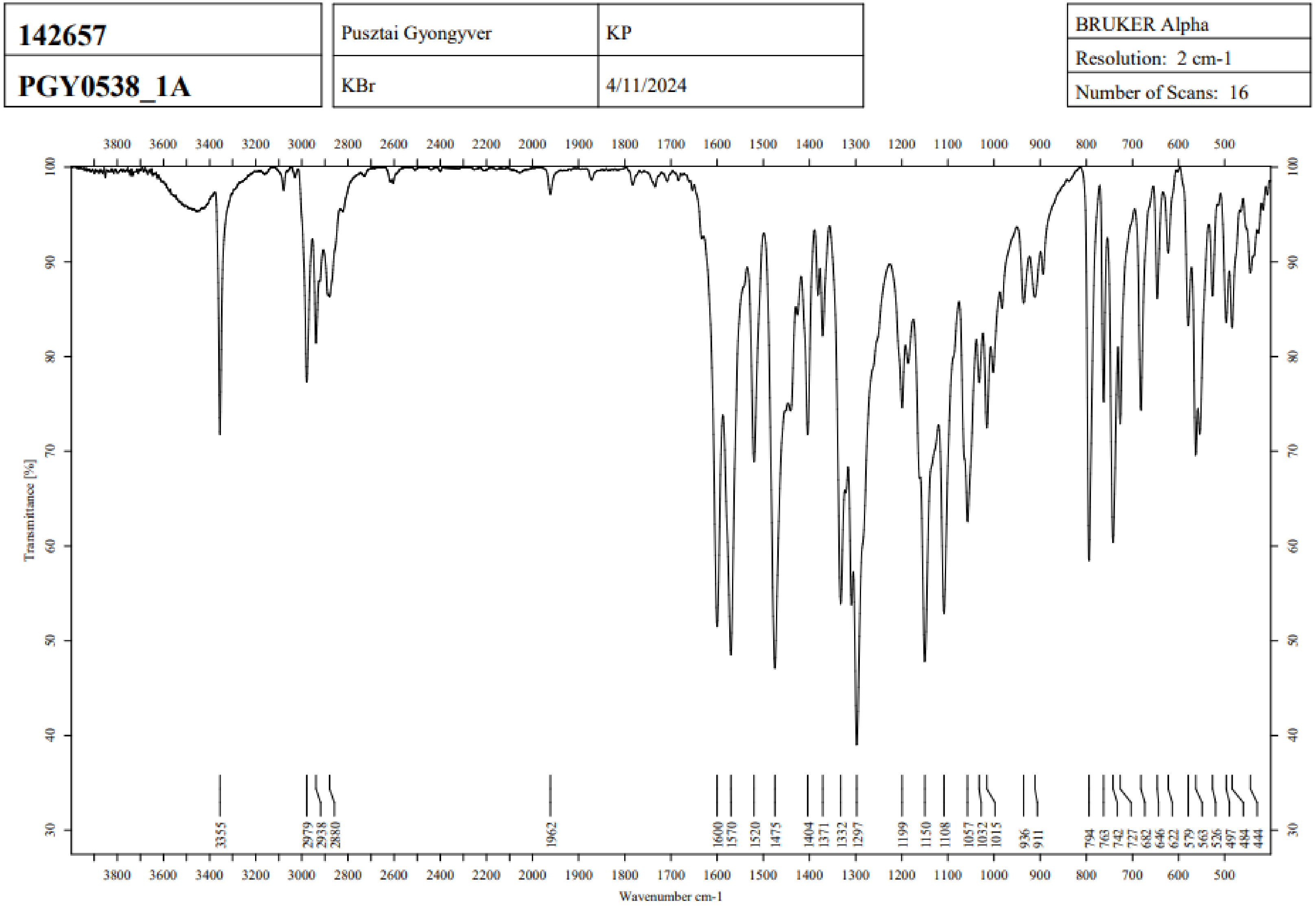

HRMS spectrum of **7b**

Spectrum Plot Report

Name	PGY0538_1A, Pusztai Gyongyver	Rack Pos.		Instrument	7250A with DIP	Operator	MM
Inj. Vol. (ul)	0.5	Plate Pos.		IRM Status	Success		
Data File	142657.D	Method (Acq)	DIP_70eV.M	Comment		Acq. Time (Local)	4/16/2024 2:01:53 PM (UTC+02:00)

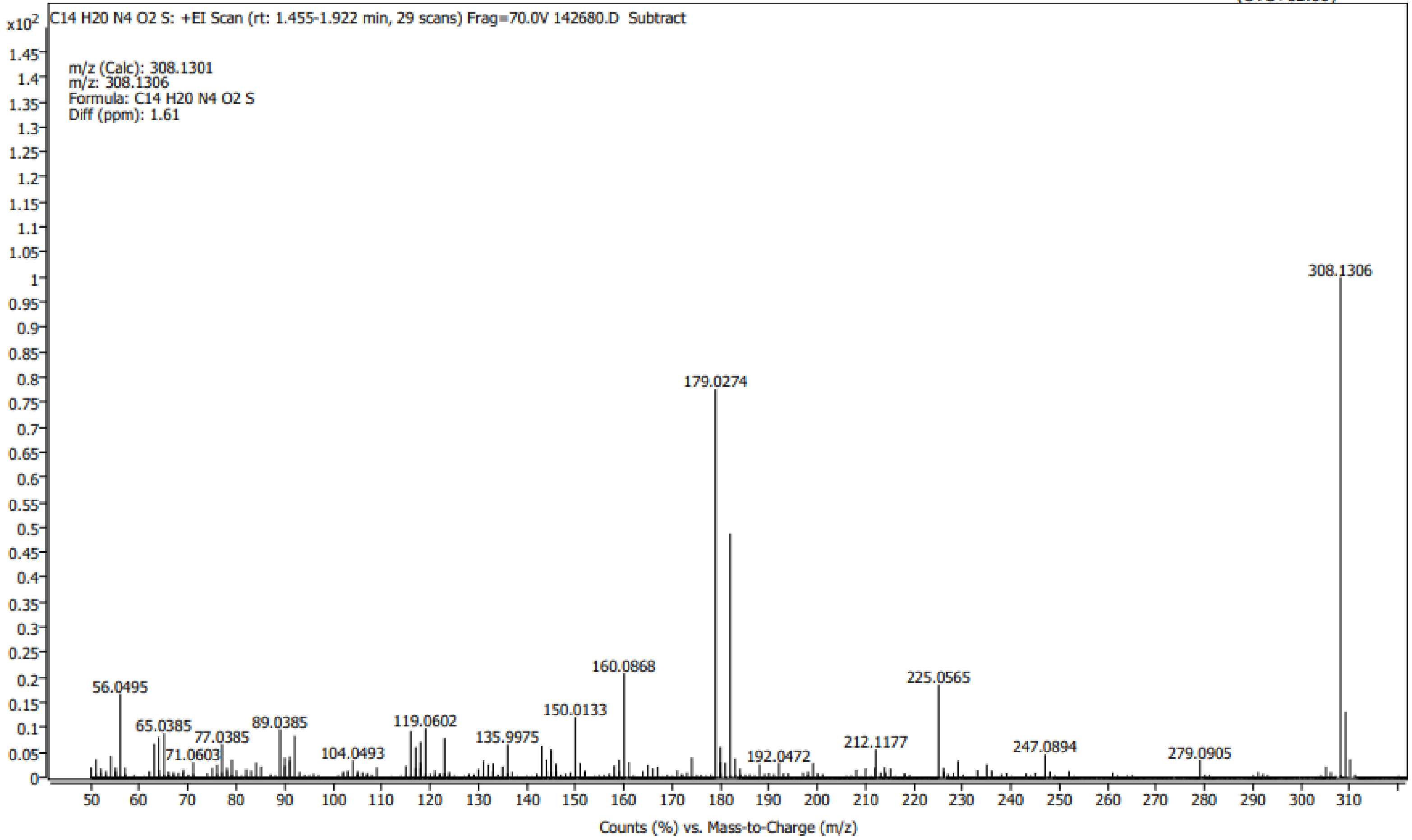

^{1}H NMR spectrum of (*E*)-**7c**

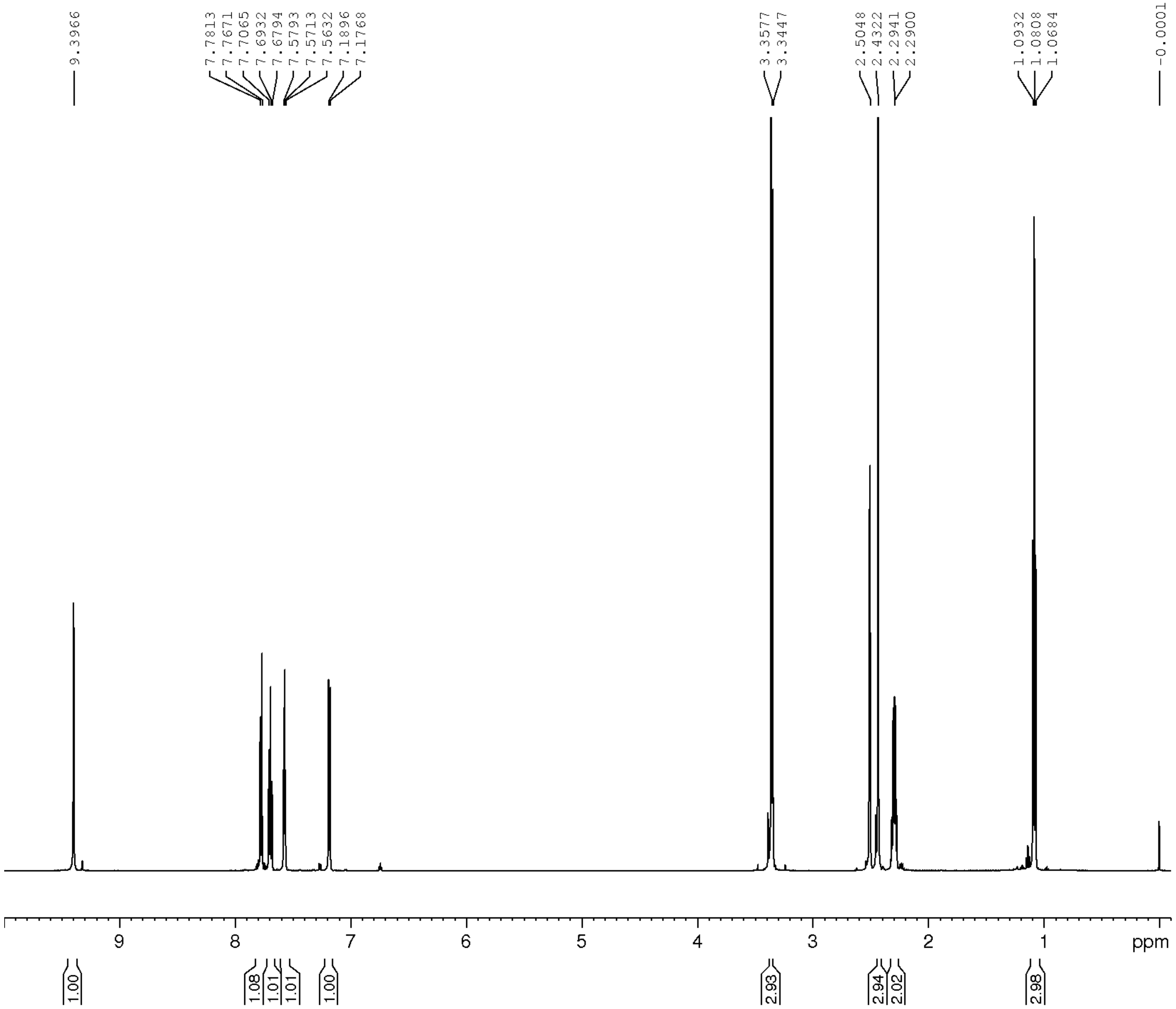

Standard 1H
143707
PGY0790_1A
Pusztai Gyongyver
2024.10.24. (KP)

Current Data Parameters
NAME 143707
EXPNO 21
PROCNO 1

F2 - Acquisition Parameters
Date_ 20241024
Time 15.44 h
INSTRUM spect
PROBHD Z145856_0002 (
PULPROG zg30
TD 65536
SOLVENT DMSO
NS 16
DS 2
SWH 12019.230 Hz
FIDRES 0.366798 Hz
AQ 2.7262976 sec
RG 196.07
DW 41.600 usec
DE 25.00 usec
TE 295.0 K
D1 1.00000000 sec
TD0 1
SFO1 600.0037050 MHz
NUC1 1H
P1 11.50 usec
PLW1 28.00000000 W

F2 - Processing parameters
SI 65536
SF 600.0000018 MHz
WDW EM
SSB 0
LB 0.30 Hz
GB 0
PC 1.00

^{13}C NMR spectrum of (*E*)-**7c**

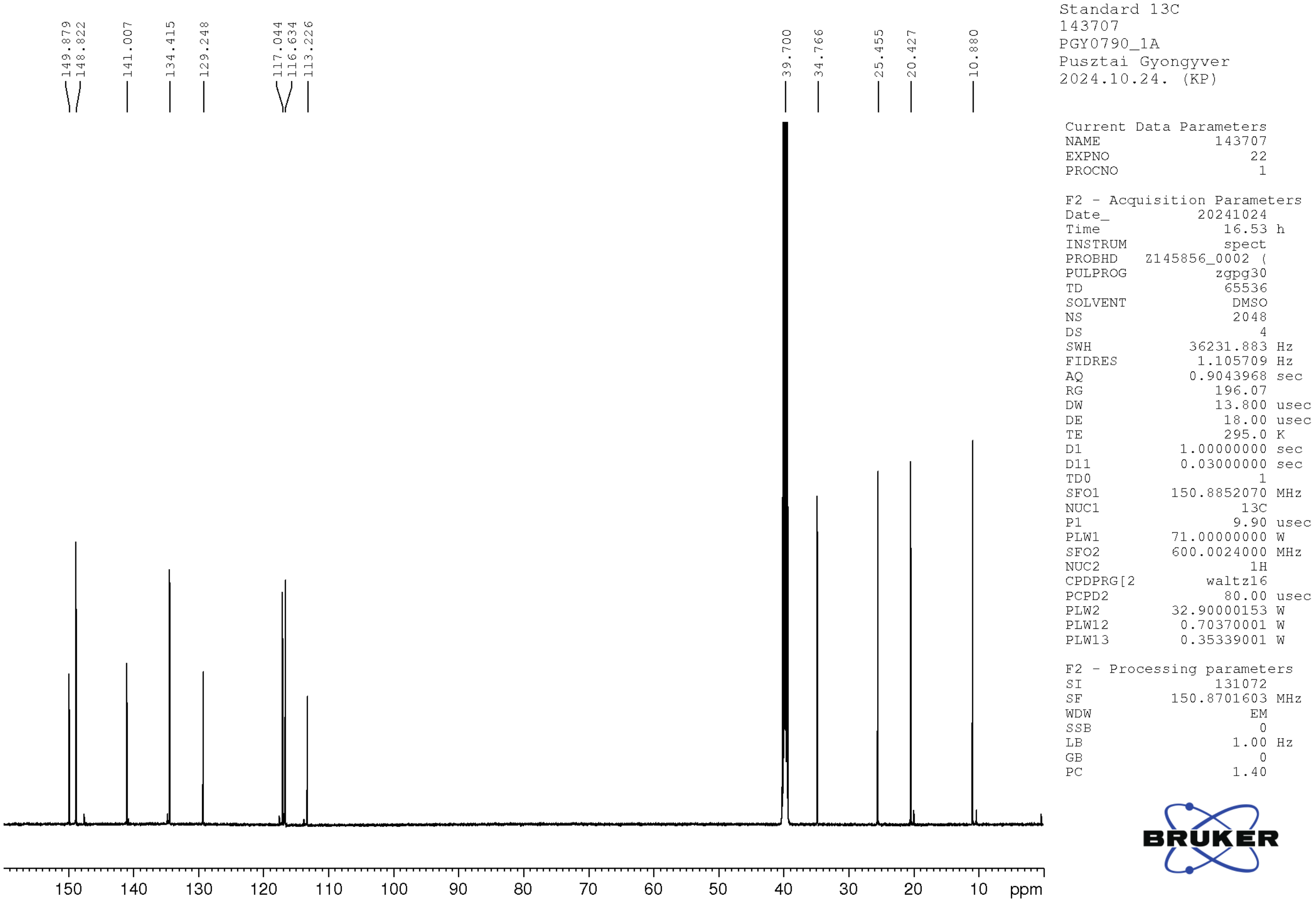

IR spectrum of (*E*)-**7c**

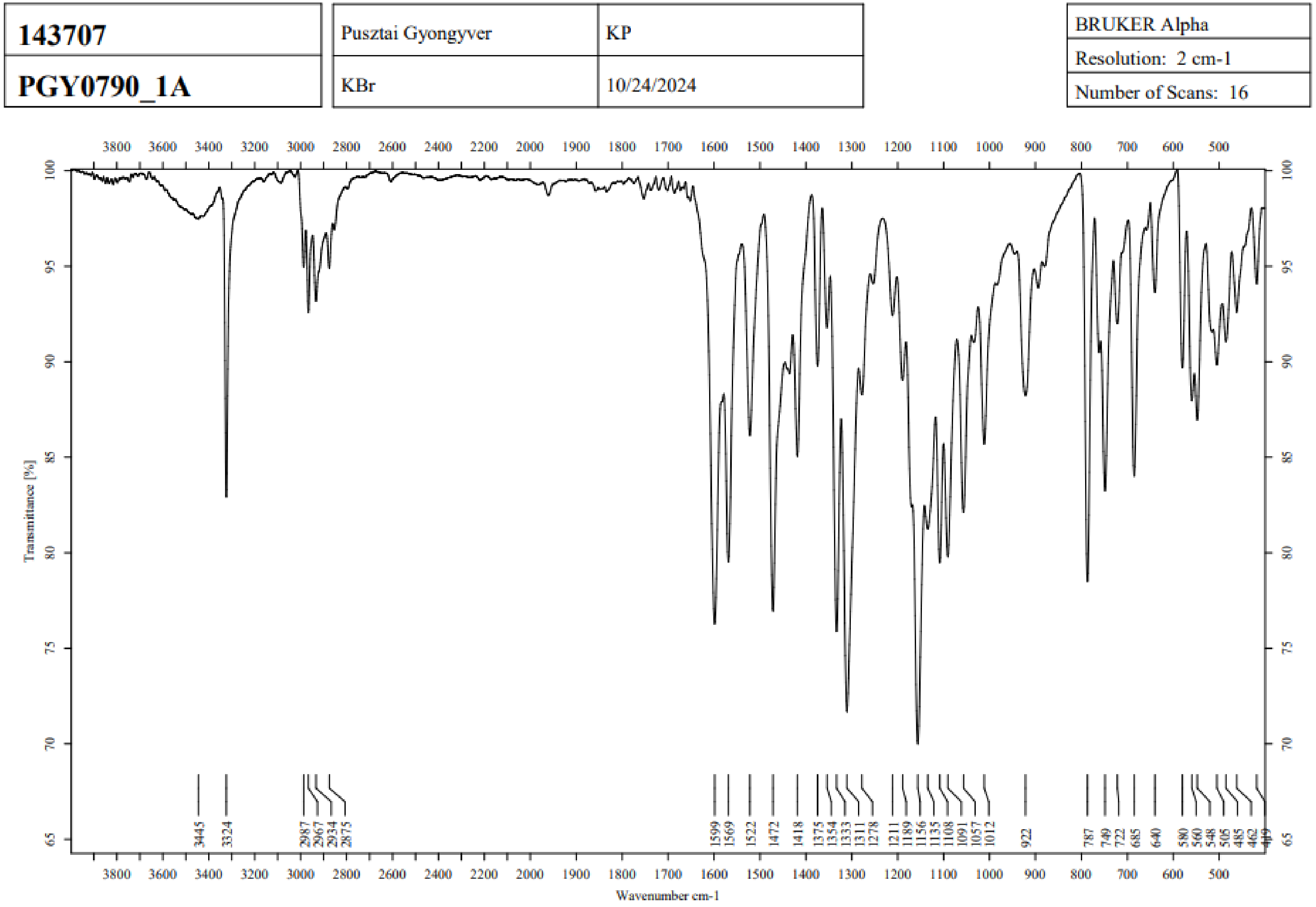

HRMS spectrum of (*E*)-**7c**

Spectrum Plot Report

Name	PGZ0790_1A, Pusztai Gyongyver	Rack Pos.		Instrument	7250A with DIP	Operator	MM
Inj. Vol. (ul)	1	Plate Pos.		IRM Status	Success		
Data File	143707qtof_dip.D	Method (Acq)	DIP_70eV_4min.M	Comment		Acq. Time (Local)	11/21/2024 5:33:16 PM (UTC+01:00)

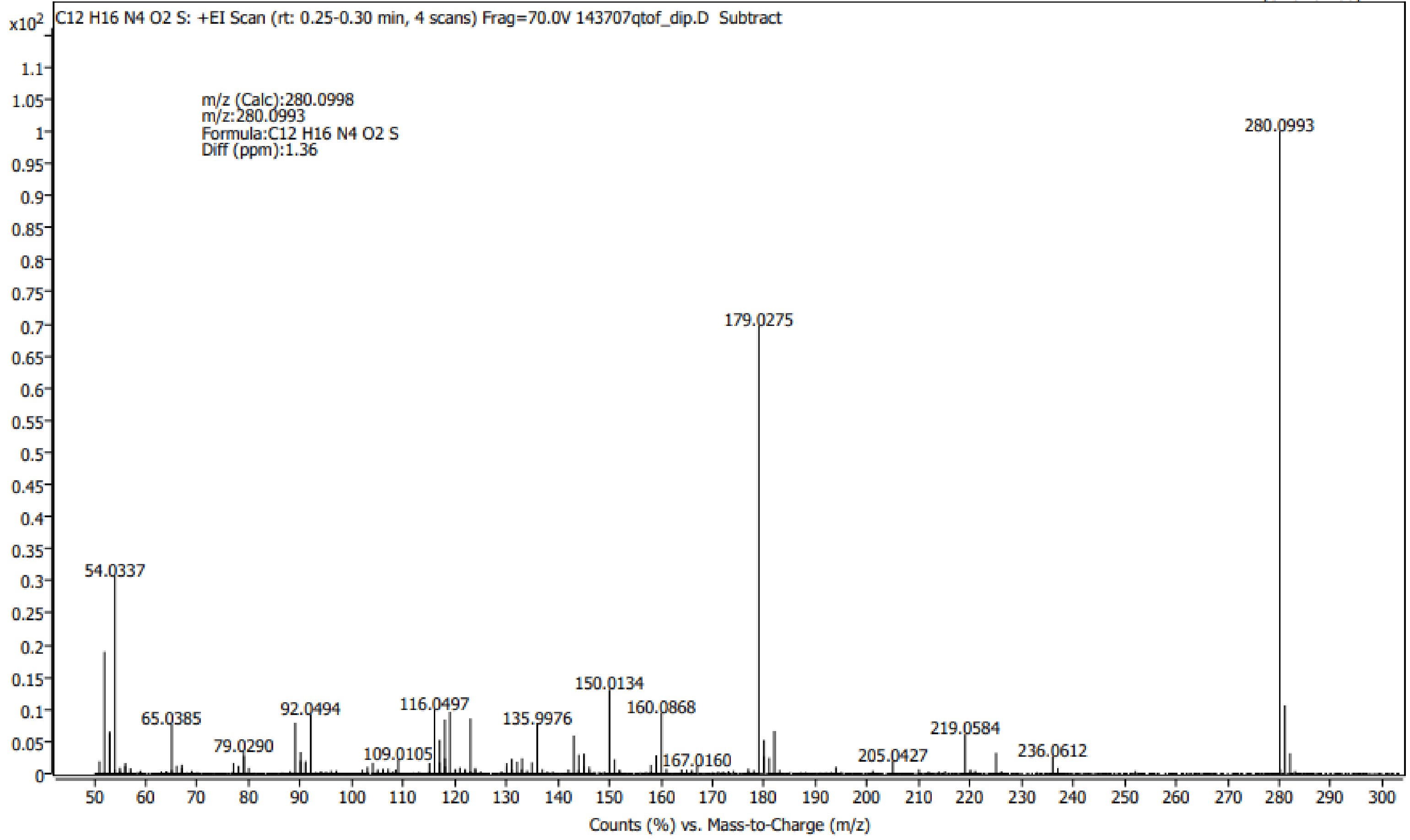

^{1}H NMR spectrum of (*Z*)-**7c**

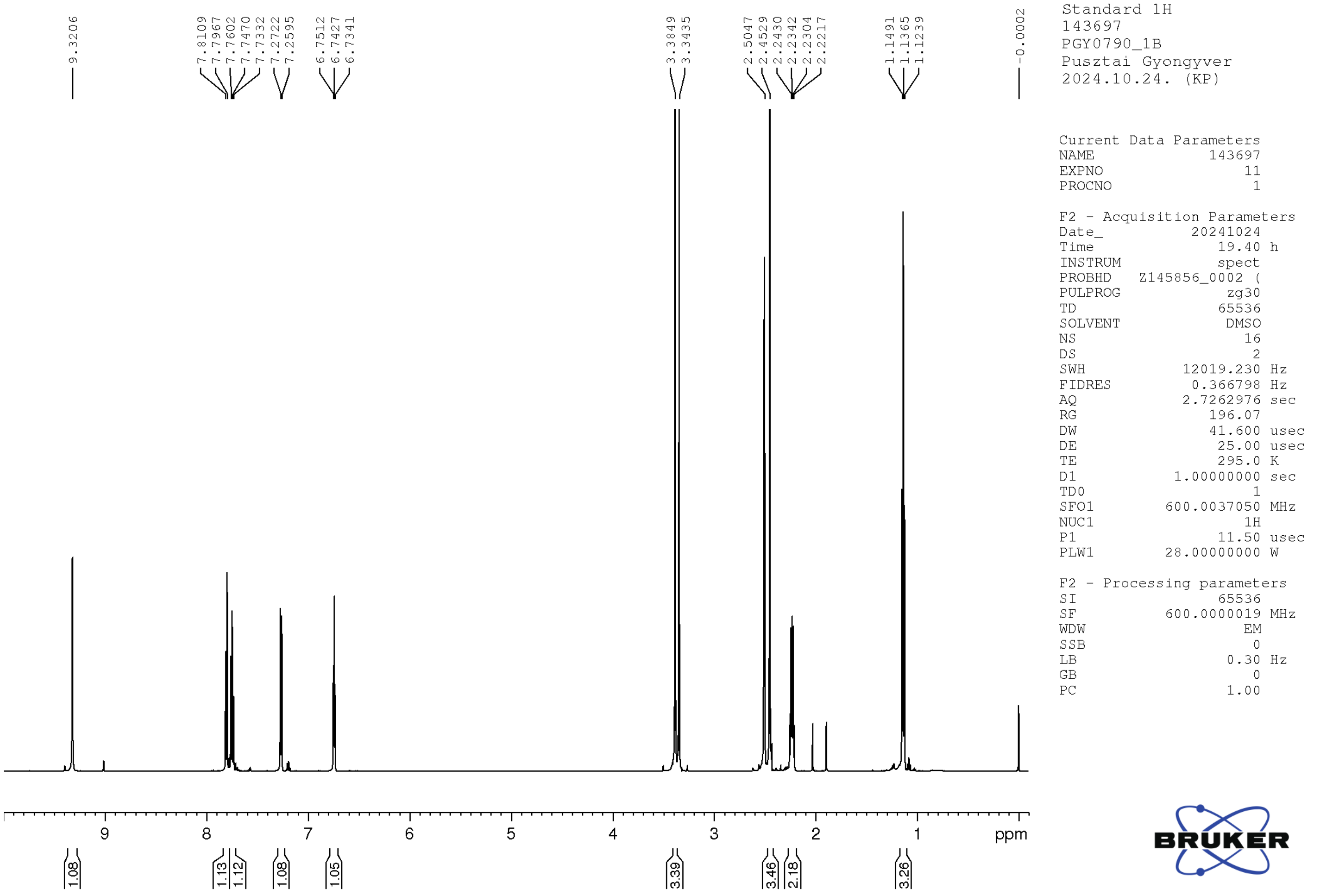

^{13}C NMR spectrum of (*Z*)-**7c**

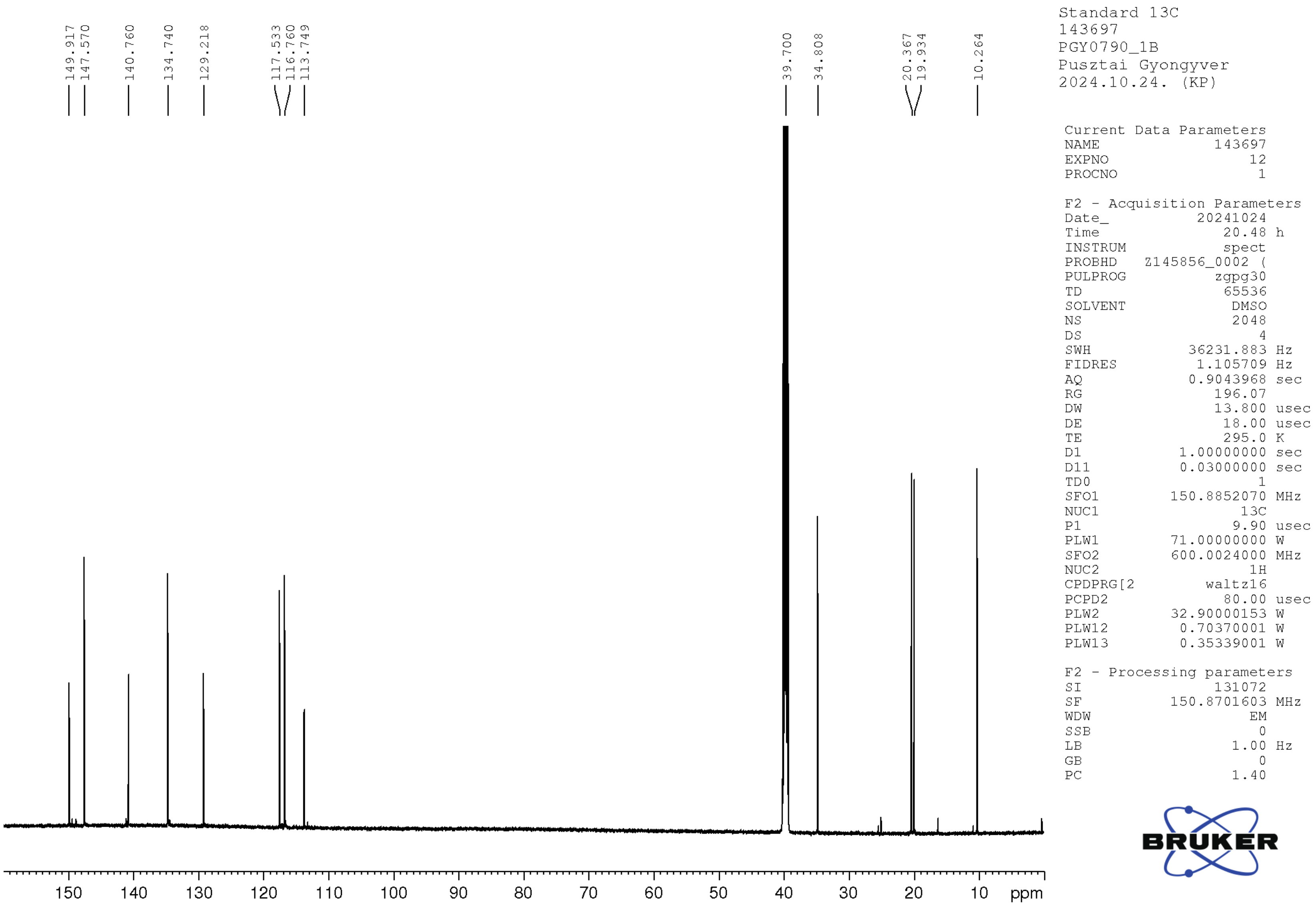

IR spectrum of (*Z*)-**7c**

**143697**	
**PGY0790_1B**	

Pusztai Gyongyver	KP
KBr	10/24/2024

BRUKER Alpha
Resolution: 2 cm-1
Number of Scans: 16

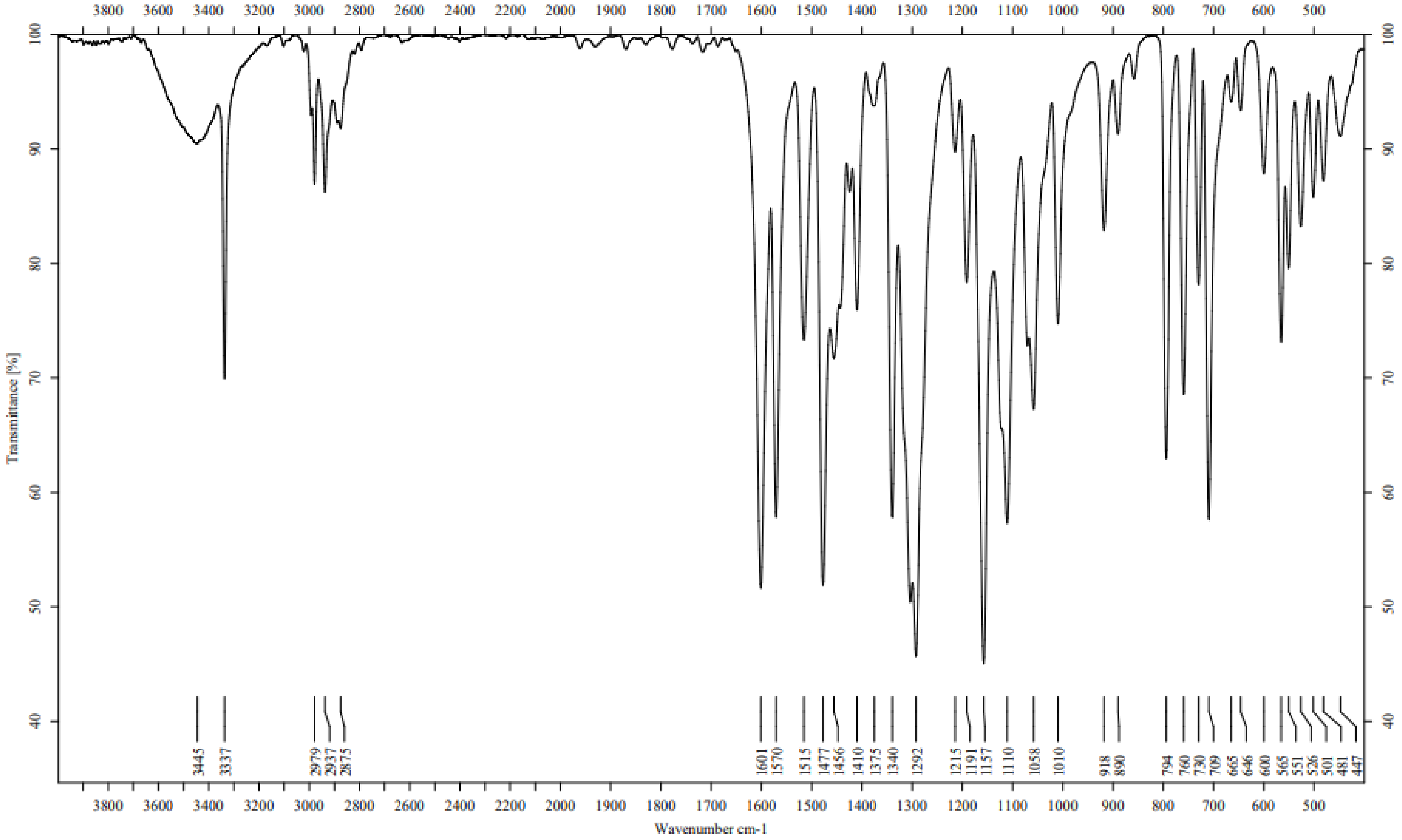

HRMS spectrum of (*Z*)-**7c**

Spectrum Plot Report

Name	PGZ0790_1B, Pusztai Gyongyver	Rack Pos.		Instrument	7250A with DIP	Operator	MM
Inj. Vol. (ul)	1	Plate Pos.		IRM Status	Success		
Data File	143697qtof_dip.D	Method (Acq)	DIP_70eV_4min.M	Comment		Acq. Time (Local)	11/21/2024 5:41:17 PM (UTC+01:00)

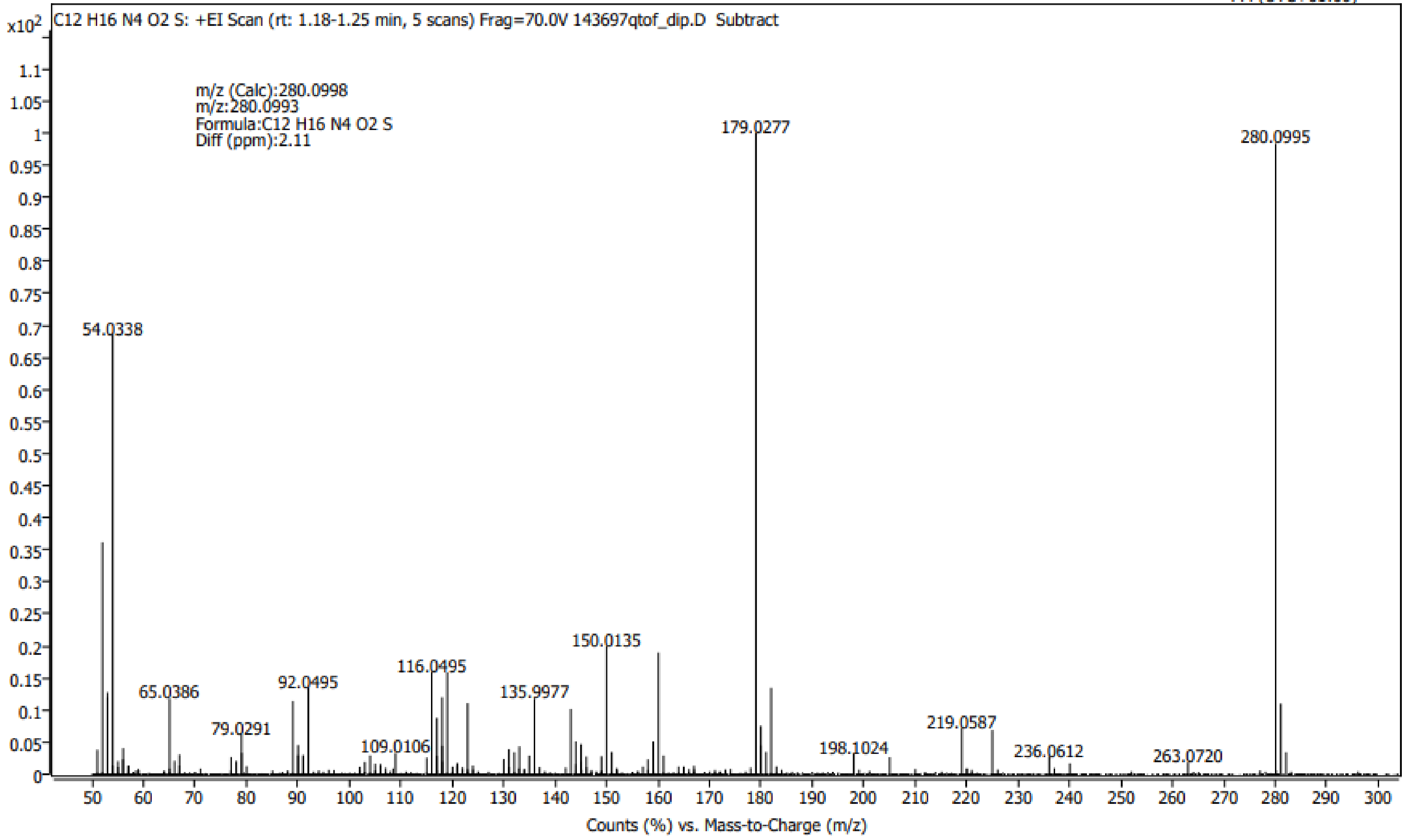

^{1}H NMR spectrum of **7d**

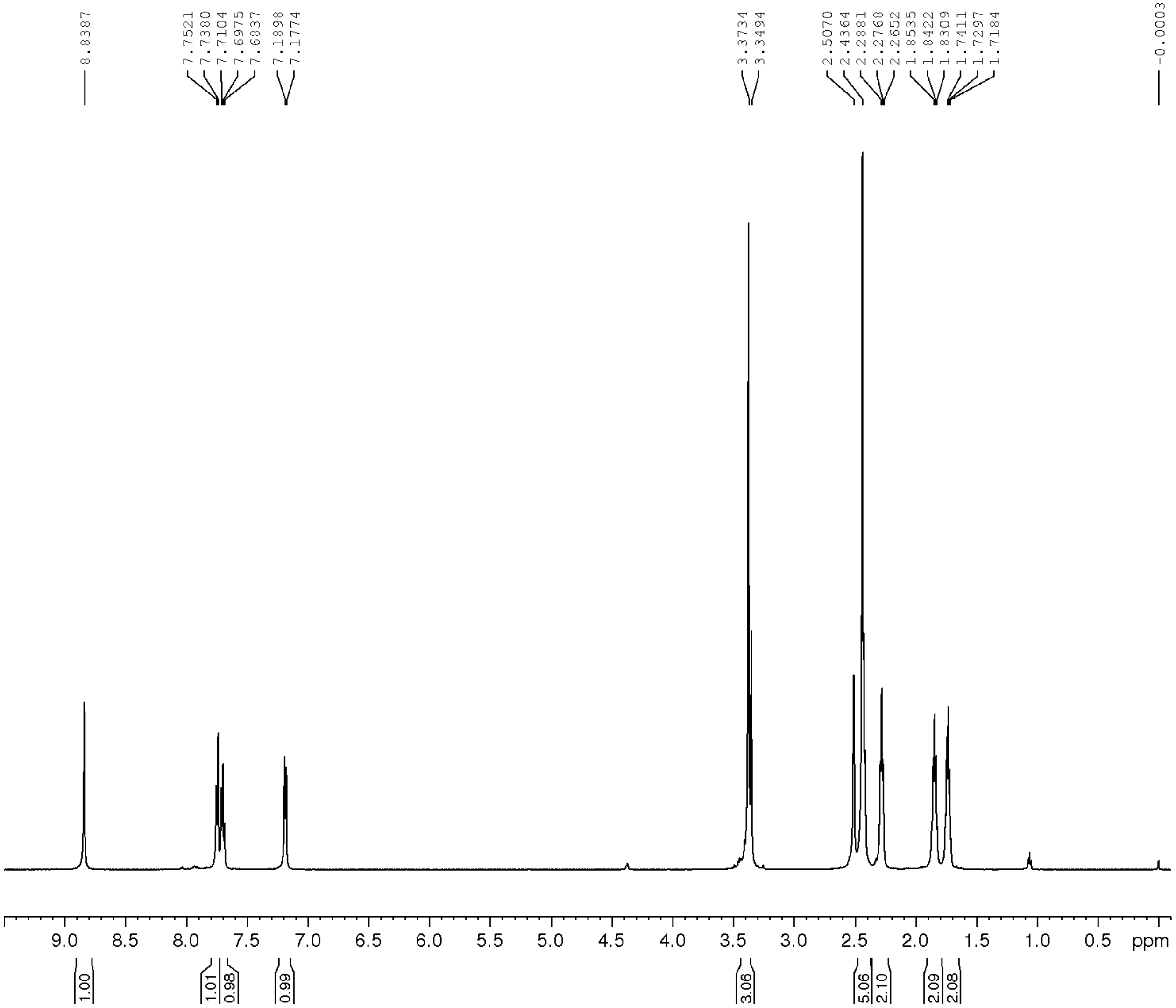

Standard 1H
143739
PGY0808_1
Pusztai Gyongyver
2024.10.21. (DA)

Current Data Parameters

NAME	143739
EXPNO	11
PROCNO	1

F2 - Acquisition Parameters

Date_	20241021
Time	17.52 h
INSTRUM	spect
PROBHD	Z145856_0002 (
PULPROG	zg30
TD	65536
SOLVENT	DMSO
NS	16
DS	2
SWH	12019.230 Hz
FIDRES	0.366798 Hz
AQ	2.7262976 sec
RG	111.4
DW	41.600 usec
DE	25.00 usec
TE	295.0 K
D1	1.00000000 sec
TD0	1
SFO1	600.0037050 MHz
NUC1	1H
P1	11.50 usec
PLW1	28.00000000 W

F2 - Processing parameters

SI	65536
SF	600.0000012 MHz
WDW	no
SSB	0
LB	0 Hz
GB	0
PC	1.00

^{13}C NMR spectrum of **7d**

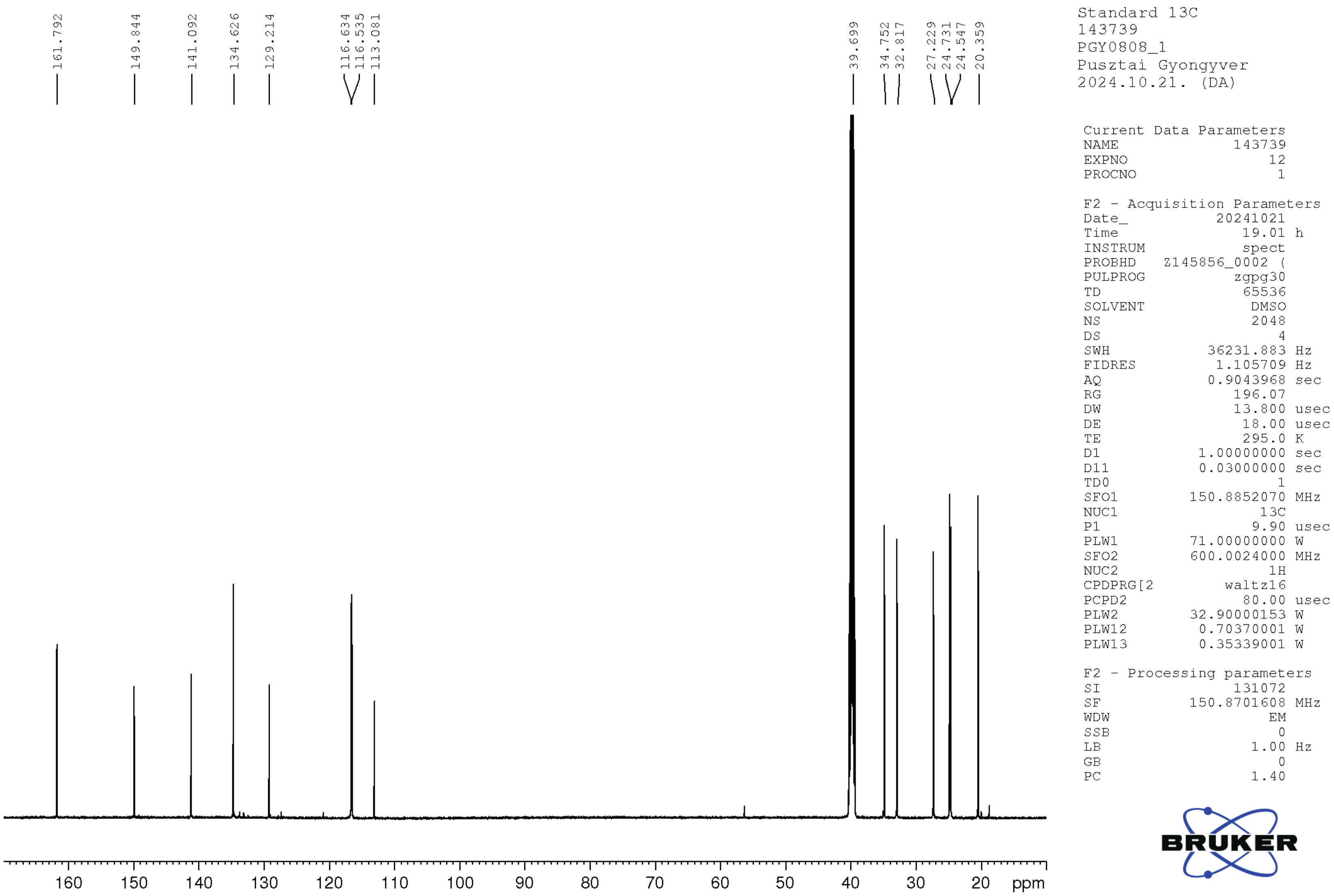

IR spectrum of **7d**

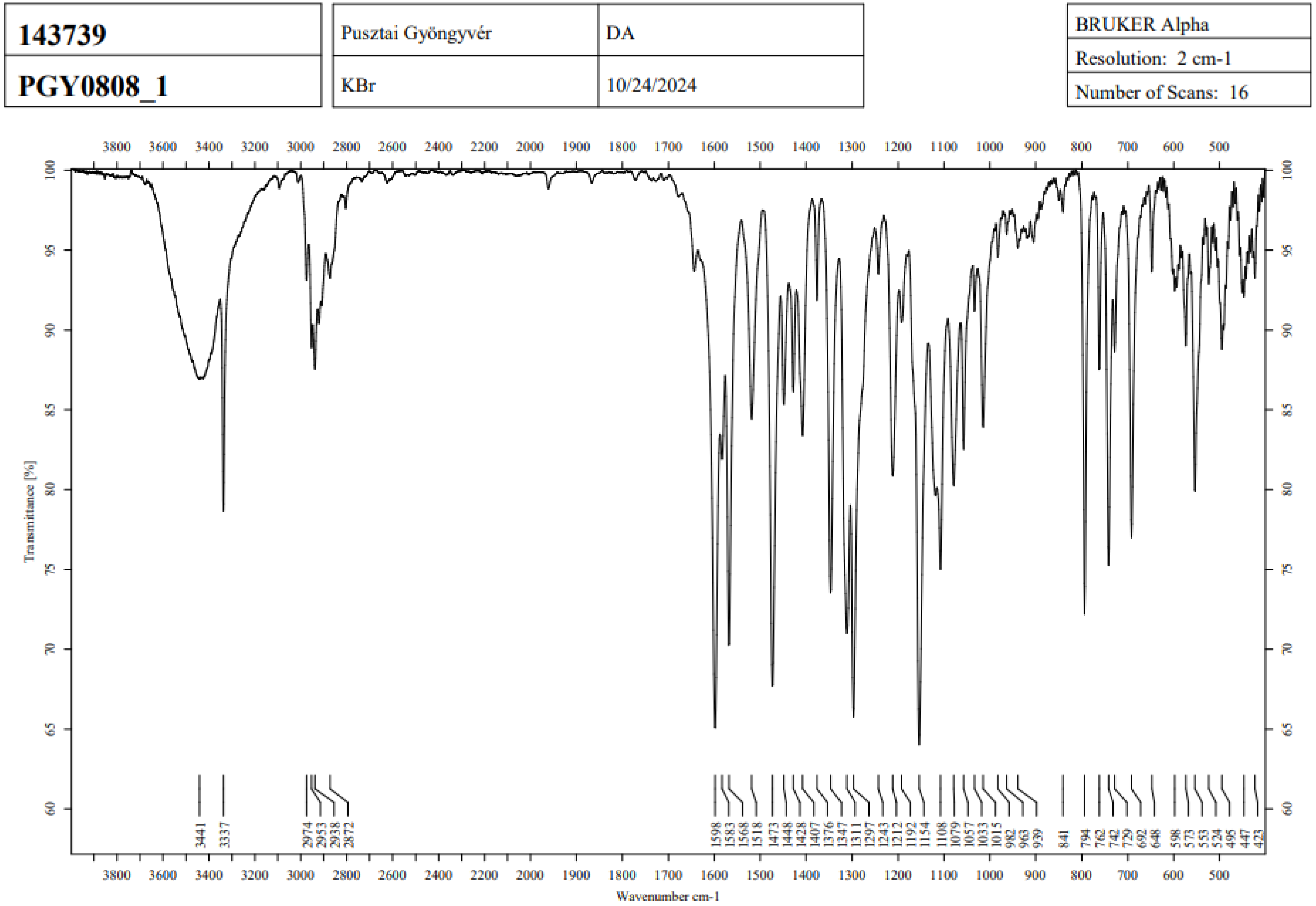

HRMS spectrum of **7d**

Spectrum Plot Report

Name	PGY00808_1, Pusztai Gyongyver	Rack Pos.		Instrument	7250A with DIP	Operator	MM
Inj. Vol. (ul)	0.5	Plate Pos.		IRM Status	Success		
Data File	143739msaqtof_dip2.D	Method (Acq)	DIP_70eV_4min.M	Comment		Acq. Time (Local)	10/24/2024 1:41:44 PM (UTC+02:00)

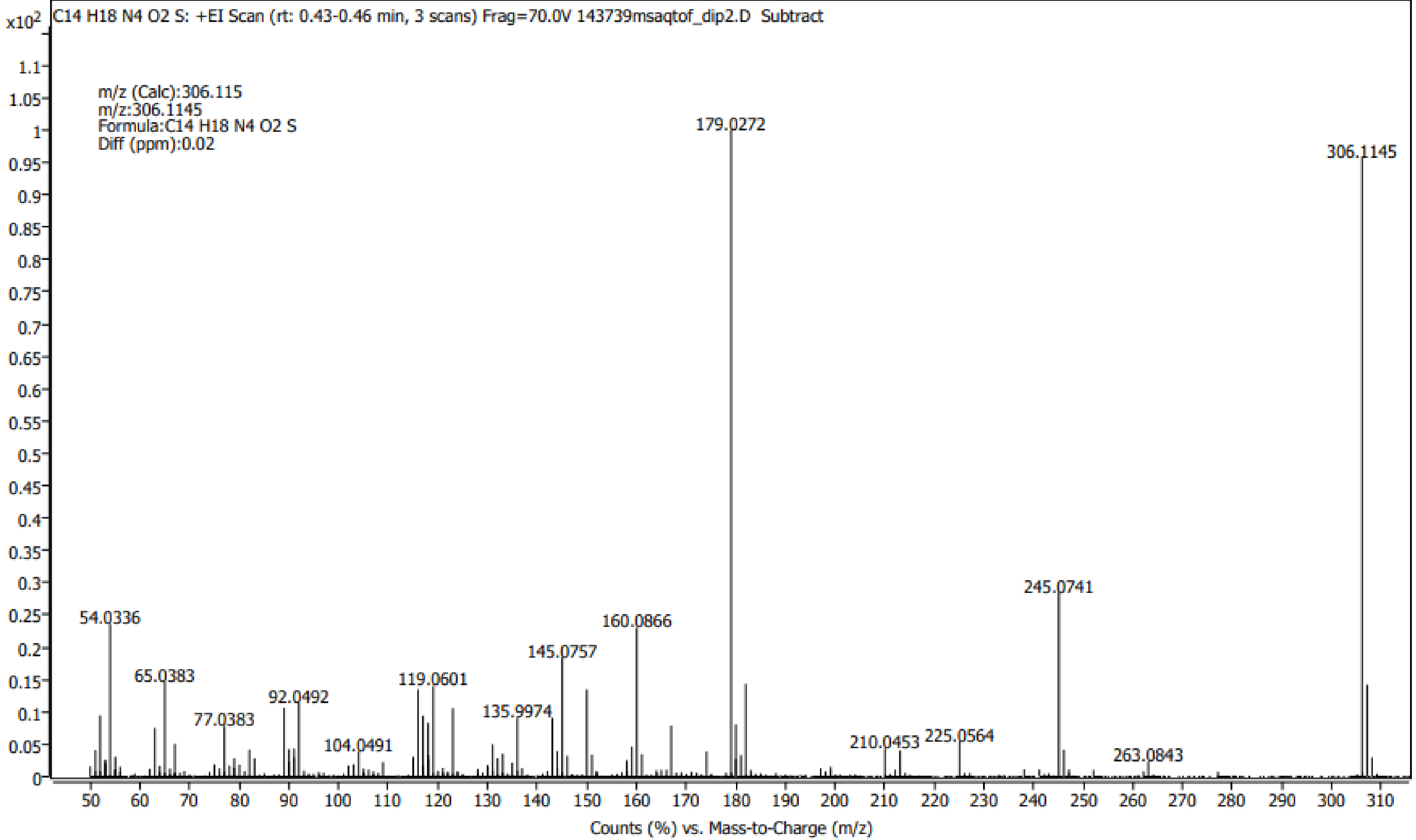

^{1}H NMR spectrum of (*E*)-**9a**

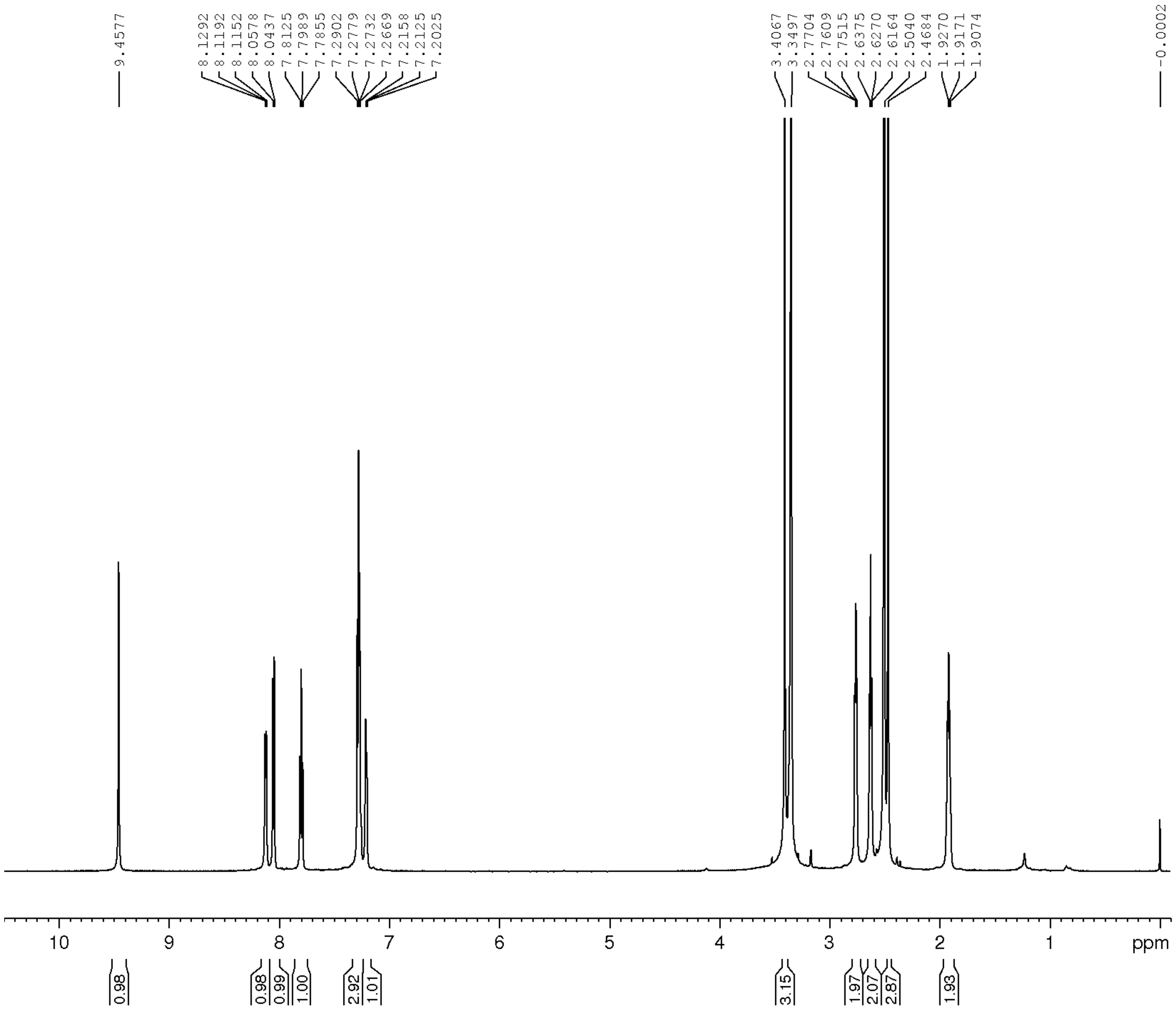

Standard 1H
143519
PGY0782_1
Pusztai Gyongyver
2024.09.17. (DA)

Current Data Parameters
NAME 143519
EXPNO 21
PROCNO 1

F2 - Acquisition Parameters
Date_ 20240918
Time 10.56 h
INSTRUM spect
PROBHD Z145856_0002 (
PULPROG zg30
TD 65536
SOLVENT DMSO
NS 16
DS 2
SWH 12019.230 Hz
FIDRES 0.366798 Hz
AQ 2.7262976 sec
RG 196.07
DW 41.600 usec
DE 25.00 usec
TE 295.0 K
D1 1.00000000 sec
TD0 1
SFO1 600.0037050 MHz
NUC1 1H
P1 11.50 usec
PLW1 28.00000000 W

F2 - Processing parameters
SI 65536
SF 600.0000025 MHz
WDW EM
SSB 0
LB 0.30 Hz
GB 0
PC 1.00

^{13}C NMR spectrum of (*E*)-**9a**

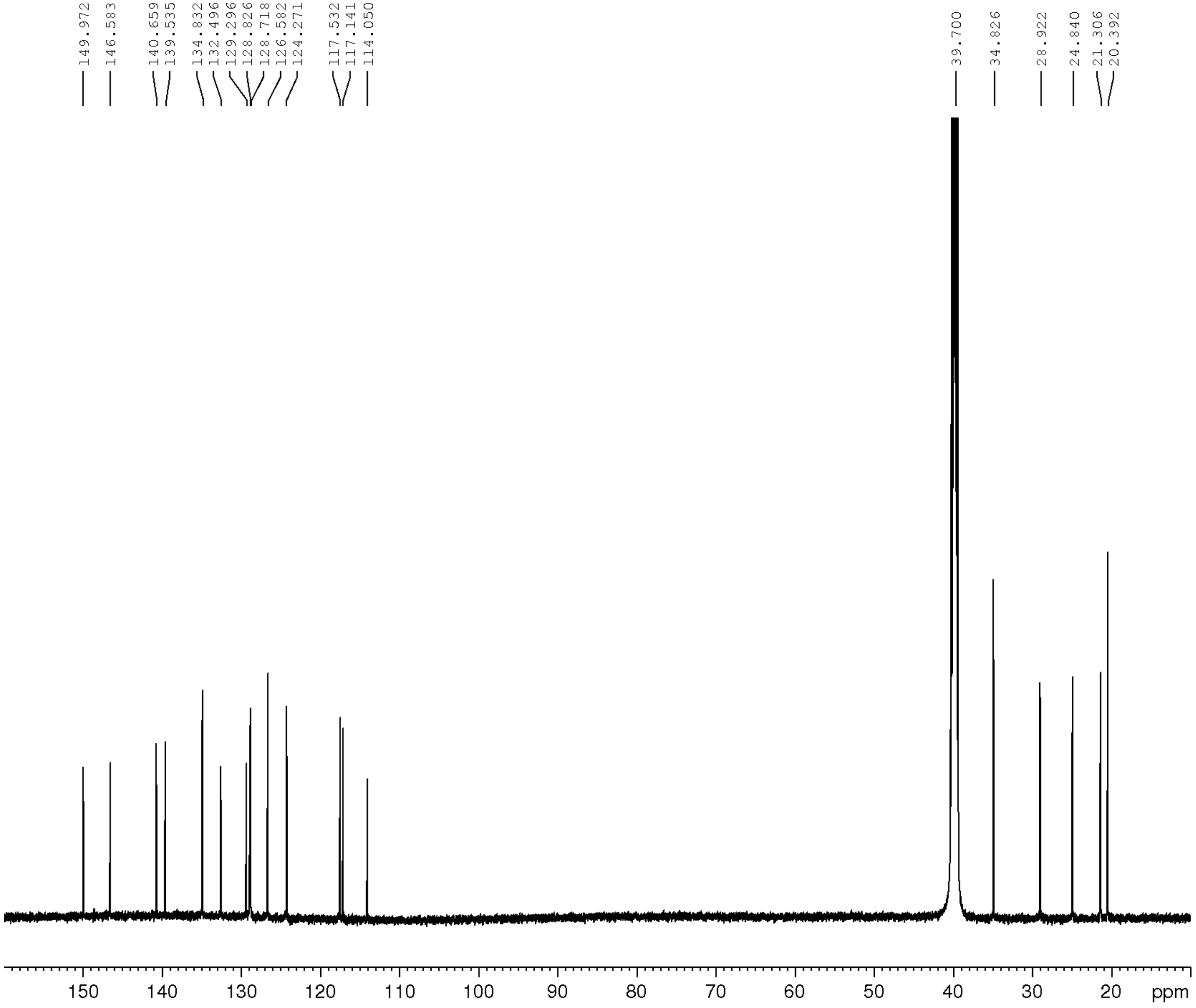

Standard 13C
143519
PGY0782_1
Pusztai Gyongyver
2024.09.17. (DA)

Current Data Parameters

NAME	143519
EXPNO	22
PROCNO	1

F2 - Acquisition Parameters

Date_	20240918
Time	12.05 h
INSTRUM	spect
PROBHD	Z145856_0002 (
PULPROG	zgpg30
TD	65536
SOLVENT	DMSO
NS	2048
DS	4
SWH	36231.883 Hz
FIDRES	1.105709 Hz
AQ	0.9043968 sec
RG	196.07
DW	13.800 usec
DE	18.00 usec
TE	295.0 K
D1	1.00000000 sec
D11	0.03000000 sec
TD0	1
SFO1	150.8852070 MHz
NUC1	13C
P1	9.90 usec
PLW1	71.00000000 W
SFO2	600.0024000 MHz
NUC2	1H
CPDPRG[2	waltz16
PCPD2	80.00 usec
PLW2	32.90000153 W
PLW12	0.70370001 W
PLW13	0.35339001 W

F2 - Processing parameters

SI	131072
SF	150.8701594 MHz
WDW	EM
SSB	0
LB	1.00 Hz
GB	0
PC	1.40

IR spectrum of (*E*)-**9a**

**143519**	
**PGY0782_1**	

Pusztai Gyongyver	KP
KBr	9/17/2024

BRUKER Alpha
Resolution: 2 cm-1
Number of Scans: 16

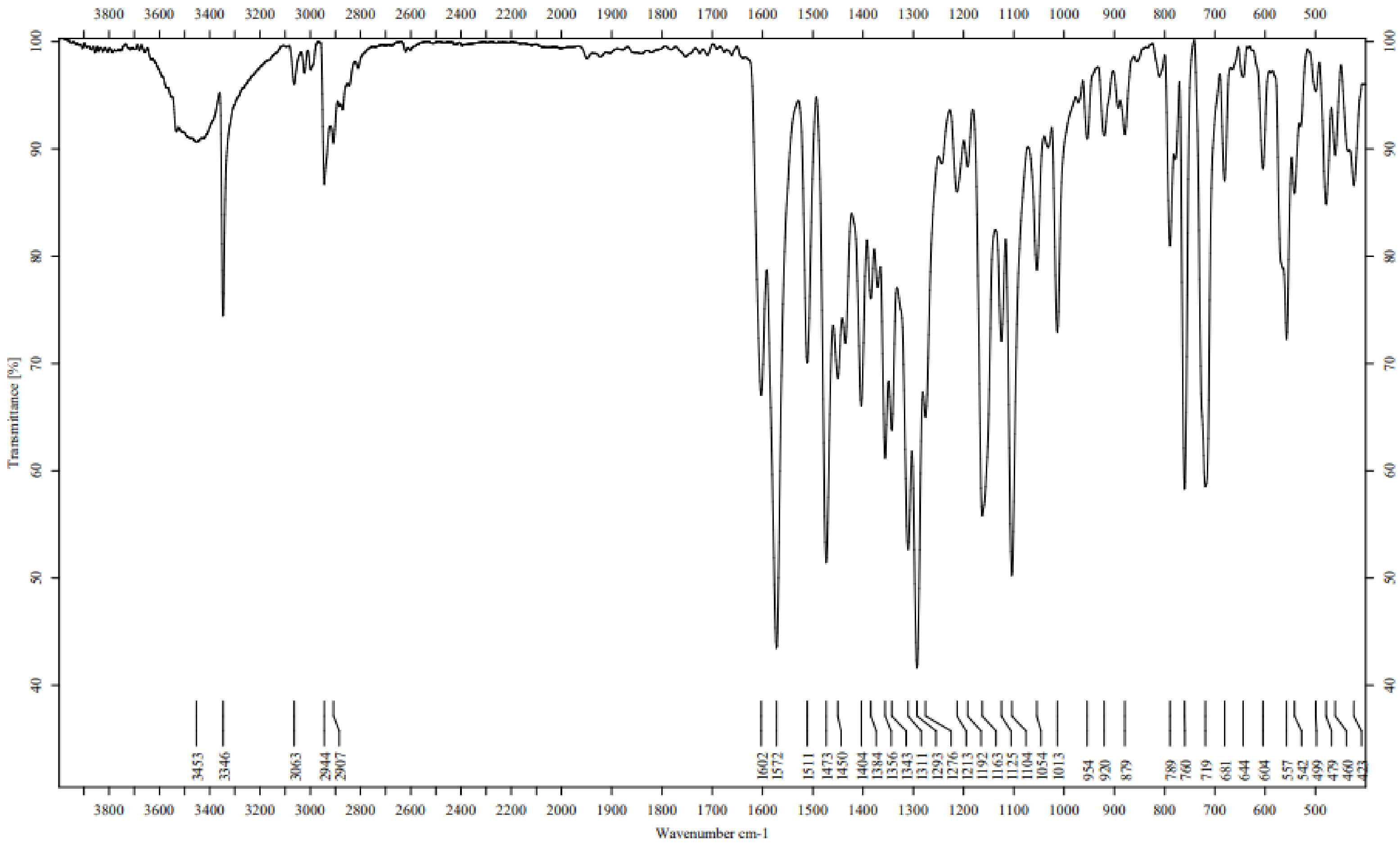

HRMS spectrum of (*E*)-**9a**

Spectrum Plot Report

Name	PGY0782_1, Pusztai Gyongyver	Rack Pos.		Instrument	7250A with DIP	Operator	MM
Inj. Vol. (ul)	0.5	Plate Pos.		IRM Status	Success		
Data File	143519msqtof_dip.D	Method (Acq)	DIP_70eV.M	Comment		Acq. Time (Local)	9/30/2024 1:27:33 PM (UTC+02:00)

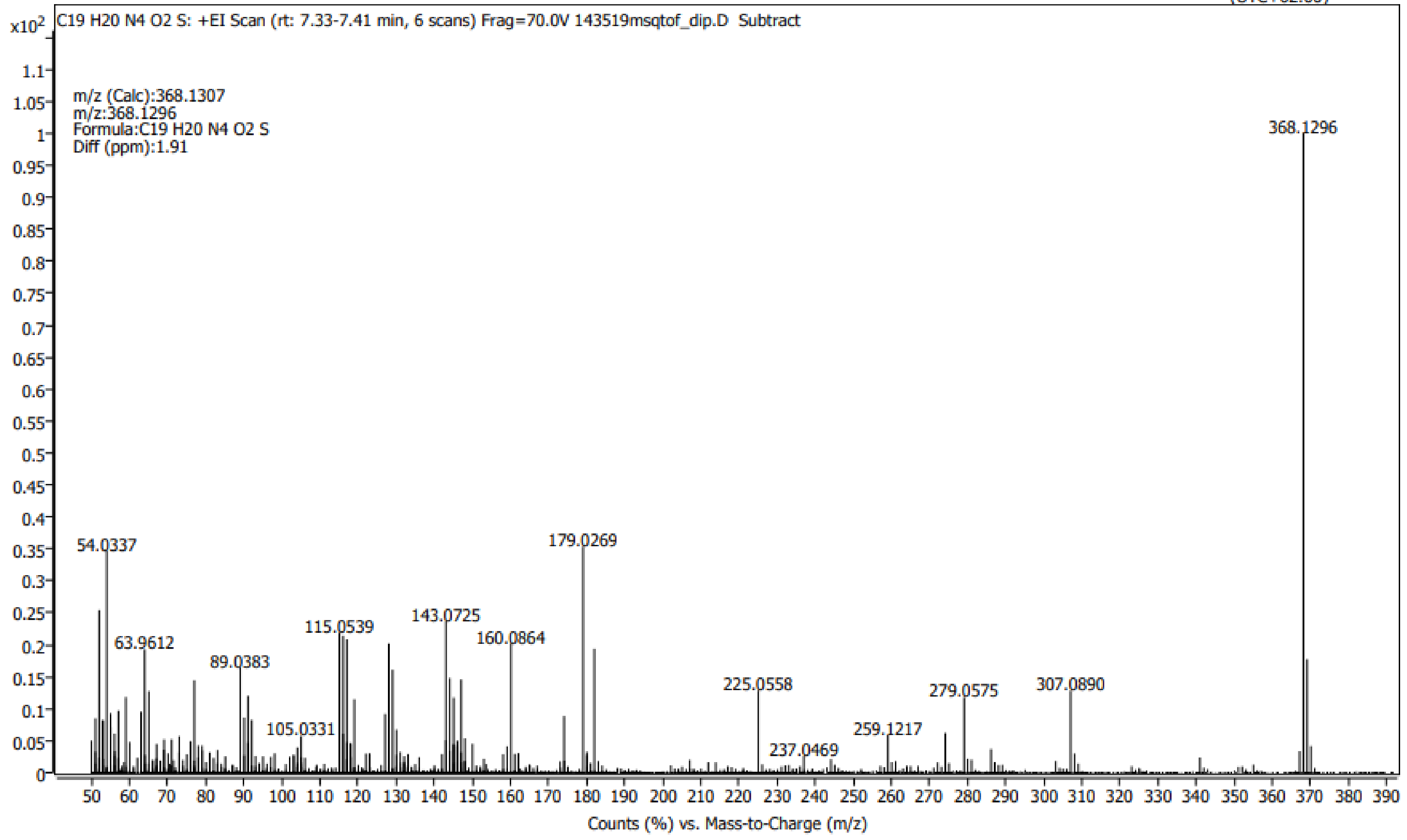

^{1}H NMR spectrum of (*E*)-**7f**

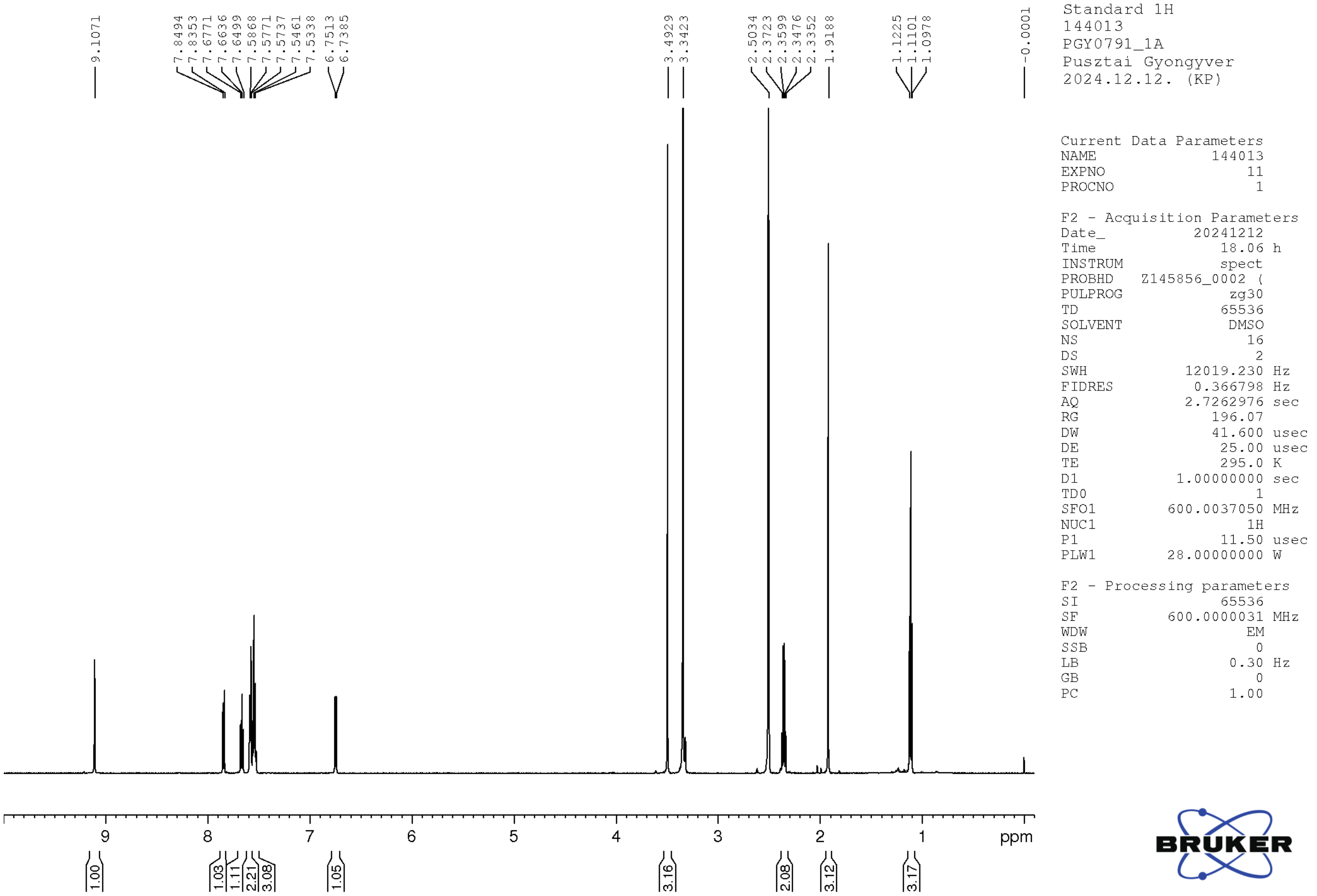

^{13}C NMR spectrum of (*E*)-**7f**

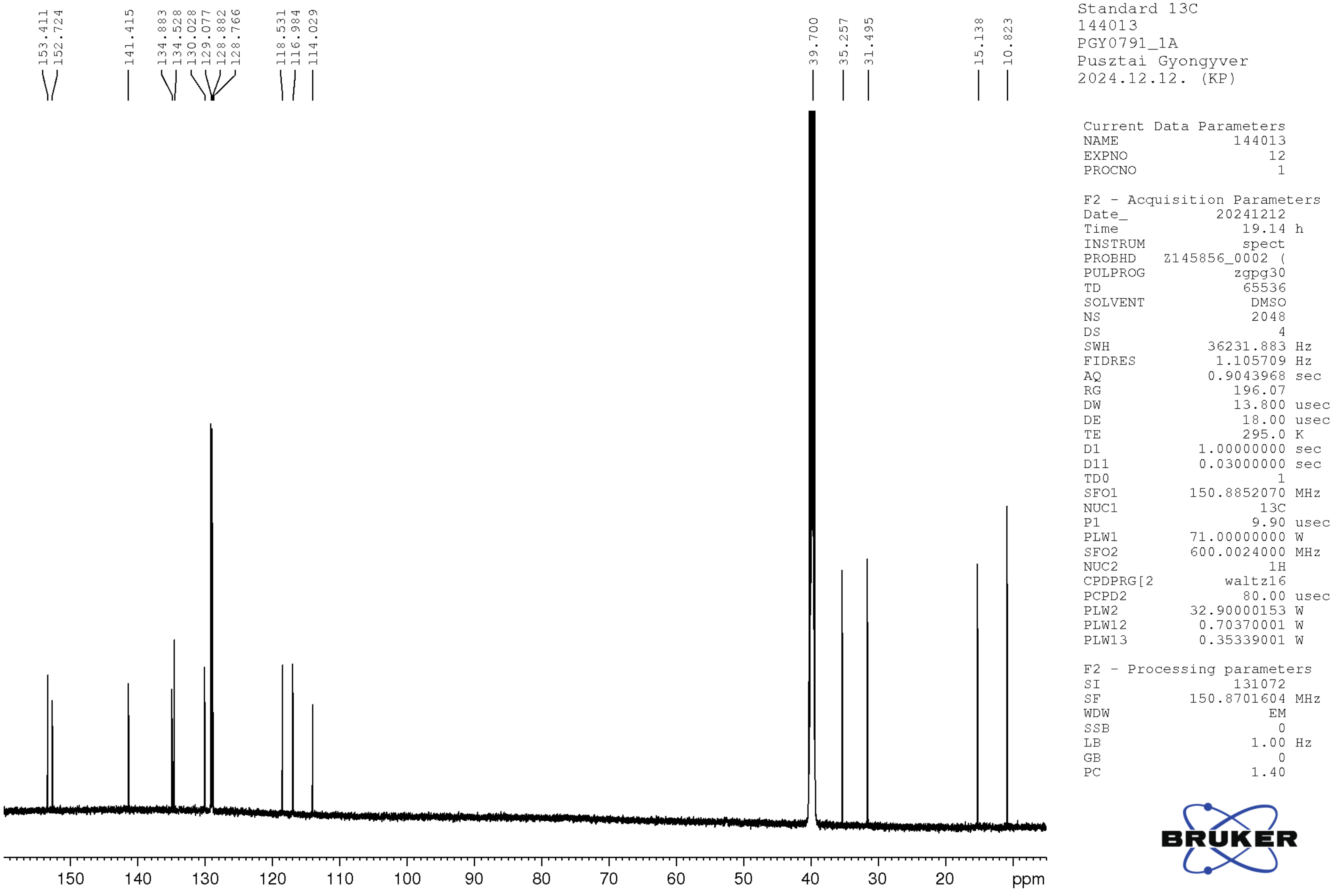

IR spectrum of (*E*)-**7f**

**144013**	Pusztai Gyongyver	KP		BRUKER Alpha
**PGY0791_1A**	KBr	12/16/2024		Resolution: 2 cm-1
				Number of Scans: 16

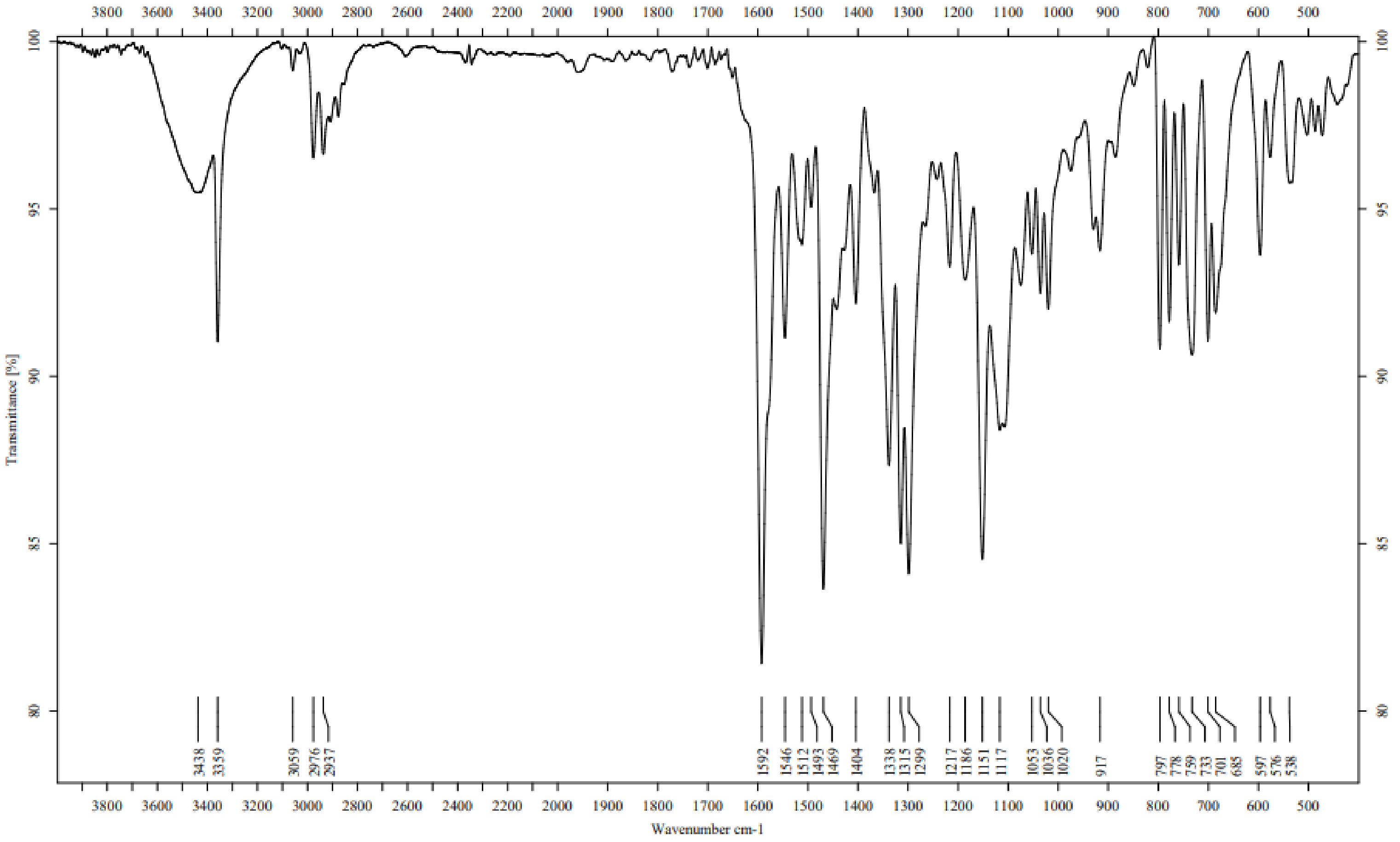

HRMS spectrum of (E)-**7f**

Spectrum Plot Report

Agilent Trusted Answers

Name	PGZ0791_1A, Pusztai Gyongyver	Rack Pos.		Instrument	7250A with DIP	Operator	MM
Inj. Vol. (ul)	0.5	Plate Pos.		IRM Status	Success		
Data File	144013_qtof_dip2.D	Method (Acq)	DIP_70eV.M	Comment		Acq. Time (Local)	12/13/2024 12:41:35 PM (UTC+01:00)

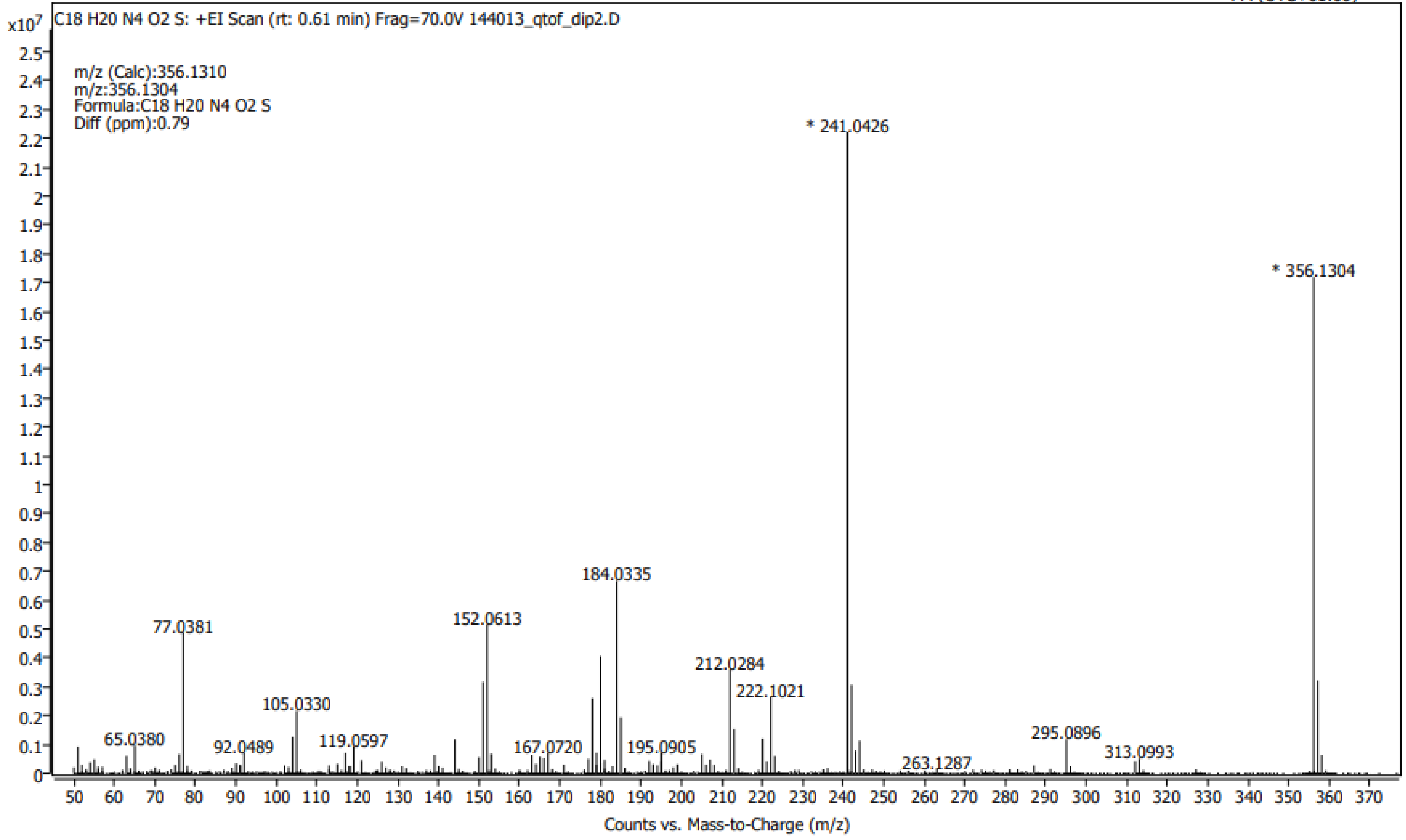

^{1}H NMR spectrum of **7g**

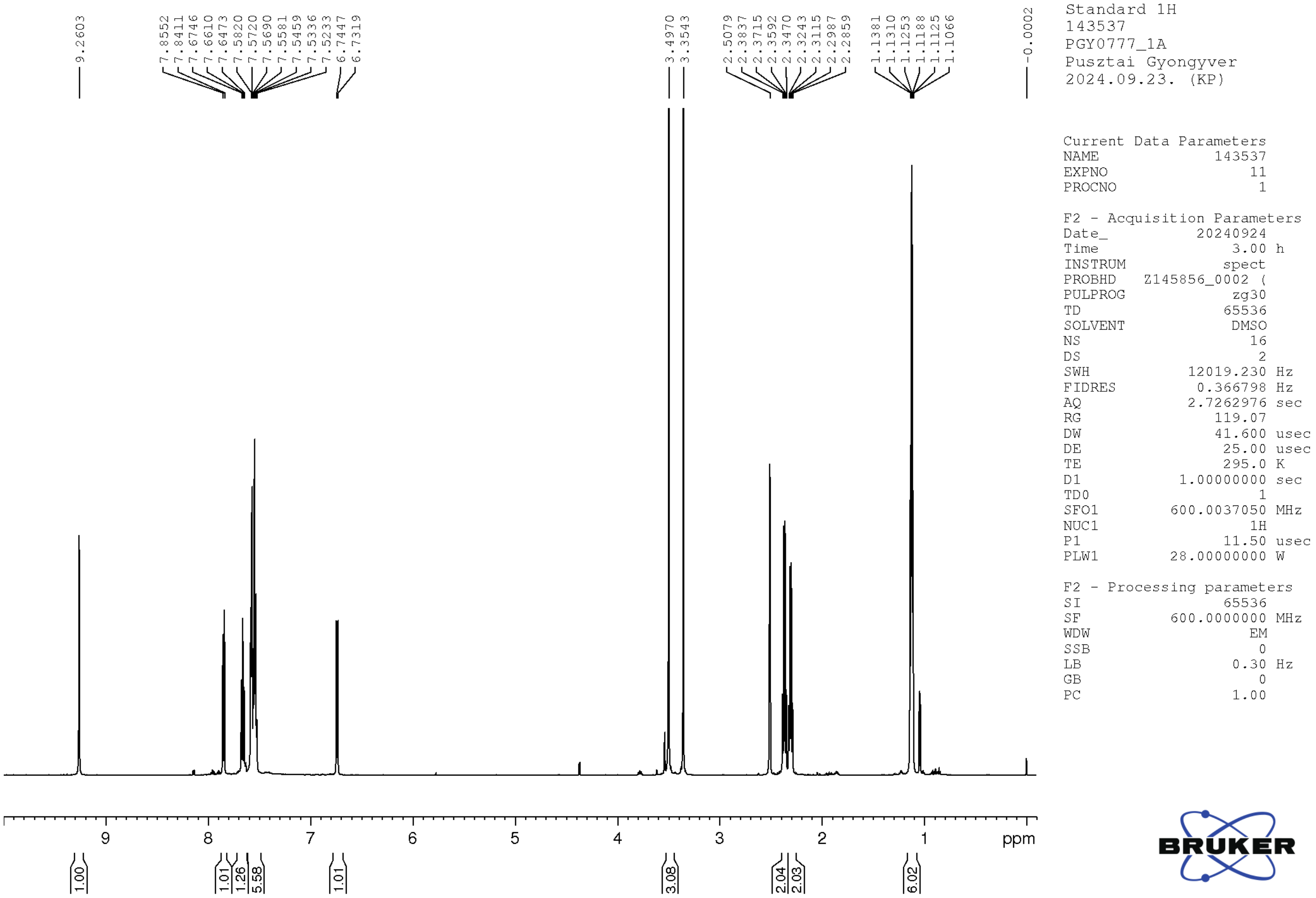

^{13}C NMR spectrum of **7g**

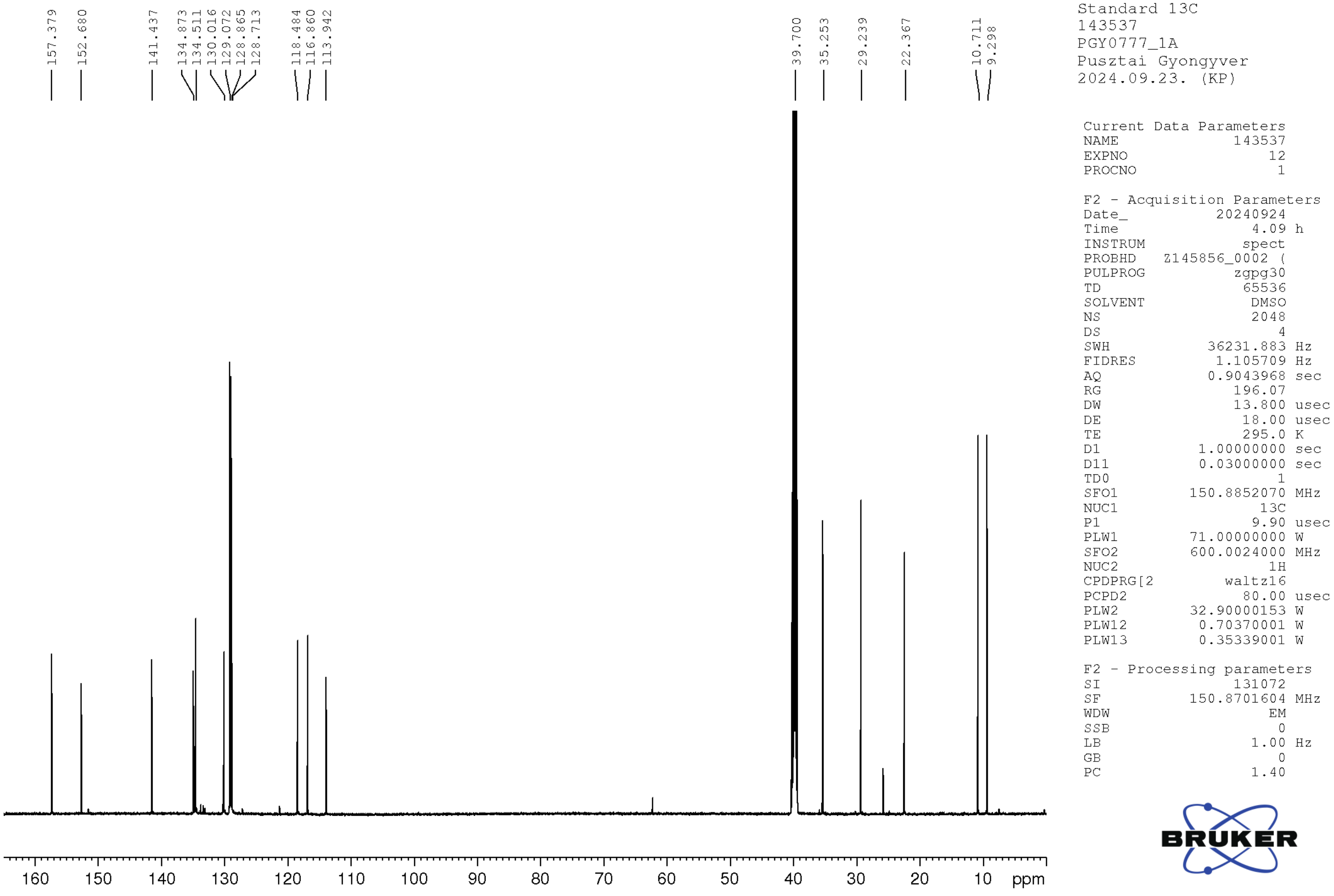

IR spectrum of **7g**

**143537**	
**PGY0777_1A**	

Pusztai Gyongyver	KP
KBr	9/23/2024

BRUKER Alpha
Resolution: 2 cm-1
Number of Scans: 16

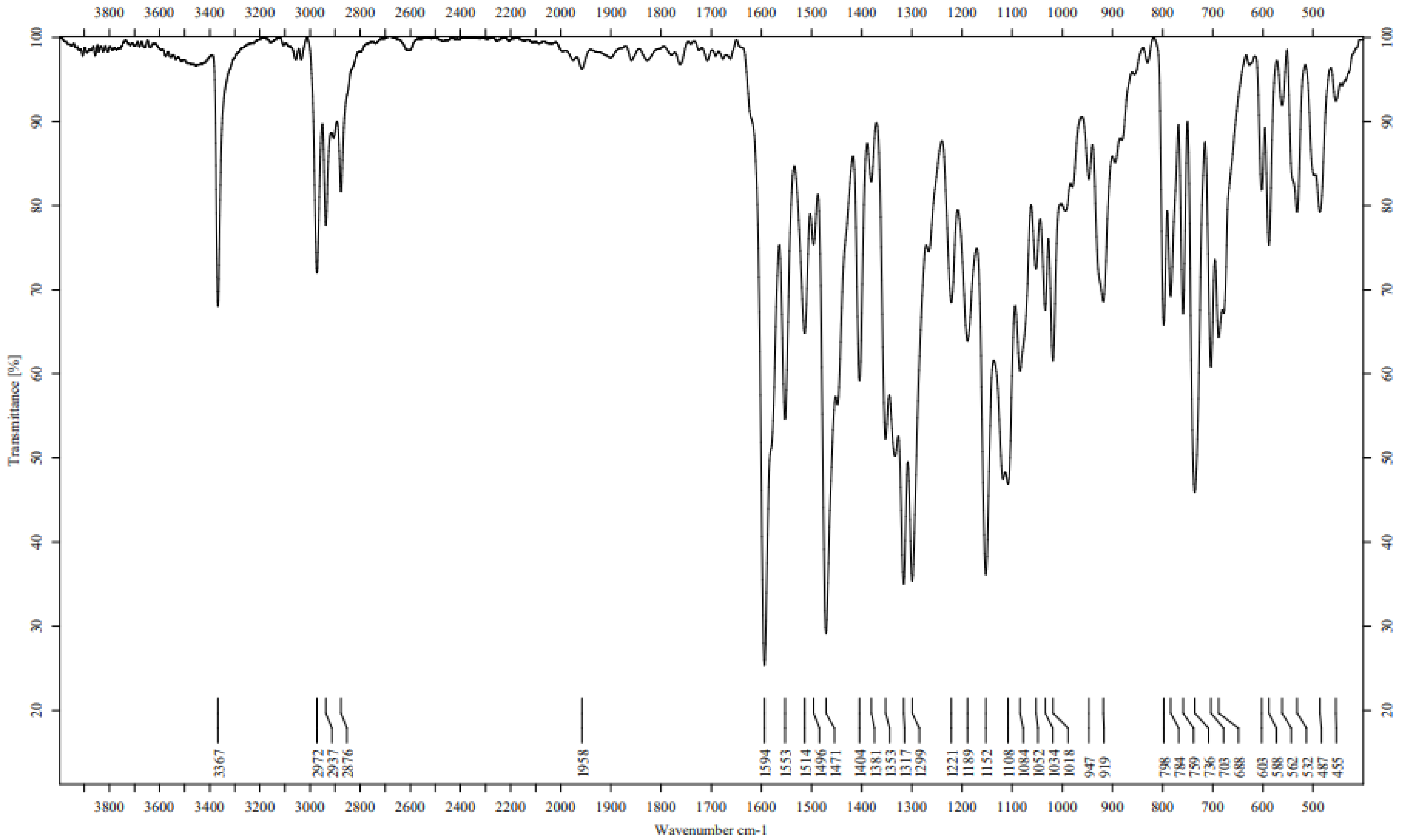

HRMS spectrum of **7g**

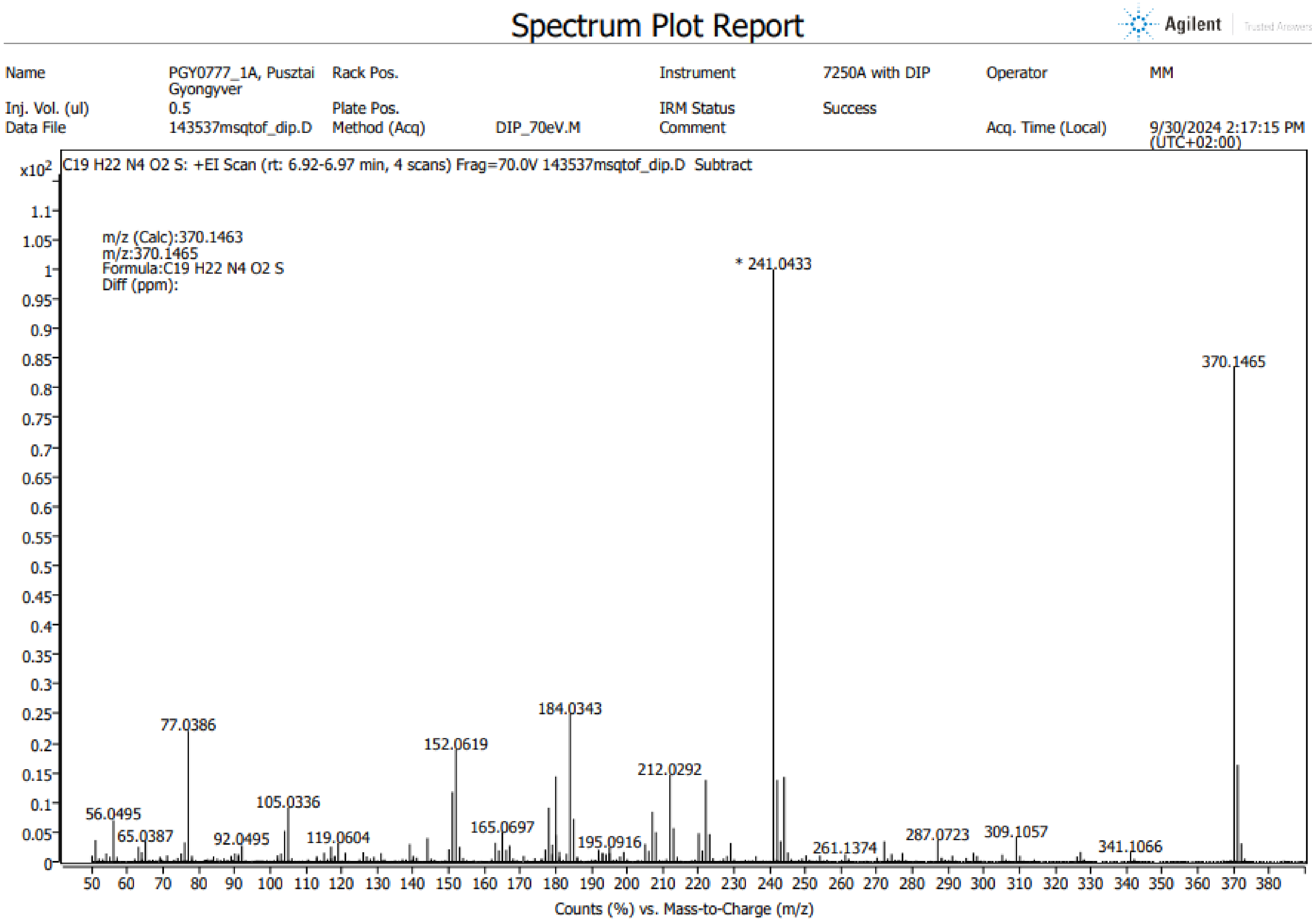

^{1}H NMR spectrum of **7i**

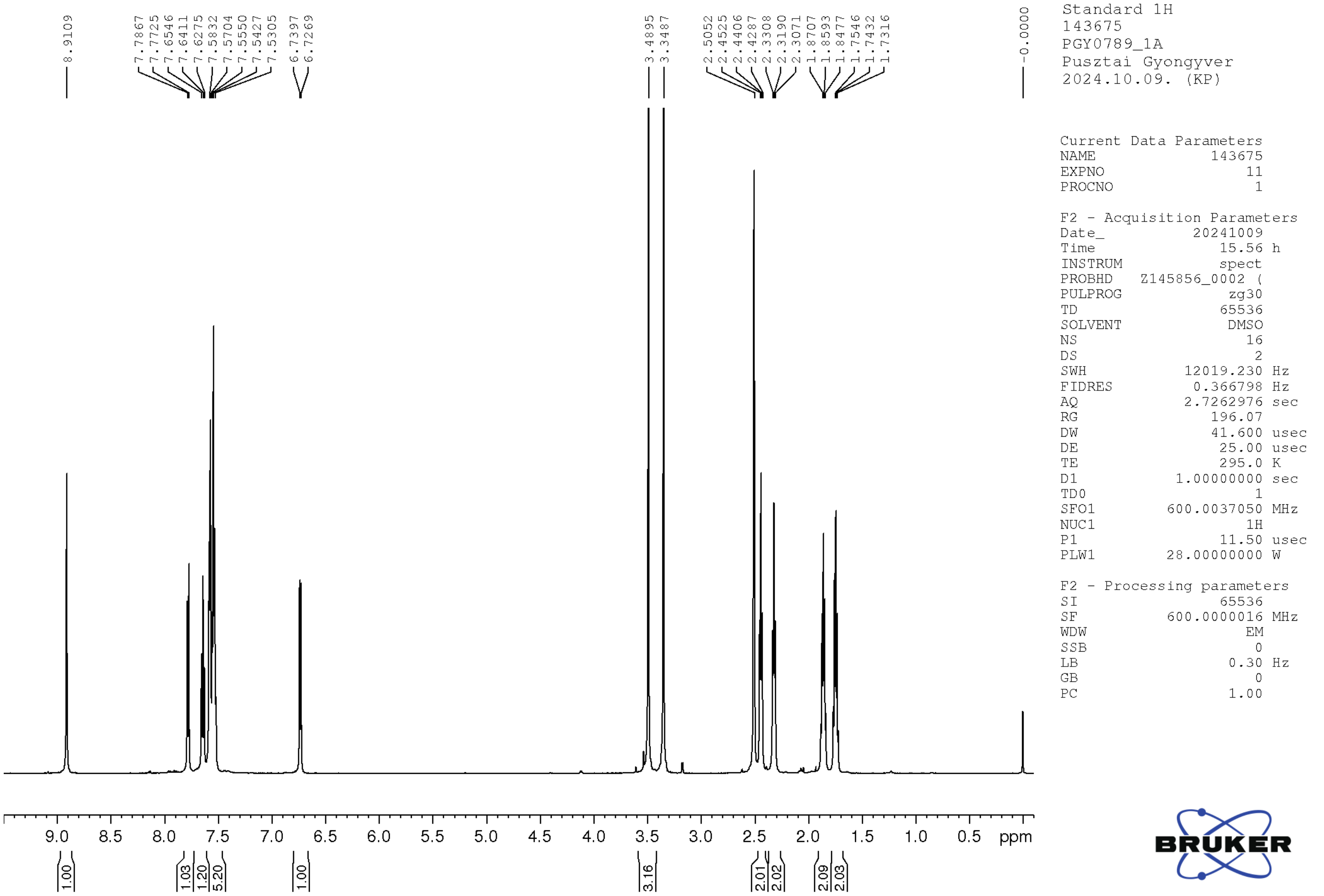

^{13}C NMR spectrum of **7i**

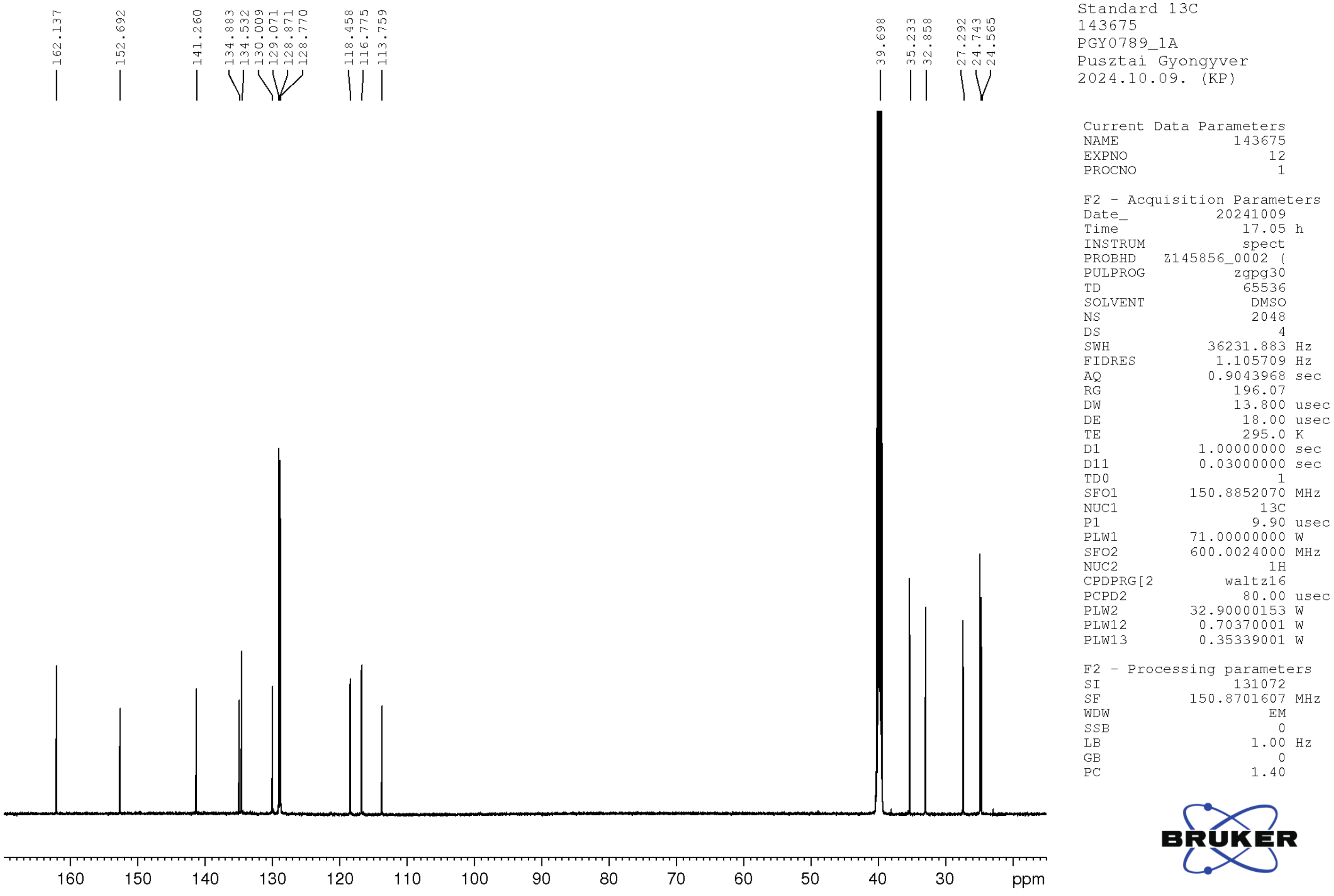

IR spectrum of **7i**

**143675**	
**PGY0789_1A**	

Pusztai Gyongyver	KP
KBr	10/9/2024

BRUKER Alpha
Resolution: 2 cm-1
Number of Scans: 16

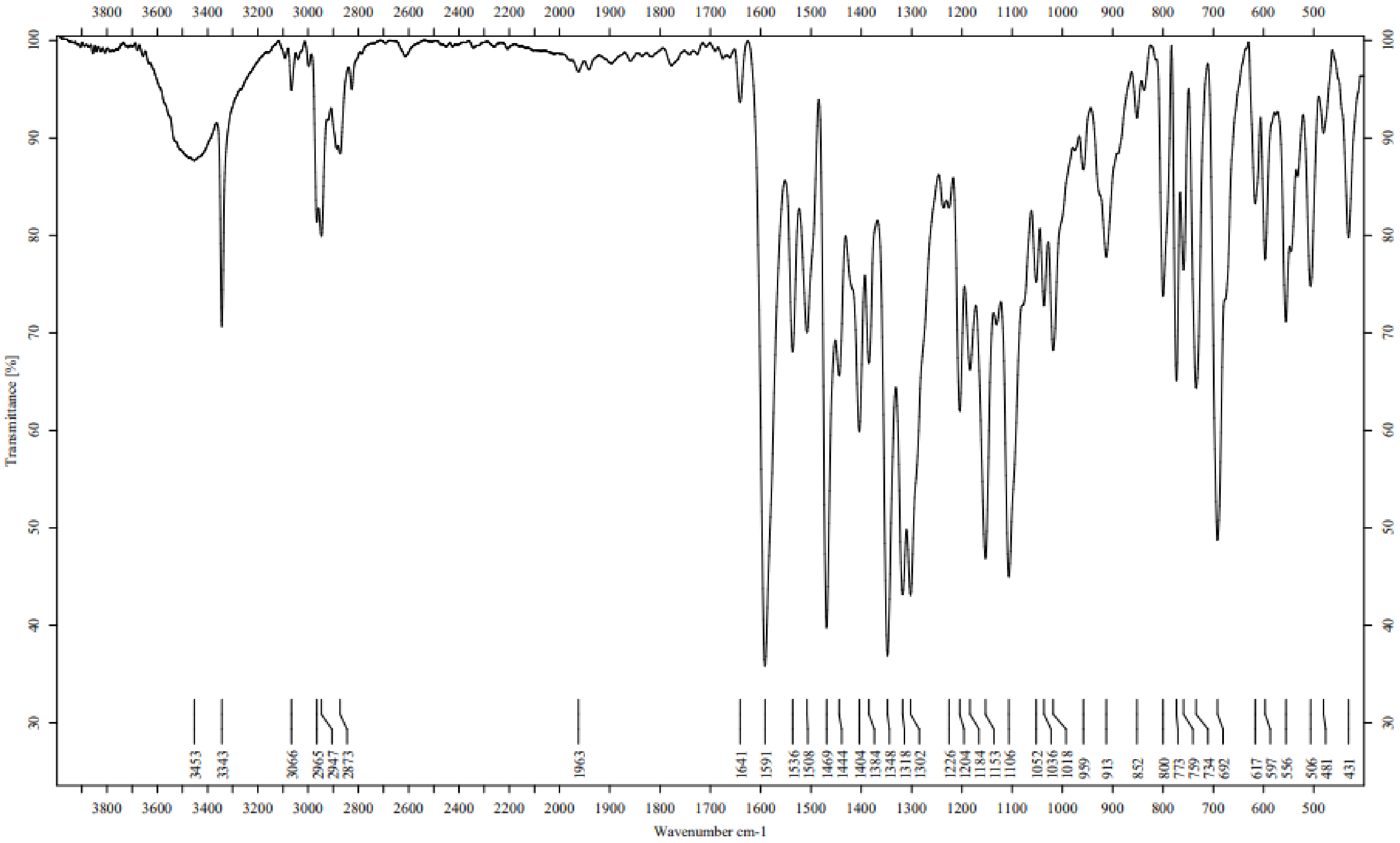

HRMS spectrum of **7i**

Sample: PGY0789_1A	Lab code: Nsz - 29676
Submitter: Pusztai Gyongyve	Project: Other

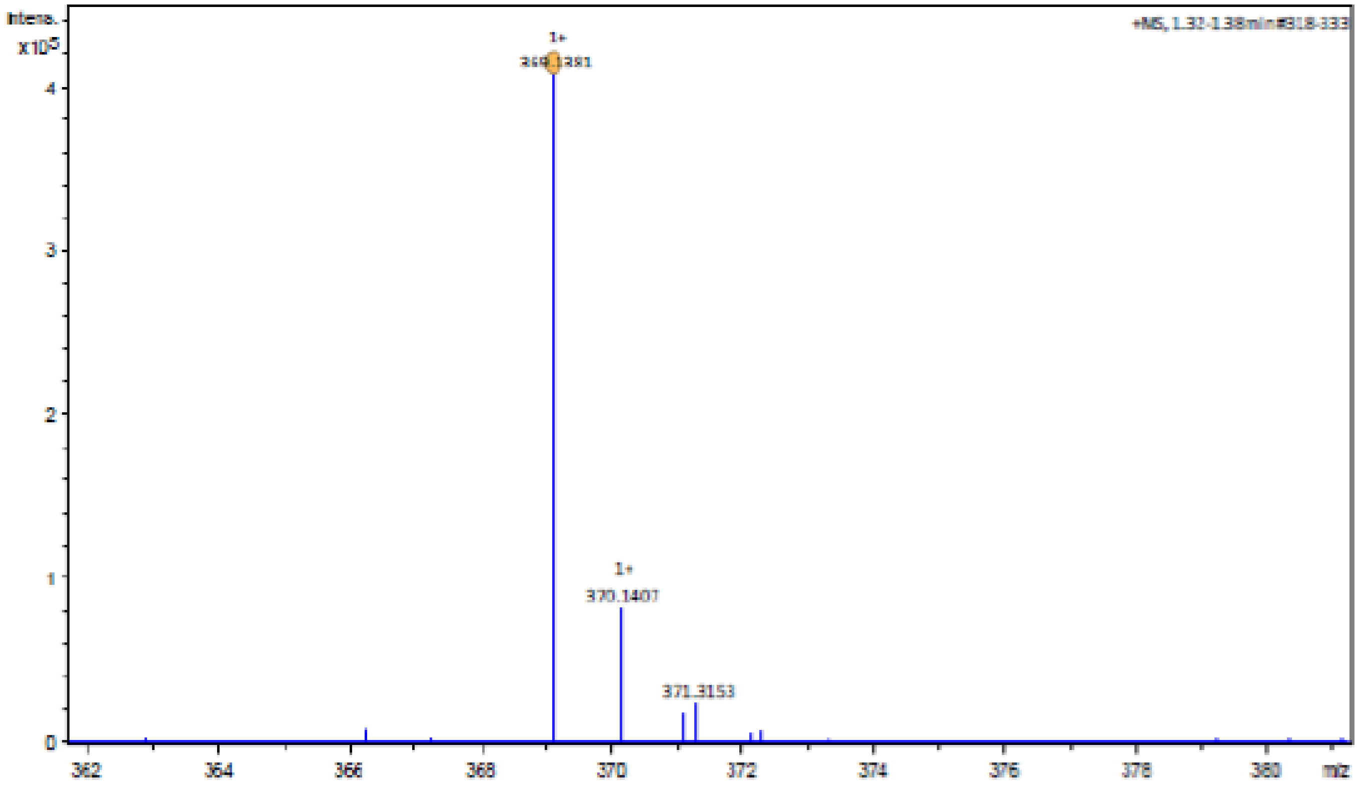

Minta: 29676_PGY0789_1A
Spektrum: MS_1.32-1.38min

Measured	Intens	Formula	Theoretical	[ppm]	[mmu]
369.1381	100	C19H21N4O2S1	369.1380	0.3	-0.1

^{1}H NMR spectrum of **7j**

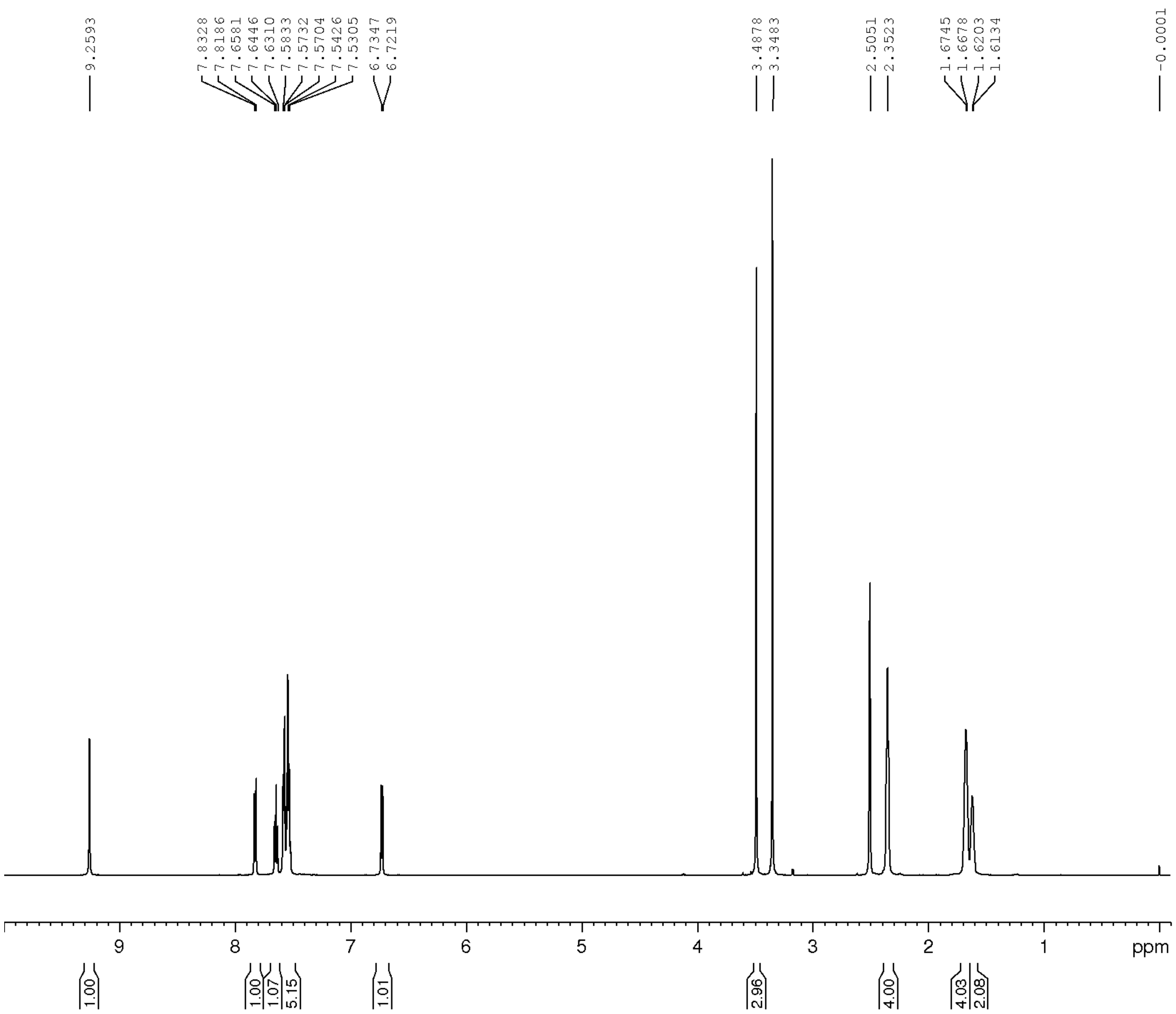


```
Standard 1H
143440
PGY0778_1
Pusztai Gyongyver
2024.09.19. (KP)

Current Data Parameters
NAME              143440
EXPNO                 21
PROCNO                 1

F2 - Acquisition Parameters
Date_           20240919
Time               15.53 h
INSTRUM            spect
PROBHD    Z145856_0002 (
PULPROG             zg30
TD                 65536
SOLVENT             DMSO
NS                    16
DS                     2
SWH            12019.230 Hz
FIDRES          0.366798 Hz
AQ             2.7262976 sec
RG                196.07
DW                41.600 usec
DE                 25.00 usec
TE                 295.0 K
D1            1.00000000 sec
TD0                    1
SFO1       600.0037050 MHz
NUC1                  1H
P1                 11.50 usec
PLW1         28.00000000 W

F2 - Processing parameters
SI                 65536
SF         600.0000018 MHz
WDW                   EM
SSB                    0
LB                  0.30 Hz
GB                     0
PC                  1.00
```

^{13}C NMR spectrum of **7j**

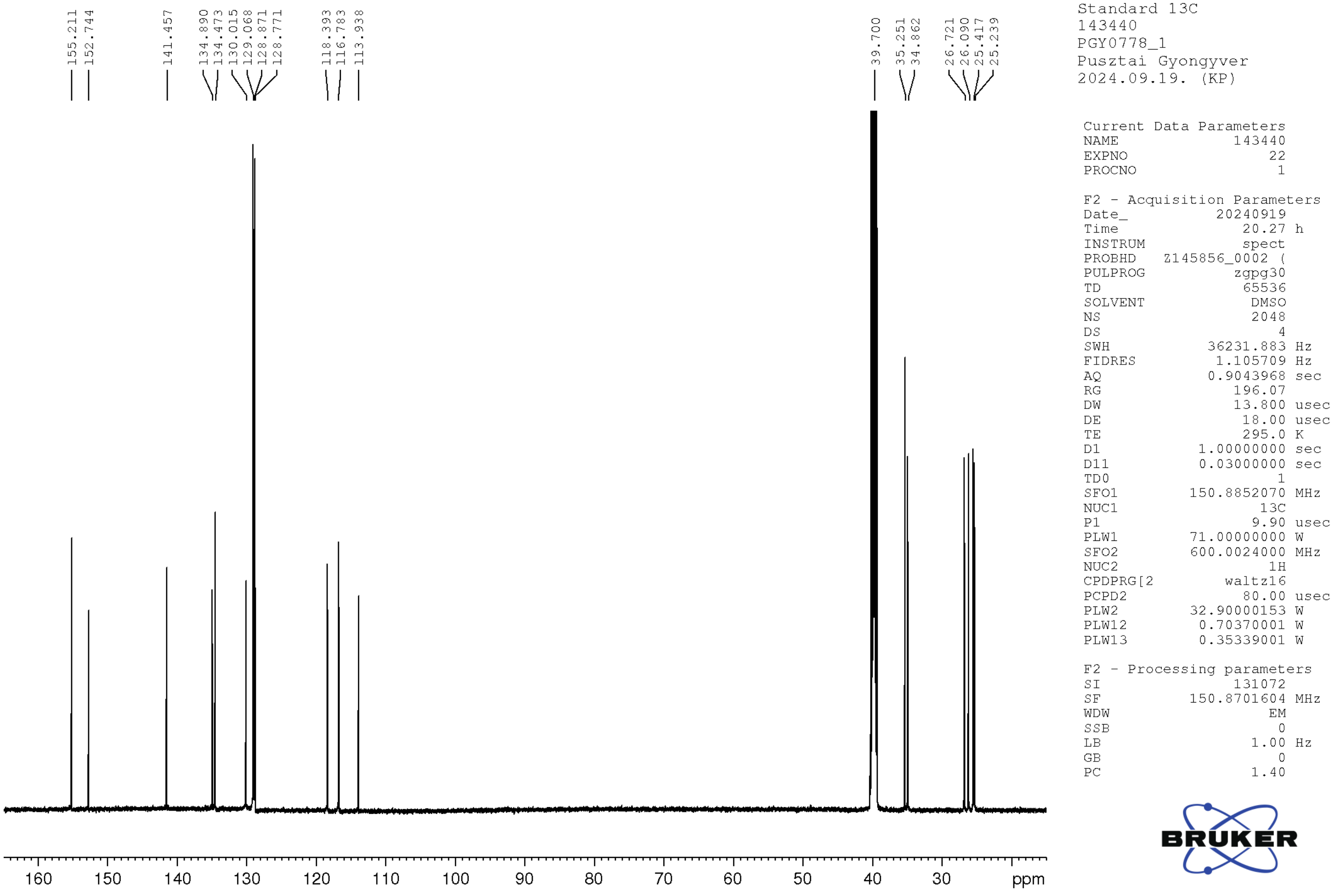

IR spectrum of **7j**

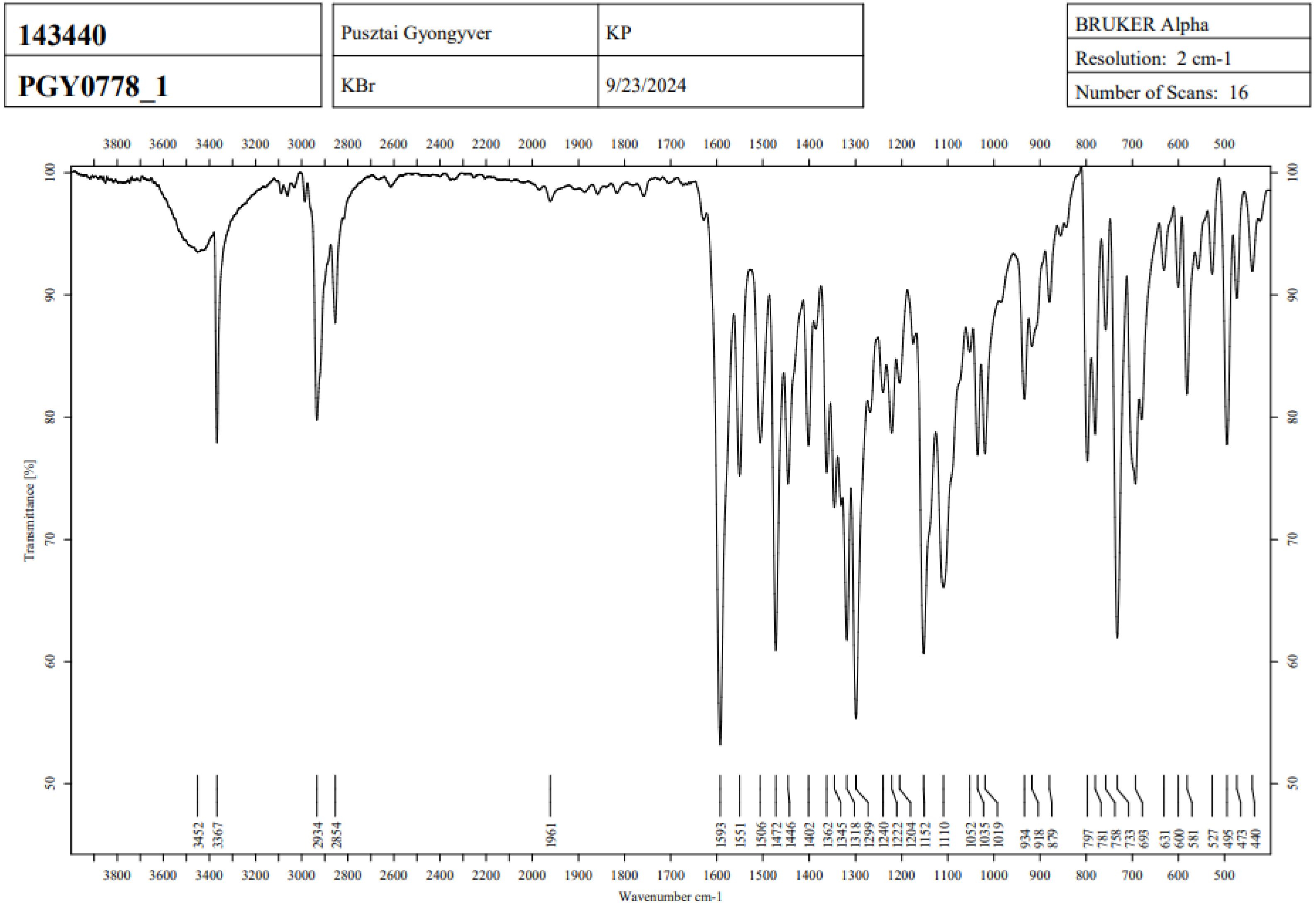

HRMS spectrum of **7j**

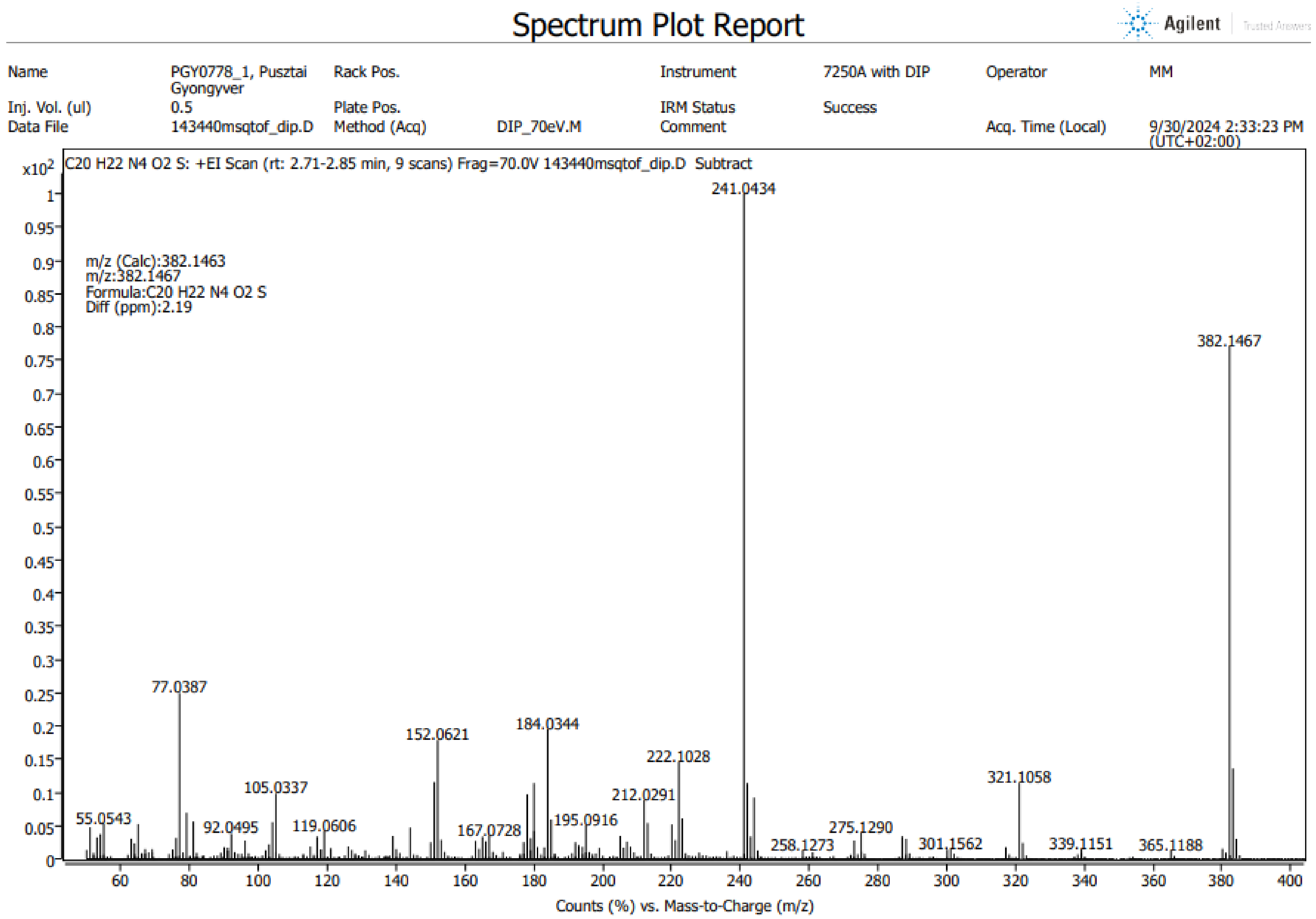

^{1}H NMR spectrum of (*E*)-**9b**

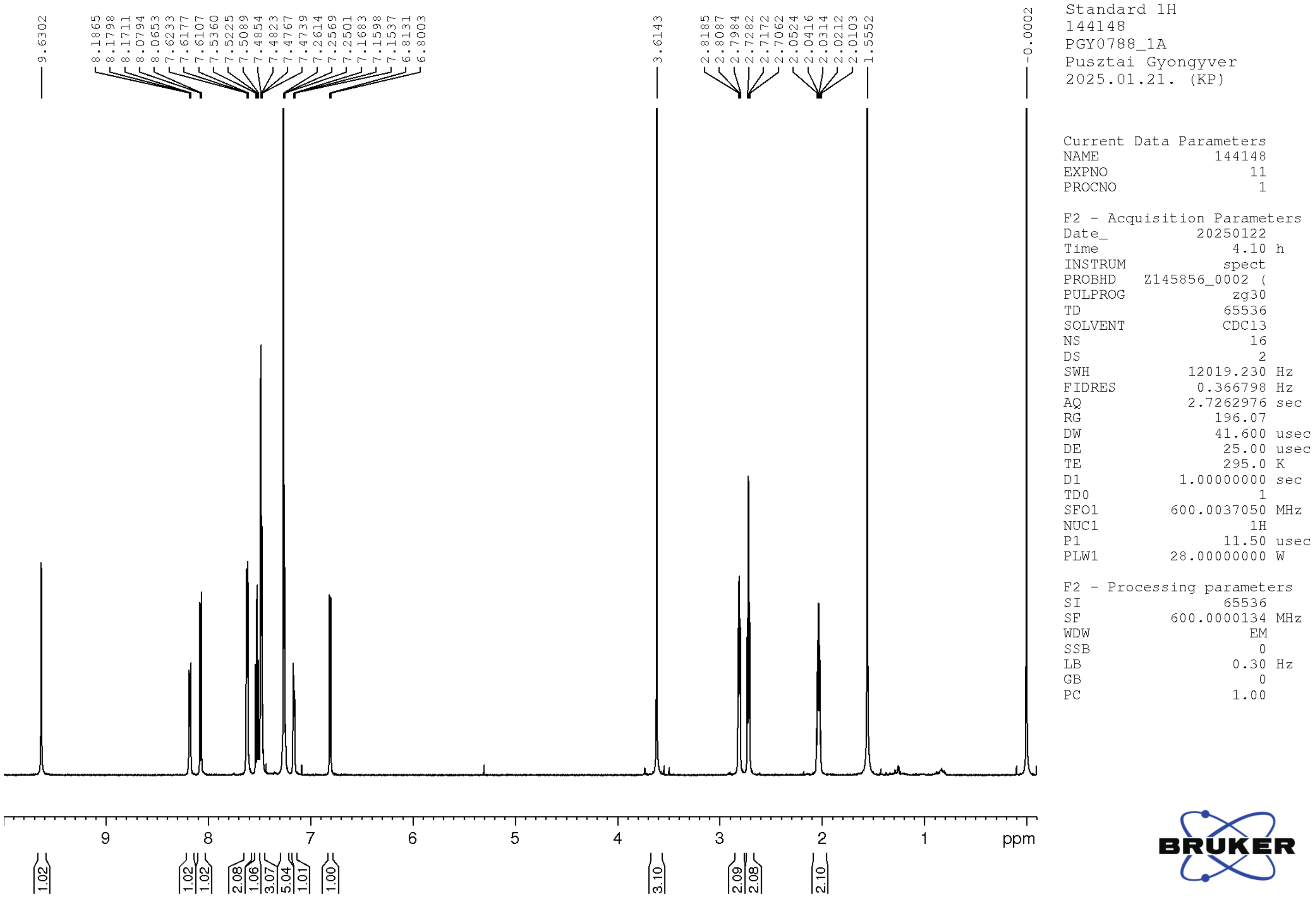

^{13}C NMR spectrum of (*E*)-**9b**

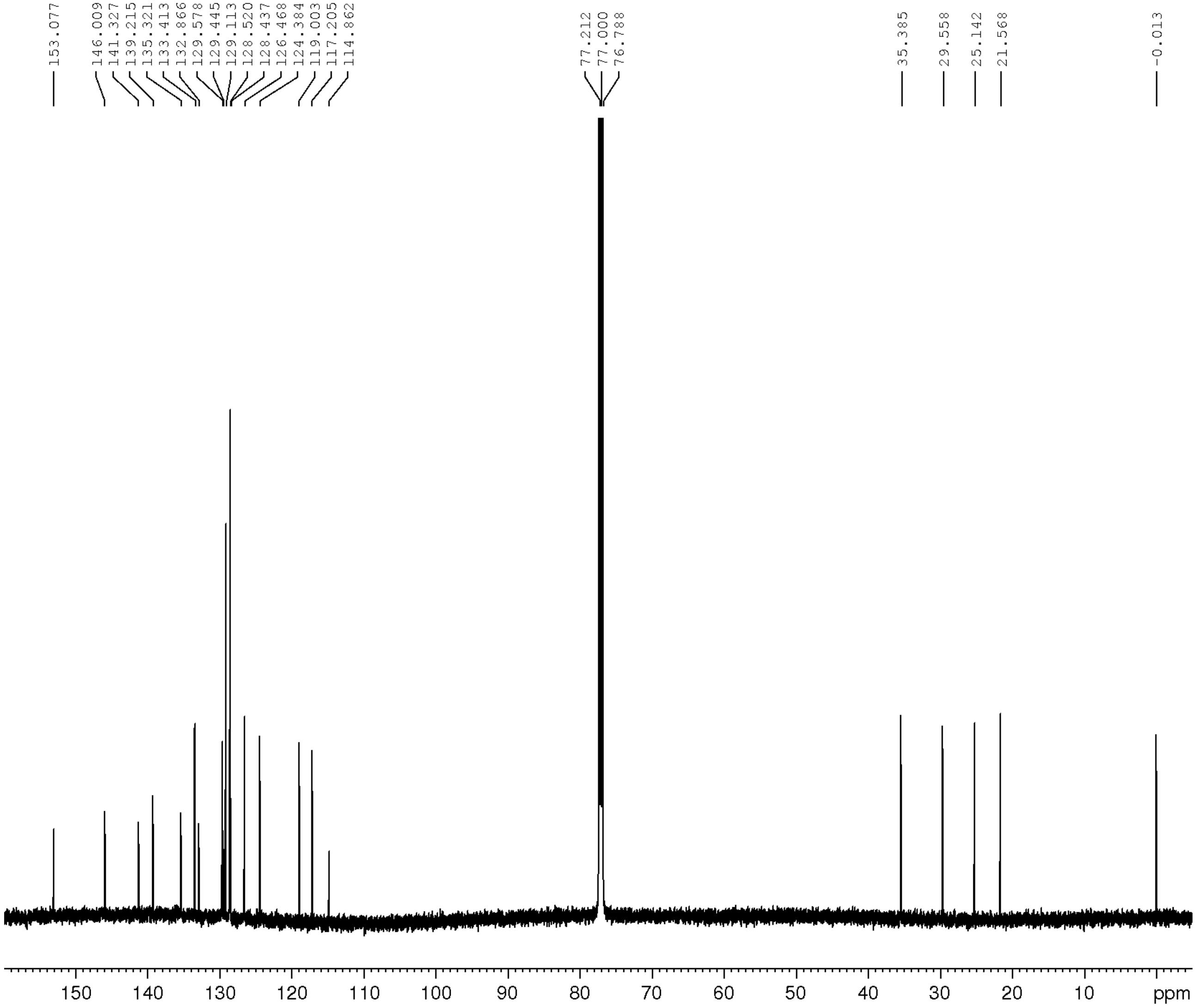

Standard 13C
144148
PGY0788_1A
Pusztai Gyongyver
2025.01.21. (KP)

Current Data Parameters
NAME 144148
EXPNO 12
PROCNO 1

F2 - Acquisition Parameters
Date_ 20250122
Time 5.19 h
INSTRUM spect
PROBHD Z145856_0002 (
PULPROG zgpg30
TD 65536
SOLVENT CDCl3
NS 2048
DS 4
SWH 36231.883 Hz
FIDRES 1.105709 Hz
AQ 0.9043968 sec
RG 196.07
DW 13.800 usec
DE 18.00 usec
TE 295.0 K
D1 1.00000000 sec
D11 0.03000000 sec
TD0 1
SFO1 150.8852070 MHz
NUC1 13C
P1 9.90 usec
PLW1 71.00000000 W
SFO2 600.0024000 MHz
NUC2 1H
CPDPRG[2 waltz16
PCPD2 80.00 usec
PLW2 32.90000153 W
PLW12 0.70370001 W
PLW13 0.35339001 W

F2 - Processing parameters
SI 131072
SF 150.8701254 MHz
WDW EM
SSB 0
LB 1.00 Hz
GB 0
PC 1.40

IR spectrum of (*E*)-**9b**

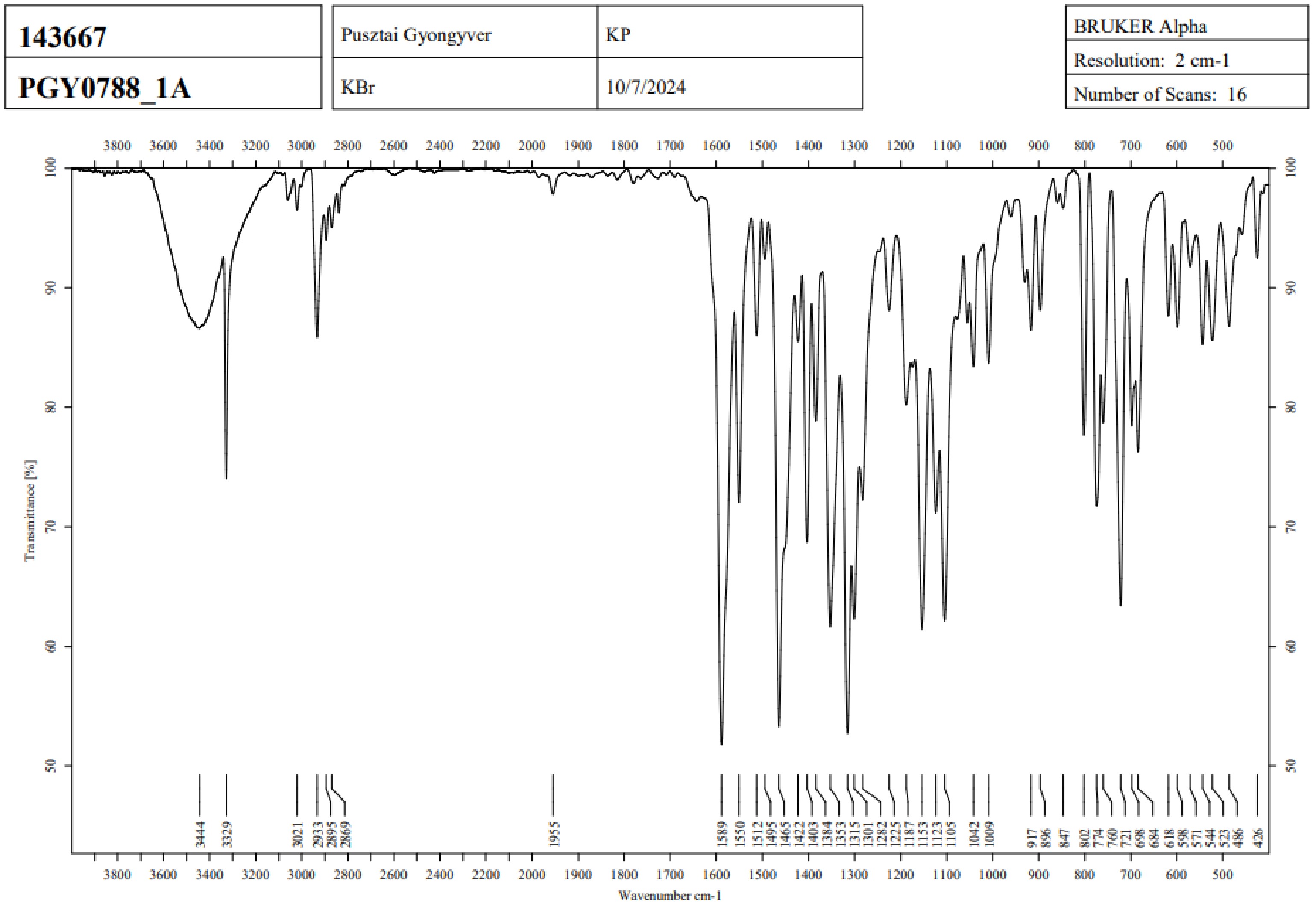

HRMS spectrum of (*E*)-**9b**

Spectrum Plot Report

Name	PGY0788_1A, Pusztai Gyongyver	Rack Pos.		Instrument	7250A with DIP	Operator	MM
Inj. Vol. (ul)	0.5	Plate Pos.		IRM Status	Success		
Data File	143667msaqtof_dip2.D	Method (Acq)	DIP_70eV_4min.M	Comment		Acq. Time (Local)	10/24/2024 1:24:33 PM (UTC+02:00)

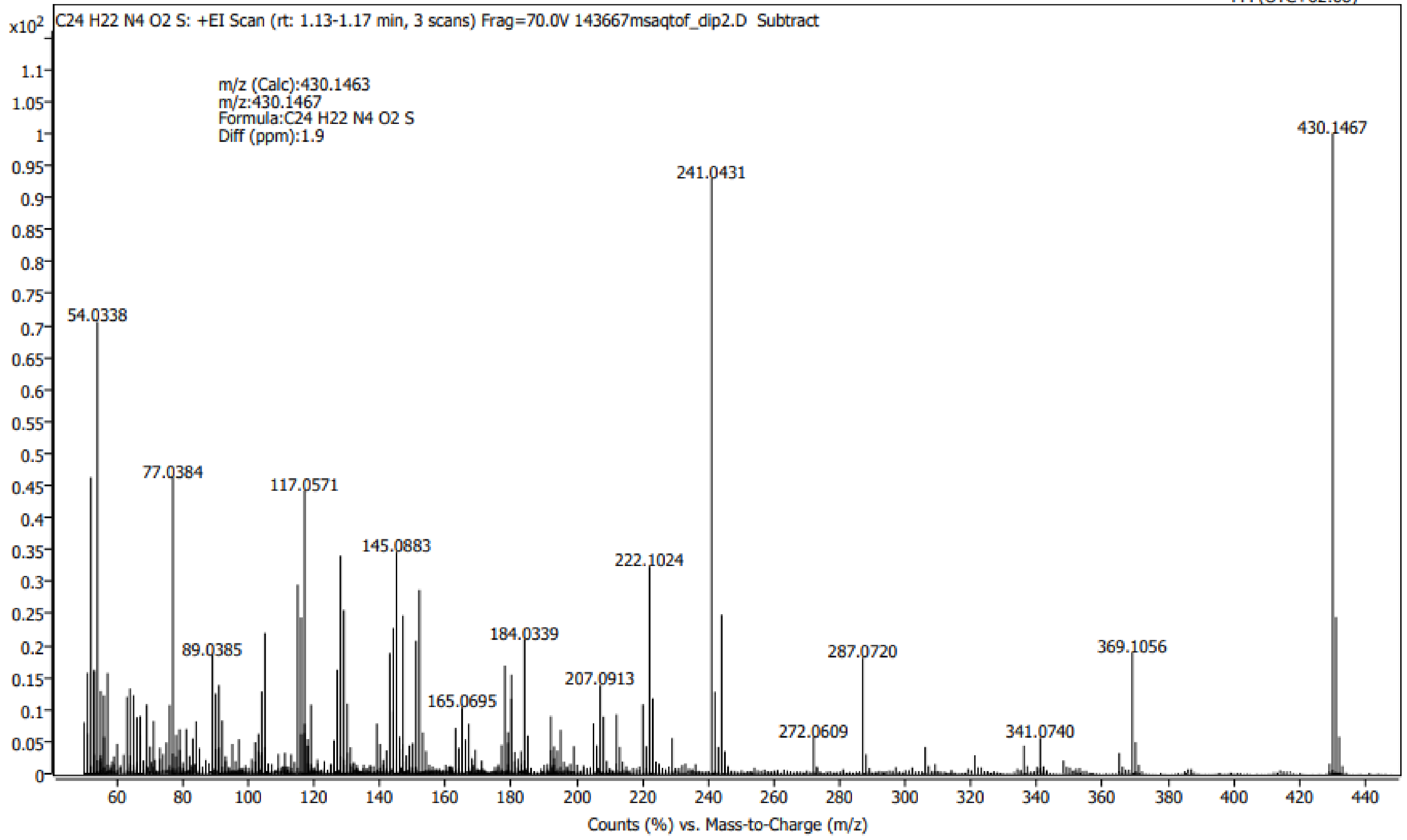

^{1}H NMR spectrum of **3a**

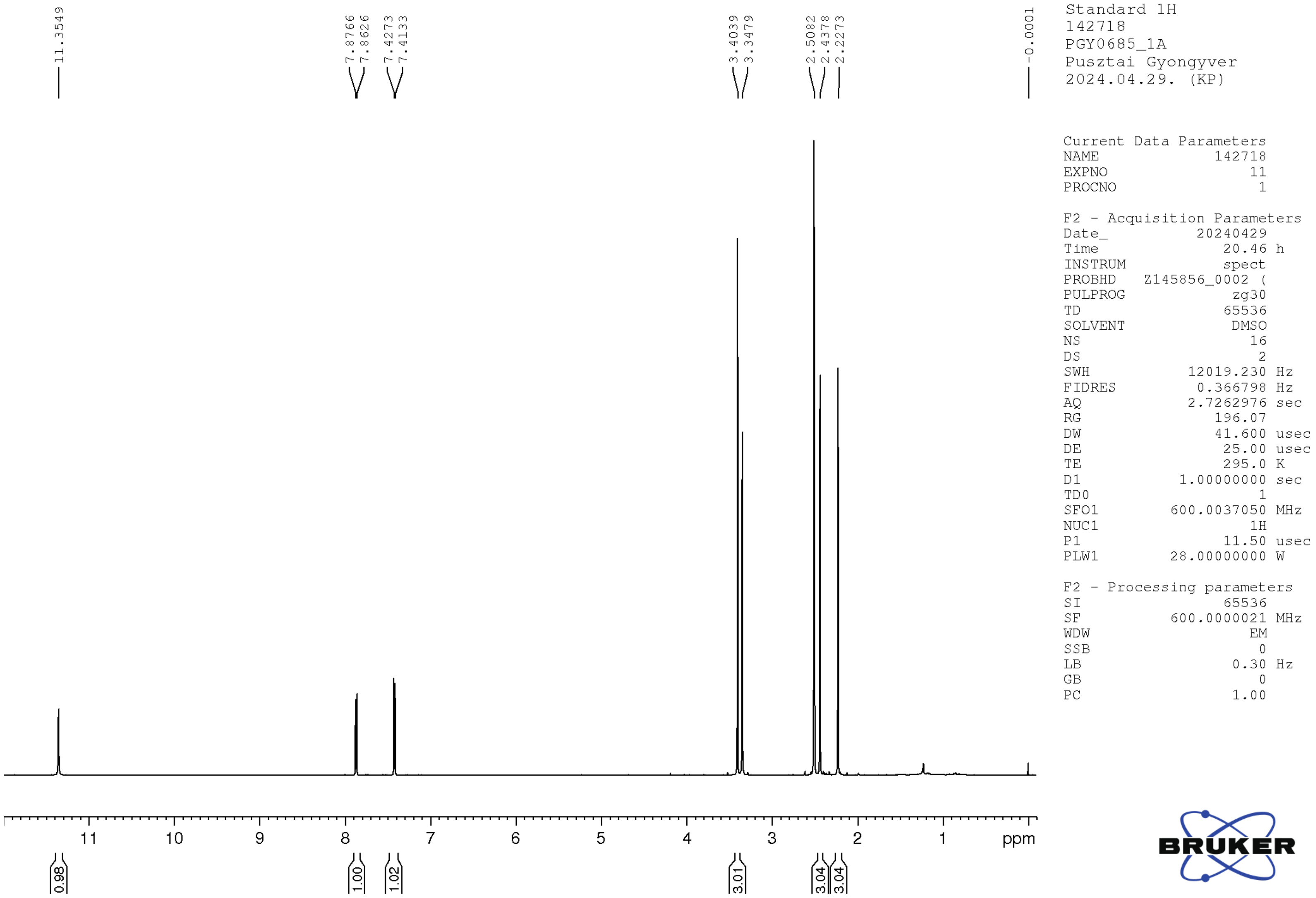

^{13}C NMR spectrum of **3a**

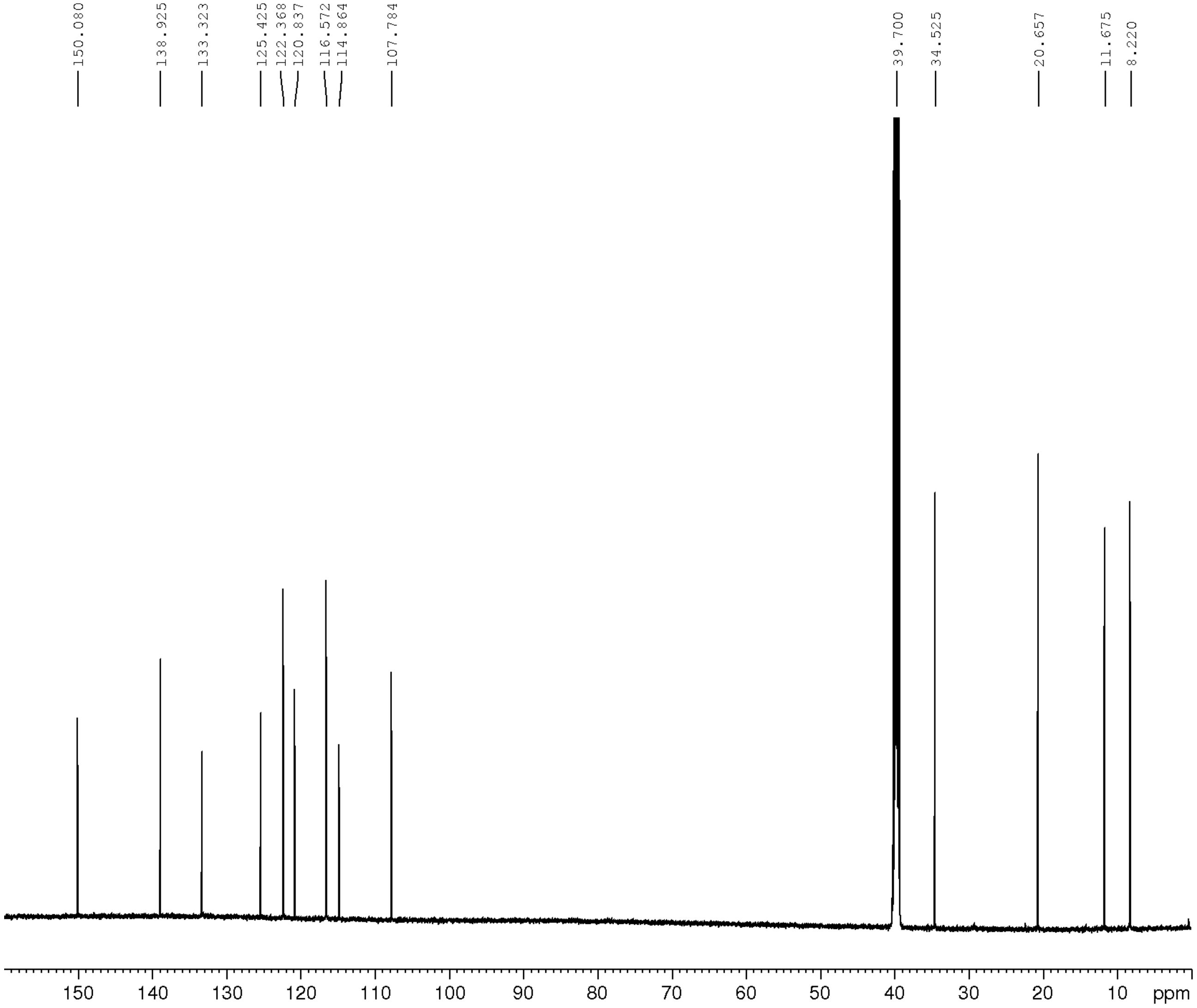

Standard 13C
142718
PGY0685_1A
Pusztai Gyongyver
2024.04.29. (KP)

Current Data Parameters

NAME	142718	
EXPNO	12	
PROCNO	1	

F2 - Acquisition Parameters

Date_	20240429	
Time	21.54	h
INSTRUM	spect	
PROBHD	Z145856_0002 (	
PULPROG	zgpg30	
TD	65536	
SOLVENT	DMSO	
NS	2048	
DS	4	
SWH	36231.883	Hz
FIDRES	1.105709	Hz
AQ	0.9043968	sec
RG	196.07	
DW	13.800	usec
DE	18.00	usec
TE	295.0	K
D1	1.00000000	sec
D11	0.03000000	sec
TD0	1	
SFO1	150.8852070	MHz
NUC1	13C	
P1	9.90	usec
PLW1	71.00000000	W
SFO2	600.0024000	MHz
NUC2	1H	
CPDPRG[2	waltz16	
PCPD2	80.00	usec
PLW2	32.90000153	W
PLW12	0.70370001	W
PLW13	0.35339001	W

F2 - Processing parameters

SI	32768	
SF	150.8701591	MHz
WDW	EM	
SSB	0	
LB	1.00	Hz
GB	0	
PC	1.40	

IR spectrum of **3a**

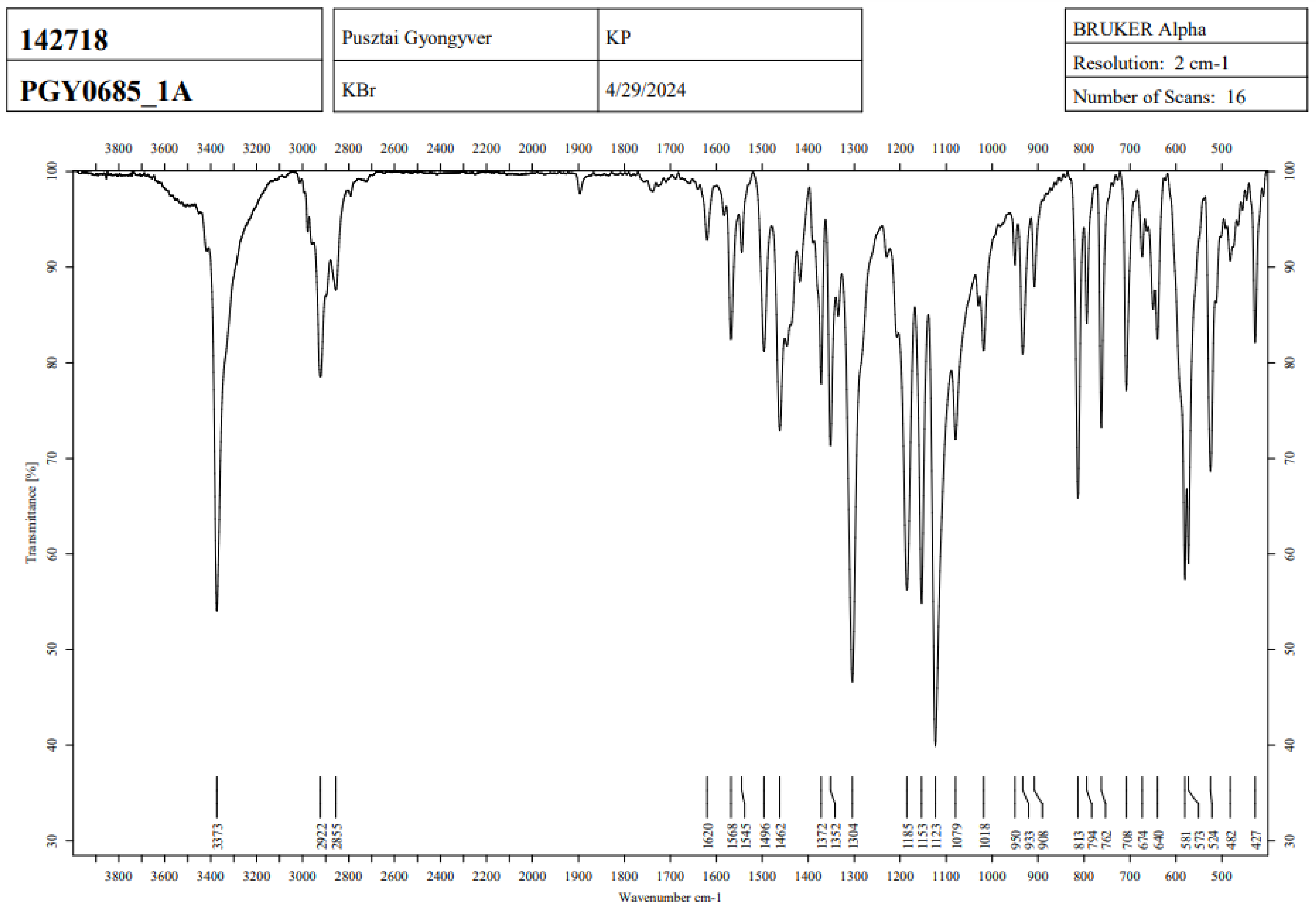

HRMS spectrum of **3a**

Spectrum Plot Report

Name	PGY0685_1A, Pusztai Gyongyver	Rack Pos.		Instrument	7250A with DIP	Operator	
Inj. Vol. (ul)	0.5	Plate Pos.		IRM Status	Success		
Data File	142718.D	Method (Acq)	DIP_70eV.M	Comment		Acq. Time (Local)	4/30/2024 11:53:20 AM (UTC+02:00)

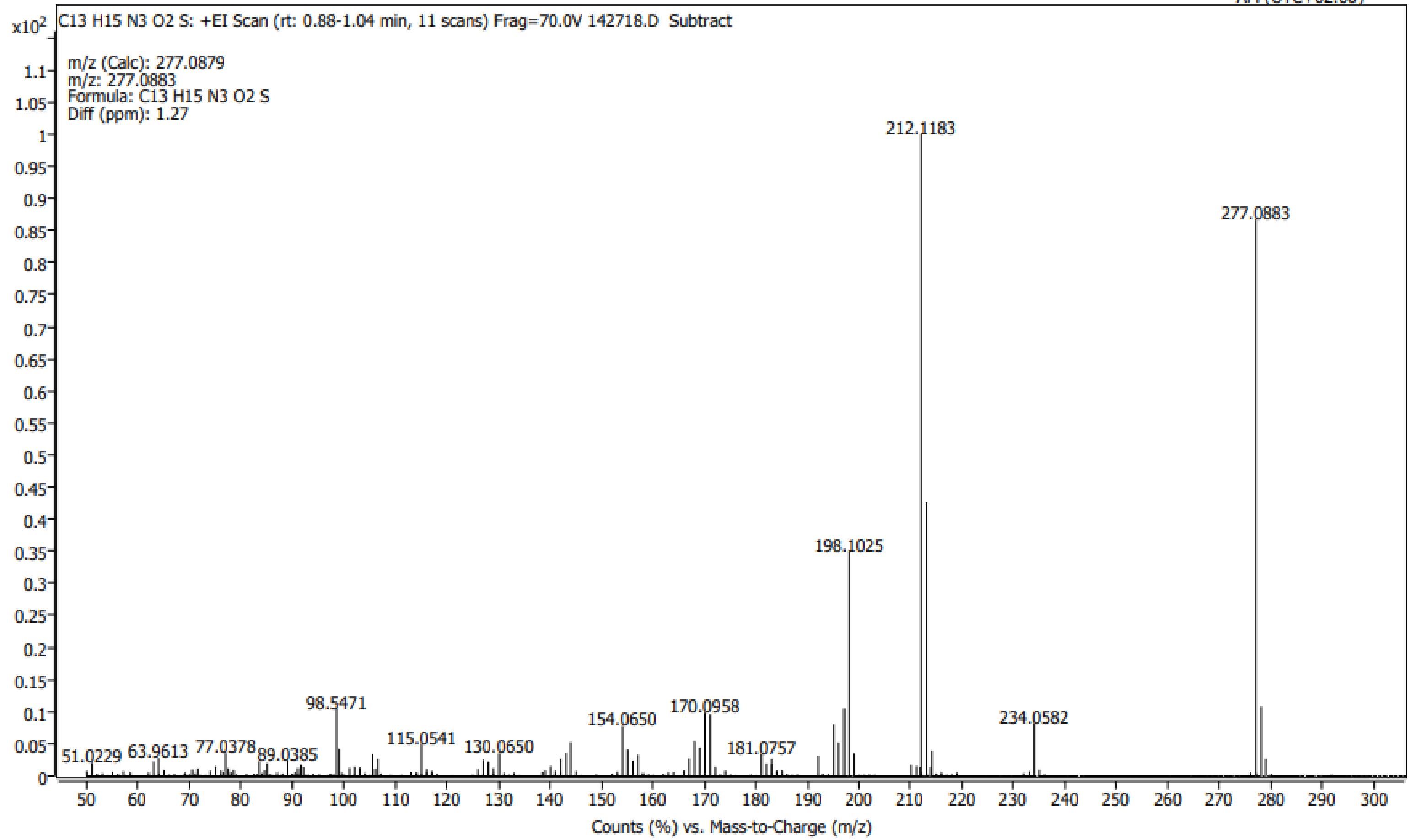

^{1}H NMR spectrum of **3b**

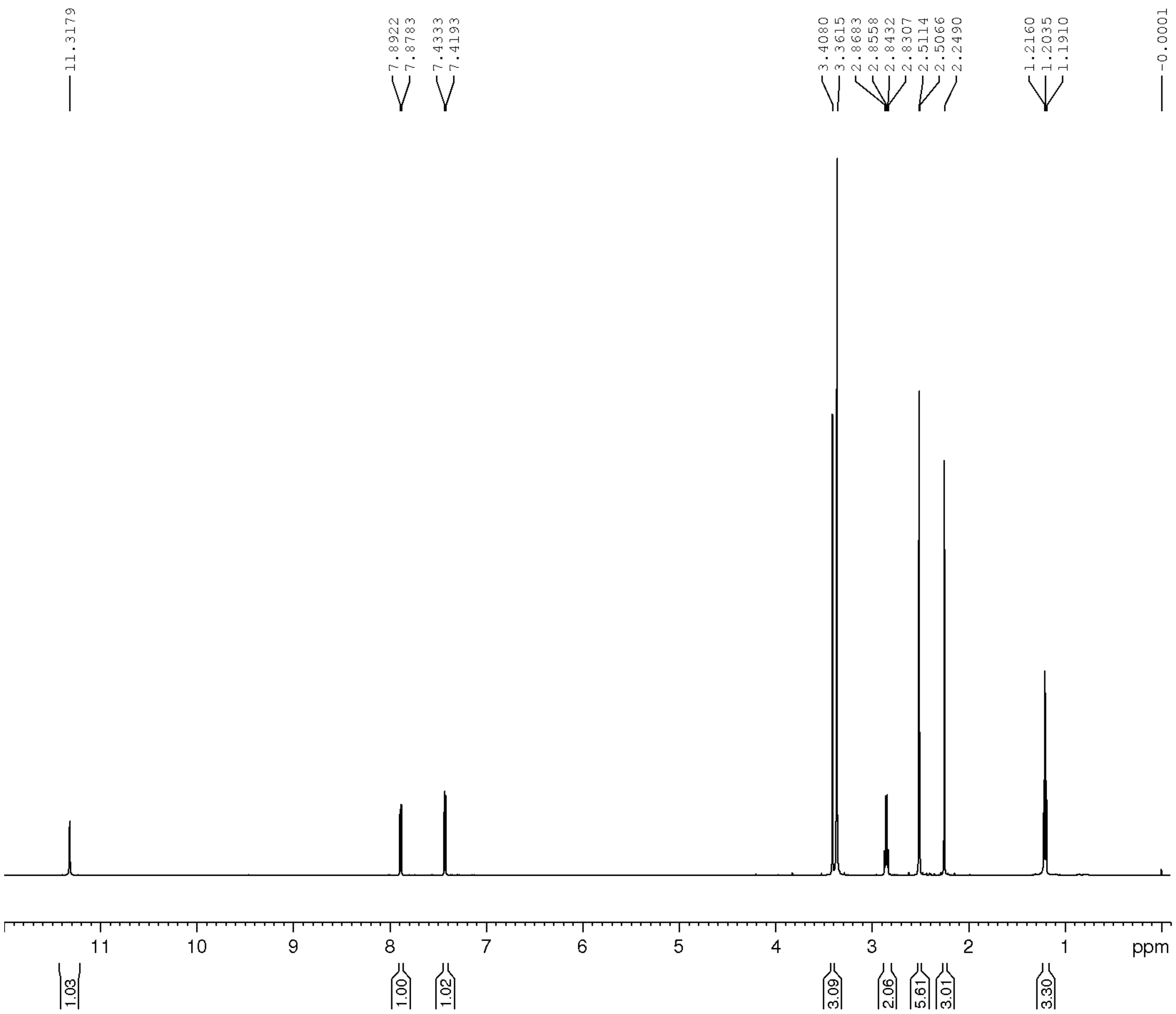

Standard 1H
142611
PGY0686_1B
Pusztai Gyongyver
2024.04.08. (KP)

Current Data Parameters
NAME 142611
EXPNO 21
PROCNO 1

F2 - Acquisition Parameters
Date_ 20240408
Time 16.49 h
INSTRUM spect
PROBHD Z145856_0002 (
PULPROG zg30
TD 65536
SOLVENT DMSO
NS 16
DS 2
SWH 12019.230 Hz
FIDRES 0.366798 Hz
AQ 2.7262976 sec
RG 196.07
DW 41.600 usec
DE 25.00 usec
TE 295.0 K
D1 1.00000000 sec
TD0 1
SFO1 600.0037050 MHz
NUC1 1H
P1 11.50 usec
PLW1 28.00000000 W

F2 - Processing parameters
SI 65536
SF 600.0000012 MHz
WDW EM
SSB 0
LB 0.30 Hz
GB 0
PC 1.00

^{13}C NMR spectrum of **3b**

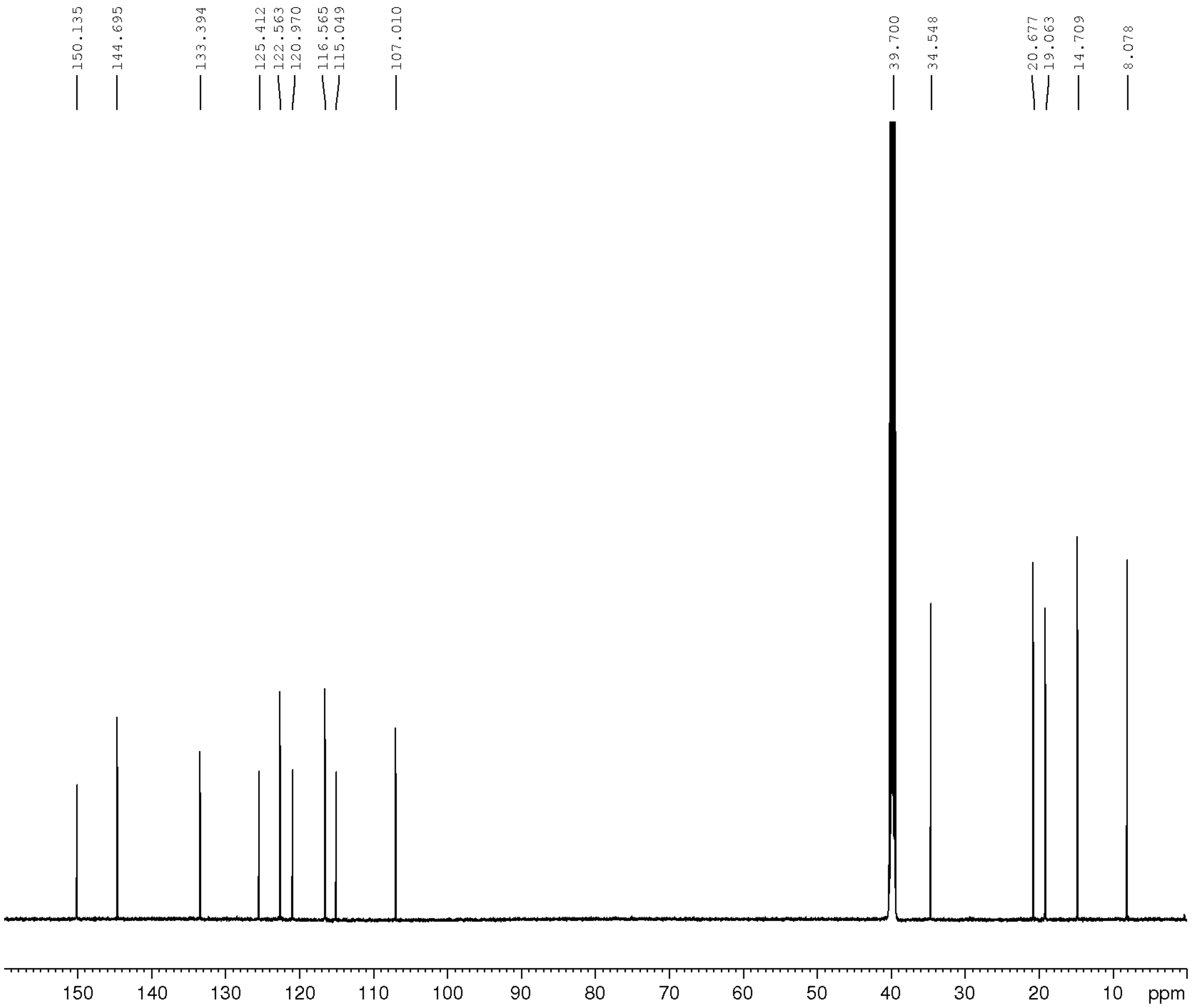

Standard 13C
142611
PGY0686_1B
Pusztai Gyongyver
2024.04.08. (KP)

Current Data Parameters

NAME	142611	
EXPNO	22	
PROCNO	1	

F2 - Acquisition Parameters

Date_	20240408	
Time	17.58	h
INSTRUM	spect	
PROBHD	Z145856_0002 (	
PULPROG	zgpg30	
TD	65536	
SOLVENT	DMSO	
NS	2048	
DS	4	
SWH	36231.883	Hz
FIDRES	1.105709	Hz
AQ	0.9043968	sec
RG	196.07	
DW	13.800	usec
DE	18.00	usec
TE	295.0	K
D1	1.00000000	sec
D11	0.03000000	sec
TD0	1	
SFO1	150.8852070	MHz
NUC1	13C	
P1	9.90	usec
PLW1	71.00000000	W
SFO2	600.0024000	MHz
NUC2	1H	
CPDPRG[2	waltz16	
PCPD2	80.00	usec
PLW2	32.90000153	W
PLW12	0.70370001	W
PLW13	0.35339001	W

F2 - Processing parameters

SI	32768	
SF	150.8701576	MHz
WDW	EM	
SSB	0	
LB	1.00	Hz
GB	0	
PC	1.40	

BRUKER

IR spectrum of **3b**

**142611**	Pusztai Gyongyver	KP
**PGY0686_1B**	KBr	4/8/2024

BRUKER Alpha
Resolution: 2 cm-1
Number of Scans: 16

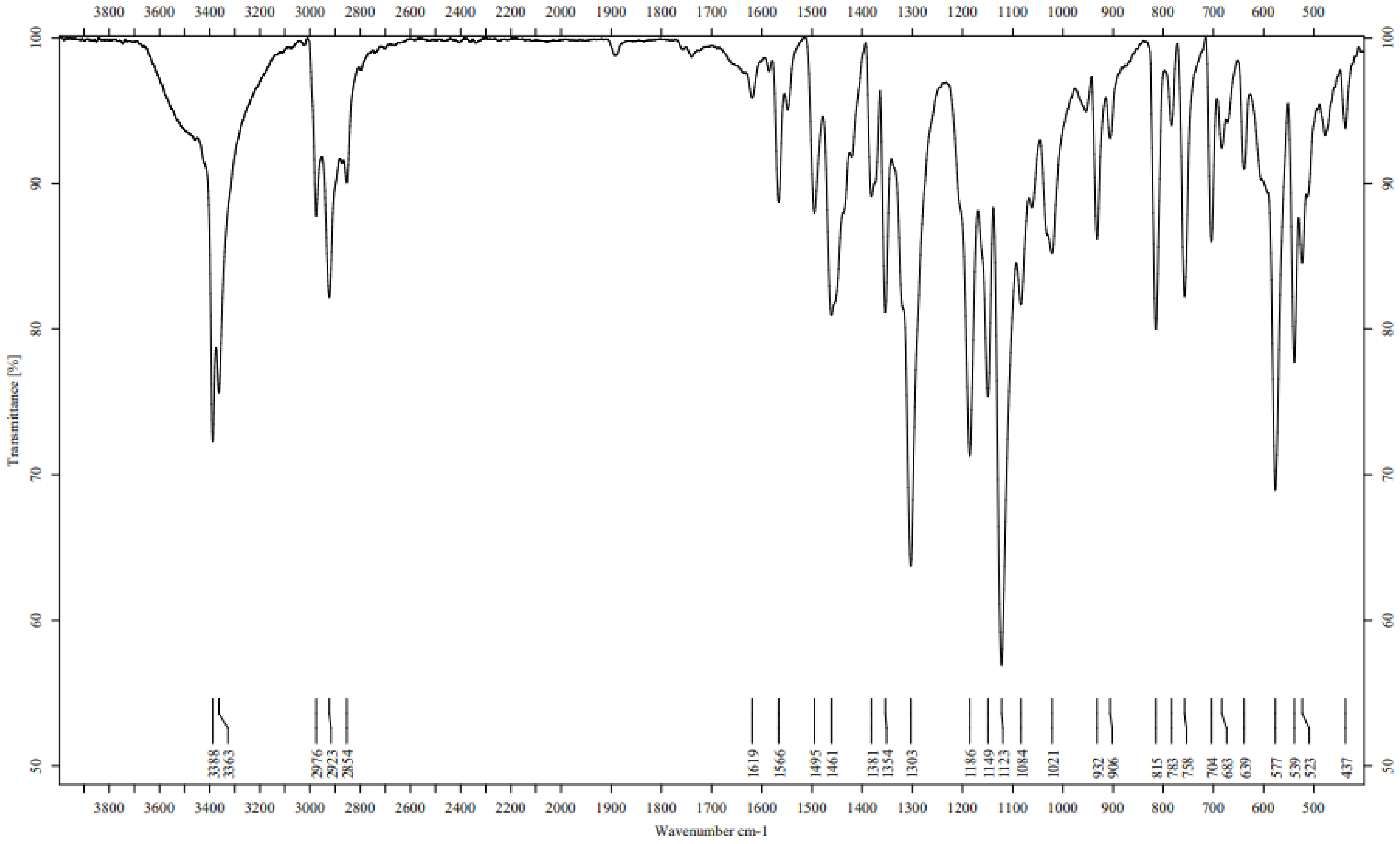

HRMS spectrum of **3b**

Sample: PGY00686_1B	Lab code: Nsz - 28962
Submitter: Pusztai Gyongyve	Project: Other

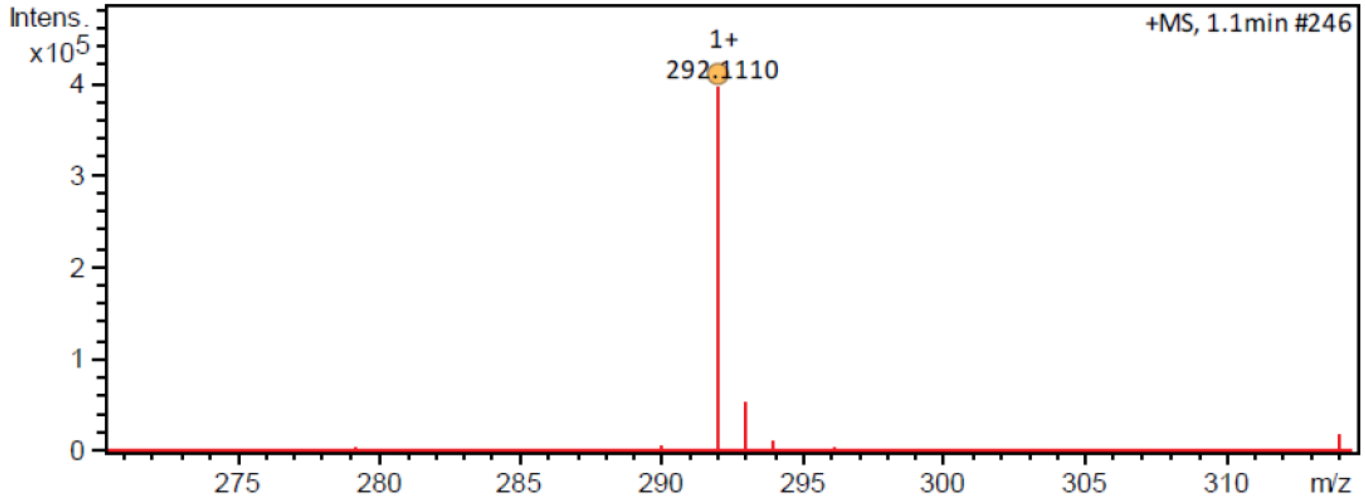

Meas. m/z	m/z	Ion Formula	err [ppm]
292.1110	292.1120	C14H18N3O2S	1.6

^{1}H NMR spectrum of **3c**

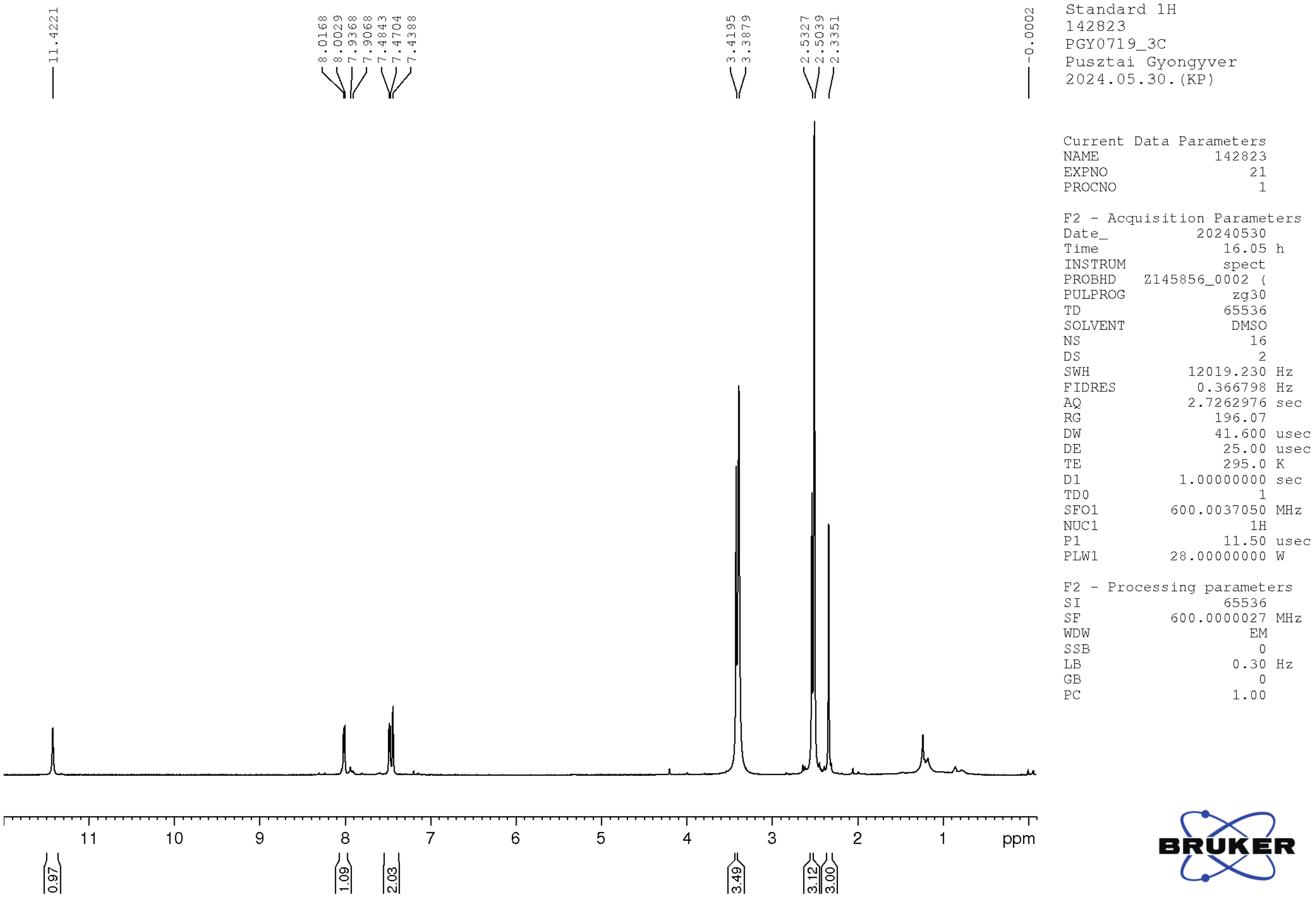

^{13}C NMR spectrum of **3c**

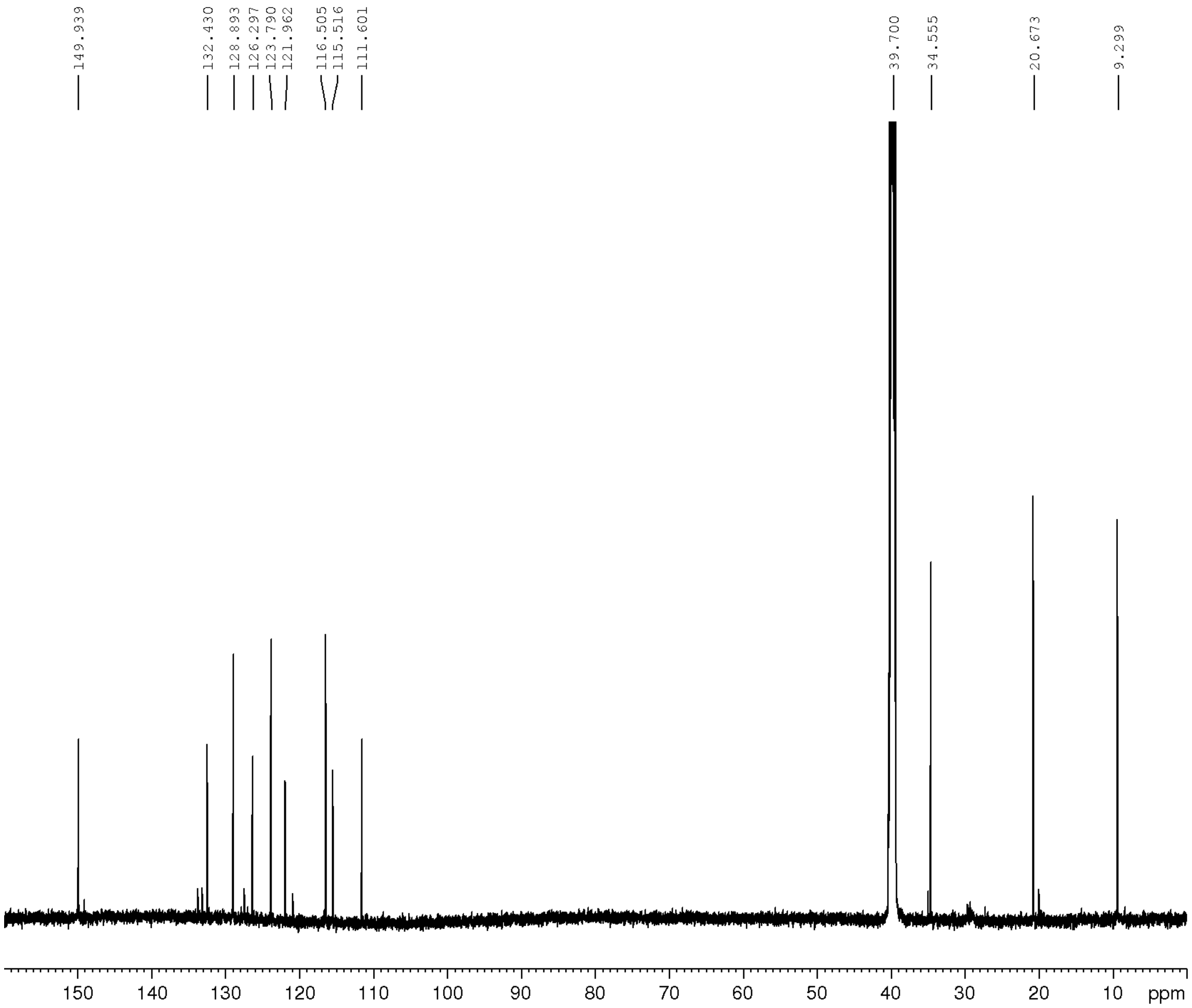

Standard 13C
142823
PGY0719_3C
Pusztai Gyongyver
2024.05.30.(KP)

Current Data Parameters

NAME	142823	
EXPNO	22	
PROCNO	1	

F2 - Acquisition Parameters

Date_	20240530	
Time	17.15	h
INSTRUM	spect	
PROBHD	Z145856_0002 (	
PULPROG	zgpg30	
TD	65536	
SOLVENT	DMSO	
NS	2048	
DS	4	
SWH	36231.883	Hz
FIDRES	1.105709	Hz
AQ	0.9043968	sec
RG	196.07	
DW	13.800	usec
DE	18.00	usec
TE	295.0	K
D1	1.00000000	sec
D11	0.03000000	sec
TD0	1	
SFO1	150.8852070	MHz
NUC1	13C	
P1	9.90	usec
PLW1	71.00000000	W
SFO2	600.0024000	MHz
NUC2	1H	
CPDPRG[2	waltz16	
PCPD2	80.00	usec
PLW2	32.90000153	W
PLW12	0.70370001	W
PLW13	0.35339001	W

F2 - Processing parameters

SI	32768	
SF	150.8701592	MHz
WDW	EM	
SSB	0	
LB	1.00	Hz
GB	0	
PC	1.40	

IR spectrum of **3c**

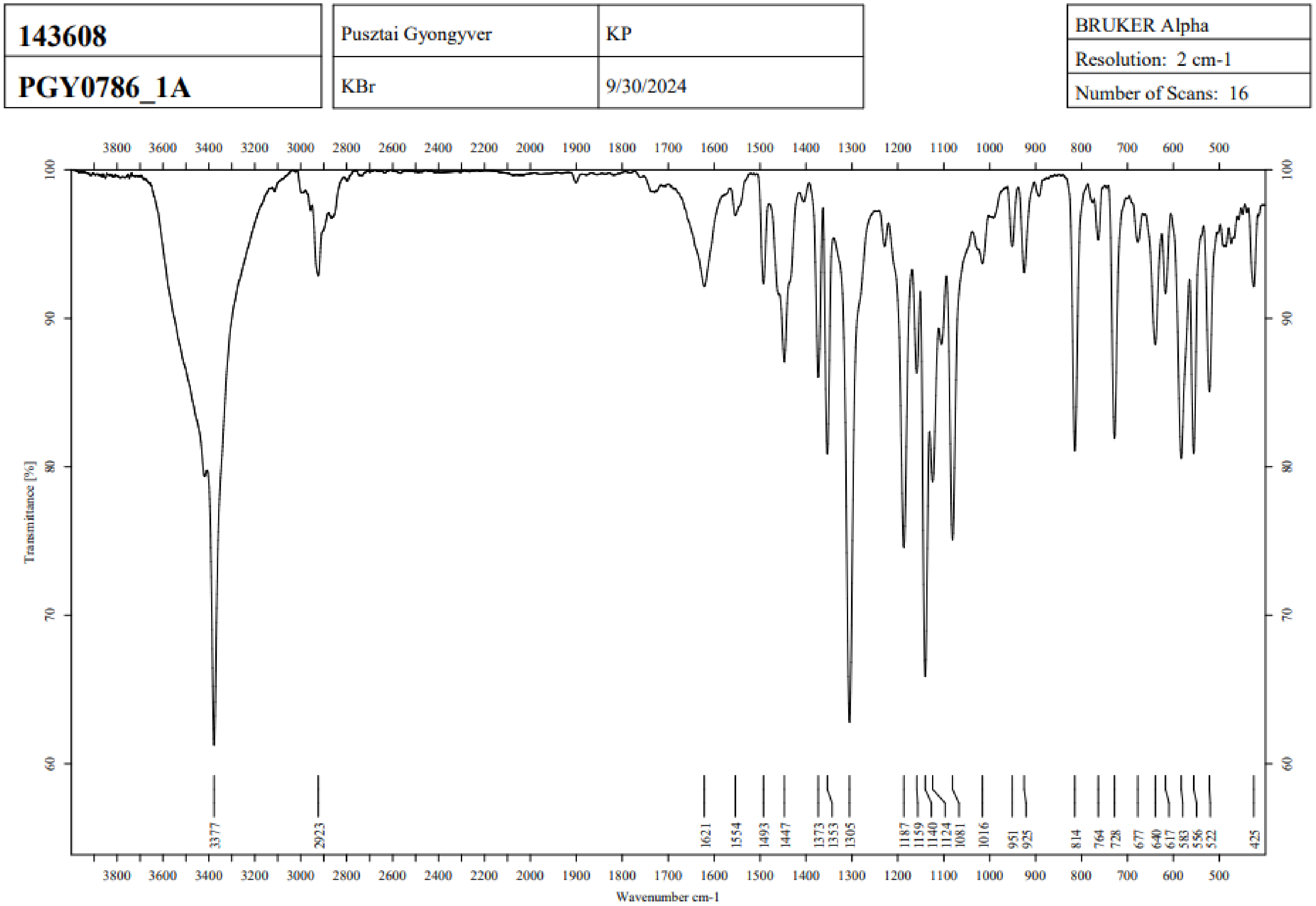

HRMS spectrum of **3c**

Spectrum Plot Report

Name	PGY0719_3C	Rack Pos.		Instrument	7250A with DIP	Operator	MM
Inj. Vol. (ul)	0.5	Plate Pos.		IRM Status	Success		
Data File	142823.D	Method (Acq)	DIP_70eV.M	Comment		Acq. Time (Local)	5/22/2024 2:18:41 PM (UTC+02:00)

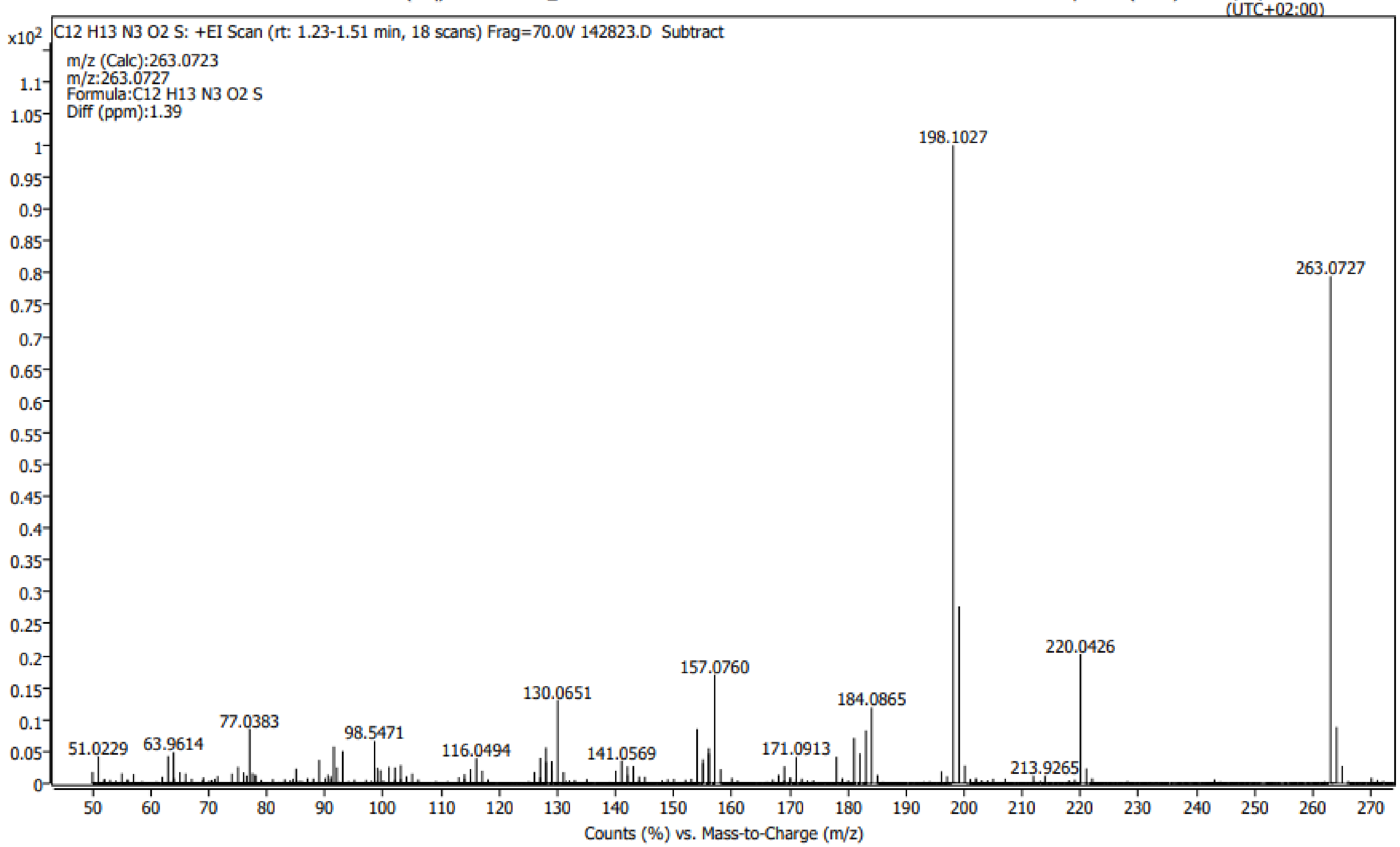

^{1}H NMR spectrum of **3d**

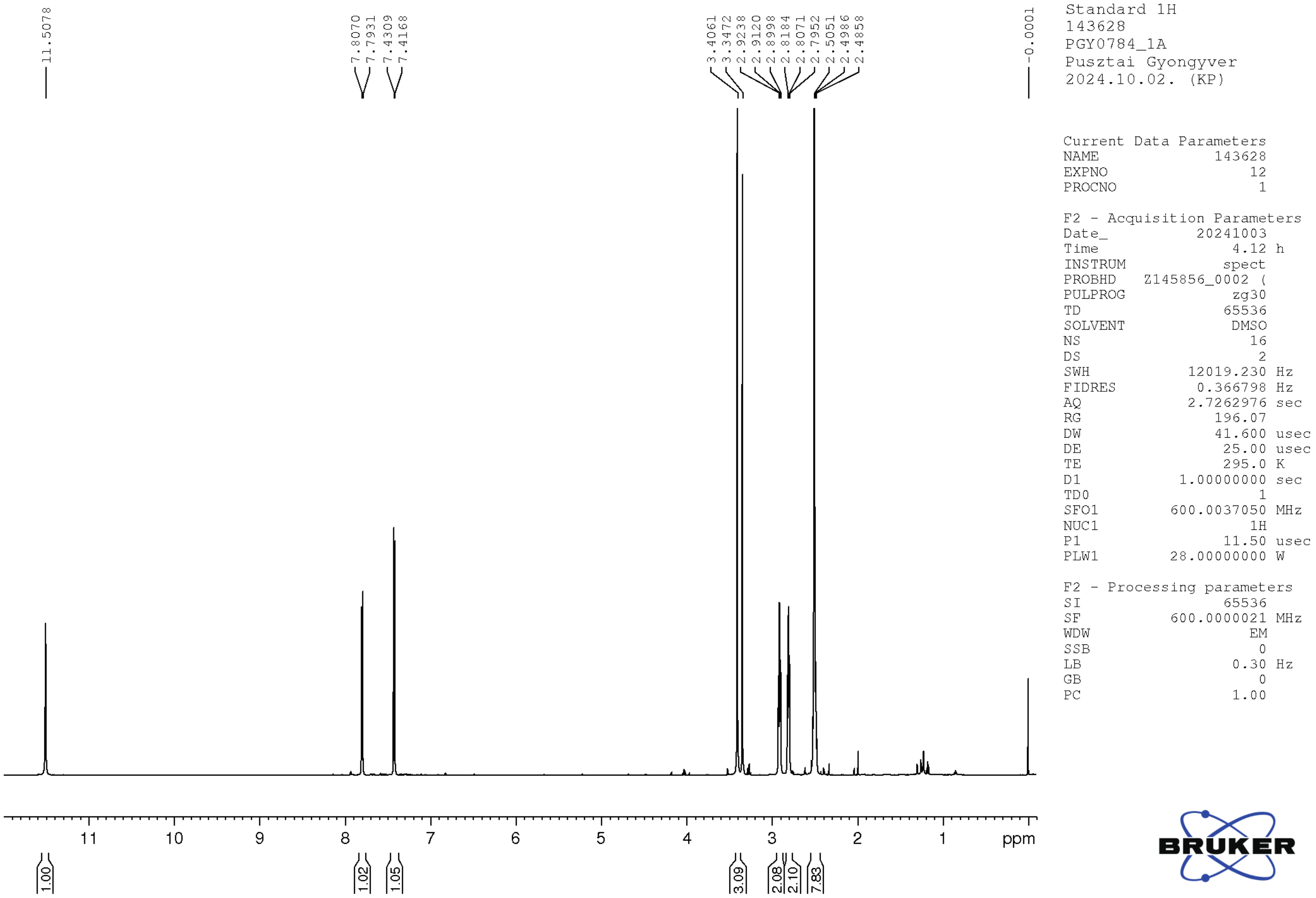

^{13}C NMR spectrum of **3d**

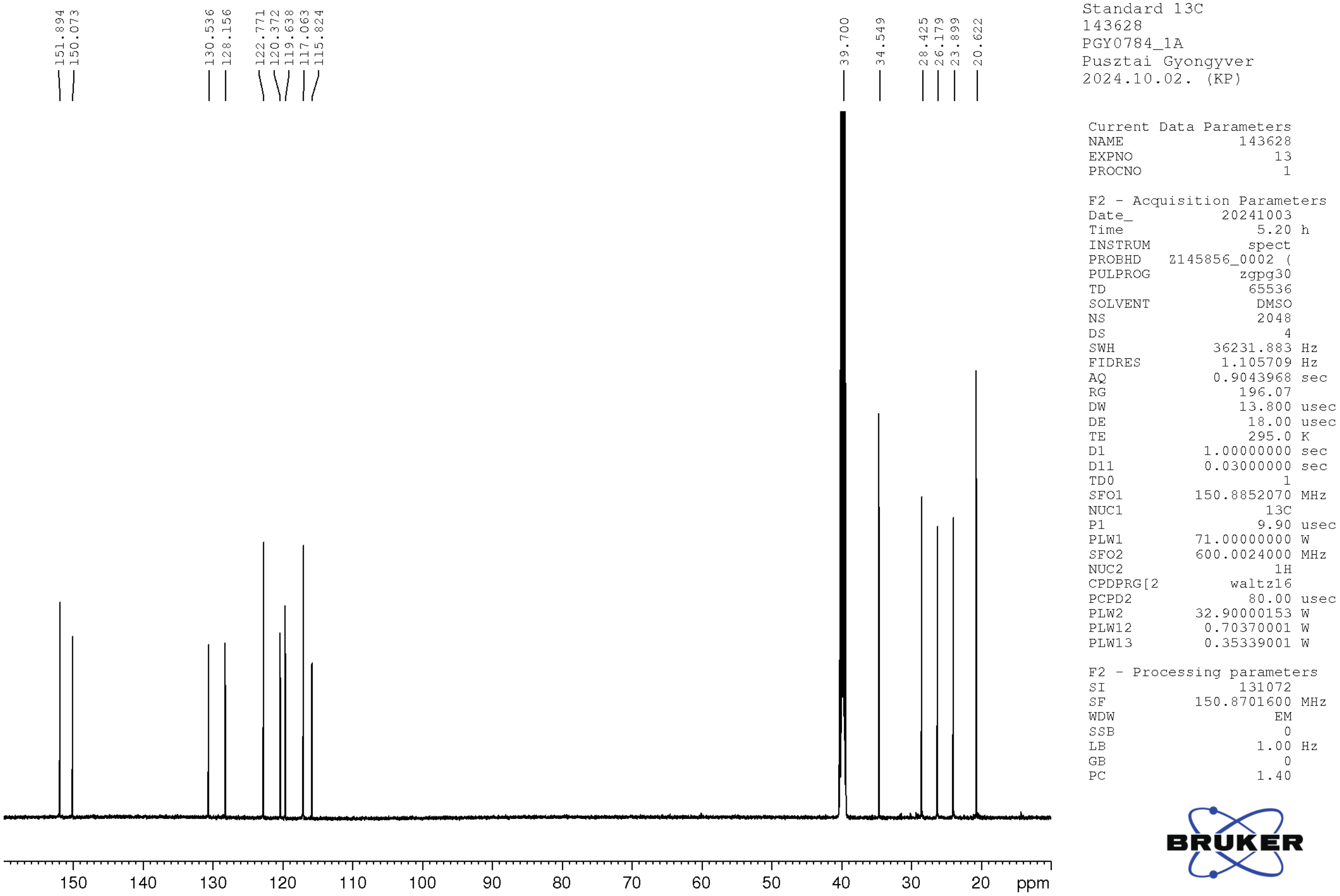

IR spectrum of **3d**

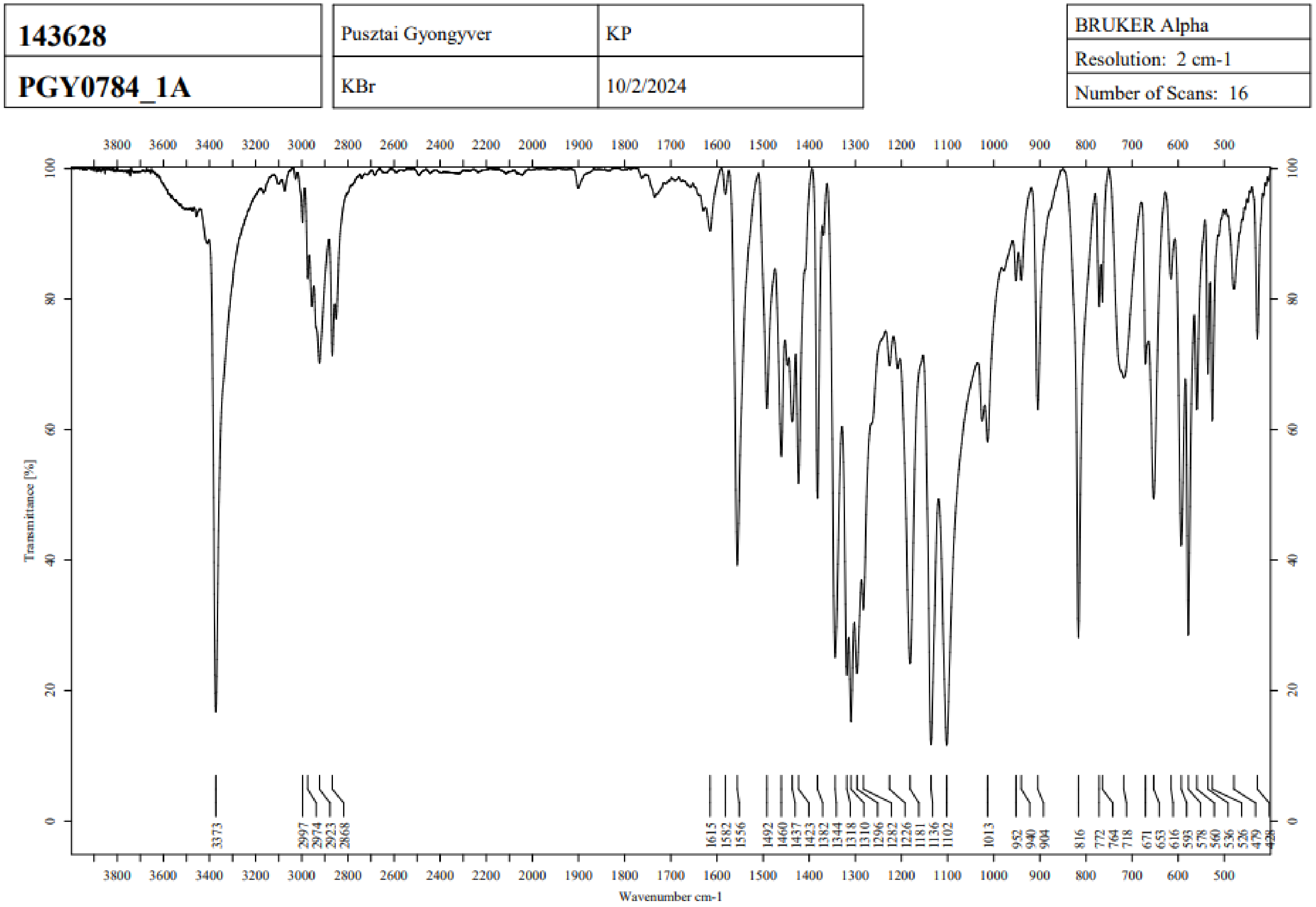

HRMS spectrum of **3d**

Spectrum Plot Report

Name	PGY0784_1A, Pusztai Gyongyver	Rack Pos.		Instrument	7250A with DIP	Operator	MM
Inj. Vol. (ul)	0.5	Plate Pos.		IRM Status	Success		
Data File	143628msaqtof_dip2.D	Method (Acq)	DIP_70eV_4min.M	Comment		Acq. Time (Local)	10/24/2024 1:31:58 PM (UTC+02:00)

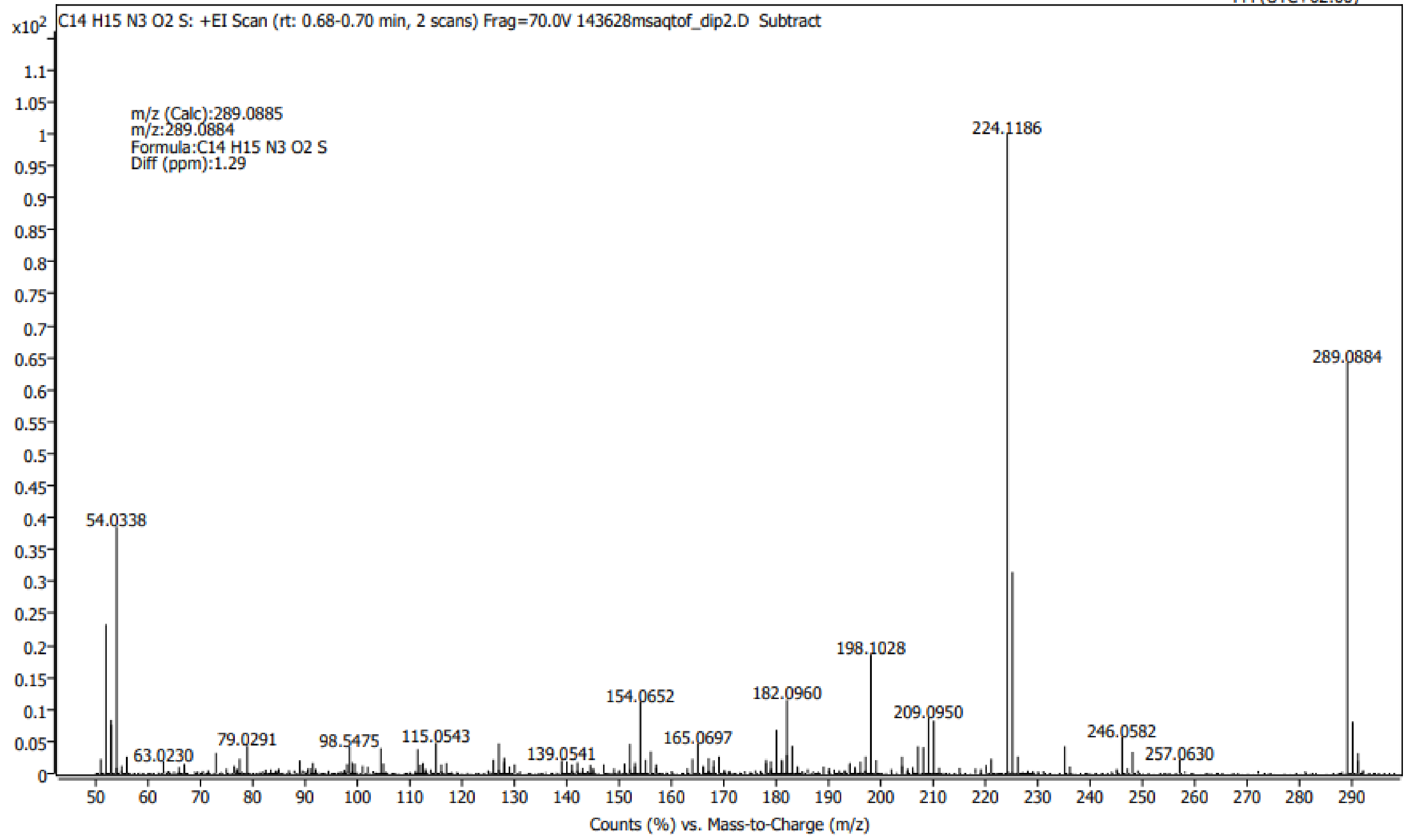

^{1}H NMR spectrum of **3f**

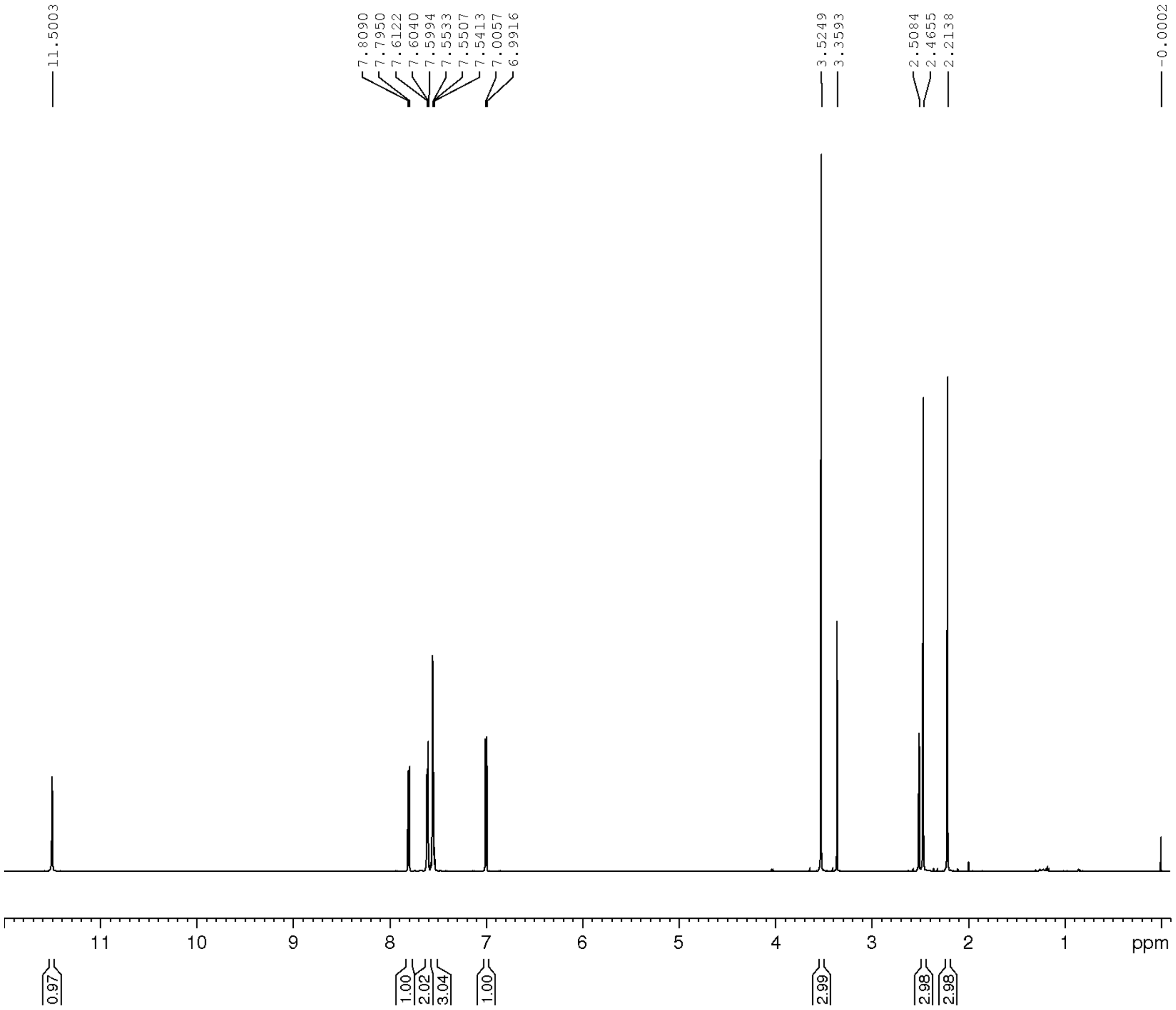

Standard 1H
143417
PGY0766_1A
Pusztai Gyongyver
2024.09.11. (KP)

Current Data Parameters
NAME 143417
EXPNO 21
PROCNO 1

F2 - Acquisition Parameters
Date_ 20240911
Time 22.21 h
INSTRUM spect
PROBHD Z145856_0002 (
PULPROG zg30
TD 65536
SOLVENT DMSO
NS 16
DS 2
SWH 12019.230 Hz
FIDRES 0.366798 Hz
AQ 2.7262976 sec
RG 196.07
DW 41.600 usec
DE 25.00 usec
TE 295.0 K
D1 1.00000000 sec
TD0 1
SFO1 600.0037050 MHz
NUC1 1H
P1 11.50 usec
PLW1 28.00000000 W

F2 - Processing parameters
SI 65536
SF 599.9999998 MHz
WDW EM
SSB 0
LB 0.30 Hz
GB 0
PC 1.00

^{13}C NMR spectrum of **3f**

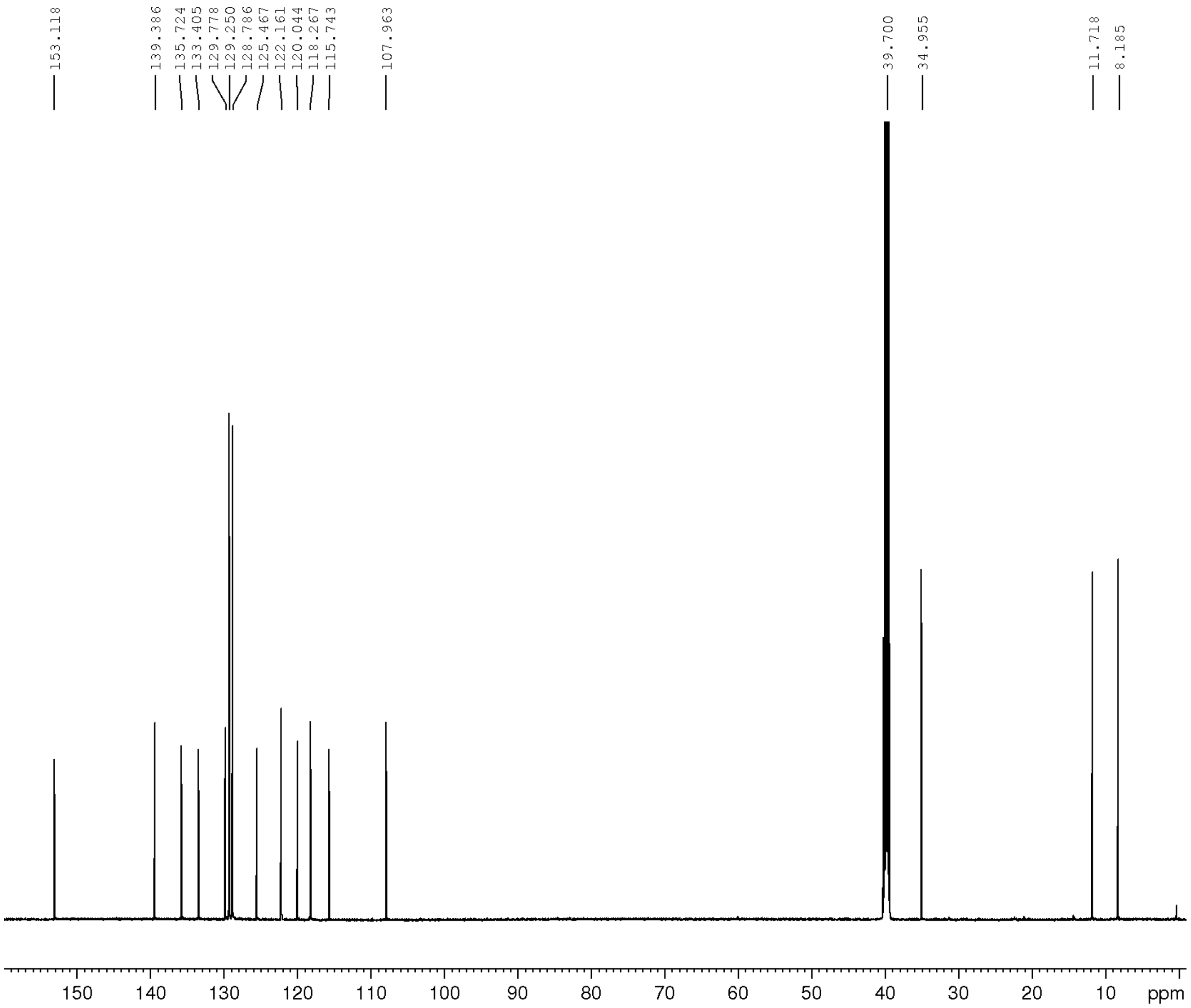

Standard 13C
143417
PGY0766_1A
Pusztai Gyongyver
2024.09.11. (KP)

Current Data Parameters	
NAME	143417
EXPNO	22
PROCNO	1

F2 - Acquisition Parameters	
Date_	20240911
Time	23.30 h
INSTRUM	spect
PROBHD	Z145856_0002 (
PULPROG	zgpg30
TD	65536
SOLVENT	DMSO
NS	2048
DS	4
SWH	36231.883 Hz
FIDRES	1.105709 Hz
AQ	0.9043968 sec
RG	196.07
DW	13.800 usec
DE	18.00 usec
TE	295.0 K
D1	1.00000000 sec
D11	0.03000000 sec
TD0	1
SFO1	150.8852070 MHz
NUC1	13C
P1	9.90 usec
PLW1	71.00000000 W
SFO2	600.0024000 MHz
NUC2	1H
CPDPRG[2	waltz16
PCPD2	80.00 usec
PLW2	32.90000153 W
PLW12	0.70370001 W
PLW13	0.35339001 W

F2 - Processing parameters	
SI	131072
SF	150.8701601 MHz
WDW	EM
SSB	0
LB	1.00 Hz
GB	0
PC	1.40

IR spectrum of **3f**

**143417**	
**PGY0766_1A**	

Pusztai Gyongyver	KP
KBr	9/11/2024

BRUKER Alpha
Resolution: 2 cm-1
Number of Scans: 16

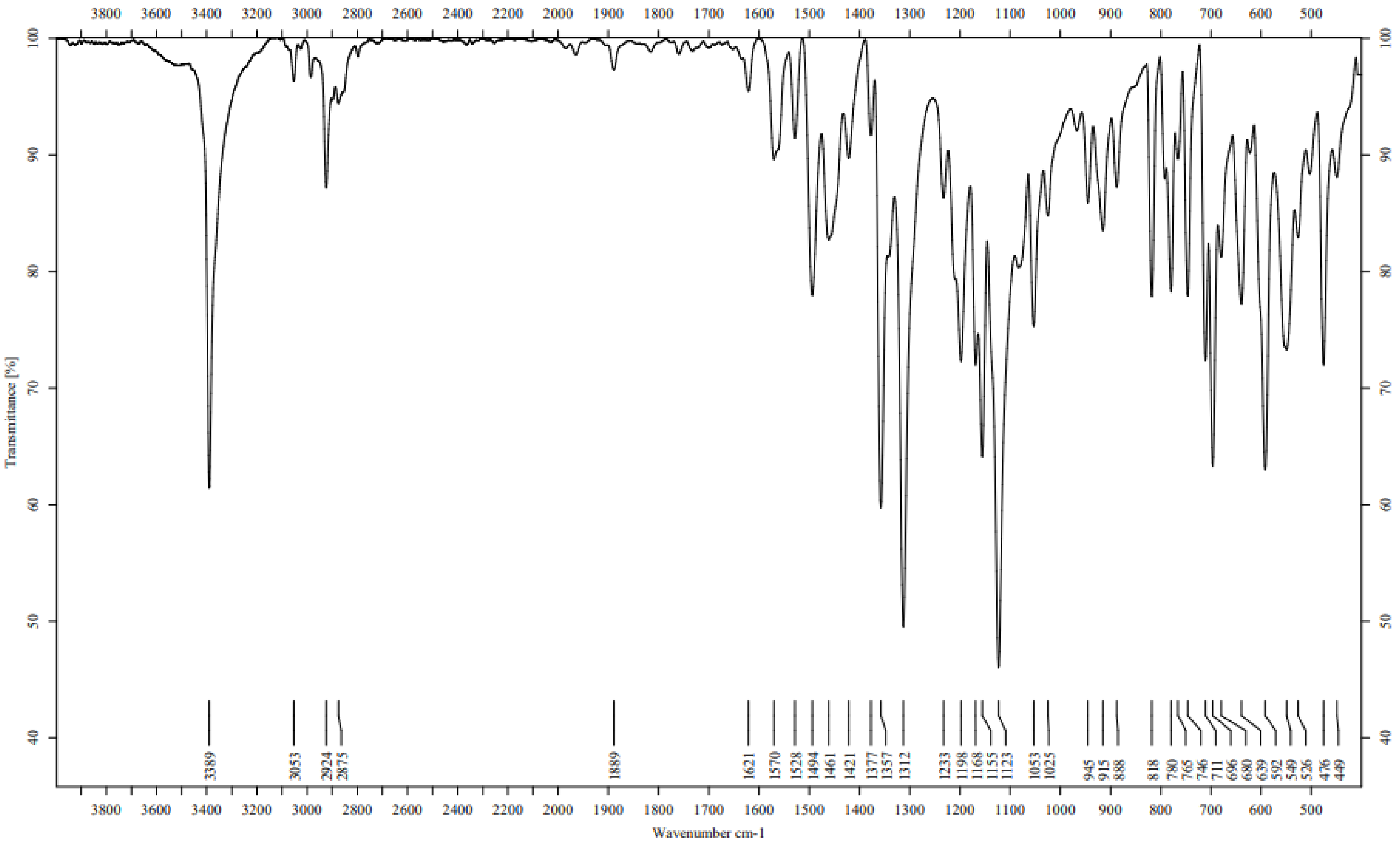

HRMS spectrum of **3f**

Spectrum Plot Report

Name	PGY0766_1A, Pusztai Gyongyver	Rack Pos.		Instrument	7250A with DIP	Operator	MM
Inj. Vol. (ul)	0.5	Plate Pos.		IRM Status	Success		
Data File	143417msqtof_dip.D	Method (Acq)	DIP_70eV.M	Comment		Acq. Time (Local)	9/18/2024 8:57:11 AM (UTC+02:00)

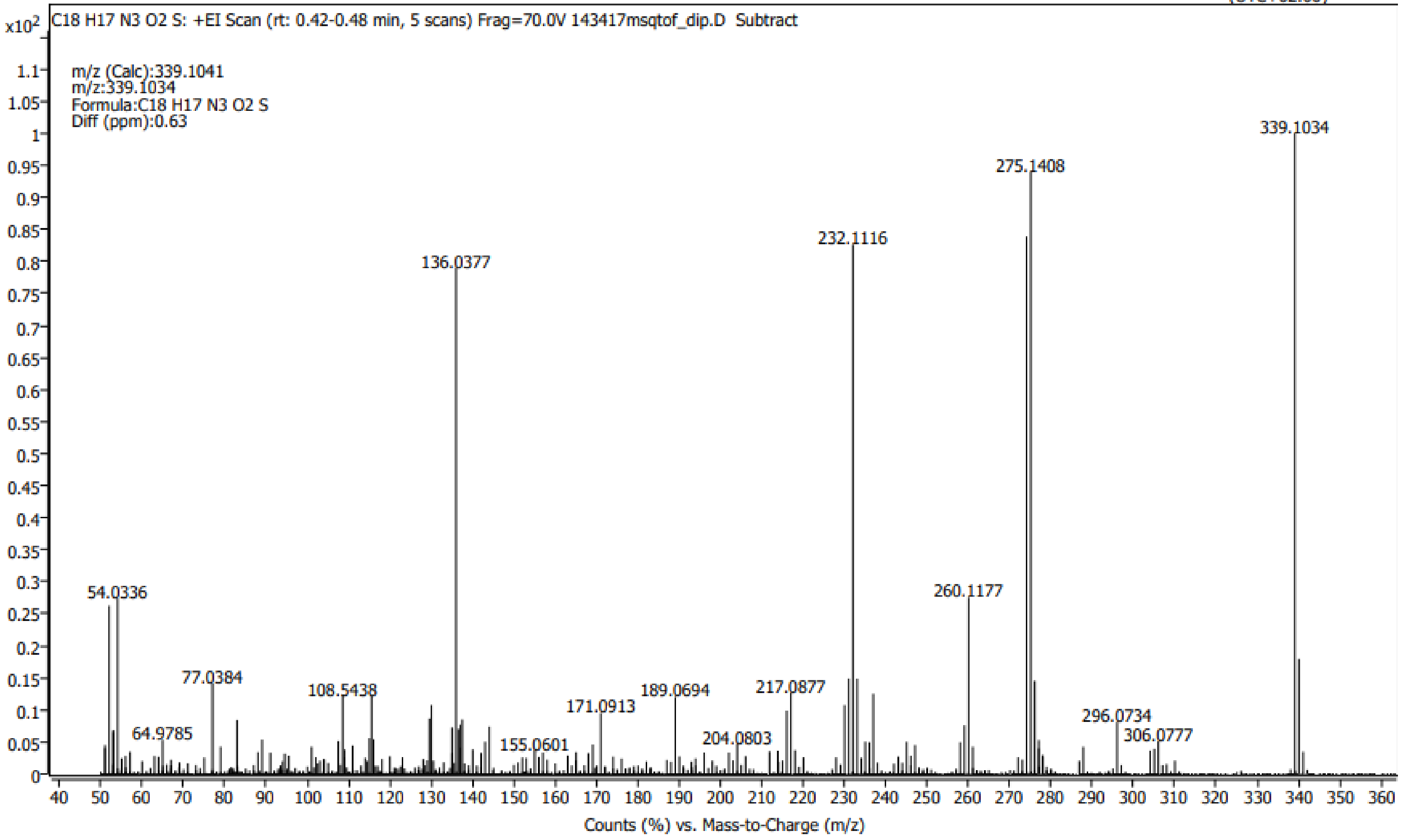

^{1}H NMR spectrum of **3g**

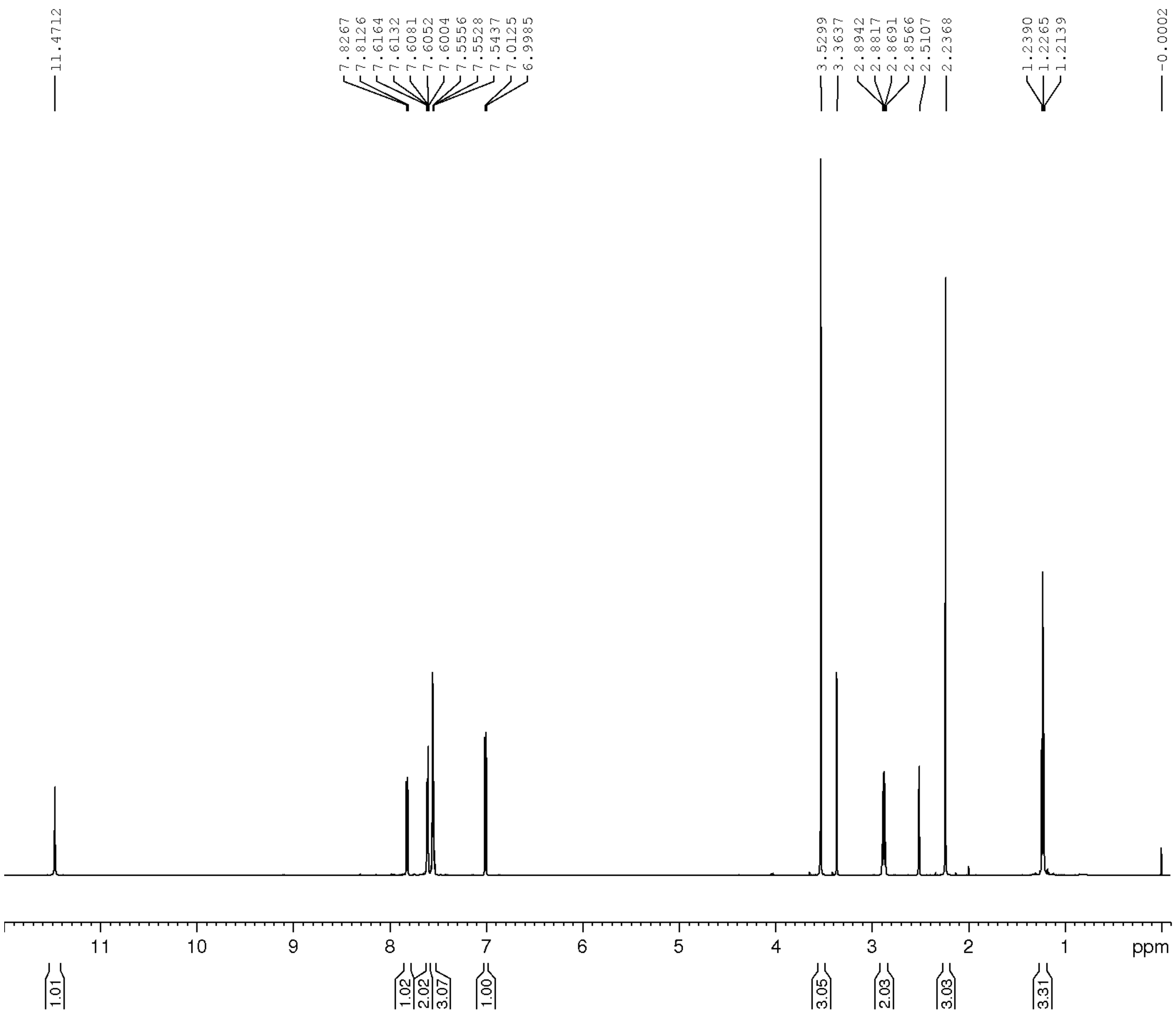

Standard 1H
143497
PGY0767_1A
Pusztai Gyongyver
2024.09.11. (KP)

Current Data Parameters

NAME	143497
EXPNO	11
PROCNO	1

F2 - Acquisition Parameters

Date_	20240912
Time	0.56 h
INSTRUM	spect
PROBHD	Z145856_0002 (
PULPROG	zg30
TD	65536
SOLVENT	DMSO
NS	16
DS	2
SWH	12019.230 Hz
FIDRES	0.366798 Hz
AQ	2.7262976 sec
RG	119.07
DW	41.600 usec
DE	25.00 usec
TE	295.0 K
D1	1.00000000 sec
TD0	1
SFO1	600.0037050 MHz
NUC1	1H
P1	11.50 usec
PLW1	28.00000000 W

F2 - Processing parameters

SI	65536
SF	599.9999984 MHz
WDW	EM
SSB	0
LB	0.30 Hz
GB	0
PC	1.00

^{13}C NMR spectrum of **3g**

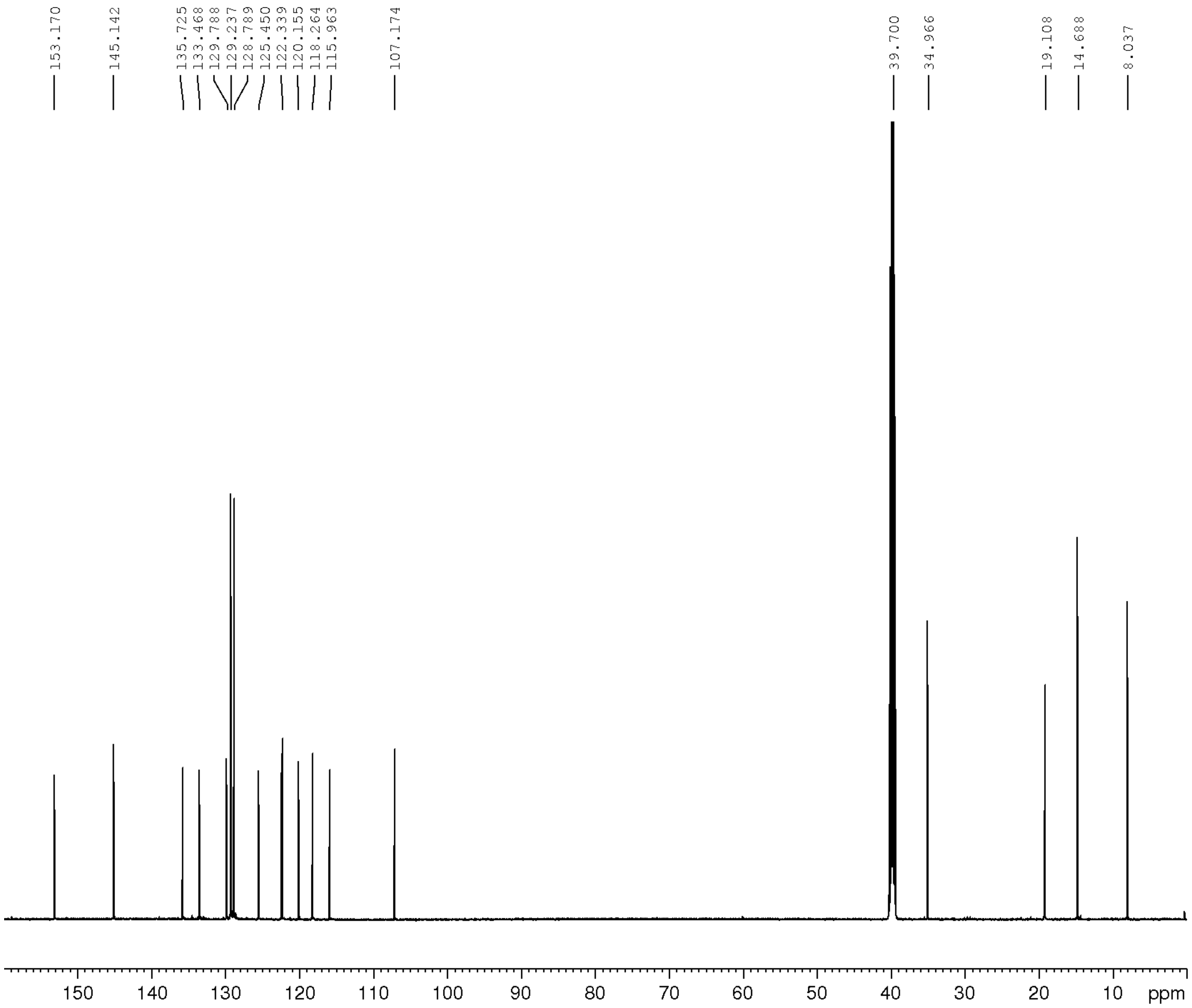

Standard 13C
143497
PGY0767_1A
Pusztai Gyongyver
2024.09.11. (KP)

Current Data Parameters

NAME	143497	
EXPNO	12	
PROCNO	1	

F2 - Acquisition Parameters

Date_	20240912	
Time	2.04	h
INSTRUM	spect	
PROBHD	Z145856_0002 (	
PULPROG	zgpg30	
TD	65536	
SOLVENT	DMSO	
NS	2048	
DS	4	
SWH	36231.883	Hz
FIDRES	1.105709	Hz
AQ	0.9043968	sec
RG	196.07	
DW	13.800	usec
DE	18.00	usec
TE	295.0	K
D1	1.00000000	sec
D11	0.03000000	sec
TD0	1	
SFO1	150.8852070	MHz
NUC1	13C	
P1	9.90	usec
PLW1	71.00000000	W
SFO2	600.0024000	MHz
NUC2	1H	
CPDPRG[2	waltz16	
PCPD2	80.00	usec
PLW2	32.90000153	W
PLW12	0.70370001	W
PLW13	0.35339001	W

F2 - Processing parameters

SI	131072	
SF	150.8701600	MHz
WDW	EM	
SSB	0	
LB	1.00	Hz
GB	0	
PC	1.40	

IR spectrum of **3g**

**143497**	
**PGY0767_1A**	

Pusztai Gyongyver	KP
KBr	9/11/2024

BRUKER Alpha
Resolution: 2 cm-1
Number of Scans: 16

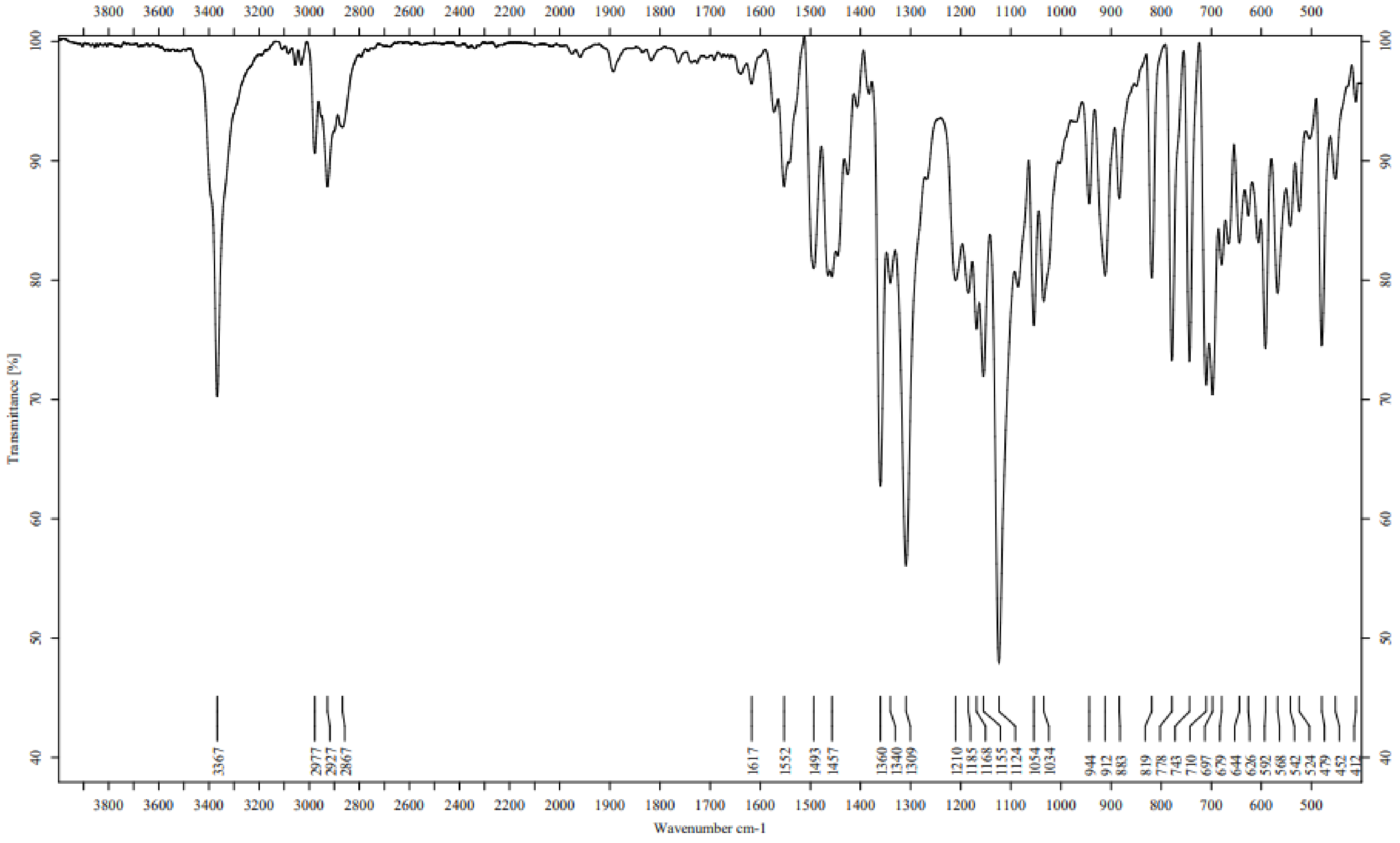

HRMS spectrum of **3g**

Spectrum Plot Report

Name	PGY0767_1A, Pusztai Gyongyver	Rack Pos.		Instrument	7250A with DIP	Operator	MM
Inj. Vol. (ul)	0.5	Plate Pos.		IRM Status	Success		
Data File	143497msqtof_dip.D	Method (Acq)	DIP_70eV.M	Comment		Acq. Time (Local)	9/18/2024 10:07:07 AM (UTC+02:00)

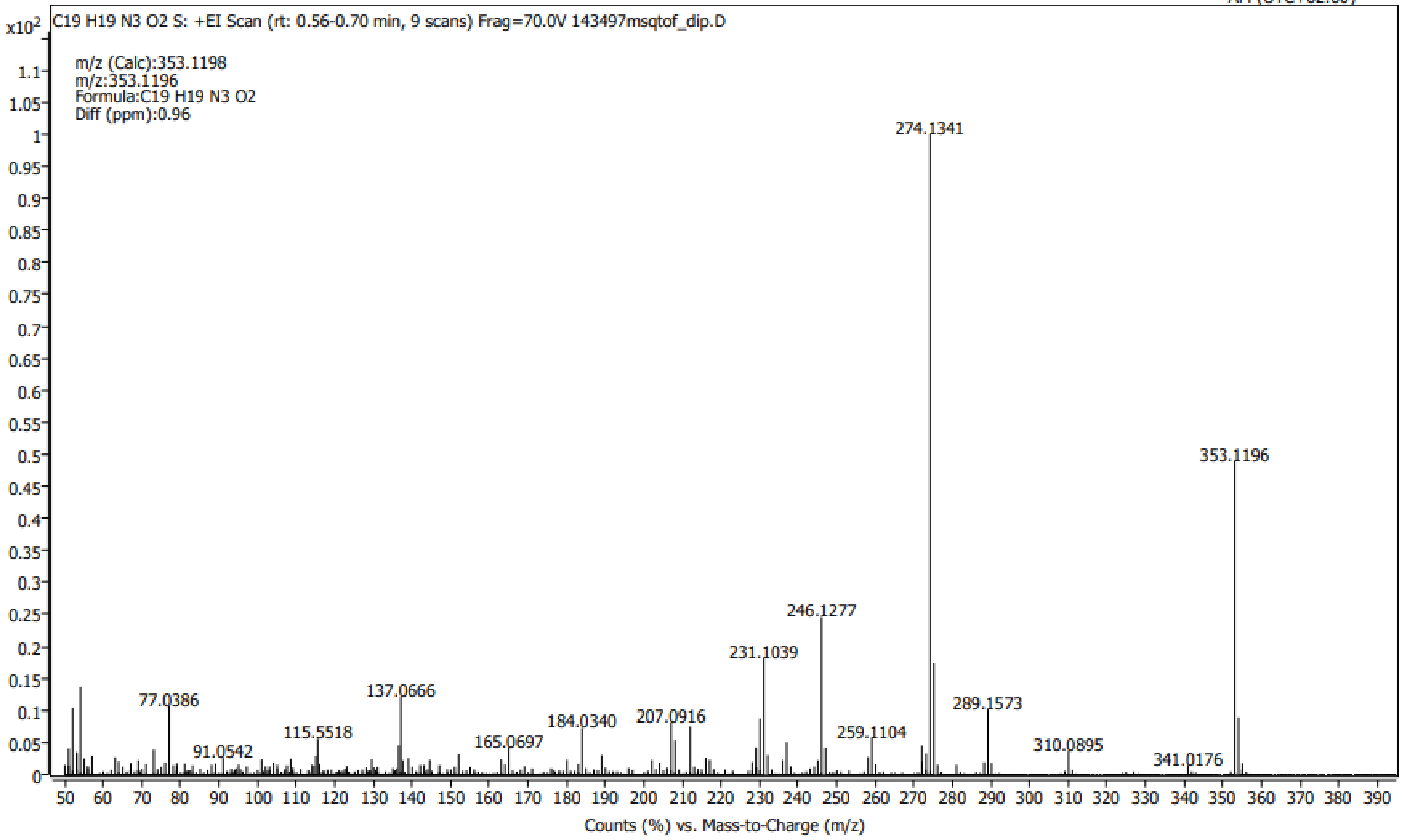

^{1}H NMR spectrum of **3i**

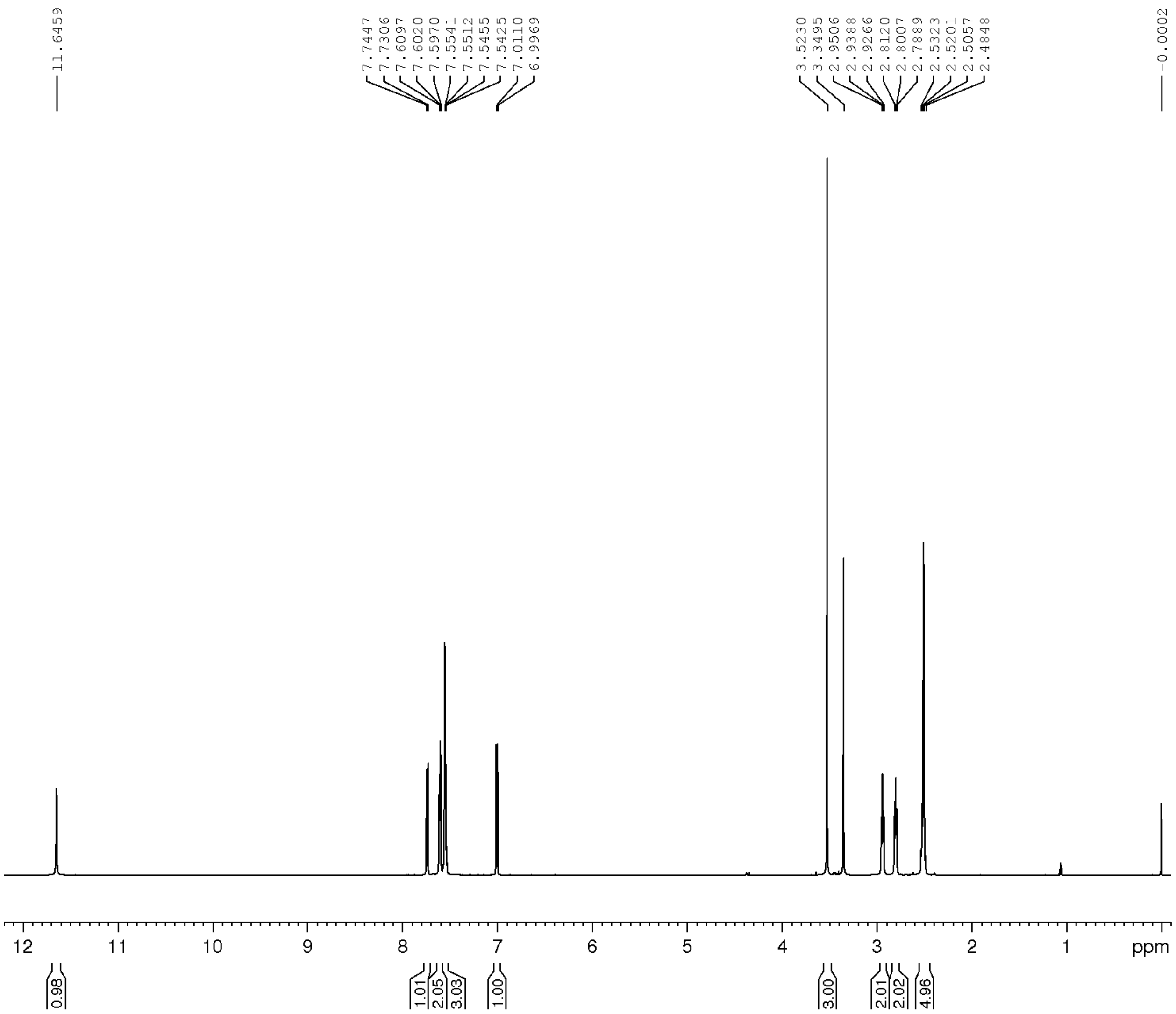

Standard 1H
143568
PGY0758_1C
Pusztai Gyongyver
2024.10.04. (KP)

Current Data Parameters
NAME 143568
EXPNO 12
PROCNO 1

F2 - Acquisition Parameters
Date_ 20241004
Time 22.39 h
INSTRUM spect
PROBHD Z145856_0002 (
PULPROG zg30
TD 65536
SOLVENT DMSO
NS 16
DS 2
SWH 12019.230 Hz
FIDRES 0.366798 Hz
AQ 2.7262976 sec
RG 196.07
DW 41.600 usec
DE 25.00 usec
TE 295.0 K
D1 1.00000000 sec
TD0 1
SFO1 600.0037050 MHz
NUC1 1H
P1 11.50 usec
PLW1 28.00000000 W

F2 - Processing parameters
SI 65536
SF 600.0000014 MHz
WDW EM
SSB 0
LB 0.30 Hz
GB 0
PC 1.00

^{13}C NMR spectrum of **3i**

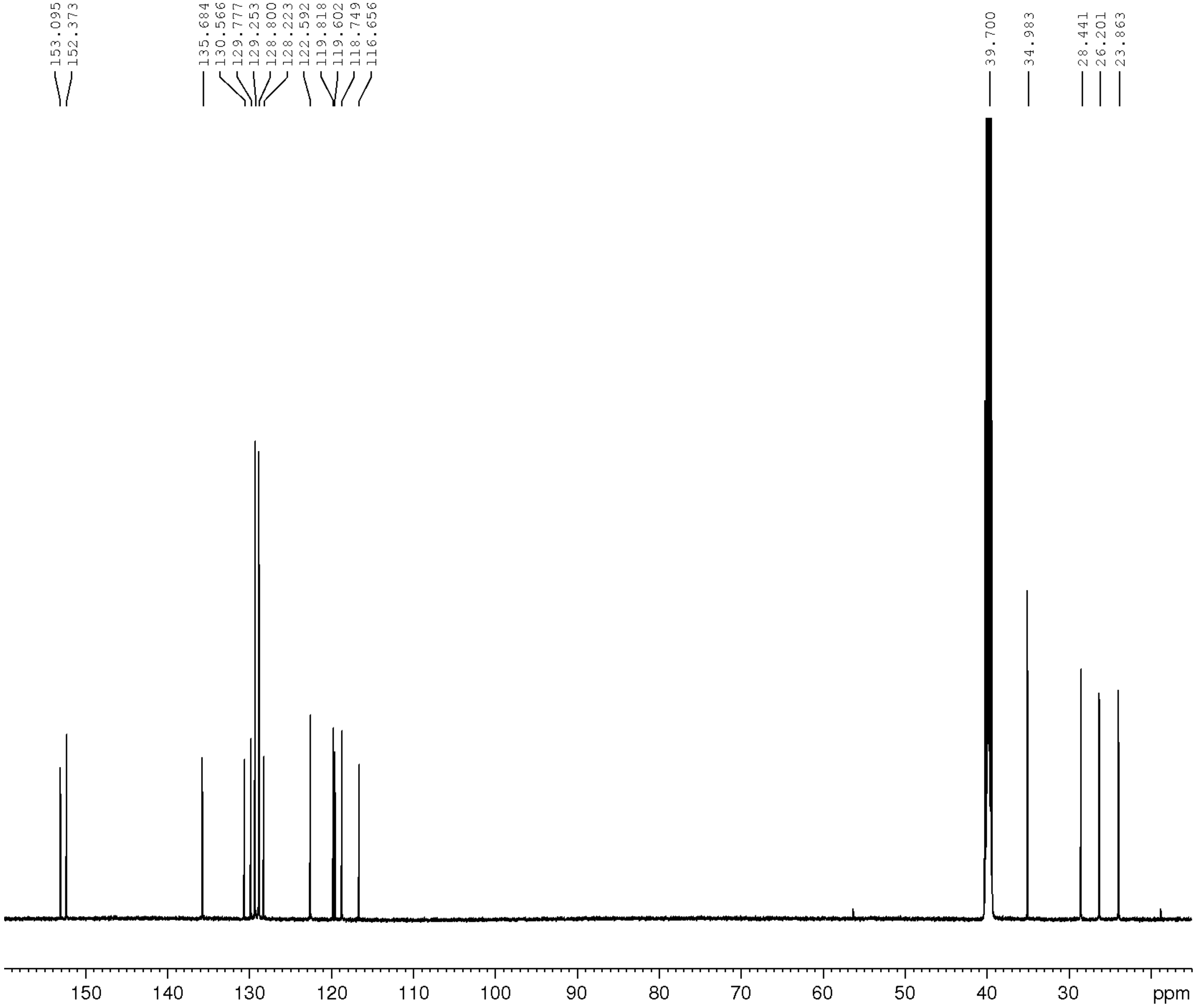

Standard 13C
143568
PGY0758_1C
Pusztai Gyongyver
2024.10.04. (KP)

Current Data Parameters

NAME	143568	
EXPNO	13	
PROCNO	1	

F2 - Acquisition Parameters

Date_	20241004	
Time	23.48	h
INSTRUM	spect	
PROBHD	Z145856_0002 (	
PULPROG	zgpg30	
TD	65536	
SOLVENT	DMSO	
NS	2048	
DS	4	
SWH	36231.883	Hz
FIDRES	1.105709	Hz
AQ	0.9043968	sec
RG	196.07	
DW	13.800	usec
DE	18.00	usec
TE	295.0	K
D1	1.00000000	sec
D11	0.03000000	sec
TD0	1	
SFO1	150.8852070	MHz
NUC1	13C	
P1	9.90	usec
PLW1	71.00000000	W
SFO2	600.0024000	MHz
NUC2	1H	
CPDPRG[2	waltz16	
PCPD2	80.00	usec
PLW2	32.90000153	W
PLW12	0.70370001	W
PLW13	0.35339001	W

F2 - Processing parameters

SI	131072	
SF	150.8701603	MHz
WDW	EM	
SSB	0	
LB	1.00	Hz
GB	0	
PC	1.40	

IR spectrum of **3i**

**143568**	
**PGY0758_1C**	

Pusztai Gyongyver	KP
KBr	10/7/2024

BRUKER Alpha
Resolution: 2 cm-1
Number of Scans: 16

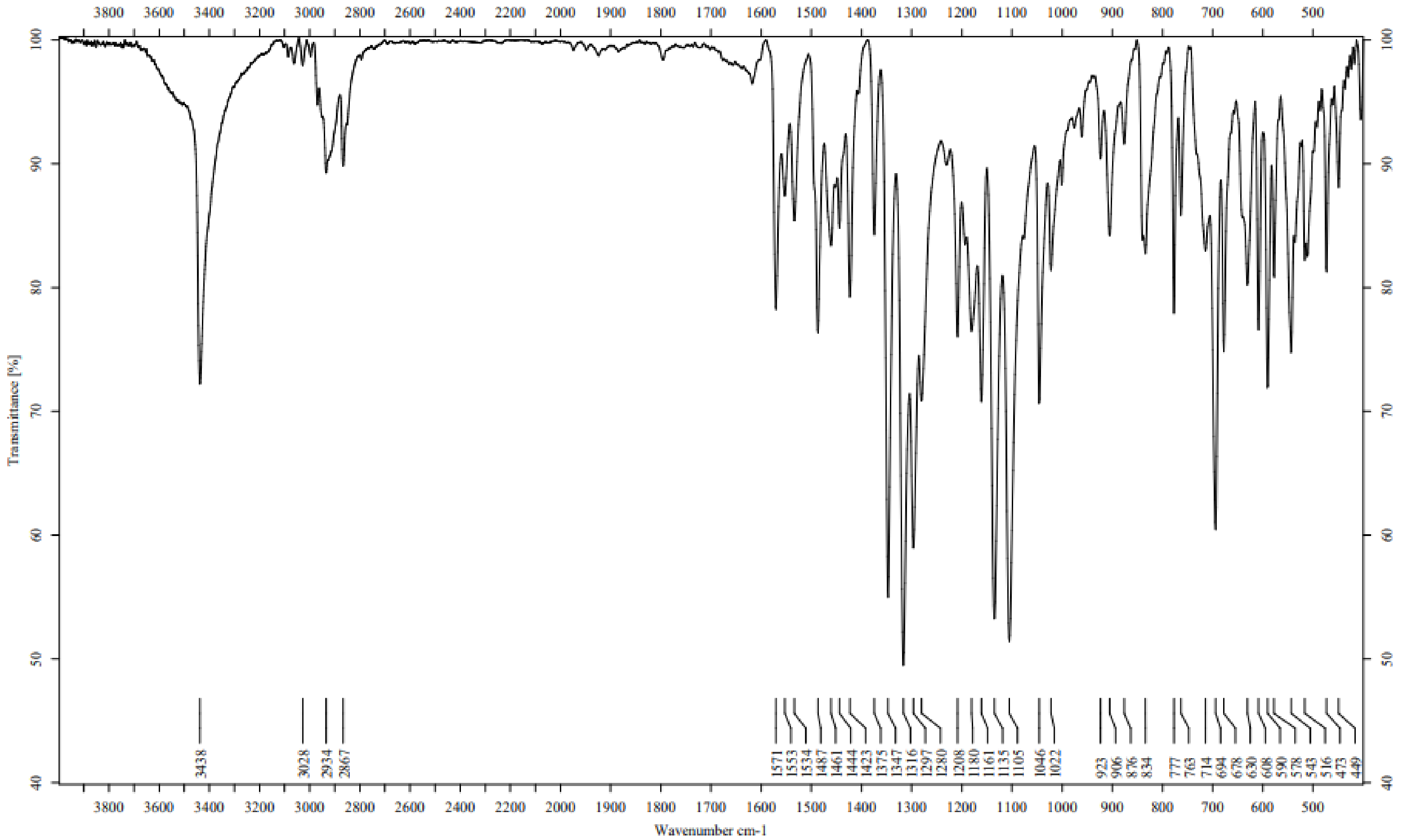

HRMS spectrum of **3i**

Spectrum Plot Report

Agilent

Name	PGY0758_1C, Pusztai Gyongyver	Rack Pos.		Instrument	7250A with DIP	Operator	MM
Inj. Vol. (ul)	0.5	Plate Pos.		IRM Status	Success		
Data File	143568msaqtof_dip.D	Method (Acq)	DIP_70eV.M	Comment		Acq. Time (Local)	10/24/2024 8:22:46 AM (UTC+02:00)

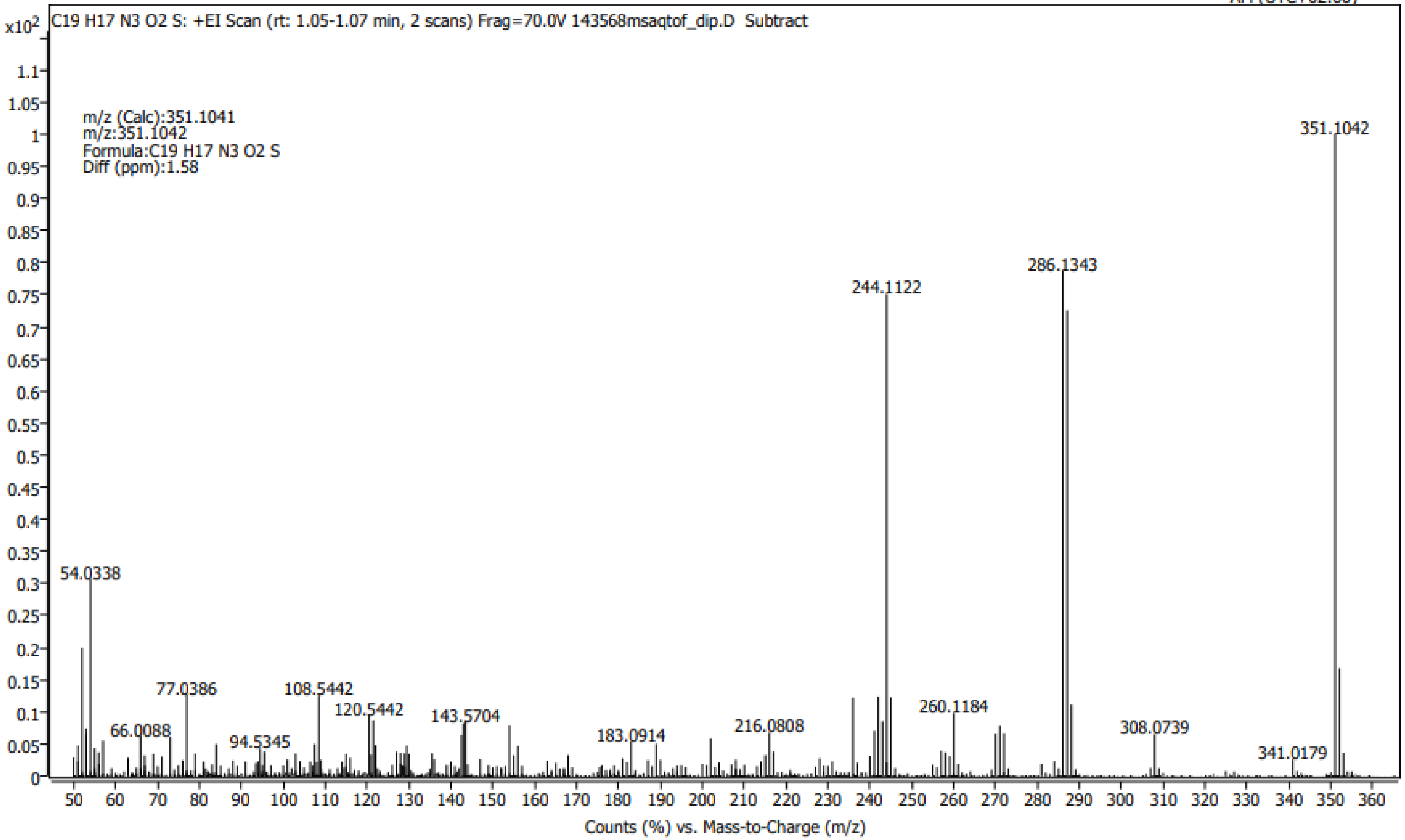

^{1}H NMR spectrum of **3j**

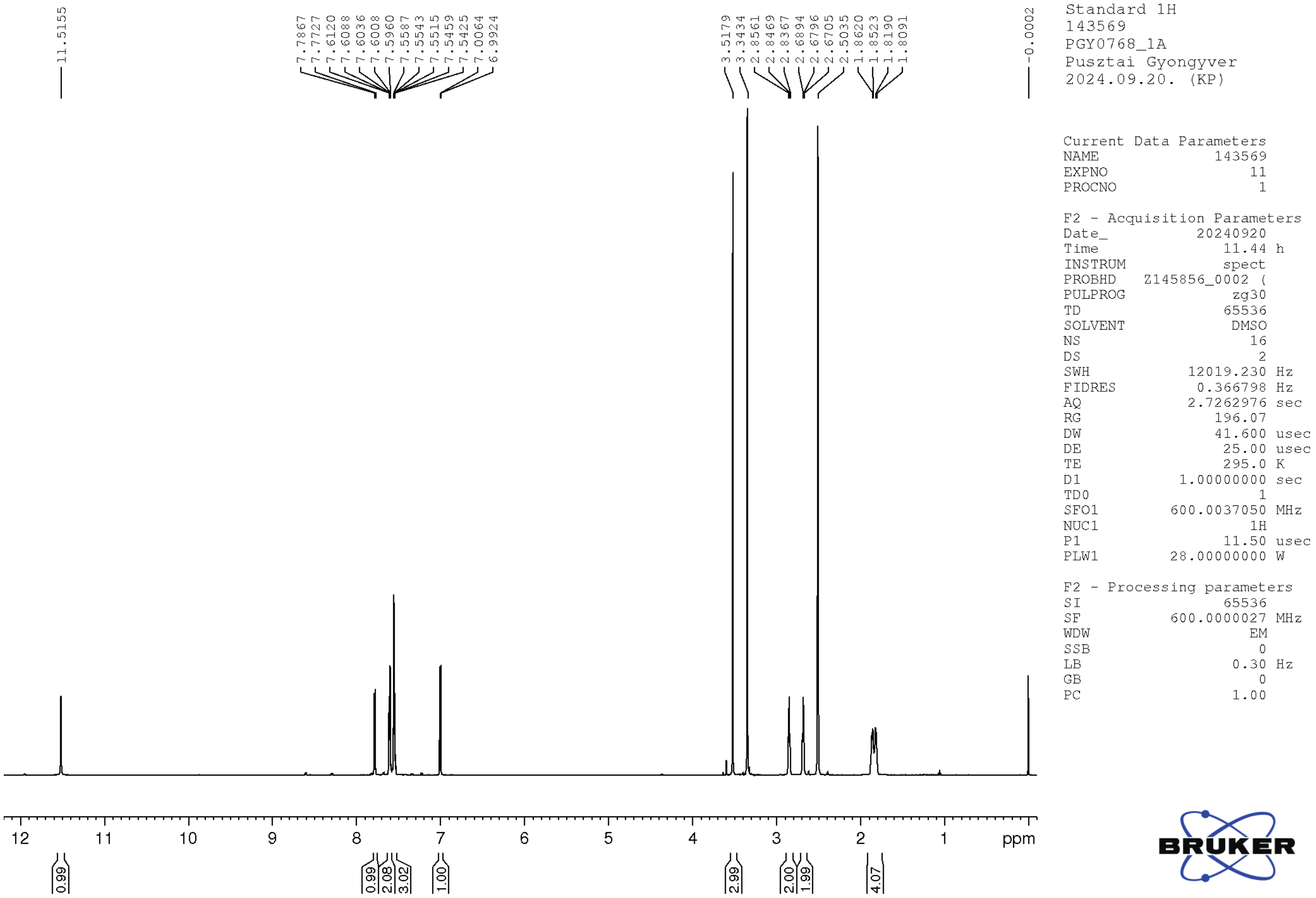

^{13}C NMR spectrum of **3j**

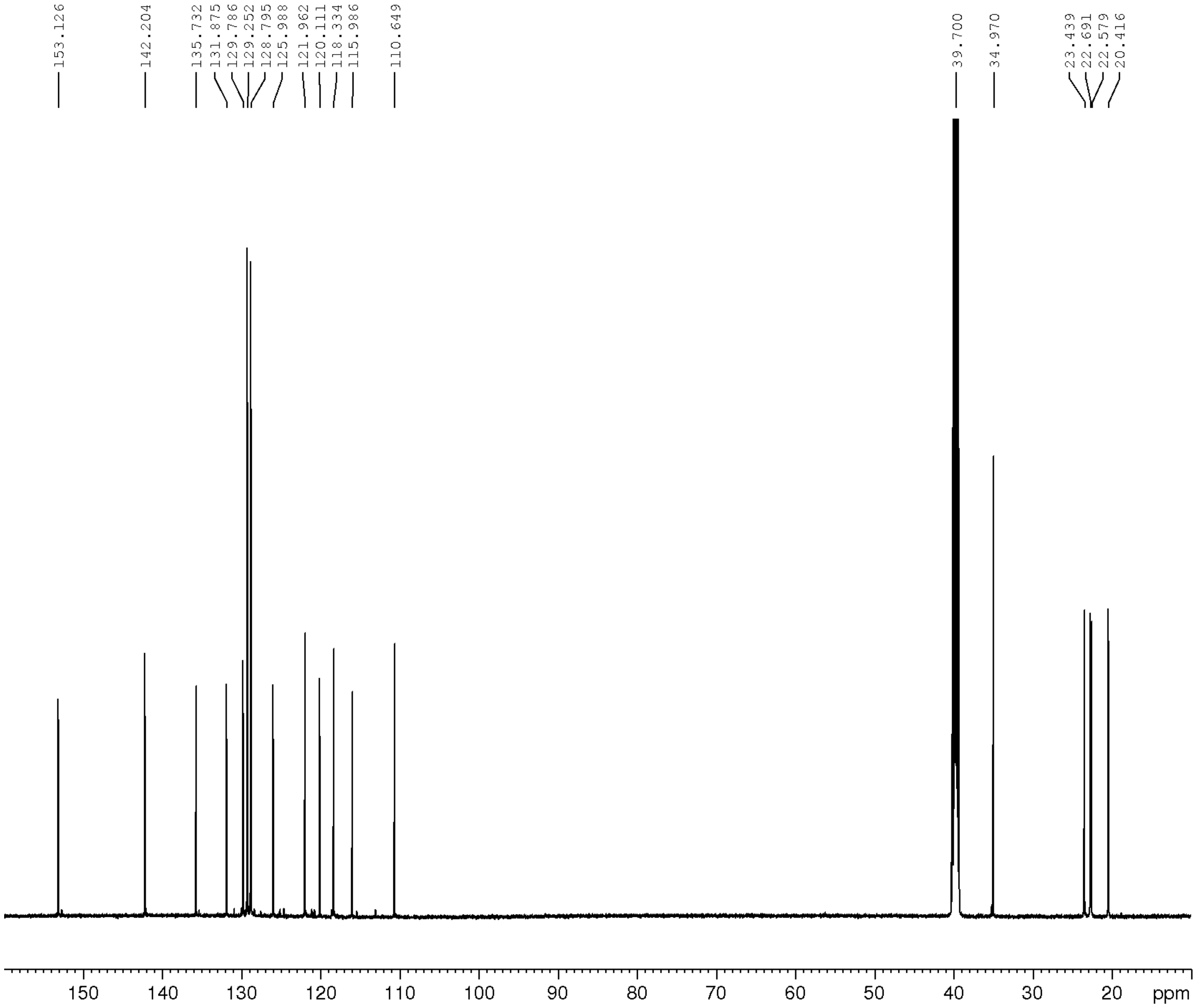

Standard 13C
143569
PGY0768_1A
Pusztai Gyongyver
2024.09.30. (KP)

Current Data Parameters

NAME	143569	
EXPNO	12	
PROCNO	1	

F2 - Acquisition Parameters

Date_	20240930	
Time	16.44	h
INSTRUM	spect	
PROBHD	Z145856_0002 (	
PULPROG	zgpg30	
TD	65536	
SOLVENT	DMSO	
NS	2048	
DS	4	
SWH	36231.883	Hz
FIDRES	1.105709	Hz
AQ	0.9043968	sec
RG	196.07	
DW	13.800	usec
DE	18.00	usec
TE	295.0	K
D1	1.00000000	sec
D11	0.03000000	sec
TD0	1	
SFO1	150.8852070	MHz
NUC1	13C	
P1	9.90	usec
PLW1	71.00000000	W
SFO2	600.0024000	MHz
NUC2	1H	
CPDPRG[2	waltz16	
PCPD2	80.00	usec
PLW2	32.90000153	W
PLW12	0.70370001	W
PLW13	0.35339001	W

F2 - Processing parameters

SI	131072	
SF	150.8701587	MHz
WDW	EM	
SSB	0	
LB	1.00	Hz
GB	0	
PC	1.40	

IR spectrum of **3j**

**143569**	Pusztai Gyongyver	KP	BRUKER Alpha
			Resolution: 2 cm-1
**PGY0768_1A**	KBr	9/30/2024	Number of Scans: 16

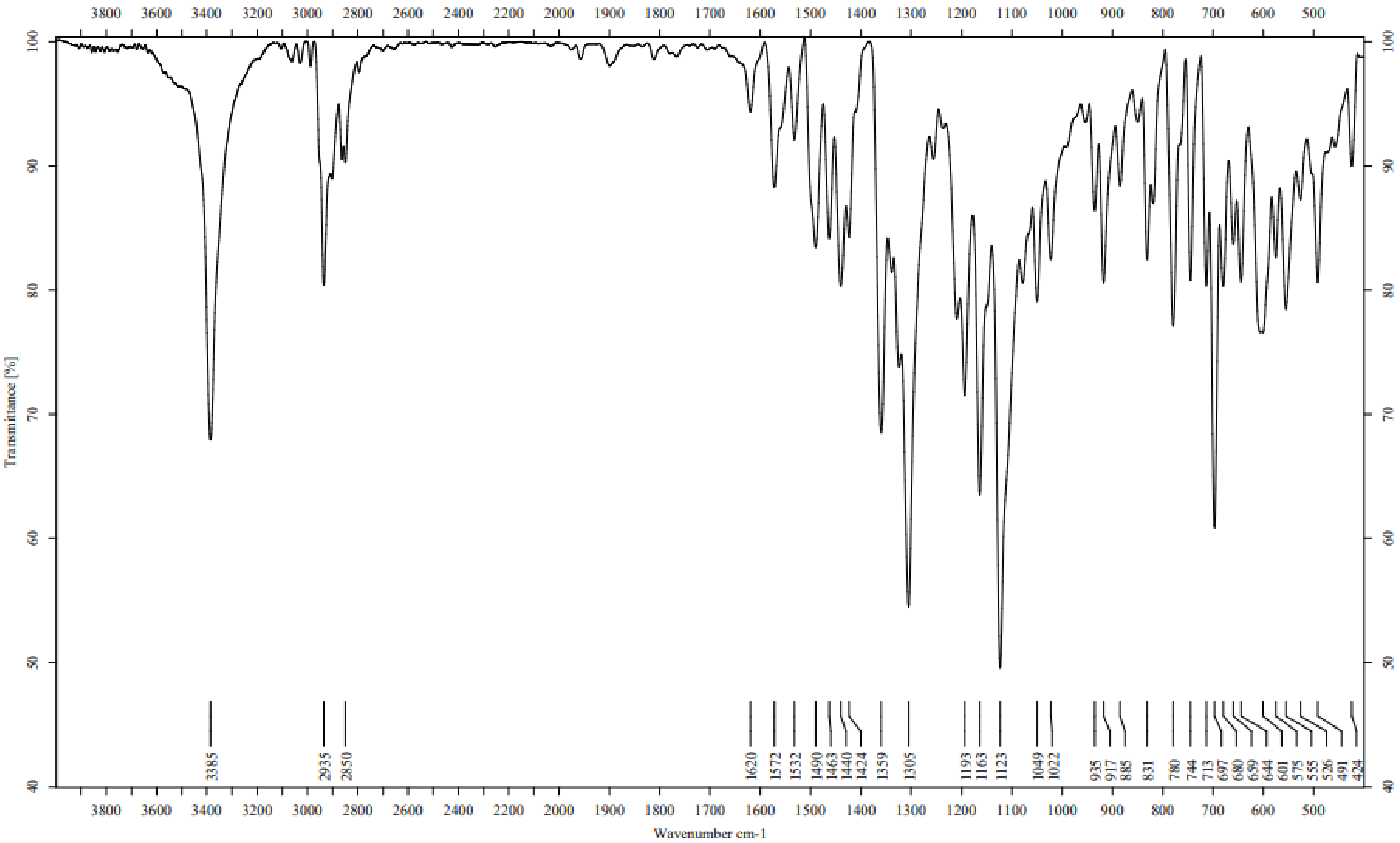

HRMS spectrum of **3j**

Spectrum Plot Report

Name		Rack Pos.		Instrument		Operator	
Inj. Vol. (ul)		Plate Pos.		IRM Status	Success		
Data File	143569msaqtof_dip2.D	Method (Acq)		Comment		Acq. Time (Local)	10/24/2024 1:11:43 PM (UTC+02:00)

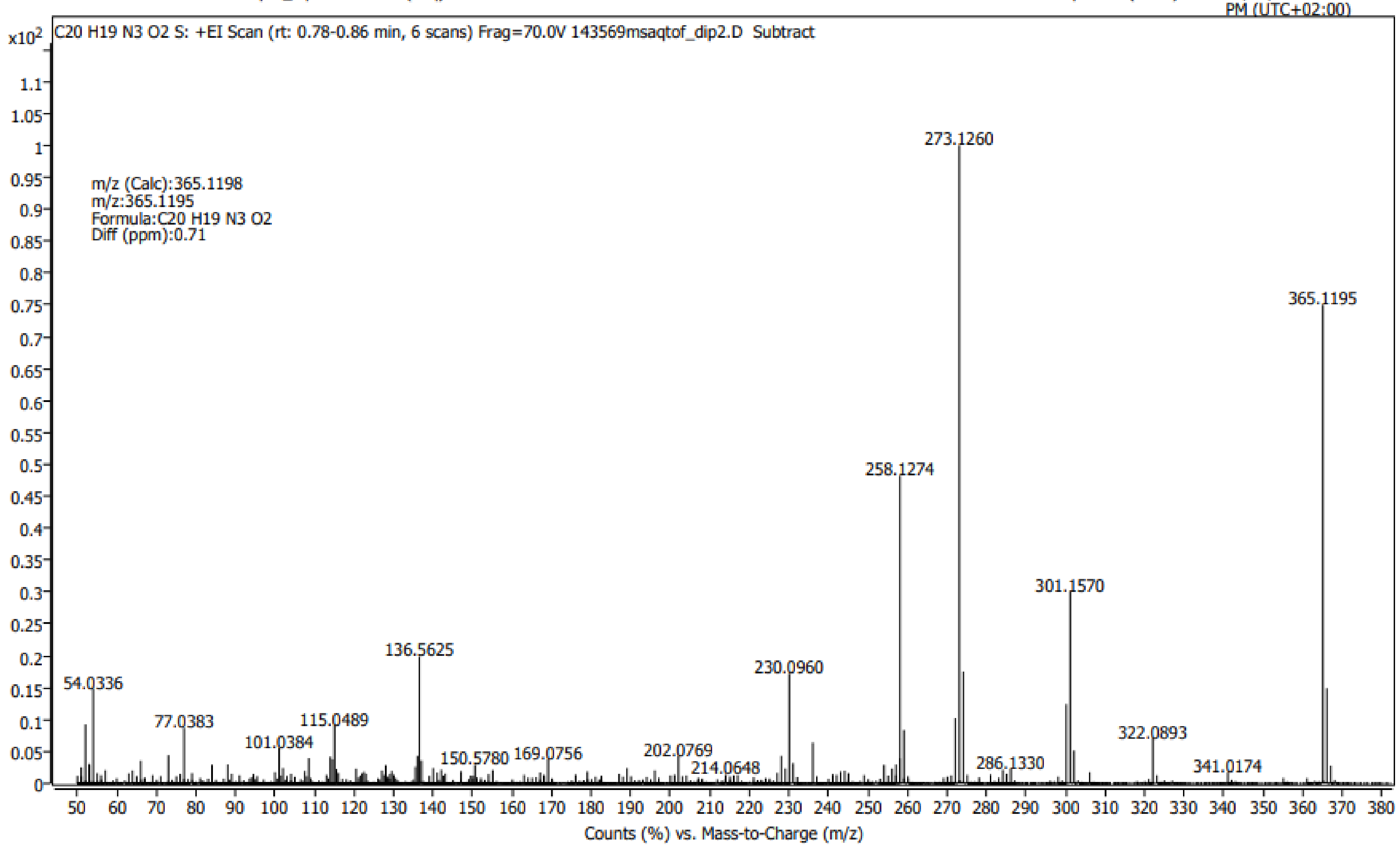

^{1}H NMR spectrum of **10a**

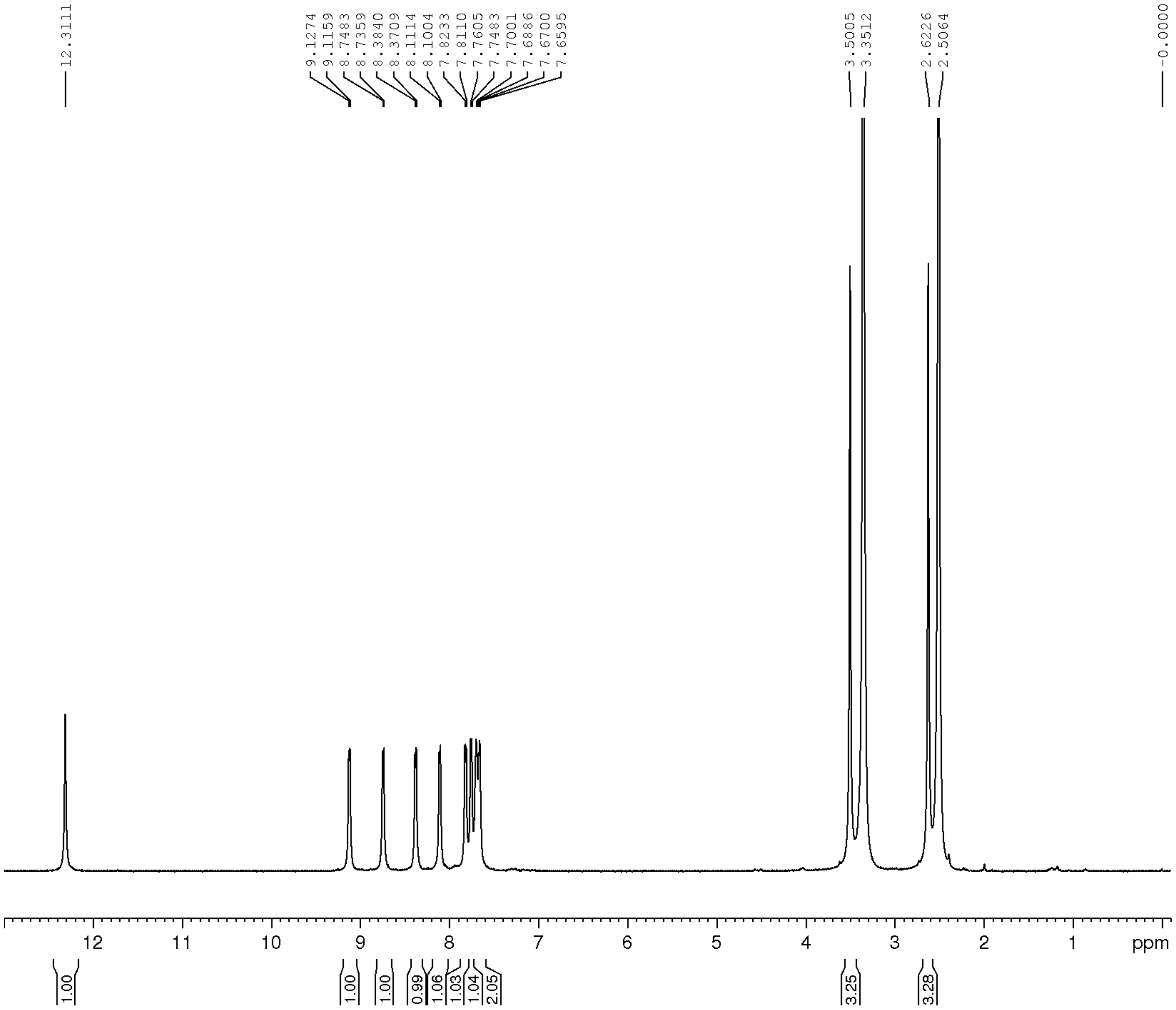

Standard 1H
144057
PGY0835_2A
Pusztai Gyongyver
2024.01.07. (KP)

Current Data Parameters

NAME	144057
EXPNO	12
PROCNO	1

F2 - Acquisition Parameters

Date_	20250107
Time	14.28 h
INSTRUM	spect
PROBHD	Z145856_0002 (
PULPROG	zg30
TD	65536
SOLVENT	DMSO
NS	16
DS	2
SWH	12019.230 Hz
FIDRES	0.366798 Hz
AQ	2.7262976 sec
RG	196.07
DW	41.600 usec
DE	25.00 usec
TE	295.0 K
D1	1.00000000 sec
TD0	1
SFO1	600.0037050 MHz
NUC1	1H
P1	11.50 usec
PLW1	28.00000000 W

F2 - Processing parameters

SI	65536
SF	600.0000012 MHz
WDW	EM
SSB	0
LB	0.30 Hz
GB	0
PC	1.00

^{13}C NMR spectrum of **10a**

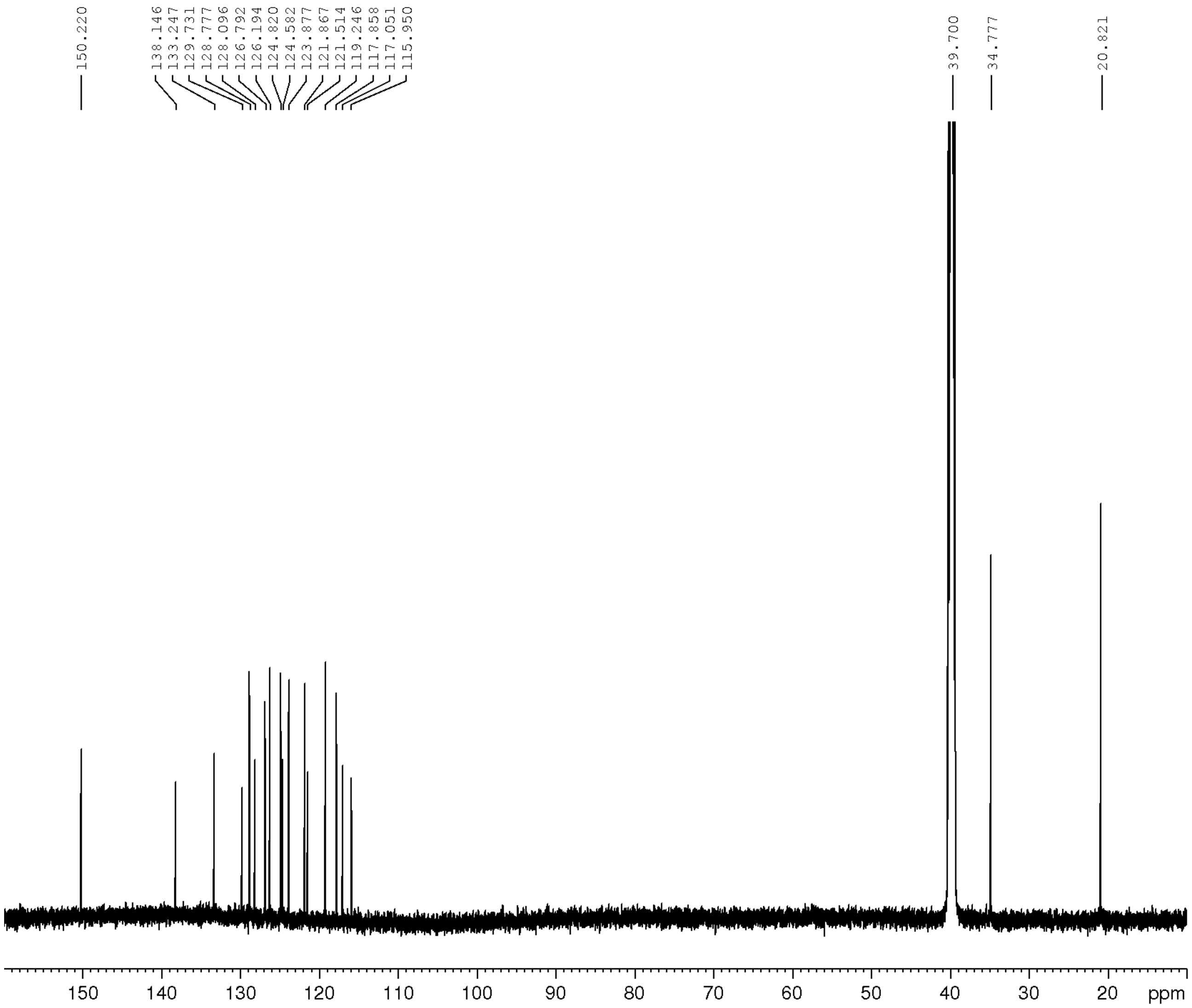

Standard 13C
144057
PGY0835_2A
Pusztai Gyongyver
2025.01.07. (KP)

Current Data Parameters

NAME	144057	
EXPNO	13	
PROCNO	1	

F2 - Acquisition Parameters

Date_	20250107	
Time	16.03	h
INSTRUM	spect	
PROBHD	Z145856_0002 (	
PULPROG	zgpg30	
TD	65536	
SOLVENT	DMSO	
NS	1024	
DS	4	
SWH	36231.883	Hz
FIDRES	1.105709	Hz
AQ	0.9043968	sec
RG	196.07	
DW	13.800	usec
DE	18.00	usec
TE	295.0	K
D1	1.00000000	sec
D11	0.03000000	sec
TD0	1	
SFO1	150.8852070	MHz
NUC1	13C	
P1	9.90	usec
PLW1	71.00000000	W
SFO2	600.0024000	MHz
NUC2	1H	
CPDPRG[2	waltz16	
PCPD2	80.00	usec
PLW2	32.90000153	W
PLW12	0.70370001	W
PLW13	0.35339001	W

F2 - Processing parameters

SI	131072	
SF	150.8701597	MHz
WDW	EM	
SSB	0	
LB	1.00	Hz
GB	0	
PC	1.40	

IR spectrum of **10a**

144057	Pusztai Gyongyver	KP	BRUKER Alpha
PGY0835_2A	KBr	1/8/2025	Resolution: 2 cm-1
			Number of Scans: 16

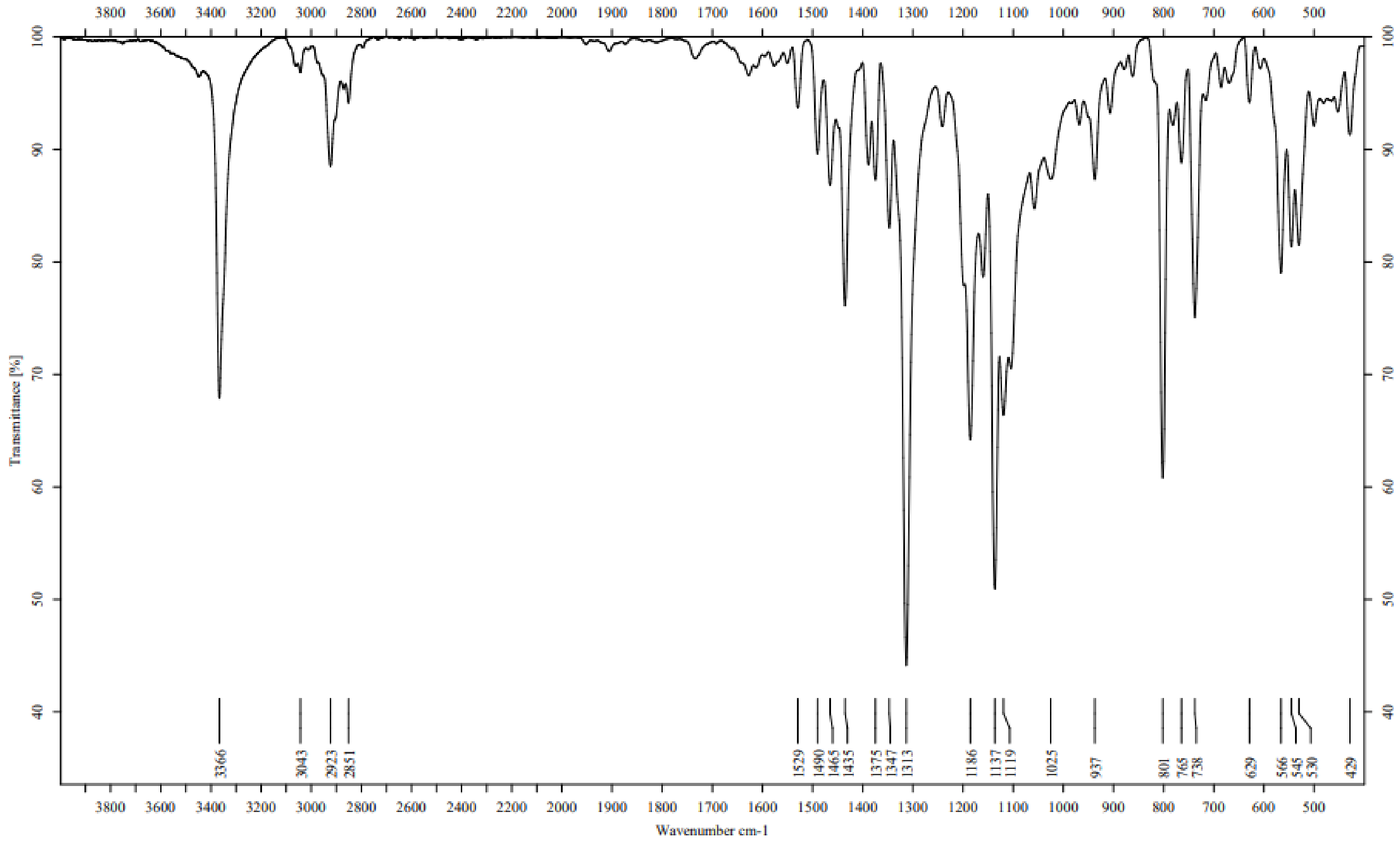

HRMS spectrum of **10a**

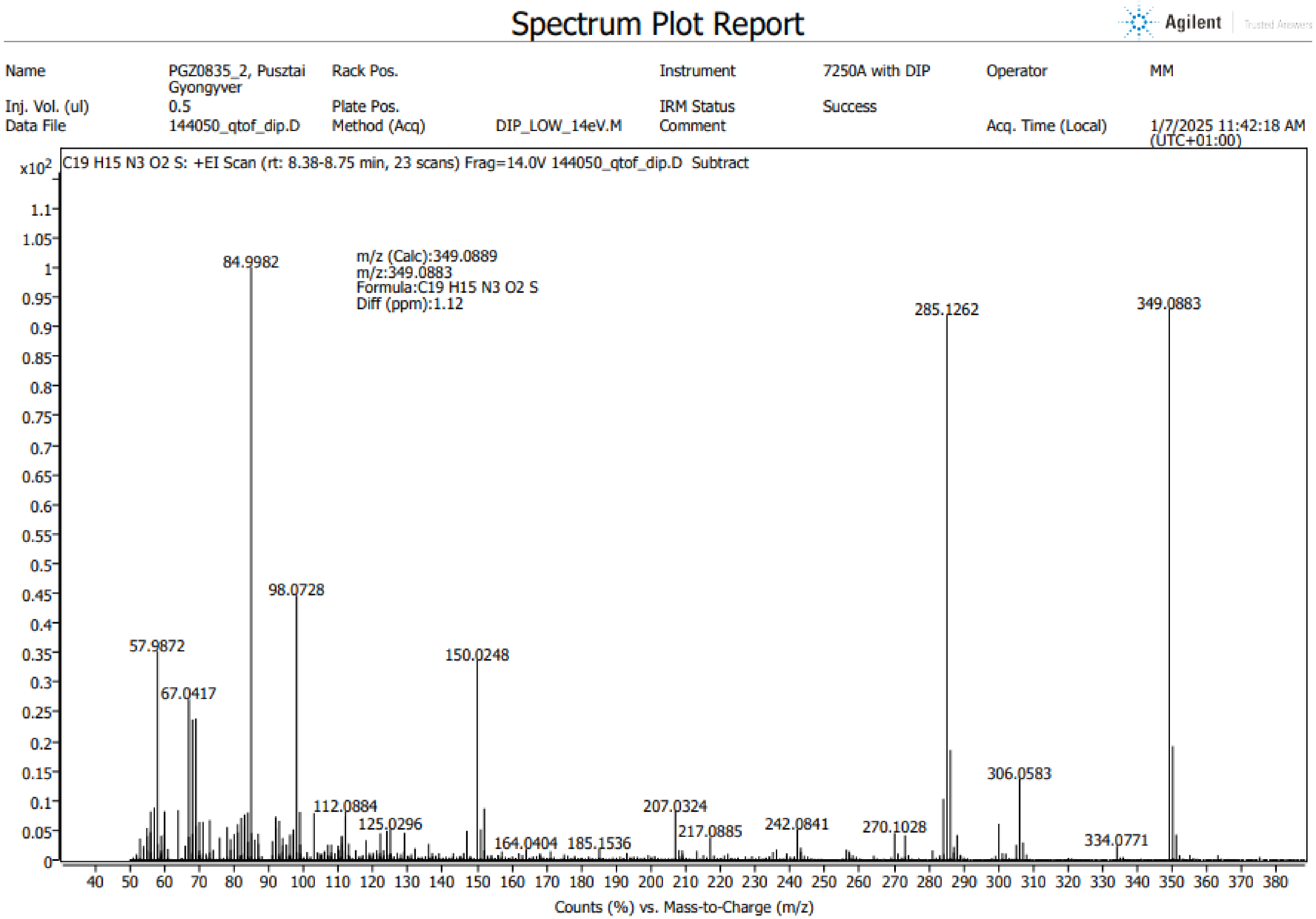

^{1}H NMR spectrum of **10b**

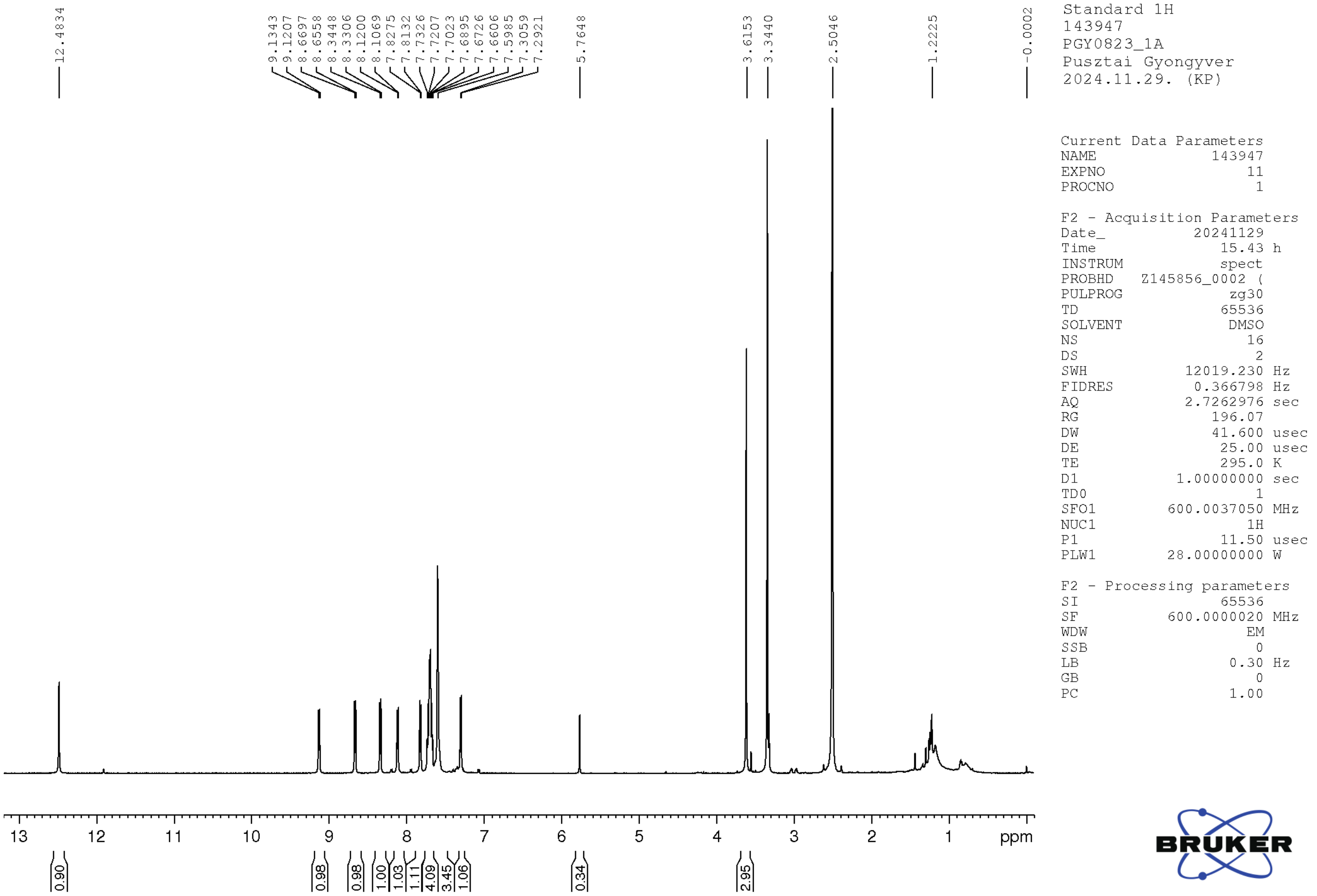

^{13}C NMR spectrum of **10b**

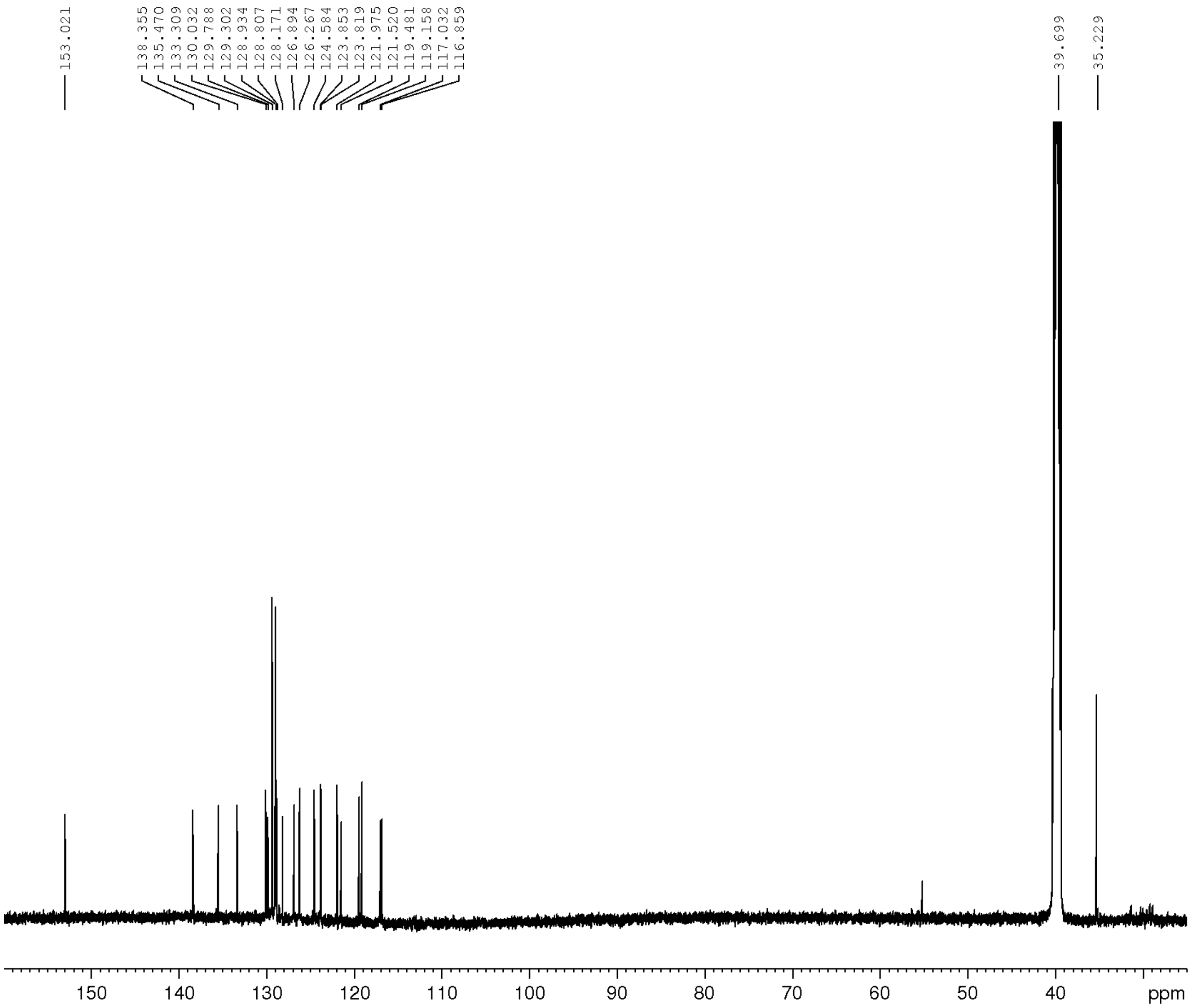

Standard 13C
143947
PGY0823_1A
Pusztai Gyongyver
2024.11.29. (KP)

Current Data Parameters

NAME	143947
EXPNO	12
PROCNO	1

F2 - Acquisition Parameters

Date_	20241129
Time	17.27 h
INSTRUM	spect
PROBHD	Z145856_0002 (
PULPROG	zgpg30
TD	65536
SOLVENT	DMSO
NS	2048
DS	4
SWH	36231.883 Hz
FIDRES	1.105709 Hz
AQ	0.9043968 sec
RG	196.07
DW	13.800 usec
DE	18.00 usec
TE	295.0 K
D1	1.00000000 sec
D11	0.03000000 sec
TD0	1
SFO1	150.8852070 MHz
NUC1	13C
P1	9.90 usec
PLW1	71.00000000 W
SFO2	600.0024000 MHz
NUC2	1H
CPDPRG[2	waltz16
PCPD2	80.00 usec
PLW2	32.90000153 W
PLW12	0.70370001 W
PLW13	0.35339001 W

F2 - Processing parameters

SI	131072
SF	150.8701607 MHz
WDW	EM
SSB	0
LB	1.00 Hz
GB	0
PC	1.40

IR spectrum of **10b**

**143947**	Pusztai Gyongyver	KP	BRUKER Alpha
**PGY0823_1A**	KBr	1/30/2025	Resolution: 2 cm-1
			Number of Scans: 16

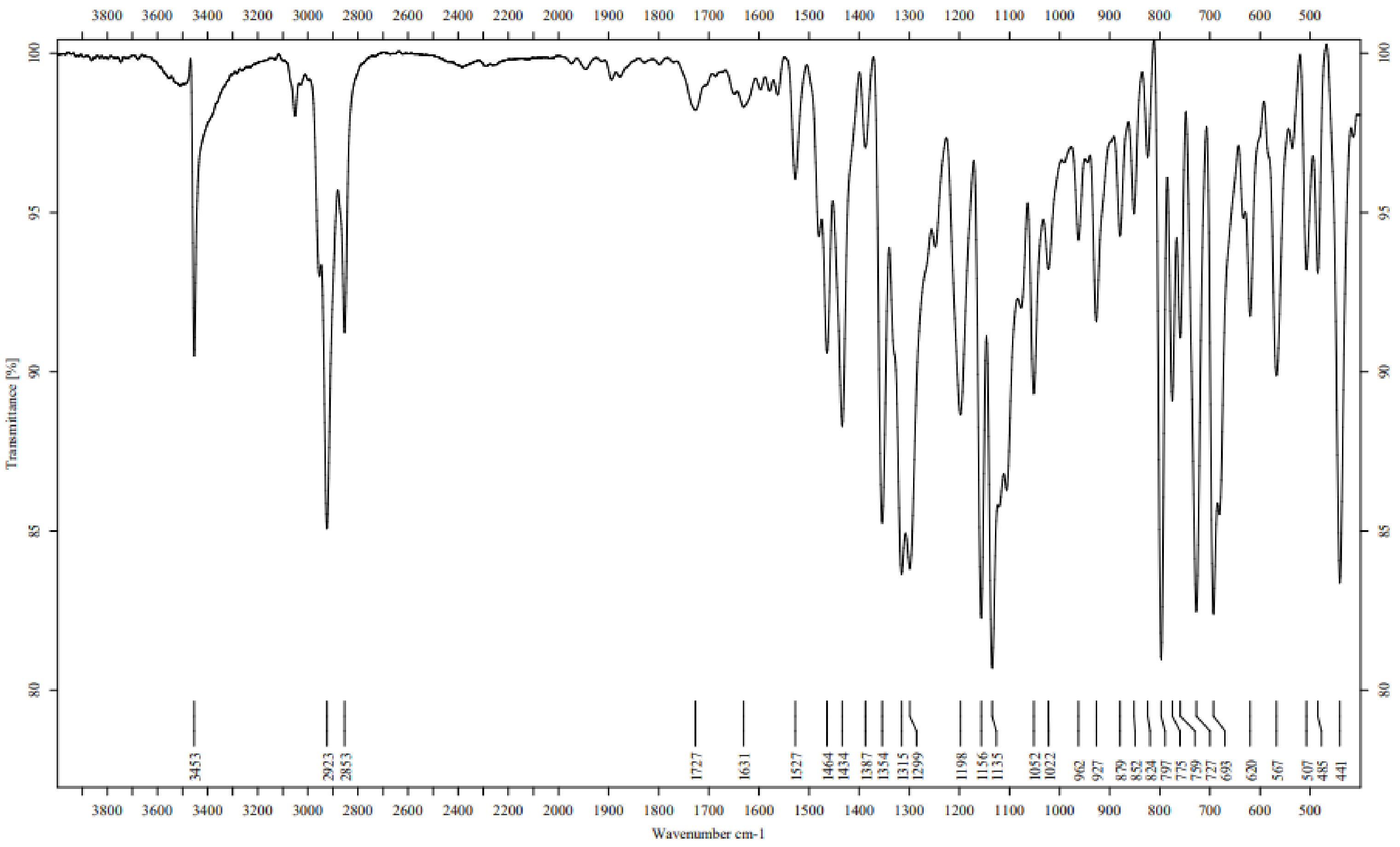

HRMS spectrum of **10b**

Spectrum Plot Report

Name	PGY0823_1A, Pusztai Gyongyver	Rack Pos.		Instrument	7250A with DIP	Operator	MM
Inj. Vol. (ul)	0.5	Plate Pos.		IRM Status	Success		
Data File	143947_qtof_dip.D	Method (Acq)	DIP_70eV.M	Comment		Acq. Time (Local)	12/3/2024 9:53:56 AM (UTC+01:00)

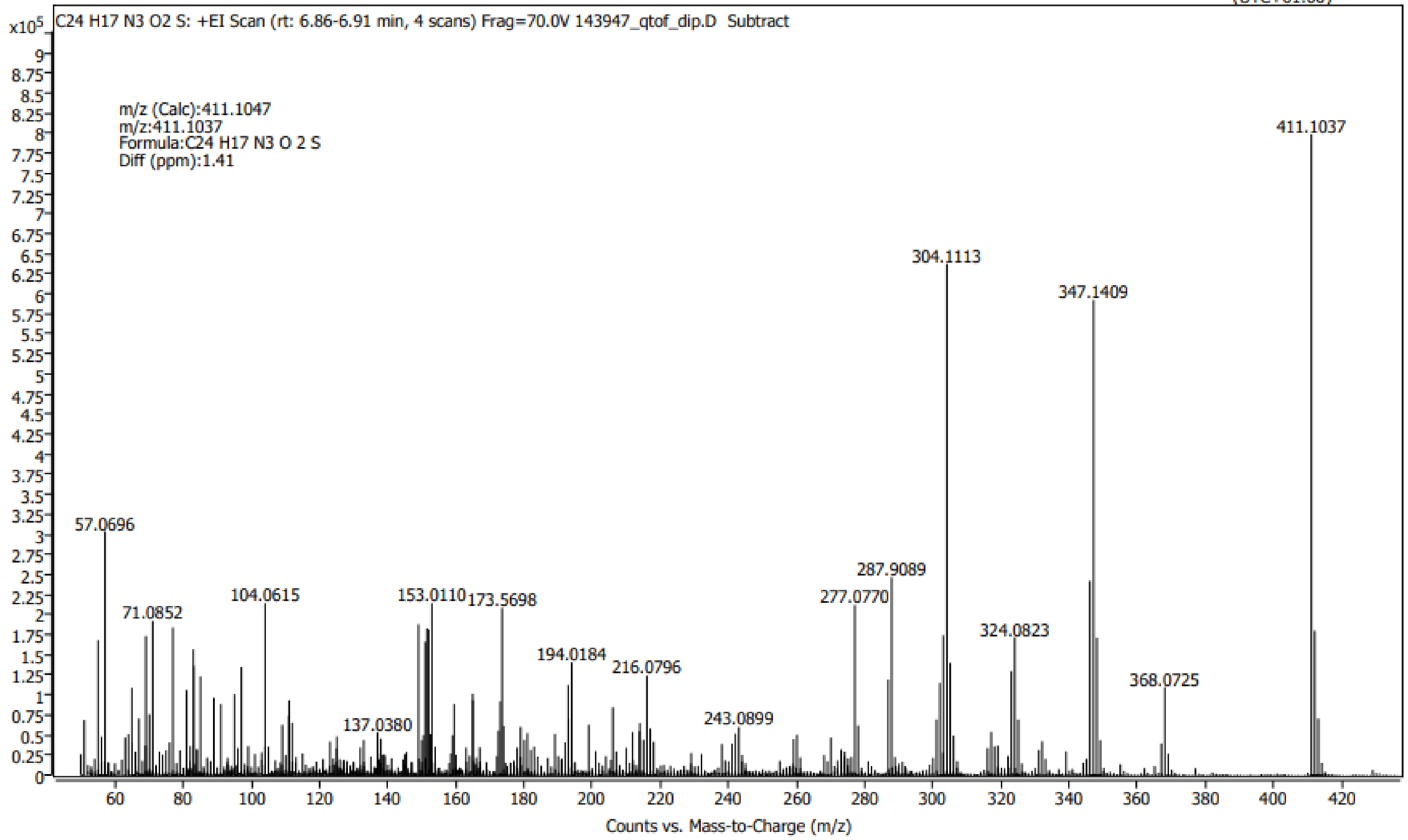

Supplement: File 1 — Experimental procedures and characterization of compounds (E)-7a, 7b, (E)-7c, (Z)-7c, 7d, (E)-7f, 7g, 7i, 7j, (E)-9a, (E)-9b, 3a–d, 3f, 3g, 3i, 3j, 10a, 10b. Kinetic solubility of compounds 7a–j, 3a–j, (E)-9a, (E)-9b, 10a and 10b; permeability and membrane retention of compounds (E)-7a, (E)-7c, (Z)-7c, 7d, 7e, 3b, 3c and 3e. [file Beilstein_J_Org_Chem-21-2220-s001.pdf]
