# Supplementary material for: Thiadiazino-indole, thiadiazino-carbazole and benzothiadiazino-carbazole dioxides: synthesis, physicochemical and early ADME characterization of representatives of new tri-, tetra- and pentacyclic ring systems and their intermediates
Source: Beilstein J Org Chem. 2025 Oct 21;21:2220–33. doi: 10.3762/bjoc.21.169 (PMC12557438; doi:10.3762/bjoc.21.169)
Supplement: File 2 — Crystallographic information files, checkcif and structure report files for compounds 3b, 3d, 3e, 3g, 3h, (E)-7a, 7b, 7d, 7e, (E)-7f, (Z)-7h, 7i and (E)-9a. [file Beilstein_J_Org_Chem-21-2220-s002.zip › Átnevezett XRD/3e_xrd.pdf]

142501

PGY0676_1A

Submitted by: Pusztai Gyongyver
Operator: Dancso Andras

X-ray Structure Report

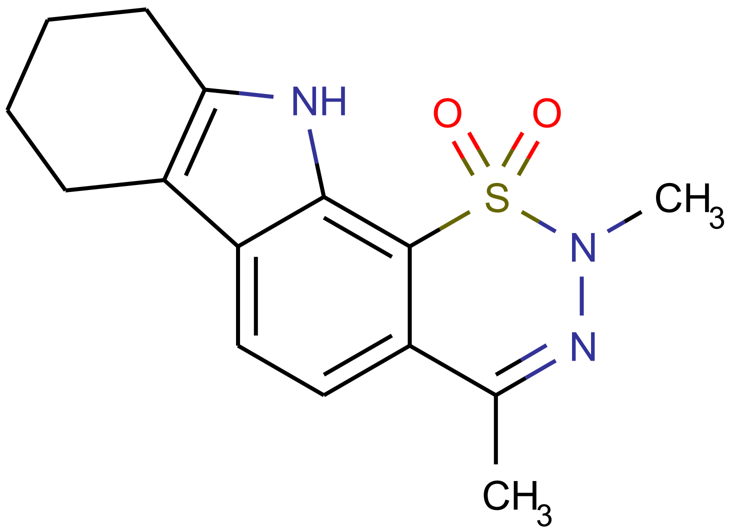

January 14, 2025

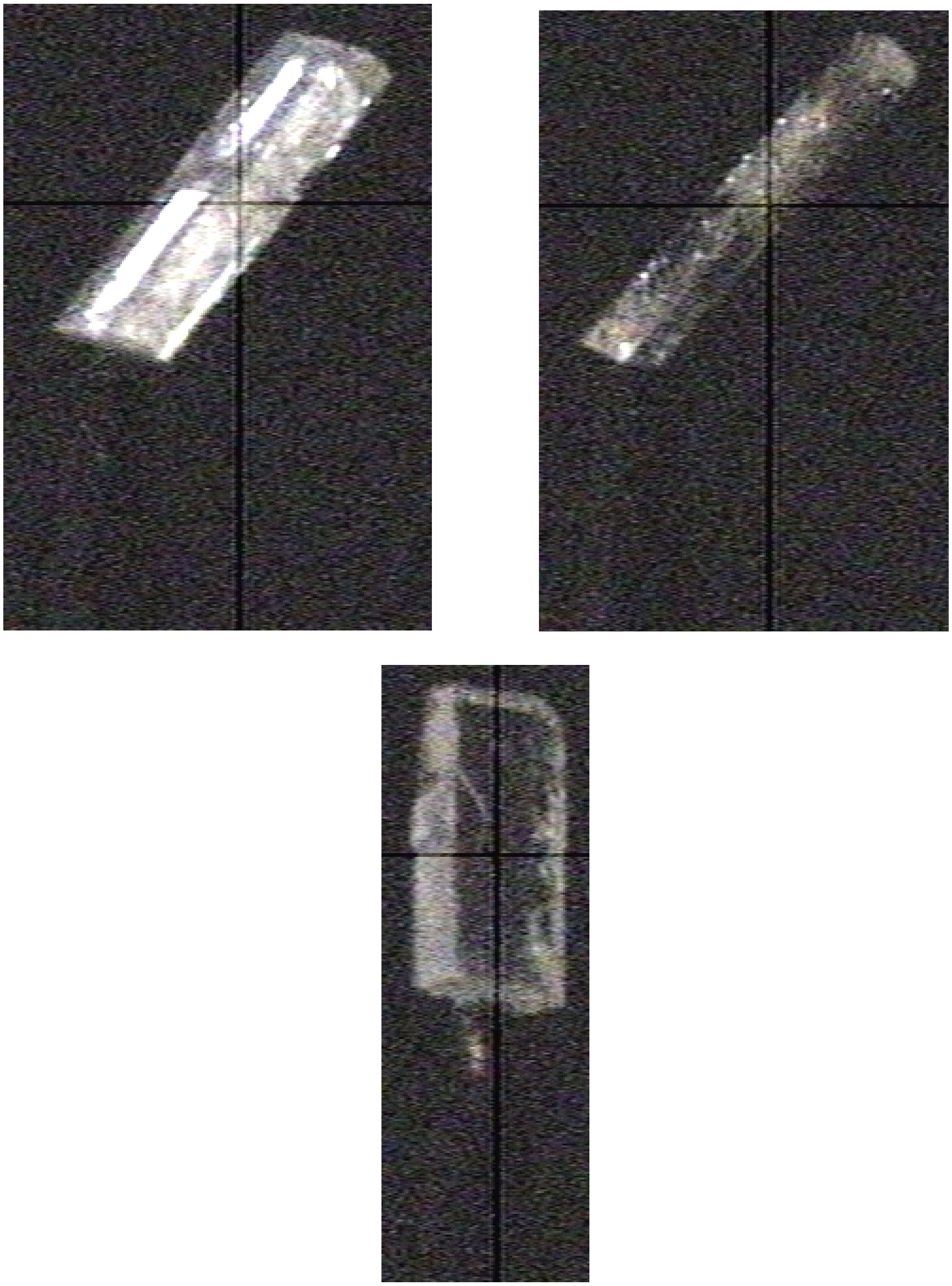

Fig. 1. The crystal

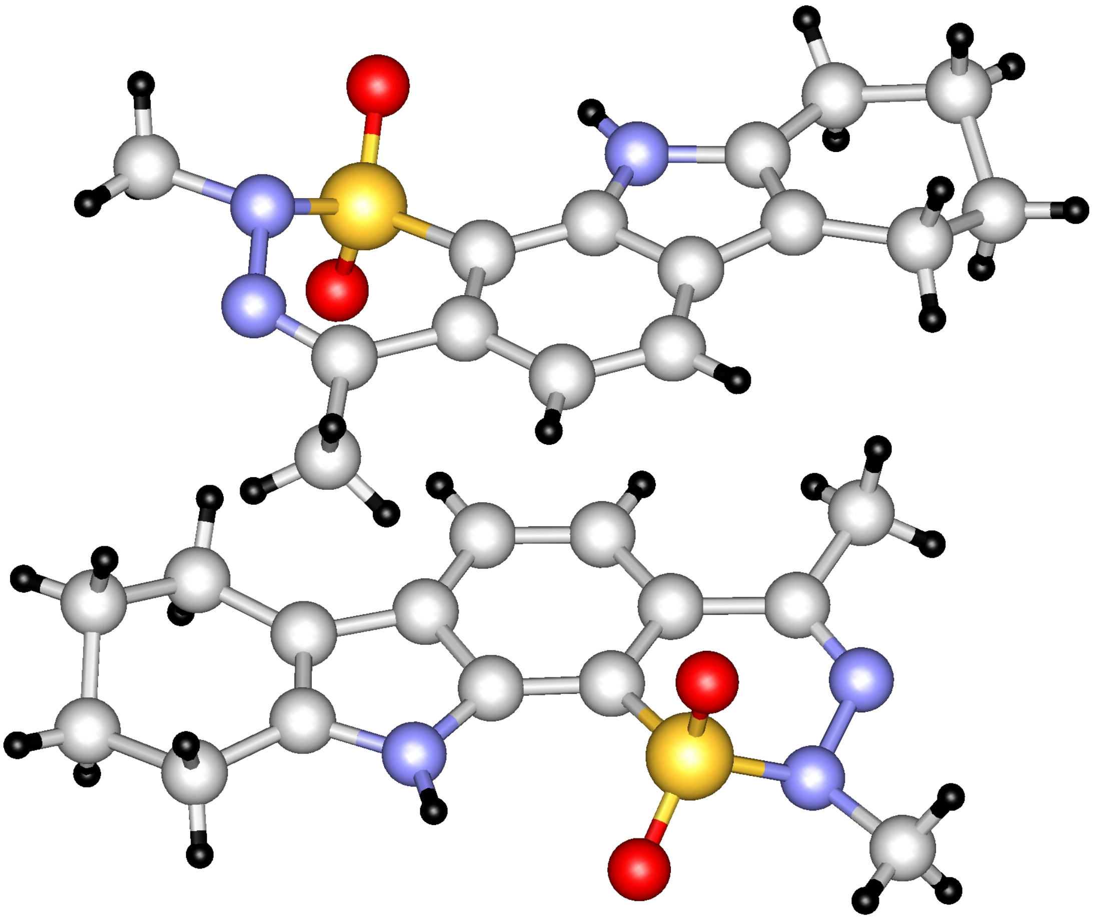

Fig. 2. Molecules in pair (hydrogens were generated by the software)

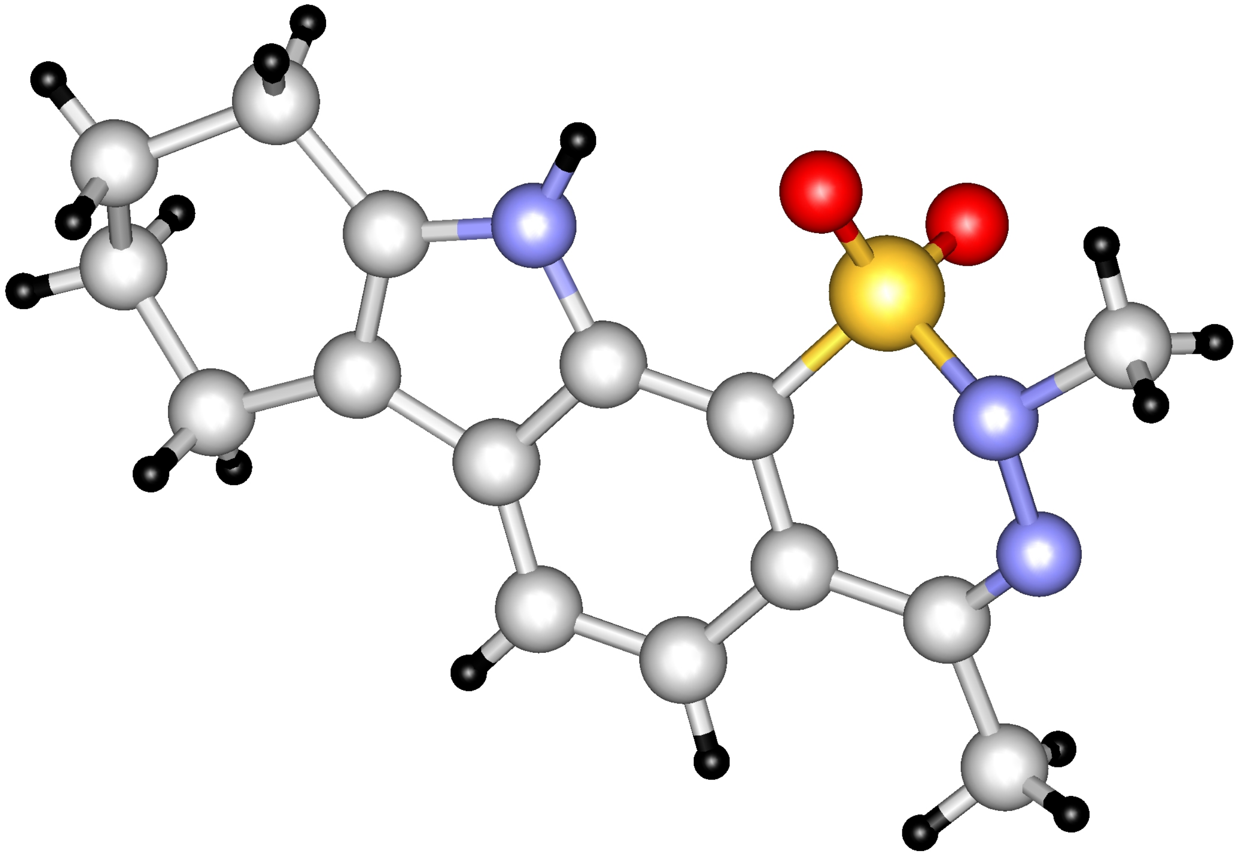

Fig. 3. Fragment 1

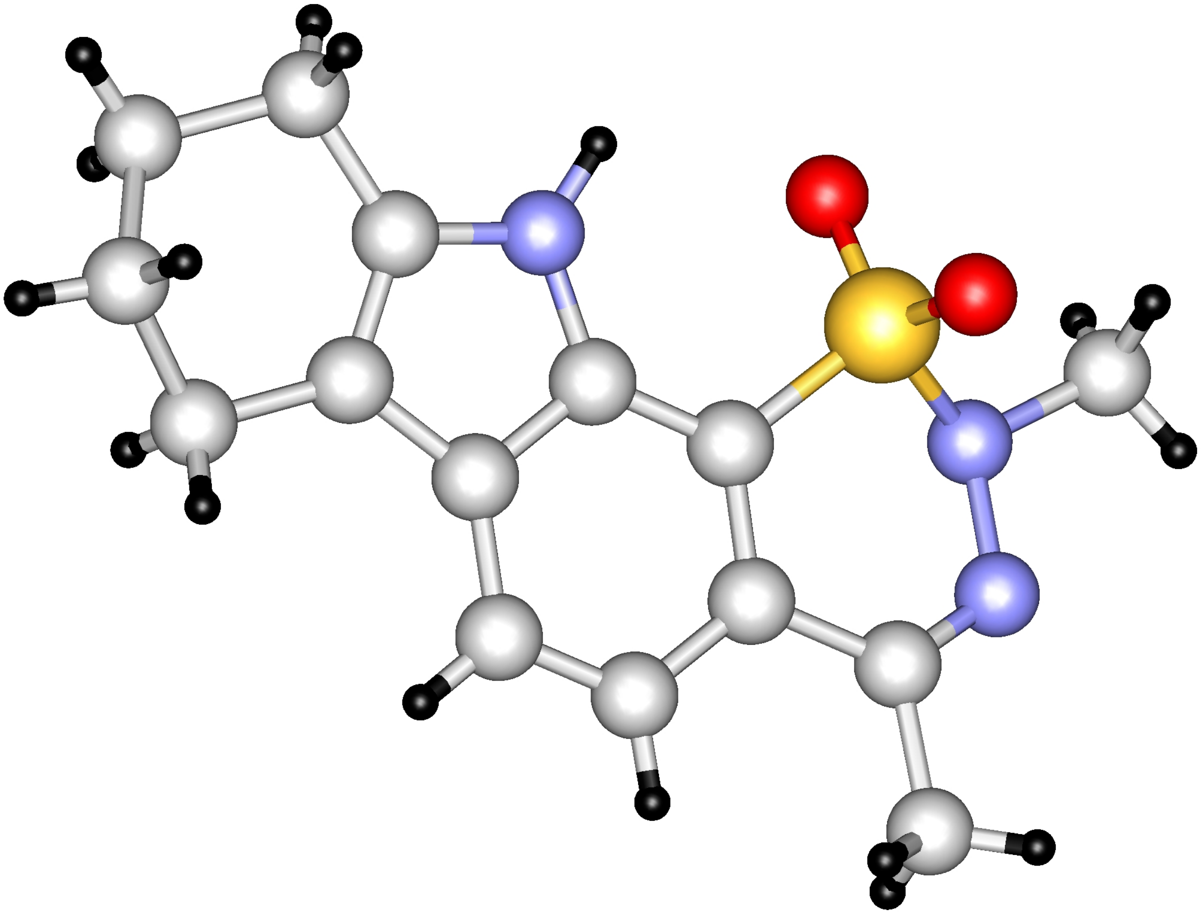

Fig. 4. Fragment 2

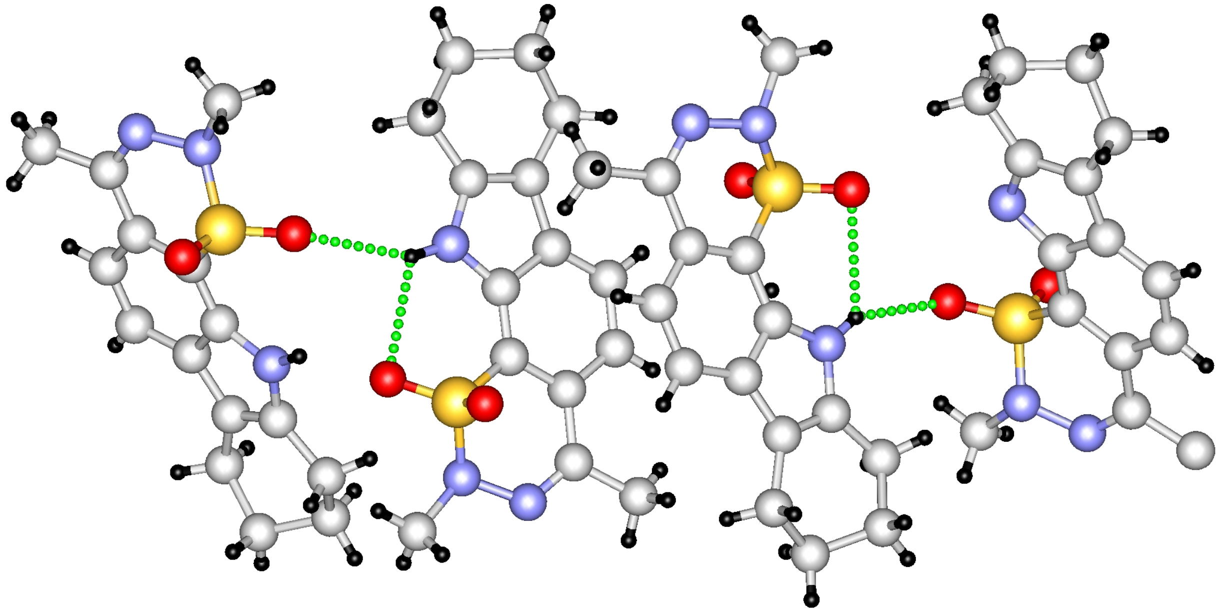

Fig. 5. Hydrogen bonds

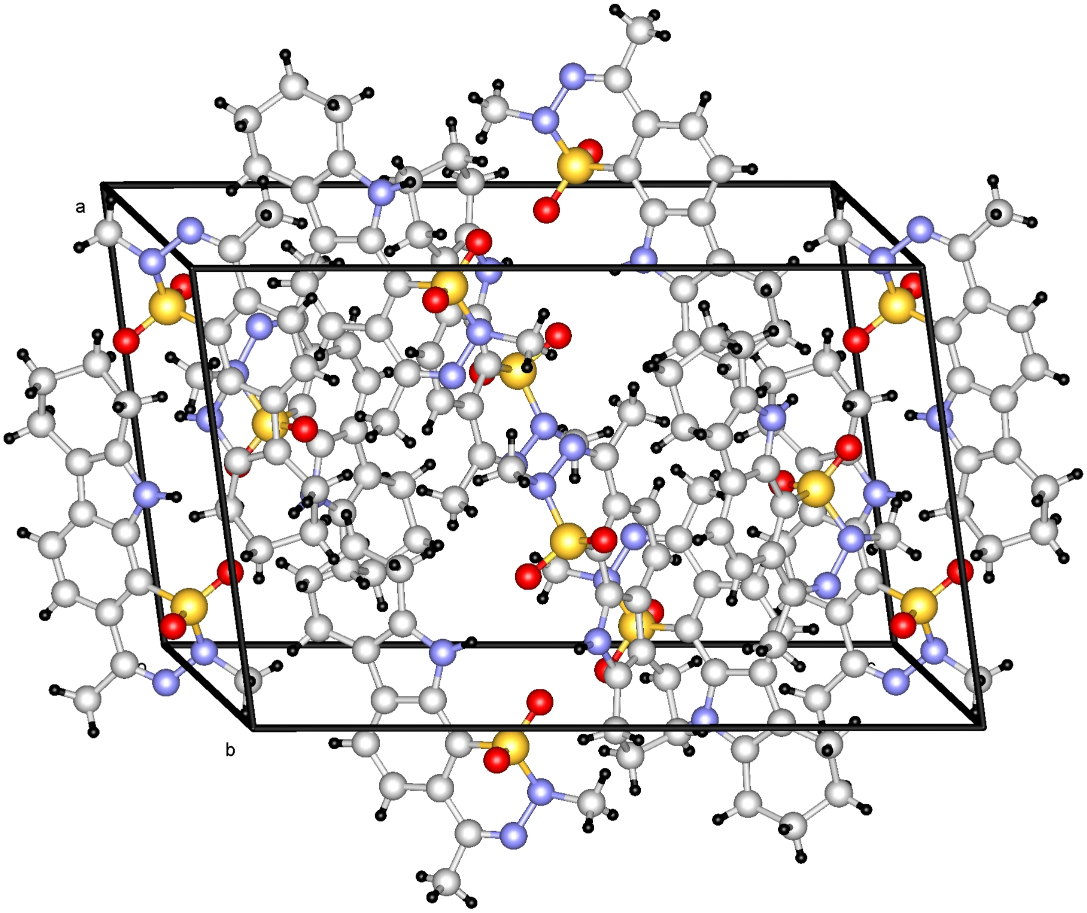

Fig. 6. Packing

Experimental

Data Collection

A colorless prism crystal of $C_{15}H_{17}N_3O_2S$ having approximate dimensions of 0.64 x 0.24 x 0.12 mm was mounted on a cactus needle. All measurements were made on a Rigaku RAXIS RAPID imaging plate area detector with graphite monochromated Cu-Kα radiation.

Indexing was performed from 4 oscillations that were exposed for 180 seconds. The crystal-to-detector distance was 127.40 mm.

Cell constants and an orientation matrix for data collection corresponded to a primitive monoclinic cell with dimensions:

a = 12.9021(7) Å

b = 11.1229(6) Å β = 100.079(4)^o

c = 20.2138(14) Å

V = 2856.1(3) Å^3

For Z = 8 and F.W. = 303.38, the calculated density is 1.411 g/cm^3. The systematic absences of:

h0l: h+l ± 2n
0k0: k ± 2n

uniquely determine the space group to be:

$P2_1/n$ (#14)

The data were collected at a temperature of 20 $\pm$ 1°C to a maximum 2θ value of 143.5^o. A total of 180 oscillation images were collected. A sweep of data was done using ω scans from 20.0 to 200.0^o in 5.0^o step, at χ=0.0^o and ϕ = 0.0^o. The exposure rate was 36.0 [sec./^{o}]. A second sweep was performed using ω scans from 20.0 to 200.0^o in 5.0^o step, at χ=54.0^o and ϕ = 0.0^o. The exposure rate was 36.0 [sec./^{o}]. Another sweep was performed using ω scans from 20.0 to 200.0^o in 5.0^o step, at χ=54.0^o and ϕ = 90.0^o. The exposure rate was 36.0 [sec./^{o}]. Another sweep was performed using ω scans from 20.0 to 200.0^o in 5.0^o step, at χ=54.0^o and ϕ = 180.0^o. The exposure rate was 36.0 [sec./^{o}]. Another sweep was performed using ω scans from 20.0 to 200.0^o in 5.0^o step, at χ=54.0^o and ϕ = 270.0^o. The exposure rate was 36.0 [sec./^{o}]. The crystal-to-detector distance was 127.40 mm. Readout was performed in the 0.100 mm pixel mode.

Data Reduction

Of the 32046 reflections that were collected, 5330 were unique (R_{int} = 0.067).

The linear absorption coefficient, μ, for Cu-Kα radiation is 20.887 cm^{-1}. An empirical absorption correction was applied which resulted in transmission factors ranging from 0.441 to 0.773. The data were corrected for Lorentz and polarization effects.

Structure Solution and Refinement

The structure was solved by direct methods[1] and expanded using Fourier techniques[2]. The non-hydrogen atoms were refined anisotropically. Hydrogen atoms were refined using the riding model. The final cycle of full-matrix least-squares refinement[3] on F was based on 24702 observed reflections ($I > 2.00\sigma(I)$) and 413 variable parameters and converged (largest parameter shift was 0.00 times its esd) with unweighted and weighted agreement factors of:

$$R = \Sigma ||Fo| - |Fc|| / \Sigma |Fo| = 0.1000$$

$$R_w = [\ \Sigma\ w\ (|Fo| - |Fc|)^2 / \Sigma\ w\ Fo^2]^{1/2} = 0.1295$$

The standard deviation of an observation of unit weight[4] was 7.74. Unit weights were used. Plots of $\Sigma\ w\ (|Fo| - |Fc|)^2$ versus |Fo|, reflection order in data collection, sin θ/λ and various classes of indices showed no unusual trends. The maximum and minimum peaks on the final difference Fourier map corresponded to 8.13 and -8.21 $e^-/Å^3$, respectively.

Neutral atom scattering factors were taken from Cromer and Waber[5]. Anomalous dispersion effects were included in Fcalc[6]; the values for Δf' and Δf" were those of Creagh and McAuley[7]. The values for the mass attenuation coefficients are those of Creagh and Hubbell[8]. All calculations were performed using the CrystalStructure[9,10] crystallographic software package.

References

(1) SIR92: Altomare, A., Cascarano, G., Giacovazzo, C., Guagliardi, A., Burla, M., Polidori, G., and Camalli, M. (1994) J. Appl. Cryst., 27, 435.

(2) DIRDIF99: Beurskens, P.T., Admiraal, G., Beurskens, G., Bosman, W.P., de Gelder, R., Israel, R. and Smits, J.M.M.(1999). The DIRDIF-99 program system, Technical Report of the Crystallography Laboratory, University of Nijmegen, The Netherlands.

(3) Least Squares function minimized:

$$\Sigma w(|F_o|-|F_c|)^2 \quad \text{where w = Least Squares weights.}$$

(4) Standard deviation of an observation of unit weight:

$$[\Sigma w(|F_o|-|F_c|)^2/(N_o-N_v)]^{1/2}$$

where: N_o = number of observations
N_v = number of variables

(5) Cromer, D. T. & Waber, J. T.; "International Tables for X-ray Crystallography", Vol. IV, The Kynoch Press, Birmingham, England, Table 2.2 A (1974).

(6) Ibers, J. A. & Hamilton, W. C.; Acta Crystallogr., 17, 781 (1964).

(7) Creagh, D. C. & McAuley, W.J .; "International Tables for Crystallography", Vol C, (A.J.C. Wilson, ed.), Kluwer Academic Publishers, Boston, Table 4.2.6.8, pages 219-222 (1992).

(8) Creagh, D. C. & Hubbell, J.H..; "International Tables for Crystallography", Vol C, (A.J.C. Wilson, ed.), Kluwer Academic Publishers, Boston, Table 4.2.4.3, pages 200-206 (1992).

(9) CrystalStructure 3.7.0: Crystal Structure Analysis Package, Rigaku and Rigaku/MSC (2000-2005). 9009 New Trails Dr. The Woodlands TX 77381 USA.

(10) CRYSTALS Issue 10: Watkin, D.J., Prout, C.K. Carruthers, J.R. & Betteridge, P.W. Chemical Crystallography Laboratory, Oxford, UK. (1996)

EXPERIMENTAL DETAILS

A. Crystal Data

Empirical Formula	$C_{15}H_{17}N_3O_2S$
Formula Weight	303.38
Crystal Color, Habit	colorless, prism
Crystal Dimensions	0.64 X 0.24 X 0.12 mm
Crystal System	monoclinic
Lattice Type	Primitive
Indexing Images	4 oscillations @ 180.0 seconds
Detector Position	127.40 mm
Pixel Size	0.100 mm
Lattice Parameters	a = 12.9021(7) Å<br>b = 11.1229(6) Å<br>c = 20.2138(14) Å<br>β = 100.079(4) ^{o}<br>V = 2856.1(3) Å^3
Space Group	$P2_1/n$ (#14)
Z value	8
D_{calc}	1.411 g/cm^3
F_{000}	1280.00
μ(CuKα)	20.887 cm^{-1}

B. Intensity Measurements

Diffractometer	Rigaku RAXIS-RAPID
Radiation	CuKα (λ = 1.54187 Å)<br>graphite monochromated
Detector Aperture	280 mm x 256 mm
Data Images	180 exposures
ω oscillation Range (χ=0.0, ϕ=0.0)	20.0 - 200.0^{o}
Exposure Rate	36.0 sec./^{o}
ω oscillation Range (χ=54.0, ϕ=0.0)	20.0 - 200.0^{o}
Exposure Rate	36.0 sec./^{o}
ω oscillation Range (χ=54.0, ϕ=90.0)	20.0 - 200.0^{o}
Exposure Rate	36.0 sec./^{o}
ω oscillation Range (χ=54.0, ϕ=180.0)	20.0 - 200.0^{o}
Exposure Rate	36.0 sec./^{o}
ω oscillation Range (χ=54.0, ϕ=270.0)	20.0 - 200.0^{o}
Exposure Rate	36.0 sec./^{o}
Detector Position	127.40 mm
Pixel Size	0.100 mm
$2\theta_{max}$	143.5^{o}
No. of Reflections Measured	Total: 32046<br>Unique: 5330 (R_{int} = 0.067)
Corrections	Lorentz-polarization<br>Absorption<br>(trans. factors: 0.441 - 0.773)

C. Structure Solution and Refinement

Structure Solution	Direct Methods (SIR92)
Refinement	Full-matrix least-squares on F
Function Minimized	Σ w (|Fo| - |Fc|)^2
Least Squares Weights	1
$2\theta_{max}$ cutoff	143.5^{o}
Anomalous Dispersion	All non-hydrogen atoms
No. Observations (I>2.00σ(I))	24702
No. Variables	413
Reflection/Parameter Ratio	59.81
Residuals: R (I>2.00σ(I))	0.1000
Residuals: Rw (I>2.00σ(I))	0.1295
Goodness of Fit Indicator	7.738
Max Shift/Error in Final Cycle	0.000
Maximum peak in Final Diff. Map	8.13 e^-/Å^3
Minimum peak in Final Diff. Map	-8.21 e^-/Å^3

Table 1. Atomic coordinates and B_{iso}/B_{eq}

atom	x	y	z	B_{eq}
S(1)	0.05601(8)	0.08549(9)	0.64161(6)	1.99(2)
S(2)	0.13417(8)	0.28176(10)	1.01086(6)	2.16(2)
O(1)	0.2098(2)	0.2864(2)	1.07288(14)	3.22(8)
O(3)	-0.0367(2)	0.1059(2)	0.59121(13)	2.60(7)
O(5)	0.0647(2)	-0.0288(2)	0.67514(16)	3.08(7)
O(6)	0.1114(2)	0.3913(2)	0.97348(14)	2.87(7)
N(1)	-0.1232(2)	0.2245(2)	0.70270(17)	2.03(8)
N(8)	0.1600(2)	0.1144(2)	0.60534(17)	2.03(8)
N(9)	0.0251(2)	0.2192(3)	1.02980(18)	2.43(9)
N(10)	0.2584(2)	0.1330(2)	0.64886(19)	2.09(8)
N(11)	0.3650(2)	0.2085(2)	0.98115(17)	2.26(8)
N(12)	-0.0503(2)	0.1733(3)	0.9769(2)	2.71(9)
C(13)	-0.0166(3)	0.2450(3)	0.7231(2)	1.91(10)
C(14)	0.0984(3)	0.3615(3)	0.8102(2)	2.46(11)
C(15)	0.1685(3)	0.1694(3)	0.9585(2)	1.85(10)
C(16)	0.4477(3)	0.1601(3)	0.9547(2)	2.30(11)
C(17)	0.2629(2)	0.1883(3)	0.7038(2)	1.85(10)
C(18)	0.0702(3)	0.1991(3)	0.6997(2)	1.72(9)
C(19)	-0.0022(3)	0.3266(3)	0.7799(2)	1.99(10)
C(20)	0.1733(3)	0.2347(3)	0.7301(2)	1.94(10)
C(21)	-0.1752(3)	0.2909(3)	0.7453(2)	2.51(11)
C(22)	0.2728(3)	0.1535(3)	0.9512(2)	1.98(10)
C(23)	0.2990(3)	0.0702(3)	0.9044(2)	2.12(10)
C(24)	0.0898(3)	0.1027(3)	0.9195(2)	2.17(10)
C(25)	0.4115(3)	0.0749(3)	0.9086(2)	2.24(10)
C(26)	-0.1060(3)	0.3545(3)	0.7933(2)	2.26(11)
C(27)	-0.0204(3)	0.1198(3)	0.9289(2)	2.49(11)
C(28)	0.2182(3)	0.0044(3)	0.8637(2)	2.97(12)
C(29)	0.5595(3)	0.2016(4)	0.9719(2)	3.29(12)
C(30)	0.1166(3)	0.0217(3)	0.8714(2)	2.93(12)
C(31)	0.4815(3)	0.0020(4)	0.8712(2)	3.20(12)
C(32)	0.1835(3)	0.3179(3)	0.7851(2)	2.51(11)
C(33)	0.3723(2)	0.2089(4)	0.7411(2)	3.27(12)
C(34)	0.1693(3)	0.0326(3)	0.5469(2)	3.19(12)
C(35)	-0.1402(3)	0.4288(4)	0.8465(2)	3.18(12)
C(36)	-0.0247(3)	0.2925(4)	1.0770(2)	4.14(14)
C(37)	0.6313(3)	0.1089(4)	0.9468(2)	3.72(13)

Table 1. Atomic coordinates and B_{iso}/B_{eq} (continued)

atom	x	y	z	B_{eq}
C(38)	-0.1063(3)	0.0641(4)	0.8774(2)	4.04(13)
C(39)	-0.2938(3)	0.2891(4)	0.7395(2)	3.47(12)
C(40)	-0.3241(3)	0.3918(4)	0.7866(2)	4.92(16)
C(41)	-0.2521(3)	0.3954(4)	0.8546(2)	4.40(15)
C(42)	0.5870(3)	0.0713(4)	0.8737(2)	3.99(14)
H(1)	0.3706	0.2697	1.0144	2.79
H(2)	0.1077	0.4149	0.8475	2.91
H(3)	0.2353	-0.0515	0.8317	3.61
H(4)	0.0616	-0.0210	0.8438	3.51
H(5)	0.2522	0.3436	0.8047	2.90
H(6)	0.5672	0.2763	0.9505	3.98
H(7)	0.5780	0.2111	1.0192	3.99
H(8)	0.4957	-0.0738	0.8925	3.91
H(9)	0.4476	-0.0097	0.8260	3.90
H(10)	-0.0930	0.4161	0.8876	3.88
H(11)	-0.1390	0.5112	0.8343	3.88
H(12)	0.7004	0.1405	0.9499	4.51
H(13)	0.6334	0.0391	0.9742	4.51
H(14)	-0.3176	0.4668	0.7652	6.06
H(15)	-0.3948	0.3811	0.7928	6.05
H(16)	-0.2509	0.3173	0.8736	5.44
H(17)	-0.2779	0.4513	0.8833	5.43
H(18)	0.6368	0.0233	0.8562	4.97
H(19)	0.5734	0.1424	0.8475	4.97
H(20)	0.3938	0.1434	0.7704	3.88
H(21)	0.3734	0.2809	0.7665	3.87
H(22)	0.4190	0.2165	0.7098	3.87
H(23)	0.2103	-0.0357	0.5629	3.83
H(24)	0.2027	0.0748	0.5155	3.82
H(25)	0.1013	0.0073	0.5255	3.81
H(26)	0.0037	0.2703	1.1219	5.20
H(27)	-0.0985	0.2788	1.0688	5.21
H(28)	-0.0112	0.3752	1.0705	5.19
H(29)	-0.1179	-0.0162	0.8904	4.86
H(30)	-0.0856	0.0639	0.8346	4.86
H(31)	-0.1693	0.1092	0.8752	4.86
H(32)	-0.1547	0.1744	0.6667	2.46

Table 1. Atomic coordinates and B_{iso}/B_{eq} (continued)

atom	x	y	z	B_{eq}
H(33)	-0.3262	0.3024	0.6942	4.23
H(34)	-0.3158	0.2137	0.7541	4.24

$B_{eq} = 8/3\ \pi^2(U_{11}(aa^*)^2 + U_{22}(bb^*)^2 + U_{33}(cc^*)^2 + 2U_{12}(aa^*bb^*)\cos\gamma + 2U_{13}(aa^*cc^*)\cos\beta + 2U_{23}(bb^*cc^*)\cos\alpha)$

Table 2. Anisotropic displacement parameters

atom	U_{11}	U_{22}	U_{33}	U_{12}	U_{13}	U_{23}
S(1)	0.0231(5)	0.0267(6)	0.0258(7)	-0.0014(4)	0.0035(4)	-0.0038(5)
S(2)	0.0253(5)	0.0323(6)	0.0260(7)	0.0004(4)	0.0083(5)	-0.0050(5)
O(1)	0.0320(18)	0.062(2)	0.029(2)	0.0025(15)	0.0087(15)	-0.0212(17)
O(3)	0.0248(16)	0.0466(19)	0.026(2)	-0.0008(13)	-0.0002(14)	-0.0081(15)
O(5)	0.0366(18)	0.0257(17)	0.056(2)	-0.0000(14)	0.0122(16)	0.0101(16)
O(6)	0.0457(19)	0.0274(17)	0.039(2)	0.0027(14)	0.0165(16)	0.0049(15)
N(1)	0.0205(19)	0.030(2)	0.027(2)	-0.0014(15)	0.0065(16)	-0.0154(17)
N(8)	0.0223(19)	0.032(2)	0.023(2)	-0.0015(15)	0.0037(16)	-0.0012(17)
N(9)	0.028(2)	0.038(2)	0.031(2)	-0.0003(17)	0.0177(18)	-0.0001(19)
N(10)	0.0215(19)	0.030(2)	0.027(2)	0.0012(15)	0.0023(17)	0.0029(18)
N(11)	0.0262(19)	0.032(2)	0.029(2)	-0.0014(16)	0.0092(17)	-0.0165(18)
N(12)	0.021(2)	0.041(2)	0.043(2)	-0.0046(16)	0.0083(19)	-0.012(2)
C(13)	0.026(2)	0.025(2)	0.023(3)	-0.0027(18)	0.007(2)	0.002(2)
C(14)	0.032(2)	0.041(2)	0.020(3)	-0.008(2)	0.003(2)	-0.017(2)
C(15)	0.025(2)	0.026(2)	0.021(2)	0.0038(18)	0.009(2)	-0.005(2)
C(16)	0.023(2)	0.037(2)	0.029(3)	-0.002(2)	0.010(2)	-0.008(2)
C(17)	0.019(2)	0.030(2)	0.022(2)	-0.0044(17)	0.0034(19)	0.001(2)
C(18)	0.025(2)	0.025(2)	0.015(2)	-0.0016(18)	0.0038(19)	-0.0069(19)
C(19)	0.029(2)	0.027(2)	0.023(2)	-0.0021(18)	0.012(2)	0.000(2)
C(20)	0.026(2)	0.027(2)	0.020(2)	-0.0049(18)	0.003(2)	0.004(2)
C(21)	0.028(2)	0.033(2)	0.036(3)	0.001(2)	0.011(2)	0.008(2)
C(22)	0.027(2)	0.026(2)	0.023(2)	0.0007(18)	0.007(2)	-0.006(2)
C(23)	0.026(2)	0.029(2)	0.027(3)	0.0008(19)	0.008(2)	-0.007(2)
C(24)	0.022(2)	0.031(2)	0.030(3)	-0.0022(19)	0.005(2)	-0.009(2)
C(25)	0.021(2)	0.034(2)	0.032(3)	-0.0004(19)	0.011(2)	-0.009(2)
C(26)	0.036(2)	0.028(2)	0.023(3)	0.001(2)	0.007(2)	-0.004(2)
C(27)	0.025(2)	0.032(2)	0.036(3)	-0.0046(19)	0.002(2)	-0.008(2)
C(28)	0.035(2)	0.037(2)	0.042(3)	0.000(2)	0.010(2)	-0.022(2)
C(29)	0.027(2)	0.056(3)	0.043(3)	-0.009(2)	0.009(2)	-0.021(2)
C(30)	0.023(2)	0.039(2)	0.049(3)	-0.009(2)	0.006(2)	-0.019(2)
C(31)	0.029(2)	0.048(3)	0.047(3)	0.005(2)	0.011(2)	-0.016(2)
C(32)	0.024(2)	0.047(3)	0.021(3)	-0.010(2)	-0.006(2)	-0.004(2)
C(33)	0.026(2)	0.051(3)	0.044(3)	0.001(2)	-0.002(2)	-0.003(2)
C(34)	0.039(2)	0.051(3)	0.031(3)	0.011(2)	0.006(2)	-0.014(2)
C(35)	0.045(3)	0.047(3)	0.031(3)	0.003(2)	0.012(2)	-0.019(2)
C(36)	0.041(3)	0.071(3)	0.052(4)	-0.008(2)	0.027(2)	-0.031(3)
C(37)	0.021(2)	0.069(3)	0.053(3)	-0.004(2)	0.011(2)	-0.022(3)

Table 2. Anisotropic displacement parameters (continued)

atom	U_{11}	U_{22}	U_{33}	U_{12}	U_{13}	U_{23}
C(38)	0.019(2)	0.062(3)	0.073(4)	-0.002(2)	0.007(2)	-0.024(3)
C(39)	0.022(2)	0.057(3)	0.055(3)	0.001(2)	0.012(2)	-0.007(2)
C(40)	0.037(3)	0.082(4)	0.072(4)	0.018(2)	0.022(3)	-0.025(3)
C(41)	0.054(3)	0.068(4)	0.050(4)	0.013(2)	0.022(3)	-0.024(3)
C(42)	0.022(2)	0.066(3)	0.070(4)	0.005(2)	0.023(2)	-0.007(3)

The general temperature factor expression: $\exp(-2\pi^2(a^{*2}U_{11}h^2 + b^{*2}U_{22}k^2 + c^{*2}U_{33}l^2 + 2a^*b^*U_{12}hk + 2a^*c^*U_{13}hl + 2b^*c^*U_{23}kl))$

Table 3. Bond lengths (Å)

atom	atom	distance	atom	atom	distance
S(1)	O(3)	1.447(2)	S(1)	O(5)	1.435(2)
S(1)	N(8)	1.669(3)	S(1)	C(18)	1.713(4)
S(2)	O(1)	1.449(2)	S(2)	O(6)	1.437(2)
S(2)	N(9)	1.673(3)	S(2)	C(15)	1.744(4)
N(1)	C(13)	1.384(4)	N(1)	C(21)	1.393(5)
N(1)	H(32)	0.950	N(8)	N(10)	1.427(4)
N(8)	C(34)	1.513(5)	N(9)	N(12)	1.410(4)
N(9)	C(36)	1.485(6)	N(10)	C(17)	1.262(5)
N(11)	C(16)	1.384(5)	N(11)	C(22)	1.380(4)
N(11)	H(1)	0.950	N(12)	C(27)	1.255(6)
C(13)	C(18)	1.387(6)	C(13)	C(19)	1.450(5)
C(14)	C(19)	1.390(5)	C(14)	C(32)	1.375(6)
C(14)	H(2)	0.950	C(15)	C(22)	1.391(5)
C(15)	C(24)	1.387(5)	C(16)	C(25)	1.353(5)
C(16)	C(29)	1.496(5)	C(17)	C(20)	1.449(6)
C(17)	C(33)	1.497(5)	C(18)	C(20)	1.421(5)
C(19)	C(26)	1.446(6)	C(20)	C(32)	1.435(6)
C(21)	C(26)	1.392(5)	C(21)	C(39)	1.513(5)
C(22)	C(23)	1.405(6)	C(23)	C(25)	1.439(5)
C(23)	C(28)	1.414(5)	C(24)	C(27)	1.479(6)
C(24)	C(30)	1.412(6)	C(25)	C(31)	1.512(6)
C(26)	C(35)	1.483(6)	C(27)	C(38)	1.514(5)
C(28)	C(30)	1.360(6)	C(28)	H(3)	0.950
C(29)	C(37)	1.531(6)	C(29)	H(6)	0.950
C(29)	H(7)	0.950	C(30)	H(4)	0.950
C(31)	C(42)	1.557(6)	C(31)	H(8)	0.950
C(31)	H(9)	0.950	C(32)	H(5)	0.950
C(33)	H(20)	0.950	C(33)	H(21)	0.950
C(33)	H(22)	0.950	C(34)	H(23)	0.950
C(34)	H(24)	0.950	C(34)	H(25)	0.950
C(35)	C(41)	1.526(6)	C(35)	H(10)	0.950
C(35)	H(11)	0.950	C(36)	H(26)	0.950
C(36)	H(27)	0.950	C(36)	H(28)	0.950
C(37)	C(42)	1.547(7)	C(37)	H(12)	0.950
C(37)	H(13)	0.950	C(38)	H(29)	0.950
C(38)	H(30)	0.950	C(38)	H(31)	0.950
C(39)	C(40)	1.580(7)	C(39)	H(33)	0.950

Table 3. Bond lengths (Å) (continued)

atom	atom	distance	atom	atom	distance
C(39)	H(34)	0.950	C(40)	C(41)	1.518(6)
C(40)	H(14)	0.950	C(40)	H(15)	0.950
C(41)	H(16)	0.950	C(41)	H(17)	0.950
C(42)	H(18)	0.950	C(42)	H(19)	0.950

Table 4. Bond angles (^{o})

atom	atom	atom	angle	atom	atom	atom	angle
O(3)	S(1)	O(5)	117.27(16)	O(3)	S(1)	N(8)	106.83(17)
O(3)	S(1)	C(18)	110.04(17)	O(5)	S(1)	N(8)	112.06(17)
O(5)	S(1)	C(18)	109.82(19)	N(8)	S(1)	C(18)	99.29(18)
O(1)	S(2)	O(6)	117.99(17)	O(1)	S(2)	N(9)	106.44(18)
O(1)	S(2)	C(15)	110.29(18)	O(6)	S(2)	N(9)	111.75(17)
O(6)	S(2)	C(15)	109.80(19)	N(9)	S(2)	C(15)	98.85(19)
C(13)	N(1)	C(21)	106.8(3)	C(13)	N(1)	H(32)	126.5
C(21)	N(1)	H(32)	126.7	S(1)	N(8)	N(10)	117.0(2)
S(1)	N(8)	C(34)	114.0(2)	N(10)	N(8)	C(34)	112.7(3)
S(2)	N(9)	N(12)	118.3(2)	S(2)	N(9)	C(36)	113.2(2)
N(12)	N(9)	C(36)	111.6(3)	N(8)	N(10)	C(17)	120.8(3)
C(16)	N(11)	C(22)	108.7(3)	C(16)	N(11)	H(1)	125.7
C(22)	N(11)	H(1)	125.6	N(9)	N(12)	C(27)	119.5(3)
N(1)	C(13)	C(18)	131.1(3)	N(1)	C(13)	C(19)	108.8(3)
C(18)	C(13)	C(19)	120.1(3)	C(19)	C(14)	C(32)	118.9(3)
C(19)	C(14)	H(2)	120.1	C(32)	C(14)	H(2)	120.9
S(2)	C(15)	C(22)	120.6(2)	S(2)	C(15)	C(24)	119.4(3)
C(22)	C(15)	C(24)	119.8(3)	N(11)	C(16)	C(25)	109.9(3)
N(11)	C(16)	C(29)	124.6(3)	C(25)	C(16)	C(29)	125.4(4)
N(10)	C(17)	C(20)	125.4(3)	N(10)	C(17)	C(33)	114.3(3)
C(20)	C(17)	C(33)	120.2(3)	S(1)	C(18)	C(13)	120.6(2)
S(1)	C(18)	C(20)	118.8(3)	C(13)	C(18)	C(20)	120.0(3)
C(13)	C(19)	C(14)	120.2(3)	C(13)	C(19)	C(26)	106.8(3)
C(14)	C(19)	C(26)	133.0(4)	C(17)	C(20)	C(18)	119.2(3)
C(17)	C(20)	C(32)	122.9(3)	C(18)	C(20)	C(32)	117.8(3)
N(1)	C(21)	C(26)	112.4(3)	N(1)	C(21)	C(39)	122.3(3)
C(26)	C(21)	C(39)	125.3(4)	N(11)	C(22)	C(15)	132.2(3)
N(11)	C(22)	C(23)	107.3(3)	C(15)	C(22)	C(23)	120.5(3)
C(22)	C(23)	C(25)	107.1(3)	C(22)	C(23)	C(28)	119.5(3)
C(25)	C(23)	C(28)	133.3(4)	C(15)	C(24)	C(27)	118.8(3)
C(15)	C(24)	C(30)	119.3(3)	C(27)	C(24)	C(30)	121.9(3)
C(16)	C(25)	C(23)	106.9(3)	C(16)	C(25)	C(31)	123.7(3)
C(23)	C(25)	C(31)	129.5(3)	C(19)	C(26)	C(21)	105.2(3)
C(19)	C(26)	C(35)	131.2(3)	C(21)	C(26)	C(35)	123.5(3)
N(12)	C(27)	C(24)	126.4(3)	N(12)	C(27)	C(38)	115.9(3)
C(24)	C(27)	C(38)	117.6(4)	C(23)	C(28)	C(30)	119.2(4)
C(23)	C(28)	H(3)	119.9	C(30)	C(28)	H(3)	120.9

Table 4. Bond angles (^{o}) (continued)

atom	atom	atom	angle	atom	atom	atom	angle
C(16)	C(29)	C(37)	109.3(3)	C(16)	C(29)	H(6)	109.8
C(16)	C(29)	H(7)	109.3	C(37)	C(29)	H(6)	108.7
C(37)	C(29)	H(7)	110.3	H(6)	C(29)	H(7)	109.5
C(24)	C(30)	C(28)	121.8(3)	C(24)	C(30)	H(4)	118.3
C(28)	C(30)	H(4)	119.9	C(25)	C(31)	C(42)	108.3(3)
C(25)	C(31)	H(8)	109.5	C(25)	C(31)	H(9)	109.9
C(42)	C(31)	H(8)	109.1	C(42)	C(31)	H(9)	110.5
H(8)	C(31)	H(9)	109.5	C(14)	C(32)	C(20)	122.8(3)
C(14)	C(32)	H(5)	119.2	C(20)	C(32)	H(5)	117.9
C(17)	C(33)	H(20)	110.1	C(17)	C(33)	H(21)	109.0
C(17)	C(33)	H(22)	109.3	H(20)	C(33)	H(21)	109.5
H(20)	C(33)	H(22)	109.5	H(21)	C(33)	H(22)	109.5
N(8)	C(34)	H(23)	109.2	N(8)	C(34)	H(24)	109.3
N(8)	C(34)	H(25)	109.9	H(23)	C(34)	H(24)	109.5
H(23)	C(34)	H(25)	109.5	H(24)	C(34)	H(25)	109.5
C(26)	C(35)	C(41)	110.4(3)	C(26)	C(35)	H(10)	108.9
C(26)	C(35)	H(11)	109.2	C(41)	C(35)	H(10)	110.1
C(41)	C(35)	H(11)	108.7	H(10)	C(35)	H(11)	109.5
N(9)	C(36)	H(26)	109.5	N(9)	C(36)	H(27)	109.4
N(9)	C(36)	H(28)	109.4	H(26)	C(36)	H(27)	109.5
H(26)	C(36)	H(28)	109.5	H(27)	C(36)	H(28)	109.5
C(29)	C(37)	C(42)	110.7(3)	C(29)	C(37)	H(12)	110.2
C(29)	C(37)	H(13)	108.4	C(42)	C(37)	H(12)	110.4
C(42)	C(37)	H(13)	107.6	H(12)	C(37)	H(13)	109.5
C(27)	C(38)	H(29)	109.1	C(27)	C(38)	H(30)	109.8
C(27)	C(38)	H(31)	109.5	H(29)	C(38)	H(30)	109.5
H(29)	C(38)	H(31)	109.5	H(30)	C(38)	H(31)	109.5
C(21)	C(39)	C(40)	107.3(3)	C(21)	C(39)	H(33)	110.0
C(21)	C(39)	H(34)	110.0	C(40)	C(39)	H(33)	110.8
C(40)	C(39)	H(34)	109.4	H(33)	C(39)	H(34)	109.5
C(39)	C(40)	C(41)	112.8(3)	C(39)	C(40)	H(14)	108.1
C(39)	C(40)	H(15)	109.5	C(41)	C(40)	H(14)	107.4
C(41)	C(40)	H(15)	109.5	H(14)	C(40)	H(15)	109.5
C(35)	C(41)	C(40)	110.5(4)	C(35)	C(41)	H(16)	108.5
C(35)	C(41)	H(17)	110.2	C(40)	C(41)	H(16)	107.9
C(40)	C(41)	H(17)	110.3	H(16)	C(41)	H(17)	109.5
C(31)	C(42)	C(37)	109.8(4)	C(31)	C(42)	H(18)	110.7

Table 4. Bond angles (^{o}) (continued)

atom	atom	atom	angle	atom	atom	atom	angle
C(31)	C(42)	H(19)	108.6	C(37)	C(42)	H(18)	110.2
C(37)	C(42)	H(19)	108.0	H(18)	C(42)	H(19)	109.5

Table 5. Torsion Angles(o)

atom1	atom2	atom3	atom4	angle
O(3)	S(1)	N(8)	N(10)	163.1(2)
O(3)	S(1)	C(18)	C(13)	41.0(3)
O(5)	S(1)	N(8)	N(10)	-67.2(3)
O(5)	S(1)	C(18)	C(13)	-89.5(3)
N(8)	S(1)	C(18)	C(13)	152.9(3)
C(18)	S(1)	N(8)	N(10)	48.8(2)
O(1)	S(2)	N(9)	N(12)	-162.8(2)
O(1)	S(2)	C(15)	C(22)	-42.2(4)
O(6)	S(2)	N(9)	N(12)	67.1(3)
O(6)	S(2)	C(15)	C(22)	89.4(3)
N(9)	S(2)	C(15)	C(22)	-153.5(3)
C(15)	S(2)	N(9)	N(12)	-48.4(3)
C(13)	N(1)	C(21)	C(26)	-0.1(3)
C(21)	N(1)	C(13)	C(18)	177.9(4)
S(1)	N(8)	N(10)	C(17)	-37.4(4)
S(2)	N(9)	N(12)	C(27)	38.8(4)
N(8)	N(10)	C(17)	C(20)	2.1(6)
C(16)	N(11)	C(22)	C(15)	-178.9(4)
C(22)	N(11)	C(16)	C(25)	-0.3(4)
N(9)	N(12)	C(27)	C(24)	-2.8(6)
N(1)	C(13)	C(18)	S(1)	-7.9(6)
N(1)	C(13)	C(19)	C(14)	180(179)
C(18)	C(13)	C(19)	C(14)	1.6(6)
C(19)	C(13)	C(18)	S(1)	169.8(3)
C(19)	C(14)	C(32)	C(20)	-1.9(6)
C(32)	C(14)	C(19)	C(26)	179.8(4)
S(2)	C(15)	C(22)	C(23)	-174.3(3)
S(2)	C(15)	C(24)	C(30)	172.6(3)
C(22)	C(15)	C(24)	C(30)	-1.8(6)
C(24)	C(15)	C(22)	C(23)	0.1(4)
N(11)	C(16)	C(25)	C(31)	-178.5(3)
C(25)	C(16)	C(29)	C(37)	-17.5(6)
C(29)	C(16)	C(25)	C(31)	4.8(7)
N(10)	C(17)	C(20)	C(32)	-166.5(4)
C(33)	C(17)	C(20)	C(32)	10.8(6)
S(1)	C(18)	C(20)	C(32)	-171.5(3)
C(13)	C(18)	C(20)	C(32)	0.4(5)

atom1	atom2	atom3	atom4	angle
O(3)	S(1)	N(8)	C(34)	-62.4(2)
O(3)	S(1)	C(18)	C(20)	-147.2(3)
O(5)	S(1)	N(8)	C(34)	67.3(2)
O(5)	S(1)	C(18)	C(20)	82.3(3)
N(8)	S(1)	C(18)	C(20)	-35.3(3)
C(18)	S(1)	N(8)	C(34)	-176.8(2)
O(1)	S(2)	N(9)	C(36)	64.0(3)
O(1)	S(2)	C(15)	C(24)	143.4(3)
O(6)	S(2)	N(9)	C(36)	-66.1(3)
O(6)	S(2)	C(15)	C(24)	-84.9(3)
N(9)	S(2)	C(15)	C(24)	32.1(3)
C(15)	S(2)	N(9)	C(36)	178.3(2)
C(13)	N(1)	C(21)	C(39)	-178.6(3)
C(21)	N(1)	C(13)	C(19)	-0.1(3)
C(34)	N(8)	N(10)	C(17)	-172.4(3)
C(36)	N(9)	N(12)	C(27)	172.7(4)
N(8)	N(10)	C(17)	C(33)	-175.4(3)
C(16)	N(11)	C(22)	C(23)	-0.8(4)
C(22)	N(11)	C(16)	C(29)	176.4(4)
N(9)	N(12)	C(27)	C(38)	174.8(3)
N(1)	C(13)	C(18)	C(20)	-179.7(4)
N(1)	C(13)	C(19)	C(26)	0.2(4)
C(18)	C(13)	C(19)	C(26)	-178.0(3)
C(19)	C(13)	C(18)	C(20)	-1.9(5)
C(32)	C(14)	C(19)	C(13)	0.3(5)
S(2)	C(15)	C(22)	N(11)	3.6(6)
S(2)	C(15)	C(24)	C(27)	-6.5(5)
C(22)	C(15)	C(24)	C(27)	179.1(3)
C(24)	C(15)	C(22)	N(11)	177.9(4)
N(11)	C(16)	C(25)	C(23)	1.2(4)
N(11)	C(16)	C(29)	C(37)	166.3(4)
C(29)	C(16)	C(25)	C(23)	-175.4(4)
N(10)	C(17)	C(20)	C(18)	12.1(6)
C(33)	C(17)	C(20)	C(18)	-170.5(3)
S(1)	C(18)	C(20)	C(17)	9.8(5)
C(13)	C(18)	C(20)	C(17)	-178.3(3)
C(13)	C(19)	C(26)	C(21)	-0.3(4)

Table 5. Torsion angles (^{o}) (continued)

atom1	atom2	atom3	atom4	angle	atom1	atom2	atom3	atom4	angle
C(13)	C(19)	C(26)	C(35)	177.4(4)	C(14)	C(19)	C(26)	C(21)	-179.8(4)
C(14)	C(19)	C(26)	C(35)	-2.2(8)	C(17)	C(20)	C(32)	C(14)	-179.8(4)
C(18)	C(20)	C(32)	C(14)	1.6(6)	N(1)	C(21)	C(26)	C(19)	0.2(4)
N(1)	C(21)	C(26)	C(35)	-177.6(3)	N(1)	C(21)	C(39)	C(40)	-169.9(4)
C(26)	C(21)	C(39)	C(40)	11.7(6)	C(39)	C(21)	C(26)	C(19)	178.7(4)
C(39)	C(21)	C(26)	C(35)	0.9(7)	N(11)	C(22)	C(23)	C(25)	1.5(4)
N(11)	C(22)	C(23)	C(28)	-177.0(3)	C(15)	C(22)	C(23)	C(25)	179.9(3)
C(15)	C(22)	C(23)	C(28)	1.3(6)	C(22)	C(23)	C(25)	C(16)	-1.7(4)
C(22)	C(23)	C(25)	C(31)	178.0(4)	C(22)	C(23)	C(28)	C(30)	-1.0(6)
C(25)	C(23)	C(28)	C(30)	-179.0(4)	C(28)	C(23)	C(25)	C(16)	176.6(4)
C(28)	C(23)	C(25)	C(31)	-3.7(8)	C(15)	C(24)	C(27)	N(12)	-13.5(6)
C(15)	C(24)	C(27)	C(38)	168.8(3)	C(15)	C(24)	C(30)	C(28)	2.2(6)
C(27)	C(24)	C(30)	C(28)	-178.7(4)	C(30)	C(24)	C(27)	N(12)	167.3(4)
C(30)	C(24)	C(27)	C(38)	-10.3(6)	C(16)	C(25)	C(31)	C(42)	-20.9(6)
C(23)	C(25)	C(31)	C(42)	159.4(4)	C(19)	C(26)	C(35)	C(41)	-159.9(4)
C(21)	C(26)	C(35)	C(41)	17.4(5)	C(23)	C(28)	C(30)	C(24)	-0.8(6)
C(16)	C(29)	C(37)	C(42)	47.0(5)	C(25)	C(31)	C(42)	C(37)	49.8(4)
C(26)	C(35)	C(41)	C(40)	-48.6(5)	C(29)	C(37)	C(42)	C(31)	-66.4(4)
C(21)	C(39)	C(40)	C(41)	-43.7(5)	C(39)	C(40)	C(41)	C(35)	65.1(5)

The sign is positive if when looking from atom 2 to atom 3 a clock-wise motion of atom 1 would superimpose it on atom 4.

Table 6. Distances beyond the asymmetric unit out to 3.60 Å

atom	atom	distance	atom	atom	distance
S(1)	H(1)[1]	3.574	S(1)	H(7)[1]	3.402
S(1)	H(15)[2]	3.495	S(2)	H(2)	3.581
S(2)	H(23)[3]	3.380	S(2)	H(29)[4]	3.591
O(1)	O(3)[5]	3.441(3)	O(1)	N(1)[5]	3.092(4)
O(1)	C(39)[5]	3.479(5)	O(1)	H(11)[6]	3.165
O(1)	H(17)[6]	3.129	O(1)	H(29)[4]	3.362
O(1)	H(32)[5]	2.383	O(1)	H(33)[5]	2.757
O(3)	O(1)[1]	3.441(3)	O(3)	N(11)[1]	3.135(4)
O(3)	C(34)[7]	3.379(4)	O(3)	H(1)[1]	2.259
O(3)	H(6)[1]	3.597	O(3)	H(7)[1]	3.035
O(3)	H(17)[2]	3.062	O(3)	H(23)[7]	3.591
O(3)	H(24)[7]	3.415	O(3)	H(25)[7]	2.676
O(5)	C(33)[8]	3.400(5)	O(5)	C(40)[2]	3.443(5)
O(5)	H(3)	3.534	O(5)	H(4)	3.418
O(5)	H(5)[8]	2.724	O(5)	H(6)[8]	3.540
O(5)	H(15)[2]	2.594	O(5)	H(21)[8]	2.486
O(6)	C(14)	3.293(5)	O(6)	C(34)[3]	3.324(5)
O(6)	H(2)	2.553	O(6)	H(10)	2.906
O(6)	H(10)[6]	3.573	O(6)	H(23)[3]	2.662
O(6)	H(24)[3]	3.128	O(6)	H(28)[6]	2.967
N(1)	O(1)[1]	3.092(4)	N(1)	H(8)[3]	3.543
N(1)	H(14)[2]	3.065	N(1)	H(18)[3]	3.524
N(1)	H(30)	3.175	N(8)	C(29)[1]	3.453(5)
N(8)	H(5)[8]	3.597	N(8)	H(6)[1]	3.376
N(8)	H(7)[1]	2.698	N(8)	H(27)[9]	3.531
N(9)	H(29)[4]	2.908	N(9)	H(33)[5]	3.542
N(10)	C(14)[8]	3.562(5)	N(10)	C(36)[9]	3.472(6)
N(10)	H(2)[8]	2.972	N(10)	H(3)[3]	3.531
N(10)	H(5)[8]	3.363	N(10)	H(26)[9]	3.475
N(10)	H(27)[9]	2.835	N(11)	O(3)[5]	3.135(4)
N(11)	H(8)[10]	3.223	N(11)	H(13)[10]	2.898
N(11)	H(23)[3]	3.087	N(11)	H(25)[3]	3.357
N(12)	H(10)	3.241	N(12)	H(12)[11]	3.187
N(12)	H(16)	3.424	N(12)	H(29)[4]	3.595
C(13)	C(31)[3]	3.509(6)	C(13)	H(8)[3]	3.133
C(13)	H(9)[3]	3.087	C(13)	H(30)	3.260
C(14)	O(6)	3.293(5)	C(14)	N(10)[3]	3.562(5)

Table 6. Distances beyond the asymmetric unit out to 3.60 Å (continued)

atom	atom	distance	atom	atom	distance
C(14)	H(9)[3]	3.068	C(14)	H(20)[3]	3.543
C(14)	H(23)[3]	3.429	C(15)	H(2)	3.534
C(15)	H(13)[10]	3.542	C(16)	H(8)[10]	3.194
C(16)	H(13)[10]	2.933	C(17)	H(3)[3]	2.983
C(17)	H(27)[9]	3.534	C(18)	H(8)[3]	3.166
C(18)	H(9)[3]	3.282	C(19)	H(9)[3]	2.988
C(19)	H(30)	3.366	C(20)	H(3)[3]	3.021
C(20)	H(9)[3]	3.344	C(21)	H(18)[3]	3.391
C(21)	H(30)	3.200	C(21)	H(31)	3.303
C(22)	H(13)[10]	2.773	C(22)	H(23)[3]	3.479
C(23)	C(37)[10]	3.588(6)	C(23)	H(13)[10]	2.741
C(23)	H(20)	3.263	C(25)	H(7)[10]	3.492
C(25)	H(13)[10]	2.835	C(25)	H(20)	2.866
C(26)	H(18)[3]	3.521	C(26)	H(30)	3.338
C(26)	H(31)	3.366	C(27)	H(10)	3.488
C(28)	H(13)[10]	3.527	C(28)	H(20)	3.546
C(29)	N(8)[5]	3.453(5)	C(29)	C(34)[5]	3.506(6)
C(29)	H(8)[10]	3.275	C(29)	H(24)[5]	3.132
C(29)	H(25)[5]	3.427	C(30)	H(27)[4]	3.576
C(31)	C(13)[8]	3.509(6)	C(31)	C(33)	3.593(6)
C(31)	H(7)[10]	3.422	C(31)	H(20)	2.664
C(32)	H(3)[3]	3.108	C(32)	H(9)[3]	3.203
C(32)	H(23)[3]	3.533	C(33)	O(5)[3]	3.400(5)
C(33)	C(31)	3.593(6)	C(33)	H(3)[3]	3.238
C(33)	H(9)	3.038	C(33)	H(15)[12]	3.563
C(33)	H(19)	3.155	C(33)	H(26)[9]	3.187
C(33)	H(27)[9]	3.571	C(34)	O(3)[7]	3.379(4)
C(34)	O(6)[8]	3.324(5)	C(34)	C(29)[1]	3.506(6)
C(34)	H(1)[8]	3.181	C(34)	H(2)[8]	3.522
C(34)	H(6)[1]	3.025	C(34)	H(7)[1]	3.099
C(34)	H(17)[9]	3.495	C(34)	H(25)[7]	3.569
C(35)	H(28)[6]	3.199	C(36)	N(10)[13]	3.472(6)
C(36)	H(4)[4]	3.489	C(36)	H(10)[6]	3.598
C(36)	H(11)[6]	3.334	C(36)	H(22)[13]	2.903
C(36)	H(29)[4]	3.583	C(36)	H(33)[5]	3.340
C(37)	C(23)[10]	3.588(6)	C(37)	H(16)[12]	3.265
C(37)	H(31)[12]	3.165	C(38)	H(12)[11]	3.220

Table 6. Distances beyond the asymmetric unit out to 3.60 Å (continued)

atom	atom	distance	atom	atom	distance
C(38)	H(14)[2]	3.075	C(38)	H(16)	3.372
C(38)	H(18)[11]	3.299	C(38)	H(33)[2]	3.299
C(39)	O(1)[1]	3.479(5)	C(39)	H(11)[2]	3.475
C(39)	H(19)[11]	3.418	C(39)	H(26)[1]	3.277
C(39)	H(29)[14]	3.441	C(39)	H(31)	3.545
C(40)	O(5)[14]	3.443(5)	C(40)	H(19)[11]	3.396
C(40)	H(30)[14]	3.165	C(40)	H(32)[14]	3.307
C(41)	H(6)[11]	3.540	C(41)	H(12)[11]	3.542
C(41)	H(19)[11]	3.591	C(41)	H(24)[13]	3.421
C(41)	H(31)	3.361	C(41)	H(32)[14]	3.328
C(42)	H(16)[12]	3.444	C(42)	H(20)	3.064
C(42)	H(31)[12]	3.167	C(42)	H(34)[12]	3.313
H(1)	S(1)[5]	3.574	H(1)	O(3)[5]	2.259
H(1)	C(34)[3]	3.181	H(1)	H(8)[10]	3.183
H(1)	H(13)[10]	3.445	H(1)	H(23)[3]	2.764
H(1)	H(24)[3]	3.545	H(1)	H(25)[3]	2.804
H(1)	H(32)[5]	3.214	H(2)	S(2)	3.581
H(2)	O(6)	2.553	H(2)	N(10)[3]	2.972
H(2)	C(15)	3.534	H(2)	C(34)[3]	3.522
H(2)	H(9)[3]	3.555	H(2)	H(20)[3]	3.482
H(2)	H(22)[3]	3.546	H(2)	H(23)[3]	2.758
H(2)	H(28)[6]	3.237	H(3)	O(5)	3.534
H(3)	N(10)[8]	3.531	H(3)	C(17)[8]	2.983
H(3)	C(20)[8]	3.021	H(3)	C(32)[8]	3.108
H(3)	C(33)[8]	3.238	H(3)	H(5)[8]	3.024
H(3)	H(20)	3.362	H(3)	H(21)[8]	2.900
H(3)	H(22)[8]	3.279	H(4)	O(5)	3.418
H(4)	C(36)[4]	3.489	H(4)	H(14)[2]	3.515
H(4)	H(15)[2]	3.371	H(4)	H(21)[8]	3.344
H(4)	H(22)[8]	3.140	H(4)	H(26)[4]	3.014
H(4)	H(27)[4]	3.358	H(4)	H(33)[2]	3.585
H(5)	O(5)[3]	2.724	H(5)	N(8)[3]	3.597
H(5)	N(10)[3]	3.363	H(5)	H(3)[3]	3.024
H(5)	H(23)[3]	2.956	H(6)	O(3)[5]	3.597
H(6)	O(5)[3]	3.540	H(6)	N(8)[5]	3.376
H(6)	C(34)[5]	3.025	H(6)	C(41)[12]	3.540
H(6)	H(15)[12]	3.507	H(6)	H(16)[12]	3.067

Table 6. Distances beyond the asymmetric unit out to 3.60 Å (continued)

atom	atom	distance	atom	atom	distance
H(6)	H(17)$^{12)}$	3.252	H(6)	H(24)$^{5)}$	2.592
H(6)	H(25)$^{3)}$	3.454	H(6)	H(25)$^{5)}$	2.838
H(7)	S(1)$^{5)}$	3.402	H(7)	O(3)$^{5)}$	3.035
H(7)	N(8)$^{5)}$	2.698	H(7)	C(25)$^{10)}$	3.492
H(7)	C(31)$^{10)}$	3.422	H(7)	C(34)$^{5)}$	3.099
H(7)	H(8)$^{10)}$	2.649	H(7)	H(24)$^{5)}$	2.882
H(7)	H(25)$^{5)}$	3.148	H(8)	N(1)$^{8)}$	3.543
H(8)	N(11)$^{10)}$	3.223	H(8)	C(13)$^{8)}$	3.133
H(8)	C(16)$^{10)}$	3.194	H(8)	C(18)$^{8)}$	3.166
H(8)	C(29)$^{10)}$	3.275	H(8)	H(1)$^{10)}$	3.183
H(8)	H(7)$^{10)}$	2.649	H(8)	H(13)$^{10)}$	3.429
H(8)	H(20)	3.538	H(9)	C(13)$^{8)}$	3.087
H(9)	C(14)$^{8)}$	3.068	H(9)	C(18)$^{8)}$	3.282
H(9)	C(19)$^{8)}$	2.988	H(9)	C(20)$^{8)}$	3.344
H(9)	C(32)$^{8)}$	3.203	H(9)	C(33)	3.038
H(9)	H(2)$^{8)}$	3.555	H(9)	H(20)	2.092
H(9)	H(21)	3.524	H(9)	H(22)	3.418
H(10)	O(6)	2.906	H(10)	O(6)$^{6)}$	3.573
H(10)	N(12)	3.241	H(10)	C(27)	3.488
H(10)	C(36)$^{6)}$	3.598	H(10)	H(28)$^{6)}$	2.741
H(10)	H(31)	3.550	H(11)	O(1)$^{6)}$	3.165
H(11)	C(36)$^{6)}$	3.334	H(11)	C(39)$^{14)}$	3.475
H(11)	H(26)$^{6)}$	3.032	H(11)	H(28)$^{6)}$	2.783
H(11)	H(32)$^{14)}$	3.218	H(11)	H(33)$^{14)}$	3.307
H(11)	H(34)$^{14)}$	2.871	H(12)	N(12)$^{12)}$	3.187
H(12)	C(38)$^{12)}$	3.220	H(12)	C(41)$^{12)}$	3.542
H(12)	H(16)$^{12)}$	2.642	H(12)	H(24)$^{5)}$	3.431
H(12)	H(27)$^{12)}$	3.562	H(12)	H(29)$^{12)}$	3.311
H(12)	H(31)$^{12)}$	2.473	H(13)	N(11)$^{10)}$	2.898
H(13)	C(15)$^{10)}$	3.542	H(13)	C(16)$^{10)}$	2.933
H(13)	C(22)$^{10)}$	2.773	H(13)	C(23)$^{10)}$	2.741
H(13)	C(25)$^{10)}$	2.835	H(13)	C(28)$^{10)}$	3.527
H(13)	H(1)$^{10)}$	3.445	H(13)	H(8)$^{10)}$	3.429
H(13)	H(31)$^{12)}$	3.587	H(14)	N(1)$^{14)}$	3.065
H(14)	C(38)$^{14)}$	3.075	H(14)	H(4)$^{14)}$	3.515
H(14)	H(29)$^{14)}$	3.116	H(14)	H(30)$^{14)}$	2.431
H(14)	H(31)$^{14)}$	3.229	H(14)	H(32)$^{14)}$	2.743

Table 6. Distances beyond the asymmetric unit out to 3.60 Å (continued)

atom	atom	distance	atom	atom	distance
H(14)	H(34)[14]	3.300	H(15)	S(1)[14]	3.495
H(15)	O(5)[14]	2.594	H(15)	C(33)[11]	3.563
H(15)	H(4)[14]	3.371	H(15)	H(6)[11]	3.507
H(15)	H(19)[11]	2.933	H(15)	H(21)[11]	3.148
H(15)	H(22)[11]	3.245	H(15)	H(30)[14]	3.255
H(15)	H(32)[14]	3.396	H(16)	N(12)	3.424
H(16)	C(37)[11]	3.265	H(16)	C(38)	3.372
H(16)	C(42)[11]	3.444	H(16)	H(6)[11]	3.067
H(16)	H(12)[11]	2.642	H(16)	H(18)[11]	3.570
H(16)	H(19)[11]	2.964	H(16)	H(24)[13]	3.260
H(16)	H(31)	2.541	H(17)	O(1)[6]	3.129
H(17)	O(3)[14]	3.062	H(17)	C(34)[13]	3.495
H(17)	H(6)[11]	3.252	H(17)	H(24)[13]	2.743
H(17)	H(25)[13]	3.532	H(17)	H(32)[14]	2.761
H(18)	N(1)[8]	3.524	H(18)	C(21)[8]	3.391
H(18)	C(26)[8]	3.521	H(18)	C(38)[12]	3.299
H(18)	H(16)[12]	3.570	H(18)	H(20)	3.567
H(18)	H(29)[12]	3.150	H(18)	H(31)[12]	2.642
H(18)	H(34)[12]	3.092	H(19)	C(33)	3.155
H(19)	C(39)[12]	3.418	H(19)	C(40)[12]	3.396
H(19)	C(41)[12]	3.591	H(19)	H(15)[12]	2.933
H(19)	H(16)[12]	2.964	H(19)	H(20)	2.556
H(19)	H(21)	3.199	H(19)	H(22)	3.233
H(19)	H(31)[12]	3.289	H(19)	H(34)[12]	2.679
H(20)	C(14)[8]	3.543	H(20)	C(23)	3.263
H(20)	C(25)	2.866	H(20)	C(28)	3.546
H(20)	C(31)	2.664	H(20)	C(42)	3.064
H(20)	H(2)[8]	3.482	H(20)	H(3)	3.362
H(20)	H(8)	3.538	H(20)	H(9)	2.092
H(20)	H(18)	3.567	H(20)	H(19)	2.556
H(21)	O(5)[3]	2.486	H(21)	H(3)[3]	2.900
H(21)	H(4)[3]	3.344	H(21)	H(9)	3.524
H(21)	H(15)[12]	3.148	H(21)	H(19)	3.199
H(22)	C(36)[9]	2.903	H(22)	H(2)[8]	3.546
H(22)	H(3)[3]	3.279	H(22)	H(4)[3]	3.140
H(22)	H(9)	3.418	H(22)	H(15)[12]	3.245
H(22)	H(19)	3.233	H(22)	H(26)[9]	2.247

Table 6. Distances beyond the asymmetric unit out to 3.60 Å (continued)

atom	atom	distance	atom	atom	distance
H(22)	H(27)[9]	2.821	H(22)	H(28)[9]	3.266
H(22)	H(33)[12]	3.491	H(22)	H(34)[12]	3.382
H(23)	S(2)[8]	3.380	H(23)	O(3)[7]	3.591
H(23)	O(6)[8]	2.662	H(23)	N(11)[8]	3.087
H(23)	C(14)[8]	3.429	H(23)	C(22)[8]	3.479
H(23)	C(32)[8]	3.533	H(23)	H(1)[8]	2.764
H(23)	H(2)[8]	2.758	H(23)	H(5)[8]	2.956
H(24)	O(3)[7]	3.415	H(24)	O(6)[8]	3.128
H(24)	C(29)[1]	3.132	H(24)	C(41)[9]	3.421
H(24)	H(1)[8]	3.545	H(24)	H(6)[1]	2.592
H(24)	H(7)[1]	2.882	H(24)	H(12)[1]	3.431
H(24)	H(16)[9]	3.260	H(24)	H(17)[9]	2.743
H(24)	H(27)[9]	3.070	H(25)	O(3)[7]	2.676
H(25)	N(11)[8]	3.357	H(25)	C(29)[1]	3.427
H(25)	C(34)[7]	3.569	H(25)	H(1)[8]	2.804
H(25)	H(6)[8]	3.454	H(25)	H(6)[1]	2.838
H(25)	H(7)[1]	3.148	H(25)	H(17)[9]	3.532
H(25)	H(25)[7]	2.642	H(26)	N(10)[13]	3.475
H(26)	C(33)[13]	3.187	H(26)	C(39)[5]	3.277
H(26)	H(4)[4]	3.014	H(26)	H(11)[6]	3.032
H(26)	H(22)[13]	2.247	H(26)	H(29)[4]	3.218
H(26)	H(33)[5]	2.546	H(26)	H(34)[5]	3.227
H(27)	N(8)[13]	3.531	H(27)	N(10)[13]	2.835
H(27)	C(17)[13]	3.534	H(27)	C(30)[4]	3.576
H(27)	C(33)[13]	3.571	H(27)	H(4)[4]	3.358
H(27)	H(12)[11]	3.562	H(27)	H(22)[13]	2.821
H(27)	H(24)[13]	3.070	H(28)	O(6)[6]	2.967
H(28)	C(35)[6]	3.199	H(28)	H(2)[6]	3.237
H(28)	H(10)[6]	2.741	H(28)	H(11)[6]	2.783
H(28)	H(22)[13]	3.266	H(29)	S(2)[4]	3.591
H(29)	O(1)[4]	3.362	H(29)	N(9)[4]	2.908
H(29)	N(12)[4]	3.595	H(29)	C(36)[4]	3.583
H(29)	C(39)[2]	3.441	H(29)	H(12)[11]	3.311
H(29)	H(14)[2]	3.116	H(29)	H(18)[11]	3.150
H(29)	H(26)[4]	3.218	H(29)	H(33)[2]	2.661
H(30)	N(1)	3.175	H(30)	C(13)	3.260
H(30)	C(19)	3.366	H(30)	C(21)	3.200

Table 6. Distances beyond the asymmetric unit out to 3.60 Å (continued)

atom	atom	distance	atom	atom	distance
H(30)	C(26)	3.338	H(30)	C(40)[2]	3.165
H(30)	H(14)[2]	2.431	H(30)	H(15)[2]	3.255
H(30)	H(32)	3.573	H(30)	H(33)[2]	3.139
H(30)	H(34)	3.543	H(31)	C(21)	3.303
H(31)	C(26)	3.366	H(31)	C(37)[11]	3.165
H(31)	C(39)	3.545	H(31)	C(41)	3.361
H(31)	C(42)[11]	3.167	H(31)	H(10)	3.550
H(31)	H(12)[11]	2.473	H(31)	H(13)[11]	3.587
H(31)	H(14)[2]	3.229	H(31)	H(16)	2.541
H(31)	H(18)[11]	2.642	H(31)	H(19)[11]	3.289
H(31)	H(34)	3.049	H(32)	O(1)[1]	2.383
H(32)	C(40)[2]	3.307	H(32)	C(41)[2]	3.328
H(32)	H(1)[1]	3.214	H(32)	H(11)[2]	3.218
H(32)	H(14)[2]	2.743	H(32)	H(15)[2]	3.396
H(32)	H(17)[2]	2.761	H(32)	H(30)	3.573
H(33)	O(1)[1]	2.757	H(33)	N(9)[1]	3.542
H(33)	C(36)[1]	3.340	H(33)	C(38)[14]	3.299
H(33)	H(4)[14]	3.585	H(33)	H(11)[2]	3.307
H(33)	H(22)[11]	3.491	H(33)	H(26)[1]	2.546
H(33)	H(29)[14]	2.661	H(33)	H(30)[14]	3.139
H(34)	C(42)[11]	3.313	H(34)	H(11)[2]	2.871
H(34)	H(14)[2]	3.300	H(34)	H(18)[11]	3.092
H(34)	H(19)[11]	2.679	H(34)	H(22)[11]	3.382
H(34)	H(26)[1]	3.227	H(34)	H(30)	3.543
H(34)	H(31)	3.049			

Symmetry Operators:

(1) X+1/2-1,-Y+1/2,Z+1/2-1
(2) -X+1/2-1,Y+1/2-1,-Z+1/2+1
(3) -X+1/2,Y+1/2,-Z+1/2+1
(4) -X,-Y,-Z+2
(5) X+1/2,-Y+1/2,Z+1/2
(6) -X,-Y+1,-Z+2
(7) -X,-Y,-Z+1
(8) -X+1/2,Y+1/2-1,-Z+1/2+1
(9) X+1/2,-Y+1/2,Z+1/2-1
(10) -X+1,-Y,-Z+2
(11) X-1,Y,Z
(12) X+1,Y,Z
(13) X+1/2-1,-Y+1/2,Z+1/2
(14) -X+1/2-1,Y+1/2,-Z+1/2+1

Table 7. Intramolecular and Intermolecular Hydrogen bonds

D	H	A	D...A	D-H	H...A	D-H...A
N(1)	H(32)	O(1)[4:-1:0:-1]	3.092(4)	0.950	2.383	131.1
N(1)	H(32)	O(3)	2.991(4)	0.950	2.457	115.4
N(11)	H(1)	O(1)	3.082(4)	0.950	2.569	114.2
N(11)	H(1)	O(3)[4:0:0:0]	3.135(4)	0.950	2.259	152.9

Note) 1. The symmetry operations are applied to the acceptors.
2. Estimated standard deviations (esd's) are shown in the parentheses.
They are not calculated when all atoms have an esd=0.0.
