# Supplementary material for: Thiadiazino-indole, thiadiazino-carbazole and benzothiadiazino-carbazole dioxides: synthesis, physicochemical and early ADME characterization of representatives of new tri-, tetra- and pentacyclic ring systems and their intermediates
Source: Beilstein J Org Chem. 2025 Oct 21;21:2220–33. doi: 10.3762/bjoc.21.169 (PMC12557438; doi:10.3762/bjoc.21.169)
Supplement: File 2 — Crystallographic information files, checkcif and structure report files for compounds 3b, 3d, 3e, 3g, 3h, (E)-7a, 7b, 7d, 7e, (E)-7f, (Z)-7h, 7i and (E)-9a. [file Beilstein_J_Org_Chem-21-2220-s002.zip › Átnevezett XRD/7h_xrd.pdf]

# 143768

# PGY0792_1

Submitted by: Pusztai Gyongyver
Operator: Dancso Andras

X-ray Structure Report

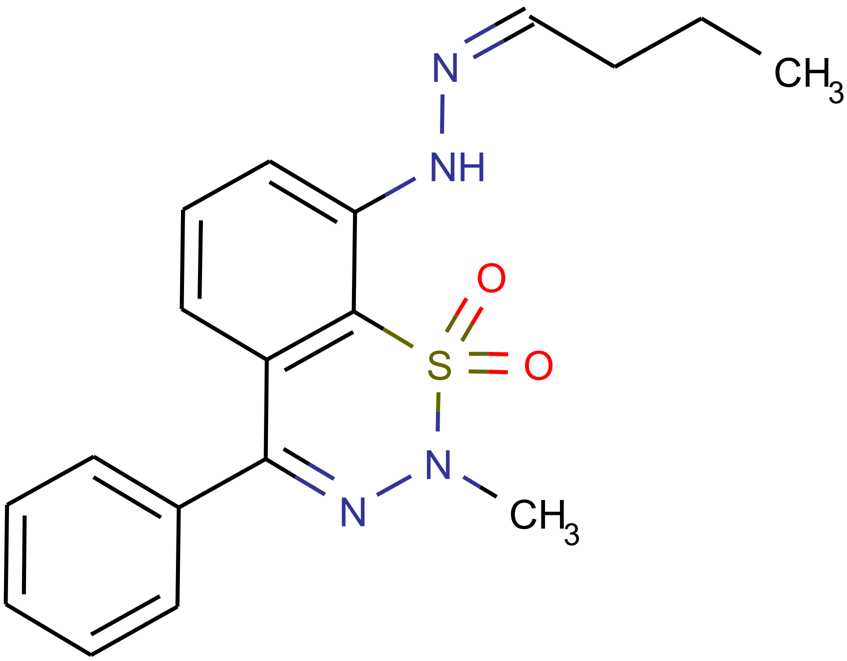


December 2, 2024

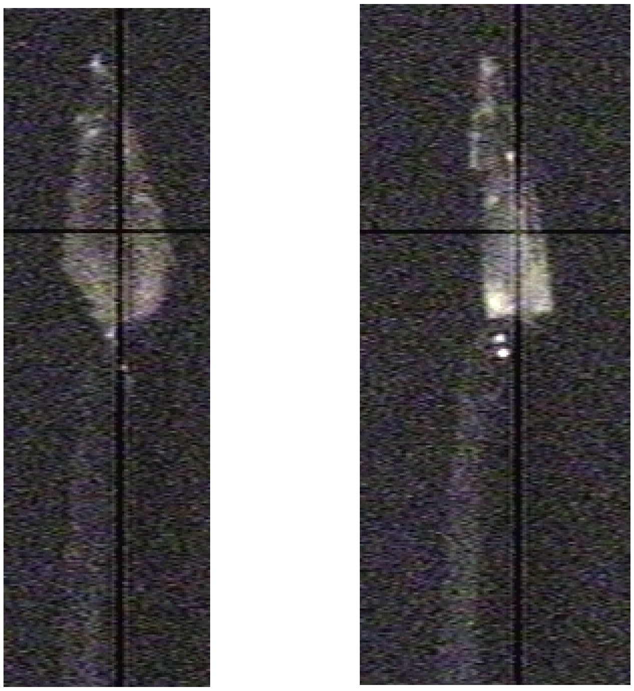

Fig. 1. The crystal

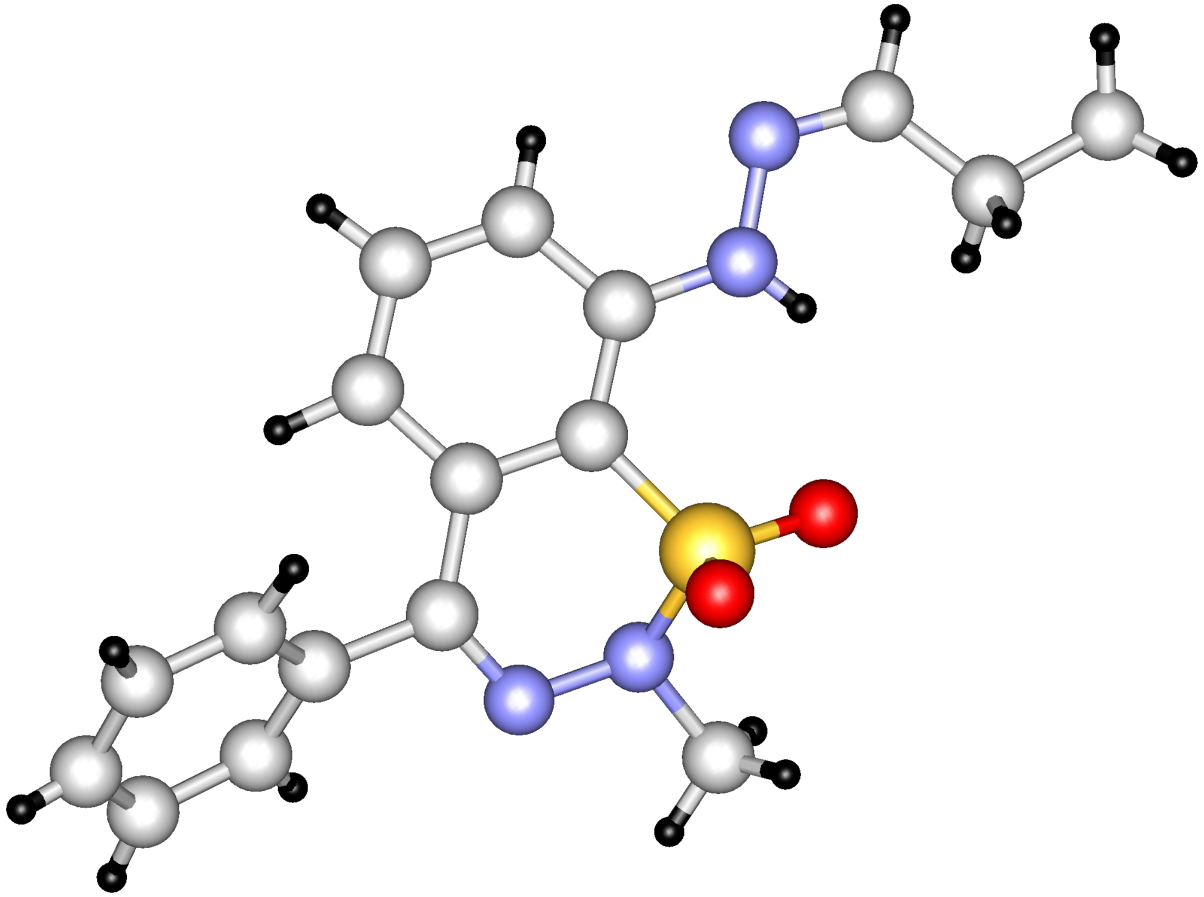

Fig. 2. the molecule (some hydrogens were generated by the software)

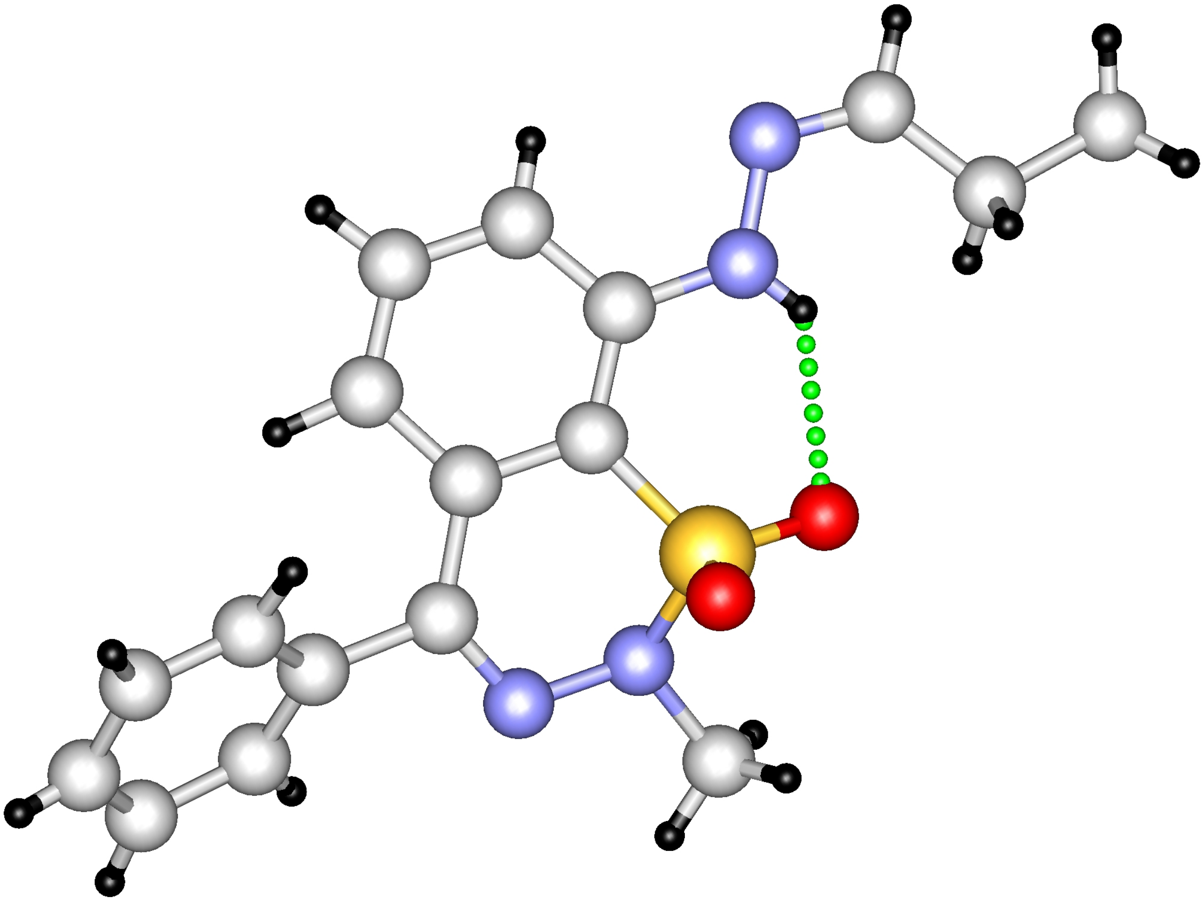

Fig. 3. Hydrogen bond

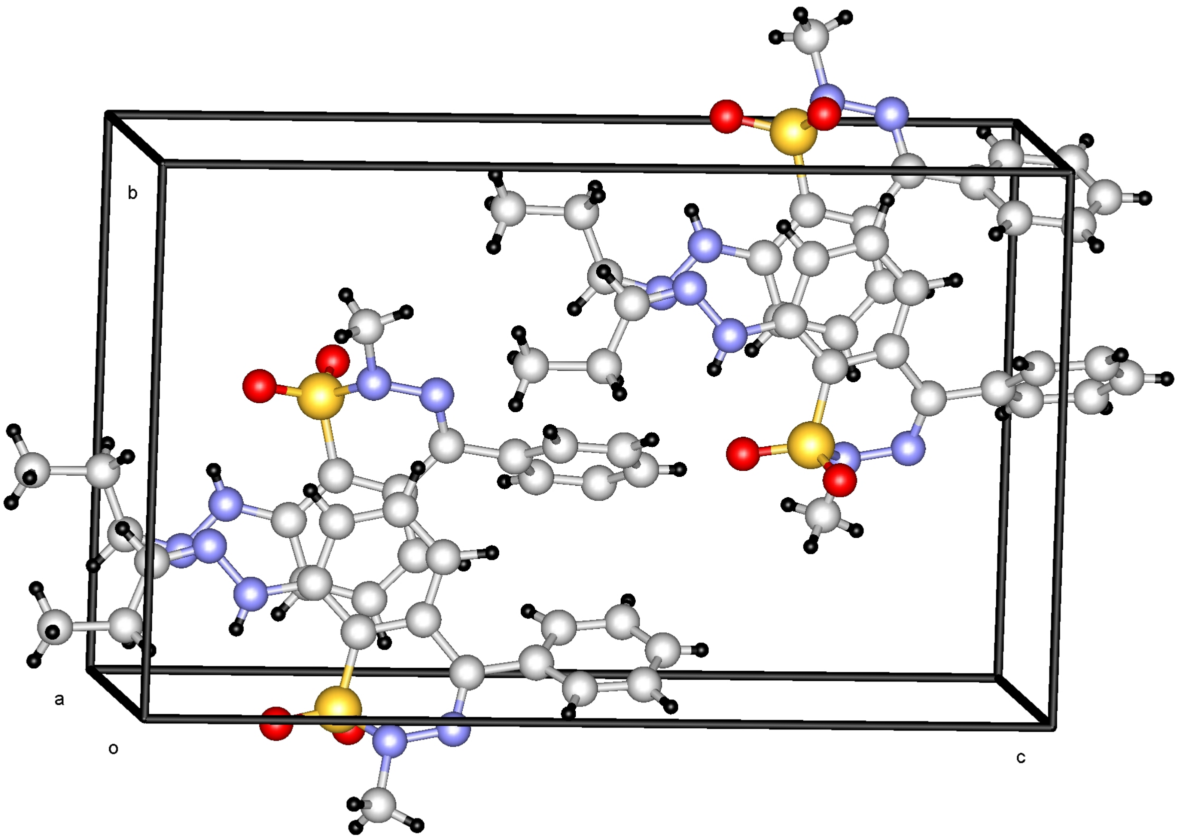


Fig. 4. Packing

*Experimental*

## Data Collection

A colorless prism crystal of $C_{17}H_{18}N_4O_2S$ having approximate dimensions of 0.39 x 0.14 x 0.09 mm was mounted on a cactus needle. All measurements were made on a Rigaku RAXIS RAPID imaging plate area detector with graphite monochromated Cu-K$\alpha$ radiation.

Indexing was performed from 4 oscillations that were exposed for 480 seconds. The crystal-to-detector distance was 127.40 mm.

Cell constants and an orientation matrix for data collection corresponded to a primitive orthorhombic cell with dimensions:

a = 7.1655(3) Å
b = 11.9214(5) Å
c = 19.5707(9) Å
V = 1671.78(13) $Å^3$

For Z = 4 and F.W. = 342.41, the calculated density is 1.360 $g/cm^3$. Based on the systematic absences of:

0kl: k+l ± 2n
h0l: h ± 2n

packing considerations, a statistical analysis of intensity distribution, and the successful solution and refinement of the structure, the space group was determined to be:

$Pna2_1$ (#33)

The data were collected at a temperature of 20 $\pm$ $1^oC$ to a maximum 2$\theta$ value of $143.2^o$. A total of 180 oscillation images were collected. A sweep of data was done using $\omega$ scans from 20.0 to $200.0^o$ in $5.0^o$ step, at $\chi$=$0.0^o$ and $\phi$ = $0.0^o$. The exposure rate was 96.0 [sec./$^o$]. A second sweep was performed using $\omega$ scans from 20.0 to $200.0^o$ in $5.0^o$ step, at $\chi$=$54.0^o$ and $\phi$ = $0.0^o$. The exposure rate was 96.0 [sec./$^o$]. Another sweep was performed using $\omega$ scans from 20.0 to $200.0^o$ in $5.0^o$ step, at $\chi$=$54.0^o$ and $\phi$ = $90.0^o$. The exposure rate was 96.0 [sec./$^o$]. Another sweep was performed using $\omega$ scans from 20.0 to $200.0^o$ in $5.0^o$ step, at $\chi$=$54.0^o$ and $\phi$ = $180.0^o$. The exposure rate was 96.0 [sec./$^o$]. Another sweep was performed using $\omega$ scans from 20.0 to $200.0^o$ in $5.0^o$ step, at $\chi$=$54.0^o$ and $\phi$ = $270.0^o$. The exposure rate was 96.0 [sec./$^o$]. The crystal-to-detector distance was 127.40 mm. Readout was performed in the 0.100 mm pixel mode.

## Data Reduction

Of the 18065 reflections that were collected, 2751 were unique ($R_{int}$ = 0.050).

The linear absorption coefficient, μ, for Cu-Kα radiation is 18.688 $cm^{-1}$. An empirical absorption correction was applied which resulted in transmission factors ranging from 0.604 to 0.853. The data were corrected for Lorentz and polarization effects.

## Structure Solution and Refinement

The structure was solved by direct methods[1] and expanded using Fourier techniques[2]. The non-hydrogen atoms were refined anisotropically. Some hydrogen atoms were refined isotropically and the rest were refined using the riding model.The final cycle of full-matrix least-squares refinement[3] on F was based on 13381 observed reflections ($I > 2.00\sigma(I)$) and 262 variable parameters and converged (largest parameter shift was 0.00 times its esd) with unweighted and weighted agreement factors of:

$$R = \Sigma \, ||Fo| - |Fc|| \, / \, \Sigma \, |Fo| = 0.0556$$

$$R_W = [\, \Sigma \, w \, (|Fo| - |Fc|)^2 \, / \, \Sigma \, w \, Fo^2]^{1/2} = 0.0550$$

The standard deviation of an observation of unit weight[4] was 2.93. Unit weights were used. Plots of $\Sigma \, w \, (|Fo| - |Fc|)^2$ versus |Fo|, reflection order in data collection, sin θ/λ and various classes of indices showed no unusual trends. The maximum and minimum peaks on the final difference Fourier map corresponded to 7.02 and -4.97 $e^-/Å^3$, respectively.

Neutral atom scattering factors were taken from Cromer and Waber[5]. Anomalous dispersion effects were included in Fcalc[6]; the values for Δf' and Δf" were those of Creagh and McAuley[7]. The values for the mass attenuation coefficients are those of Creagh and Hubbell[8]. All calculations were performed using the CrystalStructure[9,10] crystallographic software package.

## *References*

(1) SIR92: Altomare, A., Cascarano, G., Giacovazzo, C., Guagliardi, A., Burla, M., Polidori, G., and Camalli, M. (1994) J. Appl. Cryst., 27, 435.

(2) DIRDIF99: Beurskens, P.T., Admiraal, G., Beurskens, G., Bosman, W.P., de Gelder, R., Israel, R. and Smits, J.M.M.(1999). The DIRDIF-99 program system, Technical Report of the Crystallography Laboratory, University of Nijmegen, The Netherlands.

(3) Least Squares function minimized:

$$\Sigma w(|F_o|-|F_c|)^2 \quad \text{where w = Least Squares weights.}$$

(4) Standard deviation of an observation of unit weight:

$$[\Sigma w(|F_o|-|F_c|)^2/(N_o-N_v)]^{1/2}$$

where: $N_o$ = number of observations
$N_v$ = number of variables

(5) Cromer, D. T. & Waber, J. T.; "International Tables for X-ray Crystallography", Vol. IV, The Kynoch Press, Birmingham, England, Table 2.2 A (1974).

(6) Ibers, J. A. & Hamilton, W. C.; Acta Crystallogr., 17, 781 (1964).

(7) Creagh, D. C. & McAuley, W.J .; "International Tables for Crystallography", Vol C, (A.J.C. Wilson, ed.), Kluwer Academic Publishers, Boston, Table 4.2.6.8, pages 219-222 (1992).

(8) Creagh, D. C. & Hubbell, J.H..; "International Tables for Crystallography", Vol C, (A.J.C. Wilson, ed.), Kluwer Academic Publishers, Boston, Table 4.2.4.3, pages 200-206 (1992).

(9) CrystalStructure 3.7.0: Crystal Structure Analysis Package, Rigaku and Rigaku/MSC (2000-2005). 9009 New Trails Dr. The Woodlands TX 77381 USA.

(10) CRYSTALS Issue 10: Watkin, D.J., Prout, C.K. Carruthers, J.R. & Betteridge, P.W. Chemical Crystallography Laboratory, Oxford, UK. (1996)

# *EXPERIMENTAL DETAILS*

## A. Crystal Data

| | |
|---|---|
| Empirical Formula | $C_{17}H_{18}N_4O_2S$ |
| Formula Weight | 342.41 |
| Crystal Color, Habit | colorless, prism |
| Crystal Dimensions | 0.39 X 0.14 X 0.09 mm |
| Crystal System | orthorhombic |
| Lattice Type | Primitive |
| Indexing Images | 4 oscillations @ 480.0 seconds |
| Detector Position | 127.40 mm |
| Pixel Size | 0.100 mm |
| Lattice Parameters | a = 7.1655(3) Å<br>b = 11.9214(5) Å<br>c = 19.5707(9) Å<br>V = 1671.78(13) $Å^3$ |
| Space Group | $Pna2_1$ (#33) |
| Z value | 4 |
| $D_{calc}$ | 1.360 $g/cm^3$ |
| $F_{000}$ | 720.00 |
| $\mu(CuK\alpha)$ | 18.688 $cm^{-1}$ |

## B. Intensity Measurements

| | |
|---|---|
| Diffractometer | Rigaku RAXIS-RAPID |
| Radiation | CuK$\alpha$ ($\lambda$ = 1.54187 Å)<br>graphite monochromated |
| Detector Aperture | 280 mm x 256 mm |
| Data Images | 180 exposures |
| $\omega$ oscillation Range ($\chi$=0.0, $\phi$=0.0) | 20.0 - 200.0$^{o}$ |
| Exposure Rate | 96.0 sec./$^{o}$ |
| $\omega$ oscillation Range ($\chi$=54.0, $\phi$=0.0) | 20.0 - 200.0$^{o}$ |
| Exposure Rate | 96.0 sec./$^{o}$ |
| $\omega$ oscillation Range ($\chi$=54.0, $\phi$=90.0) | 20.0 - 200.0$^{o}$ |
| Exposure Rate | 96.0 sec./$^{o}$ |
| $\omega$ oscillation Range ($\chi$=54.0, $\phi$=180.0) | 20.0 - 200.0$^{o}$ |
| Exposure Rate | 96.0 sec./$^{o}$ |
| $\omega$ oscillation Range ($\chi$=54.0, $\phi$=270.0) | 20.0 - 200.0$^{o}$ |
| Exposure Rate | 96.0 sec./$^{o}$ |
| Detector Position | 127.40 mm |
| Pixel Size | 0.100 mm |
| $2\theta_{max}$ | 143.2$^{o}$ |
| No. of Reflections Measured | Total: 18065<br>Unique: 2751 ($R_{int}$ = 0.050) |
| Corrections | Lorentz-polarization<br>Absorption<br>(trans. factors: 0.604 - 0.853) |

## C. Structure Solution and Refinement

| | |
|---|---|
| Structure Solution | Direct Methods (SIR92) |
| Refinement | Full-matrix least-squares on F |
| Function Minimized | $\Sigma$ w (\|Fo\| - \|Fc\|)$^2$ |
| Least Squares Weights | 1 |
| $2\theta_{max}$ cutoff | 143.2$^o$ |
| Anomalous Dispersion | All non-hydrogen atoms |
| No. Observations (I>2.00$\sigma$(I)) | 13381 |
| No. Variables | 262 |
| Reflection/Parameter Ratio | 51.07 |
| Residuals: R (I>2.00$\sigma$(I)) | 0.0556 |
| Residuals: Rw (I>2.00$\sigma$(I)) | 0.0550 |
| Goodness of Fit Indicator | 2.934 |
| Max Shift/Error in Final Cycle | 0.000 |
| Maximum peak in Final Diff. Map | 7.02 $e^-/Å^3$ |
| Minimum peak in Final Diff. Map | -4.97 $e^-/Å^3$ |

Table 1. Atomic coordinates and $B_{iso}/B_{eq}$

| atom | x | y | z | $B_{eq}$ |
|---|---|---|---|---|
| S(1) | 0.33904(11) | -0.01007(6) | 0.23394(6) | 4.036(18) |
| O(2) | 0.2844(2) | -0.03193(14) | 0.16304(12) | 5.15(6) |
| O(3) | 0.5094(2) | -0.05803(16) | 0.25655(12) | 4.77(5) |
| N(1) | 0.4054(4) | 0.2765(2) | 0.09041(17) | 5.13(9) |
| N(4) | 0.1615(3) | -0.05043(19) | 0.28126(14) | 4.36(7) |
| N(5) | 0.1719(3) | -0.0290(2) | 0.35126(14) | 4.04(7) |
| N(6) | 0.3967(4) | 0.1911(2) | 0.13725(18) | 5.03(9) |
| C(1) | 0.3279(3) | 0.1311(2) | 0.25314(17) | 3.54(8) |
| C(8) | 0.3609(4) | 0.2155(2) | 0.20479(18) | 3.85(9) |
| C(9) | 0.2936(3) | 0.1572(2) | 0.32179(17) | 3.24(8) |
| C(10) | 0.2365(3) | 0.0674(2) | 0.36832(16) | 3.37(8) |
| C(12) | 0.2886(3) | 0.2705(2) | 0.3414(2) | 3.79(8) |
| C(13) | 0.2383(4) | 0.0865(2) | 0.44402(17) | 3.93(8) |
| C(14) | 0.3191(4) | 0.3529(2) | 0.2938(2) | 4.09(9) |
| C(15) | 0.0925(5) | 0.0531(2) | 0.4844(2) | 4.97(11) |
| C(16) | 0.4039(4) | 0.2480(3) | 0.0278(2) | 5.95(11) |
| C(17) | 0.0791(4) | -0.1589(2) | 0.26567(18) | 6.57(11) |
| C(18) | 0.1011(6) | 0.0612(3) | 0.5551(2) | 5.89(13) |
| C(19) | 0.3507(4) | 0.3270(2) | 0.2267(2) | 4.53(9) |
| C(20) | 0.4016(7) | 0.1470(3) | 0.5473(2) | 5.68(13) |
| C(21) | 0.3934(5) | 0.1333(3) | -0.00015(19) | 5.87(12) |
| C(22) | 0.3927(5) | 0.1366(3) | 0.4774(2) | 5.42(12) |
| C(23) | 0.2538(7) | 0.1095(3) | 0.5843(2) | 6.99(15) |
| C(24) | 0.3668(5) | 0.1308(3) | -0.0788(2) | 8.13(15) |
| H(1) | -0.014(4) | 0.032(2) | 0.5786(17) | 7.3(9) |
| H(2) | -0.023(4) | 0.018(2) | 0.4657(12) | 3.8(7) |
| H(3) | 0.269(2) | 0.1095(16) | 0.6274(11) | 0.7(5) |
| H(4) | 0.526(3) | 0.174(2) | 0.5607(15) | 4.5(8) |
| H(5) | 0.509(3) | 0.1555(18) | 0.4501(12) | 2.6(6) |
| H(6) | 0.255(2) | 0.2823(17) | 0.3923(12) | 2.2(5) |
| H(7) | 0.303(3) | 0.429(2) | 0.3111(13) | 4.3(7) |
| H(8) | 0.344(3) | 0.383(2) | 0.1957(14) | 4.7(8) |
| H(9) | 0.385(3) | 0.1293(18) | 0.1218(11) | 0.6(5) |
| H(10) | 0.4150 | 0.3057 | -0.0054 | 7.15 |
| H(12) | 0.5042 | 0.0937 | 0.0112 | 7.06 |
| H(13) | 0.2893 | 0.0975 | 0.0206 | 7.04 |
| H(14) | -0.0173 | -0.1492 | 0.2329 | 7.91 |
| H(15) | 0.0284 | -0.1906 | 0.3062 | 7.91 |
| H(16) | 0.1718 | -0.2077 | 0.2477 | 7.92 |
| H(17) | 0.2375 | 0.1332 | -0.0895 | 9.79 |
| H(18) | 0.4204 | 0.0648 | -0.0977 | 9.79 |
| H(19) | 0.4269 | 0.1949 | -0.0974 | 9.77 |

Table 1. Atomic coordinates and $B_{iso}/B_{eq}$ (continued)

$B_{eq} = 8/3\ \pi^2(U_{11}(aa^*)^2 + U_{22}(bb^*)^2 + U_{33}(cc^*)^2 + 2U_{12}(aa^*bb^*)\cos\gamma + 2U_{13}(aa^*cc^*)\cos\beta + 2U_{23}(bb^*cc^*)\cos\alpha)$

Table 2. Anisotropic displacement parameters

| atom | $U_{11}$ | $U_{22}$ | $U_{33}$ | $U_{12}$ | $U_{13}$ | $U_{23}$ |
|---|---|---|---|---|---|---|
| S(1) | 0.0553(4) | 0.0446(4) | 0.0534(5) | -0.0005(3) | 0.0031(5) | -0.0016(5) |
| O(2) | 0.0850(16) | 0.0590(14) | 0.0516(16) | -0.0106(11) | -0.0017(13) | -0.0055(12) |
| O(3) | 0.0606(13) | 0.0551(11) | 0.0657(18) | 0.0143(11) | -0.0060(12) | -0.0087(11) |
| N(1) | 0.082(2) | 0.0532(19) | 0.060(2) | -0.0065(15) | 0.0132(19) | 0.0098(18) |
| N(4) | 0.0689(18) | 0.0434(15) | 0.054(2) | -0.0033(13) | -0.0036(16) | -0.0071(13) |
| N(5) | 0.0568(16) | 0.0467(16) | 0.050(2) | -0.0071(13) | -0.0004(14) | 0.0011(13) |
| N(6) | 0.087(2) | 0.046(2) | 0.058(2) | -0.0105(17) | 0.0017(18) | 0.0020(19) |
| C(1) | 0.0377(16) | 0.0536(18) | 0.043(2) | 0.0028(14) | -0.0041(16) | 0.0021(15) |
| C(8) | 0.041(2) | 0.058(2) | 0.047(2) | 0.0028(15) | -0.0072(17) | 0.0024(17) |
| C(9) | 0.0298(16) | 0.0460(19) | 0.047(2) | -0.0047(12) | -0.0058(15) | -0.0012(17) |
| C(10) | 0.0335(16) | 0.054(2) | 0.040(2) | 0.0009(14) | 0.0035(15) | -0.0014(18) |
| C(12) | 0.0418(18) | 0.050(2) | 0.052(2) | -0.0019(14) | 0.0063(17) | -0.0057(19) |
| C(13) | 0.054(2) | 0.0395(18) | 0.056(2) | -0.0041(15) | 0.0008(19) | 0.0033(17) |
| C(14) | 0.0497(19) | 0.047(2) | 0.059(2) | 0.0020(16) | 0.0031(19) | -0.010(2) |
| C(15) | 0.067(2) | 0.055(2) | 0.066(3) | -0.0144(18) | 0.009(2) | 0.002(2) |
| C(16) | 0.089(2) | 0.081(2) | 0.057(3) | 0.002(2) | 0.016(2) | 0.029(2) |
| C(17) | 0.110(2) | 0.066(2) | 0.073(3) | -0.036(2) | 0.012(2) | -0.011(2) |
| C(18) | 0.086(3) | 0.067(2) | 0.071(4) | -0.005(2) | 0.025(2) | -0.002(2) |
| C(19) | 0.0500(18) | 0.0454(19) | 0.077(3) | -0.0020(15) | -0.004(2) | 0.026(2) |
| C(20) | 0.097(3) | 0.067(2) | 0.051(3) | -0.004(2) | -0.008(2) | -0.008(2) |
| C(21) | 0.100(3) | 0.075(3) | 0.049(3) | -0.002(2) | 0.008(2) | 0.009(2) |
| C(22) | 0.064(2) | 0.064(2) | 0.077(3) | -0.007(2) | 0.015(2) | 0.008(2) |
| C(23) | 0.148(5) | 0.074(2) | 0.043(3) | -0.001(3) | 0.006(3) | 0.001(2) |
| C(24) | 0.117(4) | 0.127(3) | 0.065(3) | 0.001(2) | 0.006(2) | -0.007(3) |

The general temperature factor expression: $\exp(-2\pi^2(a^{*2}U_{11}h^2 + b^{*2}U_{22}k^2 + c^{*2}U_{33}l^2 + 2a^*b^*U_{12}hk + 2a^*c^*U_{13}hl + 2b^*c^*U_{23}kl))$

Table 3. Bond lengths (Å)

| atom | atom | distance | atom | atom | distance |
|---|---|---|---|---|---|
| S(1) | O(2) | 1.465(2) | S(1) | O(3) | 1.419(2) |
| S(1) | N(4) | 1.646(2) | S(1) | C(1) | 1.727(2) |
| N(1) | N(6) | 1.371(4) | N(1) | C(16) | 1.271(5) |
| N(4) | N(5) | 1.396(4) | N(4) | C(17) | 1.454(3) |
| N(5) | C(10) | 1.284(3) | N(6) | C(8) | 1.377(4) |
| N(6) | H(9) | 0.80(2) | C(1) | C(8) | 1.401(4) |
| C(1) | C(9) | 1.401(4) | C(8) | C(19) | 1.399(4) |
| C(9) | C(10) | 1.464(4) | C(9) | C(12) | 1.404(4) |
| C(10) | C(13) | 1.499(4) | C(12) | C(14) | 1.371(5) |
| C(12) | H(6) | 1.04(2) | C(13) | C(15) | 1.369(5) |
| C(13) | C(22) | 1.417(5) | C(14) | C(19) | 1.367(6) |
| C(14) | H(7) | 0.98(2) | C(15) | C(18) | 1.389(6) |
| C(15) | H(2) | 1.00(2) | C(16) | C(21) | 1.475(5) |
| C(16) | H(10) | 0.950 | C(17) | H(14) | 0.950 |
| C(17) | H(15) | 0.950 | C(17) | H(16) | 0.950 |
| C(18) | C(23) | 1.363(6) | C(18) | H(1) | 1.01(3) |
| C(19) | H(8) | 0.91(2) | C(20) | C(22) | 1.374(6) |
| C(20) | C(23) | 1.359(7) | C(20) | H(4) | 0.99(2) |
| C(21) | C(24) | 1.551(5) | C(21) | H(12) | 0.950 |
| C(21) | H(13) | 0.950 | C(22) | H(5) | 1.02(2) |
| C(23) | H(3) | 0.85(2) | C(24) | H(17) | 0.950 |
| C(24) | H(18) | 0.950 | C(24) | H(19) | 0.950 |

Table 4. Bond angles ($^{o}$)

| atom | atom | atom | angle | atom | atom | atom | angle |
|---|---|---|---|---|---|---|---|
| O(2) | S(1) | O(3) | 116.98(13) | O(2) | S(1) | N(4) | 105.92(13) |
| O(2) | S(1) | C(1) | 111.54(14) | O(3) | S(1) | N(4) | 111.84(14) |
| O(3) | S(1) | C(1) | 111.40(13) | N(4) | S(1) | C(1) | 97.28(13) |
| N(6) | N(1) | C(16) | 116.5(3) | S(1) | N(4) | N(5) | 117.23(19) |
| S(1) | N(4) | C(17) | 117.1(2) | N(5) | N(4) | C(17) | 113.0(2) |
| N(4) | N(5) | C(10) | 116.0(2) | N(1) | N(6) | C(8) | 119.6(2) |
| N(1) | N(6) | H(9) | 115.7(17) | C(8) | N(6) | H(9) | 122.5(17) |
| S(1) | C(1) | C(8) | 123.0(2) | S(1) | C(1) | C(9) | 115.7(2) |
| C(8) | C(1) | C(9) | 121.2(2) | N(6) | C(8) | C(1) | 121.9(2) |
| N(6) | C(8) | C(19) | 120.3(3) | C(1) | C(8) | C(19) | 117.8(3) |
| C(1) | C(9) | C(10) | 118.9(2) | C(1) | C(9) | C(12) | 118.7(2) |
| C(10) | C(9) | C(12) | 121.8(2) | N(5) | C(10) | C(9) | 126.4(2) |
| N(5) | C(10) | C(13) | 113.3(2) | C(9) | C(10) | C(13) | 120.1(2) |
| C(9) | C(12) | C(14) | 120.0(3) | C(9) | C(12) | H(6) | 113.5(11) |
| C(14) | C(12) | H(6) | 126.5(11) | C(10) | C(13) | C(15) | 121.3(3) |
| C(10) | C(13) | C(22) | 121.8(3) | C(15) | C(13) | C(22) | 116.9(3) |
| C(12) | C(14) | C(19) | 121.1(3) | C(12) | C(14) | H(7) | 114.5(15) |
| C(19) | C(14) | H(7) | 124.1(15) | C(13) | C(15) | C(18) | 121.4(3) |
| C(13) | C(15) | H(2) | 122.9(15) | C(18) | C(15) | H(2) | 115.6(15) |
| N(1) | C(16) | C(21) | 127.3(3) | N(1) | C(16) | H(10) | 117.7 |
| C(21) | C(16) | H(10) | 115.0 | N(4) | C(17) | H(14) | 109.2 |
| N(4) | C(17) | H(15) | 109.5 | N(4) | C(17) | H(16) | 109.8 |
| H(14) | C(17) | H(15) | 109.5 | H(14) | C(17) | H(16) | 109.5 |
| H(15) | C(17) | H(16) | 109.5 | C(15) | C(18) | C(23) | 118.9(4) |
| C(15) | C(18) | H(1) | 113.2(19) | C(23) | C(18) | H(1) | 128(2) |
| C(8) | C(19) | C(14) | 121.2(3) | C(8) | C(19) | H(8) | 120.1(18) |
| C(14) | C(19) | H(8) | 117.8(17) | C(22) | C(20) | C(23) | 117.7(4) |
| C(22) | C(20) | H(4) | 109.8(17) | C(23) | C(20) | H(4) | 132.1(17) |
| C(16) | C(21) | C(24) | 113.1(3) | C(16) | C(21) | H(12) | 109.3 |
| C(16) | C(21) | H(13) | 107.3 | C(24) | C(21) | H(12) | 109.0 |
| C(24) | C(21) | H(13) | 108.6 | H(12) | C(21) | H(13) | 109.5 |
| C(13) | C(22) | C(20) | 122.2(3) | C(13) | C(22) | H(5) | 119.5(14) |
| C(20) | C(22) | H(5) | 117.7(14) | C(18) | C(23) | C(20) | 122.7(4) |
| C(18) | C(23) | H(3) | 121.1(14) | C(20) | C(23) | H(3) | 115.6(14) |
| C(21) | C(24) | H(17) | 109.7 | C(21) | C(24) | H(18) | 110.7 |
| C(21) | C(24) | H(19) | 108.0 | H(17) | C(24) | H(18) | 109.5 |
| H(17) | C(24) | H(19) | 109.5 | H(18) | C(24) | H(19) | 109.5 |

Table 4. Bond angles ($^{o}$) (continued)

| atom | atom | atom | angle | atom | atom | atom | angle |
|---|---|---|---|---|---|---|---|

Table 5. Torsion Angles(°)

| atom1 | atom2 | atom3 | atom4 | angle | atom1 | atom2 | atom3 | atom4 | angle |
|---|---|---|---|---|---|---|---|---|---|
| O(2) | S(1) | N(4) | N(5) | -174.43(18) | O(2) | S(1) | N(4) | C(17) | 46.4(2) |
| O(2) | S(1) | C(1) | C(8) | -31.2(2) | O(2) | S(1) | C(1) | C(9) | 152.26(19) |
| O(3) | S(1) | N(4) | N(5) | 57.1(2) | O(3) | S(1) | N(4) | C(17) | -82.1(2) |
| O(3) | S(1) | C(1) | C(8) | 101.5(2) | O(3) | S(1) | C(1) | C(9) | -75.0(2) |
| N(4) | S(1) | C(1) | C(8) | -141.6(2) | N(4) | S(1) | C(1) | C(9) | 41.9(2) |
| C(1) | S(1) | N(4) | N(5) | -59.5(2) | C(1) | S(1) | N(4) | C(17) | 161.3(2) |
| N(6) | N(1) | C(16) | C(21) | 0.2(4) | C(16) | N(1) | N(6) | C(8) | -167.3(3) |
| S(1) | N(4) | N(5) | C(10) | 41.9(3) | C(17) | N(4) | N(5) | C(10) | -177.3(2) |
| N(4) | N(5) | C(10) | C(9) | 2.2(4) | N(4) | N(5) | C(10) | C(13) | 178.2(2) |
| N(1) | N(6) | C(8) | C(1) | 172.0(2) | N(1) | N(6) | C(8) | C(19) | -6.0(4) |
| S(1) | C(1) | C(8) | N(6) | 3.0(4) | S(1) | C(1) | C(8) | C(19) | -179.0(2) |
| S(1) | C(1) | C(9) | C(10) | -11.2(3) | S(1) | C(1) | C(9) | C(12) | 178.02(19) |
| C(8) | C(1) | C(9) | C(10) | 172.2(2) | C(8) | C(1) | C(9) | C(12) | 1.4(3) |
| C(9) | C(1) | C(8) | N(6) | 179.3(2) | C(9) | C(1) | C(8) | C(19) | -2.7(4) |
| N(6) | C(8) | C(19) | C(14) | -178.7(3) | C(1) | C(8) | C(19) | C(14) | 3.3(4) |
| C(1) | C(9) | C(10) | N(5) | -17.3(4) | C(1) | C(9) | C(10) | C(13) | 166.9(2) |
| C(1) | C(9) | C(12) | C(14) | -0.7(3) | C(10) | C(9) | C(12) | C(14) | -171.3(2) |
| C(12) | C(9) | C(10) | N(5) | 153.2(2) | C(12) | C(9) | C(10) | C(13) | -22.5(3) |
| N(5) | C(10) | C(13) | C(15) | -41.1(4) | N(5) | C(10) | C(13) | C(22) | 137.0(3) |
| C(9) | C(10) | C(13) | C(15) | 135.2(3) | C(9) | C(10) | C(13) | C(22) | -46.8(4) |
| C(9) | C(12) | C(14) | C(19) | 1.4(4) | C(10) | C(13) | C(15) | C(18) | 173.9(3) |
| C(10) | C(13) | C(22) | C(20) | -175.2(3) | C(15) | C(13) | C(22) | C(20) | 3.0(5) |
| C(22) | C(13) | C(15) | C(18) | -4.2(5) | C(12) | C(14) | C(19) | C(8) | -2.7(4) |
| C(13) | C(15) | C(18) | C(23) | 3.9(5) | N(1) | C(16) | C(21) | C(24) | 171.9(3) |
| C(15) | C(18) | C(23) | C(20) | -2.3(6) | C(22) | C(20) | C(23) | C(18) | 1.1(6) |
| C(23) | C(20) | C(22) | C(13) | -1.4(6) | | | | | |

The sign is positive if when looking from atom 2 to atom 3 a clock-wise motion of atom 1 would superimpose it on atom 4.

Table 6. Distances beyond the asymmetric unit out to 3.60 Å

| atom | atom | distance | atom | atom | distance |
|---|---|---|---|---|---|
| O(2) | C(18)[1] | 3.495(4) | O(2) | C(20)[2] | 3.476(5) |
| O(2) | H(1)[1] | 2.55(3) | O(2) | H(3)[2] | 3.40(2) |
| O(2) | H(4)[2] | 2.95(2) | O(3) | C(14)[3] | 3.382(3) |
| O(3) | C(17)[4] | 3.416(3) | O(3) | C(24)[5] | 3.453(4) |
| O(3) | H(3)[2] | 3.05(2) | O(3) | H(7)[3] | 2.81(2) |
| O(3) | H(8)[3] | 3.39(2) | O(3) | H(14)[6] | 3.591 |
| O(3) | H(14)[4] | 3.526 | O(3) | H(15)[4] | 3.153 |
| O(3) | H(16)[4] | 3.030 | O(3) | H(18)[5] | 2.898 |
| O(3) | H(19)[5] | 3.323 | N(1) | C(18)[7] | 3.464(5) |
| N(1) | H(1)[7] | 3.15(3) | N(1) | H(13)[3] | 3.419 |
| N(4) | C(14)[8] | 3.410(4) | N(4) | H(7)[8] | 3.00(2) |
| N(4) | H(8)[8] | 3.46(2) | N(5) | C(14)[8] | 3.474(4) |
| N(5) | H(7)[8] | 3.00(2) | N(5) | H(10)[9] | 3.484 |
| N(5) | H(17)[10] | 3.390 | N(5) | H(18)[5] | 3.117 |
| N(5) | H(19)[9] | 3.514 | N(6) | H(8)[3] | 3.52(2) |
| C(1) | C(19)[8] | 3.494(4) | C(8) | C(19)[3] | 3.572(4) |
| C(9) | C(14)[8] | 3.446(4) | C(10) | C(14)[8] | 3.461(4) |
| C(10) | H(7)[8] | 3.30(2) | C(10) | H(18)[5] | 2.996 |
| C(12) | H(5)[8] | 3.05(2) | C(12) | H(6)[3] | 3.54(2) |
| C(13) | H(5)[8] | 3.49(2) | C(13) | H(12)[5] | 3.122 |
| C(13) | H(18)[5] | 3.147 | C(14) | O(3)[8] | 3.382(3) |
| C(14) | N(4)[3] | 3.410(4) | C(14) | N(5)[3] | 3.474(4) |
| C(14) | C(9)[3] | 3.446(4) | C(14) | C(10)[3] | 3.461(4) |
| C(15) | H(5)[8] | 3.59(2) | C(15) | H(6)[8] | 3.60(2) |
| C(15) | H(10)[9] | 2.956 | C(15) | H(12)[5] | 3.419 |
| C(15) | H(13)[10] | 3.349 | C(15) | H(17)[10] | 3.552 |
| C(16) | C(16)[8] | 3.583(4) | C(16) | C(16)[3] | 3.583(4) |
| C(16) | H(2)[7] | 3.55(2) | C(16) | H(12)[8] | 3.446 |
| C(16) | H(13)[3] | 3.323 | C(17) | O(3)[11] | 3.416(3) |
| C(17) | H(7)[8] | 3.49(2) | C(17) | H(16)[11] | 3.342 |
| C(17) | H(19)[9] | 3.197 | C(18) | O(2)[10] | 3.495(4) |
| C(18) | N(1)[9] | 3.464(5) | C(18) | H(4)[8] | 3.20(2) |
| C(18) | H(8)[9] | 3.50(2) | C(18) | H(10)[9] | 3.269 |
| C(18) | H(12)[5] | 3.485 | C(18) | H(13)[10] | 3.444 |
| C(19) | C(1)[3] | 3.494(4) | C(19) | C(8)[8] | 3.572(4) |
| C(20) | O(2)[5] | 3.476(5) | C(20) | H(4)[8] | 3.44(2) |
| C(20) | H(12)[5] | 3.030 | C(21) | C(22)[2] | 3.590(5) |

Table 6. Distances beyond the asymmetric unit out to 3.60 Å (continued)

| atom | atom | distance | atom | atom | distance |
|---|---|---|---|---|---|
| C(21) | H(2)$^{1)}$ | 3.28(2) | C(21) | H(10)$^{8)}$ | 3.506 |
| C(22) | C(21)$^{5)}$ | 3.590(5) | C(22) | H(6)$^{3)}$ | 3.23(2) |
| C(22) | H(12)$^{5)}$ | 2.918 | C(22) | H(18)$^{5)}$ | 3.117 |
| C(23) | H(4)$^{8)}$ | 3.09(2) | C(23) | H(8)$^{9)}$ | 3.54(2) |
| C(23) | H(12)$^{5)}$ | 3.305 | C(23) | H(14)$^{10)}$ | 3.399 |
| C(24) | O(3)$^{2)}$ | 3.453(4) | C(24) | H(2)$^{1)}$ | 3.16(2) |
| C(24) | H(5)$^{2)}$ | 3.57(2) | C(24) | H(7)$^{12)}$ | 3.45(2) |
| C(24) | H(15)$^{7)}$ | 3.189 | H(1) | O(2)$^{10)}$ | 2.55(3) |
| H(1) | N(1)$^{9)}$ | 3.15(3) | H(1) | H(4)$^{8)}$ | 3.54(4) |
| H(1) | H(8)$^{9)}$ | 3.14(4) | H(1) | H(9)$^{10)}$ | 3.38(3) |
| H(1) | H(10)$^{9)}$ | 3.232 | H(1) | H(13)$^{10)}$ | 2.748 |
| H(1) | H(14)$^{10)}$ | 3.338 | H(2) | C(16)$^{9)}$ | 3.55(2) |
| H(2) | C(21)$^{10)}$ | 3.28(2) | H(2) | C(24)$^{10)}$ | 3.16(2) |
| H(2) | H(6)$^{8)}$ | 3.20(3) | H(2) | H(7)$^{8)}$ | 3.33(3) |
| H(2) | H(10)$^{9)}$ | 2.706 | H(2) | H(13)$^{10)}$ | 2.586 |
| H(2) | H(17)$^{10)}$ | 2.602 | H(2) | H(18)$^{10)}$ | 3.258 |
| H(3) | O(2)$^{5)}$ | 3.40(2) | H(3) | O(3)$^{5)}$ | 3.05(2) |
| H(3) | H(4)$^{8)}$ | 3.37(3) | H(3) | H(8)$^{9)}$ | 3.12(3) |
| H(3) | H(14)$^{10)}$ | 2.780 | H(3) | H(16)$^{13)}$ | 3.236 |
| H(4) | O(2)$^{5)}$ | 2.95(2) | H(4) | C(18)$^{3)}$ | 3.20(2) |
| H(4) | C(20)$^{3)}$ | 3.44(2) | H(4) | C(23)$^{3)}$ | 3.09(2) |
| H(4) | H(1)$^{3)}$ | 3.54(4) | H(4) | H(3)$^{3)}$ | 3.37(3) |
| H(4) | H(12)$^{5)}$ | 3.343 | H(4) | H(13)$^{5)}$ | 3.585 |
| H(5) | C(12)$^{3)}$ | 3.05(2) | H(5) | C(13)$^{3)}$ | 3.49(2) |
| H(5) | C(15)$^{3)}$ | 3.59(2) | H(5) | C(24)$^{5)}$ | 3.57(2) |
| H(5) | H(6)$^{3)}$ | 2.22(3) | H(5) | H(7)$^{3)}$ | 3.59(3) |
| H(5) | H(12)$^{5)}$ | 3.204 | H(5) | H(18)$^{5)}$ | 2.833 |
| H(6) | C(12)$^{8)}$ | 3.54(2) | H(6) | C(15)$^{3)}$ | 3.60(2) |
| H(6) | C(22)$^{8)}$ | 3.23(2) | H(6) | H(2)$^{3)}$ | 3.20(3) |
| H(6) | H(5)$^{8)}$ | 2.22(3) | H(7) | O(3)$^{8)}$ | 2.81(2) |
| H(7) | N(4)$^{3)}$ | 3.00(2) | H(7) | N(5)$^{3)}$ | 3.00(2) |
| H(7) | C(10)$^{3)}$ | 3.30(2) | H(7) | C(17)$^{3)}$ | 3.49(2) |
| H(7) | C(24)$^{13)}$ | 3.45(2) | H(7) | H(2)$^{3)}$ | 3.33(3) |
| H(7) | H(5)$^{8)}$ | 3.59(3) | H(7) | H(14)$^{3)}$ | 3.296 |
| H(7) | H(15)$^{3)}$ | 3.509 | H(7) | H(17)$^{13)}$ | 3.127 |
| H(7) | H(18)$^{13)}$ | 2.890 | H(8) | O(3)$^{8)}$ | 3.39(2) |
| H(8) | N(4)$^{3)}$ | 3.46(2) | H(8) | N(6)$^{8)}$ | 3.52(2) |

Table 6. Distances beyond the asymmetric unit out to 3.60 Å (continued)

| atom | atom | distance | atom | atom | distance |
|---|---|---|---|---|---|
| H(8) | C(18)$^{7)}$ | 3.50(2) | H(8) | C(23)$^{7)}$ | 3.54(2) |
| H(8) | H(1)$^{7)}$ | 3.14(4) | H(8) | H(3)$^{7)}$ | 3.12(3) |
| H(8) | H(9)$^{8)}$ | 3.60(3) | H(8) | H(14)$^{3)}$ | 3.400 |
| H(9) | H(1)$^{1)}$ | 3.38(3) | H(9) | H(8)$^{3)}$ | 3.60(3) |
| H(10) | N(5)$^{7)}$ | 3.484 | H(10) | C(15)$^{7)}$ | 2.956 |
| H(10) | C(18)$^{7)}$ | 3.269 | H(10) | C(21)$^{3)}$ | 3.506 |
| H(10) | H(1)$^{7)}$ | 3.232 | H(10) | H(2)$^{7)}$ | 2.706 |
| H(10) | H(12)$^{8)}$ | 3.195 | H(10) | H(13)$^{3)}$ | 2.963 |
| H(10) | H(17)$^{3)}$ | 2.929 | H(12) | C(13)$^{2)}$ | 3.122 |
| H(12) | C(15)$^{2)}$ | 3.419 | H(12) | C(16)$^{3)}$ | 3.446 |
| H(12) | C(18)$^{2)}$ | 3.485 | H(12) | C(20)$^{2)}$ | 3.030 |
| H(12) | C(22)$^{2)}$ | 2.918 | H(12) | C(23)$^{2)}$ | 3.305 |
| H(12) | H(4)$^{2)}$ | 3.343 | H(12) | H(5)$^{2)}$ | 3.204 |
| H(12) | H(10)$^{3)}$ | 3.195 | H(13) | N(1)$^{8)}$ | 3.419 |
| H(13) | C(15)$^{1)}$ | 3.349 | H(13) | C(16)$^{8)}$ | 3.323 |
| H(13) | C(18)$^{1)}$ | 3.444 | H(13) | H(1)$^{1)}$ | 2.748 |
| H(13) | H(2)$^{1)}$ | 2.586 | H(13) | H(4)$^{2)}$ | 3.585 |
| H(13) | H(10)$^{8)}$ | 2.963 | H(14) | O(3)$^{14)}$ | 3.591 |
| H(14) | O(3)$^{11)}$ | 3.526 | H(14) | C(23)$^{1)}$ | 3.399 |
| H(14) | H(1)$^{1)}$ | 3.338 | H(14) | H(3)$^{1)}$ | 2.780 |
| H(14) | H(7)$^{8)}$ | 3.296 | H(14) | H(8)$^{8)}$ | 3.400 |
| H(14) | H(16)$^{11)}$ | 2.820 | H(15) | O(3)$^{11)}$ | 3.153 |
| H(15) | C(24)$^{9)}$ | 3.189 | H(15) | H(7)$^{8)}$ | 3.509 |
| H(15) | H(16)$^{11)}$ | 3.051 | H(15) | H(17)$^{9)}$ | 3.376 |
| H(15) | H(17)$^{10)}$ | 2.876 | H(15) | H(18)$^{9)}$ | 3.490 |
| H(15) | H(19)$^{9)}$ | 2.352 | H(16) | O(3)$^{11)}$ | 3.030 |
| H(16) | C(17)$^{4)}$ | 3.342 | H(16) | H(3)$^{12)}$ | 3.236 |
| H(16) | H(14)$^{4)}$ | 2.820 | H(16) | H(15)$^{4)}$ | 3.051 |
| H(16) | H(19)$^{9)}$ | 3.323 | H(17) | N(5)$^{1)}$ | 3.390 |
| H(17) | C(15)$^{1)}$ | 3.552 | H(17) | H(2)$^{1)}$ | 2.602 |
| H(17) | H(7)$^{12)}$ | 3.127 | H(17) | H(10)$^{8)}$ | 2.929 |
| H(17) | H(15)$^{7)}$ | 3.376 | H(17) | H(15)$^{1)}$ | 2.876 |
| H(17) | H(19)$^{8)}$ | 3.029 | H(18) | O(3)$^{2)}$ | 2.898 |
| H(18) | N(5)$^{2)}$ | 3.117 | H(18) | C(10)$^{2)}$ | 2.996 |
| H(18) | C(13)$^{2)}$ | 3.147 | H(18) | C(22)$^{2)}$ | 3.117 |
| H(18) | H(2)$^{1)}$ | 3.258 | H(18) | H(5)$^{2)}$ | 2.833 |
| H(18) | H(7)$^{12)}$ | 2.890 | H(18) | H(15)$^{7)}$ | 3.490 |

Table 6. Distances beyond the asymmetric unit out to 3.60 Å (continued)

| atom | atom | distance | atom | atom | distance |
|---|---|---|---|---|---|
| H(19) | O(3)[2)] | 3.323 | H(19) | N(5)[7)] | 3.514 |
| H(19) | C(17)[7)] | 3.197 | H(19) | H(15)[7)] | 2.352 |
| H(19) | H(16)[7)] | 3.323 | H(19) | H(17)[3)] | 3.029 |

Symmetry Operators:

(1) -X,-Y,Z+1/2-1
(2) -X+1,-Y,Z+1/2-1
(3) X+1/2,-Y+1/2,Z
(4) X+1/2,-Y+1/2-1,Z
(5) -X+1,-Y,Z+1/2
(6) X+1,Y,Z
(7) -X+1/2,Y+1/2,Z+1/2-1
(8) X+1/2-1,-Y+1/2,Z
(9) -X+1/2,Y+1/2-1,Z+1/2
(10) -X,-Y,Z+1/2
(11) X+1/2-1,-Y+1/2-1,Z
(12) -X+1/2,Y+1/2-1,Z+1/2-1
(13) -X+1/2,Y+1/2,Z+1/2
(14) X-1,Y,Z

Table 7. Intramolecular and Intermolecular Hydrogen bonds

| D | H | A | D...A | D-H | H...A | D-H...A |
|---|---|---|---|---|---|---|
| N(6) | H(9) | O(2) | 2.824(3) | 0.80(2) | 2.21(2) | 134(2) |

Note) 1. The symmetry operations are applied to the acceptors.
2. Estimated standard deviations (esd's) are shown in the parentheses.
They are not calculated when all atoms have an esd=0.0.
