# Supplementary material for: Thiadiazino-indole, thiadiazino-carbazole and benzothiadiazino-carbazole dioxides: synthesis, physicochemical and early ADME characterization of representatives of new tri-, tetra- and pentacyclic ring systems and their intermediates
Source: Beilstein J Org Chem. 2025 Oct 21;21:2220–33. doi: 10.3762/bjoc.21.169 (PMC12557438; doi:10.3762/bjoc.21.169)

checkCIF/PLATON report

You have not supplied any structure factors. As a result the full set of tests cannot be run.

THIS REPORT IS FOR GUIDANCE ONLY. IF USED AS PART OF A REVIEW PROCEDURE FOR PUBLICATION, IT SHOULD NOT REPLACE THE EXPERTISE OF AN EXPERIENCED CRYSTALLOGRAPHIC REFEREE.

No syntax errors found. CIF dictionary Interpreting this report

Datablock: _143519_2

Bond precision: C-C = 0.0088 A Wavelength=1.54187

Cell:	a=6.3364(8)	b=9.2356(13)	c=15.0269(18)
	alpha=94.676(9)	beta=95.441(7)	gamma=93.115(8)
Temperature:	293 K		

	Calculated	Reported
Volume	870.8(2)	870.80(19)
Space group	P 1	P 1
Hall group	P 1	P 1
Moiety formula	C19 H20 N4 O2 S	C19 H20 N4 O2 S
Sum formula	C19 H20 N4 O2 S	C19 H20 N4 O2 S
Mr	368.45	368.45
Dx,g cm-3	1.405	1.405
Z	2	2
Mu (mm-1)	1.835	1.836
F000	388.0	388.0
F000'	389.73	
h,k,lmax	7,11,18	7,11,18
Nref	6830[3415]	5016
Tmin,Tmax	0.573,0.879	0.653,0.879
Tmin'	0.520	

Correction method= # Reported T Limits: Tmin=0.653 Tmax=0.879
AbsCorr = MULTI-SCAN

Data completeness= 1.47/0.73 Theta(max)= 71.650

R(reflections)= 0.0406(7920) wR2(reflections)= 0.0647(7963)

S = 0.940 Npar= 509

The following ALERTS were generated. Each ALERT has the format
test-name_ALERT_alert-type_alert-level.
Click on the hyperlinks for more details of the test.

Alert level A

```
DIFMN02_ALERT_2_A  The minimum difference density is < -0.1*ZMAX*2.00
            _refine_diff_density_min given =    -13.300
            Test value =     -3.200
```

Author Response: Altough the residual density is out of border but there is no doubt about the chemical structure taking into account other evidences, e.g. NMR. The error maybe due to crystal imperfections.

```
SHFSU01_ALERT_2_A  The absolute value of parameter shift to su ratio > 0.20
            Absolute value of the parameter shift to su ratio given   3.363
            Additional refinement cycles may be required.
```

Author Response: There was some convergence problem at the refinement, however there is no doubt about the chemical structure taking into account other evidences, e.g. NMR. The error maybe due to crystal imperfections.

```
PLAT029_ALERT_3_A _diffrn_measured_fraction_theta_full value Low .      0.892 Why?
```

Author Response: Altough some large-theta reflexions are too weak but there is no doubt about the chemical structure taking into account other evidences, e.g. NMR. The error maybe due to crystal imperfections.

```
PLAT080_ALERT_2_A Maximum Shift/Error ...........................       3.36 Why ?
```

Author Response: There was some convergence problem at the refinement, however there is no doubt about the chemical structure taking into account other evidences, e.g. NMR. The error maybe due to crystal imperfections.

```
PLAT097_ALERT_2_A Large Reported Max.  (Positive) Residual Density       3.27 eA-3
```

Author Response: Altough the residual density is out of border but there is no doubt about the chemical structure taking into account other evidences, e.g. NMR. The error maybe due to crystal imperfections.

```
PLAT098_ALERT_2_A Large Reported Min.  (Negative) Residual Density     -13.30 eA-3
```

Author Response: Altough the residual density is out of border but there is no doubt about the chemical structure taking into account other evidences, e.g. NMR. The error maybe due to crystal imperfections.

```
PLAT703_ALERT_1_A Torsion Calc   -178.7(6), Rep   -176.8(9), Dev..       3.17 Sigma
            N(13-C(1)-C(25-C(42  1_555  1_555  1_555  1_555      #   44 Check
```

Author Response: The structure is affected by some strain. Crystal imperfections can not be excluded either.

```
PLAT703_ALERT_1_A Torsion Calc     -0.2(9), Rep     2.6(14), Dev..       3.11 Sigma
            C(37-C(1)-C(25-C(42  1_555  1_555  1_555  1_555      #   48 Check
```

Author Response: The structure is affected by some strain. Crystal imperfections can not be excluded either.

```
PLAT703_ALERT_1_A Torsion Calc   -176.9(6), Rep   -174.9(9), Dev..       3.33 Sigma
            C(41-C(3)-C(27-C(49  1_555  1_555  1_555  1_555      #   59 Check
```

Author Response: The structure is affected by some strain. Crystal imperfections can not be excluded either.

```
PLAT703_ALERT_1_A Torsion Calc    -6.4(10), Rep    -9.8(15), Dev..       3.40 Sigma
            C(34-C(24-C(31-C(27  1_555  1_555  1_555  1_555      #   83 Check
```

Author Response: The structure is affected by some strain. Crystal imperfections can not be excluded either.

```
PLAT703_ALERT_1_A Torsion Calc     -6.4(9), Rep    -9.9(14), Dev..       3.89 Sigma
            C(1)-C(25-C(42-C(46  1_555  1_555  1_555  1_555      #   87 Check
```

Author Response: The structure is affected by some strain. Crystal imperfections can not be excluded either.

```
PLAT703_ALERT_1_A Torsion Calc     1.1(10), Rep     5.8(15), Dev..       4.70 Sigma
            C(40-C(25-C(42-C(45  1_555  1_555  1_555  1_555      #   88 Check
```

Author Response: The structure is affected by some strain. Crystal imperfections can not be excluded either.

```
PLAT703_ALERT_1_A Torsion Calc     3.5(10), Rep     0.2(11), Dev..       3.30 Sigma
            C(42-C(25-C(40-C(30  1_555  1_555  1_555  1_555      #   90 Check
```

Author Response: The structure is affected by some strain. Crystal imperfections can not be excluded either.

```
PLAT703_ALERT_1_A Torsion Calc      4.5(9), Rep     8.0(14), Dev..       3.89 Sigma
            C(3)-C(27-C(31-C(24  1_555  1_555  1_555  1_555      #   91 Check
```

Author Response: The structure is affected by some strain. Crystal imperfections can not be excluded either.

```
PLAT703_ALERT_1_A Torsion Calc    148.7(6), Rep   145.3(10), Dev..       5.67 Sigma
              C(24-C(31-C(43-C(39  1_555  1_555  1_555  1_555      #  104 Check
```

Author Response: The structure is affected by some strain. Crystal imperfections can not be excluded either.

```
PLAT703_ALERT_1_A Torsion Calc     54.8(7), Rep    57.0(11), Dev..       3.14 Sigma
              C(35-C(39-C(43-C(31  1_555  1_555  1_555  1_555      #  113 Check
```

Author Response: The structure is affected by some strain. Crystal imperfections can not be excluded either.

```
PLAT703_ALERT_1_A Torsion Calc    -4.6(10), Rep    -7.8(14), Dev..       3.20 Sigma
              C(25-C(42-C(45-C(29  1_555  1_555  1_555  1_555      #  114 Check
```

Author Response: The structure is affected by some strain. Crystal imperfections can not be excluded either.

```
PLAT703_ALERT_1_A Torsion Calc   -150.1(6), Rep   -152.1(8), Dev..       3.33 Sigma
              C(45-C(42-C(46-C(48  1_555  1_555  1_555  1_555      #  116 Check
```

Author Response: The structure is affected by some strain. Crystal imperfections can not be excluded either.

Alert level B

```
REFLT02_ALERT_1_B  The number of reflections greater than the sigma threshold
            cannot exceed the number of symmetry-independent reflections
            Number of symmetry-independent reflections =   5016
            Number of reflections greater than sigma threshold =   7920
PLAT111_ALERT_2_B ADDSYM Detects New (Pseudo) Centre of Symmetry .        100 %Fit
PLAT113_ALERT_2_B ADDSYM Suggests Possible Pseudo/New  Space Group        P-1 Check
              Check Model Parameter Symmetry for Reflection Data Support
PLAT230_ALERT_2_B Hirshfeld Test Diff for    S1       --O3       .       10.0 s.u.
PLAT230_ALERT_2_B Hirshfeld Test Diff for    N4       --N8       .       11.5 s.u.
PLAT230_ALERT_2_B Hirshfeld Test Diff for    C28      --C50      .       10.4 s.u.
PLAT230_ALERT_2_B Hirshfeld Test Diff for    S2       --O5       .       11.0 s.u.
PLAT230_ALERT_2_B Hirshfeld Test Diff for    N1       --N2       .       13.5 s.u.
PLAT230_ALERT_2_B Hirshfeld Test Diff for    C16      --C20      .       11.5 s.u.
PLAT230_ALERT_2_B Hirshfeld Test Diff for    C29      --C45      .        7.7 s.u.
PLAT703_ALERT_1_B Torsion Calc   -175.0(5), Rep   -176.1(7), Dev..       2.20 Sigma
              C(19-N(4)-N(8)-C(21  1_555  1_555  1_555  1_555      #   35 Check
```

Author Response: The structure is affected by some strain. Crystal imperfections can not be excluded either.

```
PLAT703_ALERT_1_B Torsion Calc    178.6(5), Rep    179.9(6), Dev..       2.60 Sigma
              N(5)-N(10-C(49-C(27  1_555  1_555  1_555  1_555      #   39 Check
```

Author Response: The structure is affected by some strain. Crystal imperfections can not be excluded either.

```
PLAT703_ALERT_1_B Torsion Calc   -178.6(5), Rep   -177.2(8), Dev..       2.80 Sigma
              N(3)-N(13-C(1)-C(25  1_555  1_555  1_555  1_555      #   41 Check
```

Author Response: The structure is affected by some strain. Crystal imperfections can not be excluded either.

```
PLAT703_ALERT_1_B Torsion Calc     -0.9(9), Rep    -3.5(14), Dev..       2.89 Sigma
              N(13-C(1)-C(25-C(40  1_555  1_555  1_555  1_555      #   43 Check
```

Author Response: The structure is affected by some strain. Crystal imperfections can not be excluded either.

```
PLAT703_ALERT_1_B Torsion Calc    177.6(6), Rep    175.8(9), Dev..       3.00 Sigma
              C(37-C(1)-C(25-C(40  1_555  1_555  1_555  1_555      #   47 Check
```

Author Response: The structure is affected by some strain. Crystal imperfections can not be excluded either.

```
PLAT703_ALERT_1_B Torsion Calc     -8.8(9), Rep   -10.9(14), Dev..       2.33 Sigma
              C(36-C(23-C(26-C(44  1_555  1_555  1_555  1_555      #   78 Check
```

Author Response: The structure is affected by some strain. Crystal imperfections can not be excluded either.

```
PLAT703_ALERT_1_B Torsion Calc   -174.3(6), Rep   -173.0(9), Dev..       2.17 Sigma
              C(1)-C(25-C(40-C(30  1_555  1_555  1_555  1_555      #   85 Check
```

Author Response: The structure is affected by some strain. Crystal imperfections can not be excluded either.

```
PLAT703_ALERT_1_B Torsion Calc      4.2(9), Rep     2.2(14), Dev..       2.22 Sigma
              C(3)-C(27-C(49-N(10  1_555  1_555  1_555  1_555      #   93 Check
```

Author Response: The structure is affected by some strain. Crystal imperfections can not be excluded either.

```
PLAT703_ALERT_1_B Torsion Calc      4.5(9), Rep     6.9(13), Dev..       2.67 Sigma
              C(31-C(27-C(49-C(35  1_555  1_555  1_555  1_555      #   96 Check
```

Author Response: The structure is affected by some strain. Crystal imperfections can not be excluded either.

```
PLAT703_ALERT_1_B Torsion Calc     -2.7(9), Rep    -5.3(14), Dev..       2.89 Sigma
              C(49-C(27-C(31-C(43  1_555  1_555  1_555  1_555      #   98 Check
```

Author Response: The structure is affected by some strain. Crystal imperfections can not be excluded either.

```
PLAT703_ALERT_1_B Torsion Calc    178.7(6), Rep    177.4(9), Dev..       2.17 Sigma
              N(5)-C(28-C(50-C(52  1_555  1_555  1_555  1_555      #   99 Check
```

Author Response: The structure is affected by some strain. Crystal imperfections can not be excluded either.

```
PLAT703_ALERT_1_B Torsion Calc     -4.4(9), Rep    -6.3(14), Dev..       2.11 Sigma
              C(2)-C(28-C(50-C(52  1_555  1_555  1_555  1_555      #  100 Check
```

Author Response: The structure is affected by some strain. Crystal imperfections can not be excluded either.

```
PLAT703_ALERT_1_B Torsion Calc   -159.5(6), Rep   -157.9(8), Dev..       2.67 Sigma
              C(39-C(35-C(49-N(10  1_555  1_555  1_555  1_555      #  107 Check
```

Author Response: The structure is affected by some strain. Crystal imperfections can not be excluded either.

```
PLAT703_ALERT_1_B Torsion Calc    -51.7(7), Rep   -53.7(10), Dev..       2.86 Sigma
              C(49-C(35-C(39-C(43  1_555  1_555  1_555  1_555      #  109 Check
```

Author Response: The structure is affected by some strain. Crystal imperfections can not be excluded either.

```
PLAT703_ALERT_1_B Torsion Calc    178.8(6), Rep   -179.8(7), Dev..       2.33 Sigma
              C(19-C(38-C(47-C(52  1_555  1_555  1_555  1_555      #  112 Check
```

Author Response: The structure is affected by some strain. Crystal imperfections can not be excluded either.

```
PLAT703_ALERT_1_B Torsion Calc     7.1(11), Rep    10.0(16), Dev..       2.64 Sigma
              C(28-C(50-C(52-C(47  1_555  1_555  1_555  1_555      #  120 Check
```

Author Response: The structure is affected by some strain. Crystal imperfections can not be excluded either.

Alert level C

```
DIFMN03_ALERT_1_C  The minimum difference density is < -0.1*ZMAX*0.75
            The relevant atom site should be identified.
DIFMX02_ALERT_1_C  The maximum difference density is > 0.1*ZMAX*0.75
            The relevant atom site should be identified.
PLAT034_ALERT_1_C No Flack Parameter Given. Z > Si, NonCentro ....     Please Do !
PLAT089_ALERT_3_C Poor Data / Parameter Ratio (Zmax < 18) ........       6.71 Note
PLAT213_ALERT_2_C Atom C21             has ADP max/min Ratio .....        3.1 prolat
PLAT230_ALERT_2_C Hirshfeld Test Diff for    N8       --C21      .        5.5 s.u.
PLAT230_ALERT_2_C Hirshfeld Test Diff for    C2       --C28      .        5.4 s.u.
PLAT230_ALERT_2_C Hirshfeld Test Diff for    C19      --C32      .        5.4 s.u.
PLAT230_ALERT_2_C Hirshfeld Test Diff for    C19      --C38      .        7.0 s.u.
PLAT230_ALERT_2_C Hirshfeld Test Diff for    C24      --C31      .        6.8 s.u.
PLAT230_ALERT_2_C Hirshfeld Test Diff for    C24      --C34      .        5.2 s.u.
PLAT230_ALERT_2_C Hirshfeld Test Diff for    C35      --C39      .        6.2 s.u.
PLAT230_ALERT_2_C Hirshfeld Test Diff for    C1       --C37      .        5.4 s.u.
PLAT230_ALERT_2_C Hirshfeld Test Diff for    C42      --C45      .        5.7 s.u.
PLAT241_ALERT_2_C High   'MainMol' Ueq as Compared to Neighbors of        C39 Check
PLAT241_ALERT_2_C High   'MainMol' Ueq as Compared to Neighbors of        C18 Check
PLAT340_ALERT_3_C Low Bond Precision on  C-C Bonds ..............    0.00876 Ang.
PLAT369_ALERT_2_C Long   C(sp2)-C(sp2) Bond  C1       - C25      .       1.54 Ang.
PLAT414_ALERT_2_C Short Intra D-H..H-X       H1       ..H18      .       1.95 Ang.
                                                      x,y,z  =      1_555 Check
PLAT703_ALERT_1_C Torsion Calc     39.6(6), Rep     40.3(9), Dev..       1.17 Sigma
              O(2)-S(1)-C(2)-C(28  1_555  1_555  1_555  1_555      #    3 Check
```

Author Response: The structure is affected by some strain. Crystal imperfections can not be excluded either.

```
PLAT703_ALERT_1_C Torsion Calc   -146.8(5), Rep   -146.1(7), Dev..       1.40 Sigma
              O(2)-S(1)-C(2)-C(38  1_555  1_555  1_555  1_555      #    4 Check
```

Author Response: The structure is affected by some strain. Crystal imperfections can not be excluded either.

```
PLAT703_ALERT_1_C Torsion Calc    152.8(5), Rep    153.4(8), Dev..       1.20 Sigma
              N(8)-S(1)-C(2)-C(28  1_555  1_555  1_555  1_555      #    9 Check
```

Author Response: The structure is affected by some strain. Crystal imperfections can not be excluded either.

```
PLAT703_ALERT_1_C Torsion Calc     49.9(5), Rep     50.5(8), Dev..       1.20 Sigma
              C(2)-S(1)-N(8)-N(4)  1_555  1_555  1_555  1_555      #   11 Check
```

Author Response: The structure is affected by some strain. Crystal imperfections can not be excluded either.

```
PLAT703_ALERT_1_C Torsion Calc   -176.4(4), Rep   -175.6(6), Dev..       2.00 Sigma
              C(2)-S(1)-N(8)-C(21  1_555  1_555  1_555  1_555      #   12 Check
```

Author Response: The structure is affected by some strain. Crystal imperfections can not be excluded either.

```
PLAT703_ALERT_1_C Torsion Calc    -80.2(5), Rep    -79.6(7), Dev..       1.20 Sigma
              O(1)-S(2)-C(33-C(23  1_555  1_555  1_555  1_555      #   16 Check
```

Author Response: The structure is affected by some strain. Crystal imperfections can not be excluded either.

```
PLAT703_ALERT_1_C Torsion Calc    148.9(5), Rep    149.7(7), Dev..       1.60 Sigma
              O(5)-S(2)-C(33-C(23  1_555  1_555  1_555  1_555      #   20 Check
```

Author Response: The structure is affected by some strain. Crystal imperfections can not be excluded either.

```
PLAT703_ALERT_1_C Torsion Calc     37.3(5), Rep     38.2(8), Dev..       1.80 Sigma
              N(2)-S(2)-C(33-C(23  1_555  1_555  1_555  1_555      #   22 Check
```

Author Response: The structure is affected by some strain. Crystal imperfections can not be excluded either.

```
PLAT703_ALERT_1_C Torsion Calc    170.7(6), Rep    169.8(9), Dev..       1.50 Sigma
              C(26-N(1)-N(2)-C(51  1_555  1_555  1_555  1_555      #   28 Check
```

Author Response: The structure is affected by some strain. Crystal imperfections can not be excluded either.

```
PLAT703_ALERT_1_C Torsion Calc    -38.4(6), Rep   -39.1(10), Dev..       1.17 Sigma
              C(19-N(4)-N(8)-S(1)  1_555  1_555  1_555  1_555      #   34 Check
```

Author Response: The structure is affected by some strain. Crystal imperfections can not be excluded either.

```
PLAT703_ALERT_1_C Torsion Calc   -176.6(6), Rep   -177.3(9), Dev..       1.17 Sigma
              C(28-C(2)-C(38-C(19  1_555  1_555  1_555  1_555      #   53 Check
```

Author Response: The structure is affected by some strain. Crystal imperfections can not be excluded either.

```
PLAT703_ALERT_1_C Torsion Calc    -4.7(10), Rep    -5.9(15), Dev..       1.20 Sigma
              C(27-C(3)-C(41-C(34  1_555  1_555  1_555  1_555      #   57 Check
```

Author Response: The structure is affected by some strain. Crystal imperfections can not be excluded either.

```
PLAT703_ALERT_1_C Torsion Calc      1.0(9), Rep    -0.3(11), Dev..       1.44 Sigma
              C(41-C(3)-C(27-C(31  1_555  1_555  1_555  1_555      #   58 Check
```

Author Response: The structure is affected by some strain. Crystal imperfections can not be excluded either.

```
PLAT703_ALERT_1_C Torsion Calc    175.7(6), Rep    174.6(8), Dev..       1.83 Sigma
              N(3)-C(16-C(20-C(18  1_555  1_555  1_555  1_555      #   60 Check
```

Author Response: The structure is affected by some strain. Crystal imperfections can not be excluded either.

```
PLAT703_ALERT_1_C Torsion Calc      3.4(9), Rep     2.4(12), Dev..       1.11 Sigma
              C(20-C(16-C(33-C(23  1_555  1_555  1_555  1_555      #   64 Check
```

Author Response: The structure is affected by some strain. Crystal imperfections can not be excluded either.

```
PLAT703_ALERT_1_C Torsion Calc     5.0(10), Rep     6.2(13), Dev..       1.20 Sigma
              C(36-C(18-C(20-C(16  1_555  1_555  1_555  1_555      #   67 Check
```

Author Response: The structure is affected by some strain. Crystal imperfections can not be excluded either.

```
PLAT703_ALERT_1_C Torsion Calc   -168.7(6), Rep   -169.5(9), Dev..       1.33 Sigma
              N(4)-C(19-C(38-C(47  1_555  1_555  1_555  1_555      #   69 Check
```

Author Response: The structure is affected by some strain. Crystal imperfections can not be excluded either.

```
PLAT703_ALERT_1_C Torsion Calc     10.6(9), Rep     9.3(14), Dev..       1.44 Sigma
              C(32-C(19-C(38-C(47  1_555  1_555  1_555  1_555      #   71 Check
```

Author Response: The structure is affected by some strain. Crystal imperfections can not be excluded either.

```
PLAT703_ALERT_1_C Torsion Calc    -11.8(8), Rep   -13.2(12), Dev..       1.75 Sigma
              C(26-C(23-C(33-S(2)  1_555  1_555  1_555  1_555      #   72 Check
```

Author Response: The structure is affected by some strain. Crystal imperfections can not be excluded either.

```
PLAT703_ALERT_1_C Torsion Calc   -12.6(10), Rep   -11.5(15), Dev..       1.10 Sigma
              C(33-C(23-C(26-N(1)  1_555  1_555  1_555  1_555      #   74 Check
```

Author Response: The structure is affected by some strain. Crystal imperfections can not be excluded either.

```
PLAT703_ALERT_1_C Torsion Calc   -175.9(7), Rep   -174.6(9), Dev..       1.86 Sigma
              C(26-C(23-C(36-C(18  1_555  1_555  1_555  1_555      #   76 Check
```

Author Response: The structure is affected by some strain. Crystal imperfections can not be excluded either.

```
PLAT703_ALERT_1_C Torsion Calc    165.6(7), Rep   164.6(10), Dev..       1.43 Sigma
              C(36-C(23-C(26-N(1)  1_555  1_555  1_555  1_555      #   77 Check
```

Author Response: The structure is affected by some strain. Crystal imperfections can not be excluded either.

```
PLAT703_ALERT_1_C Torsion Calc    169.8(5), Rep    170.5(7), Dev..       1.40 Sigma
              C(36-C(23-C(33-S(2)  1_555  1_555  1_555  1_555      #   80 Check
```

Author Response: The structure is affected by some strain. Crystal imperfections can not be excluded either.

```
PLAT703_ALERT_1_C Torsion Calc     -2.1(9), Rep    -0.5(11), Dev..       1.78 Sigma
              C(36-C(23-C(33-C(16  1_555  1_555  1_555  1_555      #   81 Check
```

Author Response: The structure is affected by some strain. Crystal imperfections can not be excluded either.

```
PLAT703_ALERT_1_C Torsion Calc    175.9(6), Rep    176.7(9), Dev..       1.33 Sigma
              C(40-C(25-C(42-C(46  1_555  1_555  1_555  1_555      #   89 Check
```

Author Response: The structure is affected by some strain. Crystal imperfections can not be excluded either.

```
PLAT703_ALERT_1_C Torsion Calc   -177.7(5), Rep   -178.6(8), Dev..       1.80 Sigma
              C(3)-C(27-C(49-C(35  1_555  1_555  1_555  1_555      #   94 Check
```

Author Response: The structure is affected by some strain. Crystal imperfections can not be excluded either.

```
PLAT703_ALERT_1_C Torsion Calc   -173.5(6), Rep   -172.3(9), Dev..       2.00 Sigma
              C(31-C(27-C(49-N(10  1_555  1_555  1_555  1_555      #   95 Check
```

Author Response: The structure is affected by some strain. Crystal imperfections can not be excluded either.

```
PLAT703_ALERT_1_C Torsion Calc     1.0(11), Rep     2.3(16), Dev..       1.18 Sigma
              C(45-C(29-C(30-C(40  1_555  1_555  1_555  1_555      #  102 Check
```

Author Response: The structure is affected by some strain. Crystal imperfections can not be excluded either.

```
PLAT703_ALERT_1_C Torsion Calc     2.8(10), Rep     4.4(14), Dev..       1.60 Sigma
              C(24-C(34-C(41-C(3)  1_555  1_555  1_555  1_555      #  106 Check
```

Author Response: The structure is affected by some strain. Crystal imperfections can not be excluded either.

```
PLAT703_ALERT_1_C Torsion Calc     50.9(7), Rep    50.0(10), Dev..       1.29 Sigma
              C(1)-C(37-C(48-C(46  1_555  1_555  1_555  1_555      #  110 Check
```

Author Response: The structure is affected by some strain. Crystal imperfections can not be excluded either.

```
PLAT703_ALERT_1_C Torsion Calc     35.3(9), Rep    36.8(12), Dev..       1.67 Sigma
              C(25-C(42-C(46-C(48  1_555  1_555  1_555  1_555      #  115 Check
```

Author Response: The structure is affected by some strain. Crystal imperfections can not be excluded either.

```
PLAT703_ALERT_1_C Torsion Calc    -55.2(7), Rep   -54.0(10), Dev..       1.71 Sigma
              C(42-C(46-C(48-C(37  1_555  1_555  1_555  1_555      #  118 Check
```

Author Response: The structure is affected by some strain. Crystal imperfections can not be excluded either.

```
PLAT703_ALERT_1_C Torsion Calc    -5.0(10), Rep    -6.9(15), Dev..       1.90 Sigma
              C(38-C(47-C(52-C(50  1_555  1_555  1_555  1_555      #  119 Check
```

Author Response: The structure is affected by some strain. Crystal imperfections can not be excluded either.

Alert level G

```
PLAT005_ALERT_5_G No Embedded Refinement Details Found  in the CIF     Please Do !
```

```
PLAT007_ALERT_5_G Number of Unrefined Donor-H Atoms .............          2 Report
              H1    H2
PLAT199_ALERT_1_G Reported _cell_measurement_temperature ..... (K)        293 Check
PLAT200_ALERT_1_G Reported   _diffrn_ambient_temperature ..... (K)        293 Check
PLAT790_ALERT_4_G Centre of Gravity not Within Unit Cell: Resd.  #          2 Note
              C19 H20 N4 O2 S
PLAT808_ALERT_5_G No Parseable SHELXL Style Weighting Scheme Found     Please Check
PLAT882_ALERT_1_G No Datum for _diffrn_reflns_av_unetI/netI ......     Please Do !
PLAT883_ALERT_1_G Absent Datum for _atom_sites_solution_primary ..     Please Do !
```

```
  18 ALERT level A = Most likely a serious problem - resolve or explain
  26 ALERT level B = A potentially serious problem, consider carefully
  52 ALERT level C = Check. Ensure it is not caused by an omission or oversight
   8 ALERT level G = General information/check it is not something unexpected

  69 ALERT type 1 CIF construction/syntax error, inconsistent or missing data
  28 ALERT type 2 Indicator that the structure model may be wrong or deficient
   3 ALERT type 3 Indicator that the structure quality may be low
   1 ALERT type 4 Improvement, methodology, query or suggestion
   3 ALERT type 5 Informative message, check
```

It is advisable to attempt to resolve as many as possible of the alerts in all categories. Often the minor alerts point to easily fixed oversights, errors and omissions in your CIF or refinement strategy, so attention to these fine details can be worthwhile. In order to resolve some of the more serious problems it may be necessary to carry out additional measurements or structure refinements. However, the purpose of your study may justify the reported deviations and the more serious of these should normally be commented upon in the discussion or experimental section of a paper or in the "special_details" fields of the CIF. checkCIF was carefully designed to identify outliers and unusual parameters, but every test has its limitations and alerts that are not important in a particular case may appear. Conversely, the absence of alerts does not guarantee there are no aspects of the results needing attention. It is up to the individual to critically assess their own results and, if necessary, seek expert advice.

Publication of your CIF in IUCr journals

A basic structural check has been run on your CIF. These basic checks will be run on all CIFs submitted for publication in IUCr journals (*Acta Crystallographica*, *Journal of Applied Crystallography*, *Journal of Synchrotron Radiation*); however, if you intend to submit to *Acta Crystallographica Section C* or *E* or *IUCrData*, you should make sure that full publication checks are run on the final version of your CIF prior to submission.

Publication of your CIF in other journals

Please refer to the *Notes for Authors* of the relevant journal for any special instructions relating to CIF submission.

Validation response form

Please find below a validation response form (VRF) that can be filled in and pasted into your CIF.

```
# start Validation Reply Form
_vrf_REFLT02__143519_2
;
PROBLEM: The number of reflections greater than the sigma threshold
RESPONSE: ...
;
_vrf_DIFMN03__143519_2
;
PROBLEM: The minimum difference density is < -0.1*ZMAX*0.75
RESPONSE: ...
;
_vrf_DIFMX02__143519_2
;
PROBLEM: The maximum difference density is > 0.1*ZMAX*0.75
RESPONSE: ...
;
_vrf_PLAT111__143519_2
;
PROBLEM: ADDSYM Detects New (Pseudo) Centre of Symmetry .        100 %Fit
RESPONSE: ...
;
_vrf_PLAT113__143519_2
;
PROBLEM: ADDSYM Suggests Possible Pseudo/New  Space Group        P-1 Check
RESPONSE: ...
;
_vrf_PLAT230__143519_2
;
PROBLEM: Hirshfeld Test Diff for    S1       --O3       .       10.0 s.u.
RESPONSE: ...
;
_vrf_PLAT034__143519_2
;
PROBLEM: No Flack Parameter Given. Z > Si, NonCentro ....     Please Do !
RESPONSE: ...
;
_vrf_PLAT089__143519_2
;
PROBLEM: Poor Data / Parameter Ratio (Zmax < 18) ........       6.71 Note
RESPONSE: ...
;
_vrf_PLAT213__143519_2
;
PROBLEM: Atom C21             has ADP max/min Ratio .....        3.1 prolat
RESPONSE: ...
;
_vrf_PLAT241__143519_2
;
PROBLEM: High   'MainMol' Ueq as Compared to Neighbors of        C39 Check
RESPONSE: ...
;
_vrf_PLAT340__143519_2
;
PROBLEM: Low Bond Precision on  C-C Bonds ..............    0.00876 Ang.
RESPONSE: ...
```

```
;
_vrf_PLAT369___143519_2
;
PROBLEM: Long   C(sp2)-C(sp2) Bond  C1        - C25       .       1.54 Ang.
RESPONSE: ...
;
_vrf_PLAT414___143519_2
;
PROBLEM: Short Intra D-H..H-X       H1        ..H18       .       1.95 Ang.
RESPONSE: ...
;
# end Validation Reply Form
```

PLATON version of 02/02/2025; check.def file version of 02/02/2025

Datablock _143519_2 - ellipsoid plot

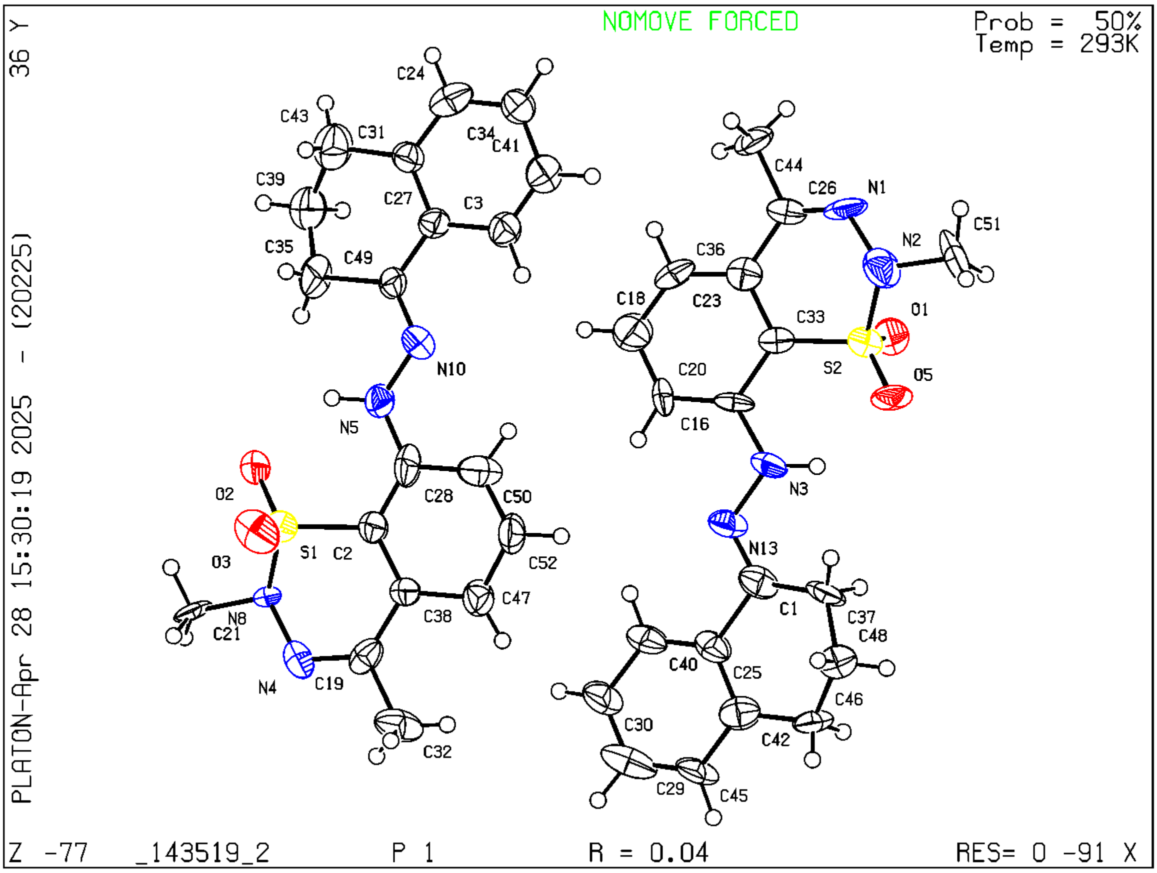

Supplement: File 2 — Crystallographic information files, checkcif and structure report files for compounds 3b, 3d, 3e, 3g, 3h, (E)-7a, 7b, 7d, 7e, (E)-7f, (Z)-7h, 7i and (E)-9a. [file (E)-9a_checkcif.pdf › Átnevezett XRD/(E)-9a_checkcif.pdf]
