# Supplementary material for: Thiadiazino-indole, thiadiazino-carbazole and benzothiadiazino-carbazole dioxides: synthesis, physicochemical and early ADME characterization of representatives of new tri-, tetra- and pentacyclic ring systems and their intermediates
Source: Beilstein J Org Chem. 2025 Oct 21;21:2220–33. doi: 10.3762/bjoc.21.169 (PMC12557438; doi:10.3762/bjoc.21.169)

# checkCIF/PLATON report

You have not supplied any structure factors. As a result the full set of tests cannot be run.

THIS REPORT IS FOR GUIDANCE ONLY. IF USED AS PART OF A REVIEW PROCEDURE FOR PUBLICATION, IT SHOULD NOT REPLACE THE EXPERTISE OF AN EXPERIENCED CRYSTALLOGRAPHIC REFEREE.

No syntax errors found.  CIF dictionary  Interpreting this report

# Datablock: _142611_1

---

```
Bond precision:   C-C = 0.0023 A          Wavelength=1.54187

Cell:              a=7.6009(5)     b=17.131(1)        c=10.9012(8)
                   alpha=90        beta=95.705(4)     gamma=90
Temperature:       293 K

                 Calculated                   Reported
Volume           1412.43(16)                  1412.43(16)
Space group      P 21/a                       P 1 21/a 1
Hall group       -P 2yab                      -P 2yab
Moiety formula   C14 H17 N3 O2 S              C14 H17 N3 O2 S
Sum formula      C14 H17 N3 O2 S              C14 H17 N3 O2 S
Mr               291.37                       291.37
Dx,g cm-3        1.370                        1.370
Z                4                            4
Mu (mm-1)        2.086                        2.086
F000             616.0                        616.0
F000'            619.00
h,k,lmax         9,21,13                      9,21,12
Nref             2745                         2587
Tmin,Tmax        0.669,0.882                  0.605,0.882
Tmin'            0.251

Correction method= # Reported T Limits: Tmin=0.605 Tmax=0.882
AbsCorr = MULTI-SCAN

Data completeness= 0.942           Theta(max)= 71.390

                                                     wR2(reflections)=
R(reflections)= 0.0510( 9521)                        0.0706( 9573)

S = 1.119                  Npar= 204
```

The following ALERTS were generated. Each ALERT has the format
**test-name_ALERT_alert-type_alert-level.**
Click on the hyperlinks for more details of the test.

**Alert level A**

```
DIFMN02_ALERT_2_A  The minimum difference density is < -0.1*ZMAX*2.00
            _refine_diff_density_min given =     -4.410
            Test value =     -3.200
```

**Author Response: Altough the residual density is out of border but there is no doubt about the chemical structure taking into account other evidences, e.g. NMR. The error maybe due to crystal imperfections.**

```
PLAT097_ALERT_2_A Large Reported Max.  (Positive) Residual Density       5.84 eA-3
```

**Author Response: Altough the residual density is out of border but there is no doubt about the chemical structure taking into account other evidences, e.g. NMR. The error maybe due to crystal imperfections.**

```
PLAT098_ALERT_2_A Large Reported Min.  (Negative) Residual Density      -4.41 eA-3
```

**Author Response: Altough the residual density is out of border but there is no doubt about the chemical structure taking into account other evidences, e.g. NMR. The error maybe due to crystal imperfections.**

```
PLAT703_ALERT_1_A Torsion Calc  176.52(12), Rep    176.9(2), Dev..       3.17 Sigma
              N(6)-N(5)-C(16-C(17  1_555  1_555  1_555  1_555      #   18 Check
```

**Author Response: The structre is affected by some strain. Crystal imperfections can not be excluded either.**

```
PLAT703_ALERT_1_A Torsion Calc -174.83(13), Rep   -174.3(2), Dev..       4.08 Sigma
              C(16-N(5)-N(6)-C(19  1_555  1_555  1_555  1_555      #   20 Check
```

**Author Response: The structre is affected by some strain. Crystal imperfections can not be excluded either.**

```
PLAT703_ALERT_1_A Torsion Calc -176.32(12), Rep   -176.8(2), Dev..       4.00 Sigma
              C(10-C(7)-C(15-S(1)  1_555  1_555  1_555  1_555      #   25 Check
```

**Author Response: The structre is affected by some strain. Crystal imperfections can not be excluded either.**

```
PLAT703_ALERT_1_A Torsion Calc  165.75(14), Rep    164.9(2), Dev..       6.07 Sigma
              C(15-C(11-C(16-C(17  1_555  1_555  1_555  1_555      #   43 Check
```

**Author Response: The structre is affected by some strain. Crystal imperfections can not be excluded either.**

```
PLAT703_ALERT_1_A Torsion Calc   69.09(19), Rep     69.9(3), Dev..       4.26 Sigma
              C(20-C(14-C(18-N(4)  1_555  1_555  1_555  1_555      #   50 Check
```

**Author Response: The structre is affected by some strain. Crystal imperfections can not be excluded either.**

```
PLAT703_ALERT_1_A Torsion Calc   -108.8(2), Rep   -107.6(3), Dev..       6.00 Sigma
              C(20-C(14-C(18-C(12  1_555  1_555  1_555  1_555      #   51 Check
```

**Author Response: The structre is affected by some strain. Crystal imperfections can not be excluded either.**

---

**Alert level B**

```
REFLT02_ALERT_1_B  The number of reflections greater than the sigma threshold
            cannot exceed the number of symmetry-independent reflections
            Number of symmetry-independent reflections =   2587
            Number of reflections greater than sigma threshold =   9521
PLAT029_ALERT_3_B _diffrn_measured_fraction_theta_full value Low .      0.942 Why?
PLAT230_ALERT_2_B Hirshfeld Test Diff for    S1       --O2       .        9.1 s.u.
PLAT230_ALERT_2_B Hirshfeld Test Diff for    S1       --O3       .        9.9 s.u.
PLAT230_ALERT_2_B Hirshfeld Test Diff for    S1       --N6       .        7.3 s.u.
PLAT230_ALERT_2_B Hirshfeld Test Diff for    C7       --C15      .        9.2 s.u.
PLAT230_ALERT_2_B Hirshfeld Test Diff for    C12      --C13      .        9.5 s.u.
PLAT703_ALERT_1_B Torsion Calc  150.26(12), Rep    149.9(2), Dev..       3.00 Sigma
              O(3)-S(1)-C(15-C(11  1_555  1_555  1_555  1_555      #    8 Check
```

**Author Response: The structre is affected by some strain. Crystal imperfections can not be excluded either.**

```
PLAT703_ALERT_1_B Torsion Calc    0.58(18), Rep      0.2(2), Dev..       2.11 Sigma
              C(7)-N(4)-C(18-C(12  1_555  1_555  1_555  1_555      #   13 Check
```

**Author Response: The structre is affected by some strain. Crystal imperfections can not be excluded either.**

```
PLAT703_ALERT_1_B Torsion Calc   -0.57(17), Rep     -0.1(2), Dev..       2.76 Sigma
              C(18-N(4)-C(7)-C(10  1_555  1_555  1_555  1_555      #   15 Check
```

**Author Response: The structre is affected by some strain. Crystal imperfections can not be excluded either.**

```
PLAT703_ALERT_1_B Torsion Calc -179.61(16), Rep   -179.2(2), Dev..       2.56 Sigma
              N(4)-C(7)-C(15-C(11  1_555  1_555  1_555  1_555      #   24 Check
```

**Author Response: The structre is affected by some strain. Crystal imperfections can not be excluded either.**

```
PLAT703_ALERT_1_B Torsion Calc     -0.4(2), Rep      0.1(3), Dev..       2.50 Sigma
              C(15-C(7)-C(10-C(9)  1_555  1_555  1_555  1_555      #   27 Check
```

**Author Response: The structre is affected by some strain. Crystal imperfections can not be excluded either.**

```
PLAT703_ALERT_1_B Torsion Calc  179.66(15), Rep   -180.0(2), Dev..       2.27 Sigma
              C(15-C(7)-C(10-C(12  1_555  1_555  1_555  1_555      #   28 Check
```

**Author Response: The structre is affected by some strain. Crystal imperfections can not be excluded either.**

```
PLAT703_ALERT_1_B Torsion Calc    -17.8(2), Rep    -18.4(4), Dev..       3.00 Sigma
              C(8)-C(11-C(16-C(17  1_555  1_555  1_555  1_555      #   41 Check
```

**Author Response: The structre is affected by some strain. Crystal imperfections can not be excluded either.**

```
PLAT703_ALERT_1_B Torsion Calc    -16.8(2), Rep    -17.3(4), Dev..       2.50 Sigma
              C(15-C(11-C(16-N(5)  1_555  1_555  1_555  1_555      #   42 Check
```

**Author Response: The structre is affected by some strain. Crystal imperfections can not be excluded either.**

---

### Alert level C

```
DIFMN03_ALERT_1_C  The minimum difference density is < -0.1*ZMAX*0.75
            The relevant atom site should be identified.
DIFMX02_ALERT_1_C  The maximum difference density is > 0.1*ZMAX*0.75
            The relevant atom site should be identified.
PLAT166_ALERT_4_C S.U's Given on Coordinates for Calc-flagged ....          H1 Note
PLAT166_ALERT_4_C S.U's Given on Coordinates for Calc-flagged ....          H2 Note
PLAT220_ALERT_2_C NonSolvent   Resd 1  C   Ueq(max)/Ueq(min) Range        3.1 Ratio
PLAT230_ALERT_2_C Hirshfeld Test Diff for    N5       --N6       .        6.1 s.u.
PLAT230_ALERT_2_C Hirshfeld Test Diff for    C11      --C16      .        5.5 s.u.
PLAT703_ALERT_1_C Torsion Calc   98.60(14), Rep     98.4(2), Dev..       1.43 Sigma
              O(2)-S(1)-C(15-C(7)  1_555  1_555  1_555  1_555      #    3 Check
```

**Author Response: The structre is affected by some strain. Crystal imperfections can not be excluded either.**

```
PLAT703_ALERT_1_C Torsion Calc  -79.05(13), Rep    -79.3(2), Dev..       1.92 Sigma
              O(2)-S(1)-C(15-C(11  1_555  1_555  1_555  1_555      #    4 Check
```

**Author Response: The structre is affected by some strain. Crystal imperfections can not be excluded either.**

```
PLAT703_ALERT_1_C Torsion Calc  -32.08(16), Rep    -32.4(2), Dev..       2.00 Sigma
              O(3)-S(1)-C(15-C(7)  1_555  1_555  1_555  1_555      #    7 Check
```

**Author Response: The structre is affected by some strain. Crystal imperfections can not be excluded either.**

```
PLAT703_ALERT_1_C Torsion Calc -145.05(14), Rep   -144.8(2), Dev..       1.79 Sigma
              N(6)-S(1)-C(15-C(7)  1_555  1_555  1_555  1_555      #    9 Check
```

**Author Response: The structre is affected by some strain. Crystal imperfections can not be excluded either.**

```
PLAT703_ALERT_1_C Torsion Calc   37.30(14), Rep     37.5(2), Dev..       1.43 Sigma
              N(6)-S(1)-C(15-C(11  1_555  1_555  1_555  1_555      #   10 Check
```

**Author Response: The structre is affected by some strain. Crystal imperfections can not be excluded either.**

```
PLAT703_ALERT_1_C Torsion Calc  -56.42(12), Rep    -56.6(2), Dev..       1.50 Sigma
              C(15-S(1)-N(6)-N(5)  1_555  1_555  1_555  1_555      #   11 Check
```

**Author Response: The structre is affected by some strain. Crystal imperfections can not be excluded either.**

```
PLAT703_ALERT_1_C Torsion Calc  163.17(12), Rep    163.0(2), Dev..       1.42 Sigma
              C(15-S(1)-N(6)-C(19  1_555  1_555  1_555  1_555      #   12 Check
```

**Author Response: The structre is affected by some strain. Crystal imperfections can not be excluded either.**

```
PLAT703_ALERT_1_C Torsion Calc -179.78(17), Rep    179.9(2), Dev..       1.88 Sigma
              C(18-N(4)-C(7)-C(15  1_555  1_555  1_555  1_555      #   16 Check
```

**Author Response: The structre is affected by some strain. Crystal imperfections can not be excluded either.**

```
PLAT703_ALERT_1_C Torsion Calc   43.16(17), Rep     43.5(3), Dev..       2.00 Sigma
              C(16-N(5)-N(6)-S(1)  1_555  1_555  1_555  1_555      #   19 Check
```

**Author Response: The structre is affected by some strain. Crystal imperfections can not be excluded either.**

```
PLAT703_ALERT_1_C Torsion Calc -179.73(14), Rep   -179.9(2), Dev..       1.21 Sigma
              N(4)-C(7)-C(10-C(9)  1_555  1_555  1_555  1_555      #   21 Check
```

**Author Response: The structre is affected by some strain. Crystal imperfections can not be excluded either.**

```
PLAT703_ALERT_1_C Torsion Calc    0.35(17), Rep      0.1(2), Dev..       1.47 Sigma
              N(4)-C(7)-C(10-C(12  1_555  1_555  1_555  1_555      #   22 Check
```

**Author Response: The structre is affected by some strain. Crystal imperfections can not be excluded either.**

```
PLAT703_ALERT_1_C Torsion Calc      1.3(2), Rep      0.9(4), Dev..       2.00 Sigma
              C(10-C(7)-C(15-C(11  1_555  1_555  1_555  1_555      #   26 Check
```

**Author Response: The structre is affected by some strain. Crystal imperfections can not be excluded either.**

```
PLAT703_ALERT_1_C Torsion Calc     -0.1(2), Rep     -0.4(4), Dev..       1.50 Sigma
              C(8)-C(9)-C(10-C(7)  1_555  1_555  1_555  1_555      #   32 Check
```

**Author Response: The structre is affected by some strain. Crystal imperfections can not be excluded either.**

```
PLAT703_ALERT_1_C Torsion Calc -179.27(15), Rep   -179.0(2), Dev..       1.80 Sigma
              C(7)-C(10-C(12-C(13  1_555  1_555  1_555  1_555      #   34 Check
```

**Author Response: The structre is affected by some strain. Crystal imperfections can not be excluded either.**

```
PLAT703_ALERT_1_C Torsion Calc  176.17(12), Rep    176.3(2), Dev..       1.08 Sigma
              C(8)-C(11-C(15-S(1)  1_555  1_555  1_555  1_555      #   38 Check
```

**Author Response: The structre is affected by some strain. Crystal imperfections can not be excluded either.**

```
PLAT703_ALERT_1_C Torsion Calc  159.71(16), Rep    159.4(2), Dev..       1.94 Sigma
              C(8)-C(11-C(16-N(5)  1_555  1_555  1_555  1_555      #   40 Check
```

**Author Response: The structre is affected by some strain. Crystal imperfections can not be excluded either.**

```
PLAT703_ALERT_1_C Torsion Calc     -7.2(2), Rep     -6.9(3), Dev..       1.50 Sigma
              C(16-C(11-C(15-S(1)  1_555  1_555  1_555  1_555      #   44 Check
```

**Author Response: The structre is affected by some strain. Crystal imperfections can not be excluded either.**

```
PLAT703_ALERT_1_C Torsion Calc  175.07(15), Rep    175.3(2), Dev..       1.53 Sigma
              C(16-C(11-C(15-C(7)  1_555  1_555  1_555  1_555      #   45 Check
```

**Author Response: The structre is affected by some strain. Crystal imperfections can not be excluded either.**

```
PLAT703_ALERT_1_C Torsion Calc   -0.35(18), Rep     -0.1(2), Dev..       1.39 Sigma
              C(10-C(12-C(18-N(4)  1_555  1_555  1_555  1_555      #   46 Check
```

**Author Response: The structre is affected by some strain. Crystal imperfections can not be excluded either.**

```
PLAT703_ALERT_1_C Torsion Calc  177.72(16), Rep    177.5(2), Dev..       1.38 Sigma
              C(10-C(12-C(18-C(14  1_555  1_555  1_555  1_555      #   47 Check
```

**Author Response: The structre is affected by some strain. Crystal imperfections can not be excluded either.**

```
PLAT703_ALERT_1_C Torsion Calc     -3.0(3), Rep     -3.4(5), Dev..       1.33 Sigma
              C(13-C(12-C(18-C(14  1_555  1_555  1_555  1_555      #   49 Check
```

**Author Response: The structre is affected by some strain. Crystal imperfections can not be excluded either.**

---

**Alert level G**

```
PLAT005_ALERT_5_G No Embedded Refinement Details Found  in the CIF     Please Do !
PLAT007_ALERT_5_G Number of Unrefined Donor-H Atoms .............           1 Report
              H3
PLAT063_ALERT_4_G Crystal Size Possibly too Large for Beam Size ..       0.64 mm
PLAT128_ALERT_4_G Alternate Setting for Input Space Group  P21/a        P21/c Note
PLAT199_ALERT_1_G Reported _cell_measurement_temperature ..... (K)        293 Check
PLAT200_ALERT_1_G Reported   _diffrn_ambient_temperature ..... (K)        293 Check
PLAT808_ALERT_5_G No Parseable SHELXL Style Weighting Scheme Found     Please Check
PLAT882_ALERT_1_G No Datum for _diffrn_reflns_av_unetI/netI ......     Please Do !
PLAT883_ALERT_1_G Absent Datum for _atom_sites_solution_primary ..     Please Do !
```

---

```
 9 ALERT level A = Most likely a serious problem - resolve or explain
15 ALERT level B = A potentially serious problem, consider carefully
28 ALERT level C = Check. Ensure it is not caused by an omission or oversight
 9 ALERT level G = General information/check it is not something unexpected

42 ALERT type 1 CIF construction/syntax error, inconsistent or missing data
11 ALERT type 2 Indicator that the structure model may be wrong or deficient
 1 ALERT type 3 Indicator that the structure quality may be low
 4 ALERT type 4 Improvement, methodology, query or suggestion
 3 ALERT type 5 Informative message, check
```

---

It is advisable to attempt to resolve as many as possible of the alerts in all categories. Often the minor alerts point to easily fixed oversights, errors and omissions in your CIF or refinement strategy, so attention to these fine details can be worthwhile. In order to resolve some of the more serious problems it may be necessary to carry out additional measurements or structure refinements. However, the purpose of your study may justify the reported deviations and the more serious of these should normally be commented upon in the discussion or experimental section of a paper or in the "special_details" fields of the CIF. checkCIF was carefully designed to identify outliers and unusual parameters, but every test has its limitations and alerts that are not important in a particular case may appear. Conversely, the absence of alerts does not guarantee there are no aspects of the results needing attention. It is up to the individual to critically assess their own results and, if necessary, seek expert advice.

**Publication of your CIF in IUCr journals**

A basic structural check has been run on your CIF. These basic checks will be run on all CIFs submitted for publication in IUCr journals (*Acta Crystallographica*, *Journal of Applied Crystallography*, *Journal of Synchrotron Radiation*); however, if you intend to submit to *Acta Crystallographica Section C* or *E* or *IUCrData*, you should make sure that full publication checks are run on the final version of your CIF prior to submission.

**Publication of your CIF in other journals**

Please refer to the *Notes for Authors* of the relevant journal for any special instructions relating to CIF submission.

## Validation response form

Please find below a validation response form (VRF) that can be filled in and pasted into your CIF.

```
# start Validation Reply Form
_vrf_REFLT02__142611_1
;
PROBLEM: The number of reflections greater than the sigma threshold
RESPONSE: ...
;
_vrf_DIFMN03__142611_1
;
PROBLEM: The minimum difference density is < -0.1*ZMAX*0.75
RESPONSE: ...
```

```
;
_vrf_DIFMX02__142611_1
;
PROBLEM: The maximum difference density is > 0.1*ZMAX*0.75
RESPONSE: ...
;
_vrf_PLAT029__142611_1
;
PROBLEM: _diffrn_measured_fraction_theta_full value Low .      0.942 Why?
RESPONSE: ...
;
_vrf_PLAT230__142611_1
;
PROBLEM: Hirshfeld Test Diff for    S1       --O2       .        9.1 s.u.
RESPONSE: ...
;
_vrf_PLAT166__142611_1
;
PROBLEM: S.U's Given on Coordinates for Calc-flagged ....          H1 Note
RESPONSE: ...
;
_vrf_PLAT220__142611_1
;
PROBLEM: NonSolvent   Resd 1  C   Ueq(max)/Ueq(min) Range        3.1 Ratio
RESPONSE: ...
;
# end Validation Reply Form
```

---

**PLATON version of 02/02/2025; check.def file version of 02/02/2025**

**Datablock _142611_1** - ellipsoid plot

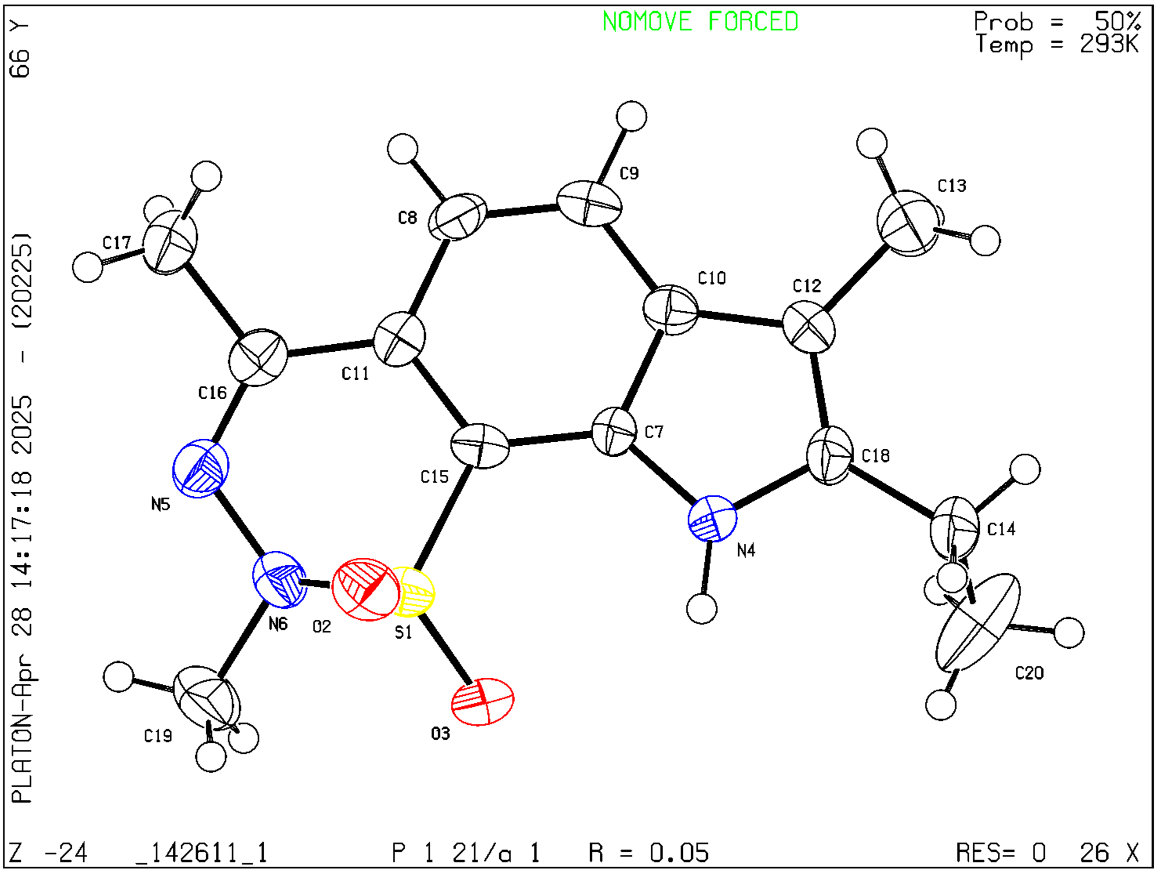

Supplement: File 2 — Crystallographic information files, checkcif and structure report files for compounds 3b, 3d, 3e, 3g, 3h, (E)-7a, 7b, 7d, 7e, (E)-7f, (Z)-7h, 7i and (E)-9a. [file 3b_checkcif.pdf › Átnevezett XRD/3b_checkcif.pdf]
