# Supplementary material for: Thiadiazino-indole, thiadiazino-carbazole and benzothiadiazino-carbazole dioxides: synthesis, physicochemical and early ADME characterization of representatives of new tri-, tetra- and pentacyclic ring systems and their intermediates
Source: Beilstein J Org Chem. 2025 Oct 21;21:2220–33. doi: 10.3762/bjoc.21.169 (PMC12557438; doi:10.3762/bjoc.21.169)

checkCIF/PLATON report

You have not supplied any structure factors. As a result the full set of tests cannot be run.

THIS REPORT IS FOR GUIDANCE ONLY. IF USED AS PART OF A REVIEW PROCEDURE FOR PUBLICATION, IT SHOULD NOT REPLACE THE EXPERTISE OF AN EXPERIENCED CRYSTALLOGRAPHIC REFEREE.

No syntax errors found. CIF dictionary Interpreting this report

Datablock: _142501_1

```
Bond precision:   C-C = 0.0030 A          Wavelength=1.54187

Cell:            a=12.9021(7)    b=11.1229(6)       c=20.2138(14)
                 alpha=90        beta=100.079(4)    gamma=90
Temperature:     293 K

                Calculated                    Reported
Volume          2856.1(3)                     2856.1(3)
Space group     P 21/n                        P 1 21/n 1
Hall group      -P 2yn                        -P 2yn
Moiety formula  C15 H17 N3 O2 S               C15 H17 N3 O2 S
Sum formula     C15 H17 N3 O2 S               C15 H17 N3 O2 S
Mr              303.38                        303.38
Dx,g cm-3       1.411                         1.411
Z               8                             8
Mu (mm-1)       2.089                         2.089
F000            1280.0                        1280.0
F000'           1286.14
h,k,lmax        15,13,24                      15,13,23
Nref            5587                          5330
Tmin,Tmax       0.575,0.778                   0.441,0.773
Tmin'           0.250

Correction method= # Reported T Limits: Tmin=0.441 Tmax=0.773
AbsCorr = MULTI-SCAN

Data completeness= 0.954          Theta(max)= 71.740

                                                 wR2(reflections)=
R(reflections)= 0.0819( 24540)                   0.1403( 24702)

S = 0.944                   Npar= 413
```

The following ALERTS were generated. Each ALERT has the format
test-name_ALERT_alert-type_alert-level.
Click on the hyperlinks for more details of the test.

Alert level A

```
DIFMN02_ALERT_2_A  The minimum difference density is < -0.1*ZMAX*2.00
            _refine_diff_density_min given =      -8.210
            Test value =      -3.200
```

Author Response: Altough the residual density is out of border but there is no doubt about the chemical structure taking into account other evidences, e.g. NMR. The error maybe due to crystal imperfections.

```
PLAT097_ALERT_2_A Large Reported Max.  (Positive) Residual Density       8.13 eA-3
```

Author Response: Altough the residual density is out of border but there is no doubt about the chemical structure taking into account other evidences, e.g. NMR. The error maybe due to crystal imperfections.

```
PLAT098_ALERT_2_A Large Reported Min.  (Negative) Residual Density      -8.21 eA-3
```

Author Response: Altough the residual density is out of border but there is no doubt about the chemical structure taking into account other evidences, e.g. NMR. The error maybe due to crystal imperfections.

```
PLAT703_ALERT_1_A Torsion Calc  163.61(12), Rep    163.1(2), Dev..       4.25 Sigma
              O(3)-S(1)-N(8)-N(10  1_555  1_555  1_555  1_555      #    1 Check
```

Author Response: The structre is affected by some strain. Crystal imperfections can not be excluded either.

```
PLAT703_ALERT_1_A Torsion Calc   39.77(19), Rep     41.0(3), Dev..       6.47 Sigma
              O(3)-S(1)-C(18-C(13  1_555  1_555  1_555  1_555      #    3 Check
```

Author Response: The structre is affected by some strain. Crystal imperfections can not be excluded either.

```
PLAT703_ALERT_1_A Torsion Calc   66.12(15), Rep     67.3(2), Dev..       7.87 Sigma
              O(5)-S(1)-N(8)-C(34  1_555  1_555  1_555  1_555      #    6 Check
```

Author Response: The structre is affected by some strain. Crystal imperfections can not be excluded either.

```
PLAT703_ALERT_1_A Torsion Calc  -90.18(17), Rep    -89.5(3), Dev..       4.00 Sigma
             O(5)-S(1)-C(18-C(13  1_555  1_555  1_555  1_555      #    7 Check
```

Author Response: The structre is affected by some strain. Crystal imperfections can not be excluded either.

```
PLAT703_ALERT_1_A Torsion Calc   83.14(17), Rep     82.3(3), Dev..       4.94 Sigma
             O(5)-S(1)-C(18-C(20  1_555  1_555  1_555  1_555      #    8 Check
```

Author Response: The structre is affected by some strain. Crystal imperfections can not be excluded either.

```
PLAT703_ALERT_1_A Torsion Calc  -34.19(17), Rep    -35.3(3), Dev..       6.53 Sigma
             N(8)-S(1)-C(18-C(20  1_555  1_555  1_555  1_555      #   10 Check
```

Author Response: The structre is affected by some strain. Crystal imperfections can not be excluded either.

```
PLAT703_ALERT_1_A Torsion Calc -177.59(14), Rep   -176.8(2), Dev..       5.64 Sigma
             C(18-S(1)-N(8)-C(34  1_555  1_555  1_555  1_555      #   12 Check
```

Author Response: The structre is affected by some strain. Crystal imperfections can not be excluded either.

```
PLAT703_ALERT_1_A Torsion Calc  144.77(16), Rep    143.4(3), Dev..       8.56 Sigma
             O(1)-S(2)-C(15-C(24  1_555  1_555  1_555  1_555      #   16 Check
```

Author Response: The structre is affected by some strain. Crystal imperfections can not be excluded either.

```
PLAT703_ALERT_1_A Torsion Calc   67.86(15), Rep     67.1(3), Dev..       5.07 Sigma
             O(6)-S(2)-N(9)-N(12  1_555  1_555  1_555  1_555      #   17 Check
```

Author Response: The structre is affected by some strain. Crystal imperfections can not be excluded either.

```
PLAT703_ALERT_1_A Torsion Calc  -64.65(16), Rep    -66.1(3), Dev..       9.06 Sigma
             O(6)-S(2)-N(9)-C(36  1_555  1_555  1_555  1_555      #   18 Check
```

Author Response: The structre is affected by some strain. Crystal imperfections can not be excluded either.

```
PLAT703_ALERT_1_A Torsion Calc   88.70(18), Rep     89.4(3), Dev..       3.89 Sigma
             O(6)-S(2)-C(15-C(22  1_555  1_555  1_555  1_555      #   19 Check
```

Author Response: The structre is affected by some strain. Crystal imperfections can not be excluded either.

```
PLAT703_ALERT_1_A Torsion Calc -154.58(17), Rep   -153.5(3), Dev..       6.35 Sigma
             N(9)-S(2)-C(15-C(22  1_555  1_555  1_555  1_555      #   21 Check
```

Author Response: The structre is affected by some strain. Crystal imperfections can not be excluded either.

```
PLAT703_ALERT_1_A Torsion Calc  179.40(15), Rep    178.3(2), Dev..       7.33 Sigma
             C(15-S(2)-N(9)-C(36  1_555  1_555  1_555  1_555      #   24 Check
```

Author Response: The structre is affected by some strain. Crystal imperfections can not be excluded either.

```
PLAT703_ALERT_1_A Torsion Calc    176.8(2), Rep    177.9(4), Dev..       5.50 Sigma
             C(21-N(1)-C(13-C(18  1_555  1_555  1_555  1_555      #   27 Check
```

Author Response: The structre is affected by some strain. Crystal imperfections can not be excluded either.

```
PLAT703_ALERT_1_A Torsion Calc     38.1(2), Rep     38.8(4), Dev..       3.50 Sigma
             S(2)-N(9)-N(12-C(27  1_555  1_555  1_555  1_555      #   31 Check
```

Author Response: The structre is affected by some strain. Crystal imperfections can not be excluded either.

```
PLAT703_ALERT_1_A Torsion Calc  171.66(18), Rep    172.7(4), Dev..       5.78 Sigma
             C(36-N(9)-N(12-C(27  1_555  1_555  1_555  1_555      #   32 Check
```

Author Response: The structre is affected by some strain. Crystal imperfections can not be excluded either.

```
PLAT703_ALERT_1_A Torsion Calc -174.84(16), Rep   -175.4(3), Dev..       3.50 Sigma
             N(8)-N(10-C(17-C(33  1_555  1_555  1_555  1_555      #   34 Check
```

Author Response: The structre is affected by some strain. Crystal imperfections can not be excluded either.

```
PLAT703_ALERT_1_A Torsion Calc     -6.0(3), Rep     -7.9(6), Dev..       6.33 Sigma
             N(1)-C(13-C(18-S(1)  1_555  1_555  1_555  1_555      #   41 Check
```

Author Response: The structre is affected by some strain. Crystal imperfections can not be excluded either.

```
PLAT703_ALERT_1_A Torsion Calc     -0.5(2), Rep      0.2(4), Dev..       3.50 Sigma
             N(1)-C(13-C(19-C(26  1_555  1_555  1_555  1_555      #   44 Check
```

Author Response: The structre is affected by some strain. Crystal imperfections can not be excluded either.

```
PLAT703_ALERT_1_A Torsion Calc      3.1(3), Rep      1.6(6), Dev..       5.00 Sigma
             C(18-C(13-C(19-C(14  1_555  1_555  1_555  1_555      #   45 Check
```

Author Response: The structre is affected by some strain. Crystal imperfections can not be excluded either.

```
PLAT703_ALERT_1_A Torsion Calc -177.32(18), Rep   -178.0(3), Dev..       3.78 Sigma
             C(18-C(13-C(19-C(26  1_555  1_555  1_555  1_555      #   46 Check
```

Author Response: The structre is affected by some strain. Crystal imperfections can not be excluded either.

```
PLAT703_ALERT_1_A Torsion Calc     -3.2(3), Rep     -1.9(5), Dev..       4.33 Sigma
             C(19-C(13-C(18-C(20  1_555  1_555  1_555  1_555      #   48 Check
```

Author Response: The structre is affected by some strain. Crystal imperfections can not be excluded either.

```
PLAT703_ALERT_1_A Torsion Calc     -0.8(3), Rep      0.3(5), Dev..       3.67 Sigma
             C(32-C(14-C(19-C(13  1_555  1_555  1_555  1_555      #   50 Check
```

Author Response: The structre is affected by some strain. Crystal imperfections can not be excluded either.

```
PLAT703_ALERT_1_A Torsion Calc     -0.5(3), Rep     -1.8(6), Dev..       4.33 Sigma
             C(22-C(15-C(24-C(30  1_555  1_555  1_555  1_555      #   57 Check
```

Author Response: The structre is affected by some strain. Crystal imperfections can not be excluded either.

```
PLAT703_ALERT_1_A Torsion Calc  167.82(19), Rep    166.3(4), Dev..       8.00 Sigma
             N(11-C(16-C(29-C(37  1_555  1_555  1_555  1_555      #   62 Check
```

Author Response: The structre is affected by some strain. Crystal imperfections can not be excluded either.

```
PLAT703_ALERT_1_A Torsion Calc    -16.0(3), Rep    -17.5(6), Dev..       5.00 Sigma
             C(25-C(16-C(29-C(37  1_555  1_555  1_555  1_555      #   63 Check
```

Author Response: The structre is affected by some strain. Crystal imperfections can not be excluded either.

```
PLAT703_ALERT_1_A Torsion Calc      8.8(3), Rep      9.8(5), Dev..       3.33 Sigma
             S(1)-C(18-C(20-C(17  1_555  1_555  1_555  1_555      #   70 Check
```

Author Response: The structre is affected by some strain. Crystal imperfections can not be excluded either.

```
PLAT703_ALERT_1_A Torsion Calc -172.22(15), Rep   -171.5(3), Dev..       4.80 Sigma
             S(1)-C(18-C(20-C(32  1_555  1_555  1_555  1_555      #   71 Check
```

Author Response: The structre is affected by some strain. Crystal imperfections can not be excluded either.

```
PLAT703_ALERT_1_A Torsion Calc    178.3(2), Rep    177.4(4), Dev..       4.50 Sigma
             C(13-C(19-C(26-C(35  1_555  1_555  1_555  1_555      #   75 Check
```

Author Response: The structre is affected by some strain. Crystal imperfections can not be excluded either.

```
PLAT703_ALERT_1_A Torsion Calc -178.24(18), Rep   -177.6(3), Dev..       3.56 Sigma
             N(1)-C(21-C(26-C(35  1_555  1_555  1_555  1_555      #   81 Check
```

Author Response: The structre is affected by some strain. Crystal imperfections can not be excluded either.

```
PLAT703_ALERT_1_A Torsion Calc    177.3(2), Rep    176.6(4), Dev..       3.50 Sigma
             C(28-C(23-C(25-C(16  1_555  1_555  1_555  1_555      #   94 Check
```

Author Response: The structre is affected by some strain. Crystal imperfections can not be excluded either.

```
PLAT703_ALERT_1_A Torsion Calc  169.89(19), Rep    168.8(3), Dev..       5.74 Sigma
             C(15-C(24-C(27-C(38  1_555  1_555  1_555  1_555      #   97 Check
```

Author Response: The structre is affected by some strain. Crystal imperfections can not be excluded either.

```
PLAT703_ALERT_1_A Torsion Calc      1.2(3), Rep      2.2(6), Dev..       3.33 Sigma
             C(15-C(24-C(30-C(28  1_555  1_555  1_555  1_555      #   98 Check
```

Author Response: The structre is affected by some strain. Crystal imperfections can not be excluded either.

```
PLAT703_ALERT_1_A Torsion Calc   -161.3(2), Rep   -159.9(4), Dev..       7.00 Sigma
           C(19-C(26-C(35-C(41  1_555  1_555  1_555  1_555      #  104 Check
```

Author Response: The structre is affected by some strain. Crystal imperfections can not be excluded either.

```
PLAT703_ALERT_1_A Torsion Calc     16.4(3), Rep     17.4(5), Dev..       3.33 Sigma
           C(21-C(26-C(35-C(41  1_555  1_555  1_555  1_555      #  105 Check
```

Author Response: The structre is affected by some strain. Crystal imperfections can not be excluded either.

```
PLAT703_ALERT_1_A Torsion Calc     45.0(2), Rep     47.0(5), Dev..      10.00 Sigma
           C(16-C(29-C(37-C(42  1_555  1_555  1_555  1_555      #  107 Check
```

Author Response: The structre is affected by some strain. Crystal imperfections can not be excluded either.

```
PLAT703_ALERT_1_A Torsion Calc     48.6(2), Rep     49.8(4), Dev..       6.00 Sigma
           C(25-C(31-C(42-C(37  1_555  1_555  1_555  1_555      #  108 Check
```

Author Response: The structre is affected by some strain. Crystal imperfections can not be excluded either.

```
PLAT703_ALERT_1_A Torsion Calc    -45.6(2), Rep    -48.6(5), Dev..      15.00 Sigma
           C(26-C(35-C(41-C(40  1_555  1_555  1_555  1_555      #  109 Check
```

Author Response: The structre is affected by some strain. Crystal imperfections can not be excluded either.

```
PLAT703_ALERT_1_A Torsion Calc    -64.4(2), Rep    -66.4(4), Dev..      10.00 Sigma
           C(29-C(37-C(42-C(31  1_555  1_555  1_555  1_555      #  110 Check
```

Author Response: The structre is affected by some strain. Crystal imperfections can not be excluded either.

```
PLAT703_ALERT_1_A Torsion Calc    -40.9(3), Rep    -43.7(5), Dev..       9.33 Sigma
           C(21-C(39-C(40-C(41  1_555  1_555  1_555  1_555      #  111 Check
```

Author Response: The structre is affected by some strain. Crystal imperfections can not be excluded either.

```
PLAT703_ALERT_1_A Torsion Calc     61.1(2), Rep     65.1(5), Dev..      20.00 Sigma
           C(39-C(40-C(41-C(35  1_555  1_555  1_555  1_555      #  112 Check
```

Author Response: The structre is affected by some strain. Crystal imperfections can not be excluded either.

Alert level B

```
REFLT02_ALERT_1_B  The number of reflections greater than the sigma threshold
            cannot exceed the number of symmetry-independent reflections
            Number of symmetry-independent reflections =   5330
            Number of reflections greater than sigma threshold =  24540
PLAT029_ALERT_3_B _diffrn_measured_fraction_theta_full value Low .      0.954 Why?
PLAT703_ALERT_1_B Torsion Calc  -62.71(15), Rep    -62.4(2), Dev..       2.07 Sigma
              O(3)-S(1)-N(8)-C(34  1_555  1_555  1_555  1_555      #    2 Check
```

Author Response: The structre is affected by some strain. Crystal imperfections can not be excluded either.

```
PLAT703_ALERT_1_B Torsion Calc  -67.57(14), Rep    -67.2(3), Dev..       2.64 Sigma
              O(5)-S(1)-N(8)-N(10  1_555  1_555  1_555  1_555      #    5 Check
```

Author Response: The structre is affected by some strain. Crystal imperfections can not be excluded either.

```
PLAT703_ALERT_1_B Torsion Calc  152.49(16), Rep    152.9(3), Dev..       2.56 Sigma
              N(8)-S(1)-C(18-C(13  1_555  1_555  1_555  1_555      #    9 Check
```

Author Response: The structre is affected by some strain. Crystal imperfections can not be excluded either.

```
PLAT703_ALERT_1_B Torsion Calc -163.15(13), Rep   -162.8(2), Dev..       2.69 Sigma
              O(1)-S(2)-N(9)-N(12  1_555  1_555  1_555  1_555      #   13 Check
```

Author Response: The structre is affected by some strain. Crystal imperfections can not be excluded either.

```
PLAT703_ALERT_1_B Torsion Calc   64.35(16), Rep     64.0(3), Dev..       2.19 Sigma
              O(1)-S(2)-N(9)-C(36  1_555  1_555  1_555  1_555      #   14 Check
```

Author Response: The structre is affected by some strain. Crystal imperfections can not be excluded either.

```
PLAT703_ALERT_1_B Torsion Calc  -41.76(19), Rep    -42.2(4), Dev..       2.32 Sigma
              O(1)-S(2)-C(15-C(22  1_555  1_555  1_555  1_555      #   15 Check
```

Author Response: The structre is affected by some strain. Crystal imperfections can not be excluded either.

```
PLAT703_ALERT_1_B Torsion Calc  -48.09(15), Rep    -48.4(3), Dev..       2.07 Sigma
              C(15-S(2)-N(9)-N(12  1_555  1_555  1_555  1_555      #   23 Check
```

Author Response: The structre is affected by some strain. Crystal imperfections can not be excluded either.

```
PLAT703_ALERT_1_B Torsion Calc -179.11(19), Rep   -178.6(3), Dev..       2.68 Sigma
              C(13-N(1)-C(21-C(39  1_555  1_555  1_555  1_555      #   26 Check
```

Author Response: The structre is affected by some strain. Crystal imperfections can not be excluded either.

```
PLAT703_ALERT_1_B Torsion Calc      0.4(2), Rep     -0.1(3), Dev..       2.50 Sigma
              C(21-N(1)-C(13-C(19  1_555  1_555  1_555  1_555      #   28 Check
```

Author Response: The structre is affected by some strain. Crystal imperfections can not be excluded either.

```
PLAT703_ALERT_1_B Torsion Calc   -179.5(2), Rep   -178.9(4), Dev..       3.00 Sigma
              C(16-N(11-C(22-C(15  1_555  1_555  1_555  1_555      #   35 Check
```

Author Response: The structre is affected by some strain. Crystal imperfections can not be excluded either.

```
PLAT703_ALERT_1_B Torsion Calc -179.24(19), Rep   -179.7(4), Dev..       2.42 Sigma
              N(1)-C(13-C(18-C(20  1_555  1_555  1_555  1_555      #   42 Check
```

Author Response: The structre is affected by some strain. Crystal imperfections can not be excluded either.

```
PLAT703_ALERT_1_B Torsion Calc     -1.2(3), Rep     -1.9(6), Dev..       2.33 Sigma
              C(19-C(14-C(32-C(20  1_555  1_555  1_555  1_555      #   49 Check
```

Author Response: The structre is affected by some strain. Crystal imperfections can not be excluded either.

```
PLAT703_ALERT_1_B Torsion Calc      4.3(3), Rep      3.6(6), Dev..       2.33 Sigma
              S(2)-C(15-C(22-N(11  1_555  1_555  1_555  1_555      #   52 Check
```

Author Response: The structre is affected by some strain. Crystal imperfections can not be excluded either.

```
PLAT703_ALERT_1_B Torsion Calc  173.07(16), Rep    172.6(3), Dev..       2.94 Sigma
              S(2)-C(15-C(24-C(30  1_555  1_555  1_555  1_555      #   55 Check
```

Author Response: The structre is affected by some strain. Crystal imperfections can not be excluded either.

```
PLAT703_ALERT_1_B Torsion Calc  179.50(19), Rep    179.1(3), Dev..       2.11 Sigma
             C(22-C(15-C(24-C(27  1_555  1_555  1_555  1_555      #   56 Check
```

Author Response: The structre is affected by some strain. Crystal imperfections can not be excluded either.

```
PLAT703_ALERT_1_B Torsion Calc     -0.8(3), Rep      0.1(4), Dev..       3.00 Sigma
             C(24-C(15-C(22-C(23  1_555  1_555  1_555  1_555      #   59 Check
```

Author Response: The structre is affected by some strain. Crystal imperfections can not be excluded either.

```
PLAT703_ALERT_1_B Torsion Calc -177.76(18), Rep   -178.3(3), Dev..       3.00 Sigma
             C(13-C(18-C(20-C(17  1_555  1_555  1_555  1_555      #   72 Check
```

Author Response: The structre is affected by some strain. Crystal imperfections can not be excluded either.

```
PLAT703_ALERT_1_B Torsion Calc      1.2(3), Rep      0.4(5), Dev..       2.67 Sigma
             C(13-C(18-C(20-C(32  1_555  1_555  1_555  1_555      #   73 Check
```

Author Response: The structre is affected by some strain. Crystal imperfections can not be excluded either.

```
PLAT703_ALERT_1_B Torsion Calc      0.3(2), Rep     -0.3(4), Dev..       3.00 Sigma
             C(13-C(19-C(26-C(21  1_555  1_555  1_555  1_555      #   74 Check
```

Author Response: The structre is affected by some strain. Crystal imperfections can not be excluded either.

```
PLAT703_ALERT_1_B Torsion Calc     11.0(3), Rep     11.7(6), Dev..       2.33 Sigma
             C(26-C(21-C(39-C(40  1_555  1_555  1_555  1_555      #   83 Check
```

Author Response: The structre is affected by some strain. Crystal imperfections can not be excluded either.

```
PLAT703_ALERT_1_B Torsion Calc -177.51(17), Rep   -177.0(3), Dev..       3.00 Sigma
             N(11-C(22-C(23-C(28  1_555  1_555  1_555  1_555      #   87 Check
```

Author Response: The structre is affected by some strain. Crystal imperfections can not be excluded either.

Alert level C

```
DIFMN03_ALERT_1_C  The minimum difference density is < -0.1*ZMAX*0.75
            The relevant atom site should be identified.
DIFMX02_ALERT_1_C  The maximum difference density is > 0.1*ZMAX*0.75
            The relevant atom site should be identified.
PLAT230_ALERT_2_C Hirshfeld Test Diff for    S2       --O6       .        5.7 s.u.
PLAT230_ALERT_2_C Hirshfeld Test Diff for    S2       --C15      .        6.4 s.u.
PLAT413_ALERT_2_C Short Inter XH3 .. XHn     H9       ..H20      .       2.11 Ang.
                                                      x,y,z  =      1_555 Check
PLAT703_ALERT_1_C Torsion Calc -146.90(15), Rep   -147.2(3), Dev..       2.00 Sigma
              O(3)-S(1)-C(18-C(20  1_555  1_555  1_555  1_555      #    4 Check
```

Author Response: The structre is affected by some strain. Crystal imperfections can not be excluded either.

```
PLAT703_ALERT_1_C Torsion Calc -172.71(17), Rep   -172.4(3), Dev..       1.82 Sigma
              C(34-N(8)-N(10-C(17  1_555  1_555  1_555  1_555      #   30 Check
```

Author Response: The structre is affected by some strain. Crystal imperfections can not be excluded either.

```
PLAT703_ALERT_1_C Torsion Calc      0.0(2), Rep     -0.3(4), Dev..       1.50 Sigma
              C(22-N(11-C(16-C(25  1_555  1_555  1_555  1_555      #   37 Check
```

Author Response: The structre is affected by some strain. Crystal imperfections can not be excluded either.

```
PLAT703_ALERT_1_C Torsion Calc  176.65(19), Rep    176.4(4), Dev..       1.32 Sigma
              C(22-N(11-C(16-C(29  1_555  1_555  1_555  1_555      #   38 Check
```

Author Response: The structre is affected by some strain. Crystal imperfections can not be excluded either.

```
PLAT703_ALERT_1_C Torsion Calc  170.04(15), Rep    169.8(3), Dev..       1.60 Sigma
              C(19-C(13-C(18-S(1)  1_555  1_555  1_555  1_555      #   47 Check
```

Author Response: The structre is affected by some strain. Crystal imperfections can not be excluded either.

```
PLAT703_ALERT_1_C Torsion Calc     -6.9(3), Rep     -6.5(5), Dev..       1.33 Sigma
              S(2)-C(15-C(24-C(27  1_555  1_555  1_555  1_555      #   54 Check
```

Author Response: The structre is affected by some strain. Crystal imperfections can not be excluded either.

```
PLAT703_ALERT_1_C Torsion Calc      0.9(2), Rep      1.2(4), Dev..       1.50 Sigma
              N(11-C(16-C(25-C(23  1_555  1_555  1_555  1_555      #   60 Check
```

Author Response: The structre is affected by some strain. Crystal imperfections can not be excluded either.

```
PLAT703_ALERT_1_C Torsion Calc   -175.7(2), Rep   -175.4(4), Dev..       1.50 Sigma
              C(29-C(16-C(25-C(23  1_555  1_555  1_555  1_555      #   64 Check
```

Author Response: The structre is affected by some strain. Crystal imperfections can not be excluded either.

```
PLAT703_ALERT_1_C Torsion Calc -170.85(18), Rep   -170.5(3), Dev..       1.94 Sigma
              C(33-C(17-C(20-C(18  1_555  1_555  1_555  1_555      #   68 Check
```

Author Response: The structre is affected by some strain. Crystal imperfections can not be excluded either.

```
PLAT703_ALERT_1_C Torsion Calc     10.3(3), Rep     10.8(6), Dev..       1.67 Sigma
              C(33-C(17-C(20-C(32  1_555  1_555  1_555  1_555      #   69 Check
```

Author Response: The structre is affected by some strain. Crystal imperfections can not be excluded either.

```
PLAT703_ALERT_1_C Torsion Calc    179.9(2), Rep   -179.8(4), Dev..       1.50 Sigma
              C(14-C(19-C(26-C(21  1_555  1_555  1_555  1_555      #   76 Check
```

Author Response: The structre is affected by some strain. Crystal imperfections can not be excluded either.

```
PLAT703_ALERT_1_C Torsion Calc  179.94(19), Rep   -179.8(4), Dev..       1.37 Sigma
              C(17-C(20-C(32-C(14  1_555  1_555  1_555  1_555      #   78 Check
```

Author Response: The structre is affected by some strain. Crystal imperfections can not be excluded either.

```
PLAT703_ALERT_1_C Torsion Calc      1.0(3), Rep      1.6(6), Dev..       2.00 Sigma
              C(18-C(20-C(32-C(14  1_555  1_555  1_555  1_555      #   79 Check
```

Author Response: The structre is affected by some strain. Crystal imperfections can not be excluded either.

```
PLAT703_ALERT_1_C Torsion Calc     -0.1(2), Rep      0.2(4), Dev..       1.50 Sigma
              N(1)-C(21-C(26-C(19  1_555  1_555  1_555  1_555      #   80 Check
```

Author Response: The structre is affected by some strain. Crystal imperfections can not be excluded either.

```
PLAT703_ALERT_1_C Torsion Calc -170.25(19), Rep   -169.9(4), Dev..       1.84 Sigma
              N(1)-C(21-C(39-C(40  1_555  1_555  1_555  1_555      #   82 Check
```

Author Response: The structre is affected by some strain. Crystal imperfections can not be excluded either.

```
PLAT703_ALERT_1_C Torsion Calc -179.74(18), Rep    179.9(3), Dev..       2.00 Sigma
              C(15-C(22-C(23-C(25  1_555  1_555  1_555  1_555      #   88 Check
```

Author Response: The structre is affected by some strain. Crystal imperfections can not be excluded either.

```
PLAT703_ALERT_1_C Torsion Calc     -1.4(2), Rep     -1.7(4), Dev..       1.50 Sigma
              C(22-C(23-C(25-C(16  1_555  1_555  1_555  1_555      #   90 Check
```

Author Response: The structre is affected by some strain. Crystal imperfections can not be excluded either.

```
PLAT703_ALERT_1_C Torsion Calc     -0.6(3), Rep     -1.0(6), Dev..       1.33 Sigma
              C(22-C(23-C(28-C(30  1_555  1_555  1_555  1_555      #   92 Check
```

Author Response: The structre is affected by some strain. Crystal imperfections can not be excluded either.

```
PLAT703_ALERT_1_C Torsion Calc    -12.9(3), Rep    -13.5(6), Dev..       2.00 Sigma
              C(15-C(24-C(27-N(12  1_555  1_555  1_555  1_555      #   96 Check
```

Author Response: The structre is affected by some strain. Crystal imperfections can not be excluded either.

```
PLAT703_ALERT_1_C Torsion Calc    159.7(2), Rep    159.4(4), Dev..       1.50 Sigma
              C(23-C(25-C(31-C(42  1_555  1_555  1_555  1_555      #  103 Check
```

Author Response: The structre is affected by some strain. Crystal imperfections can not be excluded either.

Alert level G

```
PLAT005_ALERT_5_G No Embedded Refinement Details Found  in the CIF     Please Do !
PLAT007_ALERT_5_G Number of Unrefined Donor-H Atoms .............          2 Report
              H32   H1
PLAT063_ALERT_4_G Crystal Size Possibly too Large for Beam Size ..       0.64 mm
```

```
PLAT199_ALERT_1_G Reported _cell_measurement_temperature ..... (K)        293 Check
PLAT200_ALERT_1_G Reported   _diffrn_ambient_temperature ..... (K)        293 Check
PLAT808_ALERT_5_G No Parseable SHELXL Style Weighting Scheme Found     Please Check
PLAT882_ALERT_1_G No Datum for _diffrn_reflns_av_unetI/netI ......     Please Do !
PLAT883_ALERT_1_G Absent Datum for _atom_sites_solution_primary ..     Please Do !
```

```
  44 ALERT level A = Most likely a serious problem - resolve or explain
  23 ALERT level B = A potentially serious problem, consider carefully
  25 ALERT level C = Check. Ensure it is not caused by an omission or oversight
   8 ALERT level G = General information/check it is not something unexpected

  89 ALERT type 1 CIF construction/syntax error, inconsistent or missing data
   6 ALERT type 2 Indicator that the structure model may be wrong or deficient
   1 ALERT type 3 Indicator that the structure quality may be low
   1 ALERT type 4 Improvement, methodology, query or suggestion
   3 ALERT type 5 Informative message, check
```

It is advisable to attempt to resolve as many as possible of the alerts in all categories. Often the minor alerts point to easily fixed oversights, errors and omissions in your CIF or refinement strategy, so attention to these fine details can be worthwhile. In order to resolve some of the more serious problems it may be necessary to carry out additional measurements or structure refinements. However, the purpose of your study may justify the reported deviations and the more serious of these should normally be commented upon in the discussion or experimental section of a paper or in the "special_details" fields of the CIF. checkCIF was carefully designed to identify outliers and unusual parameters, but every test has its limitations and alerts that are not important in a particular case may appear. Conversely, the absence of alerts does not guarantee there are no aspects of the results needing attention. It is up to the individual to critically assess their own results and, if necessary, seek expert advice.

Publication of your CIF in IUCr journals

A basic structural check has been run on your CIF. These basic checks will be run on all CIFs submitted for publication in IUCr journals (*Acta Crystallographica*, *Journal of Applied Crystallography*, *Journal of Synchrotron Radiation*); however, if you intend to submit to *Acta Crystallographica Section C* or *E* or *IUCrData*, you should make sure that full publication checks are run on the final version of your CIF prior to submission.

Publication of your CIF in other journals

Please refer to the *Notes for Authors* of the relevant journal for any special instructions relating to CIF submission.

Validation response form

Please find below a validation response form (VRF) that can be filled in and pasted into your CIF.

```
# start Validation Reply Form
_vrf_REFLT02__142501_1
;
PROBLEM: The number of reflections greater than the sigma threshold
RESPONSE: ...
;
_vrf_DIFMN03__142501_1
;
PROBLEM: The minimum difference density is < -0.1*ZMAX*0.75
RESPONSE: ...
;
_vrf_DIFMX02__142501_1
;
PROBLEM: The maximum difference density is > 0.1*ZMAX*0.75
RESPONSE: ...
;
_vrf_PLAT029__142501_1
;
PROBLEM: _diffrn_measured_fraction_theta_full value Low .      0.954 Why?
RESPONSE: ...
;
_vrf_PLAT230__142501_1
;
PROBLEM: Hirshfeld Test Diff for    S2       --O6       .        5.7 s.u.
RESPONSE: ...
;
_vrf_PLAT413__142501_1
;
PROBLEM: Short Inter XH3 .. XHn     H9       ..H20      .       2.11 Ang.
RESPONSE: ...
;
# end Validation Reply Form
```

PLATON version of 02/02/2025; check.def file version of 02/02/2025

Datablock _142501_1 - ellipsoid plot

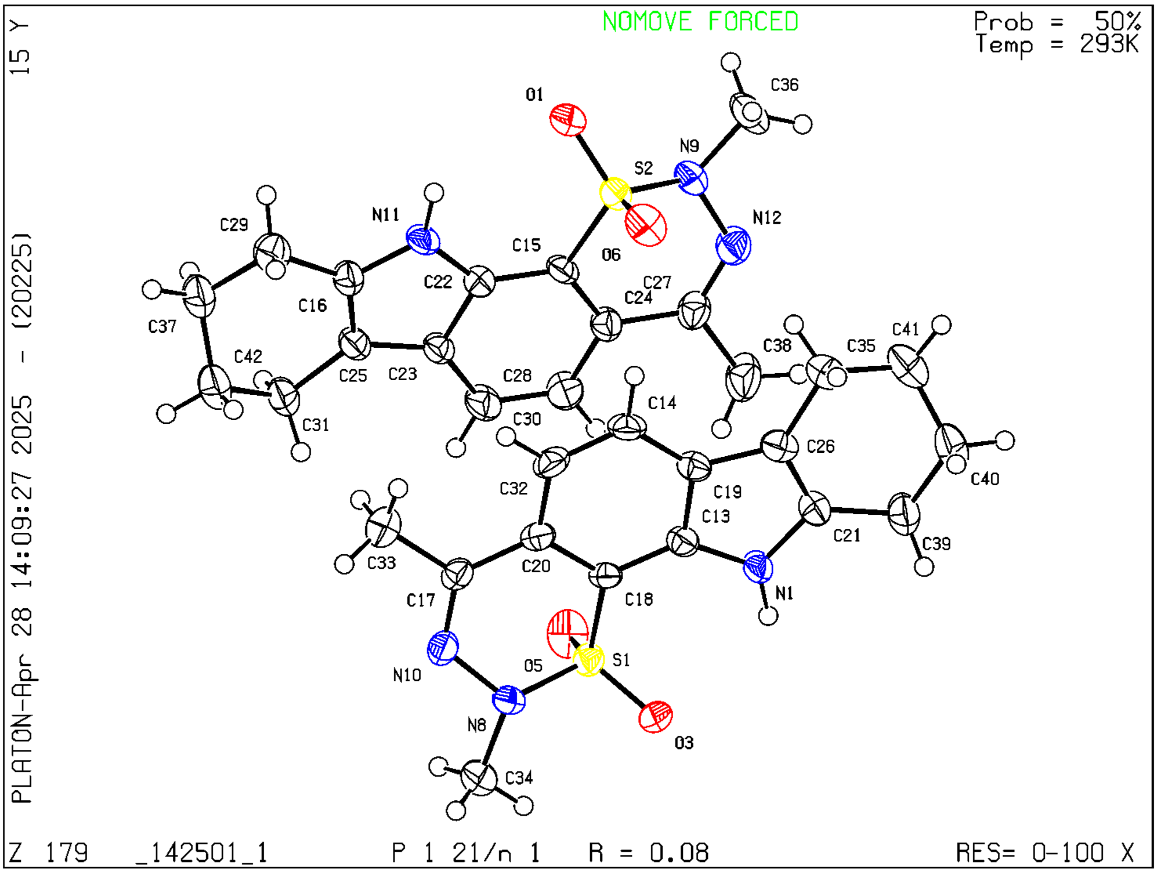

Supplement: File 2 — Crystallographic information files, checkcif and structure report files for compounds 3b, 3d, 3e, 3g, 3h, (E)-7a, 7b, 7d, 7e, (E)-7f, (Z)-7h, 7i and (E)-9a. [file 3e_checkcif.pdf › Átnevezett XRD/3e_checkcif.pdf]
