# Supplementary material for: Thiadiazino-indole, thiadiazino-carbazole and benzothiadiazino-carbazole dioxides: synthesis, physicochemical and early ADME characterization of representatives of new tri-, tetra- and pentacyclic ring systems and their intermediates
Source: Beilstein J Org Chem. 2025 Oct 21;21:2220–33. doi: 10.3762/bjoc.21.169 (PMC12557438; doi:10.3762/bjoc.21.169)

checkCIF/PLATON report

You have not supplied any structure factors. As a result the full set of tests cannot be run.

THIS REPORT IS FOR GUIDANCE ONLY. IF USED AS PART OF A REVIEW PROCEDURE FOR PUBLICATION, IT SHOULD NOT REPLACE THE EXPERTISE OF AN EXPERIENCED CRYSTALLOGRAPHIC REFEREE.

No syntax errors found. CIF dictionary Interpreting this report

Datablock: _142698_1

Bond precision: C-C = 0.0150 A Wavelength=1.54187

Cell:	a=8.5535(15)	b=12.697(3)	c=13.488(3)
	alpha=89.323(11)	beta=89.449(9)	gamma=86.785(11)
Temperature:	293 K		

	Calculated	Reported
Volume	1462.4(5)	1462.3(5)
Space group	P -1	P -1
Hall group	-P 1	-P 1
Moiety formula	C13 H18 N4 O2 S, C12 H15 N4 O2 S, C H3	C13 H18 N4 O2 S
Sum formula	C26 H36 N8 O4 S2	C13 H18 N4 O2 S
Mr	588.75	294.37
Dx,g cm-3	1.337	1.337
Z	2	4
Mu (mm-1)	2.038	2.038
F000	624.0	624.0
F000'	627.04	
h,k,lmax	10,15,16	10,15,16
Nref	5732	5193
Tmin,Tmax	0.660,0.832	0.622,0.836
Tmin'	0.496	

Correction method= # Reported T Limits: Tmin=0.622 Tmax=0.836
AbsCorr = MULTI-SCAN

Data completeness= 0.906 Theta(max)= 71.620

R(reflections)= 0.0634(6795) wR2(reflections)= wR= 0.0767(6866)

S = 0.937 Npar= 397

The following ALERTS were generated. Each ALERT has the format
test-name_ALERT_alert-type_alert-level.
Click on the hyperlinks for more details of the test.

Alert level A

DIFMN02_ALERT_2_A The minimum difference density is < -0.1*ZMAX*2.00
_refine_diff_density_min given = -6.190
Test value = -3.200

Author Response: Altough the residual density is out of border but there is no doubt about the chemical structure taking into account other evidences, e.g. NMR. The error maybe due to crystal imperfections.

PLAT029_ALERT_3_A _diffrn_measured_fraction_theta_full value Low . 0.906 Why?

Author Response: Altough some large-theta reflexions are too weak but there is no doubt about the chemical structure taking into account other evidences, e.g. NMR. The error maybe due to crystal imperfections.

PLAT098_ALERT_2_A Large Reported Min. (Negative) Residual Density -6.19 eA-3

Author Response: Altough the residual density is out of border but there is no doubt about the chemical structure taking into account other evidences, e.g. NMR. The error maybe due to crystal imperfections.

PLAT211_ALERT_2_A ADP of Atom C31 is N.P.D. or (nearly) 2D . Please Check

Author Response: The structure is affected by some strain. Crystal imperfections can not be excluded either.

Alert level B

REFLT02_ALERT_1_B The number of reflections greater than the sigma threshold
cannot exceed the number of symmetry-independent reflections
Number of symmetry-independent reflections = 5193
Number of reflections greater than sigma threshold = 6795
PLAT097_ALERT_2_B Large Reported Max. (Positive) Residual Density 3.09 eA-3
PLAT110_ALERT_2_B ADDSYM Detects Potential Lattice Translation ... ? Check
PLAT112_ALERT_2_B ADDSYM Detects New (Pseudo) Symm. Elem B 100 %Fit
PLAT113_ALERT_2_B ADDSYM Suggests Possible Pseudo/New Space Group P-1 Check
Check Model Parameter Symmetry for Reflection Data Support
PLAT213_ALERT_2_B Atom O4 has ADP max/min Ratio ..... 4.6 prolat
PLAT230_ALERT_2_B Hirshfeld Test Diff for S2 --O3 . 23.3 s.u.
PLAT230_ALERT_2_B Hirshfeld Test Diff for S2 --C1 . 9.0 s.u.
PLAT230_ALERT_2_B Hirshfeld Test Diff for N11 --C24 . 8.9 s.u.
PLAT230_ALERT_2_B Hirshfeld Test Diff for N14 --C26 . 9.8 s.u.
PLAT230_ALERT_2_B Hirshfeld Test Diff for C26 --C27 . 8.2 s.u.

```
PLAT230_ALERT_2_B Hirshfeld Test Diff for    C29      --C31      .       11.5 s.u.
PLAT230_ALERT_2_B Hirshfeld Test Diff for    S1       --O6       .        8.3 s.u.
PLAT230_ALERT_2_B Hirshfeld Test Diff for    N1       --C36      .        9.4 s.u.
PLAT230_ALERT_2_B Hirshfeld Test Diff for    N10      --C19      .        8.0 s.u.
PLAT230_ALERT_2_B Hirshfeld Test Diff for    N13      --C22      .        9.8 s.u.
PLAT230_ALERT_2_B Hirshfeld Test Diff for    C17      --C19      .        7.8 s.u.
PLAT230_ALERT_2_B Hirshfeld Test Diff for    C22      --C33      .        7.4 s.u.
PLAT242_ALERT_2_B Low    'MainMol' Ueq as Compared to Neighbors of        C36 Check
PLAT340_ALERT_3_B Low Bond Precision on  C-C Bonds ..............      0.015 Ang.
PLAT374_ALERT_2_B Long   N - N         Bond  N1       - N13      .       1.61 Ang.
```

Alert level C

```
DIFMN03_ALERT_1_C  The minimum difference density is < -0.1*ZMAX*0.75
            The relevant atom site should be identified.
DIFMX02_ALERT_1_C  The maximum difference density is > 0.1*ZMAX*0.75
            The relevant atom site should be identified.
PLAT041_ALERT_1_C Calc. and Reported SumFormula    Strings  Differ     Please Check
              Calc: C26 H36 N8 O4 S2
              Rep.: C13 H18 N4 O2 S
PLAT042_ALERT_1_C Calc. and Reported MoietyFormula Strings  Differ     Please Check
              Calc: C13 H18 N4 O2 S, C12 H15 N4 O2 S, C H3
              Rep.: C13 H18 N4 O2 S
PLAT213_ALERT_2_C Atom C30             has ADP max/min Ratio .....        3.3 oblate
PLAT214_ALERT_2_C Atom C19       (Anion/Solvent) ADP max/min Ratio        4.4 oblate
PLAT220_ALERT_2_C NonSolvent   Resd 1  C    Ueq(max)/Ueq(min) Range        4.3 Ratio
PLAT230_ALERT_2_C Hirshfeld Test Diff for    S2       --O4       .        6.0 s.u.
PLAT230_ALERT_2_C Hirshfeld Test Diff for    N7       --C31      .        6.5 s.u.
PLAT230_ALERT_2_C Hirshfeld Test Diff for    N11      --N14      .        6.0 s.u.
PLAT230_ALERT_2_C Hirshfeld Test Diff for    C18      --C23      .        5.6 s.u.
PLAT230_ALERT_2_C Hirshfeld Test Diff for    C18      --C31      .        5.3 s.u.
PLAT230_ALERT_2_C Hirshfeld Test Diff for    C24      --C30      .        5.3 s.u.
PLAT230_ALERT_2_C Hirshfeld Test Diff for    C21      --C33      .        5.2 s.u.
PLAT234_ALERT_4_C Large Hirshfeld Difference C23      --C28      .       0.17 Ang.
PLAT234_ALERT_4_C Large Hirshfeld Difference C24      --C40      .       0.18 Ang.
PLAT234_ALERT_4_C Large Hirshfeld Difference C27      --C28      .       0.16 Ang.
PLAT241_ALERT_2_C High   'MainMol' Ueq as Compared to Neighbors of        N14 Check
PLAT241_ALERT_2_C High   'MainMol' Ueq as Compared to Neighbors of        C24 Check
PLAT241_ALERT_2_C High   'MainMol' Ueq as Compared to Neighbors of         N1 Check
PLAT242_ALERT_2_C Low    'MainMol' Ueq as Compared to Neighbors of        N11 Check
PLAT242_ALERT_2_C Low    'MainMol' Ueq as Compared to Neighbors of        C26 Check
PLAT242_ALERT_2_C Low    'MainMol' Ueq as Compared to Neighbors of        C31 Check
PLAT242_ALERT_2_C Low    'MainMol' Ueq as Compared to Neighbors of        C40 Check
PLAT362_ALERT_2_C Short  C(sp3)-C(sp2) Bond  C29      - C31      .       1.32 Ang.
PLAT369_ALERT_2_C Long   C(sp2)-C(sp2) Bond  C1       - C26      .       1.56 Ang.
PLAT369_ALERT_2_C Long   C(sp2)-C(sp2) Bond  C21      - C25      .       1.54 Ang.
PLAT369_ALERT_2_C Long   C(sp2)-C(sp2) Bond  C22      - C33      .       1.55 Ang.
PLAT412_ALERT_2_C Short Intra XH3 .. XHn     H4       ..H17      .       1.89 Ang.
                                                      x,y,z  =      1_555 Check
PLAT414_ALERT_2_C Short Intra D-H..H-X       H1       ..H33      .       1.95 Ang.
                                                      x,y,z  =      1_555 Check
```

Alert level G

```
PLAT005_ALERT_5_G No Embedded Refinement Details Found  in the CIF     Please Do !
PLAT007_ALERT_5_G Number of Unrefined Donor-H Atoms .............          2 Report
              H2    H1
```

```
PLAT045_ALERT_1_G Calculated and Reported Z Differ by a Factor ...      0.500 Check
PLAT116_ALERT_2_G ADDSYM Included (Pseudo) Lattice Translation ...     Please Check
PLAT199_ALERT_1_G Reported _cell_measurement_temperature ..... (K)        293 Check
PLAT200_ALERT_1_G Reported   _diffrn_ambient_temperature ..... (K)        293 Check
PLAT335_ALERT_2_G Check Large C6 Ring C-C Range C1       -C26            0.32 Ang.
PLAT335_ALERT_2_G Check Large C6 Ring C-C Range C15      -C22            0.32 Ang.
PLAT343_ALERT_2_G Unusual sp?      Angle Range in Main Residue for        C19 Check
PLAT344_ALERT_2_G Unusual sp?       Angle Range in Solvent/Ion for        C32 Check
PLAT432_ALERT_2_G Short Inter X...Y Contact  N10      ..C32      .       2.51 Ang.
                                                      x,y,z  =     1_555 Check
PLAT432_ALERT_2_G Short Inter X...Y Contact  C17      ..C32      .       2.78 Ang.
                                                      x,y,z  =     1_555 Check
PLAT432_ALERT_2_G Short Inter X...Y Contact  C19      ..C32      .       1.76 Ang.
                                                      x,y,z  =     1_555 Check
PLAT432_ALERT_2_G Short Inter X...Y Contact  C25      ..C32      .       2.91 Ang.
                                                      x,y,z  =     1_555 Check
PLAT773_ALERT_2_G Check long C-C Bond in CIF: C19      --C32             1.76 Ang.
PLAT790_ALERT_4_G Centre of Gravity not Within Unit Cell: Resd.  #          2 Note
              C12 H15 N4 O2 S
PLAT790_ALERT_4_G Centre of Gravity not Within Unit Cell: Resd.  #          3 Note
              C H3
PLAT808_ALERT_5_G No Parseable SHELXL Style Weighting Scheme Found     Please Check
PLAT882_ALERT_1_G No Datum for _diffrn_reflns_av_unetI/netI ......     Please Do !
PLAT883_ALERT_1_G Absent Datum for _atom_sites_solution_primary ..     Please Do !
```

---

 4 **ALERT level A** = Most likely a serious problem - resolve or explain
21 **ALERT level B** = A potentially serious problem, consider carefully
30 **ALERT level C** = Check. Ensure it is not caused by an omission or oversight
20 **ALERT level G** = General information/check it is not something unexpected

10 ALERT type 1 CIF construction/syntax error, inconsistent or missing data
55 ALERT type 2 Indicator that the structure model may be wrong or deficient
 2 ALERT type 3 Indicator that the structure quality may be low
 5 ALERT type 4 Improvement, methodology, query or suggestion
 3 ALERT type 5 Informative message, check

It is advisable to attempt to resolve as many as possible of the alerts in all categories. Often the minor alerts point to easily fixed oversights, errors and omissions in your CIF or refinement strategy, so attention to these fine details can be worthwhile. In order to resolve some of the more serious problems it may be necessary to carry out additional measurements or structure refinements. However, the purpose of your study may justify the reported deviations and the more serious of these should normally be commented upon in the discussion or experimental section of a paper or in the "special_details" fields of the CIF. checkCIF was carefully designed to identify outliers and unusual parameters, but every test has its limitations and alerts that are not important in a particular case may appear. Conversely, the absence of alerts does not guarantee there are no aspects of the results needing attention. It is up to the individual to critically assess their own results and, if necessary, seek expert advice.

Publication of your CIF in IUCr journals

A basic structural check has been run on your CIF. These basic checks will be run on all CIFs submitted for publication in IUCr journals (*Acta Crystallographica*, *Journal of Applied Crystallography*, *Journal of Synchrotron Radiation*); however, if you intend to submit to *Acta Crystallographica Section C* or *E* or *IUCrData*, you should make sure that full publication checks are run on the final version of your CIF prior to submission.

Publication of your CIF in other journals

Please refer to the *Notes for Authors* of the relevant journal for any special instructions relating to CIF submission.

Validation response form

Please find below a validation response form (VRF) that can be filled in and pasted into your CIF.

```
# start Validation Reply Form
_vrf_REFLT02__142698_1
;
PROBLEM: The number of reflections greater than the sigma threshold
RESPONSE: ...
;
_vrf_DIFMN03__142698_1
;
PROBLEM: The minimum difference density is < -0.1*ZMAX*0.75
RESPONSE: ...
;
_vrf_DIFMX02__142698_1
;
PROBLEM: The maximum difference density is > 0.1*ZMAX*0.75
RESPONSE: ...
;
_vrf_PLAT097__142698_1
;
PROBLEM: Large Reported Max.  (Positive) Residual Density       3.09 eA-3
RESPONSE: ...
;
_vrf_PLAT110__142698_1
```

```
;
PROBLEM: ADDSYM Detects Potential Lattice Translation ...          ? Check
RESPONSE: ...
;
_vrf_PLAT112__142698_1
;
PROBLEM: ADDSYM Detects New (Pseudo) Symm. Elem         B        100 %Fit
RESPONSE: ...
;
_vrf_PLAT113__142698_1
;
PROBLEM: ADDSYM Suggests Possible Pseudo/New  Space Group        P-1 Check
RESPONSE: ...
;
_vrf_PLAT213__142698_1
;
PROBLEM: Atom O4              has ADP max/min Ratio .....        4.6 prolat
RESPONSE: ...
;
_vrf_PLAT230__142698_1
;
PROBLEM: Hirshfeld Test Diff for    S2       --O3       .       23.3 s.u.
RESPONSE: ...
;
_vrf_PLAT242__142698_1
;
PROBLEM: Low    'MainMol' Ueq as Compared to Neighbors of        C36 Check
RESPONSE: ...
;
_vrf_PLAT340__142698_1
;
PROBLEM: Low Bond Precision on  C-C Bonds ..............      0.015 Ang.
RESPONSE: ...
;
_vrf_PLAT374__142698_1
;
PROBLEM: Long   N - N         Bond  N1       - N13      .       1.61 Ang.
RESPONSE: ...
;
_vrf_PLAT041__142698_1
;
PROBLEM: Calc. and Reported SumFormula    Strings  Differ     Please Check
RESPONSE: ...
;
_vrf_PLAT042__142698_1
;
PROBLEM: Calc. and Reported MoietyFormula Strings  Differ     Please Check
RESPONSE: ...
;
_vrf_PLAT214__142698_1
;
PROBLEM: Atom C19       (Anion/Solvent) ADP max/min Ratio        4.4 oblate
RESPONSE: ...
;
_vrf_PLAT220__142698_1
;
PROBLEM: NonSolvent   Resd 1  C   Ueq(max)/Ueq(min) Range        4.3 Ratio
```

```
RESPONSE: ...
;
_vrf_PLAT234__142698_1
;
PROBLEM: Large Hirshfeld Difference C23      --C28      .       0.17 Ang.
RESPONSE: ...
;
_vrf_PLAT241__142698_1
;
PROBLEM: High   'MainMol' Ueq as Compared to Neighbors of        N14 Check
RESPONSE: ...
;
_vrf_PLAT362__142698_1
;
PROBLEM: Short  C(sp3)-C(sp2) Bond  C29      - C31      .       1.32 Ang.
RESPONSE: ...
;
_vrf_PLAT369__142698_1
;
PROBLEM: Long   C(sp2)-C(sp2) Bond  C1       - C26      .       1.56 Ang.
RESPONSE: ...
;
_vrf_PLAT412__142698_1
;
PROBLEM: Short Intra XH3 .. XHn     H4       ..H17      .       1.89 Ang.
RESPONSE: ...
;
_vrf_PLAT414__142698_1
;
PROBLEM: Short Intra D-H..H-X       H1       ..H33      .       1.95 Ang.
RESPONSE: ...
;
# end Validation Reply Form
```

PLATON version of 02/02/2025; check.def file version of 02/02/2025

Datablock _142698_1 - ellipsoid plot

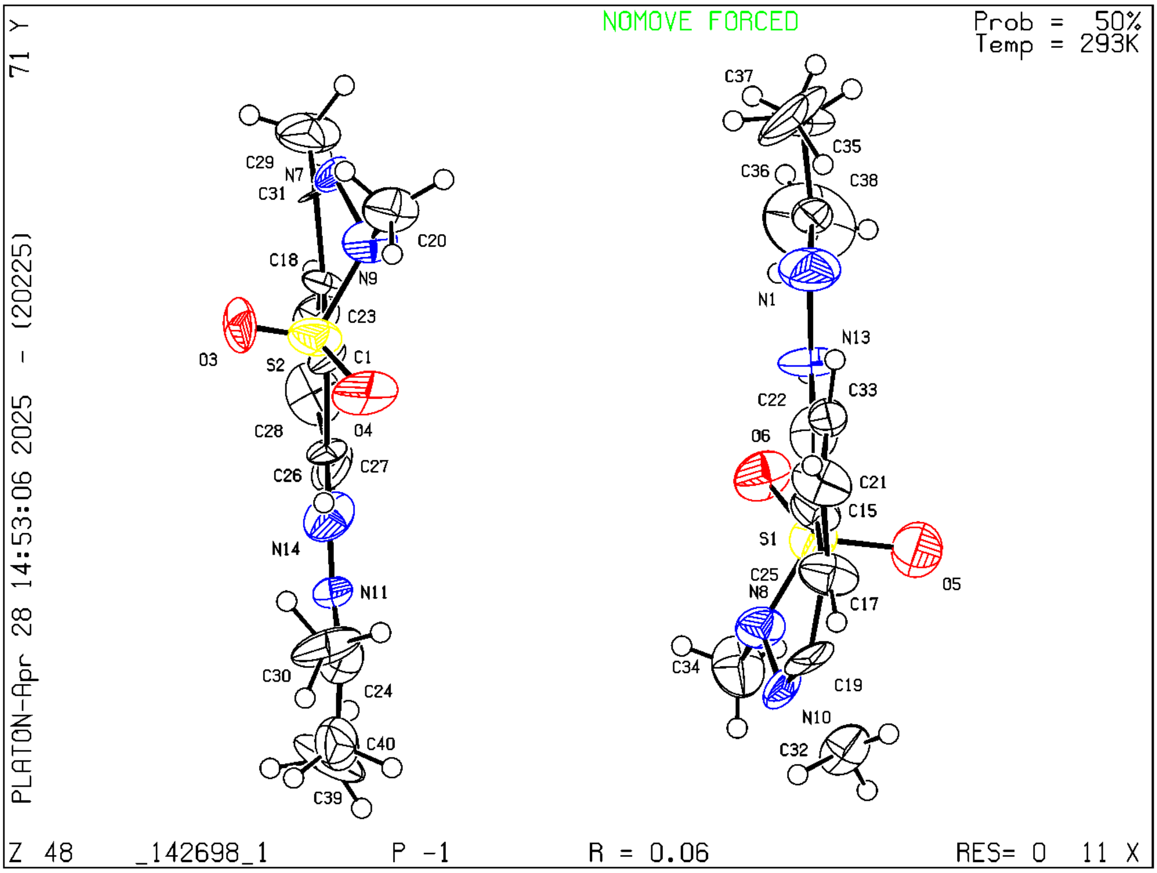

Supplement: File 2 — Crystallographic information files, checkcif and structure report files for compounds 3b, 3d, 3e, 3g, 3h, (E)-7a, 7b, 7d, 7e, (E)-7f, (Z)-7h, 7i and (E)-9a. [file 7a_checkcif.pdf › Átnevezett XRD/7a_checkcif.pdf]
