# Supplementary material for: Thiadiazino-indole, thiadiazino-carbazole and benzothiadiazino-carbazole dioxides: synthesis, physicochemical and early ADME characterization of representatives of new tri-, tetra- and pentacyclic ring systems and their intermediates
Source: Beilstein J Org Chem. 2025 Oct 21;21:2220–33. doi: 10.3762/bjoc.21.169 (PMC12557438; doi:10.3762/bjoc.21.169)

checkCIF/PLATON report

You have not supplied any structure factors. As a result the full set of tests cannot be run.

THIS REPORT IS FOR GUIDANCE ONLY. IF USED AS PART OF A REVIEW PROCEDURE FOR PUBLICATION, IT SHOULD NOT REPLACE THE EXPERTISE OF AN EXPERIENCED CRYSTALLOGRAPHIC REFEREE.

No syntax errors found.   CIF dictionary   Interpreting this report

Datablock: _143768_2

```
Bond precision:   C-C = 0.0043 A          Wavelength=1.54187

Cell:              a=7.1655(3)      b=11.9214(5)      c=19.5707(9)
                   alpha=90         beta=90           gamma=90
Temperature:       293 K
```

	Calculated	Reported
Volume	1671.78(13)	1671.78(13)
Space group	P n a 21	P n a 21
Hall group	P 2c -2n	P 2c -2n
Moiety formula	C17 H18 N4 O2 S	C17 H18 N4 O2 S
Sum formula	C17 H18 N4 O2 S	C17 H18 N4 O2 S
Mr	342.41	342.41
Dx,g cm-3	1.360	1.360
Z	4	4
Mu (mm-1)	1.869	1.869
F000	720.0	720.0
F000'	723.31	
h,k,lmax	8,14,24	8,14,21
Nref	3261[1681]	2751
Tmin,Tmax	0.767,0.845	0.604,0.853
Tmin'	0.459	

```
Correction method= # Reported T Limits: Tmin=0.604 Tmax=0.853
AbsCorr = MULTI-SCAN

Data completeness= 1.64/0.84        Theta(max)= 71.580

R(reflections)= 0.0550( 13295)      wR2(reflections)= 0.0814( 13381)

S = 1.119                    Npar= 262
```

The following ALERTS were generated. Each ALERT has the format
test-name_ALERT_alert-type_alert-level.
Click on the hyperlinks for more details of the test.

Alert level A

```
DIFMN02_ALERT_2_A  The minimum difference density is < -0.1*ZMAX*2.00
            _refine_diff_density_min given =      -4.970
            Test value =      -3.200
```

Author Response: Altough the residual density is out of border but there is no doubt about the chemical structure taking into account other evidences, e.g. NMR. The error maybe due to crystal imperfections.

```
PLAT097_ALERT_2_A Large Reported Max.  (Positive) Residual Density       7.02 eA-3
```

Author Response: Altough the residual density is out of border but there is no doubt about the chemical structure taking into account other evidences, e.g. NMR. The error maybe due to crystal imperfections.

```
PLAT098_ALERT_2_A Large Reported Min.  (Negative) Residual Density      -4.97 eA-3
```

Author Response: Altough the residual density is out of border but there is no doubt about the chemical structure taking into account other evidences, e.g. NMR. The error maybe due to crystal imperfections.

Alert level B

```
REFLT02_ALERT_1_B  The number of reflections greater than the sigma threshold
            cannot exceed the number of symmetry-independent reflections
            Number of symmetry-independent reflections =    2751
            Number of reflections greater than sigma threshold =  13295
PLAT230_ALERT_2_B Hirshfeld Test Diff for    S1       --N4       .         9.6 s.u.
PLAT230_ALERT_2_B Hirshfeld Test Diff for    S1       --C1       .         7.1 s.u.
PLAT230_ALERT_2_B Hirshfeld Test Diff for    C10      --C13      .         7.5 s.u.
PLAT230_ALERT_2_B Hirshfeld Test Diff for    C13      --C22      .         7.7 s.u.
PLAT230_ALERT_2_B Hirshfeld Test Diff for    C14      --C19      .        14.0 s.u.
PLAT230_ALERT_2_B Hirshfeld Test Diff for    C18      --C23      .         8.2 s.u.
PLAT230_ALERT_2_B Hirshfeld Test Diff for    C20      --C22      .         9.2 s.u.
PLAT245_ALERT_2_B U(iso) H3        Smaller than U(eq) C23       by      0.080 Ang**2
PLAT245_ALERT_2_B U(iso) H9        Smaller than U(eq) N6        by      0.060 Ang**2
PLAT703_ALERT_1_B Torsion Calc    174.4(2), Rep    173.9(3), Dev..       2.50 Sigma
              C(10-C(13-C(15-C(18  1_555  1_555  1_555  1_555      #   42 Check
```

Alert level C

```
DIFMN03_ALERT_1_C  The minimum difference density is < -0.1*ZMAX*0.75
            The relevant atom site should be identified.
DIFMX02_ALERT_1_C  The maximum difference density is > 0.1*ZMAX*0.75
```

```
           The relevant atom site should be identified.
PLAT029_ALERT_3_C _diffrn_measured_fraction_theta_full value Low .      0.967 Why?
PLAT034_ALERT_1_C No Flack Parameter Given. Z > Si, NonCentro ....     Please Do !
PLAT089_ALERT_3_C Poor Data / Parameter Ratio (Zmax < 18) ........       6.42 Note
PLAT166_ALERT_4_C S.U's Given on Coordinates for Calc-flagged ....         H1 Note
PLAT166_ALERT_4_C S.U's Given on Coordinates for Calc-flagged ....         H2 Note
PLAT166_ALERT_4_C S.U's Given on Coordinates for Calc-flagged ....         H3 Note
PLAT166_ALERT_4_C S.U's Given on Coordinates for Calc-flagged ....         H4 Note
PLAT166_ALERT_4_C S.U's Given on Coordinates for Calc-flagged ....         H5 Note
PLAT166_ALERT_4_C S.U's Given on Coordinates for Calc-flagged ....         H6 Note
PLAT166_ALERT_4_C S.U's Given on Coordinates for Calc-flagged ....         H7 Note
PLAT166_ALERT_4_C S.U's Given on Coordinates for Calc-flagged ....         H8 Note
PLAT166_ALERT_4_C S.U's Given on Coordinates for Calc-flagged ....         H9 Note
PLAT222_ALERT_3_C NonSolvent Resd 1  H   Uiso(max)/Uiso(min) Range       10.0 Ratio
PLAT230_ALERT_2_C Hirshfeld Test Diff for    S1       --O2       .        5.5 s.u.
PLAT230_ALERT_2_C Hirshfeld Test Diff for    C16      --C21      .        5.7 s.u.
PLAT245_ALERT_2_C U(iso) H2        Smaller than U(eq) C15       by      0.011 Ang**2
PLAT245_ALERT_2_C U(iso) H5        Smaller than U(eq) C22       by      0.040 Ang**2
PLAT245_ALERT_2_C U(iso) H6        Smaller than U(eq) C12       by      0.016 Ang**2
PLAT340_ALERT_3_C Low Bond Precision on  C-C Bonds ..............    0.00425 Ang.
PLAT703_ALERT_1_C Torsion Calc -174.69(15), Rep -174.43(18), Dev..       1.73 Sigma
              O(2)-S(1)-N(4)-N(5)  1_555  1_555  1_555  1_555      #    1 Check
PLAT703_ALERT_1_C Torsion Calc  152.41(13), Rep  152.26(19), Dev..       1.15 Sigma
              O(2)-S(1)-C(1)-C(9)  1_555  1_555  1_555  1_555      #    4 Check
PLAT703_ALERT_1_C Torsion Calc  -81.82(19), Rep    -82.1(2), Dev..       1.47 Sigma
              O(3)-S(1)-N(4)-C(17  1_555  1_555  1_555  1_555      #    6 Check
PLAT703_ALERT_1_C Torsion Calc   -167.0(2), Rep   -167.3(3), Dev..       1.50 Sigma
              C(16-N(1)-N(6)-C(8)  1_555  1_555  1_555  1_555      #   14 Check
PLAT703_ALERT_1_C Torsion Calc     42.2(2), Rep     41.9(3), Dev..       1.50 Sigma
              S(1)-N(4)-N(5)-C(10  1_555  1_555  1_555  1_555      #   15 Check
PLAT703_ALERT_1_C Torsion Calc      1.8(3), Rep      2.2(4), Dev..       1.33 Sigma
              N(4)-N(5)-C(10-C(9)  1_555  1_555  1_555  1_555      #   17 Check
PLAT703_ALERT_1_C Torsion Calc  178.03(16), Rep    178.2(2), Dev..       1.06 Sigma
              N(4)-N(5)-C(10-C(13  1_555  1_555  1_555  1_555      #   18 Check
PLAT703_ALERT_1_C Torsion Calc  179.08(18), Rep    179.3(2), Dev..       1.22 Sigma
              C(9)-C(1)-C(8)-N(6)  1_555  1_555  1_555  1_555      #   27 Check
PLAT703_ALERT_1_C Torsion Calc   -175.5(2), Rep   -175.2(3), Dev..       1.50 Sigma
              C(10-C(13-C(22-C(20  1_555  1_555  1_555  1_555      #   43 Check
PLAT703_ALERT_1_C Torsion Calc     -3.7(4), Rep     -4.2(5), Dev..       1.25 Sigma
              C(22-C(13-C(15-C(18  1_555  1_555  1_555  1_555      #   45 Check
```

Alert level G

```
PLAT005_ALERT_5_G No Embedded Refinement Details Found  in the CIF     Please Do !
PLAT199_ALERT_1_G Reported _cell_measurement_temperature ..... (K)        293 Check
PLAT200_ALERT_1_G Reported   _diffrn_ambient_temperature ..... (K)        293 Check
PLAT808_ALERT_5_G No Parseable SHELXL Style Weighting Scheme Found     Please Check
PLAT882_ALERT_1_G No Datum for _diffrn_reflns_av_unetI/netI ......     Please Do !
PLAT883_ALERT_1_G Absent Datum for _atom_sites_solution_primary ..     Please Do !
PLAT952_ALERT_5_G Calculated (ThMax) and CIF-Reported Lmax Differ.          3 Units
```

---

3 **ALERT level A** = Most likely a serious problem - resolve or explain
11 **ALERT level B** = A potentially serious problem, consider carefully
31 **ALERT level C** = Check. Ensure it is not caused by an omission or oversight
7 **ALERT level G** = General information/check it is not something unexpected

```
 19 ALERT type 1 CIF construction/syntax error, inconsistent or missing data
 17 ALERT type 2 Indicator that the structure model may be wrong or deficient
  4 ALERT type 3 Indicator that the structure quality may be low
  9 ALERT type 4 Improvement, methodology, query or suggestion
  3 ALERT type 5 Informative message, check
```

It is advisable to attempt to resolve as many as possible of the alerts in all categories. Often the minor alerts point to easily fixed oversights, errors and omissions in your CIF or refinement strategy, so attention to these fine details can be worthwhile. In order to resolve some of the more serious problems it may be necessary to carry out additional measurements or structure refinements. However, the purpose of your study may justify the reported deviations and the more serious of these should normally be commented upon in the discussion or experimental section of a paper or in the "special_details" fields of the CIF. checkCIF was carefully designed to identify outliers and unusual parameters, but every test has its limitations and alerts that are not important in a particular case may appear. Conversely, the absence of alerts does not guarantee there are no aspects of the results needing attention. It is up to the individual to critically assess their own results and, if necessary, seek expert advice.

Publication of your CIF in IUCr journals

A basic structural check has been run on your CIF. These basic checks will be run on all CIFs submitted for publication in IUCr journals (*Acta Crystallographica*, *Journal of Applied Crystallography*, *Journal of Synchrotron Radiation*); however, if you intend to submit to *Acta Crystallographica Section C* or *E* or *IUCrData*, you should make sure that full publication checks are run on the final version of your CIF prior to submission.

Publication of your CIF in other journals

Please refer to the *Notes for Authors* of the relevant journal for any special instructions relating to CIF submission.

Validation response form

Please find below a validation response form (VRF) that can be filled in and pasted into your CIF.

```
# start Validation Reply Form
_vrf_REFLT02__143768_2
;
PROBLEM: The number of reflections greater than the sigma threshold
RESPONSE: ...
;
_vrf_DIFMN03__143768_2
;
PROBLEM: The minimum difference density is < -0.1*ZMAX*0.75
RESPONSE: ...
;
_vrf_DIFMX02__143768_2
;
PROBLEM: The maximum difference density is > 0.1*ZMAX*0.75
RESPONSE: ...
```

```
;
_vrf_PLAT230__143768_2
;
PROBLEM: Hirshfeld Test Diff for    S1       --N4       .        9.6 s.u.
RESPONSE: ...
;
_vrf_PLAT245__143768_2
;
PROBLEM: U(iso) H3        Smaller than U(eq) C23       by      0.080 Ang**2
RESPONSE: ...
;
_vrf_PLAT703__143768_2
;
PROBLEM: Torsion Calc    174.4(2), Rep    173.9(3), Dev..       2.50 Sigma
RESPONSE: ...
;
_vrf_PLAT029__143768_2
;
PROBLEM: _diffrn_measured_fraction_theta_full value Low .      0.967 Why?
RESPONSE: ...
;
_vrf_PLAT034__143768_2
;
PROBLEM: No Flack Parameter Given. Z > Si, NonCentro ....     Please Do !
RESPONSE: ...
;
_vrf_PLAT089__143768_2
;
PROBLEM: Poor Data / Parameter Ratio (Zmax < 18) ........       6.42 Note
RESPONSE: ...
;
_vrf_PLAT166__143768_2
;
PROBLEM: S.U's Given on Coordinates for Calc-flagged ....         H1 Note
RESPONSE: ...
;
_vrf_PLAT222__143768_2
;
PROBLEM: NonSolvent Resd 1  H   Uiso(max)/Uiso(min) Range       10.0 Ratio
RESPONSE: ...
;
_vrf_PLAT340__143768_2
;
PROBLEM: Low Bond Precision on  C-C Bonds ...............    0.00425 Ang.
RESPONSE: ...
;
# end Validation Reply Form
```

PLATON version of 02/02/2025; check.def file version of 02/02/2025

Datablock _143768_2 - ellipsoid plot

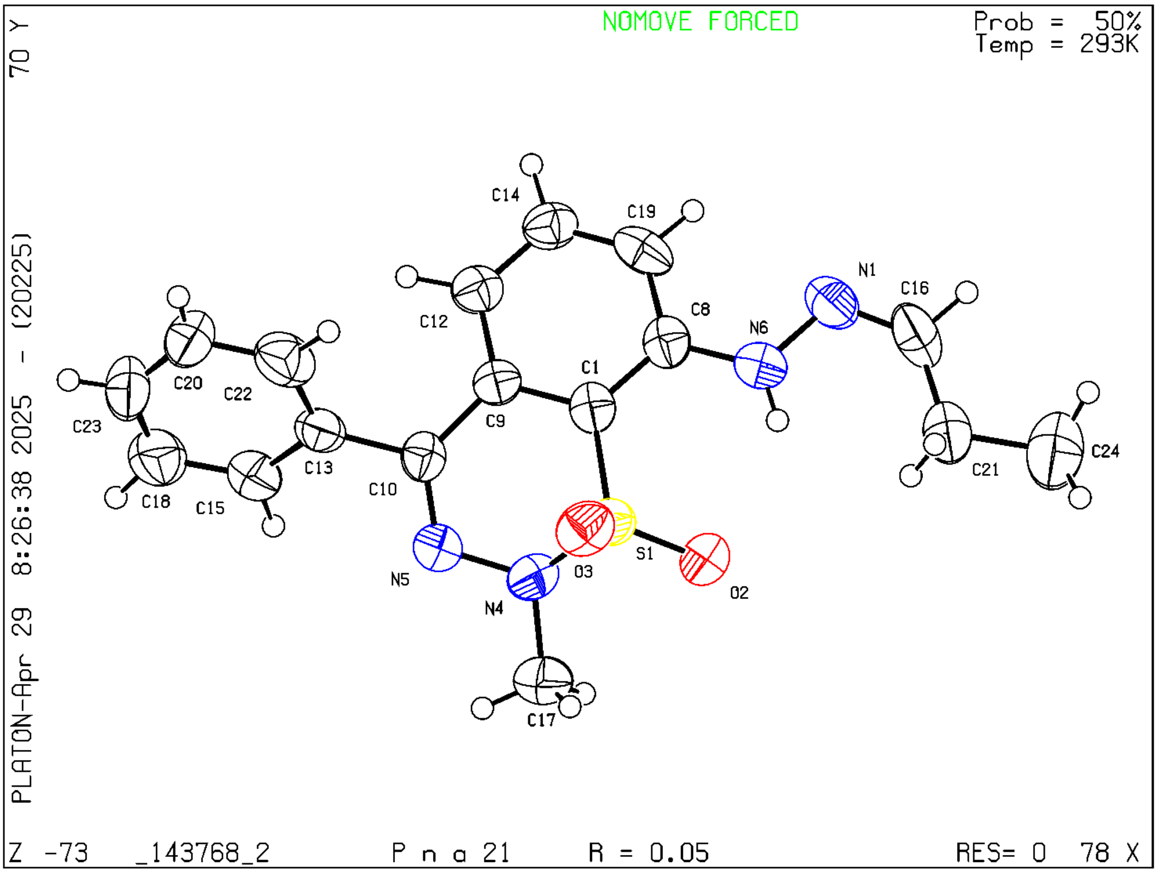

Supplement: File 2 — Crystallographic information files, checkcif and structure report files for compounds 3b, 3d, 3e, 3g, 3h, (E)-7a, 7b, 7d, 7e, (E)-7f, (Z)-7h, 7i and (E)-9a. [file 7h_checkcif.pdf › Átnevezett XRD/7h_checkcif.pdf]
