# Supplementary material for: Thiadiazino-indole, thiadiazino-carbazole and benzothiadiazino-carbazole dioxides: synthesis, physicochemical and early ADME characterization of representatives of new tri-, tetra- and pentacyclic ring systems and their intermediates
Source: Beilstein J Org Chem. 2025 Oct 21;21:2220–33. doi: 10.3762/bjoc.21.169 (PMC12557438; doi:10.3762/bjoc.21.169)

checkCIF/PLATON report

You have not supplied any structure factors. As a result the full set of tests cannot be run.

THIS REPORT IS FOR GUIDANCE ONLY. IF USED AS PART OF A REVIEW PROCEDURE FOR PUBLICATION, IT SHOULD NOT REPLACE THE EXPERTISE OF AN EXPERIENCED CRYSTALLOGRAPHIC REFEREE.

No syntax errors found. CIF dictionary Interpreting this report

Datablock: _143675_1

```
Bond precision:   C-C = 0.0028 A          Wavelength=1.54187

Cell:              a=10.0402(4)      b=10.9583(4)      c=16.5846(8)
                   alpha=90          beta=90           gamma=90
Temperature:       293 K

                Calculated                 Reported
Volume          1824.70(13)                1824.70(13)
Space group     P 21 21 21                 P 21 21 21
Hall group      P 2ac 2ab                  P 2ac 2ab
Moiety formula  C19 H20 N4 O2 S            C19 H20 N4 O2 S
Sum formula     C19 H20 N4 O2 S            C19 H20 N4 O2 S
Mr              368.45                     368.45
Dx,g cm-3       1.341                      1.341
Z               4                          4
Mu (mm-1)       1.752                      1.752
F000            776.0                      776.0
F000'           779.45
h,k,lmax        12,13,20                   12,13,20
Nref            3575[ 2049]                3511
Tmin,Tmax       0.690,0.782                0.537,0.776
Tmin'           0.300

Correction method= # Reported T Limits: Tmin=0.537 Tmax=0.776
AbsCorr = MULTI-SCAN

Data completeness= 1.71/0.98       Theta(max)= 71.590

                                                     wR2(reflections)=
R(reflections)= 0.0479( 17276)                       0.0745( 17378)

S = 1.043                 Npar= 294
```

The following ALERTS were generated. Each ALERT has the format
test-name_ALERT_alert-type_alert-level.
Click on the hyperlinks for more details of the test.

Alert level A

```
DIFMN02_ALERT_2_A  The minimum difference density is < -0.1*ZMAX*2.00
            _refine_diff_density_min given =     -5.090
            Test value =     -3.200
```

Author Response: Altough the residual density is out of border but there is no doubt about the chemical structure taking into account other evidences, e.g. NMR. The error maybe due to crystal imperfections.

```
PLAT097_ALERT_2_A Large Reported Max.  (Positive) Residual Density       5.04 eA-3
```

Author Response: Altough the residual density is out of border but there is no doubt about the chemical structure taking into account other evidences, e.g. NMR. The error maybe due to crystal imperfections.

```
PLAT098_ALERT_2_A Large Reported Min.  (Negative) Residual Density      -5.09 eA-3
```

Author Response: Altough the residual density is out of border but there is no doubt about the chemical structure taking into account other evidences, e.g. NMR. The error maybe due to crystal imperfections.

```
PLAT703_ALERT_1_A Torsion Calc   79.71(15), Rep     79.0(2), Dev..       4.73 Sigma
              O(2)-S(1)-N(5)-C(22  1_555  1_555  1_555  1_555      #    2 Check
```

Author Response: The structure is affected by some strain. Crystal imperfections can not be excluded either.

```
PLAT703_ALERT_1_A Torsion Calc  -50.05(16), Rep    -51.0(2), Dev..       5.94 Sigma
              O(3)-S(1)-N(5)-C(22  1_555  1_555  1_555  1_555      #    6 Check
```

Author Response: The structure is affected by some strain. Crystal imperfections can not be excluded either.

```
PLAT703_ALERT_1_A Torsion Calc -165.30(15), Rep -166.05(19), Dev..       5.00 Sigma
              C(15-S(1)-N(5)-C(22  1_555  1_555  1_555  1_555      #   12 Check
```

Author Response: The structure is affected by some strain. Crystal imperfections can not be excluded either.

```
PLAT703_ALERT_1_A Torsion Calc -143.33(19), Rep   -144.0(2), Dev..       3.53 Sigma
              C(13-C(8)-C(14-C(10  1_555  1_555  1_555  1_555      #   23 Check
```

Author Response: The structure is affected by some strain. Crystal imperfections can not be excluded either.

```
PLAT703_ALERT_1_A Torsion Calc   -173.1(2), Rep   -173.8(2), Dev..       3.50 Sigma
              C(14-C(8)-C(13-C(18  1_555  1_555  1_555  1_555      #   24 Check
```

Author Response: The structure is affected by some strain. Crystal imperfections can not be excluded either.

```
PLAT703_ALERT_1_A Torsion Calc      0.9(3), Rep     -0.3(4), Dev..       4.00 Sigma
              C(21-C(8)-C(13-C(18  1_555  1_555  1_555  1_555      #   26 Check
```

Author Response: The structure is affected by some strain. Crystal imperfections can not be excluded either.

```
PLAT703_ALERT_1_A Torsion Calc    174.0(2), Rep    173.3(3), Dev..       3.50 Sigma
              N(6)-C(12-C(23-C(25  1_555  1_555  1_555  1_555      #   49 Check
```

Author Response: The structure is affected by some strain. Crystal imperfections can not be excluded either.

```
PLAT703_ALERT_1_A Torsion Calc     -6.8(2), Rep     -8.1(3), Dev..       6.50 Sigma
              C(23-C(12-C(17-C(26  1_555  1_555  1_555  1_555      #   51 Check
```

Author Response: The structure is affected by some strain. Crystal imperfections can not be excluded either.

```
PLAT703_ALERT_1_A Torsion Calc     16.2(2), Rep     17.8(3), Dev..       8.00 Sigma
              C(12-C(17-C(26-C(25  1_555  1_555  1_555  1_555      #   53 Check
```

Author Response: The structure is affected by some strain. Crystal imperfections can not be excluded either.

```
PLAT703_ALERT_1_A Torsion Calc    -19.7(3), Rep    -21.1(3), Dev..       4.67 Sigma
              C(23-C(25-C(26-C(17  1_555  1_555  1_555  1_555      #   58 Check
```

Author Response: The structure is affected by some strain. Crystal imperfections can not be excluded either.

Alert level B

```
REFLT02_ALERT_1_B  The number of reflections greater than the sigma threshold
            cannot exceed the number of symmetry-independent reflections
            Number of symmetry-independent reflections =   3511
            Number of reflections greater than sigma threshold =  17276
PLAT230_ALERT_2_B Hirshfeld Test Diff for    C11      --C16      .        8.0 s.u.
PLAT230_ALERT_2_B Hirshfeld Test Diff for    C17      --C26      .        8.0 s.u.
PLAT230_ALERT_2_B Hirshfeld Test Diff for    C25      --C26      .       11.3 s.u.
PLAT241_ALERT_2_B High   'MainMol' Ueq as Compared to Neighbors of        C25 Check
PLAT703_ALERT_1_B Torsion Calc  178.33(10), Rep  178.08(13), Dev..       2.50 Sigma
              O(3)-S(1)-N(5)-N(4)  1_555  1_555  1_555  1_555      #    5 Check
```

Author Response: The structure is affected by some strain. Crystal imperfections can not be excluded either.

```
PLAT703_ALERT_1_B Torsion Calc  176.69(14), Rep  176.30(18), Dev..       2.79 Sigma
              N(5)-N(4)-C(14-C(8)  1_555  1_555  1_555  1_555      #   13 Check
```

Author Response: The structure is affected by some strain. Crystal imperfections can not be excluded either.

```
PLAT703_ALERT_1_B Torsion Calc     -1.0(3), Rep     -0.2(4), Dev..       2.67 Sigma
              N(7)-N(6)-C(12-C(23  1_555  1_555  1_555  1_555      #   18 Check
```

Author Response: The structure is affected by some strain. Crystal imperfections can not be excluded either.

```
PLAT703_ALERT_1_B Torsion Calc     -1.1(3), Rep     -0.4(4), Dev..       2.33 Sigma
              C(13-C(8)-C(21-C(24  1_555  1_555  1_555  1_555      #   25 Check
```

Author Response: The structure is affected by some strain. Crystal imperfections can not be excluded either.

```
PLAT703_ALERT_1_B Torsion Calc -135.46(19), Rep   -135.0(2), Dev..       2.42 Sigma
              C(21-C(8)-C(14-N(4)  1_555  1_555  1_555  1_555      #   28 Check
```

Author Response: The structure is affected by some strain. Crystal imperfections can not be excluded either.

```
PLAT703_ALERT_1_B Torsion Calc -155.04(17), Rep   -154.6(2), Dev..       2.59 Sigma
              C(15-C(10-C(14-C(8)  1_555  1_555  1_555  1_555      #   39 Check
```

Author Response: The structure is affected by some strain. Crystal imperfections can not be excluded either.

```
PLAT703_ALERT_1_B Torsion Calc  177.25(17), Rep    176.9(2), Dev..       2.06 Sigma
              C(14-C(10-C(20-C(11  1_555  1_555  1_555  1_555      #   40 Check
```

Author Response: The structure is affected by some strain. Crystal imperfections can not be excluded either.

```
PLAT703_ALERT_1_B Torsion Calc  174.89(19), Rep    174.4(2), Dev..       2.58 Sigma
              N(6)-C(12-C(17-C(26  1_555  1_555  1_555  1_555      #   48 Check
```

Author Response: The structure is affected by some strain. Crystal imperfections can not be excluded either.

```
PLAT703_ALERT_1_B Torsion Calc      0.0(4), Rep      1.2(4), Dev..       3.00 Sigma
              C(8)-C(13-C(18-C(19  1_555  1_555  1_555  1_555      #   52 Check
```

Author Response: The structure is affected by some strain. Crystal imperfections can not be excluded either.

Alert level C

```
DIFMN03_ALERT_1_C  The minimum difference density is < -0.1*ZMAX*0.75
            The relevant atom site should be identified.
DIFMX02_ALERT_1_C  The maximum difference density is > 0.1*ZMAX*0.75
            The relevant atom site should be identified.
PLAT034_ALERT_1_C No Flack Parameter Given. Z > Si, NonCentro ....     Please Do !
PLAT089_ALERT_3_C Poor Data / Parameter Ratio (Zmax < 18) ........       6.97 Note
PLAT166_ALERT_4_C S.U's Given on Coordinates for Calc-flagged ....         H1 Note
PLAT166_ALERT_4_C S.U's Given on Coordinates for Calc-flagged ....         H2 Note
PLAT166_ALERT_4_C S.U's Given on Coordinates for Calc-flagged ....         H3 Note
PLAT166_ALERT_4_C S.U's Given on Coordinates for Calc-flagged ....         H4 Note
PLAT166_ALERT_4_C S.U's Given on Coordinates for Calc-flagged ....         H5 Note
PLAT166_ALERT_4_C S.U's Given on Coordinates for Calc-flagged ....         H6 Note
PLAT166_ALERT_4_C S.U's Given on Coordinates for Calc-flagged ....         H7 Note
PLAT166_ALERT_4_C S.U's Given on Coordinates for Calc-flagged ....         H8 Note
PLAT166_ALERT_4_C S.U's Given on Coordinates for Calc-flagged ....         H9 Note
PLAT166_ALERT_4_C S.U's Given on Coordinates for Calc-flagged ....        H10 Note
PLAT166_ALERT_4_C S.U's Given on Coordinates for Calc-flagged ....        H11 Note
PLAT166_ALERT_4_C S.U's Given on Coordinates for Calc-flagged ....        H12 Note
PLAT166_ALERT_4_C S.U's Given on Coordinates for Calc-flagged ....        H13 Note
PLAT222_ALERT_3_C NonSolvent Resd 1  H   Uiso(max)/Uiso(min) Range        6.3 Ratio
PLAT230_ALERT_2_C Hirshfeld Test Diff for    S1       --O2       .        6.0 s.u.
PLAT230_ALERT_2_C Hirshfeld Test Diff for    S1       --N5       .        5.7 s.u.
PLAT230_ALERT_2_C Hirshfeld Test Diff for    S1       --C15      .        6.0 s.u.
PLAT230_ALERT_2_C Hirshfeld Test Diff for    C8       --C13      .        5.7 s.u.
PLAT230_ALERT_2_C Hirshfeld Test Diff for    C8       --C14      .        6.4 s.u.
PLAT242_ALERT_2_C Low    'MainMol' Ueq as Compared to Neighbors of        C23 Check
PLAT245_ALERT_2_C U(iso) H3        Smaller than U(eq) C20       by      0.019 Ang**2
PLAT245_ALERT_2_C U(iso) H4        Smaller than U(eq) C13       by      0.030 Ang**2
PLAT245_ALERT_2_C U(iso) H5        Smaller than U(eq) C18       by      0.029 Ang**2
PLAT245_ALERT_2_C U(iso) H9        Smaller than U(eq) C22       by      0.021 Ang**2
PLAT245_ALERT_2_C U(iso) H11       Smaller than U(eq) N7        by      0.039 Ang**2
```

```
PLAT245_ALERT_2_C U(iso) H12       Smaller than U(eq) C23       by      0.015 Ang**2
PLAT351_ALERT_3_C Long   C-H (X0.96,N1.08A)  C23      - H13      .       1.13 Ang.
PLAT360_ALERT_2_C Short  C(sp3)-C(sp3) Bond  C25      - C26      .       1.42 Ang.
PLAT703_ALERT_1_C Torsion Calc  142.27(15), Rep  142.00(19), Dev..       1.80 Sigma
             N(5)-S(1)-C(15-C(9)  1_555  1_555  1_555  1_555      #    9 Check
```

Author Response: The structure is affected by some strain. Crystal imperfections can not be excluded either.

```
PLAT703_ALERT_1_C Torsion Calc  -40.25(14), Rep  -40.42(18), Dev..       1.21 Sigma
             N(5)-S(1)-C(15-C(10  1_555  1_555  1_555  1_555      #   10 Check
```

Author Response: The structure is affected by some strain. Crystal imperfections can not be excluded either.

```
PLAT703_ALERT_1_C Torsion Calc     -1.4(2), Rep     -1.1(3), Dev..       1.50 Sigma
             N(5)-N(4)-C(14-C(10  1_555  1_555  1_555  1_555      #   14 Check
```

Author Response: The structure is affected by some strain. Crystal imperfections can not be excluded either.

```
PLAT703_ALERT_1_C Torsion Calc -179.74(17), Rep   -179.5(2), Dev..       1.41 Sigma
             C(14-N(4)-N(5)-C(22  1_555  1_555  1_555  1_555      #   16 Check
```

Author Response: The structure is affected by some strain. Crystal imperfections can not be excluded either.

```
PLAT703_ALERT_1_C Torsion Calc  176.88(17), Rep    176.7(2), Dev..       1.06 Sigma
             N(7)-N(6)-C(12-C(17  1_555  1_555  1_555  1_555      #   17 Check
```

Author Response: The structure is affected by some strain. Crystal imperfections can not be excluded either.

```
PLAT703_ALERT_1_C Torsion Calc  174.17(18), Rep    174.5(2), Dev..       1.83 Sigma
             C(12-N(6)-N(7)-C(9)  1_555  1_555  1_555  1_555      #   19 Check
```

Author Response: The structure is affected by some strain. Crystal imperfections can not be excluded either.

```
PLAT703_ALERT_1_C Torsion Calc -177.65(16), Rep   -177.4(2), Dev..       1.56 Sigma
             C(14-C(10-C(15-C(9)  1_555  1_555  1_555  1_555      #   37 Check
```

Author Response: The structure is affected by some strain. Crystal imperfections can not be excluded either.

```
PLAT703_ALERT_1_C Torsion Calc -153.79(18), Rep   -154.0(2), Dev..       1.17 Sigma
              C(20-C(10-C(14-N(4)  1_555  1_555  1_555  1_555      #   41 Check
```

Author Response: The structure is affected by some strain. Crystal imperfections can not be excluded either.

```
PLAT703_ALERT_1_C Torsion Calc     28.2(3), Rep     28.7(3), Dev..       1.67 Sigma
              C(20-C(10-C(14-C(8)  1_555  1_555  1_555  1_555      #   42 Check
```

Author Response: The structure is affected by some strain. Crystal imperfections can not be excluded either.

```
PLAT703_ALERT_1_C Torsion Calc     -0.7(4), Rep     -1.4(5), Dev..       1.75 Sigma
              C(13-C(18-C(19-C(24  1_555  1_555  1_555  1_555      #   54 Check
```

Author Response: The structure is affected by some strain. Crystal imperfections can not be excluded either.

```
PLAT703_ALERT_1_C Torsion Calc     14.4(3), Rep     15.0(3), Dev..       2.00 Sigma
              C(12-C(23-C(25-C(26  1_555  1_555  1_555  1_555      #   57 Check
```

Author Response: The structure is affected by some strain. Crystal imperfections can not be excluded either.

Alert level G

```
PLAT005_ALERT_5_G No Embedded Refinement Details Found  in the CIF     Please Do !
PLAT063_ALERT_4_G Crystal Size Possibly too Large for Beam Size ..       0.66 mm
PLAT199_ALERT_1_G Reported _cell_measurement_temperature ..... (K)        293 Check
PLAT200_ALERT_1_G Reported   _diffrn_ambient_temperature ..... (K)        293 Check
PLAT808_ALERT_5_G No Parseable SHELXL Style Weighting Scheme Found     Please Check
PLAT882_ALERT_1_G No Datum for _diffrn_reflns_av_unetI/netI ......     Please Do !
PLAT883_ALERT_1_G Absent Datum for _atom_sites_solution_primary ..     Please Do !
```

```
 13 ALERT level A = Most likely a serious problem - resolve or explain
 14 ALERT level B = A potentially serious problem, consider carefully
 43 ALERT level C = Check. Ensure it is not caused by an omission or oversight
  7 ALERT level G = General information/check it is not something unexpected

 38 ALERT type 1 CIF construction/syntax error, inconsistent or missing data
 20 ALERT type 2 Indicator that the structure model may be wrong or deficient
  3 ALERT type 3 Indicator that the structure quality may be low
 14 ALERT type 4 Improvement, methodology, query or suggestion
  2 ALERT type 5 Informative message, check
```

It is advisable to attempt to resolve as many as possible of the alerts in all categories. Often the minor alerts point to easily fixed oversights, errors and omissions in your CIF or refinement strategy, so attention to these fine details can be worthwhile. In order to resolve some of the more serious problems it may be necessary to carry out additional measurements or structure refinements. However, the purpose of your study may justify the reported deviations and the more serious of these should normally be commented upon in the discussion or experimental section of a paper or in the "special_details" fields of the CIF. checkCIF was carefully designed to identify outliers and unusual parameters, but every test has its limitations and alerts that are not important in a particular case may appear. Conversely, the absence of alerts does not guarantee there are no aspects of the results needing attention. It is up to the individual to critically assess their own results and, if necessary, seek expert advice.

Publication of your CIF in IUCr journals

A basic structural check has been run on your CIF. These basic checks will be run on all CIFs submitted for publication in IUCr journals (*Acta Crystallographica*, *Journal of Applied Crystallography*, *Journal of Synchrotron Radiation*); however, if you intend to submit to *Acta Crystallographica Section C* or *E* or *IUCrData*, you should make sure that full publication checks are run on the final version of your CIF prior to submission.

Publication of your CIF in other journals

Please refer to the *Notes for Authors* of the relevant journal for any special instructions relating to CIF submission.

Validation response form

Please find below a validation response form (VRF) that can be filled in and pasted into your CIF.

```
# start Validation Reply Form
_vrf_REFLT02__143675_1
;
PROBLEM: The number of reflections greater than the sigma threshold
RESPONSE: ...
;
_vrf_DIFMN03__143675_1
;
PROBLEM: The minimum difference density is < -0.1*ZMAX*0.75
RESPONSE: ...
;
_vrf_DIFMX02__143675_1
;
PROBLEM: The maximum difference density is > 0.1*ZMAX*0.75
RESPONSE: ...
;
_vrf_PLAT230__143675_1
;
PROBLEM: Hirshfeld Test Diff for    C11      --C16      .        8.0 s.u.
RESPONSE: ...
;
_vrf_PLAT241__143675_1
```

```
;
PROBLEM: High   'MainMol' Ueq as Compared to Neighbors of        C25 Check
RESPONSE: ...
;
_vrf_PLAT034__143675_1
;
PROBLEM: No Flack Parameter Given. Z > Si, NonCentro ....     Please Do !
RESPONSE: ...
;
_vrf_PLAT089__143675_1
;
PROBLEM: Poor Data / Parameter Ratio (Zmax < 18) ........       6.97 Note
RESPONSE: ...
;
_vrf_PLAT166__143675_1
;
PROBLEM: S.U's Given on Coordinates for Calc-flagged ....         H1 Note
RESPONSE: ...
;
_vrf_PLAT222__143675_1
;
PROBLEM: NonSolvent Resd 1  H   Uiso(max)/Uiso(min) Range        6.3 Ratio
RESPONSE: ...
;
_vrf_PLAT242__143675_1
;
PROBLEM: Low    'MainMol' Ueq as Compared to Neighbors of        C23 Check
RESPONSE: ...
;
_vrf_PLAT245__143675_1
;
PROBLEM: U(iso) H3        Smaller than U(eq) C20       by      0.019 Ang**2
RESPONSE: ...
;
_vrf_PLAT351__143675_1
;
PROBLEM: Long   C-H (X0.96,N1.08A)  C23      - H13      .       1.13 Ang.
RESPONSE: ...
;
_vrf_PLAT360__143675_1
;
PROBLEM: Short  C(sp3)-C(sp3) Bond  C25      - C26      .       1.42 Ang.
RESPONSE: ...
;
# end Validation Reply Form
```

PLATON version of 02/02/2025; check.def file version of 02/02/2025

Datablock _143675_1 - ellipsoid plot

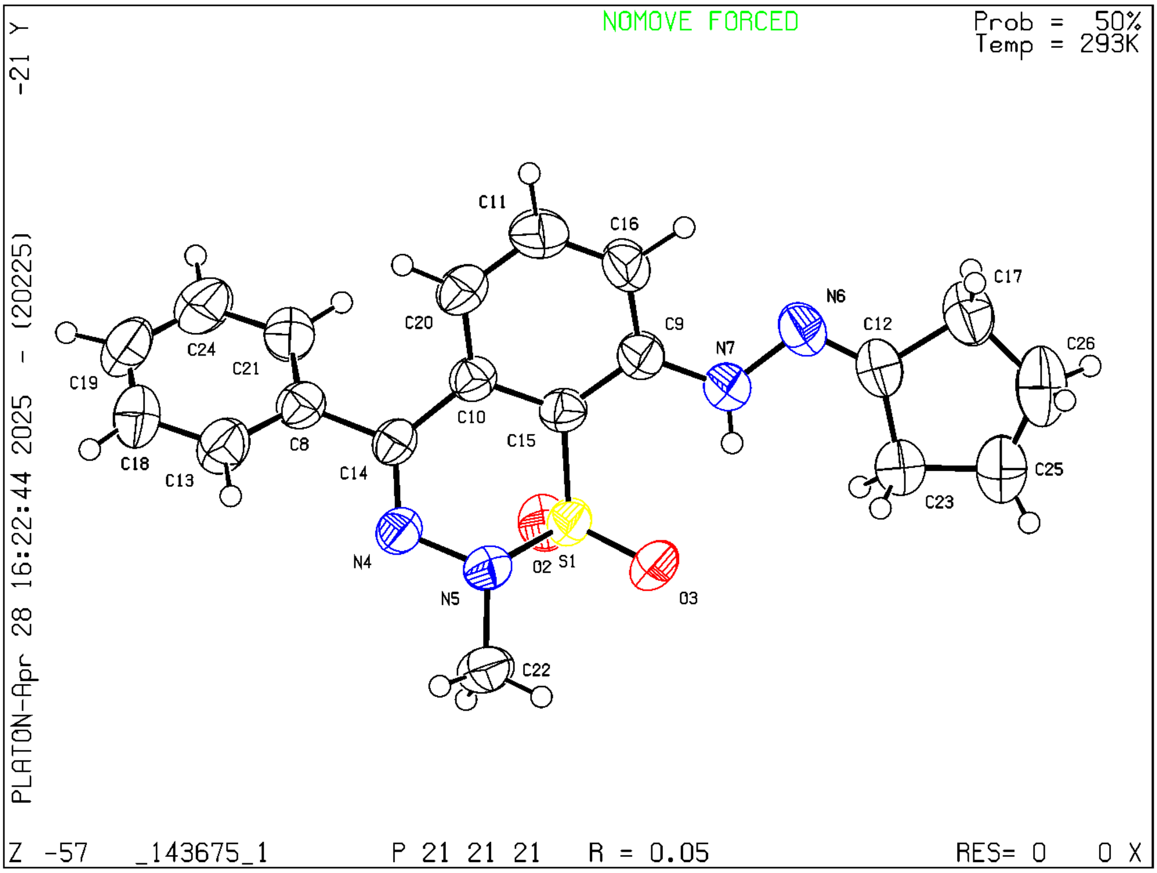

Supplement: File 2 — Crystallographic information files, checkcif and structure report files for compounds 3b, 3d, 3e, 3g, 3h, (E)-7a, 7b, 7d, 7e, (E)-7f, (Z)-7h, 7i and (E)-9a. [file 7i_checkcif.pdf › Átnevezett XRD/7i_checkcif.pdf]
